# Supplementary material for: Rhodium-catalyzed enantioselective and diastereodivergent access to diaxially chiral heterocycles
Source: Nat Commun. 2023 Aug 3;14:4661. doi: 10.1038/s41467-023-39968-3 (PMC10400608; doi:10.1038/s41467-023-39968-3)
Supplement: Supplementary file 1 — Supplementary Information [file 41467_2023_39968_MOESM1_ESM.pdf]

# Supplementary Information

## Rhodium-Catalyzed Enantioselective and Diastereodivergent Access to Diaxially Chiral Heterocycles


Yishou Wang,[1] Xiaohan Zhu,[2] Deng Pan,[3] Jierui Jing,[2] Fen Wang,[2*] Ruijie Mi,[1,2] Genping Huang,[3*] Xingwei Li[1,2*]

[1]*Institute of Molecular Science and Engineering, Institute of Frontier and Interdisciplinary Sciences, Shandong University, Qingdao 266237, China*

[2]*School of Chemistry and Chemical Engineering, Shaanxi Normal University, Xi'an 710062, China*

[3]*Department of Chemistry, School of Science and Tianjin Key Laboratory of Molecular Optoelectronic Sciences, Tianjin University, Tianjin 300072, China.*

*Emails: lixw@snnu.edu.cn, gphuang@tju.edu.cn, fenwang@snnu.edu.cn


## Table of Contents


1. Supplementary Notes ----------------------------------------------------------------------S2
2. Supplementary Methods -------------------------------------------------------------------S3
   - 2.1 Experimental Section --------------------------------------------------------------------S3
   - 2.2 Characterization (NMR, HRMS, and HPLC) Data-----------------------------------S4
   - 2.3 Reductive Cleavage of the Directing Group toward Enantiodivergence--------------S73
   - 2.4 Synthetic applications------------------------------------------------------------------------S79
   - 2.5 Determination of Rotation Barrier for Products 3, 37, 81------------------------------S86
   - 2.6 Mechanistic Studies -------------------------------------------------------------------S89
   - 2.7 X-Ray Crystallographic Data --------------------------------------------------------S102
   - 2.8 ECD spectrum and DFT calculations of N-N single-axis product -------------------S108
   - 2.9 NMR Spectra ---------------------------------------------------------------------------S109
3. Supplementary References ----------------------------------------------------------S233

# 1. Supplementary Notes

## General Information

Commercially available chemicals were obtained from Adamas, Acros Organics, Aldrich Chemical Co., Alfa Aesar and TCI and were used as received unless otherwise stated. Anhydrous solvent, purchased from Adamas, Acros Organics and J&K Chemical, were used as received. All reactions were carried out using schlenk tube at $N_2$ atmosphere unless stated otherwise. NMR Spectra were recorded on a 400 or 600 MHz NMR spectrometer in the solvent indicated. The chemical shift is given in dimensionless $\delta$ values and is frequency referenced relative to TMS in $^1$H and $^{13}$C NMR spectroscopy. HRMS data were obtained on a Thermo Scientific LTQ Orbitrap Discovery spectrometer (Bremen, Germany). Column chromatography was performed on silica gel (200-300 mesh) using ethyl acetate/hexanes. The enantiomeric excess (e.e.) of the products were determined by high-performance liquid chromatography (HPLC) with a chiral stationary phase in comparison with the authenticated racemate. All the chiral stationary phases including Chiralcel AD-H, OD-H, OD-3, OX-H, IA, IC, IE, IJ-3 and IG-H used were purchased from Daicel Chiral Technologies. Optical rotations were reported as follows: $[\alpha]_D^T$ = ($c$: g/100 mL, in $CHCl_3$).

Chiral rhodium cataysts[1] and alkyne substrates[2] were prepared according to published procedures.

## 2. Supplementary Methods

## 2.1Experimental Section

## General Synthetic Procedure

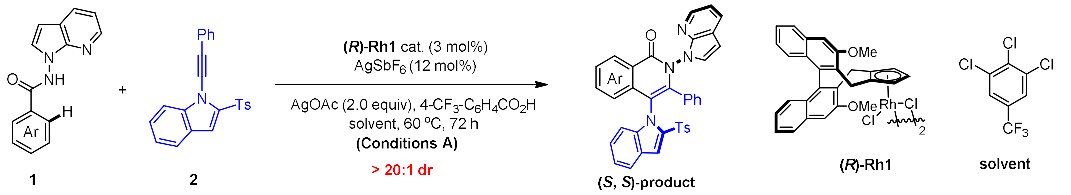


**Asymmetric Synthesis of Biaryl Products with Twofold Chiral Axes. (Conditions A).** A scew-cap vial (8 mL) was charged with N-(7-azaindol-1-yl)benzamide **1** (23.7 mg, 0.1 mmol, 1.0 equiv), 1-alkynylindole **2** (37.1 mg, 0.1 mmol, 1.0 equiv), **(*R*)-Rh-1** (3.5 mg, 3 mol%), $AgSbF_6$ (4.2 mg, 12 mol%), AgOAc (33.4 mg, 0.2 mmol, 2.0 equiv), and 4-$CF_3$-$C_6H_4CO_2H$ (19.0 mg, 0.1 mmol, 1.0 equiv). Trifluoromethyltrichlorobenzene solvent (2 mL) was then added and the mixture was stirred at 60 °C for 72 h. The reaction mixture was evaporated under vacuum and the residue was purified by preparative TLC to give the corresponding product.

**Racemic Synthesis of the Above Biaryl Products**. A similar synthetic procedure was followed for synthesis of racemic products except that racemic **Rh-1** (3 mol %) was used.

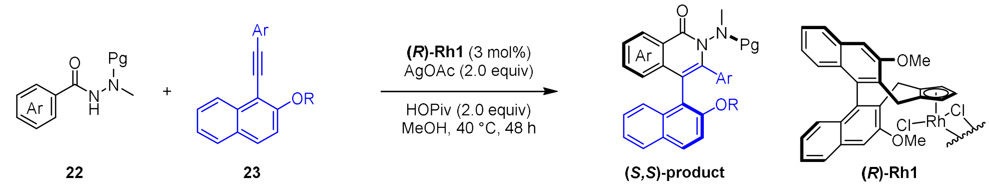


**Conditions B.** A scew-cap vial (8 mL) was charged with benzamides **22** (PG = Boc or Cbz, 0.1 mmol, 1.0 equiv), 2-substituted 1-alkynylnaphthalene **23** (0.12 mmol, 1.2 equiv), **(*R*)-Rh-1** (3 mol%), AgOAc (0.2 mmol, 2.0 equiv), HOPiv (0.2 mmol, 2.0 equiv) and MeOH (2 mL) was then added, and the mixture was stirred at 40 °C for 48 h under air. The reaction mixture was evaporated under vacuum and the residue was purified by preparative TLC to give the corresponding product. The enantiomeric excess was determined by chiral HPLC analysis.

**Racemic Synthesis of products 24-48**. A similar synthetic procedure was followed for synthesis of racemic products except that **(*rac*)-Rh-1** (3 mol%) was used at 40 °C for 24 h for most racemic products. However, $[Cp^*RhCl_2]_2$ (4 mol %) was used the synthesis of (*rac*)-**36, 37, 41, 42,** and **44-46**. In theses cases, the HPLC chromatogram of the racemic products should contain four peaks.

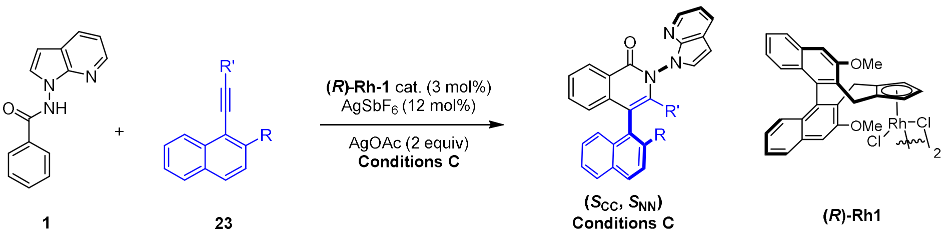


**Conditions C.** A scew-cap vial (8 mL) was charged with N-(7-azaindol-1-yl)benzamide **1** (23.7 mg, 0.1 mmol, 1.0 equiv), 2-substituted 1-alkynylnaphthalene **23** (0.1 mmol, 1.0 equiv), **(*R*)-Rh-1** (3.5 mg, 3 mol%), $AgSbF_6$ ( 4.2 mg, 12 mol%), AgOAc (33.4 mg, 0.2 mmol, 2.0 equiv), and 4-$CF_3$-$C_6H_4CO_2H$

(19.0 mg, 0.1 mmol, 1.0 equiv). DCE (1 mL) was then added, and the mixture was stirred at 60 °C for 48 h. The reaction mixture was evaporated under vacuum and the residue was purified by preparative TLC to give the corresponding product.

**Racemic Synthesis of the Above Biaryl Products**. A similar synthetic procedure was followed for synthesis of racemic products except that racemic **Rh-1** (3 mol %) was used.

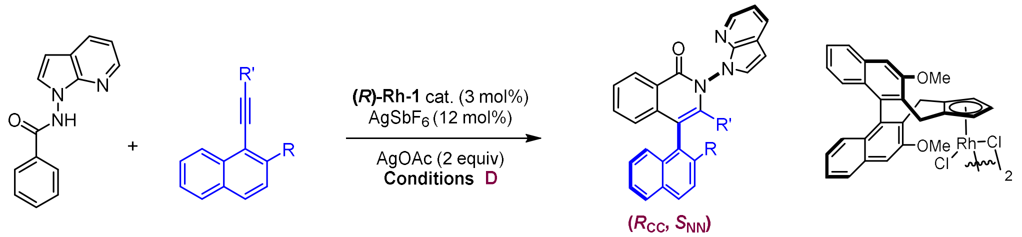


1 23 ($R_{CC}$, $S_{NN}$) Conditions D (*R*)-Rh1

**Conditions D.** A scew-cap vial (8 mL) was charged with N-(7-azaindol-1-yl)benzamide **1** (23.7 mg, 0.1 mmol, 1.0 equiv), 2-substituted 1-alkynylnaphthalene **23** (37.1 mg, 0.1 mmol, 1.0 equiv), **(*R*)-Rh-1** (3.5 mg, 3 mol%), $AgSbF_6$ ( 4.2 mg, 12 mol%), AgOAc (33.4 mg, 0.2 mmol, 2.0 equiv), and AcOH (6.0 mg, 0.1 mmol, 1.0 equiv). HFIP (1 mL) was then added and the mixture was stirred at 25 °C for 24 h. The reaction mixture was evaporated under vacuum and the residue was purified by preparative TLC to give the corresponding product.

**Racemic Synthesis of the Above Biaryl Products**. A similar synthetic procedure was followed for synthesis of racemic products except that racemic **Rh-1** (3 mol %) was used.

## 2.2 Characterization (NMR, HRMS, and HPLC) Data

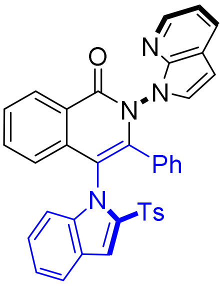


**(*S,S*)-3-phenyl-2-(1H-pyrrolo[2,3-b]pyridin-1-yl)-4-(2-tosyl-1H-indol-1-yl)isoquinolin-1(2H)-one (3).** White solid (42.4 mg, 70%), $^1$H NMR (600 MHz, $CDCl_3$) δ 8.44 (dd, *J* = 4.7, 1.5 Hz, 1H), 8.28 – 8.23 (m, 1H), 7.86 (td, *J* = 6.2, 3.2 Hz, 1H), 7.77 (dd, *J* = 7.8, 1.5 Hz, 1H), 7.71 – 7.66 (m, 2H), 7.62 (dt, *J* = 8.1, 1.0 Hz, 1H), 7.43 (d, *J* = 0.9 Hz, 1H), 7.28 – 7.23 (m, 3H), 7.15 (m, 1H), 7.11 – 7.07 (m, 2H), 7.04 (dd, *J* = 7.8, 4.8 Hz, 1H), 6.94 (ddd, *J* = 8.4, 7.2, 1.4 Hz, 1H), 6.92 – 6.88 (m, 1H), 6.84 (d, *J* = 3.8 Hz, 1H), 6.83 – 6.79 (m, 2H), 6.75 (t, *J* = 7.9 Hz, 2H), 6.27 (d, *J* = 3.8 Hz, 1H), 5.57 (dt, *J* = 8.2, 0.9 Hz, 1H), 2.08 (s, 3H).

$^{13}$C NMR (151 MHz, $CDCl_3$) δ 159.83, 146.42, 146.15, 143.80, 143.08, 140.25, 137.29, 135.29, 134.15, 132.21, 128.42, 128.39, 127.96, 127.51, 127.33, 126.77, 126.66, 126.06, 125.33, 124.05, 123.36, 121.99, 121.33, 120.96, 117.43, 116.49, 115.89, 111.89, 111.28, 110.57, 99.97, 98.52, 20.35.

**HRMS** (ESI-TOF) (m/z): Calcd for $C_{37}H_{26}N_4O_3S^+$, ([M + H]$^+$), 607.1798, found 607.1798. **$[\alpha]_D^{20}$** = -18 (c = 0.1, $CHCl_3$).

**HPLC** conditions: Daicel Chiralpak OD-3 column (80: 20 hexane: 2-propanol, 0.8 mL/min, 40 °C, 254 nm); tr (major) = 8.7 min, tr (minor) = 11.5 min, 92% e.e.. > 20:1 dr determined by crude NMR.

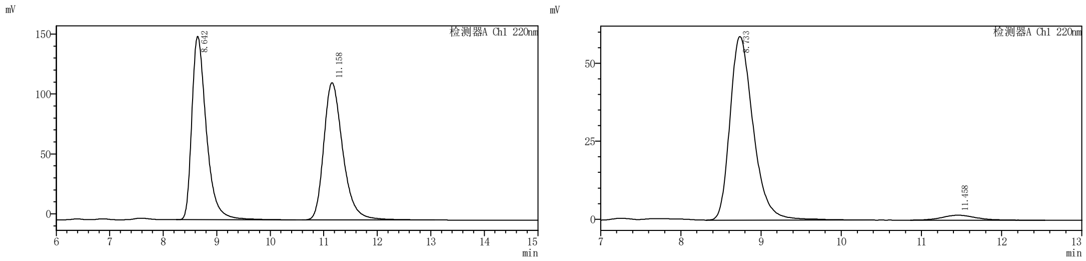


| No. | Time | Area | Area (%) | No. | Time | Area | Area (%) |
|---|---|---|---|---|---|---|---|
| 1 | 8.642 | 2888304 | 50.426 | 1 | 8.733 | 1183779 | 96.108 |
| 2 | 11.158 | 2839502 | 49.574 | 2 | 11.458 | 47945 | 3.892 |

**Supplementary Figure 1. HPLC data of 3.**

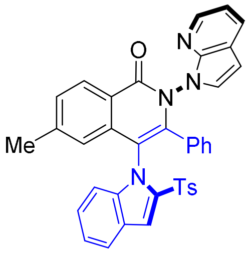


**(*S,S*)-6-methyl-3-phenyl-2-(1H-pyrrolo[2,3-b]pyridin-1-yl)-4-(2-tosyl-1H-indol-1-yl)isoquinolin-1(2H)-one (4).** White solid (39.1 mg, 63%) $^{1}$H NMR (600 MHz, $CDCl_3$) δ 8.43 (dd, $J$ = 4.9, 1.6 Hz, 1H), 8.18 (dd, $J$ = 8.3, 2.0 Hz, 1H), 7.78 (dd, $J$ = 7.9, 1.6 Hz, 1H), 7.70 – 7.64 (m, 2H), 7.62 (d, $J$ = 8.0 Hz, 1H), 7.46 (d, $J$ = 7.6 Hz, 1H), 7.41 (d, $J$ = 2.0 Hz, 1H), 7.26 (t, $J$ = 7.8 Hz, 1H), 7.14 (t, $J$ = 7.5 Hz, 1H), 7.06 - 7.10 (m, 3H), 6.95 (d, $J$ = 8.7 Hz, 1H), 6.87 (d, $J$ = 5.5 Hz, 2H), 6.85 – 6.79 (m, 2H), 6.72 (t, $J$ = 7.8 Hz, 2H), 6.27 (d, $J$ = 3.7 Hz, 1H), 5.33 (s, 1H), 2.11 (s, 3H), 1.87 (s, 3H).

$^{13}$C NMR (151 MHz, $CDCl_3$) δ 170.15, 159.82, 146.11, 145.92, 143.38, 143.12, 142.82, 140.19, 136.88, 135.56, 134.28, 128.72, 128.29, 128.17, 128.04, 127.82, 127.79, 127.62, 127.01, 125.27, 124.00, 123.44, 122.95, 121.93, 121.13, 120.98, 120.87, 117.61, 116.41, 111.67, 111.20, 110.58, 99.92, 20.66, 20.32.

**HRMS** (ESI-TOF) (m/z): Calcd for $C_{38}H_{29}N_4O_3S^+$, ([M + H]$^+$), 621.1955, found 621.1943. **$[\alpha]_D^{20}$** = -23 (c = 0.1, $CHCl_3$).

**HPLC** conditions: Daicel Chiralpak OD-3 column (80: 20 hexane: 2-propanol, 0.8 mL/min, 40 ºC, 254 nm); tr (major) = 12.2 min, tr (minor) = 14.2 min, 93% e.e.. > 20:1 dr determined by crude NMR.

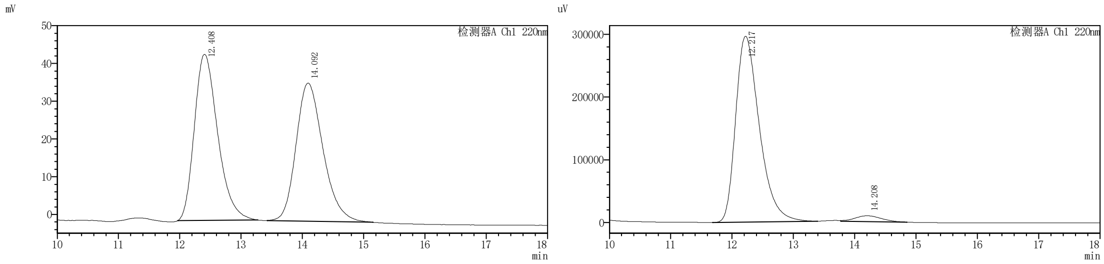


| No. | Time | Area | Area (%) | No. | Time | Area | Area (%) |
|---|---|---|---|---|---|---|---|
| 1 | 12.408 | 1169003 | 50.959 | 1 | 12.217 | 7965589 | 96.583 |
| 2 | 14.092 | 1124992 | 49.041 | 2 | 14.208 | 281791 | 3.417 |

**Supplementary Figure 2. HPLC data of 4.**

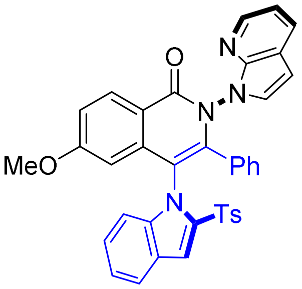

**(*S,S*)-6-methoxy-3-phenyl-2-(1H-pyrrolo[2,3-b]pyridin-1-yl)-4-(2-tosyl-1H-indol-1-yl)isoquinolin-1(2H)-one (5)** White solid (49.6 mg, 78%) $^{1}$H NMR (600 MHz, $CDCl_3$) δ 8.51 (dd, *J* = 4.7, 1.7 Hz, 1H), 8.27 (dd, *J* = 8.8, 1.6 Hz, 1H), 7.85 (dt, *J* = 7.9, 1.6 Hz, 1H), 7.78 (dd, *J* = 8.4, 1.7 Hz, 2H), 7.70 – 7.64 (m, 1H), 7.55 (d, *J* = 8.4 Hz, 1H), 7.49 (t, *J* = 1.1 Hz, 1H), 7.40 – 7.30 (m, 1H), 7.23 – 7.14 (m, 3H), 7.06 (s, 1H), 6.97 (td, *J* = 7.5, 1.4 Hz, 1H), 6.95 – 6.90 (m, 3H), 6.88 (dt, *J* = 8.9, 2.0 Hz, 1H), 6.82 (ddd, *J* = 8.7, 7.5, 1.5 Hz, 2H), 6.34 (dd, *J* = 3.8, 1.6 Hz, 1H), 4.89 (t, *J* = 2.0 Hz, 1H), 3.27 (s, 3H), 2.19 (s, 3H).

$^{13}$C NMR (151 MHz, $CDCl_3$) δ 170.15, 162.27, 159.43, 146.82, 146.14, 143.49, 143.10, 140.16, 137.07, 136.47, 135.46, 129.67, 128.62, 128.32, 128.12, 127.94, 127.02, 125.37, 124.00, 121.95, 120.95, 117.56, 116.90, 116.41, 115.15, 111.46, 111.25, 110.56, 102.78, 99.89, 53.85, 20.32.

**HRMS** (ESI-TOF) (m/z): Calcd for $C_{38}H_{29}N_4O_4S^+$, ([M + H]$^+$), 637.1904, found 637.1902. **$[\alpha]_D^{20}$** = -38 (c = 0.1, $CHCl_3$).

**HPLC** conditions: Daicel Chiralpak OD-3 column (80: 20 hexane: 2-propanol, 0.8 mL/min, 40 °C, 254 nm); tr (major) = 13.2 min, tr (minor) = 15.5 min, 90% e.e.. > 20:1 dr determined by crude NMR.

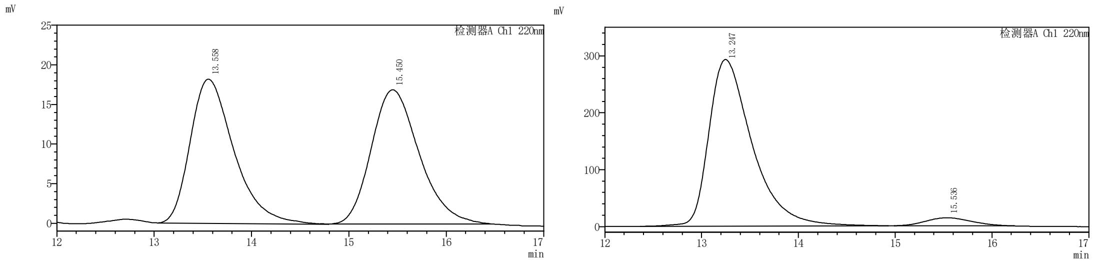

| No. | Time | Area | Area (%) | No. | Time | Area | Area (%) |
|---|---|---|---|---|---|---|---|
| 1 | 13.558 | 569974 | 49.507 | 1 | 13.247 | 9110454 | 95.166 |
| 2 | 15.450 | 581324 | 50.493 | 2 | 15.536 | 462795 | 4.834 |

**Supplementary Figure 3. HPLC data of 5.**

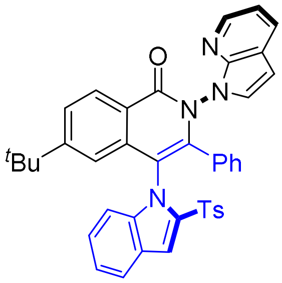

**(*S,S*)-6-(tert-butyl)-3-phenyl-2-(1H-pyrrolo[2,3-b]pyridin-1-yl)-4-(2-tosyl-1H-indol-1-yl)isoquinolin-1(2H)-one (6)**

White solid (51.0 mg, 77%) $^{1}$H NMR (600 MHz, $CDCl_3$) δ 8.45 (dd, *J* = 4.6, 1.5 Hz, 1H), 8.12 (dd, *J* = 8.4, 1.2 Hz, 1H), 7.78 - 7.81 (m, 3H), 7.64 (d, *J* = 8.1 Hz, 1H), 7.47 (s, 2H), 7.32 – 7.23 (m, 2H), 7.17 – 7.02 (m, 4H), 6.94 (td, *J* = 7.6, 1.4 Hz, 1H), 6.89 – 6.70 (m, 5H), 6.27 (dd, *J* = 3.7, 1.2 Hz, 1H), 5.37 (s, 1H), 2.03 (s, 3H), 0.82 (s, 9H).

$^{13}$C NMR (151 MHz, $CDCl_3$) δ 159.60, 155.44, 146.54, 145.82, 143.60, 142.91, 140.74, 138.25, 137.86, 135.05, 133.85, 128.56, 128.41, 128.23, 128.06, 127.05, 126.92, 125.34, 124.42, 124.08, 121.99, 121.19, 120.98, 117.56, 117.44, 116.46, 112.51, 111.38, 110.76, 99.97, 29.39, 20.33.

**HRMS** (ESI-TOF) (m/z): Calcd for $C_{41}H_{35}N_4O_3S^+$, ([M + H]$^+$), 663.2424, found 663.2432. **$[\alpha]_D^{20}$** = -36 (c = 0.1, $CHCl_3$).

**HPLC** conditions: Daicel Chiralpak OD-3 column (80: 20 hexane: 2-propanol, 0.8 mL/min, 40 °C, 254 nm); tr (major) = 8.6 min, tr (minor) = 13.1 min, 88% e.e.. > 20:1 dr determined by crude NMR.

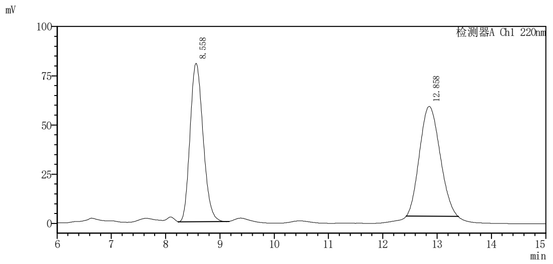

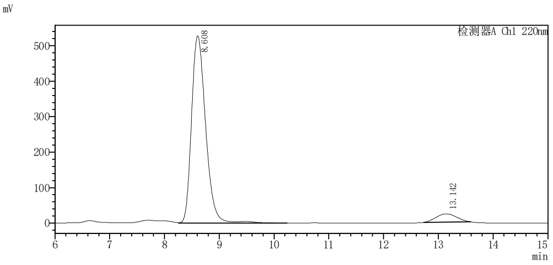

| No. | Time | Area | Area (%) | No. | Time | Area | Area (%) |
|---|---|---|---|---|---|---|---|
| 1 | 8.558 | 1342179 | 47.901 | 1 | 8.608 | 9478008 | 94.017 |
| 2 | 12.858 | 1459807 | 52.099 | 2 | 13.142 | 603132 | 5.983 |

**Supplementary Figure 4. HPLC data of 6.**

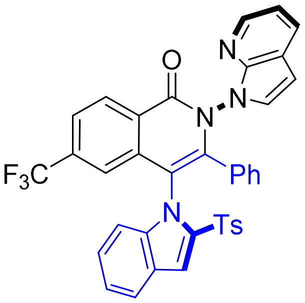


**(*S,S*)-3-phenyl-2-(1H-pyrrolo[2,3-b]pyridin-1-yl)-4-(2-tosyl-1H-indol-1-yl)-6-(trifluoromethyl)isoquinolin-1(2H)-one (7)**

White solid (31.7 mg, 47%) $^{1}$H NMR (600 MHz, $CDCl_3$) δ 8.44 (d, $J$ = 4.7 Hz, 1H), 8.40 (d, $J$ = 8.4 Hz, 1H), 7.80 (dd, $J$ = 7.9, 1.5 Hz, 1H), 7.67 (d, $J$ = 8.3 Hz, 2H), 7.63 (d, $J$ = 8.1 Hz, 1H), 7.54 – 7.41 (m, 3H), 7.31 – 7.26 (m, 1H), 7.16 (t, $J$ = 7.7 Hz, 1H), 7.12 (dd, $J$ = 7.9, 4.7 Hz, 1H), 7.06 (d, $J$ = 8.4 Hz, 1H), 7.00 – 6.89 (m, 2H), 6.87 – 6.80 (m, 3H), 6.75 (t, $J$ = 7.7 Hz, 2H), 6.29 (d, $J$ = 3.7 Hz, 1H), 5.84 (s, 1H), 2.07 (s, 3H).

$^{13}$C NMR (151 MHz, $CDCl_3$) δ 158.99, 148.00, 146.04, 143.71, 143.40, 140.16, 136.89, 135.40, 134.73, 134.02 (q, J = 32.6 Hz), 133.81, 133.59, 133.37, 128.75 (q, J = 7.7 Hz), 128.60, 127.68, 127.45, 126.54, 125.70, 125.58, 124.12, 122.65 (q, J = 273.3 Hz), 122.41, 122.25, 121.28, 118.40 (q, J = 4.5 Hz), 117.57, 116.68, 116.37, 111.89, 111.55, 110.17, 100.34, 20.22.

**HRMS** (ESI-TOF) (m/z): Calcd for $C_{38}H_{25}F_3N_4O_3S^+$, ([M + H]$^+$), 675.1672, found 675.1664. **[α]$_D^{20}$** = -27 (c = 0.1, $CHCl_3$).

**HPLC** conditions: Daicel Chiralpak OD-3 column (80: 20 hexane: 2-propanol, 0.8 mL/min, 40 ºC, 254 nm); tr (major) = 11.8 min, tr (minor) = 13.6 min, 92% e.e.. > 20:1 dr determined by crude NMR.

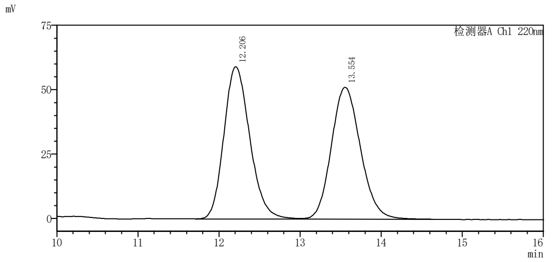

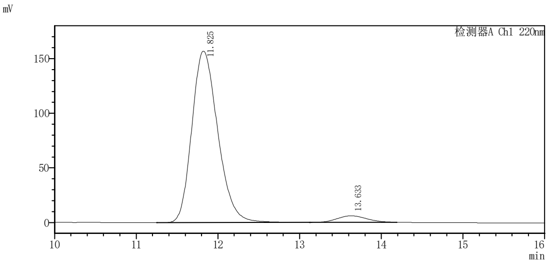

| No. | Time | Area | Area (%) | No. | Time | Area | Area (%) |
|---|---|---|---|---|---|---|---|
| 1 | 12.206 | 1318619 | 50.675 | 1 | 11.825 | 3477711 | 95.825 |
| 2 | 13.554 | 1283467 | 49.325 | 2 | 13.633 | 151534 | 4.175 |

**Supplementary Figure 5. HPLC data of 7.**

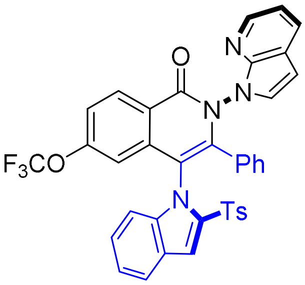


**(*S,S*)-3-phenyl-2-(1H-pyrrolo[2,3-b]pyridin-1-yl)-4-(2-tosyl-1H-indol-1-yl)-6-(trifluoromethoxy)isoquinolin-1(2H)-one (8)**

White solid (44.9 mg, 65%). [1]H NMR (600 MHz, $CDCl_3$) δ 8.44 (dt, *J* = 4.8, 1.4 Hz, 1H), 8.28 (dd, *J* = 8.8, 1.1 Hz, 1H), 7.78 (dt, *J* = 7.8, 1.3 Hz, 1H), 7.73 (d, *J* = 8.0 Hz, 2H), 7.63 (d, *J* = 8.1 Hz, 1H), 7.44 (s, 2H), 7.28 (dd, *J* = 8.4, 7.1 Hz, 1H), 7.20 – 7.14 (m, 1H), 7.13 – 7.05 (m, 3H), 6.99 (s, 1H), 6.95 – 6.87 (m, 3H), 6.82 (dd, *J* = 3.8, 1.0 Hz, 1H), 6.76 (t, *J* = 7.7 Hz, 2H), 6.28 (dd, *J* = 3.8, 1.1 Hz, 1H), 5.29 (d, *J* = 2.4 Hz, 1H), 2.10 (s, 3H).

[13]C NMR (151 MHz, $CDCl_3$) δ 159.95, 152.75, 149.02, 147.37, 144.87, 144.34, 141.14, 138.04, 137.38, 136.32, 131.20, 129.96, 129.69, 129.61, 129.53, 129.45, 128.87, 128.64, 127.63, 126.68, 125.62, 125.15, 123.24, 122.37, 122.28 (q, *J* = 258.5 Hz), 118.79, 118.52, 117.68, 112.88, 112.85, 112.28, 111.29, 101.28, 21.26.

**HRMS** (ESI-TOF) (m/z): Calcd for $C_{38}H_{26}F_3N_4O_4S^+$, ([M + H]$^+$), 691.1621, found 691.1623. $[\alpha]_D^{20}$ = -44 (c = 0.1, $CHCl_3$).

**HPLC** conditions: Daicel Chiralpak OD-3 column (80: 20 hexane: 2-propanol, 1.0 mL/min, 40 ºC, 254 nm); tr (major) = 10.0 min, tr (minor) = 12.6 min, 92% e.e.. > 20:1 dr determined by crude NMR.

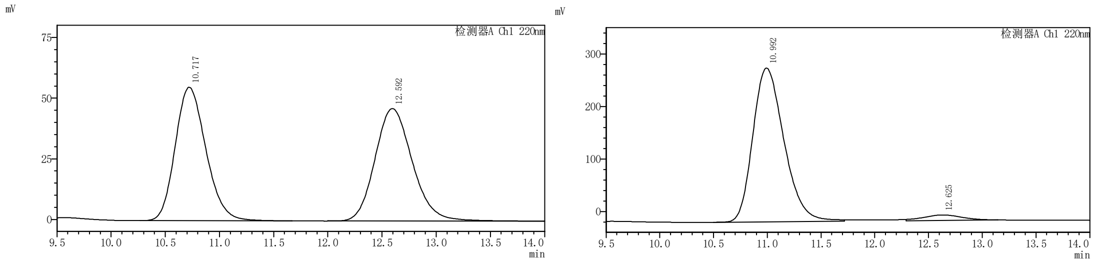


| No. | Time | Area | Area (%) | No. | Time | Area | Area (%) |
|---|---|---|---|---|---|---|---|
| 1 | 10.717 | 1086273 | 49.364 | 1 | 10.992 | 5916671 | 95.794 |
| 2 | 12.592 | 1114274 | 50.636 | 2 | 12.625 | 259811 | 4.206 |

**Supplementary Figure 6. HPLC data of 8.**

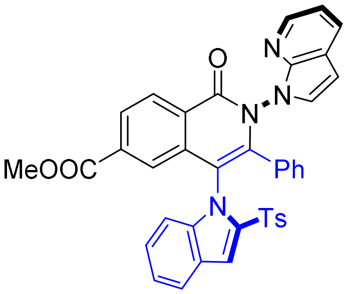


**(*S,S*)-methyl 1-oxo-3-phenyl-2-(1H-pyrrolo[2,3-b]pyridin-1-yl)-4-(2-tosyl-1H-indol-1-yl)-1,2-dihydroisoquinoline-6-carboxylate (9)**

White solid (34.5 mg, 52%) [1]H NMR (600 MHz, $CDCl_3$) δ 8.51 (dd, *J* = 4.8, 1.5 Hz, 1H), 8.44 (d, *J* = 8.3 Hz, 1H), 7.95 (dd, *J* = 8.3, 1.6 Hz, 1H), 7.84 (dd, *J* = 7.8, 1.5 Hz, 1H), 7.69 (dt, *J* = 8.0, 1.0 Hz, 1H), 7.67 – 7.63 (m, 2H), 7.58 – 7.48 (m, 2H), 7.33 (ddd, *J* = 8.3, 7.1, 1.2 Hz, 1H), 7.22 (ddd, *J* = 8.1, 7.0, 0.9 Hz, 1H), 7.18 – 7.11 (m, 2H), 7.06 – 6.98 (m, 1H), 6.98 – 6.93 (m, 2H), 6.85 – 6.76 (m, 4H), 6.40 – 6.33 (m, 2H), 3.76 (s, 3H), 2.14 (s, 3H).

[13]C NMR (151 MHz, $CDCl_3$) δ 165.16, 160.41, 148.20, 147.07, 144.82, 144.03, 141.25, 137.66, 136.75, 135.39, 134.30, 129.56, 129.51, 129.33, 128.96, 128.83, 128.67, 128.59, 127.64, 127.25, 127.08, 126.52, 125.17, 125.03, 124.00, 123.17, 122.11, 118.49, 117.61, 112.89, 112.85, 111.32, 101.17, 52.42, 21.27.

**HRMS** (ESI-TOF) (m/z): Calcd for $C_{39}H_{29}N_4O_5S^+$, ([M + H]$^+$), 665.1853, found 665.1835. $[\alpha]_D^{20}$ = -20 (c = 0.1, $CHCl_3$).

**HPLC** conditions: Daicel Chiralpak OD-3 column (80: 20 hexane: 2-propanol, 0.8 mL/min, 40 °C, 254 nm); tr (major) = 18.8 min, tr (minor) = 16.4 min, 90% e.e.. > 20:1 dr determined by crude NMR.

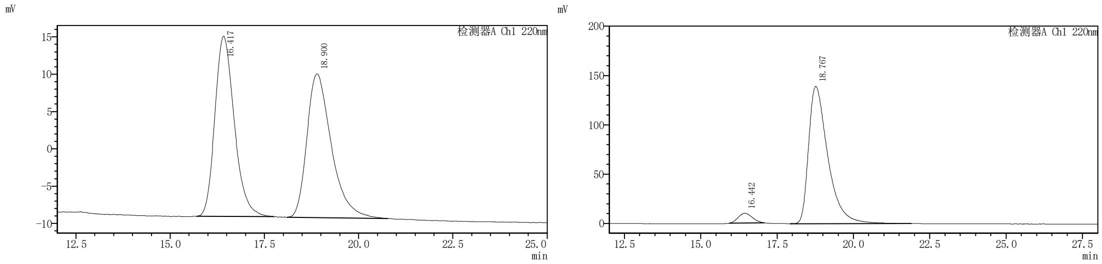


| No. | Time | Area | Area (%) | No. | Time | Area | Area (%) |
|---|---|---|---|---|---|---|---|
| 1 | 16.417 | 867080 | 49.895 | 1 | 16.442 | 313163 | 4.870 |
| 2 | 18.900 | 870724 | 50.105 | 2 | 18.767 | 6117090 | 95.130 |

**Supplementary Figure 7. HPLC data of 9.**

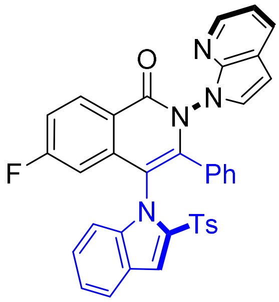


**(*S,S*)-6-fluoro-3-phenyl-2-(1H-pyrrolo[2,3-b]pyridin-1-yl)-4-(2-tosyl-1H-indol-1-yl)isoquinolin-1(2H)-one (10)**

White solid (43.7 mg, 70%) $^{1}$H NMR (600 MHz, $CDCl_3$) δ 8.52 (dd, $J$ = 4.8, 1.5 Hz, 1H), 8.37 (dd, $J$ = 8.8, 5.5 Hz, 1H), 7.85 (dd, $J$ = 7.8, 1.5 Hz, 1H), 7.76 – 7.72 (m, 2H), 7.69 (dt, $J$ = 8.1, 1.0 Hz, 1H), 7.58 – 7.46 (m, 2H), 7.35 (ddd, $J$ = 8.3, 7.1, 1.2 Hz, 1H), 7.23 (ddd, $J$ = 8.0, 7.0, 1.0 Hz, 1H), 7.19 – 7.14 (m, 2H), 7.03 (tt, $J$ = 8.5, 4.3 Hz, 2H), 6.99 – 6.89 (m, 4H), 6.81 (td, $J$ = 7.5, 1.3 Hz, 2H), 6.34 (d, $J$ = 3.8 Hz, 1H), 5.25 (dd, $J$ = 9.5, 2.4 Hz, 1H), 2.20 (s, 3H).

$^{13}$C NMR (151 MHz, $CDCl_3$) δ 166.45 (d, $J$ = 254.3 Hz), 160.15, 148.83, 147.25, 144.84, 144.32, 141.01, 138.13, 137.78, 136.53, 131.90 (d, $J$ = 15 Hz), 129.55, 129.44, 128.80, 128.70, 127.69, 126.56, 125.12, 123.16, 122.17, 121.88, 118.47, 117.60, 115.86 (d, $J$ = 23.2 Hz), 112.69, 112.18, 111.32, 108.12 (d, $J$ = 23.7 Hz), 101.12, 21.31.

**HRMS** (ESI-TOF) (m/z): Calcd for $C_{37}H_{26}FN_4O_3S^+$, ([M + H]$^+$), 625.1704, found 625.1707. **$[\alpha]_D^{20}$** = -27 (c = 0.1, $CHCl_3$).

**HPLC** conditions: Daicel Chiralpak OD-3 column (80: 20 hexane: 2-propanol, 0.8 mL/min, 40 °C, 254 nm); tr (major) = 11.9 min, tr (minor) = 14.2 min, 94% e.e.. > 20:1 dr determined by crude NMR.

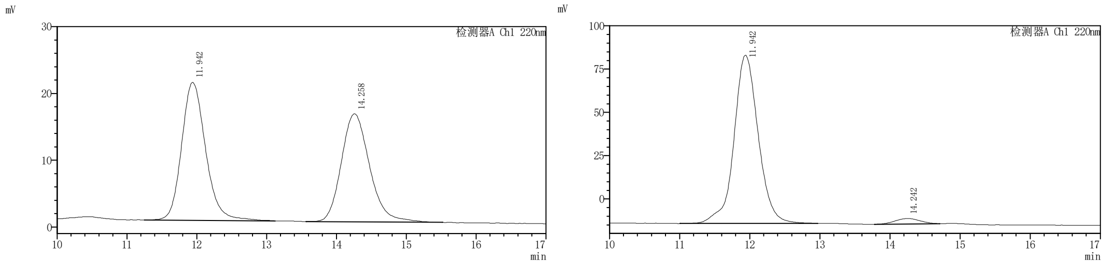


| No. | Time | Area | Area (%) | No. | Time | Area | Area (%) |
|---|---|---|---|---|---|---|---|
| 1 | 11.942 | 494089 | 52.042 | 1 | 11.942 | 2342771 | 96.833 |
| 2 | 14.258 | 455320 | 47.958 | 2 | 14.242 | 76623 | 3.167 |

**Supplementary Figure 8. HPLC data of 10.**

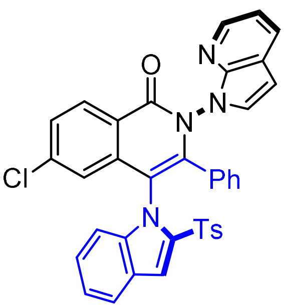


**(*S,S*)-6-chloro-3-phenyl-2-(1H-pyrrolo[2,3-b]pyridin-1-yl)-4-(2-tosyl-1H-indol-1-yl)isoquinolin-1(2H)-one (11)**

White solid (42.2 mg, 66%) $^1$H NMR (600 MHz, $CDCl_3$) δ 8.49 – 8.40 (m, 1H), 8.22 (d, *J* = 8.5 Hz, 1H), 7.80 (d, *J* = 7.4 Hz, 1H), 7.69 – 7.65 (m, 2H), 7.63 (dd, *J* = 8.1, 0.9 Hz, 1H), 7.50 – 7.43 (m, 1H), 7.42 (s, 1H), 7.32 – 7.25 (m, 1H), 7.24 – 7.15 (m, 3H), 7.13 – 7.04 (m, 2H), 6.84 - 6.94 (m, 4H), 6.85 (d, *J* = 3.6 Hz, 1H), 6.73 (t, *J* = 7.6 Hz, 2H), 6.28 (d, *J* = 3.4 Hz, 1H), 5.49 (d, *J* = 1.9 Hz, 1H), 2.15 (s, 3H).

$^{13}$C NMR (151 MHz, $CDCl_3$) δ 160.28, 148.80, 146.83, 144.51, 144.33, 141.04, 140.37, 137.74, 136.67, 136.39, 130.33, 129.91, 129.59, 129.53, 128.69, 128.65, 127.84, 127.82, 127.40, 126.59, 125.10, 123.17, 122.66, 122.17, 121.76, 118.67, 117.62, 112.62, 111.85, 111.32, 101.24, 21.44.

**HRMS** (ESI-TOF) (m/z): Calcd for $C_{37}H_{26}ClN_4O_3S^+$, ([M + H]$^+$), 641.1402, found 641.1402. **[α]$_D^{20}$** = -29 (c = 0.1, $CHCl_3$).

**HPLC** conditions: Daicel Chiralpak IA column (80: 20 hexane: 2-propanol, 0.8 mL/min, 40 °C, 254 nm); tr (major) = 19.3 min, tr (minor) = 16.7 min, 96% e.e.. > 20:1 dr determined by crude NMR.

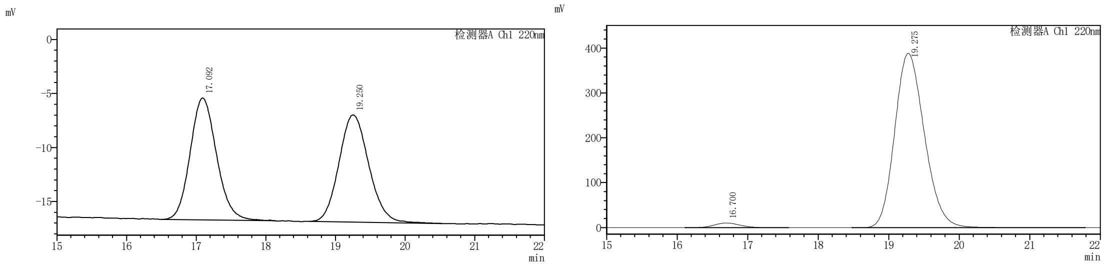


| No. | Time | Area | Area (%) | No. | Time | Area | Area (%) |
|---|---|---|---|---|---|---|---|
| 1 | 17.092 | 298298 | 50.192 | 1 | 16.700 | 274099 | 2.343 |
| 2 | 19.250 | 296013 | 49.808 | 2 | 19.275 | 11424021 | 97.657 |

**Supplementary Figure 9. HPLC data of 11.**

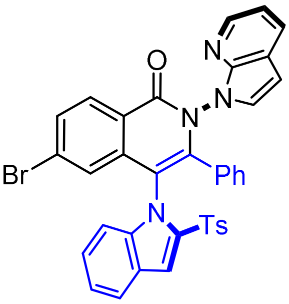


**(*S,S*)-6-bromo-3-phenyl-2-(1H-pyrrolo[2,3-b]pyridin-1-yl)-4-(2-tosyl-1H-indol-1-yl)isoquinolin-1(2H)-one (12)**

White solid (28.7 mg, 42%) $^1$H NMR (600 MHz, $CDCl_3$) δ 8.43 (dt, *J* = 4.8, 1.4 Hz, 1H), 8.13 (dd, *J* = 8.5, 1.3 Hz, 1H), 7.77 (dt, *J* = 7.8, 1.4 Hz, 1H), 7.66 (dd, *J* = 8.2, 1.4 Hz, 2H), 7.62 (d, *J* = 8.1 Hz, 1H), 7.51 – 7.44 (m, 1H), 7.42 (d, *J* = 1.4 Hz, 1H), 7.36 (dt, *J* = 8.5, 1.6 Hz, 1H), 7.29 (ddd, *J* = 8.4, 7.0, 1.3 Hz, 1H), 7.18 – 7.13 (m, 1H), 7.13 – 7.04 (m, 2H), 6.97 – 6.87 (m, 4H), 6.84 (dd, *J* = 3.8, 1.2 Hz, 1H), 6.73 (t, *J* = 7.7 Hz, 2H), 6.27 (dd, *J* = 3.8, 1.3 Hz, 1H), 5.68 (t, *J* = 1.6 Hz, 1H), 2.16 (s, 3H).

$^{13}$C NMR (151 MHz, $CDCl_3$) δ 160.45, 148.87, 147.16, 144.85, 144.34, 141.03, 137.70, 136.68, 136.39, 130.66, 130.22, 129.59, 129.58, 129.28, 128.62, 127.63, 127.39, 126.59, 125.64, 125.62, 125.09, 124.82, 123.16, 123.00, 122.17, 118.48, 117.62, 112.64, 111.70, 111.32, 101.17, 21.53.

**HRMS** (ESI-TOF) (m/z): Calcd for $C_{37}H_{26}BrN_4O_3S^+$, ([M + H]$^+$), 685.0904, found 685.0888. **[α]$_D^{20}$** = -29 (c = 0.1, $CHCl_3$).

**HPLC** conditions: Daicel Chiralpak OD-3 column (80: 20 hexane: 2-propanol, 0.8 mL/min, 40 °C, 254 nm); tr (major) = 13.9 min, tr (minor) = 15.4 min, 99% e.e.. > 20:1 dr determined by crude NMR.

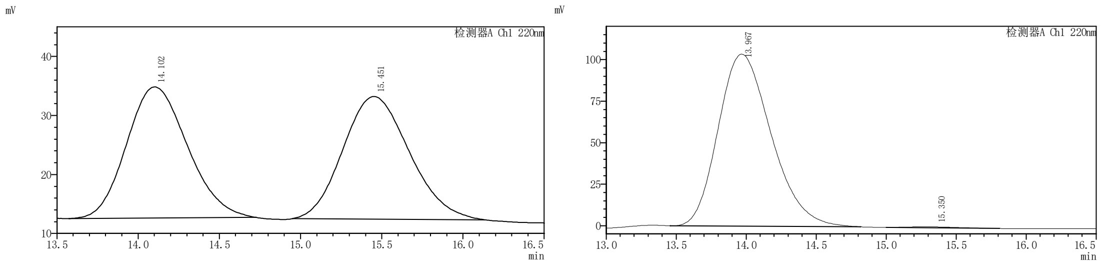


| No. | Time | Area | Area (%) | No. | Time | Area | Area (%) |
|---|---|---|---|---|---|---|---|
| 1 | 14.102 | 580836 | 49.521 | 1 | 13.967 | 2732255 | 99.493 |
| 2 | 15.451 | 592082 | 50.479 | 2 | 15.350 | 13935 | 0.507 |

**Supplementary Figure 10. HPLC data of 12.**

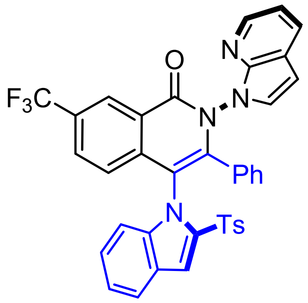


**(*S,S*)-3-phenyl-2-(1H-pyrrolo[2,3-b]pyridin-1-yl)-4-(2-tosyl-1H-indol-1-yl)-7-(trifluoromethyl)isoquinolin-1(2H)-one (13)**

White solid (36.4 mg, 54%) $^1$H NMR (600 MHz, $CDCl_3$) δ 8.59 (s, 1H), 8.51 (dd, $J$ = 4.7, 1.5 Hz, 1H), 7.86 (dt, $J$ = 7.8, 1.4 Hz, 1H), 7.80 – 7.73 (m, 2H), 7.72 (d, $J$ = 8.1 Hz, 1H), 7.61 – 7.48 (m, 2H), 7.35 (tt, $J$ = 7.0, 1.3 Hz, 1H), 7.24 (d, $J$ = 7.3 Hz, 2H), 7.21 – 7.15 (m, 3H), 7.14 – 7.05 (m, 1H), 7.00 (td, $J$ = 7.5, 1.3 Hz, 1H), 6.94 – 6.89 (m, 3H), 6.85 (d, $J$ = 10.8 Hz, 2H), 6.37 (dd, $J$ = 3.8, 1.2 Hz, 1H), 5.71 (d, $J$ = 8.5 Hz, 1H), 2.16 (s, 3H).

$^{13}$C NMR (151 MHz, $CDCl_3$) δ 159.08, 148.72, 146.28, 143.90, 143.53, 140.08, 137.06, 136.78, 135.37, 134.22, 128.72, 128.56, 128.46, 128.15, 127.94, 127.67, 127.48, 126.47, 126.31, 125.65, 124.87, 124.60 (q, $J$ = 255.6 Hz), 124.12, 123.29, 122.20, 122.19, 121.26, 117.47, 116.70, 111.78, 111.26, 110.27, 100.36, 20.07.

**HRMS** (ESI-TOF) (m/z): Calcd for $C_{38}H_{26}F_3N_4O_3S^+$, ([M + H]$^+$), 675.1672, found 675.1681. **$[\alpha]_D^{20}$** = -18 (c = 0.1, $CHCl_3$).

**HPLC** conditions: Daicel Chiralpak OD-H column (90: 10 hexane: 2-propanol, 0.8 mL/min, 40 °C, 254 nm); tr (major) = 10.1 min, tr (minor) = 6.8 min, 91% e.e.. > 20:1 dr determined by crude NMR.

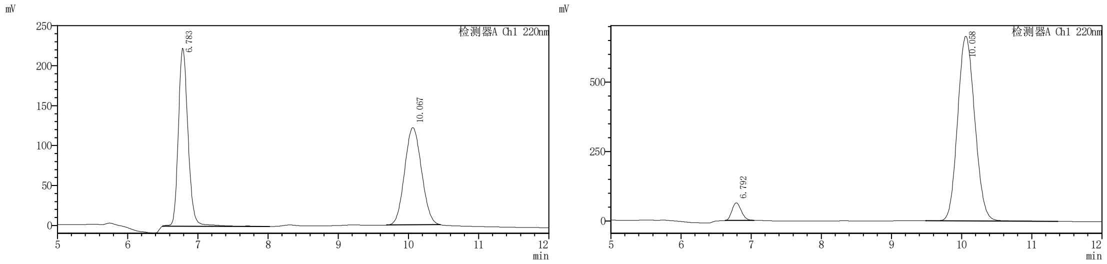


| No. | Time | Area | Area (%) | No. | Time | Area | Area (%) |
|---|---|---|---|---|---|---|---|
| 1 | 6.783 | 2138228 | 51.170 | 1 | 6.792 | 579389 | 4.862 |
| 2 | 10.067 | 2040410 | 48.830 | 2 | 10.058 | 11337028 | 95.138 |

**Supplementary Figure 11. HPLC data of 13.**

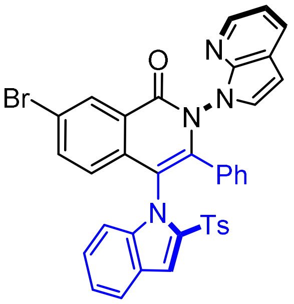

**(*S,S*)-7-bromo-3-phenyl-2-(1H-pyrrolo[2,3-b]pyridin-1-yl)-4-(2-tosyl-1H-indol-1-yl)isoquinolin-1(2H)-one (14)**

White solid (41.7 mg, 61%) $^{1}$H NMR (600 MHz, $CDCl_3$) δ 8.44 (dd, *J* = 4.8, 1.7 Hz, 1H), 8.38 (d, *J* = 2.1 Hz, 1H), 7.80 (dt, *J* = 7.8, 1.7 Hz, 1H), 7.69 (dd, *J* = 8.5, 2.1 Hz, 2H), 7.63 (dd, *J* = 8.1, 2.3 Hz, 1H), 7.44 (d, *J* = 2.3 Hz, 2H), 7.27 (td, *J* = 7.6, 6.8, 1.4 Hz, 1H), 7.16 (td, *J* = 7.6, 2.2 Hz, 1H), 7.12 (dd, *J* = 7.8, 4.7 Hz, 1H), 7.08 (dd, *J* = 8.4, 2.3 Hz, 1H), 7.05 – 6.96 (m, 2H), 6.87 - 6.93 (m, 3H), 6.83 (t, *J* = 3.1 Hz, 1H), 6.76 (d, *J* = 8.1 Hz, 2H), 6.29 (d, *J* = 3.7 Hz, 1H), 5.37 (dd, *J* = 8.7, 2.1 Hz, 1H), 2.20 (s, 3H).

$^{13}$C NMR (151 MHz, $CDCl_3$) δ 159.70, 147.81, 147.10, 144.72, 144.70, 141.13, 138.23, 136.39, 136.29, 134.03, 130.81, 129.73, 129.60, 129.51, 129.03, 128.70, 127.65, 127.38, 126.77, 126.55, 125.68, 125.09, 124.05, 123.15, 122.17, 121.35, 118.58, 117.65, 112.56, 112.52, 111.37, 101.26, 21.53.

**HRMS** (ESI-TOF) (m/z): Calcd for $C_{37}H_{26}BrN_4O_3S^+$, ([M + H]$^+$), 685.0904, found 685.0902 **[α]$_D^{20}$** = -25 (c = 0.1, $CHCl_3$).

**HPLC** conditions: Daicel Chiralpak IC column (80: 20 hexane: 2-propanol, 0.8 mL/min, 40 °C, 254 nm); tr (major) = 17.1 min, tr (minor) = 31.1 min, 91% e.e.. > 20:1 dr determined by crude NMR.

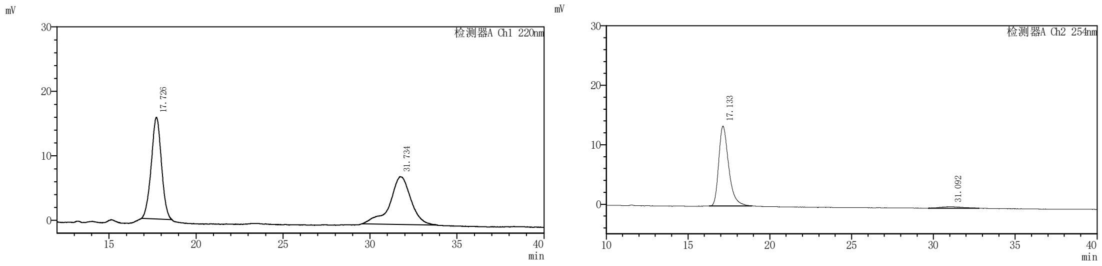


| No. | Time | Area | Area (%) | No. | Time | Area | Area (%) |
|---|---|---|---|---|---|---|---|
| 1 | 17.726 | 644204 | 50.854 | 1 | 17.133 | 566274 | 95.977 |
| 2 | 31.734 | 622570 | 49.146 | 2 | 31.092 | 23734 | 4.023 |

**Supplementary Figure 12. HPLC data of 14.**

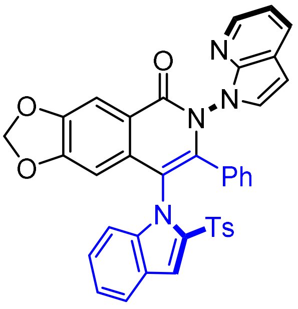

**(*S,S*)-7-phenyl-6-(1H-pyrrolo[2,3-b]pyridin-1-yl)-8-(2-tosyl-1H-indol-1-yl)-[1,3]dioxolo[4,5-g]isoquinolin-5(6H)-one (15)**

White solid (20.8 mg, 32%) $^{1}$H NMR (600 MHz, $CDCl_3$) δ 8.50 (dd, *J* = 4.8, 1.5 Hz, 1H), 7.84 (dd, *J* = 7.8, 1.5 Hz, 1H), 7.83 – 7.78 (m, 2H), 7.69 (dd, *J* = 8.1, 1.2 Hz, 1H), 7.67 (s, 1H), 7.50 (d, *J* = 0.9 Hz, 2H), 7.35 (ddd, *J* = 8.3, 7.0, 1.2 Hz, 1H), 7.22 (ddd, *J* = 8.0, 7.0, 0.9 Hz, 1H), 7.19 – 7.14 (m, 2H), 7.10 – 7.03 (m, 1H), 7.00 (d, *J* = 8.1 Hz, 2H), 6.96 (tt, *J* = 7.5, 1.3 Hz, 1H), 6.92 (d, *J* = 3.8 Hz, 1H), 6.81 (t, *J* = 7.7 Hz, 2H), 6.34 (d, *J* = 3.8 Hz, 1H), 5.97 (d, *J* = 1.1 Hz, 1H), 5.89 (d, *J* = 1.1 Hz, 1H), 4.87 (s, 1H), 2.25 (s, 3H).

$^{13}$C NMR (151 MHz, $CDCl_3$) δ 160.06, 152.45, 148.09, 147.45, 146.02, 144.85, 144.02, 141.08, 138.07, 136.47, 133.22, 129.38, 129.33, 129.31, 129.13, 129.07, 127.81, 126.42, 125.05, 123.08, 122.04, 120.00, 118.43, 117.48, 112.54, 112.41, 111.49, 106.25, 102.03, 100.95, 100.93, 21.36.

**HRMS** (ESI-TOF) (m/z): Calcd for $C_{38}H_{28}N_4O_5S^+$, ([M + H]$^+$), 651.1697, found 651.1691. **[α]$_D^{20}$** = -44 (c = 0.1, $CHCl_3$).

**HPLC** conditions: Daicel Chiralpak OD-3 column (80: 20 hexane: 2-propanol, 0.8 mL/min, 40 °C, 254 nm); tr (major) = 8.2 min, tr (minor) = 10.8 min, 93% e.e.. > 20:1 dr determined by crude NMR.

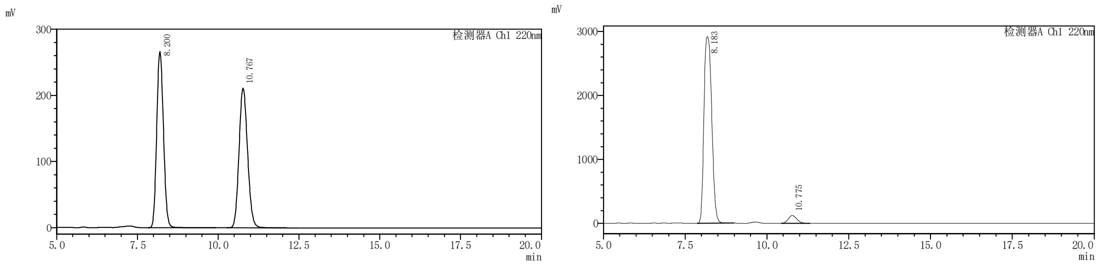


| No. | Time | Area | Area (%) | No. | Time | Area | Area (%) |
|---|---|---|---|---|---|---|---|
| 1 | 8.200 | 3882009 | 49.839 | 1 | 8.183 | 62977293 | 96.599 |
| 2 | 10.767 | 3907118 | 50.161 | 2 | 10.775 | 2217267 | 3.401 |

**Supplementary Figure 13. HPLC data of 15.**

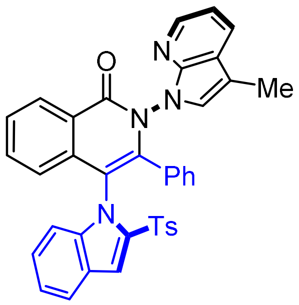


**(*S,S*)-2-(3-methyl-1H-pyrrolo[2,3-b]pyridin-1-yl)-3-phenyl-4-(2-tosyl-1H-indol-1-yl)isoquinolin-1(2H)-one (16)**

White solid (47.8 mg, 77%) $^{1}$H NMR (600 MHz, $CDCl_3$) δ 8.51 (dd, *J* = 4.7, 1.6 Hz, 1H), 8.32 (dd, *J* = 8.0, 1.5 Hz, 1H), 7.80 (dt, *J* = 7.8, 1.5 Hz, 1H), 7.73 – 7.67 (m, 3H), 7.64 – 7.55 (m, 1H), 7.48 (s, 1H), 7.36 – 7.30 (m, 2H), 7.22 (dd, *J* = 8.3, 6.9 Hz, 1H), 7.18 – 7.13 (m, 2H), 7.03 (ddd, *J* = 8.5, 7.2, 1.5 Hz, 2H), 6.97 (td, *J* = 7.5, 1.4 Hz, 1H), 6.89 – 6.77 (m, 4H), 6.68 (t, *J* = 1.4 Hz, 1H), 5.69 (d, *J* = 8.2 Hz, 1H), 2.14 (s, 3H), 2.13 (s, 3H). $^{13}$C NMR (151 MHz, $CDCl_3$) δ 161.00, 148.00, 147.28, 144.51, 144.04, 141.33, 138.27, 136.41, 135.20, 133.67, 133.15, 131.56, 130.50, 129.42, 129.30, 129.12, 128.90, 128.78, 128.34, 127.59, 127.03, 126.31, 125.04, 124.43, 122.98, 122.36, 121.95, 119.58, 116.91, 112.86, 112.27, 111.62, 110.70, 21.36, 9.64.
**HRMS** (ESI-TOF) (m/z): Calcd for $C_{38}H_{29}N_4O_3S^+$, ([M + H]$^+$), 621.1955, found 621.1943. **$[\alpha]_D^{20}$** = -27 (c = 0.1, $CHCl_3$).

**HPLC** conditions: Daicel Chiralpak OD-3 column (80: 20 hexane: 2-propanol, 0.8 mL/min, 40 °C, 254 nm); tr (major) = 7.9 min, tr (minor) = 12.2 min, 88% e.e.. > 20:1 dr determined by crude NMR.

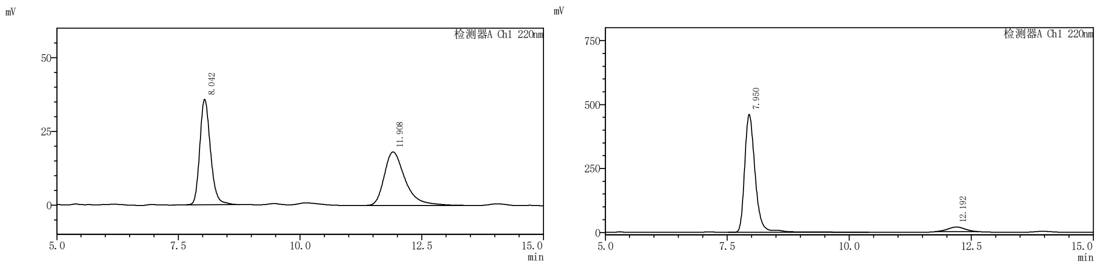


| No. | Time | Area | Area (%) | No. | Time | Area | Area (%) |
|---|---|---|---|---|---|---|---|
| 1 | 8.042 | 547217 | 50.154 | 1 | 7.950 | 6924822 | 94.030 |
| 2 | 11.908 | 543866 | 49.846 | 2 | 12.192 | 439638 | 5.970 |

**Supplementary Figure 14. HPLC data of 16.**

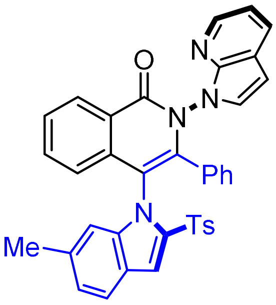


**(*S,S*)-4-(6-methyl-2-tosyl-1H-indol-1-yl)-3-phenyl-2-(1H-pyrrolo[2,3-b]pyridin-1-yl)isoquinolin-1(2H)-one (17)**

White solid (37.2 mg, 60%) $^{1}$H NMR (600 MHz, $CDCl_3$) δ 8.51 (dd, *J* = 4.7, 1.5 Hz, 1H), 8.33 (dd, *J* = 8.0, 1.3 Hz, 1H), 7.85 (dd, *J* = 7.8, 1.5 Hz, 1H), 7.77 – 7.70 (m, 2H), 7.63 – 7.49 (m, 2H), 7.45 (s, 1H), 7.36 – 7.30 (m, 1H), 7.17 (dd, *J* = 7.8, 4.7 Hz, 1H), 7.09 – 7.00 (m, 3H), 6.98 (tt, *J* = 7.6, 1.4 Hz, 1H), 6.94 – 6.91 (m, 2H), 6.88 (d, *J* = 8.1 Hz, 2H), 6.83 (s, 2H), 6.35 (d, *J* = 3.8 Hz, 1H), 5.66 (dd, *J* = 8.3, 1.0 Hz, 1H), 2.42 (s, 3H), 2.15 (s, 3H).

$^{13}$C NMR (151 MHz, $CDCl_3$) δ 173.44, 169.87, 160.85, 147.48, 147.08, 144.83, 143.93, 141.84, 137.52, 136.85, 136.51, 135.24, 133.23, 130.12, 129.40, 129.37, 129.07, 128.91, 128.33, 127.79, 127.02, 124.40, 124.07, 122.96, 122.58, 122.50, 118.45, 117.49, 112.99, 112.39, 111.14, 100.97, 22.17, 21.36.

**HRMS** (ESI-TOF) (m/z): Calcd for $C_{38}H_{29}N_4O_3S^+$, ([M + H]$^+$), 621.1955, found 621.1674. **$[\alpha]_D^{20}$** = -27 (c = 0.1, $CHCl_3$).

**HPLC** conditions: Daicel Chiralpak IC column (60: 40 hexane: 2-propanol, 0.8 mL/min, 40 °C, 254 nm); tr (major) = 11.6 min, tr (minor) = 16.7 min, 90% e.e.. > 20:1 dr determined by crude NMR.

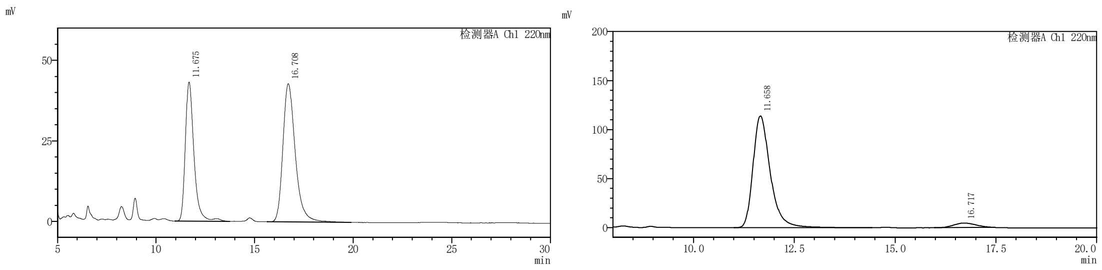


| No. | Time | Area | Area (%) | No. | Time | Area | Area (%) |
|---|---|---|---|---|---|---|---|
| 1 | 11.675 | 1322680 | 47.316 | 1 | 11.658 | 3416558 | 95.009 |
| 2 | 16.708 | 1500692 | 53.684 | 2 | 16.717 | 179465 | 4.991 |

**Supplementary Figure 15. HPLC data of 17.**

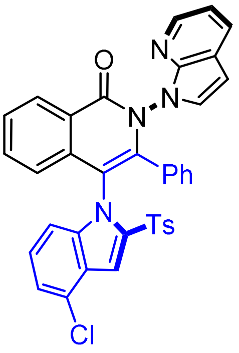


**(*S,S*)-4-(4-chloro-2-tosyl-1H-indol-1-yl)-3-phenyl-2-(1H-pyrrolo[2,3-b]pyridin-1-yl)isoquinolin-1(2H)-one (18)**

White solid (36.5 mg, 57%) $^{1}$H NMR (600 MHz, $CDCl_3$) δ 8.52 (dd, *J* = 4.7, 1.5 Hz, 1H), 8.35 (dd, *J* = 8.0, 1.3 Hz, 1H), 8.19 – 8.12 (m, 1H), 7.86 (dd, *J* = 7.9, 1.5 Hz, 1H), 7.79 – 7.73 (m, 2H), 7.71 (d, *J* = 8.1 Hz, 1H), 7.60 (d, *J* = 0.9 Hz, 1H), 7.35 (ddd, *J* = 8.1, 7.2, 1.1 Hz, 1H), 7.25 – 7.20 (m, 2H), 7.18 (dd, *J* = 7.9, 4.7 Hz, 1H), 7.06 (ddt, *J* = 9.6, 8.2, 1.1 Hz, 2H), 7.00 (tt, *J* = 7.6, 1.3 Hz, 1H), 6.92 (d, *J* = 3.8 Hz, 1H), 6.90 – 6.87 (m, 2H), 6.85 (d, *J* = 11.7 Hz, 2H), 6.35 (d, *J* = 3.8 Hz, 1H), 5.65 (dt, *J* = 8.2, 0.8 Hz, 1H), 2.16 (s, 3H).

$^{13}$C NMR (151 MHz, $CDCl_3$) δ 161.64, 147.39, 144.87, 144.38, 141.58, 139.19, 136.75, 134.90, 133.34, 129.57, 129.53, 129.42, 129.28, 129.21, 129.14, 128.81, 128.43, 128.38, 127.19, 126.43, 124.36, 124.27, 122.12, 121.76, 120.79, 118.45, 117.57, 115.11, 113.02, 110.50, 110.22, 101.06, 20.97.

**HRMS** (ESI-TOF) (m/z): Calcd for $C_{37}H_{26}ClN_4O_3S^+$, ([M + H]$^+$), 641.1409, found 641.1403. **$[\alpha]_D^{20}$** = -54 (c = 0.1, $CHCl_3$).

**HPLC** conditions: Daicel Chiralpak OD-3 column (80: 20 hexane: 2-propanol, 0.8 mL/min, 40 °C, 254 nm); tr (major) = 11.1 min, tr (minor) = 13.9 min, 90% e.e.. > 20:1 dr determined by crude NMR.

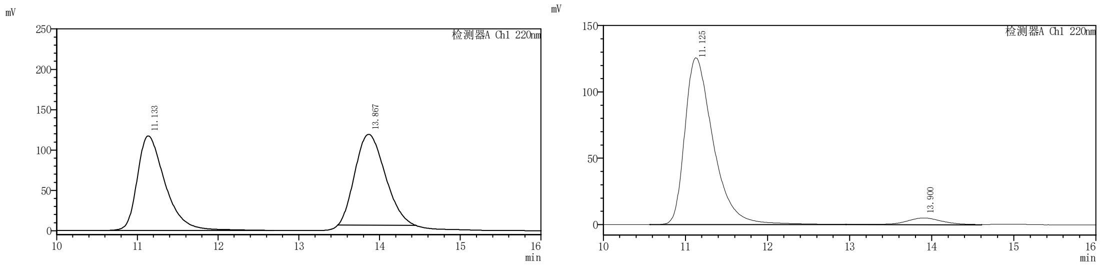


| No. | Time | Area | Area (%) | No. | Time | Area | Area (%) |
|---|---|---|---|---|---|---|---|
| 1 | 11.133 | 2808566 | 48.897 | 1 | 11.125 | 3044331 | 94.903 |
| 2 | 13.867 | 2935236 | 51.103 | 2 | 13.900 | 163506 | 5.097 |

**Supplementary Figure 16. HPLC data of 18.**

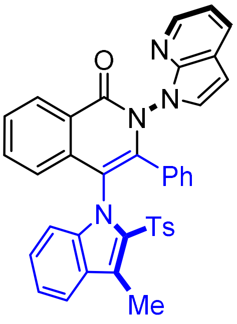


**(*S,S*)-4-(3-methyl-2-tosyl-1H-indol-1-yl)-3-phenyl-2-(1H-pyrrolo[2,3-b]pyridin-1-yl)isoquinolin-1(2H)-one (19)**

White solid (31.1 mg, 50%) $^1$H NMR (600 MHz, $CDCl_3$) δ 8.50 (dd, *J* = 4.7, 1.6 Hz, 1H), 8.38 (d, *J* = 8.0 Hz, 1H), 7.84 (dd, *J* = 7.9, 1.5 Hz, 1H), 7.67 (d, *J* = 8.1 Hz, 1H), 7.62 (d, *J* = 8.0 Hz, 2H), 7.59 – 7.47 (m, 1H), 7.36 (d, *J* = 7.7 Hz, 2H), 7.22 (t, *J* = 7.5 Hz, 1H), 7.18 – 7.12 (m, 2H), 7.10 (t, *J* = 7.7 Hz, 1H), 7.02 (d, *J* = 11.3 Hz, 1H), 6.98 (t, *J* = 7.5 Hz, 1H), 6.89 - 6.92 (m, 3H), 6.81 (t, *J* = 7.7 Hz, 2H), 6.34 (d, *J* = 3.8 Hz, 1H), 5.85 (d, *J* = 8.2 Hz, 1H), 2.67 (s, 3H), 2.20 (s, 3H).

$^{13}$C NMR (151 MHz, $cdcl_3$) δ 160.84, 147.46, 146.78, 144.83, 143.78, 140.12, 138.04, 135.54, 133.14, 132.39, 129.36, 129.34, 129.29, 129.24, 128.49, 128.09, 127.75, 127.12, 127.02, 126.98, 126.74, 124.50, 122.66, 122.23, 121.29, 121.00, 118.39, 117.41, 113.71, 111.53, 100.95, 21.39, 10.25.

**HRMS** (ESI-TOF) (m/z): Calcd for $C_{38}H_{29}N_4O_3S^+$, ([M + H]$^+$), 621.1955, found 621.1944. **$[\alpha]_D^{20}$** = -23 (c = 0.1, $CHCl_3$).

**HPLC** conditions: Daicel Chiralpak OD-3 column (80: 20 hexane: 2-propanol, 0.8 mL/min, 40 ºC, 254 nm); tr (major) = 8.5 min, tr (minor) = 12.8 min, 93% e.e.. > 20:1 dr determined by crude NMR.

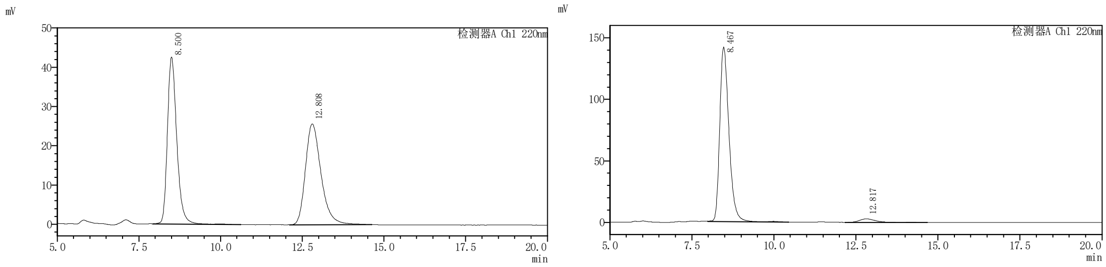


| No. | Time | Area | Area (%) | No. | Time | Area | Area (%) |
|---|---|---|---|---|---|---|---|
| 1 | 8.500 | 850831 | 49.882 | 1 | 8.467 | 2774101 | 96.592 |
| 2 | 12.808 | 854848 | 50.118 | 2 | 12.817 | 97870 | 3.408 |

**Supplementary Figure 17. HPLC data of 19.**

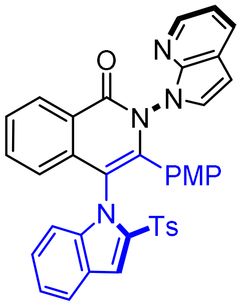


**(*S,S*)-3-(4-methoxyphenyl)-2-(1H-pyrrolo[2,3-b]pyridin-1-yl)-4-(2-tosyl-1H-indol-1-yl)isoquinolin-1(2H)-one (20)**

White solid (41.4 mg, 65%) $^1$H NMR (600 MHz, $CDCl_3$) δ 8.52 (dd, *J* = 4.8, 1.5 Hz, 1H), 8.32 (dd, *J* = 8.1, 1.4 Hz, 1H), 8.13 (d, *J* = 8.1 Hz, 1H), 7.87 (dd, *J* = 7.8, 1.5 Hz, 1H), 7.76 – 7.64 (m, 4H), 7.51 (d, *J* = 0.9 Hz, 1H), 7.33 (tdd, *J* = 8.2, 7.1, 1.1 Hz, 2H), 7.23 (ddd, *J* = 8.1, 7.0, 1.0 Hz, 1H), 7.20 – 7.14 (m, 2H), 7.02 (ddd, *J* = 8.4, 7.2, 1.4 Hz, 1H), 6.91 (d, *J* = 3.8 Hz, 1H), 6.89 – 6.82 (m, 2H), 6.37 (d, *J* = 3.8 Hz, 1H), 6.32 (dt, *J* = 8.8, 1.2 Hz, 2H), 5.69 – 5.60 (m, 1H), 3.53 (s, 3H), 2.14 (s, 3H).

$^{13}$C NMR (151 MHz, $CDCl_3$) δ 160.95, 160.02, 147.45, 147.18, 144.72, 144.06, 141.33, 138.22, 136.31, 135.21, 133.20, 130.51, 129.55, 129.42, 128.92, 128.34, 127.86, 126.97, 126.35, 125.39, 125.10, 124.35, 123.04, 122.34, 121.95, 121.23, 118.59, 117.48, 113.04, 112.19, 111.58, 101.10, 54.85, 21.35.

**HRMS** (ESI-TOF) (m/z): Calcd for $C_{38}H_{29}N_4O_4S^+$, ([M + H]$^+$), 637.1904, found 637.1907. **$[\alpha]_D^{20}$** = -47 (c = 0.1, $CHCl_3$).

**HPLC** conditions: Daicel Chiralpak OD-3 column (80: 20 hexane: 2-propanol, 0.8 mL/min, 40 °C, 254 nm); tr (major) = 22.0 min, tr (minor) = 33.6 min, 91% e.e.. > 20:1 dr determined by crude NMR.

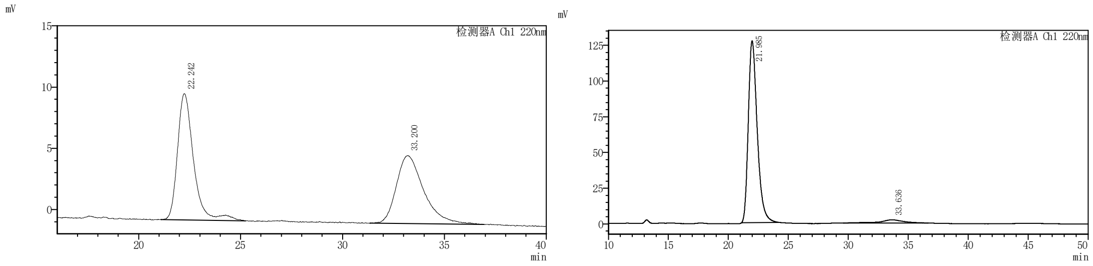


| No. | Time | Area | Area (%) | No. | Time | Area | Area (%) |
|---|---|---|---|---|---|---|---|
| 1 | 22.242 | 567170 | 52.186 | 1 | 21.985 | 6590396 | 95.460 |
| 2 | 33.200 | 519661 | 47.814 | 2 | 33.636 | 313431 | 4.540 |

**Supplementary Figure 18. HPLC data of 20.**

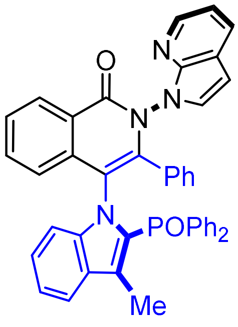


**(*S,S*)-4-(2-(diphenylphosphoryl)-3-methyl-1H-indol-1-yl)-3-phenyl-2-(1H-pyrrolo[2,3-b]pyridin-1-yl)isoquinolin-1(2H)-one (21)**

White solid (54.6 mg, 82%) $^1$H NMR (600 MHz, $CDCl_3$) δ 8.39 (dd, *J* = 8.0, 1.7 Hz, 1H), 8.28 (dt, *J* = 4.8, 1.7 Hz, 1H), 7.84 (s, 1H), 7.73 (dt, *J* = 7.8, 1.7 Hz, 1H), 7.57 – 7.49 (m, 2H), 7.47 – 7.39 (m, 6H), 7.40 – 7.33 (m, 3H), 7.32 – 7.25 (m, 5H), 7.18 – 7.05 (m, 3H), 7.02 (ddd, *J* = 7.8, 4.8, 1.6 Hz, 1H), 6.86 (q, *J* = 4.1, 3.2 Hz, 2H), 6.61 – 6.51 (m, 1H), 6.34 (dd, *J* = 3.8, 1.6 Hz, 1H), 6.25 (d, *J* = 8.1 Hz, 1H), 1.90 (s, 3H).

$^{13}$C NMR (151 MHz, $CDCl_3$) δ 161.20, 146.97, 144.84, 143.16, 140.86, 135.94, 134.45, 132.88, 132.09, 131.82, 131.75, 131.67, 129.97, 129.37, 129.19, 128.69, 128.51 (d, *J* = 15.6 Hz), 128.18 (d, *J* = 12.3 Hz), 127.85, 127.27, 125.37, 124.75, 123.07, 122.48, 120.57, 119.56, 118.56, 116.45, 115.12, 110.57, 100.31, 11.11.

**HRMS** (ESI-TOF) (m/z): Calcd for $C_{43}H_{32}N_4O_2P^+$, ([M + H]$^+$), 667.2257, found 667.2258. **$[\alpha]_D^{20}$** = -33 (c = 0.1, $CHCl_3$).

**HPLC** conditions: Daicel Chiralpak IC column (70: 30 hexane: 2-propanol, 0.8 mL/min, 40 °C, 254 nm); tr (major) = 14.6 min, tr (minor) = 22.6 min, 95% e.e.. > 20:1 dr determined by crude NMR.

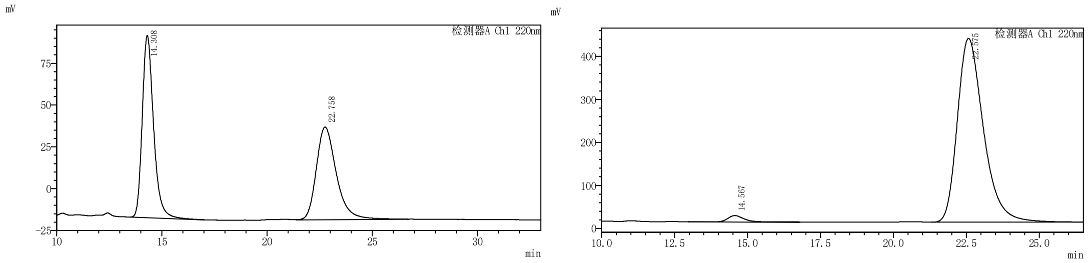


| No. | Time | Area | Area (%) | No. | Time | Area | Area (%) |
|---|---|---|---|---|---|---|---|
| 1 | 14.308 | 4083878 | 52.587 | 1 | 14.567 | 657214 | 2.411 |
| 2 | 22.758 | 3682004 | 47.413 | 2 | 22.575 | 26603537 | 97.589 |

**Supplementary Figure 19. HPLC data of 21.**

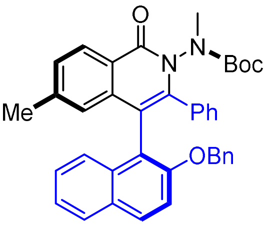


**(*S,S*)-tert-butyl (4-(2-(benzyloxy)naphthalen-1-yl)-6-methyl-1-oxo-3-phenylisoquinolin-2(1H)-yl)(methyl)carbamate (24)**

The title compound was isolated as a pale-yellow solid (54.4 mg, 91%, two amid bond rotamers 63% : 37%). $^{1}$H NMR (600 MHz, $CDCl_3$) δ 8.52 - 8.44 (m, 1H), 7.72 – 7.65 (m, 2H), 7.55 – 7.49 (m, 1H), 7.39 – 7.26 (m, 4H), 7.26 – 7.13 (m, 5H), 7.10 – 6.98 (m, 4H), 6.96 - 6.92 (m, 1H), 6.86 – 6.82 (m, 1H), 6.65 - 6.55 (m, 1H), 5.08 – 4.96 (m, 2H), 3.09 (s, 1.9H, major), 3.02 (s, 1.1H, minor), 2.20 (s, 1.9H, major), 2.17 (s, 1.1H, minor), 1.43 (s, 3.3H, minor), 1.22 (s, 5.7H, major); $^{13}$C NMR (150 MHz, $CDCl_3$) δ 160.8, 160.6, 154.9, 154.5, 154.4, 154.1, 143.6, 143.5, 143.42, 143.36, 137.8, 137.7, 137.4, 137.2, 134.8, 134.3, 133.8, 129.8, 129.7, 129.1, 129.0, 128.9, 128.8, 128.6, 128.5, 128.44, 128.40, 128.36, 128.34, 128.32, 128.2, 128.1, 128.0, 127.8, 127.7, 127.6, 127.4, 127.2, 127.1, 126.9, 126.72, 126.69, 126.6, 126.5, 125.3, 125.2, 125.14, 125.11, 123.8, 123.7, 123.59, 123.56, 119.4, 119.3, 114.8, 114.5, 112.3, 112.2, 81.5, 81.3, 70.9, 70.3, 37.8, 36.9, 28.3, 28.0, 22.1; **HRMS (ESI)**: calcd. for $C_{39}H_{36}N_2NaO_4^+$ $[M+Na]^+$ : 619.2567, found : 619.2567; $[\alpha]_D^{20}$ = -58 (c = 0.1, $CHCl_3$).

**HPLC analysis**: Daicel Chiralpak IE column (hexane: 2-propanol = 90:10, v = 1.0 mL/min, 40 °C, 254 nm); tr (minor) = 20.357 min, tr (major) = 33.021 min, 97% ee. > 20:1 dr determined by crude NMR.

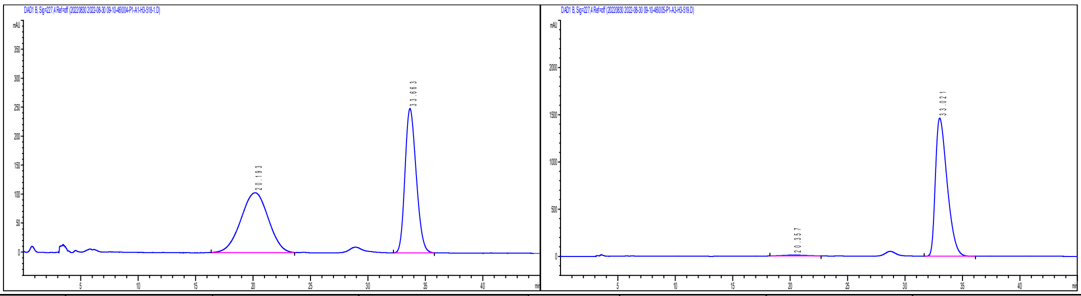

| No. | Time | Area | Area (%) | No. | Time | Area | Area (%) |
|---|---|---|---|---|---|---|---|
| 1 | 20.193 | 17134.7 | 50.41 | 1 | 20.357 | 1422.5 | 1.36 |
| 2 | 33.663 | 16853.1 | 49.59 | 2 | 33.021 | 103469.3 | 98.64 |

**Supplementary Figure 20. HPLC data of 24.**

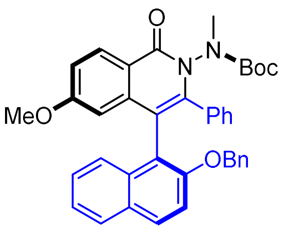

**(*S,S*)-tert-butyl (4-(2-(benzyloxy)naphthalen-1-yl)-6-methoxy-1-oxo-3-phenylisoquinolin-2(1H)-yl)(methyl)carbamate (25)**

The title compound was isolated as a pale-yellow solid (47.2 mg, 77%, two amide bond rotamers 63% : 37%) $^{1}$H NMR (600 MHz, $CDCl_3$) δ 8.51 (d, *J* = 8.8 Hz, 1H), 7.72 – 7.65 (m, 2H), 7.57 - 7.50 (m, 1H), 7.39 – 7.30 (m, 2H), 7.29 – 7.26 (m, 1H), 7.25 - 7.15 (m, 4H), 7.09 – 7.01 (m, 5H), 6.94 (d, *J* = 7.7 Hz, 1H), 6.85 (t, *J* = 7.6 Hz, 1H), 6.22 – 6.13 (m, 1H), 5.10 – 4.99 (m, 2H), 3.49 (s, 1.9H, major), 3.47 (s, 1.1H, minor), 3.08 (s, 1.9H, major), 3.02 (s, 1.1H, minor), 1.44 (s, 3.3H, minor), 1.24 (s, 5.7H, major); $^{13}$C NMR (150 MHz, $CDCl_3$) δ 163.3, 163.2, 155.0, 154.5, 154.4, 154.1, 144.1, 144.0, 139.81, 139.78, 137.4, 137.2, 134.7, 134.2, 133.8, 130.5, 130.0, 129.8, 129.1, 129.0, 128.8, 128.6, 128.44, 128.37, 128.2, 128.14, 128.06, 127.8, 127.6, 127.5, 127.4, 127.2, 127.14, 127.11, 127.0, 126.8, 126.74, 126.65, 126.58, 125.2, 125.0, 123.7, 123.6, 119.6, 119.3, 115.2, 115.1, 114.9, 114.5, 112.0, 107.7, 107.6, 81.5, 81.3, 71.0, 70.4, 55.3, 55.2, 37.8, 36.9, 28.3, 28.1; **HRMS (ESI)**: calcd. for $C_{39}H_{37}N_2O_5^+$ $[M+H]^+$ : 613.2697, found : 613.2692; **[α]$_D^{20}$** = -46 (c = 0.1, $CHCl_3$).

**HPLC analysis**: Daicel Chiralpak IE column (hexane: 2-propanol = 85:15, v = 1.0 mL/min, 40 ºC, 254 nm); tr (minor) = 16.323 min, tr (major) = 29.150 min, 92% ee. 16:1 dr determined by crude NMR.

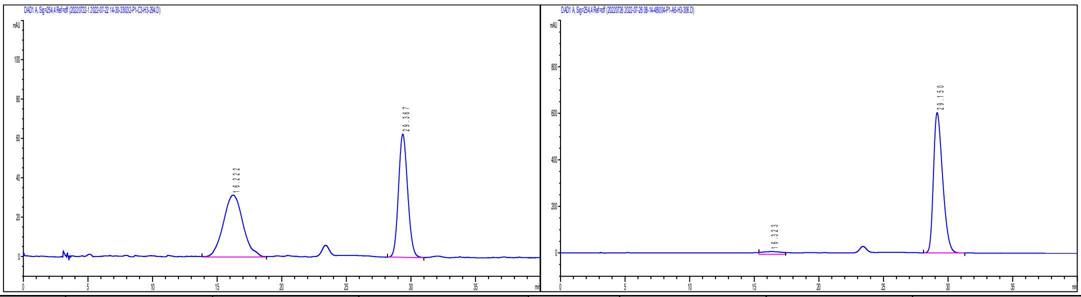

| No. | Time | Area | Area (%) | No. | Time | Area | Area (%) |
|---|---|---|---|---|---|---|---|
| 1 | 16.222 | 3344.9 | 52.06 | 1 | 16.323 | 1184.4 | 3.84 |
| 2 | 29.367 | 3080.4 | 47.94 | 2 | 29.150 | 29688.0 | 96.16 |

**Supplementary Figure 21. HPLC data of 25.**

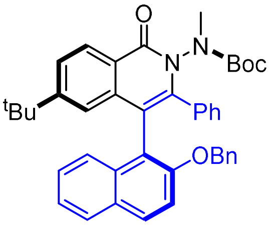

**(*S,S*)-tert-butyl (4-(2-(benzyloxy)naphthalen-1-yl)-6-(tert-butyl)-1-oxo-3-phenylisoquinolin-2(1H)-yl)(methyl)carbamate (26)**

The title compound was isolated as a pale-yellow solid (56.0 mg, 88%, two amide bond rotamers 63% : 37%) $^{1}$H NMR (600 MHz, $CDCl_3$) δ 8.56 - 8.50 (m, 1H), 7.74 – 7.65 (m, 2H), 7.60 – 7.54 (m, 2H), 7.36 – 7.26 (m, 3H), 7.25 – 7.11 (m, 4H), 7.09 – 6.98 (m, 3H), 6.98 - 6.94 (m, 1H), 6.90 – 6.85 (m, 2H), 6.83 – 6.78 (m, 1H), 5.11 – 4.97 (m, 2H), 3.11 (s, 1.9H, major), 3.02 (s, 1.1H, minor), 1.44 (s, 3.3H, minor), 1.20 (s, 5.7H, major), 1.08 (s, 5.7H, major), 1.04 (s, 3.3H, minor); $^{13}$C NMR (150 MHz, $CDCl_3$) δ 160.7, 160.6, 156.4, 156.2, 155.0, 154.54, 154.50, 154.1, 143.4, 143.3, 137.6, 137.54, 137.46, 137.2, 134.8, 134.2, 133.9, 133.8, 129.9, 129.8, 129.3, 129.1, 128.9, 128.8, 128.6, 128.4, 128.3, 128.2, 128.15, 128.08, 128.0, 127.84, 127.76, 127.7, 127.4, 127.12, 127.10, 126.94, 126.92, 126.7, 126.58, 126.56, 126.41, 125.36, 125.2, 124.8, 124.7, 123.8, 123.7, 123.6, 123.5, 121.8, 121.6, 119.4, 114.9, 114.4, 112.8, 112.7, 81.5, 81.4, 71.1, 70.4, 37.8, 36.9, 35.2, 35.1, 31.0, 30.9, 28.3, 28.0; **HRMS (ESI)**: calcd. for $C_{42}H_{43}N_2O_4^+$ $[M+H]^+$ : 639.3217, found :639.3209 ; **[α]$_D^{20}$** = -24 (c = 0.1, $CHCl_3$).

**HPLC analysis**: Daicel Chiralpak IC column (hexane: 2-propanol = 95:5, v = 1.0 mL/min, 40 ºC, 254

nm); tr (major) = 21.867 min, tr (minor) = 29.240 min, 93% ee. > 20:1 dr determined by crude NMR.

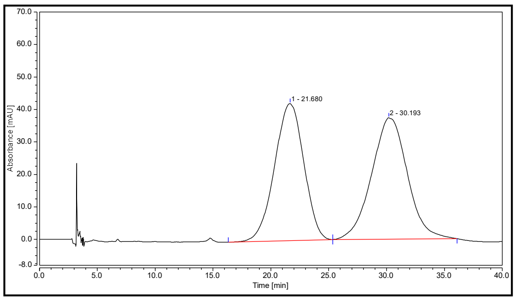

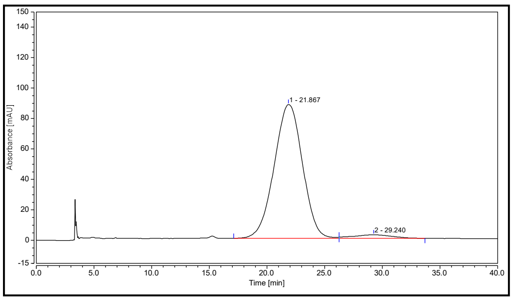

| No. | Time | Area | Area (%) | No. | Time | Area | Area (%) |
|---|---|---|---|---|---|---|---|
| 1 | 21.680 | 123.8 | 48.11 | 1 | 21.867 | 250.7 | 96.54 |
| 2 | 30.193 | 133.6 | 51.89 | 2 | 29.240 | 9.0 | 3.46 |

**Supplementary Figure 24. HPLC data of 26.**

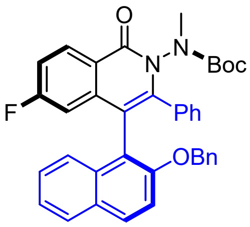

**(*S,S*)-tert-butyl (4-(2-(benzyloxy)naphthalen-1-yl)-6-fluoro-1-oxo-3-phenylisoquinolin-2(1H)-yl)(methyl)carbamate (27)**

The title compound was isolated as a pale-yellow solid (54.0 mg, 90%, two amide bond rotamers 63% : 37%) $^1$H NMR (600 MHz, $CDCl_3$**)** δ 8.61 - 8.57 (m, 1H), 7.75 – 7.67 (m, 2H), 7.52 - 7.44 (m, 1H), 7.40 – 7.26 (m, 4H), 7.23 – 7.16 (m, 4H), 7.11 – 7.02 (m, 4H), 6.98 – 6.92 (m, 1H), 6.91 - 6.85 (m, 1H), 6.46 - 6.36 (m, 1H), 5.11 – 5.01 (m, 2H), 3.08 (s, 1.9H, major), 3.03 (s, 1.1H, minor), 1.44 (s, 3.3H, minor), 1.23 (s, 5.7H, major); $^{13}$C NMR (150 MHz, $CDCl_3$) δ 166.8, 165.1, 160.2, 160.0, 154.8, 154.44, 154.40, 154.2, 145.0, 144.9, 140.4, 140.3, 137.3, 137.0, 134.5, 134.0, 133.4, 131.8, 131.7, 131.6, 131.5, 130.3, 130.2, 130.0, 128.89, 128.85, 128.64, 128.62, 128.5, 128.4, 128.3, 128.2, 128.0, 127.6, 127.5, 127.4, 127.3, 127.2, 127.1, 127.01, 126.97, 126.9, 126.6, 124.9, 124.7, 123.9, 123.7, 122.7, 122.4, 118.6, 118.5, 115.5, 115.4, 115.3, 115.2, 114.8, 114.5, 111.9, 110.9, 110.8, 110.7, 110.6, 81.7, 81.6, 71.1, 70.5, 37.8, 36.9, 28.3, 28.0; **$^{19}$F NMR (376 MHz, $CDCl_3$)** δ -105.2 (major), -105.3 (minor); **HRMS (ESI)**: calcd. for $C_{38}H_{34}FN_2O_4^+$ $[M+H]^+$ : 601.2497, found : 601.2493; **$[\alpha]_D^{20}$** = -72 (c = 0.1, $CHCl_3$).

**HPLC analysis**: Daicel Chiralpak IE column (hexane: 2-propanol = 90:10, v = 1.0 mL/min, 40 °C, 254 nm); tr (minor) = 11.950 min, tr (major) = 18.848 min, 93% ee. 12:1 dr determined by crude NMR.

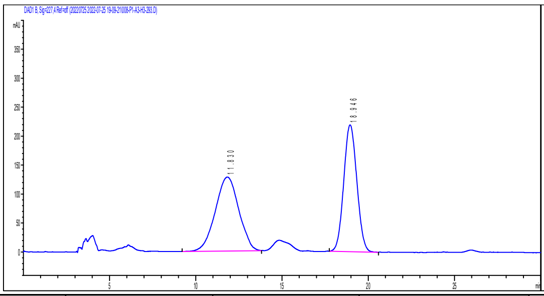

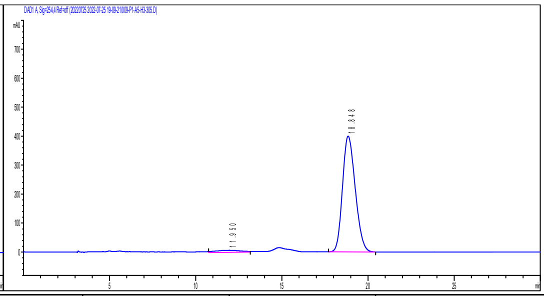

| No. | Time | Area | Area (%) | No. | Time | Area | Area (%) |
|---|---|---|---|---|---|---|---|
| 1 | 11.830 | 12051.6 | 51.04 | 1 | 11.950 | 763.8 | 3.59 |
| 2 | 18.946 | 11561.7 | 48.96 | 2 | 18.848 | 20516.6 | 96.41 |

**Supplementary Figure 23. HPLC data of 27.**

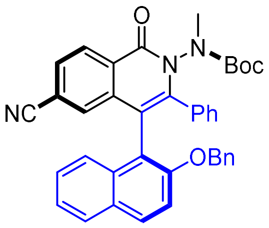


**(*S,S*)-tert-butyl (4-(2-(benzyloxy)naphthalen-1-yl)-6-cyano-1-oxo-3-phenylisoquinolin-2(1H)-yl)(methyl)carbamate (28)**

The title compound was isolated as a pale-yellow solid (49.9 mg, 82%, two amide bond rotamers 57% : 43%) $^{1}$H NMR (600 MHz, $CDCl_3$) δ 8.66 - 8.59 (m, 1H), 7.78 – 7.69 (m, 2H), 7.66 - 7.57 (m, 1H), 7.44 – 7.28 (m, 4H), 7.26 - 7.15 (m, 4H), 7.14 - 6.98 (m, 5H), 6.97 - 6.91 (m, 1H), 6.90 - 6.84 (m, 1H), 5.15 – 5.00 (m, 2H), 3.07 (s, 1.8H, major), 3.03 (s, 1.2H, minor), 1.42 (s, 3.7H, minor), 1.24 (s, 5.3H, major); $^{13}$C NMR (150 MHz, $CDCl_3$) δ 159.8, 159.6, 154.4, 154.3, 154.20, 154.16, 145.9, 145.7, 138.0, 137.0, 136.7, 134.3, 133.9, 132.92, 132.90, 130.7, 130.6, 130.4, 130.3, 129.6, 129.4, 129.00, 128.89, 128.9, 128.8, 128.68, 128.65, 128.6, 128.5, 128.43, 128.40, 128.3, 128.2, 128.1, 127.7, 127.4, 127.34, 127.32, 127.24, 127.21, 127.18, 1267.0, 126.73, 126.65, 124.4, 124.3, 124.0, 123.9, 118.3, 118.2, 117.5,117.4, 116.5, 116.4, 114.7, 114.4, 111.7, 111.6, 71.1, 70.5, 37.7, 36.7, 28.2, 28.0; **HRMS (ESI)**: calcd. for $C_{39}H_{34}N_3O_4^+$ [M+H]$^+$ : 608.2544, found :608.2541 ; **[α]$_D$$^{20}$** = -60 (c = 0.1, $CHCl_3$).

**HPLC analysis**: Daicel Chiralpak IE column (hexane: 2-propanol = 95:5, v = 1.0 mL/min, 40 °C, 254 nm); tr (minor) = 29.364 min, tr (major) = 33.906 min, 93% ee. 8:1 dr determined by crude NMR.

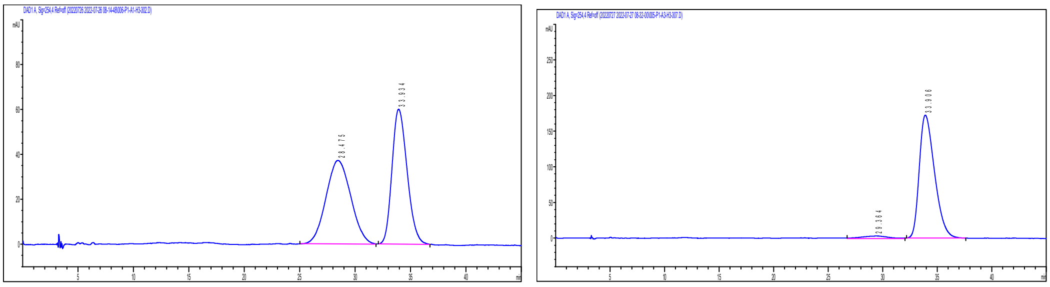


| No. | Time | Area | Area (%) | No. | Time | Area | Area (%) |
|---|---|---|---|---|---|---|---|
| 1 | 28.475 | 5786.9 | 50.35 | 1 | 29.364 | 631.0 | 3.62 |
| 2 | 33.934 | 5707.0 | 49.65 | 2 | 33.906 | 16794.9 | 96.38 |

**Supplementary Figure 24. HPLC data of 28.**

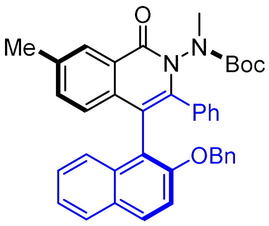


**(*S,S*)-tert-butyl (4-(2-(benzyloxy)naphthalen-1-yl)-7-methyl-1-oxo-3-phenylisoquinolin-2(1H)-yl)(methyl)carbamate (29)**

The title compound was isolated as a pale-yellow solid (48.1 mg, 81%, two amide bond rotamers 63% : 37%) $^{1}$H NMR (600 MHz, $CDCl_3$) δ 8.43 - 8.38 (m, 1H), 7.74 – 7.66 (m, 2H), 7.55 - 7.48 (m, 1H), 7.40 – 7.35 (m, 1H), 7.35 – 7.26 (m, 3H), 7.26 – 7.16 (m, 4H), 7.12 – 6.99 (m, 4H), 6.96 - 6.92 (m, 1H), 6.88 – 6.84 (m, 1H), 6.82 - 6.75 (m, 1H), 5.07 – 4.96 (m, 2H), 3.09 (s, 1.9H, major), 3.03 (s, 1.1H, minor), 2.50 (s, 1.9H, major), 2.45 (s, 1.1H, minor), 1.45 (s, 3.3H, minor), 1.24 (s, 5.7H, major); $^{13}$C NMR (150 MHz, $CDCl_3$) δ 160.8, 160.6, 155.0, 154.5, 154.4, 154.1, 142.5, 142.4, 137.4, 137.2, 136.9, 136.7, 135.4, 135.3, 134.8, 134.41, 134.37, 134.3, 133.8, 133.7, 129.9, 129.7, 129.2, 129.1, 128.9, 128.8, 128.6, 128.5, 128.4, 128.19, 128.15, 128.1, 127.9, 127.81, 127.78, 127.7, 127.4, 127.2, 127.0, 126.8, 126.7, 126.6, 126.0, 125.7, 125.6, 125.5, 125.4, 125.2, 125.1, 123.7, 123.6, 119.4, 119.3, 115.0, 114.6, 112.4, 112.3, 81.5, 81.4, 71.1, 70.5, 37.7, 36.8, 28.3, 28.0, 21.5; **HRMS (ESI)**: calcd. for $C_{39}H_{37}N_2O_4^+$ [M+H]$^+$ : 597.2748, found :597.2745 ; **[α]$_D$$^{20}$** = -32 (c = 0.1, $CHCl_3$).

**HPLC analysis**: Daicel Chiralpak IE column (hexane: 2-propanol = 85:15, v = 1.0 mL/min, 40 °C, 254 nm); tr (minor) = 12.270 min, tr (major) = 24.081 min, 98% ee. > 20:1 dr determined by crude NMR.

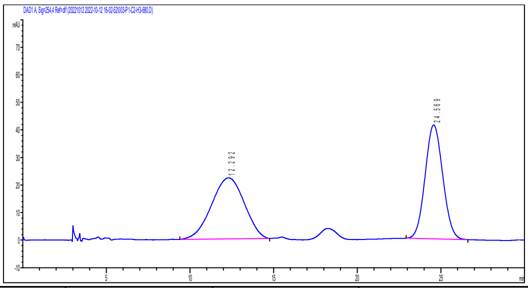

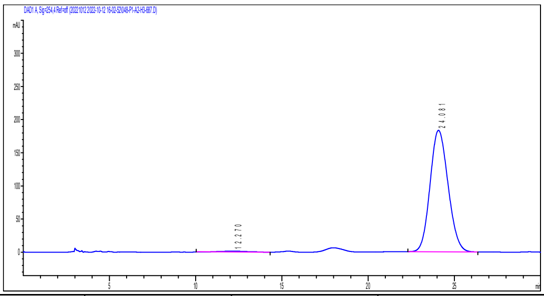

| No. | Time | Area | Area (%) | No. | Time | Area | Area (%) |
|---|---|---|---|---|---|---|---|
| 1 | 12.292 | 3028.2 | 49.93 | 1 | 12.270 | 159.0 | 1.18 |
| 2 | 24.569 | 3036.9 | 50.07 | 2 | 24.081 | 13304.0 | 98.82 |

**Supplementary Figure 25. HPLC data of 29.**

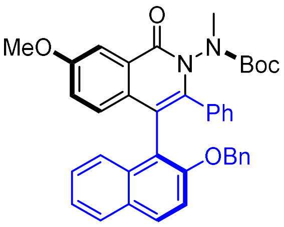


**(*S,S*)-tert-butyl (4-(2-(benzyloxy)naphthalen-1-yl)-7-methoxy-1-oxo-3-phenylisoquinolin-2(1H)-yl)(methyl)carbamate (30)**

The title compound was isolated as a pale-yellow solid (59.4 mg, 97%, two amide bond rotamers 63% : 37%) $^{1}$H NMR (600 MHz, $CDCl_3$) δ 8.05 - 7.99 (m, 1H), 7.75 – 7.66 (m, 2H), 7.55 – 7.47 (m, 1H), 7.40 – 7.37 (m, 1H), 7.36 – 7.27 (m, 3H), 7.24 – 7.16 (m, 4H), 7.09 – 7.03 (m, 4H), 6.97 – 6.94 (m, 1H), 6.89 - 6.84 (m, 1H), 6.81 - 6.76 (m, 1H), 5.05 - 4.96 (m, 2H), 3.96 (s, 1.9H, major), 3.92 (s, 1.1H, minor), 3.11 (s, 1.9H, major), 3.04 (s, 1.1H, minor), 1.46 (s, 3.3H, minor), 1.25 (s, 5.7H, major); $^{13}$C NMR (150 MHz, $CDCl_3$) δ 160.5, 160.4, 158.7, 158.6, 154.9, 154.7, 154.5, 154.4, 154.1, 141.1, 141.0, 137.4, 137.2, 134.8, 134.3, 133.7, 131.70, 131.68, 129.9, 129.8, 129.3, 129.2, 129.0, 128.9, 128.8, 128.57, 128.55, 128.5, 128.4, 128.3, 128.21, 128.16, 128.1, 127.9, 127.82, 127.80, 127.7, 127.6, 127.4, 127.31, 127.26, 127.2, 127.14, 127.12, 127.0, 126.9, 126.78, 126.75, 126.60, 126.57, 126.5, 125.2, 125.0, 123.7, 123.6, 123.3, 123.1, 120.9, 119.33, 119.26, 114.9, 114.5, 112.4, 112.3, 108.4, 108.2, 81.5, 81.4, 71.0, 70.4, 55.8, 55.7, 37.7, 36.7, 28.3, 28.0; **HRMS (ESI)**: calcd. for $C_{39}H_{37}N_2O_5^+$ [M+H]$^+$ : 613.2697, found : 613.2692; **[α]$_D^{20}$** = -48 (c = 0.1, $CHCl_3$).

**HPLC analysis**: Daicel Chiralpak IE column (hexane: 2-propanol = 85:15, v = 1.0 mL/min, 40 ºC, 254 nm); tr (minor) = 13.154 min, tr (major) = 24.886 min, 90% ee. > 20:1 dr determined by crude NMR.

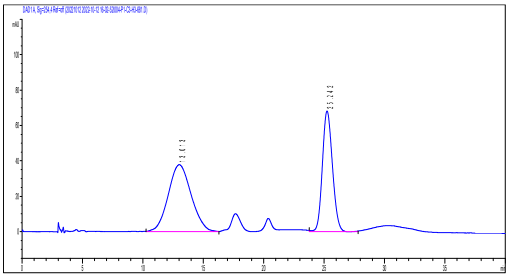

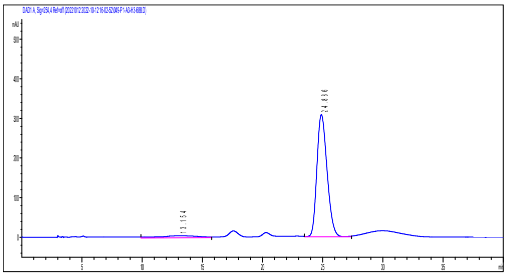

| No. | Time | Area | Area (%) | No. | Time | Area | Area (%) |
|---|---|---|---|---|---|---|---|
| 1 | 13.013 | 4965.3 | 50.10 | 1 | 13.154 | 1130.8 | 4.77 |
| 2 | 25.242 | 4945 | 49.90 | 2 | 24.886 | 22599.7 | 95.23 |

**Supplementary Figure 26. HPLC data of 30.**

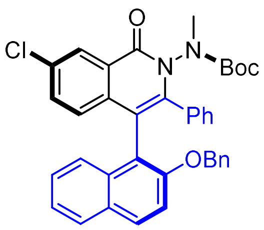

**(*S,S*)-tert-butyl (4-(2-(benzyloxy)naphthalen-1-yl)-7-chloro-1-oxo-3-phenylisoquinolin-2(1H)-yl)(methyl)carbamate (31)**

The title compound was isolated as a white solid (34.8 mg, 57%, two amide bond rotamers 63% : 37%) $^{1}$H NMR (600 MHz, $CDCl_3$) δ 8.58 - 8.51 (m, 1H), 7.75 – 7.68 (m, 2H), 7.50 - 7.44 (m, 1H), 7.36 – 7.27 (m, 4H), 7.24 – 7.14 (m, 4H), 7.13 – 7.00 (m, 4H), 6.95 - 6.92 (m, 1H), 6.89 – 6.84 (m, 1H), 6.77 - 6.71 (m, 1H), 5.08 – 4.98 (m, 2H), 3.07 (s, 1.9H, major), 3.02 (s, 1.1H, minor), 1.44 (s, 3.3H, minor), 1.24 (s, 5.7H, major); $^{13}$C NMR (150 MHz, $CDCl_3$) δ 159.9, 159.7, 154.7, 154.38, 154.35, 154.2, 143.9, 143.8, 137.3, 137.0, 136.09, 136.07, 134.6, 134.1, 133.34, 133.30, 132.8, 132.7, 130.2, 130.1, 129.1, 128.9, 128.8, 128.64, 128.60, 128.55, 128.5, 128.3, 128.2, 127.98, 127.96, 127.7, 127.6, 127.5, 127.32, 127.27, 127.25, 127.2, 127.1, 126.99, 126.98, 126.91, 126.87, 126.63, 126.57, 124.9, 124.8, 123.9, 123.7, 118.6, 118.5, 114.8, 114.5, 112.1, 112.0, 81.8, 81.6, 71.1, 70.4, 37.7, 36.7, 28.3, 28.0; **HRMS (ESI)**: calcd. for $C_{38}H_{34}ClN_2O_4^+$ [M+H]$^+$ : 617.2192, found : 617.2192; **[α]$_D$$^{20}$** = -52 (c = 0.1, $CHCl_3$).

**HPLC analysis**: Daicel Chiralpak IE column (hexane: 2-propanol = 85:15, v = 1.0 mL/min, 40 °C, 254 nm); tr (minor) = 10.547 min, tr (major) = 28.783 min, 97% ee. > 20:1 dr determined by crude NMR.

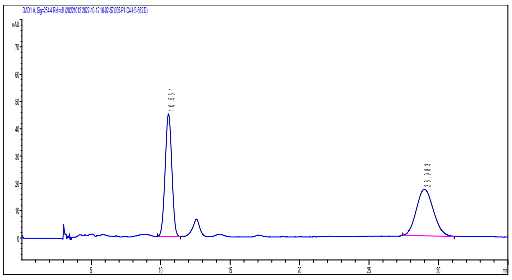

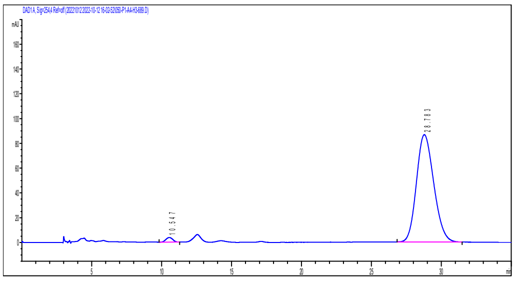

| No. | Time | Area | Area (%) | No. | Time | Area | Area (%) |
|---|---|---|---|---|---|---|---|
| 1 | 10.561 | 1502.0 | 50.87 | 1 | 10.547 | 125.3 | 1.65 |
| 2 | 28.983 | 1450.4 | 49.13 | 2 | 28.783 | 7450.2 | 98.35 |

**Supplementary Figure 27. HPLC data of 31.**

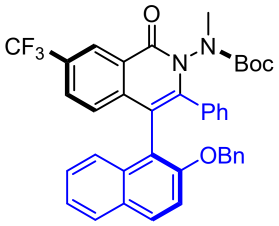

**(*S,S*)-tert-butyl (4-(2-(benzyloxy)naphthalen-1-yl)-1-oxo-3-phenyl-7-(trifluoromethyl)isoquinolin-2(1H)-yl)(methyl)carbamate (32)**

The title compound was isolated as a pale-yellow solid (35.7 mg, 55%, two amide bond rotamers 63% : 37%) $^{1}$H NMR (600 MHz, $CDCl_3$) δ 8.88 - 8.85 (m, 1H), 7.77 – 7.70 (m, 2H), 7.58 – 7.50 (m, 2H), 7.48 – 7.42 (m, 1H), 7.37 – 7.34 (m, 1H), 7.34 – 7.26 (m, 2H), 7.22 - 7.17 (m, 2H), 7.17 – 7.05 (m, 4H), 7.01 - 6.98 (m, 1H), 6.97 – 6.94 (m, 1H), 6.92 – 6.84 (m, 2H), 5.09 – 4.98 (m, 2H), 3.09 (s, 1.9H, major), 3.03 (s, 1.1H, minor), 1.45 (s, 3.3H, minor), 1.25 (s, 5.7H, major); $^{13}$C NMR (150 MHz, $CDCl_3$) δ 160.3, 160.0, 154.6, 154.42, 154.36, 154.2, 146.0, 145.9, 140.2, 140.1, 137.1, 136.9, 134.5, 134.0, 133.2, 133.1, 130.4, 130.3, 129.01, 128.99, 128.95, 128.9, 128.84, 128.78, 128.7, 128.6, 128.5, 128.4, 128.3, 128.1, 127.6, 127.5, 127.4, 127.31, 127.28, 127.2, 127.11, 127.08, 126.9, 126.6, 126.5, 126.4, 126.34, 126.31, 126.1, 126.0, 125.9, 125.6, 125.0, 124.8, 124.6, 124.0, 123.8, 123.2, 118.3, 118.2, 114.8, 114.5, 112.1, 112.0, 82.0, 81.8, 71.1, 70.5, 37.7, 36.7, 28.3, 28.0; **$^{19}$F NMR (376 MHz, $CDCl_3$)** δ -62.19 (major), -62.32 (minor); **HRMS (ESI)**: calcd. for $C_{39}H_{34}F_3N_2O_4^+$ [M+H]$^+$ : 651.2465, found : 651.2465; **[α]$_D$$^{20}$** = -64 (c = 0.1, $CHCl_3$).

**HPLC analysis**: Daicel Chiralpak AD-H column (hexane: 2-propanol = 95:5, v = 1.0 mL/min, 40 °C,

254 nm); tr (minor) = 7.708 min, tr (major) = 13.607 min, 94% ee. 19:1 dr determined by crude NMR.

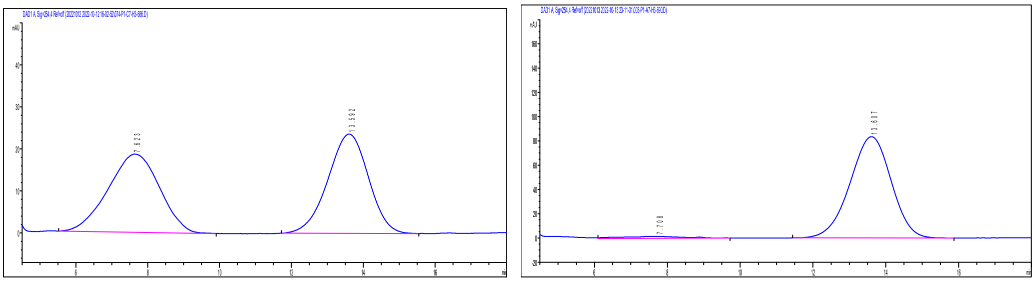

| No. | Time | Area | Area (%) | No. | Time | Area | Area (%) |
|---|---|---|---|---|---|---|---|
| 1 | 7.623 | 1852.3 | 49.87 | 1 | 7.708 | 204.4 | 3.02 |
| 2 | 13.592 | 1861.8 | 50.13 | 2 | 13.607 | 6569.3 | 96.98 |

**Supplementary Figure 28. HPLC data of 32.**

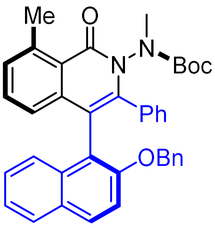


**(*S,S*)-tert-butyl (4-(2-(benzyloxy)naphthalen-1-yl)-8-methyl-1-oxo-3-phenylisoquinolin-2(1H)-yl)(methyl)carbamate (33)**

The title compound was isolated as a pale-yellow solid (20.9 mg, 36%, two amide bond rotamers 63% : 37%) $^{1}$H NMR (600 MHz, $CDCl_3$) δ 7.72 – 7.63 (m, 2H), 7.55 - 7.51 (m, 1H), 7.37 - 7.31 (m, 2H), 7.31 – 7.23 (m, 4H), 7.24 – 7.13 (m, 4H), 7.08 – 7.02 (m, 3H), 6.93 - 6.89 (m, 1H), 6.87 – 6.82 (m, 1H), 6.72 - 6.63 (m, 1H), 5.07 – 4.96 (m, 2H), 3.07 – 2.99 (m, 6H), 1.42 (s, 3.3H, minor), 1.23 (s, 5.7H, major); $^{13}$C NMR (150 MHz, $CDCl_3$) δ 161.3, 161.1, 155.2, 154.6, 154.4, 154.2, 143.4, 143.3, 142.6, 142.5, 139.3, 139.2, 137.6, 137.3, 134.8, 134.4, 133.91, 133.89, 132.12, 132.07, 130.0, 129.9, 129.8, 129.7, 129.1, 128.9, 128.8, 128.6, 128.5, 128.3, 128.2, 128.13, 128.08, 127.8, 127.6, 127.5, 127.4, 127.2, 127.1, 127.0, 126.74, 126.72, 126.68, 125.3, 125.2, 124.6, 124.2, 123.9, 123.8, 123.7, 123.6, 119.9, 119.8, 115.0, 114.6, 112.2, 112.0, 81.4, 81.2, 71.0, 70.5, 37.7, 36.8, 28.3, 28.1, 24.3, 24.2; **HRMS (ESI)**: calcd. for $C_{39}H_{37}N_2O_4^+$ $[M+H]^+$ : 597.2748, found :597.2733 ; **$[\alpha]_D^{20}$** = -28 (c = 0.1, $CHCl_3$).

**HPLC analysis**: Daicel Chiralpak IE column (hexane: 2-propanol = 95:5, v = 1.0 mL/min, 40 °C, 254 nm); tr (minor) = 15.044 min, tr (major) = 18.086 min, 94% ee. 13:1 dr determined by crude NMR.

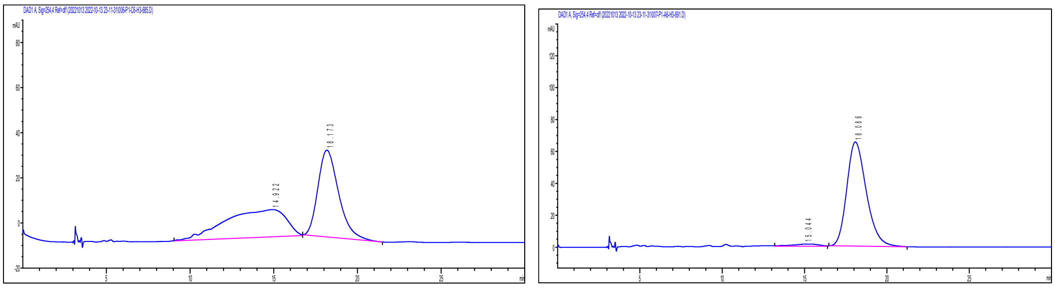

| No. | Time | Area | Area (%) | No. | Time | Area | Area (%) |
|---|---|---|---|---|---|---|---|
| 1 | 14.922 | 3091.8 | 48.22 | 1 | 15.044 | 160.2 | 2.88 |
| 2 | 18.173 | 3319.6 | 51.78 | 2 | 18.086 | 5399.0 | 97.12 |

**Supplementary Figure 29. HPLC data of 33.**

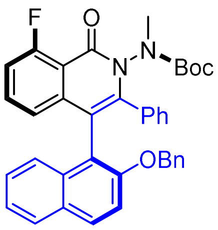

**(*S,S*)-tert-butyl (4-(2-(benzyloxy)naphthalen-1-yl)-8-fluoro-1-oxo-3-phenylisoquinolin-2(1H)-yl)(methyl)carbamate (34)**

The title compound was isolated as a pale-yellow solid (51.1 mg, 85%, two amide bond rotamers 63% : 37%) $^{1}$H NMR (600 MHz, $CDCl_3$) δ 7.73 – 7.65 (m, 2H), 7.52 – 7.46 (m, 1H), 7.38 – 7.27 (m, 4H), 7.26 – 7.16 (m, 4H), 7.15 – 7.10 (m, 1H), 7.09 – 7.00 (m, 4H), 6.96 - 6.92 (m, 1H), 6.88 - 6.83 (m, 1H), 6.64 - 6.55 (m, 1H), 5.08 – 5.01 (m, 2H), 3.09 (s, 1.9H, major), 3.03 (s, 1.1H, minor), 1.42 (s, 3.3H, minor), 1.23 (s, 5.7H, major); $^{13}$C NMR (150 MHz, $CDCl_3$) δ 163.9, 163.8, 162.10, 162.05, 157.83, 157.80, 157.62, 157.59, 154.8, 154.4, 154.1, 144.9, 144.8, 140.3, 140.2, 137.3, 137.0, 134.5, 134.0, 133.8, 133.7, 133.6, 133.44, 133.40, 130.1, 130.0, 128.9, 128.83, 128.79, 128.7, 128.6, 128.53, 128.49, 128.4, 128.2, 128.1, 127.9, 127.5, 127.4, 127.3, 127.20, 127.18, 127.0, 126.90, 126.88, 126.8, 126.5, 124.9, 124.8, 123.8, 123.6, 121.40, 121.37, 121.33, 121.30, 119.0, 118.9, 115.3, 115.2, 114.90, 114.87, 114.7, 114.4, 113.70, 113.67, 113.6, 113.5, 111.6, 111.5, 81.6, 81.5, 71.0, 70.3, 37.7, 36.8, 28.3, 28.0; **$^{19}$F NMR (376 MHz, $CDCl_3$)** δ -109.79 (major), -110.16 (minor); **HRMS (ESI)**: calcd. for $C_{38}H_{34}FN_2O_4^+$ $[M+H]^+$ : 601.2497, found : 601.2491; **$[\alpha]_D^{20}$** = -34 (c = 0.1, $CHCl_3$).

**HPLC analysis**: Daicel Chiralpak IE column (hexane: 2-propanol = 85:15, v = 1.0 mL/min, 40 ºC, 254 nm); tr (minor) = 17.494 min, tr (major) = 25.714 min, 93% ee. > 20:1 dr determined by crude NMR.

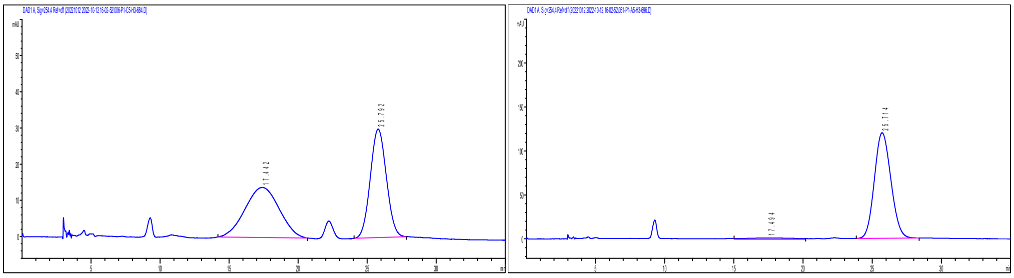

| No. | Time | Area | Area (%) | No. | Time | Area | Area (%) |
|---|---|---|---|---|---|---|---|
| 1 | 17.442 | 2382.7 | 49.54 | 1 | 17.494 | 339.0 | 3.30 |
| 2 | 25.792 | 2426.9 | 50.46 | 2 | 25.714 | 9947.5 | 96.70 |

**Supplementary Figure 30. HPLC data of 34.**

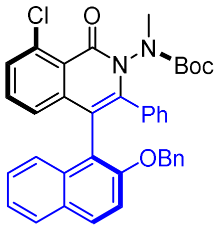

**(*S,S*)-tert-butyl (4-(2-(benzyloxy)naphthalen-1-yl)-8-chloro-1-oxo-3-phenylisoquinolin-2(1H)-yl)(methyl)carbamate (35)**

The title compound was isolated as a white solid (30.9 mg, 50%, two amide bond rotamers 63% : 37%) $^{1}$H NMR (600 MHz, $CDCl_3$) δ 7.72 – 7.65 (m, 2H), 7.50 – 7.42 (m, 2H), 7.37 – 7.32 (m, 2H), 7.32 – 7.26 (m, 2H), 7.26 – 7.16 (m, 4H), 7.10 – 7.01 (m, 4H), 6.94 - 6.89 (m, 1H), 6.87 – 6.83 (m, 1H), 6.76 - 6.68 (m, 1H), 5.08 – 4.99 (m, 2H), 3.07 (s, 1.9H, major), 3.03 (s, 1.1H, minor), 1.41 (s, 3.3H, minor), 1.24 (s, 5.7H, major); $^{13}$C NMR (150 MHz, $CDCl_3$) δ 158.9, 158.7, 154.8, 154.5, 154.4, 154.2, 144.8, 144.7, 140.6, 137.3, 137.1, 136.2, 136.0, 134.5, 134.1, 133.5, 133.4, 132.51, 132.46, 130.1, 130.02, 129.96, 129.9, 128.9, 128.8, 128.7, 128.6, 128.52, 128.51, 128.4, 128.23, 128.17, 128.0, 127.5, 127.32, 127.31, 127.22, 127.18, 127.0, 126.9, 126.8, 126.6, 124.9, 124.8, 124.7, 124.6, 123.8, 123.7, 122.7, 122.4, 119.1, 118.9, 114.7, 114.4, 111.6, 111.4, 81.6, 81.5, 71.0, 70.4, 37.7, 36.8, 28.3, 28.0; **HRMS (ESI)**: calcd. for $C_{38}H_{34}ClN_2O_4^+$ $[M+H]^+$ : 617.2202, found :617.2203 ; **$[\alpha]_D^{20}$** = -28 (c = 0.1, $CHCl_3$).

**HPLC analysis**: Daicel Chiralpak IE column (hexane: 2-propanol = 95:5, v = 1.0 mL/min, 40 °C, 254 nm); tr (minor) = 36.185 min, tr (major) = 58.762 min, 93% ee. > 20:1 dr determined by crude NMR.

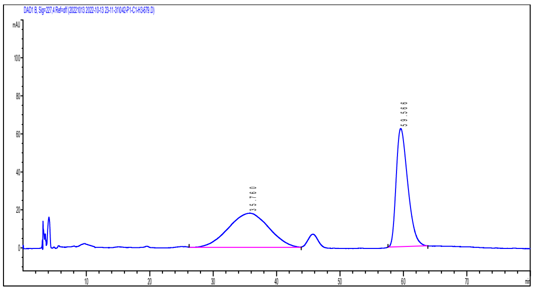
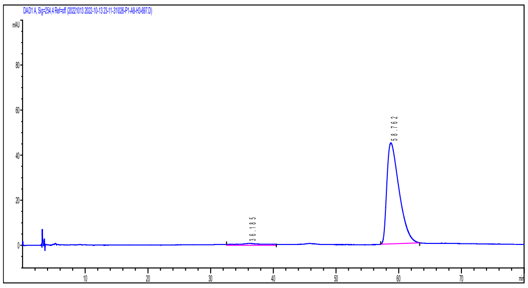

| No. | Time | Area | Area (%) | No. | Time | Area | Area (%) |
|---|---|---|---|---|---|---|---|
| 1 | 35.760 | 7950.0 | 50.09 | 1 | 36.185 | 227.0 | 3.59 |
| 2 | 59.566 | 7921.8 | 49.91 | 2 | 58.762 | 6097.3 | 96.41 |

**Supplementary Figure 31. HPLC data of 35.**

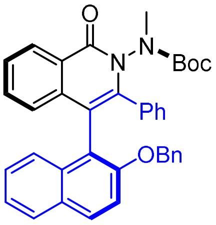


**(*S,S*)-tert-butyl (4-(2-(benzyloxy)naphthalen-1-yl)-1-oxo-3-phenylisoquinolin-2(1H)-yl)(methyl)carbamate (36)**

The title compound was isolated as a pale-yellow solid (56.4 mg, 97%, two amide bond rotamers 60% : 40%) $^1$H NMR (600 MHz, $CDCl_3$) δ 8.62 - 8.58 (m, 1H), 7.75 – 7.66 (m, 2H), 7.59 – 7.48 (m, 2H), 7.47 - 7.42 (m, 1H), 7.41 – 7.26 (m, 4H), 7.25 - 7.13 (m, 4H), 7.10 - 7.04 (m, 2H), 7.03 – 6.94 (m, 2H), 6.88 – 6.79 (m, 2H), 5.09 - 4.97 (m, 2H), 3.10 (s, 1.8H, major), 3.04 (s, 1.2H, minor), 1.45 (s, 3.6H, minor), 1.22 (s, 5.4H, major); $^{13}$C NMR (150 MHz, $CDCl_3$) δ 160.8, 160.7, 154.9, 154.5, 154.4, 154.2, 143.5, 143.4, 137.69, 137.65, 137.4, 137.1, 134.8, 134.3, 133.71, 133.68, 133.0, 132.9, 130.0, 129.8, 129.2, 129.04, 128.95, 128.8, 128.61, 128.58, 128.5, 128.4, 128.34, 128.28, 128.2, 128.1, 127.9, 127.7, 127.6, 127.4, 127.2, 127.0, 126.81, 126.79, 126.75, 126.7, 126.6, 126.1, 125.9, 125.6, 125.5, 125.2, 125.1, 123.8, 123.6, 119.2, 119.1, 114.9, 114.5, 112.5, 112.4, 81.6, 81.4, 71.1, 70.4, 37.8, 36.8, 28.3, 28.0; **HRMS (ESI)**: calcd. for $C_{38}H_{35}N_2O_4^+$ $[M+H]^+$ : 583.2591, found :583.2585 ; **[α]$_D$$^{20}$** = -36 (c = 0.1, $CHCl_3$).

**HPLC analysis**: Daicel Chiralpak IA column (hexane: 2-propanol = 95:5, v = 1.0 mL/min, 40 °C, 254 nm); tr (minor) = 10.855 min, tr (major) = 15.203 min, 96% ee. tr (minor) = 7.059 min, tr (major) = 26.139 min, 65% ee. > 20:1 dr determined by crude NMR.

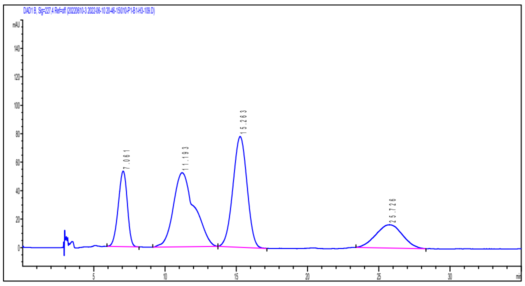
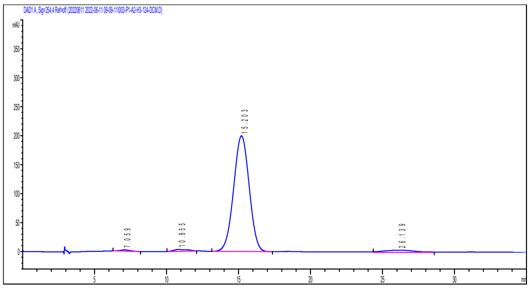

| No. | Time | Area | Area (%) | No. | Time | Area | Area (%) |
|---|---|---|---|---|---|---|---|
| 1 | 7.061 | 2347.0 | 15.45 | 1 | 7.059 | 117.1 | 0.74 |
| 2 | 11.193 | 5364.9 | 35.33 | 2 | 10.855 | 260.0 | 1.63 |
| 3 | 15.263 | 5359.2 | 35.29 | 3 | 15.203 | 15006.3 | 94.16 |
| 4 | 25.726 | 2116.2 | 13.93 | 4 | 26.139 | 553.6 | 3.47 |

**Supplementary Figure 32. HPLC data of 36.**

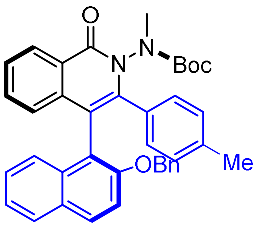


**(*S,S*)-tert-butyl (4-(2-(benzyloxy)naphthalen-1-yl)-1-oxo-3-(p-tolyl)isoquinolin-2(1H)-yl)(methyl)carbamate (37)**

The title compound was isolated as a pale-yellow solid (56.5 mg, 95%, two amide bond rotamers 63% : 37%) $^{1}$H NMR (600 MHz, $CDCl_3$) δ 8.64 - 8.54 (m, 1H), 7.75 – 7.67 (m, 2H), 7.56 – 7.40 (m, 3H), 7.38 – 7.25 (m, 4H), 7.22 – 7.08 (m, 4H), 7.04 - 6.99 (m, 1H), 6.91 – 6.73 (m, 3H), 6.69 - 6.56 (m, 1H), 5.08 – 4.95 (m, 2H), 3.10 (s, 1.9H, major), 3.03 (s, 1.1H, minor), 2.15 (s, 1.1H, minor), 2.13 (s, 1.9H, major), 1.47 (s, 3.3H, minor), 1.23 (s, 5.7H, major); $^{13}$C NMR (150 MHz, $CDCl_3$) δ 160.9, 160.7, 154.9, 154.5, 154.4, 154.1, 143.62, 143.56, 138.0, 137.9, 137.71, 137.67, 137.4, 137.2, 134.8, 134.3, 132.9, 132.8, 130.7, 129.8, 129.7, 129.01, 128.95, 128.9, 128.8, 128.6, 128.52, 128.49, 128.4, 128.3, 128.2, 128.1, 128.00, 127.95, 127.80, 127.75, 127.7, 127.6, 127.5, 127.44, 127.38, 127.2, 126.8, 126.7, 126.62, 126.56, 126.5, 126.0, 125.8, 125.5, 125.4, 125.3, 125.1, 123.73, 123.68, 123.6, 119.4, 119.3, 115.1, 114.6, 112.5, 112.3, 81.5, 81.4, 71.1, 70.4, 37.7, 36.8, 28.3, 28.0, 21.4, 21.3; **HRMS (ESI)**: calcd. for $C_{39}H_{36}N_2NaO_4^{+}$ [M+Na]$^{+}$ : 619.2567, found : 619.2563; **[α]$_D^{20}$** = -72 (c = 0.1, $CHCl_3$).

**HPLC analysis**: Daicel Chiralpak IG-H column (hexane: 2-propanol = 95:5, v = 1.0 mL/min, 40 °C, 254 nm); tr (minor) = 26.150 min, tr (major) = 37.760 min, 96% ee. tr (minor) = 16.927 min, tr (major) = 49.790 min, 65% ee. > 20:1 dr determined by crude NMR.

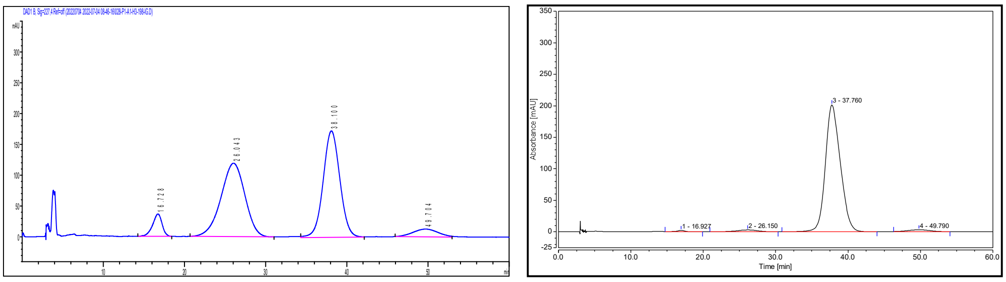


| No. | Time | Area | Area (%) | No. | Time | Area | Area (%) |
|---|---|---|---|---|---|---|---|
| 1 | 16.728 | 2957.8 | 5.40 | 1 | 16.927 | 2.21 | 0.44 |
| 2 | 26.043 | 24439.9 | 44.64 | 2 | 26.150 | 10.4 | 2.05 |
| 3 | 38.100 | 24650.6 | 45.03 | 3 | 37.760 | 482.9 | 95.45 |
| 4 | 49.704 | 2695.2 | 4.93 | 4 | 49.790 | 10.4 | 2.06 |

**Supplementary Figure 33. HPLC data of 37.**

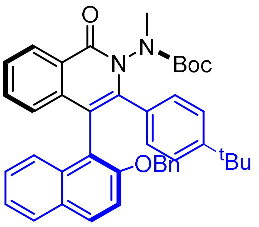


**(*S,S*)-tert-butyl (4-(2-(benzyloxy)naphthalen-1-yl)-3-(4-(tert-butyl)phenyl)-1-oxoisoquinolin-2(1H)-yl)(methyl)carbamate (38)**

The title compound was isolated as a pale-yellow solid (58.5 mg, 92%, two amide bond rotamers 63% : 37%) $^{1}$H NMR (600 MHz, $CDCl_3$) δ 8.60 - 8.55 (m, 1H), 7.73 – 7.63 (m, 2H), 7.56 – 7.44 (m, 2H), 7.43 - 7.37 (m, 1H), 7.37 – 7.24 (m, 4H), 7.24 – 7.11 (m, 4H), 7.09 – 7.04 (m, 1H), 7.02 – 6.98 (m, 1H), 6.87 - 6.81 (m, 2H), 6.81 – 6.75 (m, 1H), 5.12 – 4.92 (m, 2H), 3.08 (s, 1.9H, major), 3.03 (s, 1.1H, minor), 1.42 (s, 3.3H, minor), 1.23 (s, 5.7H, major), 1.14 (s, 3.3H, minor), 1.12 (s, 5.7H, major); $^{13}$C NMR (150 MHz, $CDCl_3$) δ 160.9, 160.7, 155.0, 154.4, 154.3, 154.2, 151.1, 151.0, 143.7, 143.6, 137.73, 137.68, 137.5, 137.2, 134.8, 134.4, 132.9, 132.8, 130.58, 130.55, 129.8, 129.7, 129.0, 128.91, 128.87, 128.8,

128.6, 128.52, 128.48, 128.4, 128.3, 128.2, 128.1, 128.0, 127.7, 127.41, 127.37, 127.3, 127.1, 126.68, 126.65, 126.6, 126.5, 126.0, 125.7, 125.5, 125.2, 125.1, 125.0, 124.2, 124.0, 123.7, 123.64, 123.60, 123.5, 123.3, 119.4, 119.3, 115.0, 114.6, 112.5, 112.4, 81.34, 81.32, 71.0, 70.5, 37.8, 36.7, 34.5, 31.21, 31.20, 28.3, 28.0; **HRMS (ESI)**: calcd. for $C_{42}H_{43}N_2O_4^+$ $[M+H]^+$ : 639.3217, found :639.3210 ; **[α]$_D$$^{20}$** = -64 (c = 0.1, **CHCl$_3$).**

**HPLC analysis**: Daicel Chiralpak IE column (hexane: 2-propanol = 80:20, v = 1.0 mL/min, 40 ºC, 254 nm); tr (minor) = 8.456 min, tr (major) = 13.093 min, 94% ee. 12:1 dr determined by crude NMR.

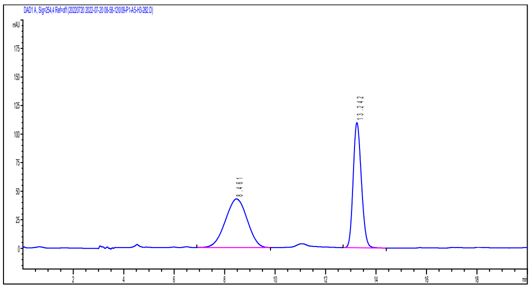

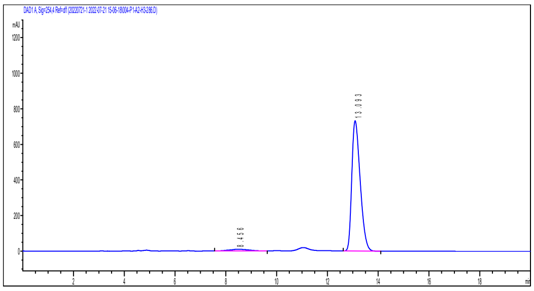

| No. | Time | Area | Area (%) | No. | Time | Area | Area (%) |
|---|---|---|---|---|---|---|---|
| 1 | 8.461 | 2558.3 | 50.76 | 1 | 8.456 | 521.7 | 3.00 |
| 2 | 13.242 | 2481.5 | 49.24 | 2 | 13.093 | 16842.6 | 97.00 |

**Supplementary Figure 34. HPLC data of 38.**

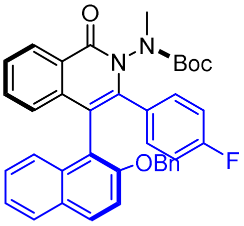


**(*S,S*)-tert-butyl (4-(2-(benzyloxy)naphthalen-1-yl)-3-(4-fluorophenyl)-1-oxoisoquinolin-2(1H)-yl)(methyl)carbamate (39)**

The title compound was isolated as a pale-yellow solid (46.2 mg, 78%, two amide bond rotamers 63% : 37%) $^1$H NMR (600 MHz, CDCl$_3$) δ 8.62 - 8.57 (m, 1H), 7.77 – 7.70 (m, 2H), 7.57 – 7.42 (m, 3H), 7.41 – 7.27 (m, 4H), 7.23 – 7.10 (m, 4H), 7.02 - 6.98 (m, 1H), 6.88 – 6.83 (m, 1H), 6.82 - 6.73 (m, 2H), 6.59 - 6.47 (m, 1H), 5.13 - 4.98 (m, 2H), 3.10 (s, 1.9H, major), 3.05 (s, 1.1H, minor), 1.47 (s, 3.3H, minor), 1.22 (s, 5.7H, major); $^{13}$C NMR (150 MHz, CDCl$_3$) δ 163.4, 163.3, 161.73, 161.68, 160.7, 160.6, 154.9, 154.5, 154.4, 154.1, 142.4, 142.3, 137.50, 137.46, 137.2, 137.0, 134.6, 134.2, 133.04, 132.98, 131.24, 131.18, 131.00, 130.95, 130.1, 130.0, 129.8, 129.7, 129.5, 129.4, 129.3, 129.0, 128.9, 128.62, 128.57, 128.5, 128.4, 128.3, 128.2, 127.9, 127.5, 127.2, 127.0, 126.9, 126.8, 126.64, 126.57, 126.1, 125.9, 125.6, 125.5, 125.0, 124.8, 123.9, 123.7, 119.0, 118.9, 114.9, 114.6, 114.54, 114.45, 114.4, 114.3, 114.2, 114.1, 114.0, 113.8, 112.9, 112.8, 81.8, 81.6, 71.1, 70.4, 37.7, 36.8, 28.3, 28.0; **$^{19}$F NMR (376 MHz, CDCl$_3$)** δ -112.56 (major), -112.88 (minor); **HRMS (ESI)**: calcd. for $C_{38}H_{34}FN_2O_4^+$ $[M+H]^+$ : 601.2497, found : 601.2493; **[α]$_D$$^{20}$** = -38 (c = 0.1, CHCl$_3$).

**HPLC analysis**: Daicel Chiralpak IE column (hexane: 2-propanol = 95:5, v = 1.0 mL/min, 40 ºC, 254 nm); tr (minor) = 22.486 min, tr (major) = 35.684 min, 96% ee. 12:1 dr determined by crude NMR.

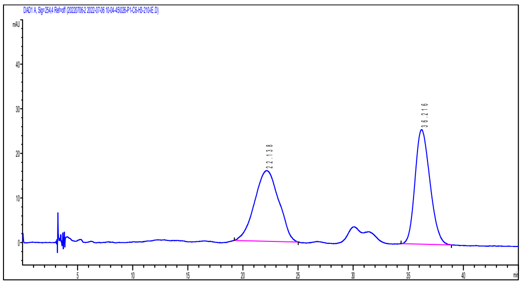

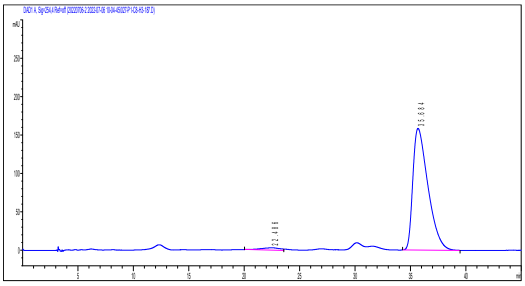

| No. | Time | Area | Area (%) | No. | Time | Area | Area (%) |
|---|---|---|---|---|---|---|---|
| 1 | 22.138 | 2410.1 | 49.80 | 1 | 22.486 | 333.0 | 2.17 |
| 2 | 36.216 | 2429.3 | 50.20 | 2 | 35.684 | 15014.9 | 97.83 |

**Supplementary Figure 35. HPLC data of 39.**

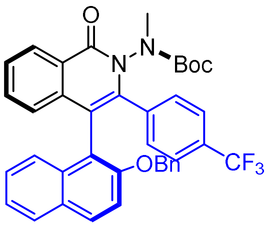


**(*S,S*)-tert-butyl (4-(2-(benzyloxy)naphthalen-1-yl)-1-oxo-3-(4-(trifluoromethyl)phenyl)isoquinolin-2(1H)-yl)(methyl)carbamate (40)**

The title compound was isolated as a white solid (39.3 mg, 61%, two amide bond rotamers 56% : 44%) $^1$H NMR (600 MHz, $CDCl_3$) δ 8.61 - 8.56 (m, 1H), 7.77 – 7.70 (m, 2H), 7.54 – 7.48 (m, 1H), 7.48 – 7.44 (m, 1H), 7.42 – 7.26 (m, 4H), 7.23 – 7.09 (m, 4H), 7.02 – 6.94 (m, 2H), 6.94 – 6.84 (m, 2H), 6.72 – 6.64 (m, 1H), 5.08 – 4.97 (m, 2H), 3.09 (s, 1.7H, major), 3.05 (s, 1.3H, minor), 1.43 (s, 4.0H, minor), 1.21 (s, 5.0H, major); $^{13}$C NMR (150 MHz, $CDCl_3$) δ 160.7, 160.5, 154.9, 154.5, 154.4, 154.1, 149.09, 142.07, 141.9, 137.44, 137.40, 137.2, 136.9, 134.6, 134.2, 133.1, 133.0, 132.3, 132.2, 131.1, 130.8, 130.3, 130.1, 129.2, 129.1, 129.0, 128.9, 128.64, 128.59, 128.5, 128.4, 128.34, 128.29, 128.0, 127.6, 127.2, 127.1, 127.02, 126.98, 126.6, 126.2, 125.9, 125.7, 125.6, 124.9, 124.7, 123.9, 123.8, 119.55, 119.45, 119.4, 119.1, 119.0, 118.8, 118.7, 118.6, 114.9, 114.5, 113.0, 112.9, 81.9, 81.7, 71.1, 70.4, 37.8, 36.8, 28.2, 28.0; **$^{19}$F NMR (376 MHz, $CDCl_3$)** δ -57.66 (major), -57.75 (minor); **HRMS (ESI)**: calcd. for $C_{39}H_{34}F_3N_2O_4^+$ $[M+H]^+$ : 651.2465, found : 651.2460; **$[\alpha]_D^{20}$** = -32 (c = 0.1, $CHCl_3$).

**HPLC analysis**: Daicel Chiralpak IE column (hexane: 2-propanol = 85:15, v = 1.0 mL/min, 40 °C, 254 nm); tr (minor) = 7.093 min, tr (major) = 9.788 min, 95% ee. 18:1 dr determined by crude NMR.

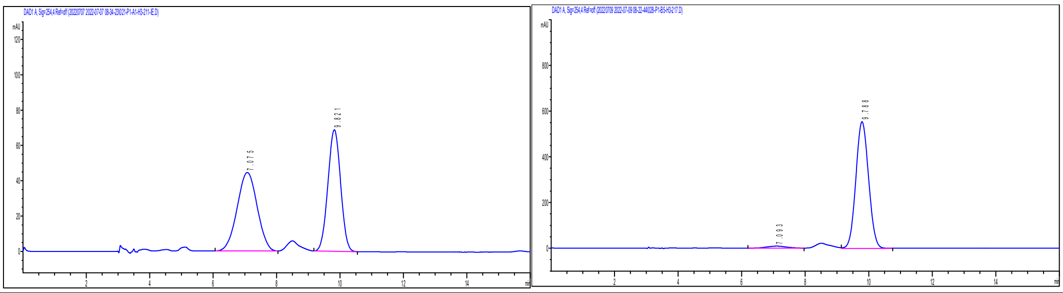

| No. | Time | Area | Area (%) | No. | Time | Area | Area (%) |
|---|---|---|---|---|---|---|---|
| 1 | 7.075 | 1949.3 | 50.40 | 1 | 7.093 | 383.6 | 2.42 |
| 2 | 9.821 | 1918.6 | 49.60 | 2 | 9.788 | 15445.4 | 97.58 |

**Supplementary Figure 36. HPLC data of 40.**

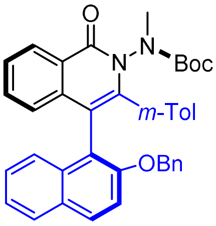


**(*S,S*)-tert-butyl (4-(2-(benzyloxy)naphthalen-1-yl)-1-oxo-3-(m-tolyl)isoquinolin-2(1H)-yl)(methyl)carbamate (41)**

The title compound was isolated as a pale-yellow solid (48.0 mg, 81%, two amide bond rotamers 58% : 42%) $^1$H NMR (600 MHz, $CDCl_3$) δ 8.62 - 8.55 (m, 1H), 7.74 – 7.63 (m, 2H), 7.56 – 7.46 (m, 2H), 7.45 - 7.40 (m, 1H), 7.39 – 7.23 (m, 4H), 7.23 – 7.12 (m, 4H), 7.08 – 7.03 (m, 1H), 7.02 – 6.92 (m, 1H), 6.89 - 6.76 (m, 2H), 6.74 – 6.62 (m, 1H), 5.18 – 4.88 (m, 2H), 3.07 (s, 1.7H, major), 3.02 (s, 1.3H, minor), 2.17 (s, 1.7H, major), 1.93 (s, 1.3H, minor), 1.44 (s, 3.7H, minor), 1.23 (s, 5.3H, major); $^{13}$C NMR (150 MHz, $CDCl_3$) δ 160.8, 160.7, 160.6, 154.89, 154.87, 154.5, 154.4, 154.1, 143.53, 143.47, 137.72, 137.69, 137.5, 137.3, 137.1, 136.5, 136.4, 136.3, 134.8, 134.4, 134.2, 133.52, 133.48, 132.92, 132.87, 129.91, 129.87, 129.8, 129.7, 129.6, 129.2, 129.1, 129.0, 128.9, 128.8, 128.59, 128.56, 128.52, 128.50, 128.42, 128.38, 128.3,

128.2, 128.1, 128.0, 127.77, 127.75, 127.4, 127.3, 127.1, 127.0, 126.9, 126.81, 126.78, 126.75, 126.7, 126.63, 126.58, 126.5, 126.2, 126.0, 125.7, 125.54, 125.46, 125.4, 125.2, 125.1, 124.9, 124.72, 123.70, 123.6, 119.2, 114.8, 114.7, 114.4, 112.5, 112.4, 112.3, 81.53, 81.45, 81.4, 81.3, 71.0, 70.9, 70.4, 70.2, 37.8, 37.7, 36.9, 36.7, 28.31, 28.28, 28.03, 27.96, 21.4, 21.1, 21.0, 20.9; **HRMS (ESI)**: calcd. for $C_{39}H_{37}N_2O_4^+$ [M+H]$^+$ : 597.2748, found :597.2748 ; **[α]$_D$$^{20}$** = -50 (c = 0.1, $CHCl_3$).

**HPLC analysis**: Daicel Chiralpak IG-H column (hexane: 2-propanol = 95:5, v = 1.0 mL/min, 40 °C, 254 nm); tr (minor) = 18.350 min, tr (major) = 35.297 min, 98% ee. tr (minor) = 14.487 min, tr (major) = 48.990 min, 39% ee. > 20:1 dr determined by crude NMR.

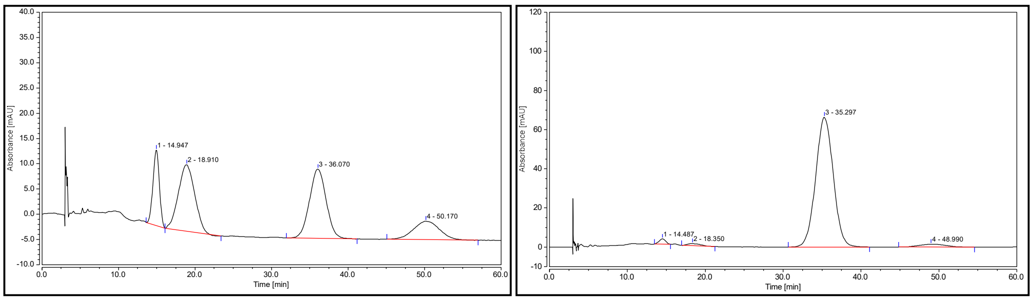


| No. | Time | Area | Area (%) | No. | Time | Area | Area (%) |
|---|---|---|---|---|---|---|---|
| 1 | 14.947 | 14.6 | 15.20 | 1 | 14.487 | 2.6 | 1.40 |
| 2 | 18.910 | 32.6 | 33.87 | 2 | 18.350 | 2.1 | 1.13 |
| 3 | 36.070 | 34.7 | 36.01 | 3 | 35.297 | 174.1 | 94.29 |
| 4 | 50.170 | 14.4 | 14.92 | 4 | 48.990 | 5.9 | 3.18 |

**Supplementary Figure 37. HPLC data of 41.**

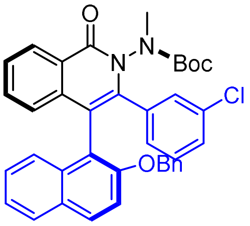


**(*S*,*S*)-tert-butyl (4-(2-(benzyloxy)naphthalen-1-yl)-3-(3-chlorophenyl)-1-oxoisoquinolin-2(1H)-yl)(methyl)carbamate (42)**

The title compound was isolated as a white solid (55.5 mg, 91%, two amide bond rotamers 63% : 37%) $^1$H NMR (600 MHz, $CDCl_3$) δ 8.61 - 855 (m, 1H), 7.76 – 7.66 (m, 2H), 7.53 – 7.42 (m, 3H), 7.40 – 7.26 (m, 3H), 7.26 – 7.15 (m, 4H), 7.14 – 7.04 (m, 2H), 7.03 - 7.01 (m, 1H), 7.00 – 6.95 (m, 1H), 6.90 – 6.77 (m, 2H), 5.10 – 4.96 (m, 2H), 3.08 (s, 1.9H, major), 3.04 (s, 1.1H, minor), 1.47 (s, 3.3H, minor), 1.23 (s, 5.7H, major); $^{13}$C NMR (150 MHz, $CDCl_3$) δ 160.5, 160.4, 160.3, 154.74, 154.71, 154.39, 154.36, 154.3, 154.1, 141.9, 141.8, 141.71, 141.69, 137.3, 137.2, 137.0, 136.9, 135.28, 135.26, 134.6, 134.2, 133.9, 133.1, 133.0, 132.92, 132.89, 132.7, 130.14, 130.06, 130.0, 129.14, 129.12, 128.9, 128.8, 128.64, 128.55, 128.51, 128.49, 128.4, 128.3, 128.2, 128.11, 128.07, 127.81, 127.78, 127.75, 127.7, 127.41, 127.35, 127.2, 127.1, 126.99, 126.97, 126.94, 126.88, 126.86, 126.8, 126.5, 126.3, 126.1, 125.83, 125.78, 125.6, 125.5, 125.4, 124.9, 124.7, 124.5, 123.9, 123.7, 123.5, 118.6, 118.4, 118.3, 114.7, 114.6, 114.3, 112.8, 112.7, 81.9, 81.70, 81.69, 81.5, 71.0, 70.9, 70.4, 70.3, 37.8, 37.6, 36.9, 36.6, 28.20, 28.17, 28.1, 27.9; **HRMS (ESI)**: calcd. for $C_{38}H_{33}ClN_2NaO_4^+$ [M+Na]$^+$ : 639.2021, found : 639.2029; **[α]$_D$$^{20}$** = -52 (c = 0.1, $CHCl_3$).

**HPLC analysis**: Daicel Chiralpak IE column (hexane: 2-propanol = 90:10, v = 1.0 mL/min, 40 °C, 254 nm); tr (minor) = 13.191 min, tr (major) = 20.021 min, 96% ee. tr (major) = 16.736 min, tr (minor) = 24.845 min, 70% ee. > 20:1 dr determined by crude NMR.

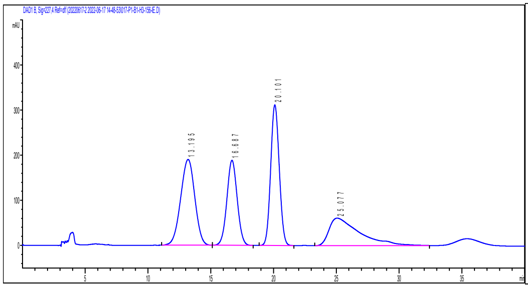

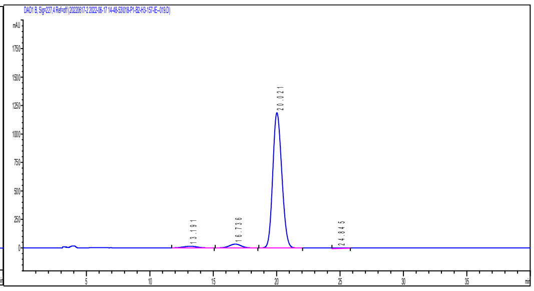

| No. | Time | Area | Area (%) | No. | Time | Area | Area (%) |
|---|---|---|---|---|---|---|---|
| 1 | 13.195 | 14887.2 | 29.30 | 1 | 13.191 | 1094 | 1.76 |
| 2 | 16.687 | 10767 | 21.19 | 2 | 16.736 | 2147.8 | 3.44 |
| 3 | 20.101 | 14800.8 | 29.13 | 3 | 20.021 | 58727.3 | 94.19 |
| 4 | 25.077 | 10356.5 | 20.38 | 4 | 24.845 | 379 | 0.61 |

**Supplementary Figure 38. HPLC data of 42.**

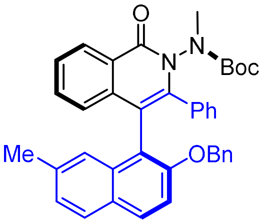


**(*S,S*)-tert-butyl (4-(2-(benzyloxy)-7-methylnaphthalen-1-yl)-1-oxo-3-phenylisoquinolin-2(1H)-yl)(methyl)carbamate (43)**

The title compound was isolated as a pale-yellow solid (58.0 mg, 97%, two amide bond rotamers 63% : 37%) $^{1}$H NMR (600 MHz, $CDCl_3$) δ 8.62 – 8.57 (m, 1H), 7.66 – 7.56 (m, 2H), 7.56 – 7.48 (m, 1H), 7.47 – 7.41 (m, 1H), 7.39 - 7.32 (m, 1H), 7.25 – 7.23 (m, 1H), 7.22 – 7.08 (m, 5H), 7.08 – 6.96 (m, 4H), 6.96 - 6.86 (m, 2H), 6.86 – 6.80 (m, 1H), 5.04 – 4.93 (m, 2H), 3.11 (s, 1.9H, major), 3.04 (s, 1.1H, minor), 2.38 (s, 1.1H, minor), 2.37 (s, 1.9H, major), 1.44 (s, 3.3H, minor), 1.20 (s, 5.7H, major); $^{13}$C NMR (150 MHz, $CDCl_3$) δ 160.9, 160.7, 154.9, 154.6, 154.5, 154.3, 143.4, 143.3, 137.8, 137.7, 137.5, 137.2, 136.53, 136.47, 135.0, 134.4, 133.7, 132.94, 132.89, 129.6, 129.5, 129.2, 129.1, 128.6, 128.52, 128.51, 128.48, 128.42, 128.38, 128.3, 128.2, 128.01, 127.98, 127.9, 127.8, 127.7, 127.6, 127.4, 127.23, 127.15, 127.1, 126.9, 126.8, 126.7, 126.6, 126.53, 126.51, 126.09, 126.07, 126.0, 125.8, 125.7, 125.6, 124.0, 123.9, 118.52, 118.46, 113.9, 113.5, 112.7, 112.6, 81.6, 81.4, 71.0, 70.4, 37.7, 36.8, 28.3, 28.0, 22.24, 22.20; **HRMS (ESI)**: calcd. for $C_{39}H_{37}N_2O_4^+$ $[M+H]^+$ : 597.2748, found :597.2743 ; $[\alpha]_D^{20}$ = -78 (c = 0.1, $CHCl_3$).

**HPLC analysis**: Daicel Chiralpak IE column (hexane: 2-propanol = 85:15, v = 1.0 mL/min, 40 °C, 254 nm); tr (minor) = 11.495 min, tr (major) = 14.320 min, 90% ee. > 20:1 dr determined by crude NMR.

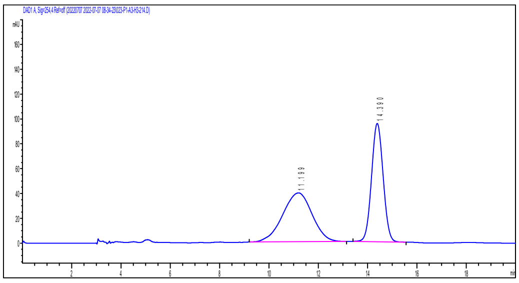

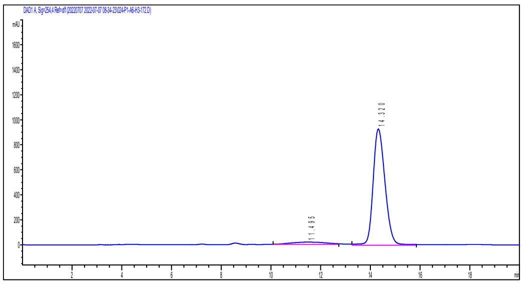

| No. | Time | Area | Area (%) | No. | Time | Area | Area (%) |
|---|---|---|---|---|---|---|---|
| 1 | 11.199 | 3276.2 | 51.28 | 1 | 11.495 | 1694.8 | 5.16 |
| 2 | 14.390 | 3112.6 | 48.72 | 2 | 14.320 | 31136.6 | 94.84 |

**Supplementary Figure 39. HPLC data of 43.**

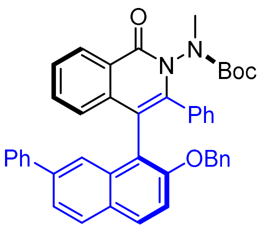

**(*S,S*)-tert-butyl (4-(2-(benzyloxy)-7-phenylnaphthalen-1-yl)-1-oxo-3-phenylisoquinolin-2(1H)-yl)(methyl)carbamate (44)**

The title compound was isolated as a pale-yellow solid (62.8 mg, 95%, two amide bond rotamers 63% : 37%) $^1$H NMR (600 MHz, $CDCl_3$) δ 8.63 - 8.57 (m, 1H), 7.82 - 7.76 (m, 1H), 7.75 – 7.65 (m, 2H), 7.59 – 7.50 (m, 2H), 7.50 – 7.44 (m, 3H), 7.43 - 7.37 (m, 3H), 7.36 – 7.27 (m, 2H), 7.25 – 7.13 (m, 4H), 7.11 – 7.05 (m, 2H), 7.03 – 7.00 (m, 1H), 6.99 - 6.97 m, 1H), 6.94 – 6.86 (m, 2H), 5.10 – 4.99 (m, 2H), 3.10 (s, 1.9H, major), 3.03 (s, 1.1H, minor), 1.45 (s, 3.3H, minor), 1.22 (s, 5.7H, major); $^{13}$C NMR (150 MHz, $CDCl_3$) δ 161.0, 160.8, 154.92, 154.89, 154.6, 154.5, 143.6, 143.5, 141.44, 141.41, 139.5, 137.7, 137.6, 137.4, 137.1, 135.0, 134.5, 133.7, 133.02, 132.96, 129.7, 129.6, 129.2, 129.1, 129.0, 128.9, 128.8, 128.7, 128.62, 128.59, 128.5, 128.4, 128.3, 128.2, 128.1, 127.9, 127.7, 127.56, 127.55, 127.52, 127.49, 127.46, 127.3, 127.2, 127.1, 126.9, 126.8, 126.6, 126.1, 125.9, 125.62, 125.55, 123.7, 123.6, 123.0, 122.9, 119.5, 119.4, 115.0, 114.5, 112.42, 112.37, 81.6, 81.5, 71.1, 70.5, 37.8, 36.9, 28.3, 28.0; **HRMS (ESI)**: calcd. for $C_{44}H_{38}N_2NaO_4^+$ $[M+Na]^+$ : 681.2724, found : 681.2725; **$[\alpha]_D^{20}$** = -120 (c = 0.1, $CHCl_3$).
**HPLC analysis**: Daicel Chiralpak IG-H column (hexane: 2-propanol = 95:5, v = 1.0 mL/min, 40 °C, 254 nm); tr (minor) = 42.083 min, tr (major) = 61.453 min, 94% ee. tr (minor) = 54.616 min, tr (major) = 90.056 min, 28% ee. 12:1 dr determined by crude NMR.

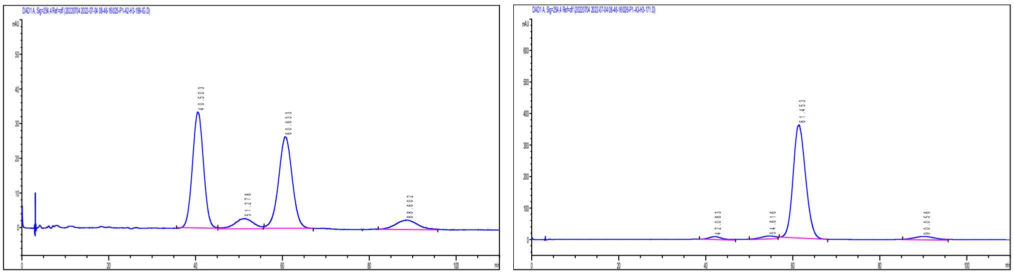

| No. | Time | Area | Area (%) | No. | Time | Area | Area (%) |
|---|---|---|---|---|---|---|---|
| 1 | 40.503 | 5898.4 | 43.72 | 1 | 42.083 | 2172.4 | 2.71 |
| 2 | 51.278 | 882.7 | 6.54 | 2 | 54.616 | 2246.7 | 2.81 |
| 3 | 60.633 | 5755.9 | 42.66 | 3 | 61.453 | 71678.2 | 89.53 |
| 4 | 88.602 | 955.2 | 7.08 | 4 | 90.056 | 3960.7 | 4.95 |

**Supplementary Figure 40. HPLC data of 44.**

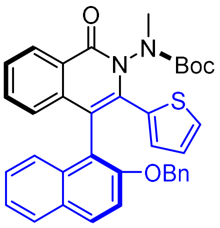

**(*S,S*)-tert-butyl (4-(2-(benzyloxy)naphthalen-1-yl)-1-oxo-3-(thiophen-2-yl)isoquinolin-2(1H)-yl)(methyl)carbamate (45)**

The title compound was isolated as a pale-yellow solid (54.6 mg, 93%, two amide bond rotamers 63% : 37%) $^1$H NMR (600 MHz, $CDCl_3$) δ 8.60 - 8.52 (m, 1H), 7.77 – 7.67 (m, 2H), 7.52 – 7.38 (m, 3H), 7.38 – 7.27 (m, 3H), 7.24 – 7.08 (m, 4H), 7.02 – 6.96 (m, 2H), 6.86 - 6.81 (m, 1H), 6.80 - 6.73 (m, 1H), 6.61 - 6.52 (m, 1H), 5.11 – 4.99 (m, 2H), 3.15 (s, 1.9H, major), 3.12 (s, 1.1H, minor), 1.47 (s, 3.3H, minor), 1.22 (s, 5.7H, major); $^{13}$C NMR (150 MHz, $CDCl_3$) δ 160.8, 160.6, 154.9, 154.42, 154.39, 154.2, 137.23, 137.20, 137.17, 136.8, 136.7, 134.8, 134.3, 133.00, 132.95, 130.1, 130.0, 128.9, 128.8, 128.7, 128.59, 128.56, 128.4, 128.3, 128.20, 128.15, 127.8, 127.4, 127.24, 127.16, 127.0, 126.92, 126.86, 126.85, 126.67, 126.65, 126.6, 126.53, 126.47, 126.2, 125.82, 125.79, 125.54, 125.50, 125.1, 125.0, 123.8, 123.7, 119.1, 119.0, 115.6, 115.5, 114.8, 114.6, 81.7, 81.5, 71.0, 70.4, 37.9, 36.7, 28.4, 28.0; **HRMS (ESI)**: calcd. for $C_{36}H_{32}N_2NaO_4S^+$ $[M+Na]^+$ : 611.1975, found : 611.1985; **$[\alpha]_D^{20}$** = -52 (c = 0.1, $CHCl_3$).

**HPLC analysis**: Daicel Chiralpak IA column (hexane: 2-propanol = 95:5, v = 1.0 mL/min, 40 °C, 254 nm); tr (minor) = 18.265 min, tr (major) = 21.283 min, 97% ee. tr (minor) = 10.362 min, tr (major) = 40.915 min, 7% ee. 19:1 dr determined by crude NMR.

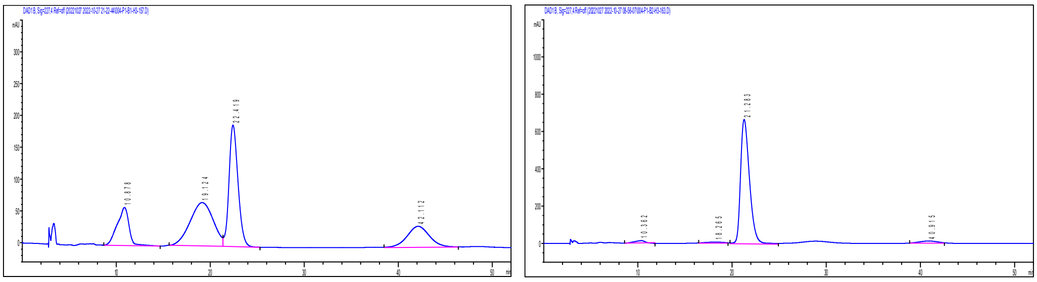

| No. | Time | Area | Area (%) | No. | Time | Area | Area (%) |
|---|---|---|---|---|---|---|---|
| 1 | 10.878 | 5233.5 | 14.87 | 1 | 10.362 | 887.1 | 1.87 |
| 2 | 19.124 | 11645.8 | 33.10 | 2 | 18.265 | 784.6 | 1.65 |
| 3 | 22.419 | 12793.8 | 36.36 | 3 | 21.283 | 44345.3 | 93.24 |
| 4 | 42.112 | 5514.4 | 15.67 | 4 | 40.915 | 1543.6 | 3.24 |

**Supplementary Figure 41. HPLC data of 45.**

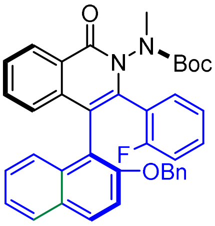


**(*S,S*)-tert-butyl (4-(2-(benzyloxy)naphthalen-1-yl)-3-(2-fluorophenyl)-1-oxoisoquinolin-2(1H)-yl)(methyl)carbamate (46)**

The title compound was isolated as a white solid (40.9 mg, 68%, two amide bond rotamers 67% : 33%) $^{1}$H NMR (600 MHz, CDCl$_3$) δ 8.62 - 8.56 (m, 1H), 7.75 – 7.66 (m, 2H), 7.64 - 7.56 (m, 1H), 7.55 - 7.48 (m, 1H), 7.47 - 7.43 (m, 1H), 7.41 – 7.26 (m, 4H), 7.26 – 7.11 (m, 4H), 7.04 – 6.97 (m, 1H), 6.96 - 6.86 (m, 2H), 6.80 – 6.75 (m, 1H), 6.73 - 6.64 (m, 1H), 5.17 – 5.02 (m, 2H), 3.28 (s, 2.0H, major), 3.25 (s, 1.0H, minor), 1.35 (s, 3.0H, minor), 1.12 (s, 6.0H, major); $^{13}$C NMR (150 MHz, CDCl$_3$) δ 160.8, 160.6, 159.0, 155.1, 154.4, 154.3, 154.1, 138.5, 137.7, 137.6, 137.3, 136.9, 134.0, 133.8, 133.0, 132.9, 131.7, 131.47, 131.46, 130.73, 130.68, 130.1, 130.0, 129.0, 128.9, 128.7, 128.5, 128.4, 128.0, 127.8, 127.7, 127.6, 127.3, 127.1, 127.0, 126.8, 126.7, 126.4, 126.2, 125.6, 125.5, 125.2, 125.1, 124.0, 123.8, 123.0, 121.7, 121.6, 119.1, 114.9, 114.8, 114.6, 114.5, 114.2, 113.9, 81.6, 81.5, 71.2, 70.5, 37.68, 37.65, 36.8, 36.7, 28.2, 27.9; **$^{19}$F NMR (376 MHz, CDCl$_3$)** δ -110.74, -111.59.; **HRMS (ESI)**: calcd. for $C_{38}H_{33}FN_2NaO_4^+$ [M+Na]$^+$ : 623.2317, found : 623.2314; **[α]$_D^{20}$** = -16 (c = 0.1, CHCl$_3$).

**HPLC analysis**: Daicel Chiralpak IE column (hexane: 2-propanol = 90:10, v = 1.0 mL/min, 40 °C, 254 nm); tr (minor) = 15.948 min, tr (major) = 26.482 min, 95% ee. tr (major) = 18.583 min, tr (minor) = 19.935 min, 4% ee. > 20:1 dr determined by crude NMR.

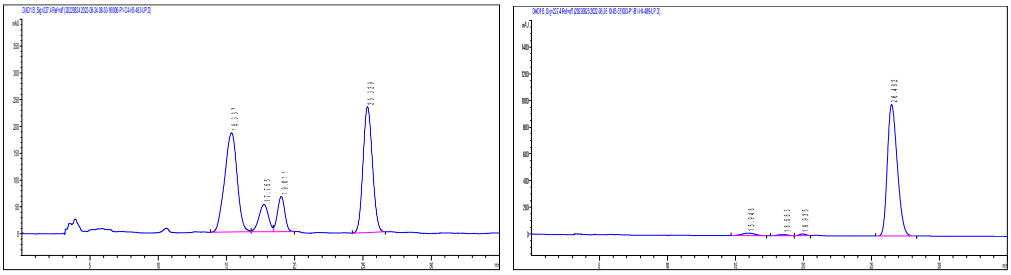

| No. | Time | Area | Area (%) | No. | Time | Area | Area (%) |
|---|---|---|---|---|---|---|---|
| 1 | 15.367 | 11762.9 | 42.19 | 1 | 15.948 | 1216.6 | 2.34 |
| 2 | 17.755 | 2445.8 | 8.77 | 2 | 18.583 | 416 | 0.80 |

| | | | | | | | |
|---|---|---|---|---|---|---|---|
| 3 | 19.011 | 2426.6 | 8.71 | 3 | 19.935 | 382.3 | 0.74 |
| 4 | 25.329 | 11242.5 | 40.33 | 4 | 26.482 | 49906.1 | 96.12 |

**Supplementary Figure 42. HPLC data of 46.**

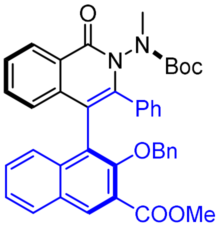


**(*S*,*S*)-methyl 3-(benzyloxy)-4-(2-((tert-butoxycarbonyl)(methyl)amino)-1-oxo-3-phenyl-1,2-dihydroisoquinolin-4-yl)-2-naphthoate (47)**

The title compound was isolated as a white solid (56.9 mg, 89%, two amide bond rotamers 51% : 49%) $^{1}H$ NMR (600 MHz, $CDCl_3$) δ 8.63 – 8.56 (m, 1H), 8.30 - 8.23 (m, 1H), 7.83 (t, *J* = 8.5 Hz, 1H), 7.61 (d, *J* = 8.1 Hz, 1H), 7.54 – 7.48 (m, 2H), 7.48 – 7.38 (m, 4H), 7.32 (t, *J* = 7.6 Hz, 1H), 7.23 (t, *J* = 7.4 Hz, 1H), 7.20 – 7.14 (m, 2H), 7.10 (t, *J* = 7.4 Hz, 1H), 7.07 – 6.99 (m, 2H), 6.97 – 6.90 (m, 1H), 6.83 – 6.70 (m, 2H), 4.85 (d, d; *J* = 10.2 Hz, 1H), 4.55 (d, d; *J* = 10.3 Hz, 1H), 3.82 (s, 1.5H, major), 3.77 (s, 1.5H, minor), 3.03 (s, 1.5H, major), 3.01 (s, 1.5H, minor), 1.47 (s, 4.4H, minor), 1.18 (s, 4.6H, major); $^{13}C$ NMR (150 MHz, $CDCl_3$) δ 166.90, 166.86, 160.7, 160.2, 154.9, 154.3, 153.5, 153.4, 144.7, 144.1, 137.7, 137.6, 137.3, 136.9, 136.2, 136.1, 133.5, 133.2, 133.10, 133.06, 129.40, 129.37, 129.32, 129.29, 129.24, 129.21, 128.9, 128.8, 128.70, 128.65, 128.62, 128.55, 128.2, 128.0, 127.8, 127.7, 127.6, 127.4, 127.22, 127.17, 127.0, 126.9, 126.6, 126.4, 126.0, 125.8, 125.74, 125.67, 125.63, 125.57, 125.5, 125.1, 111.5, 111.3, 81.74, 81.68, 76.4, 76.0, 52.4, 37.6, 36.7, 28.3, 27.9;

**HRMS (ESI)**: calcd. for $C_{40}H_{36}N_2NaO_6^+$ $[M+Na]^+$ : 663.2466, found : 663.2462; **$[\alpha]_D^{20}$** = -24 (c = 0.1, $CHCl_3$).

**HPLC analysis**: Daicel Chiralpak IE column (hexane: 2-propanol = 85:15, v = 1.0 mL/min, 40 °C, 254 nm); tr (minor) = 13.792 min, tr (major) = 17.772 min, 90% ee. > 20:1 dr determined by crude NMR.

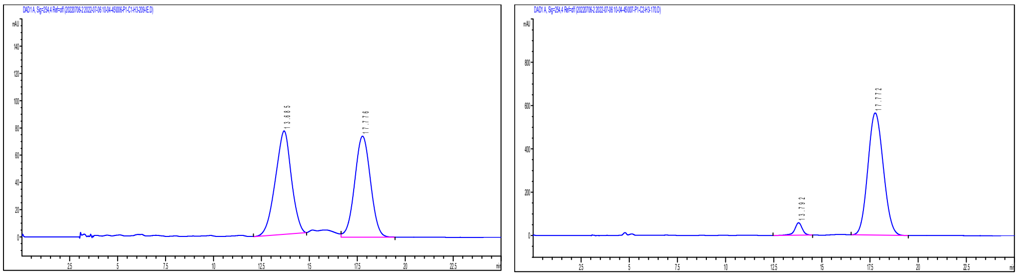

| No. | Time | Area | Area (%) | No. | Time | Area | Area (%) |
|---|---|---|---|---|---|---|---|
| 1 | 13.685 | 4404.5 | 50.87 | 1 | 13.792 | 1573 | 4.756 |
| 2 | 17.776 | 4253.6 | 49.13 | 2 | 17.772 | 31502.8 | 95.244 |

**Supplementary Figure 43. HPLC data of 47.**

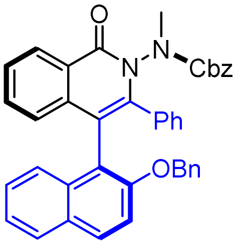


**(*S*, *S*)-benzyl (4-(2-(benzyloxy)naphthalen-1-yl)-1-oxo-3-phenylisoquinolin-2(1H)-yl)(methyl)carbamate (48)**

The title compound was isolated as a pale-yellow solid (48.0 mg, 78%, two amide bond rotamers 51% : 49%) $^{1}H$ NMR (600 MHz, $CDCl_3$) δ 8.62 - 8.57 (m, 1H), 7.74 – 7.63 (m, 2H), 7.59 – 7.26 (m, 7H), 7.25 – 7.09 (m, 8H), 7.09 - 7.05 (m, 1H), 7.04 – 6.99 (m, 2H), 6.98 – 6.94 (m, 1H), 6.90 - 6.85 (m, 1H), 6.83 – 6.73 (m, 2H), 5.32 – 4.86 (m, 4H), 3.14 - 3.09 (m, 3H); $^{13}C$ NMR (150 MHz, $CDCl_3$) δ 160.8, 160.7, 156.0, 155.8, 154.2, 154.1, 143.1, 143.0, 137.7, 137.6, 137.5, 137.4, 136.3, 136.2, 134.7, 134.38, 133.37, 133.19, 133.17, 133.1, 130.0, 129.9, 129.0, 128.91, 128.87, 128.8, 128.6, 128.52, 128.45, 128.41, 128.39, 128.37, 128.22, 128.17, 128.12, 128.07, 127.9, 127.8, 127.7, 127.6, 127.5, 127.4,

127.3, 127.00, 126.97, 126.90, 126.87, 126.86, 126.61, 126.56, 126.5, 125.9, 125.72, 125.65, 125.6, 125.5, 125.1, 125.0, 123.8, 123.7, 119.0, 115.0, 114.7, 112.9, 112.8, 70.6, 70.5, 68.14, 68.08, 37.9, 37.4; **HRMS (ESI)**: calcd. for $C_{41}H_{33}N_2O_4^+$ $[M+H]^+$ : 617.2435, found : 617.2430; **$[\alpha]_D^{20}$** = -84 (c = 0.1, $CHCl_3$).

**HPLC analysis**: Daicel Chiralpak OX-H column (hexane: 2-propanol = 85:15, v = 1.0 mL/min, 40 °C, 254 nm); tr (minor) = 21.110 min, tr (major) = 26.257 min, 96% ee. > 20:1 dr determined by crude NMR.

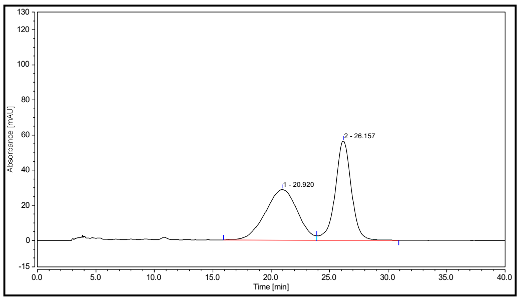
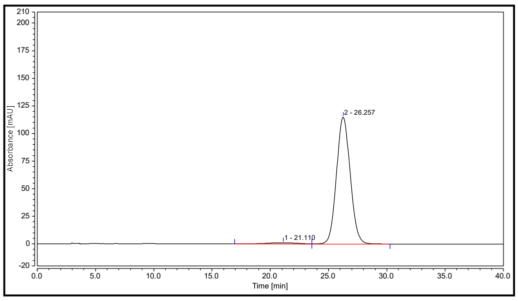

| No. | Time | Area | Area (%) | No. | Time | Area | Area (%) |
|---|---|---|---|---|---|---|---|
| 1 | 20.920 | 91.8 | 50.50 | 1 | 21.110 | 3.6 | 2.22 |
| 2 | 26.157 | 90.02 | 49.50 | 2 | 26.257 | 157.5 | 97.78 |

**Supplementary Figure 44. HPLC data of 48.**

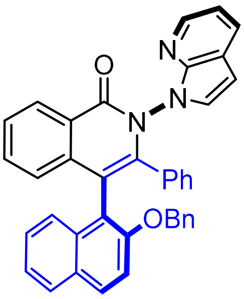

**(*S,S*)-4-(2-(benzyloxy)naphthalen-1-yl)-3-phenyl-2-(1H-pyrrolo[2,3-b]pyridin-1-yl)isoquinolin-1(2H)-one (49)**

White solid (36.4 mg, 64%) $^1$H NMR (600 MHz, $CDCl_3$) δ 8.63 – 8.55 (m, 1H), 8.38 (dd, *J* = 4.8, 1.4 Hz, 1H), 7.76 (dt, *J* = 7.9, 1.3 Hz, 1H), 7.70 (d, *J* = 8.2 Hz, 1H), 7.64 (dd, *J* = 13.0, 8.8 Hz, 2H), 7.53 – 7.45 (m, 2H), 7.41 – 7.37 (m, 1H), 7.33 – 7.24 (m, 4H), 7.24 – 7.18 (m, 4H), 7.11 (d, *J* = 3.8 Hz, 1H), 7.08 – 7.02 (m, 2H), 6.94 (dd, *J* = 7.7, 1.6 Hz, 1H), 6.87 (dd, *J* = 7.7, 1.8 Hz, 1H), 6.76 (td, *J* = 7.5, 1.2 Hz, 1H), 6.63 (t, *J* = 7.6 Hz, 1H), 6.58 (t, *J* = 7.7 Hz, 1H), 6.32 (d, *J* = 3.9 Hz, 1H), 5.09 – 4.93 (m, 2H).

$^{13}$C NMR (151 MHz, $CDCl_3$) δ 161.56, 154.16, 146.76, 143.89, 143.06, 137.64, 137.26, 134.43, 133.25, 132.37, 129.90, 129.47, 128.94, 128.73, 128.57, 128.43, 128.17, 128.10, 128.01, 127.35, 127.12, 126.82, 126.50, 126.30, 125.74, 125.59, 124.89, 123.55, 118.59, 118.36, 117.03, 114.48, 112.94, 100.52, 70.18.

**HRMS** (ESI-TOF) (m/z): Calcd for $C_{39}H_{28}N_3O_2^+$, ($[M + H]^+$), 570.2176, found 570.2169. **$[\alpha]_D^{20}$** = -16 (c = 0.1, $CHCl_3$).

**HPLC** conditions: Daicel Chiralpak IC column (80: 20 hexane: 2-propanol, 0.8 mL/min, 40 °C, 254 nm); tr (major) = 17.8 min, tr (minor) = 33.9 min, 96% e.e.. > 20:1 dr determined by crude NMR.

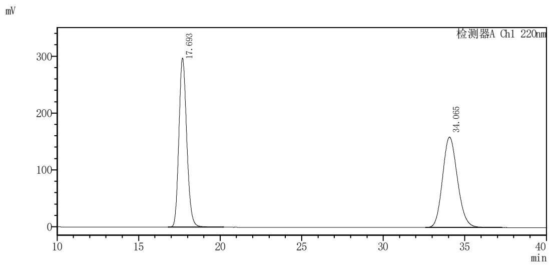
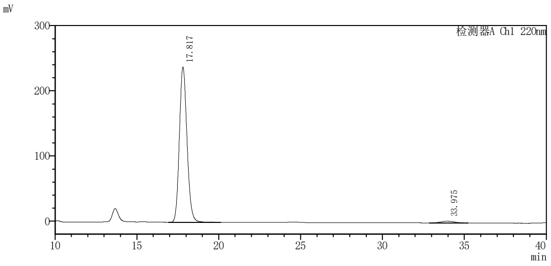

| No. | Time | Area | Area (%) | No. | Time | Area | Area (%) |
|---|---|---|---|---|---|---|---|
| 1 | 17.693 | 9935686 | 49.541 | 1 | 17.817 | 7717226 | 97.957 |

| | | | | | | | |
|---|---|---|---|---|---|---|---|
| 2 | 34.065 | 10119786 | 50.459 | 2 | 33.975 | 160920 | 2.043 |

**Supplementary Figure 45. HPLC data of 49.**

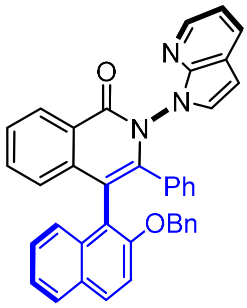


**(*R,S*)-4-(2-(benzyloxy)naphthalen-1-yl)-3-phenyl-2-(1H-pyrrolo[2,3-b]pyridin-1-yl)isoquinolin-1(2H)-one (*dia*-49)**

White solid (46.1 mg, 81%) $^{1}$H NMR (600 MHz, $CDCl_3$) δ 8.58 (dd, $J$ = 7.7, 1.6 Hz, 1H), 8.33 (dd, $J$ = 4.8, 1.5 Hz, 1H), 7.81 (dd, $J$ = 8.6, 1.1 Hz, 1H), 7.75 – 7.69 (m, 2H), 7.67 (dd, $J$ = 8.1, 1.3 Hz, 1H), 7.53 – 7.44 (m, 2H), 7.38 (ddd, $J$ = 8.3, 6.8, 1.3 Hz, 1H), 7.31 – 7.25 (m, 4H), 7.22 – 7.18 (m, 2H), 7.16 (dd, $J$ = 7.3, 2.3 Hz, 2H), 7.06 (d, $J$ = 3.8 Hz, 1H), 7.02 (dd, $J$ = 7.8, 4.8 Hz, 1H), 6.96 – 6.88 (m, 2H), 6.82 (tt, $J$ = 7.7, 3.9 Hz, 1H), 6.72 (tt, $J$ = 7.5, 1.3 Hz, 1H), 6.39 (td, $J$ = 7.6, 1.3 Hz, 1H), 6.30 (d, $J$ = 3.8 Hz, 1H), 5.13 (s, 2H).

$^{13}$C NMR (151 MHz, $CDCl_3$) δ 161.62, 154.23, 146.83, 144.04, 142.99, 137.66, 137.31, 134.00, 133.27, 132.33, 129.96, 129.19, 128.77, 128.68, 128.49, 128.37, 128.18, 128.14, 127.79, 127.75, 127.09, 126.87, 126.55, 126.43, 125.59, 125.55, 125.32, 123.75, 118.85, 118.53, 117.01, 114.05, 112.85, 100.29, 70.48.

**HRMS** (ESI-TOF) (m/z): Calcd for $C_{39}H_{28}N_3O_2^+$, ($[M + H]^+$), 570.2176, found 570.2166. **$[\alpha]_D^{20}$** = +24 (c = 0.1, $CHCl_3$).

**HPLC** conditions: Daicel Chiralpak IC column (80: 20 hexane: 2-propanol, 0.8 mL/min, 40 °C, 254 nm); tr (major) = 13.5 min, tr (minor) = 23.6 min, 93% e.e.. < 1:20 dr determined by crude NMR.

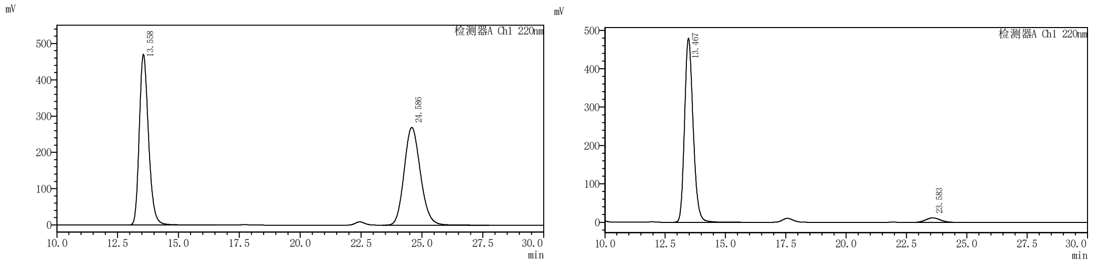


| No. | Time | Area | Area (%) | No. | Time | Area | Area (%) |
|---|---|---|---|---|---|---|---|
| 1 | 13.558 | 12437565 | 49.730 | 1 | 13.467 | 17461868 | 96.784 |
| 2 | 24.586 | 12572382 | 50.270 | 2 | 23.583 | 580234 | 3.216 |

**Supplementary Figure 46. HPLC data of *dia*-49.**

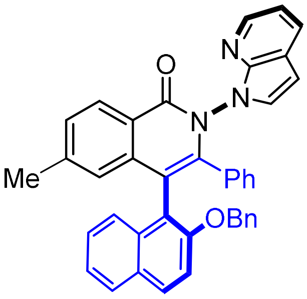


**(*S,S*)-4-(2-(benzyloxy)naphthalen-1-yl)-6-methyl-3-phenyl-2-(1H-pyrrolo[2,3-b]pyridin-1-yl)isoquinolin-1(2H)-one (50)**

White solid (40.8 mg, 70%) $^{1}$H NMR (600 MHz, $CDCl_3$) δ 8.47 (d, $J$ = 8.2 Hz, 1H), 8.34 (dd, $J$ = 4.8, 1.6 Hz, 1H), 7.78 – 7.72 (m, 2H), 7.70 (d, $J$ = 9.0 Hz, 1H), 7.64 (d, $J$ = 8.5 Hz, 1H), 7.42 (ddd, $J$ = 8.4, 6.9, 1.5 Hz, 1H), 7.37 – 7.30 (m, 6H), 7.29 – 7.26 (m, 1H), 7.21 (dd, $J$ = 7.9, 1.7 Hz, 1H), 7.15 – 7.09 (m, 2H), 7.04 (ddd, $J$ = 7.8, 4.7, 1.2 Hz, 1H), 6.86 (dd, $J$ = 7.7, 1.8 Hz, 1H), 6.76 (td, $J$ = 7.5, 1.4 Hz, 1H), 6.70 (s, 1H), 6.63 (t, $J$ = 7.6 Hz, 1H), 6.57 (t, $J$ = 7.7 Hz, 1H), 6.33 (dd, $J$ = 3.8, 1.2 Hz, 1H), 5.12 (d, $J$ = 13.1 Hz, 1H), 5.08 (d, $J$ = 13.0 Hz, 1H), 2.24 (s, 3H).

$^{13}$C NMR (151 MHz, $CDCl_3$) δ 161.54, 154.07, 147.04, 144.19, 144.03, 143.09, 137.71, 137.34, 134.50, 132.52, 129.83, 129.09, 128.78, 128.75, 128.58, 128.53, 128.50, 128.06, 128.00, 127.42, 126.78, 126.50,

126.45, 126.40, 125.35, 125.02, 123.55, 123.25, 118.70, 118.39, 116.96, 114.43, 112.67, 100.36, 70.10, 22.06.

**HRMS** (ESI-TOF) (m/z): Calcd for $C_{40}H_{30}N_3O_2^+$, ([M + H]$^+$), 584.2333, found 584.2332 . **$[\alpha]_D^{20}$** = -37 (c = 0.1, $CHCl_3$).

**HPLC** conditions: Daicel Chiralpak OD-3 column (80: 20 hexane: 2-propanol, 0.8 mL/min, 40 ºC, 254 nm); tr (major) = 18.1 min, tr (minor) = 19.5 min, 96% e.e.. > 20:1 dr determined by crude NMR.

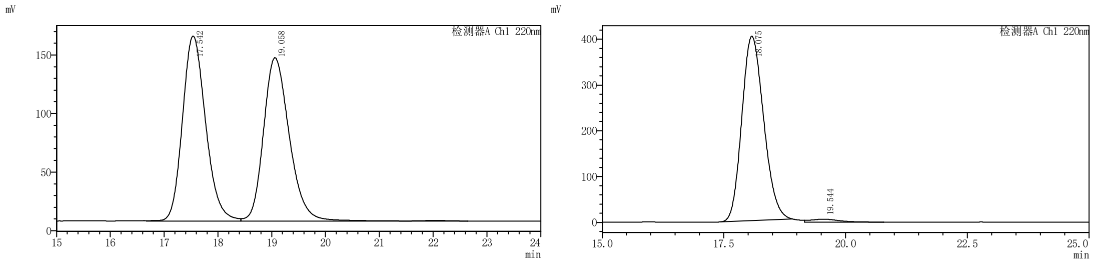

| No. | Time | Area | Area (%) | No. | Time | Area | Area (%) |
|---|---|---|---|---|---|---|---|
| 1 | 17.542 | 4734564 | 50.354 | 1 | 18.075 | 12582029 | 97.746 |
| 2 | 19.058 | 4668000 | 49.646 | 2 | 19.544 | 290130 | 2.254 |

**Supplementary Figure 47. HPLC data of 50.**

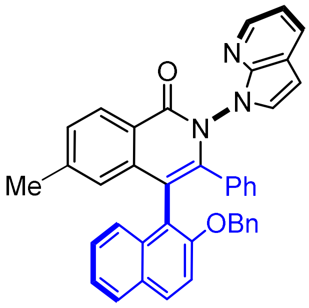

**(*R,S*)4-(2-(benzyloxy)naphthalen-1-yl)-6-methyl-3-phenyl-2-(1H-pyrrolo[2,3-b]pyridin-1-yl)isoquinolin-1(2H)-one (*dia*-50)**

White solid (49.0 mg, 84%) $^1$H NMR (600 MHz, $CDCl_3$) δ 8.46 (dd, *J* = 8.1, 3.2 Hz, 1H), 8.34 (dd, *J* = 4.8, 1.6 Hz, 1H), 7.82 (d, *J* = 8.5 Hz, 1H), 7.76 – 7.64 (m, 3H), 7.39 (dd, *J* = 8.5, 6.9 Hz, 1H), 7.36 – 7.30 (m, 2H), 7.30 – 7.27 (m, 3H), 7.16-7.21 (m, 4H), 7.09 – 7.01 (m, 2H), 6.88 (d, *J* = 7.9 Hz, 1H), 6.80 (t, *J* = 7.6 Hz, 1H), 6.74 – 6.68 (m, 2H), 6.38 (t, *J* = 7.7 Hz, 1H), 6.30 (dd, *J* = 3.8, 1.3 Hz, 1H), 5.16 (s, 2H), 2.25 (s, 3H).

$^{13}$C NMR (151 MHz, $CDCl_3$) δ 161.60, 154.17, 146.87, 144.00, 142.92, 137.77, 137.39, 134.05, 132.44, 129.87, 129.21, 128.85, 128.78, 128.76, 128.70, 128.51, 128.35, 128.16, 128.07, 127.75, 127.44, 126.80, 126.55, 126.40, 125.44, 125.25, 123.72, 123.36, 119.01, 118.55, 116.98, 113.99, 112.77, 100.23, 70.35, 22.04.

**HRMS** (ESI-TOF) (m/z): Calcd for $C_{40}H_{30}N_3O_2^+$, ([M + H]$^+$), 584.2333, found 584.2327. **$[\alpha]_D^{20}$** = +34 (c = 0.1, $CHCl_3$).

**HPLC** conditions: Daicel Chiralpak IC column (80: 20 hexane: 2-propanol, 0.8 mL/min, 40 ºC, 254 nm); tr (major) = 18.1 min, tr (minor) = 36.3 min, 87% e.e.. dr=1:8 determined by crude NMR.

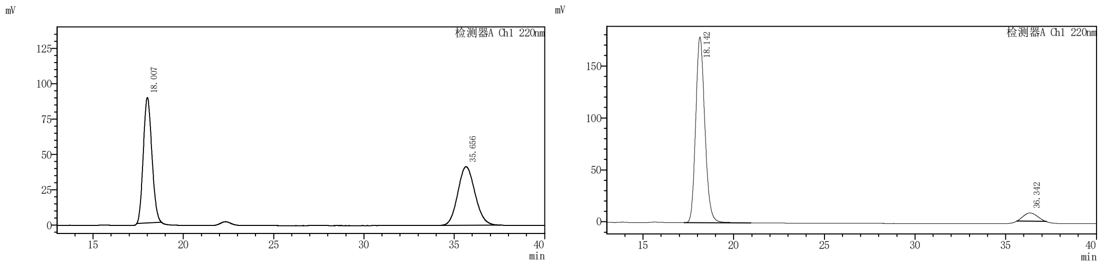

| No. | Time | Area | Area (%) | No. | Time | Area | Area (%) |
|---|---|---|---|---|---|---|---|
| 1 | 18.007 | 2931037 | 51.598 | 1 | 18.142 | 6290461 | 93.647 |
| 2 | 35.656 | 2749534 | 48.402 | 2 | 36.342 | 426765 | 6.353 |

**Supplementary Figure 48. HPLC data of dia-50.**

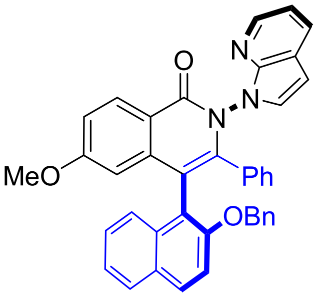


**(*S,S*)-4-(2-(benzyloxy)naphthalen-1-yl)-6-methoxy-3-phenyl-2-(1H-pyrrolo[2,3-b]pyridin-1-yl)isoquinolin-1(2H)-one (51)**

White solid (47.9 mg, 80%) $^{1}$H NMR (600 MHz, $CDCl_3$) δ 8.53 (d, *J* = 8.9 Hz, 1H), 8.46 (dd, *J* = 4.9, 1.5 Hz, 1H), 7.83 (dd, *J* = 7.9, 1.4 Hz, 1H), 7.70 (td, *J* = 8.5, 1.5 Hz, 2H), 7.66 – 7.61 (m, 2H), 7.46 – 7.40 (m, 2H), 7.31 (ddd, *J* = 8.1, 6.8, 1.2 Hz, 1H), 7.23 – 7.20 (m, 3H), 7.15 – 7.10 (m, 4H), 7.01 (d, *J* = 9.1 Hz, 1H), 6.86 (dt, *J* = 7.7, 1.5 Hz, 1H), 6.78 (tt, *J* = 7.5, 1.3 Hz, 1H), 6.63 (dtd, *J* = 15.0, 7.7, 1.3 Hz, 2H), 6.35 (d, *J* = 3.8 Hz, 1H), 6.32 (d, *J* = 2.5 Hz, 1H), 5.08 (d, *J* = 13.5 Hz, 1H), 4.99 (d, *J* = 13.5 Hz, 1H), 3.56 (s, 3H).

$^{13}$C NMR (151 MHz, $CDCl_3$) δ 168.03, 163.70, 161.27, 154.11, 143.44, 143.11, 139.90, 137.35, 134.32, 132.39, 131.04, 130.26, 129.97, 129.07, 128.65, 128.39, 128.25, 128.10, 127.96, 127.36, 126.84, 126.58, 126.27, 124.82, 123.54, 119.39, 119.08, 118.15, 117.07, 115.46, 114.39, 112.95, 108.16, 100.65, 70.11, 55.22.

**HRMS** (ESI-TOF) (m/z): Calcd for $C_{40}H_{30}N_3O_3^+$, ([M + H]$^+$), 600.2282, found 600.2270. **$[\alpha]_D^{20}$** = -34 (c = 0.1, $CHCl_3$).

**HPLC** conditions: Daicel Chiralpak OD-3 column (90: 10 hexane: 2-propanol, 0.8 mL/min, 40 °C, 254 nm); tr (major) = 22.8 min, tr (minor) = 24.9 min, 98% e.e.. > 20:1 dr determined by crude NMR.

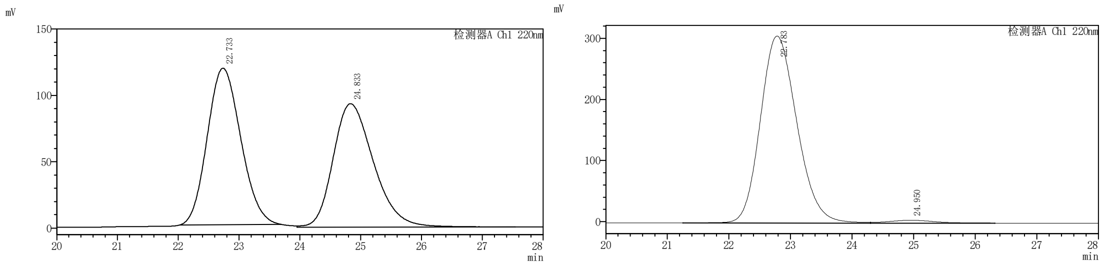


| No. | Time | Area | Area (%) | No. | Time | Area | Area (%) |
|---|---|---|---|---|---|---|---|
| 1 | 22.733 | 4525591 | 51.288 | 1 | 22.783 | 2725708 | 99.122 |
| 2 | 24.833 | 4298372 | 48.712 | 2 | 24.950 | 241437 | 0.878 |

**Supplementary Figure 49. HPLC data of 51.**

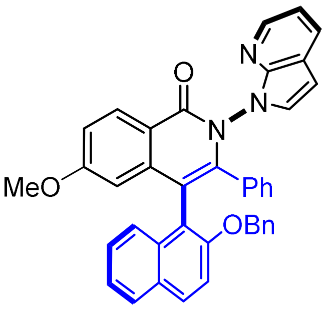


**(*R,S*)-4-(2-(benzyloxy)naphthalen-1-yl)-6-methoxy-3-phenyl-2-(1H-pyrrolo[2,3-b]pyridin-1-yl)isoquinolin-1(2H)-one (*dia*-51)**

White solid (52.1 mg, 87%) $^{1}$H NMR (600 MHz, $CDCl_3$) δ 8.50 (dd, *J* = 8.9, 3.9 Hz, 1H), 8.34 (d, *J* = 5.0 Hz, 1H), 7.83 (d, *J* = 8.5 Hz, 1H), 7.75 – 7.63 (m, 3H), 7.44 – 7.36 (m, 1H), 7.34 – 7.23 (m, 5H), 7.16-7.22 (m, 3H), 7.10 – 7.05 (m, 2H), 7.05 – 7.00 (m, 1H), 6.89 (d, *J* = 7.9 Hz, 1H), 6.81 (t, *J* = 7.6 Hz, 1H), 6.74 – 6.67 (m, 1H), 6.39 (t, *J* = 7.7 Hz, 1H), 6.32 – 6.23 (m, 2H), 5.15 (s, 2H), 3.53 (s, 3H).

$^{13}$C NMR (151 MHz, $CDCl_3$) δ 163.58, 161.28, 154.12, 146.87, 143.99, 143.58, 139.85, 137.37, 133.94, 132.45, 130.94, 129.98, 129.20, 128.92, 128.81, 128.51, 128.29, 128.13, 128.10, 127.77, 127.76, 126.86, 126.58, 126.54, 126.40, 125.33, 123.75, 119.30, 118.90, 118.54, 116.96, 115.31, 113.98, 112.55, 107.87, 100.20, 70.43, 55.21.

**HRMS** (ESI-TOF) (m/z): Calcd for $C_{40}H_{30}N_3O_3^+$, ([M + H]$^+$), 600.2282, found 600.2279. **[α]$_D^{20}$** = +51 (c = 0.1, $CHCl_3$).

**HPLC** conditions: Daicel Chiralpak IC column (80: 20 hexane: 2-propanol, 0.8 mL/min, 40 °C, 254 nm); tr (major) = 22.7 min, tr (minor) = 42.5 min, 93% e.e.. < 1:20 dr was determined by crude NMR.

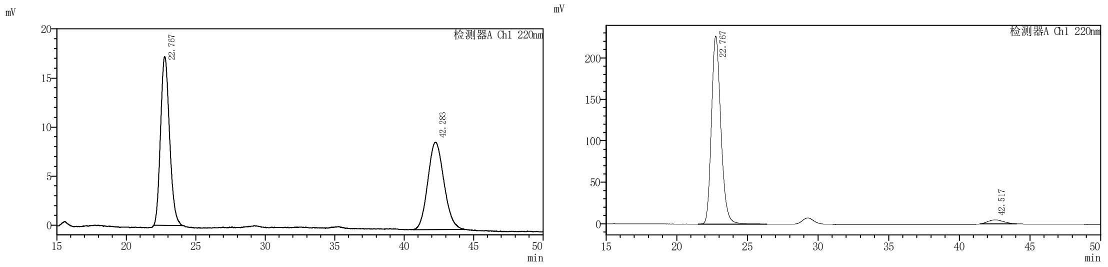


| No. | Time | Area | Area (%) | No. | Time | Area | Area (%) |
|---|---|---|---|---|---|---|---|
| 1 | 22.767 | 774730 | 51.811 | 1 | 22.767 | 10505505 | 96.749 |
| 2 | 42.283 | 720574 | 48.189 | 2 | 42.517 | 353012 | 3.251 |

**Supplementary Figure 50. HPLC data of dia-51.**

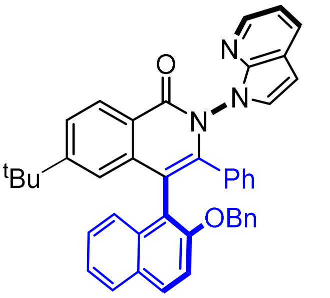


**(*S,S*)-4-(2-(benzyloxy)naphthalen-1-yl)-6-(tert-butyl)-3-phenyl-2-(1H-pyrrolo[2,3-b]pyridin-1-yl)isoquinolin-1(2H)-one (52)**

White solid (48.1 mg,77%) $^1$H NMR (600 MHz, $CDCl_3$) δ 8.50 (dd, $J$ = 8.5, 2.0 Hz, 1H), 8.34 (dd, $J$ = 4.5, 2.1 Hz, 1H), 7.76 (ddd, $J$ = 7.8, 2.4, 1.5 Hz, 1H), 7.74 – 7.70 (m, 1H), 7.68 (dd, $J$ = 9.0, 2.0 Hz, 1H), 7.66 – 7.62 (m, 1H), 7.57 (dt, $J$ = 8.5, 2.1 Hz, 1H), 7.40 (dddd, $J$ = 8.4, 6.8, 2.5, 1.3 Hz, 1H), 7.36 – 7.29 (m, 5H), 7.28 – 7.20 (m, 3H), 7.11 – 7.02 (m, 3H), 6.91 (d, $J$ = 2.1 Hz, 1H), 6.89 (dd, $J$ = 7.7, 1.9 Hz, 1H), 6.78 (tdd, $J$ = 7.5, 2.4, 1.3 Hz, 1H), 6.65 (tt, $J$ = 7.6, 1.9 Hz, 1H), 6.62 – 6.56 (m, 1H), 6.33 (dd, $J$ = 3.9, 2.2 Hz, 1H), 5.10 (s, 2H), 1.08 (s, 9H).

$^{13}$C NMR (151 MHz, $CDCl_3$) δ 161.43, 156.69, 154.07, 147.11, 144.20, 143.03, 137.48, 137.44, 134.42, 132.65, 129.87, 129.08, 128.63, 128.60, 128.52, 128.49, 128.14, 128.05, 128.02, 127.36, 126.63, 126.45, 126.27, 125.10, 125.09, 123.51, 123.17, 121.88, 118.67, 118.40, 116.96, 114.49, 113.05, 100.36, 70.20, 30.86.

**HRMS** (ESI-TOF) (m/z): Calcd for $C_{43}H_{36}N_3O_2^+$, ([M + H]$^+$), 626.2802, found 626.2791. **[α]$_D^{20}$** = -45 (c = 0.1, $CHCl_3$).

**HPLC** conditions: Daicel Chiralpak IC column (80: 20 hexane: 2-propanol, 0.8 mL/min, 40 °C, 254 nm); tr (major) = 16.5 min, tr (minor) = 12.6 min, 98% e.e. > 20:1 dr was determined by crude NMR.

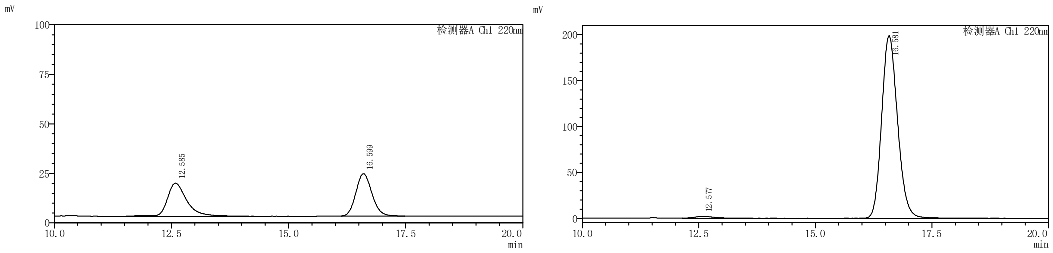


| No. | Time | Area | Area (%) | No. | Time | Area | Area (%) |
|---|---|---|---|---|---|---|---|
| 1 | 12.583 | 459199 | 49.591 | 1 | 12.575 | 51253 | 1.072 |
| 2 | 16.600 | 466771 | 50.409 | 2 | 16.583 | 4730821 | 98.928 |

**Supplementary Figure 51. HPLC data of 52.**

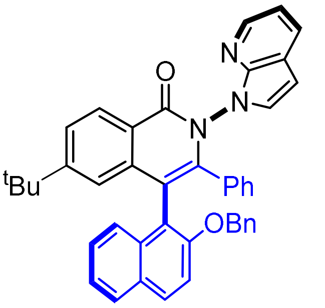

**(*R,S*)-4-(2-(benzyloxy)naphthalen-1-yl)-6-(tert-butyl)-3-phenyl-2-(1H-pyrrolo[2,3-b]pyridin-1-yl)isoquinolin-1(2H)-one (*dia*-52)**

White solid (49.4 mg,79%) [1]H NMR (600 MHz, $CDCl_3$) δ 8.53 (dd, *J* = 8.5, 1.6 Hz, 1H), 8.37 – 8.31 (m, 1H), 7.94 – 7.84 (m, 1H), 7.77 – 7.70 (m, 2H), 7.68 (d, *J* = 8.2 Hz, 1H), 7.60 (dt, *J* = 8.6, 1.7 Hz, 1H), 7.40 (ddd, *J* = 9.7, 6.3, 1.2 Hz, 1H), 7.33 – 7.25 (m, 5H), 7.23 – 7.18 (m, 2H), 7.16 – 7.11 (m, 2H), 7.08 (dd, *J* = 3.8, 1.5 Hz, 1H), 7.04 (ddd, *J* = 7.1, 4.8, 1.4 Hz, 1H), 6.98 (d, *J* = 1.8 Hz, 1H), 6.94 – 6.90 (m, 1H), 6.83 (t, *J* = 7.7 Hz, 1H), 6.73 (tt, *J* = 7.4, 1.2 Hz, 1H), 6.40 (t, *J* = 7.7 Hz, 1H), 6.31 (dt, *J* = 3.9, 1.1 Hz, 1H), 5.23 – 5.09 (m, 2H), 1.11 (s, 9H).

[13]C NMR (151 MHz, $CDCl_3$) δ 161.55, 156.79, 154.28, 146.90, 144.01, 142.86, 137.53, 137.42, 133.96, 132.55, 129.98, 129.22, 128.90, 128.78, 128.53, 128.50, 128.26, 128.09, 127.72, 127.70, 126.71, 126.44, 126.35, 125.52, 125.15, 123.71, 123.30, 121.65, 118.96, 118.57, 117.00, 113.95, 113.22, 100.23, 70.55, 30.89.

**HRMS** (ESI-TOF) (m/z): Calcd for $C_{43}H_{36}N_3O_2^+$, ([M + H]$^+$), 626.2802, found 626.2800. **$[\alpha]_D^{20}$** = +28 (c = 0.1, $CHCl_3$).

**HPLC** conditions: Daicel Chiralpak IC column (80: 20 hexane: 2-propanol, 0.8 mL/min, 40 °C, 254 nm); tr (major) = 8.9 min, tr (minor) = 11.5 min, 91% e.e.. dr = 1:7 was determined by crude NMR.

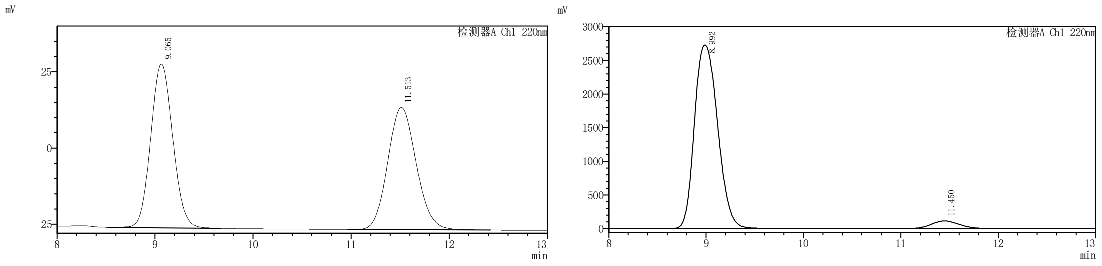


| No. | Time | Area | Area (%) | No. | Time | Area | Area (%) |
|---|---|---|---|---|---|---|---|
| 1 | 9.067 | 840883 | 50.904 | 1 | 8.992 | 45114146 | 95.367 |
| 2 | 11.517 | 811019 | 49.096 | 2 | 11.450 | 2191905 | 4.633 |

**Supplementary Figure 52. HPLC data of dia-52.**

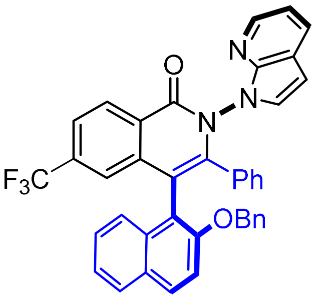

**(*S,S*)-4-(2-(benzyloxy)naphthalen-1-yl)-3-phenyl-2-(1H-pyrrolo[2,3-b]pyridin-1-yl)-6-(trifluoromethyl)isoquinolin-1(2H)-one (53)**

White solid (36.3 mg, 57%) [1]H NMR (600 MHz, $CDCl_3$) δ 8.59 (d, *J* = 8.4 Hz, 1H), 8.24 (dd, *J* = 4.8, 1.5 Hz, 1H), 7.67 (dd, *J* = 7.8, 1.5 Hz, 1H), 7.65 – 7.58 (m, 3H), 7.49 (dd, *J* = 8.5, 1.1 Hz, 1H), 7.32 (ddd, *J* = 8.3, 6.8, 1.3 Hz, 1H), 7.27 – 7.18 (m, 5H), 7.18 – 7.15 (m, 1H), 7.12 (dt, *J* = 8.4, 1.1 Hz, 2H), 7.06 – 7.01 (m, 2H), 6.97 (dd, *J* = 7.8, 4.8 Hz, 1H), 6.81 (dt, *J* = 7.8, 1.6 Hz, 1H), 6.68 (tt, *J* = 7.5, 1.3 Hz, 1H), 6.56 (td, *J* = 7.6, 1.3 Hz, 1H), 6.50 (td, *J* = 7.7, 1.3 Hz, 1H), 6.26 (d, *J* = 3.8 Hz, 1H), 5.13 – 4.99 (m, 2H).

[13]C NMR (151 MHz, $CDCl_3$) δ 160.82, 154.26, 146.73, 144.84, 144.16, 138.00, 137.15, 134.96 (q, *J* = 32.1 HZ ), 134.16, 131.93, 130.55, 129.89, 129.44, 128.76, 128.60, 128.47, 128.42, 128.40, 128.34,

127.87, 127.79, 127.60, 127.11, 126.62, 126.57, 126.44, 124.44 (q, *J* = 276.2 HZ ), 123.77, 123.24 (q, *J* = 3.3 HZ ), 122.87 (q, *J* = 3.9 HZ ), 118.57, 117.24, 114.29, 112.77, 100.84, 70.24.

**HRMS** (ESI-TOF) (m/z): Calcd for $C_{40}H_{27}F_3N_3O_2^+$, ([M + H]$^+$), 638.2050, found 638.2054. **[α]$_D^{20}$** = -56 (c = 0.1, $CHCl_3$).

**HPLC** conditions: Daicel Chiralpak IC column (80: 20 hexane: 2-propanol, 0.8 mL/min, 40 °C, 254 nm); tr (major) = 12.4 min, tr (minor) = 16.3 min, 98% e.e.. > 20:1 dr was determined by crude NMR.

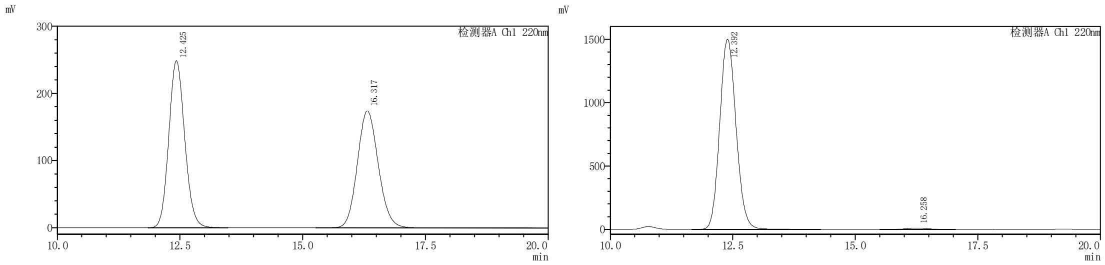


| No. | Time | Area | Area (%) | No. | Time | Area | Area (%) |
|---|---|---|---|---|---|---|---|
| 1 | 12.425 | 5628746 | 51.878 | 1 | 12.392 | 34829761 | 99.074 |
| 2 | 16.317 | 5221316 | 48.122 | 2 | 16.258 | 325512 | 0.926 |

**Supplementary Figure 53. HPLC data of 53.**

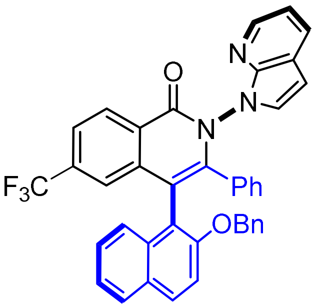


**(*R,S*)-4-(2-(benzyloxy)naphthalen-1-yl)-3-phenyl-2-(1H-pyrrolo[2,3-b]pyridin-1-yl)-6-(trifluoromethyl)isoquinolin-1(2H)-one (*dia*-53)**

White solid (45.9 mg, 72%) $^1$H NMR (600 MHz, $CDCl_3$) δ 8.69 (d, *J* = 8.4 Hz, 1H), 8.36 (dd, *J* = 4.8, 1.5 Hz, 1H), 7.79 – 7.75 (m, 2H), 7.75 – 7.70 (m, 2H), 7.68 (d, *J* = 8.1 Hz, 1H), 7.40 (ddd, *J* = 8.4, 6.8, 1.3 Hz, 1H), 7.33 – 7.26 (m, 4H), 7.24 (d, *J* = 1.7 Hz, 1H), 7.20 (d, *J* = 9.1 Hz, 1H), 7.19 – 7.13 (m, 3H), 7.07 (dd, *J* = 7.8, 4.8 Hz, 1H), 7.04 (d, *J* = 3.8 Hz, 1H), 6.88 (d, *J* = 7.8 Hz, 1H), 6.83 (t, *J* = 7.6 Hz, 1H), 6.77 – 6.72 (m, 1H), 6.40 (t, *J* = 7.7 Hz, 1H), 6.33 (d, *J* = 3.8 Hz, 1H), 5.18 (q, *J* = 12.6 Hz, 2H).

$^{13}$C NMR (151 MHz, $CDCl_3$) δ 160.83, 154.30, 146.77, 144.61, 144.18, 137.99, 137.12, 134.93, 133.72, 131.85, 130.57 (q, *J* = 34.4 HZ ), 129.81, 129.44, 128.58, 128.46, 128.40, 128.20, 127.96, 127.93, 127.90, 127.57, 127.10, 126.60, 126.56, 126.47, 124.86 (q, *J* = 272.4 HZ ), 123.88, 123.19 (q, *J* = 3.5 HZ ), 122.76 (q, *J* = 3.7 HZ ), 118.59, 117.40, 117.25, 113.71, 112.81, 100.69, 70.42.

**HRMS** (ESI-TOF) (m/z): Calcd for $C_{40}H_{27}F_3N_3O_2^+$, ([M + H]$^+$), 638.2050, found 638.2047. **[α]$_D^{20}$** = +42 (c = 0.1, $CHCl_3$).

**HPLC** conditions: Daicel Chiralpak IC column (80: 20 hexane: 2-propanol, 0.8 mL/min, 40 °C, 254 nm); tr (major) = 10.8 min, tr (minor) = 12.1 min, 96% e.e.. < 1:20 dr was determined by crude NMR.

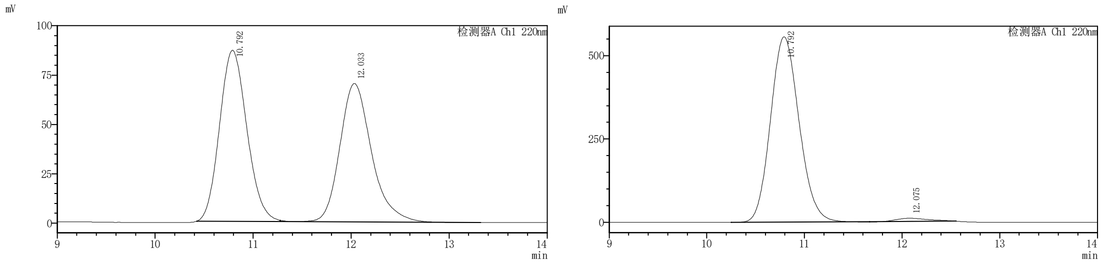

| No. | Time | Area | Area (%) | No. | Time | Area | Area (%) |
|---|---|---|---|---|---|---|---|
| 1 | 10.792 | 1680013 | 51.590 | 1 | 10.792 | 11307645 | 98.108 |
| 2 | 12.033 | 1576448 | 48.410 | 2 | 12.075 | 218009 | 1.892 |

**Supplementary Figure 54. HPLC data of dia-53.**

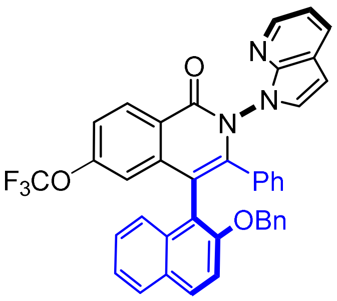


**(*S*,*S*)-4-(2-(benzyloxy)naphthalen-1-yl)-3-phenyl-2-(1H-pyrrolo[2,3-b]pyridin-1-yl)-6-(trifluoromethoxy)isoquinolin-1(2H)-one (54)**

White solid (34.0 mg, 52%), $^{1}$H NMR (600 MHz, $CDCl_3$) δ 8.53 (d, *J* = 8.9 Hz, 1H), 8.26 (dd, *J* = 4.8, 1.5 Hz, 1H), 7.70 (dd, *J* = 7.8, 1.4 Hz, 1H), 7.67 – 7.60 (m, 2H), 7.51 (dd, *J* = 8.6, 1.1 Hz, 1H), 7.34 (ddd, *J* = 8.3, 6.8, 1.3 Hz, 1H), 7.28 – 7.22 (m, 4H), 7.22 – 7.17 (m, 3H), 7.13 (dt, *J* = 7.9, 1.6 Hz, 1H), 7.05 – 6.97 (m, 3H), 6.81 (d, *J* = 7.8 Hz, 1H), 6.74 – 6.69 (m, 1H), 6.59 (ddd, *J* = 15.3, 7.4, 1.8 Hz, 2H), 6.53 (td, *J* = 7.6, 1.3 Hz, 1H), 6.27 (d, *J* = 3.8 Hz, 1H), 5.03 (q, *J* = 13.1 Hz, 2H).

$^{13}$C NMR (151 MHz, $CDCl_3$) δ 159.70, 153.18, 152.03, 145.70, 143.76, 143.03, 138.70, 136.14, 133.05, 131.00, 130.41, 129.37, 128.44, 127.69, 127.52, 127.42, 127.38, 127.35, 127.22, 126.82, 126.50, 126.01, 125.58, 125.56, 125.37, 123.47, 122.70, 118.21, 117.55, 116.41, 116.15, 115.60, 113.36, 111.37, 99.72, 69.30.

**HRMS** (ESI-TOF) (m/z): Calcd for $C_{40}H_{27}F_3N_3O_3^+$, ($[M + H]^+$), 654.1999, found 654.2004. **$[\alpha]_D^{20}$** = -36 (c = 0.1, $CHCl_3$).

**HPLC** conditions: Daicel Chiralpak IC column (80: 20 hexane: 2-propanol, 0.8 mL/min, 40 °C, 254 nm); tr (major) = 31.8 min, tr (minor) = 15.2 min, 98% e.e.. >1:20 dr was determined by crude NMR analysis.

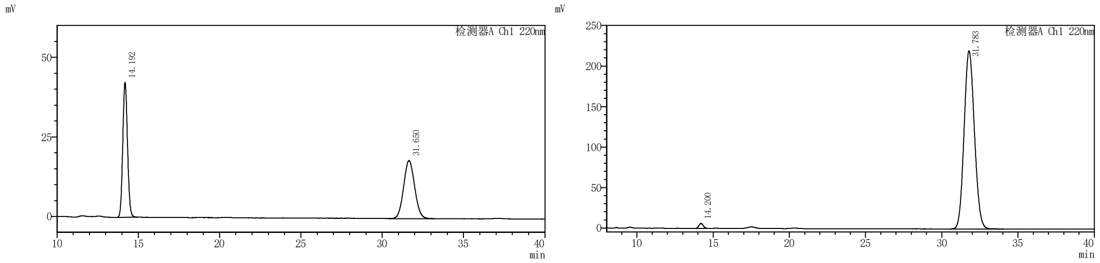


| No. | Time | Area | Area (%) | No. | Time | Area | Area (%) |
|---|---|---|---|---|---|---|---|
| 1 | 14.192 | 860384 | 50.039 | 1 | 15.183 | 8258 | 0.080 |
| 2 | 31.650 | 859036 | 49.961 | 2 | 31.783 | 10353508 | 99.920 |

**Supplementary Figure 55. HPLC data of 54.**

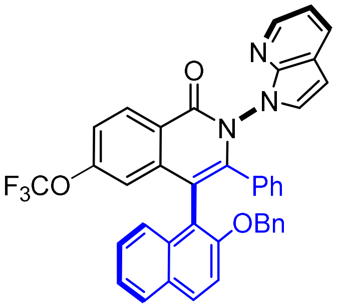


**(*R*,*S*)-4-(2-(benzyloxy)naphthalen-1-yl)-3-phenyl-2-(1H-pyrrolo[2,3-b]pyridin-1-yl)-6-(trifluoromethoxy)isoquinolin-1(2H)-one (*dia*-54)**

White solid (39.2 mg, 60%). $^{1}$H NMR (600 MHz, $CDCl_3$) δ 8.53 (d, *J* = 8.9 Hz, 1H), 8.28 (dd, *J* = 4.9, 1.5 Hz, 1H), 7.73 (d, *J* = 8.5 Hz, 1H), 7.71 – 7.67 (m, 1H), 7.65 (d, *J* = 9.1 Hz, 1H), 7.62 – 7.58 (m, 1H), 7.33 (ddd, *J* = 8.4, 6.8, 1.3 Hz, 1H), 7.27 (ddt, *J* = 10.5, 2.8, 1.3 Hz, 1H), 7.17-7.26 (m, 4H), 7.14 – 7.09 (m, 4H), 7.01 – 6.95 (m, 2H), 6.83

(d, *J* = 7.9 Hz, 1H), 6.79 – 6.74 (m, 1H), 6.69 – 6.64 (m, 2H), 6.37 – 6.31 (m, 1H), 6.25 (dd, *J* = 6.2, 3.8 Hz, 1H), 5.12 – 5.04 (m, 2H).

$^{13}$C NMR (151 MHz, $CDCl_3$) δ 160.78, 154.31, 153.08, 144.63, 139.81, 137.21, 133.72, 131.99, 131.42, 130.49, 128.84, 128.69, 128.58, 128.45, 128.23, 127.99, 127.93, 127.90, 127.13, 126.61, 126.52, 125.00, 123.93, 123.87, 119.34, 119.30, 119.22, 118.73, 117.74, 117.21, 116.60, 113.87, 112.50, 100.65, 70.58.

**HRMS** (ESI-TOF) (m/z): Calcd for $C_{40}H_{27}F_3N_3O_3^+$, ([M + H]$^+$), 654.1999, found 654.1994. **[α]$_D^{20}$** = +43 (c = 0.1, $CHCl_3$).

**HPLC** conditions: Daicel Chiralpak OD-3 column (80: 20 hexane: 2-propanol, 0.8 mL/min, 40 ºC, 254 nm); tr (major) = 34.8 min, tr (minor) = 12.9 min, 95% e.e.. dr =1:7 was determined by crude NMR.

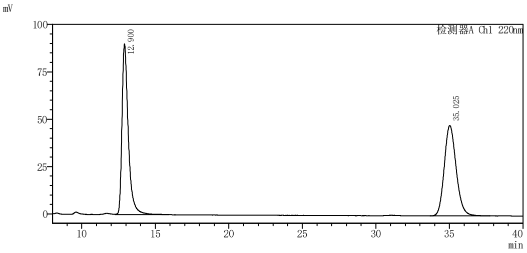


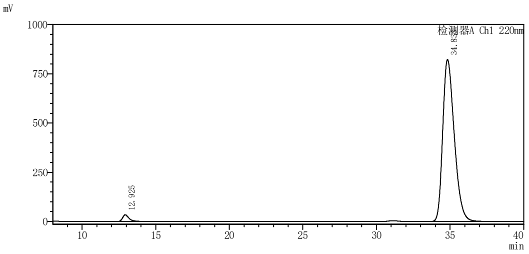


| No. | Time | Area | Area (%) | No. | Time | Area | Area (%) |
|---|---|---|---|---|---|---|---|
| 1 | 12.900 | 2687093 | 50.076 | 1 | 12.925 | 1043492 | 2.264 |
| 2 | 35.025 | 2678925 | 49.924 | 2 | 34.833 | 45055004 | 97.736 |

**Supplementary Figure 56. HPLC data of dia-54.**

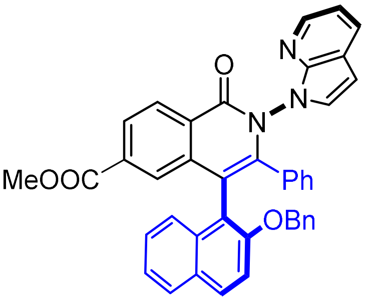


**(*S*,*S*)-methyl 4-(2-(benzyloxy)naphthalen-1-yl)-1-oxo-3-phenyl-2-(1H-pyrrolo[2,3-b]pyridin-1-yl)-1,2-dihydroisoquinoline-6-carboxylate (55)**

White solid (27.0 mg, 43%) $^1$H NMR (600 MHz, $CDCl_3$) δ 8.63 (dd, *J* = 8.4, 1.9 Hz, 1H), 8.36 (dq, *J* = 3.5, 1.7 Hz, 1H), 8.10 (dt, *J* = 8.4, 1.8 Hz, 1H), 7.78 (dt, *J* = 7.9, 1.8 Hz, 1H), 7.70 (ddd, *J* = 16.1, 7.8, 1.9 Hz, 2H), 7.63 (q, *J* = 1.9 Hz, 1H), 7.57 (d, *J* = 8.5 Hz, 1H), 7.39 (ddd, *J* = 8.4, 6.9, 1.6 Hz, 1H), 7.32 – 7.26 (m, 3H), 7.24 – 7.19 (m, 3H), 7.18 – 7.15 (m, 1H), 7.14 (dd, *J* = 3.9, 1.8 Hz, 1H), 7.11 – 7.05 (m, 2H), 6.87 (dd, *J* = 7.7, 1.9 Hz, 1H), 6.76 (td, *J* = 7.5, 3.8 Hz, 1H), 6.64 (dt, *J* = 9.3, 4.7 Hz, 1H), 6.61 – 6.55 (m, 1H), 6.35 (dt, *J* = 3.9, 1.9 Hz, 1H), 5.14 – 4.99 (m, 2H), 3.78 (s, 3H).

$^{13}$C NMR (151 MHz, $CDCl_3$) δ 166.28, 161.10, 154.26, 146.53, 143.95, 143.90, 137.72, 137.15, 134.46, 134.30, 131.97, 130.34, 129.62, 129.15, 128.77, 128.49, 128.38, 128.35, 128.25, 127.88, 127.45, 127.43, 127.26, 126.92, 126.57, 126.52, 126.34, 124.55, 123.60, 118.65, 117.49, 117.19, 114.26, 113.24, 100.74, 70.17, 52.39.

**HRMS** (ESI-TOF) (m/z): Calcd for $C_{41}H_{30}N_3O_4^+$, ([M + H]$^+$), 628.2231, found 628.2226. **[α]$_D^{20}$** = -22 (c = 0.1, $CHCl_3$).

**HPLC** conditions: Daicel Chiralpak OD-3 column (80: 20 hexane: 2-propanol, 0.8 mL/min, 40 ºC, 254 nm); tr (major) = 18.9 min, tr (minor) = 11.9 min, 97% e.e.. >20:1 dr was determined by crude NMR.

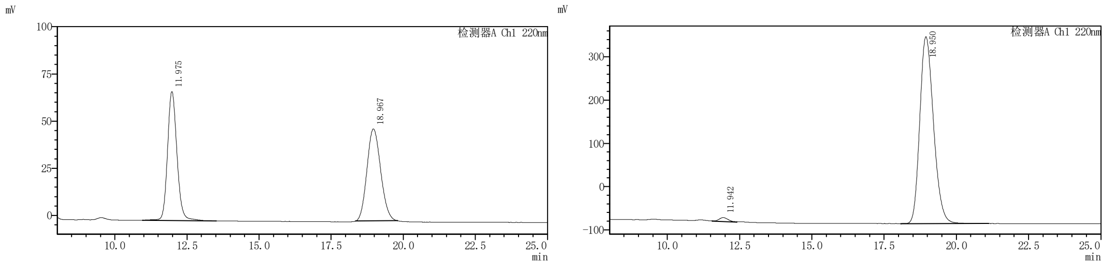

| No. | Time | Area | Area (%) | No. | Time | Area | Area (%) |
|---|---|---|---|---|---|---|---|
| 1 | 11.975 | 1565563 | 49.258 | 1 | 11.942 | 209083 | 1.439 |
| 2 | 18.967 | 1612752 | 50.742 | 2 | 18.950 | 14317331 | 98.561 |

**Supplementary Figure 57. HPLC data of 55.**

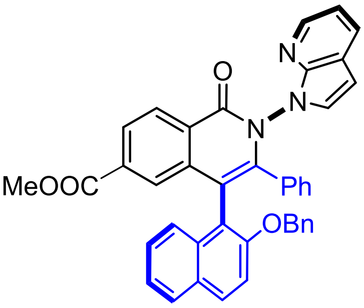


**(*R,S*)-methyl 4-(2-(benzyloxy)naphthalen-1-yl)-1-oxo-3-phenyl-2-(1H-pyrrolo[2,3-b]pyridin-1-yl)-1,2-dihydroisoquinoline-6-carboxylate (*dia*-55)**

White solid (40.8 mg, 65%) $^1$H NMR (600 MHz, $CDCl_3$) δ 8.64 (dd, *J* = 8.4, 1.3 Hz, 1H), 8.36 (dd, *J* = 4.8, 1.5 Hz, 1H), 8.12 (dt, *J* = 8.3, 1.6 Hz, 1H), 7.80 – 7.75 (m, 2H), 7.73 (d, *J* = 9.0 Hz, 1H), 7.69 – 7.62 (m, 2H), 7.40 – 7.35 (m, 1H), 7.29 – 7.24 (m, 4H), 7.22 (dd, *J* = 9.0, 1.3 Hz, 1H), 7.12-7.20 (m, 3H), 7.07 (ddd, *J* = 7.8, 4.8, 1.3 Hz, 1H), 7.03 (dd, *J* = 3.9, 1.3 Hz, 1H), 6.88 (d, *J* = 7.8 Hz, 1H), 6.82 (t, *J* = 7.6 Hz, 1H), 6.73 (td, *J* = 7.5, 1.4 Hz, 1H), 6.39 (t, *J* = 7.6 Hz, 1H), 6.32 (dd, *J* = 3.8, 1.3 Hz, 1H), 5.17 (d, *J* = 2.2 Hz, 2H), 3.78 (s, 3H).

$^{13}$C NMR (151 MHz, $CDCl_3$) δ 166.28, 161.08, 154.33, 146.77, 144.13, 143.83, 137.70, 137.23, 134.35, 133.84, 131.99, 130.38, 129.39, 129.09, 128.88, 128.52, 128.45, 128.41, 128.33, 128.29, 127.97, 127.90, 127.77, 127.35, 127.21, 126.93, 126.54, 126.49, 125.02, 123.77, 118.58, 117.94, 117.17, 113.88, 113.17, 100.58, 70.46, 52.37.

**HRMS** (ESI-TOF) (m/z): Calcd for $C_{41}H_{30}N_3O_4^+$, ([M + H]$^+$), 628.2231, found 628.2227. **$[\alpha]_D^{20}$** = +63 (c = 0.1, $CHCl_3$).

**HPLC** conditions: Daicel Chiralpak OD-3 column (80: 20 hexane: 2-propanol, 0.8 mL/min, 40 ºC, 254 nm); tr (major) = 12.6 min, tr (minor) = 19.0 min, 98% e.e.. < 1:20 dr determined by crude NMR.

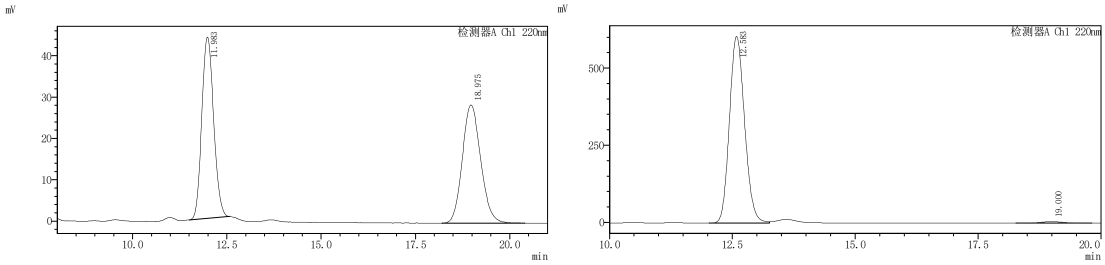

| No. | Time | Area | Area (%) | No. | Time | Area | Area (%) |
|---|---|---|---|---|---|---|---|
| 1 | 11.983 | 958138 | 49.721 | 1 | 12.583 | 12978064 | 98.788 |
| 2 | 18.975 | 968872 | 50.279 | 2 | 19.000 | 159229 | 1.212 |

**Supplementary Figure 58. HPLC data of dia-55.**

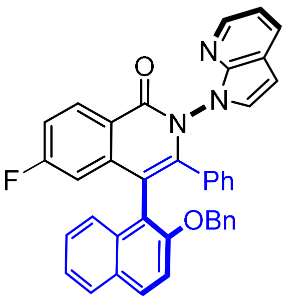


**(*S,S*)-4-(2-(benzyloxy)naphthalen-1-yl)-6-fluoro-3-phenyl-2-(1H-pyrrolo[2,3-b]pyridin-1-yl)isoquinolin-1(2H)-one (56)**

White solid (39.9 mg, 68%) $^1$H NMR (600 MHz, $CDCl_3$) δ 8.58 (dd, $J$ = 8.8, 5.7 Hz, 1H), 8.34 (dd, $J$ = 4.8, 1.5 Hz, 1H), 7.79 – 7.69 (m, 3H), 7.62 (d, $J$ = 8.5 Hz, 1H), 7.47 – 7.41 (m, 1H), 7.37 – 7.30 (m, 5H), 7.28 (td, $J$ = 7.0, 1.7 Hz, 1H), 7.23 – 7.17 (m, 2H), 7.14 – 7.10 (m, 2H), 7.06 (dd, $J$ = 7.8, 4.8 Hz, 1H), 6.89 (dt, $J$ = 7.8, 1.5 Hz, 1H), 6.79 (td, $J$ = 7.5, 1.4 Hz, 1H), 6.66 (t, $J$ = 7.6 Hz, 1H), 6.60 (t, $J$ = 7.7 Hz, 1H), 6.53 (dd, $J$ = 10.1, 2.5 Hz, 1H), 6.34 (d, $J$ = 3.8 Hz, 1H), 5.12 (d, $J$ = 2.3 Hz, 2H).

$^{13}$C NMR (151 MHz, $CDCl_3$) δ 166.91 (d, $J$ = 252.3 Hz), 160.86, 154.11, 146.97, 144.55, 144.26, 140.40, 137.13, 134.15, 132.14, 132.03, 130.27, 129.24, 128.74, 128.57, 128.42, 128.36, 128.25, 127.85, 127.55, 127.04, 126.58, 126.52, 124.61, 123.71, 122.08, 118.43, 117.82, 117.12, 115.86, 115.70, 114.40, 112.33, 111.11, 100.61, 70.26.

**HRMS** (ESI-TOF) (m/z): Calcd for $C_{39}H_{27}FN_3O_2^+$, ([M + H]$^+$), 588.2082, found 588.2072. **[α]$_D^{20}$** = -55 (c = 0.1, $CHCl_3$).

**HPLC** conditions: Daicel Chiralpak IC column (80: 20 hexane: 2-propanol, 0.8 mL/min, 40 °C, 254 nm); tr (major) = 14.9 min, tr (minor) = 24.1 min, 91% e.e.. >20:1 dr determined by crude NMR.

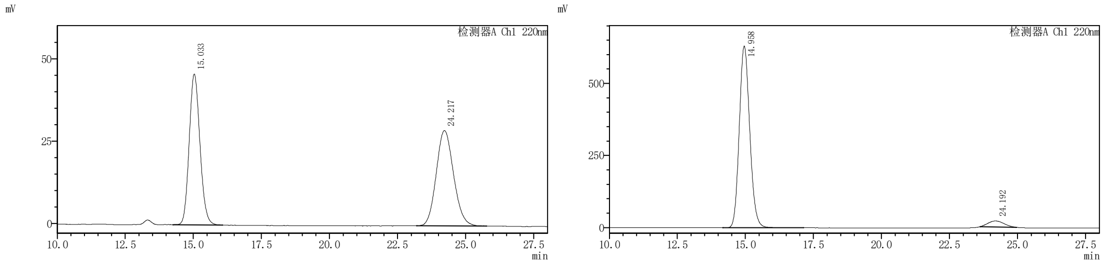


| No. | Time | Area | Area (%) | No. | Time | Area | Area (%) |
|---|---|---|---|---|---|---|---|
| 1 | 15.033 | 1258227 | 49.557 | 1 | 14.958 | 16891736 | 95.253 |
| 2 | 24.217 | 1280739 | 50.443 | 2 | 24.192 | 841869 | 4.747 |

**Supplementary Figure 59. HPLC data of 56.**

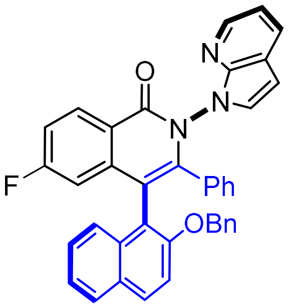


**(*R,S*)-4-(2-(benzyloxy)naphthalen-1-yl)-6-fluoro-3-phenyl-2-(1H-pyrrolo[2,3-b]pyridin-1-yl)isoquinolin-1(2H)-one (*dia*-56)**

White solid (47.6 mg, 81%) $^1$H NMR (600 MHz, $CDCl_3$) δ 8.58 (ddd, $J$ = 8.9, 5.8, 1.2 Hz, 1H), 8.34 (dt, $J$ = 4.9, 1.4 Hz, 1H), 7.79 (d, $J$ = 8.5 Hz, 1H), 7.76 – 7.70 (m, 2H), 7.67 (d, $J$ = 8.2 Hz, 1H), 7.44 – 7.36 (m, 1H), 7.32 – 7.26 (m, 4H), 7.25 – 7.16 (m, 5H), 7.07 – 7.01 (m, 2H), 6.92 – 6.86 (m, 1H), 6.83 (t, $J$ = 7.6 Hz, 1H), 6.74 (tdd, $J$ = 7.5, 6.3, 1.3 Hz, 1H), 6.53 (dt, $J$ = 10.2, 1.8 Hz, 1H), 6.40 (t, $J$ = 7.6 Hz, 1H), 6.35 – 6.29 (m, 1H), 5.16 (s, 2H).

$^{13}$C NMR (151 MHz, $CDCl_3$) δ 166.91 (d, $J$ = 254.8 Hz), 160.90, 154.16, 146.77, 144.37, 144.07, 140.45, 137.17, 133.75, 132.15, 132.06, 130.29, 129.33, 128.82, 128.65, 128.55, 128.33, 128.20, 127.97, 127.91, 127.88, 127.05, 126.62, 126.51, 125.00, 123.86, 122.17, 118.55, 118.11, 117.12, 115.79, 115.63, 113.93, 112.41, 112.39, 110.98, 110.83, 100.46, 70.47.

**HRMS** (ESI-TOF) (m/z): Calcd for $C_{39}H_{27}FN_3O_2^+$, ([M + H]$^+$), 588.2082, found 588.2082. **[α]$_D^{20}$** = +50 (c = 0.1, $CHCl_3$).

**HPLC** conditions: Daicel Chiralpak IC column (80: 20 hexane: 2-propanol, 0.8 mL/min, 40 °C, 254 nm); tr (major) = 11.9 min, tr (minor) = 18.2 min, 92% e.e.. dr =1:10 determined by crude NMR.

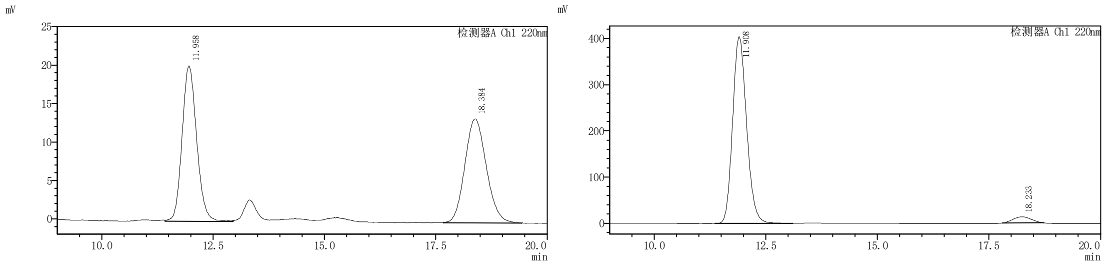


| No. | Time | Area | Area (%) | No. | Time | Area | Area (%) |
|---|---|---|---|---|---|---|---|
| 1 | 11.958 | 462897 | 50.455 | 1 | 11.908 | 8788231 | 95.868 |
| 2 | 18.384 | 454555 | 49.545 | 2 | 18.233 | 378793 | 4.132 |

**Supplementary Figure 60. HPLC data of dia-56.**

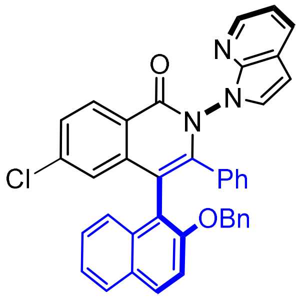


**(*S,S*)-4-(2-(benzyloxy)naphthalen-1-yl)-6-chloro-3-phenyl-2-(1H-pyrrolo[2,3-b]pyridin-1-yl)isoquinolin-1(2H)-one (57)**

White solid (36.8 mg, 61%), $^1H$ NMR (600 MHz, $CDCl_3$) δ 8.42 (d, $J$ = 8.6 Hz, 1H), 8.30 – 8.23 (m, 1H), 7.71 (dd, $J$ = 7.8, 1.4 Hz, 1H), 7.67 – 7.60 (m, 2H), 7.51 (dd, $J$ = 8.5, 1.1 Hz, 1H), 7.41 – 7.32 (m, 2H), 7.28 – 7.17 (m, 6H), 7.14 (dt, $J$ = 7.8, 1.6 Hz, 1H), 7.04 (dd, $J$ = 6.5, 2.6 Hz, 2H), 7.00 (dd, $J$ = 7.8, 4.8 Hz, 1H), 6.79 (q, $J$ = 2.8 Hz, 2H), 6.70 (tt, $J$ = 7.5, 1.3 Hz, 1H), 6.57 (td, $J$ = 7.6, 1.3 Hz, 1H), 6.52 (td, $J$ = 7.6, 1.3 Hz, 1H), 6.27 (d, $J$ = 3.8 Hz, 1H), 5.06 (s, 2H).

$^{13}C$ NMR (151 MHz, $CDCl_3$) δ 159.93, 153.12, 143.44, 142.69, 139.12, 138.05, 136.12, 133.16, 131.03, 129.50, 129.26, 128.68, 127.58, 127.55, 127.38, 127.33, 127.21, 126.80, 126.77, 126.52, 126.00, 125.59, 125.56, 125.51, 124.02, 123.53, 122.85, 122.66, 117.69, 116.57, 116.09, 113.36, 111.18, 99.73, 69.20.

**HRMS** (ESI-TOF) (m/z): Calcd for $C_{39}H_{27}ClN_3O_2^+$, ([M + H]$^+$), 604.1786, found 604.1771. **[α]$_D^{20}$** = -37 (c = 0.1, $CHCl_3$).

**HPLC** conditions: Daicel Chiralpak IC column (80: 20 hexane: 2-propanol, 0.8 mL/min, 40 °C, 254 nm); tr (major) = 15.6 min, tr (minor) = 26.3 min, 96% e.e.. >20:1 dr determined by crude NMR.

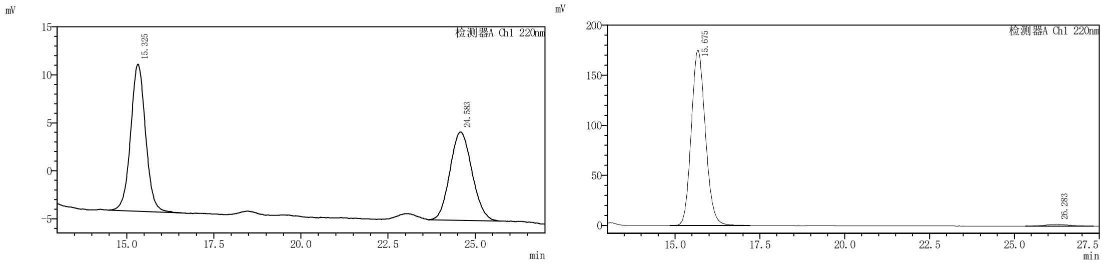


| No. | Time | Area | Area (%) | No. | Time | Area | Area (%) |
|---|---|---|---|---|---|---|---|
| 1 | 15.325 | 454264 | 51.949 | 1 | 15.675 | 5061786 | 98.353 |
| 2 | 24.583 | 420183 | 48.051 | 2 | 26.283 | 84738 | 1.647 |

**Supplementary Figure 61. HPLC data of 57.**

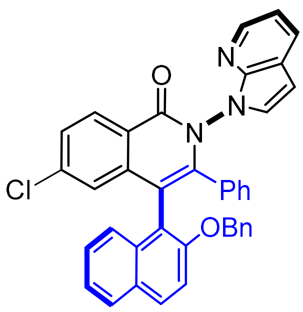

**(*R,S*)-4-(2-(benzyloxy)naphthalen-1-yl)-6-chloro-3-phenyl-2-(1H-pyrrolo[2,3-b]pyridin-1-yl)isoquinolin-1(2H)-one (*dia*-57)**

White solid (39.2 mg, 65%), $^{1}$H NMR (600 MHz, $CDCl_3$) δ 8.50 (d, *J* = 8.6 Hz, 1H), 8.35 (dd, *J* = 4.8, 1.4 Hz, 1H), 7.81 – 7.70 (m, 3H), 7.68 (d, *J* = 8.1 Hz, 1H), 7.46 (dd, *J* = 8.6, 2.0 Hz, 1H), 7.40 (ddd, *J* = 8.3, 6.8, 1.2 Hz, 1H), 7.35 – 7.26 (m, 4H), 7.23 – 7.14 (m, 4H), 7.08 – 7.02 (m, 2H), 6.93 – 6.85 (m, 2H), 6.82 (t, *J* = 7.6 Hz, 1H), 6.77 – 6.70 (m, 1H), 6.39 (t, *J* = 7.6 Hz, 1H), 6.31 (d, *J* = 3.8 Hz, 1H), 5.18 (s, 2H).

$^{13}$C NMR (151 MHz, $CDCl_3$) δ 161.03, 154.18, 146.78, 144.42, 144.12, 140.08, 139.13, 137.17, 133.77, 132.01, 130.47, 130.33, 129.34, 128.80, 128.61, 128.55, 128.34, 128.19, 127.94, 127.89, 127.72, 127.05, 126.62, 126.50, 124.99, 124.93, 123.98, 123.85, 118.55, 117.85, 117.15, 113.83, 112.15, 100.51, 70.40.

**HRMS** (ESI-TOF) (m/z): Calcd for $C_{39}H_{27}ClN_3O_2^+$, ([M + H]$^+$), 604.1786, found 604.1783. **$[\alpha]_D^{20}$** = +18 (c = 0.1, $CHCl_3$).

**HPLC** conditions: Daicel Chiralpak IC column (80: 20 hexane: 2-propanol, 0.8 mL/min, 40 °C, 254 nm); tr (major) = 13.2 min, tr (minor) = 19.5 min, 95% e.e.. dr = 1:17 determined by crude NMR.

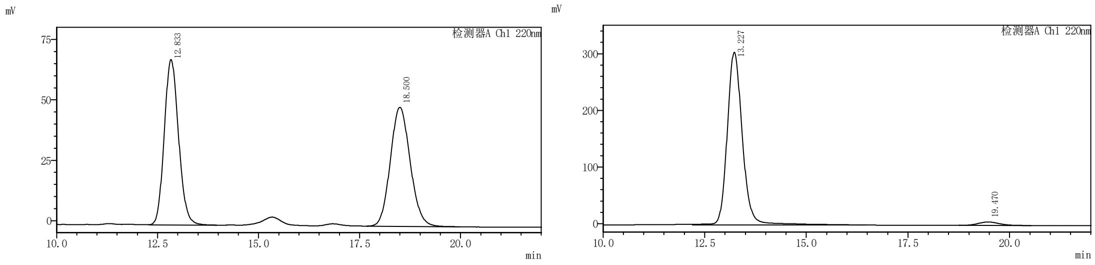


| No. | Time | Area | Area (%) | No. | Time | Area | Area (%) |
|---|---|---|---|---|---|---|---|
| 1 | 12.833 | 1689075 | 49.952 | 1 | 6.658 | 7998434 | 97.344 |
| 2 | 18.500 | 1692297 | 50.048 | 2 | 19.470 | 218249 | 2.656 |

**Supplementary Figure 62. HPLC data of dia-57.**

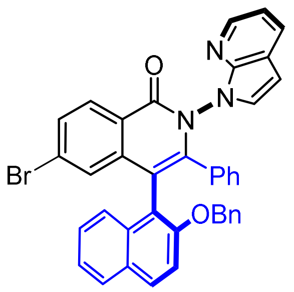

**(*S,S*)-4-(2-(benzyloxy)naphthalen-1-yl)-6-bromo-3-phenyl-2-(1H-pyrrolo[2,3-b]pyridin-1-yl)isoquinolin-1(2H)-one (58)**

White solid (34.9 mg, 54%), $^{1}$H NMR (600 MHz, $CDCl_3$) δ 8.33 (d, *J* = 8.5 Hz, 1H), 8.26 (dd, *J* = 4.8, 1.5 Hz, 1H), 7.70 (dd, *J* = 7.8, 1.5 Hz, 1H), 7.67 – 7.60 (m, 2H), 7.52 (ddd, *J* = 9.9, 8.5, 1.5 Hz, 2H), 7.35 (ddd, *J* = 8.4, 6.8, 1.3 Hz, 1H), 7.31 – 7.21 (m, 5H), 7.22 – 7.18 (m, 1H), 7.14 (dt, *J* = 7.7, 1.5 Hz, 1H), 7.06 – 7.02 (m, 2H), 7.02 – 6.95 (m, 2H), 6.79 (dt, *J* = 7.7, 1.6 Hz, 1H), 6.69 (tt, *J* = 7.5, 1.3 Hz, 1H), 6.56 (td, *J* = 7.6, 1.3 Hz, 1H), 6.51 (td, *J* = 7.7, 1.4 Hz, 1H), 6.27 (d, *J* = 3.9 Hz, 1H), 5.06 (s, 2H).

$^{13}$C NMR (151 MHz, $CDCl_3$) δ 160.06, 153.12, 143.45, 142.73, 138.16, 136.13, 133.16, 131.00, 129.58, 129.49, 129.28, 128.64, 127.97, 127.68, 127.58, 127.56, 127.37, 127.33, 127.21, 127.08, 126.79, 126.53, 126.01, 125.54, 125.49, 123.53, 123.22, 122.66, 117.66, 116.50, 116.10, 113.31, 111.08, 99.72, 69.18.

**HRMS** (ESI-TOF) (m/z): Calcd for $C_{39}H_{27}BrN_3O_2^+$, ([M + H]$^+$), 648.1281, found 648.1270. **$[\alpha]_D^{20}$** = -24 (c = 0.1, $CHCl_3$).

**HPLC** conditions: Daicel Chiralpak IC column (80: 20 hexane: 2-propanol, 0.8 mL/min, 40 °C, 254 nm); tr (major) = 16.8 min, tr (minor) = 27.7 min, 96% e.e.. >20:1 dr determined by crude NMR.

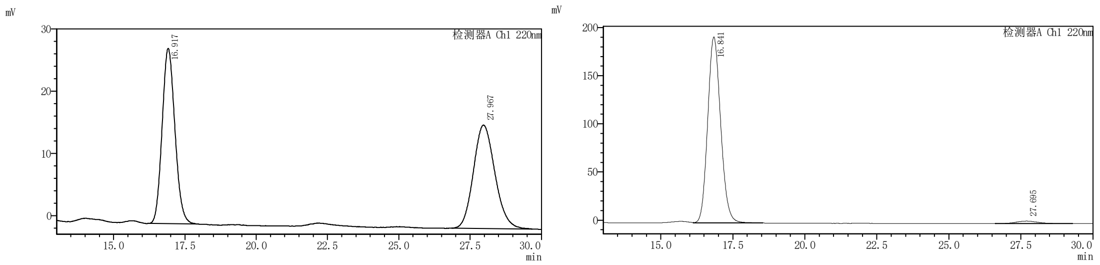


| No. | Time | Area | Area (%) | No. | Time | Area | Area (%) |
|---|---|---|---|---|---|---|---|
| 1 | 16.917 | 880727 | 50.255 | 1 | 16.841 | 6063456 | 97.787 |
| 2 | 27.967 | 871780 | 49.745 | 2 | 27.695 | 137250 | 2.213 |

**Supplementary Figure 63. HPLC data of 58.**

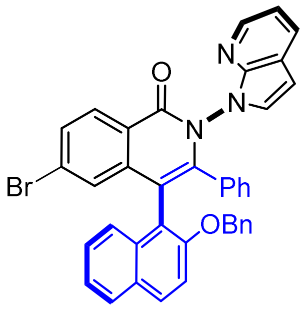


**(*R,S*)-4-(2-(benzyloxy)naphthalen-1-yl)-6-bromo-3-phenyl-2-(1H-pyrrolo[2,3-b]pyridin-1-yl)isoquinolin-1(2H)-one (*dia*-58)**

White solid (42.7 mg, 66%), $^{1}$H NMR (600 MHz, $CDCl_3$) δ 8.42 (d, *J* = 8.6 Hz, 1H), 8.35 (dd, *J* = 4.8, 1.5 Hz, 1H), 7.77 (ddd, *J* = 13.6, 8.1, 1.3 Hz, 2H), 7.73 (d, *J* = 9.0 Hz, 1H), 7.68 (d, *J* = 8.2 Hz, 1H), 7.62 (dd, *J* = 8.6, 1.9 Hz, 1H), 7.41 (ddd, *J* = 8.4, 6.7, 1.3 Hz, 1H), 7.35 – 7.26 (m, 4H), 7.20 (d, *J* = 8.7 Hz, 3H), 7.15 (dd, *J* = 7.9, 1.7 Hz, 1H), 7.09 (d, *J* = 1.9 Hz, 1H), 7.06 (dd, *J* = 7.8, 4.8 Hz, 1H), 7.03 (d, *J* = 3.8 Hz, 1H), 6.87 (dt, *J* = 7.8, 1.5 Hz, 1H), 6.84 – 6.79 (m, 1H), 6.74 (tt, *J* = 7.5, 1.3 Hz, 1H), 6.42 – 6.36 (m, 1H), 6.31 (d, *J* = 3.8 Hz, 1H), 5.19 (s, 2H).

$^{13}$C NMR (151 MHz, $CDCl_3$) δ 161.15, 154.21, 146.81, 144.43, 144.15, 139.24, 137.19, 133.77, 132.01, 130.54, 130.48, 130.35, 129.33, 128.90, 128.80, 128.64, 128.51, 128.35, 128.19, 128.01, 127.95, 127.89, 127.05, 126.60, 126.50, 124.99, 124.36, 123.84, 118.55, 117.80, 117.16, 113.81, 112.05, 100.52, 70.39.

**HRMS** (ESI-TOF) (m/z): Calcd for $C_{39}H_{27}BrN_3O_2^+$, ([M + H]$^+$), 648.1281, found 648.1267. **$[\alpha]_D^{20}$** = +25 (c = 0.1, $CHCl_3$).

**HPLC** conditions: Daicel Chiralpak IC column (80: 20 hexane: 2-propanol, 0.8 mL/min, 40 °C, 254 nm); tr (major) = 14.3 min, tr (minor) = 20.3 min, 96% e.e.. dr = 1:19 determined by crude NMR.

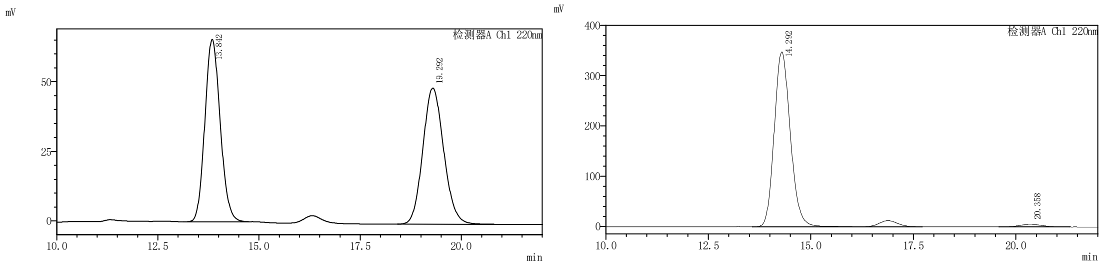


| No. | Time | Area | Area (%) | No. | Time | Area | Area (%) |
|---|---|---|---|---|---|---|---|
| 1 | 13.842 | 1731268 | 49.535 | 1 | 14.292 | 9903996 | 98.084 |
| 2 | 19.292 | 1763801 | 50.465 | 2 | 20.358 | 193460 | 1.916 |

**Supplementary Figure 64. HPLC data of dia-58.**

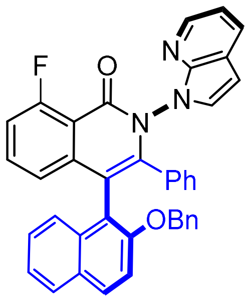

**(*S,S*)-4-(2-(benzyloxy)naphthalen-1-yl)-8-fluoro-3-phenyl-2-(1H-pyrrolo[2,3-b]pyridin-1-yl)isoquinolin-1(2H)-one (59)**

White solid (25.8 mg, 44%), $^{1}$H NMR (600 MHz, $CDCl_3$) δ 8.26 (d, *J* = 4.7 Hz, 1H), 7.70 (d, *J* = 7.7 Hz, 1H), 7.64 (dd, *J* = 18.4, 8.6 Hz, 2H), 7.53 (d, *J* = 8.5 Hz, 1H), 7.38 – 7.29 (m, 2H), 7.28 – 7.16 (m, 7H), 7.10 – 7.01 (m, 3H), 7.01 – 6.97 (m, 1H), 6.84 – 6.77 (m, 1H), 6.73 – 6.68 (m, 1H), 6.62 (d, *J* = 8.2 Hz, 1H), 6.58 (t, *J* = 7.6 Hz, 1H), 6.54 (t, *J* = 7.7 Hz, 1H), 6.28 (dd, *J* = 3.9, 1.3 Hz, 1H), 5.04 (s, 2H).

$^{13}$C NMR (151 MHz, $CDCl_3$) δ 162.11 (d, *J* = 264.4 Hz), 158.41, 154.18, 146.71, 144.36, 143.83, 140.17, 137.23, 134.27, 132.20, 130.12, 129.50, 128.78, 128.57, 128.39, 128.32, 128.19, 127.79, 127.53, 126.98, 126.64, 126.56, 124.70, 123.67, 121.54, 118.75, 118.36, 117.09, 114.66, 114.46, 114.28, 112.12, 100.76, 70.29.

**HRMS** (ESI-TOF) (m/z): Calcd for $C_{39}H_{27}FN_3O_2^+$, ([M + H]$^+$), 588.2082, found 588.2077. **[α]$_D^{20}$** = -48 (c = 0.1, $CHCl_3$).

**HPLC** conditions: Daicel Chiralpak OD-3 column (80: 20 hexane: 2-propanol, 0.8 mL/min, 40 °C, 254 nm); tr (major) = 10.4 min, tr (minor) = 11.5 min, 99% e.e.. >20:1 dr determined by crude NMR.

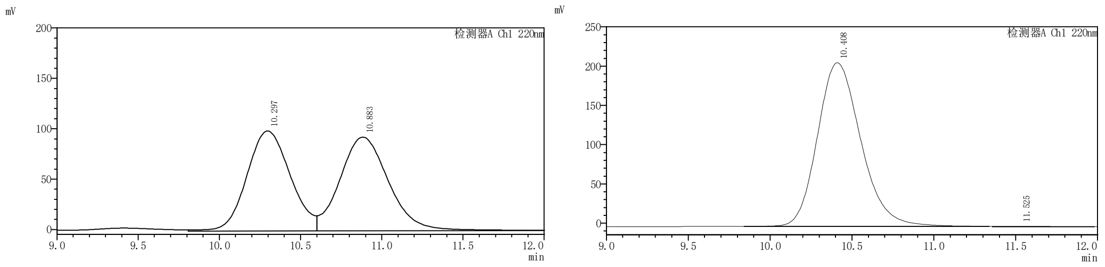


| No. | Time | Area | Area (%) | No. | Time | Area | Area (%) |
|---|---|---|---|---|---|---|---|
| 1 | 10.297 | 1852473 | 48.783 | 1 | 10.408 | 3847782 | 99.702 |
| 2 | 10.883 | 1944871 | 51.217 | 2 | 11.525 | 11482 | 0.298 |

**Supplementary Figure 65. HPLC data of 59.**

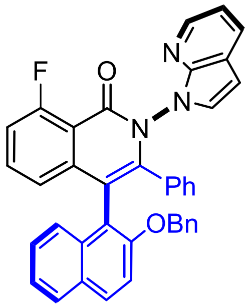

**(*R,S*)-4-(2-(benzyloxy)naphthalen-1-yl)-8-fluoro-3-phenyl-2-(1H-pyrrolo[2,3-b]pyridin-1-yl)isoquinolin-1(2H)-one (*dia*-59)**

White solid (42.3 mg, 72%), $^{1}$H NMR (600 MHz, $CDCl_3$) δ 8.26 (dd, *J* = 4.8, 1.5 Hz, 1H), 7.78 (d, *J* = 8.5 Hz, 1H), 7.68 (dd, *J* = 7.8, 1.5 Hz, 1H), 7.65 (d, *J* = 9.0 Hz, 1H), 7.61 (d, *J* = 8.1 Hz, 1H), 7.37 – 7.29 (m, 2H), 7.26 – 7.19 (m, 4H), 7.14 – 7.04 (m, 5H), 6.97 (dd, *J* = 6.7, 4.2 Hz, 2H), 6.85 (d, *J* = 7.9 Hz, 1H), 6.75 (t, *J* = 7.4 Hz, 1H), 6.66 (tt, *J* = 7.5, 1.3 Hz, 1H), 6.63 (dd, *J* = 8.3, 1.0 Hz, 1H), 6.33 (t, *J* = 7.7 Hz, 1H), 6.25 (d, *J* = 3.8 Hz, 1H), 5.07 (s, 2H).

$^{13}$C NMR (151 MHz, $CDCl_3$) δ 162.83 (d, *J* = 264.1 Hz), 161.07, 157.47, 153.14, 143.19, 139.22, 136.21, 133.21, 133.14, 132.90, 131.08, 129.14, 127.97, 127.77, 127.54, 127.27, 127.08, 126.98, 126.84, 126.80, 126.03, 125.55, 125.52, 125.50, 125.48, 124.22, 122.83, 120.43, 120.40, 117.65, 116.06, 113.71, 113.68, 113.19, 113.05, 112.91, 111.16, 99.52, 69.43.

**HRMS** (ESI-TOF) (m/z): Calcd for $C_{39}H_{27}FN_3O_2^+$, ([M + H]$^+$), 588.2082, found 588.2066. **[α]$_D^{20}$** = +37 (c = 0.1, $CHCl_3$).

**HPLC** conditions: Daicel Chiralpak OD-3 column (80: 20 hexane: 2-propanol, 0.8 mL/min, 40 °C, 254 nm); tr (major) = 9.0 min, tr (minor) = 4.7 min, 94% e.e.. <1:20 dr determined by crude NMR.

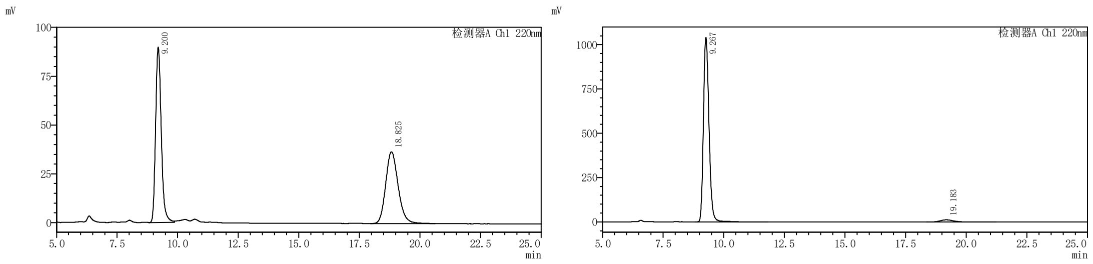


| No. | Time | Area | Area (%) | No. | Time | Area | Area (%) |
|---|---|---|---|---|---|---|---|
| 1 | 9.200 | 1463186 | 52.677 | 1 | 9.267 | 16611071 | 97.177 |
| 2 | 18.825 | 1314493 | 47.323 | 2 | 19.183 | 482477 | 2.823 |

**Supplementary Figure 66. HPLC data of dia-59.**

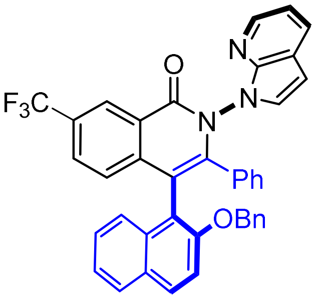


**(*S*,*S*)-4-(2-(benzyloxy)naphthalen-1-yl)-3-phenyl-2-(1H-pyrrolo[2,3-b]pyridin-1-yl)-7-(trifluoromethyl)isoquinolin-1(2H)-one (60)**

White solid (36.3 mg, 57%), $^1$H NMR (600 MHz, $CDCl_3$) δ 8.84 (d, *J* = 2.1 Hz, 1H), 8.33 (dd, *J* = 4.8, 1.5 Hz, 1H), 7.78 – 7.68 (m, 3H), 7.61 (dd, *J* = 8.6, 2.0 Hz, 1H), 7.57 (d, *J* = 8.5 Hz, 1H), 7.42 (ddd, *J* = 8.3, 6.8, 1.3 Hz, 1H), 7.36 – 7.24 (m, 6H), 7.19 (dt, *J* = 7.8, 1.6 Hz, 1H), 7.13 (d, *J* = 9.1 Hz, 1H), 7.08 (d, *J* = 3.8 Hz, 1H), 7.05 (dd, *J* = 7.8, 4.8 Hz, 1H), 6.99 (d, *J* = 8.5 Hz, 1H), 6.88 (dt, *J* = 7.8, 1.6 Hz, 1H), 6.78 (tt, *J* = 7.5, 1.4 Hz, 1H), 6.69 – 6.62 (m, 1H), 6.62 – 6.56 (m, 1H), 6.33 (d, *J* = 3.8 Hz, 1H), 5.13 – 4.99 (m, 2H).

$^{13}$C NMR (151 MHz, $CDCl_3$) δ 160.85, 154.19, 146.95, 145.63, 144.33, 140.18, 137.02, 134.19, 131.91, 130.36, 129.26, 128.74, 128.55, 128.51, 128.30, 128.17, 127.79, 127.59, 127.13, 126.61, 126.56, 126.38, 125.39, 124.53, 123.78, 118.44, 117.68, 117.21, 114.44, 112.34, 100.80, 70.36.

**HRMS** (ESI-TOF) (m/z): Calcd for $C_{40}H_{27}F_3N_3O_2^+$, ([M + H]$^+$), 638.2050, found 638.2041. **$[\alpha]_D^{20}$** = -23 (c = 0.1, $CHCl_3$).

**HPLC** conditions: Daicel Chiralpak IC column (90: 10 hexane: 2-propanol, 0.8 mL/min, 40 °C, 254 nm); tr (major) = 14.7 min, tr (minor) = 12.9 min, 97% e.e.. >20:1 dr determined by crude NMR.

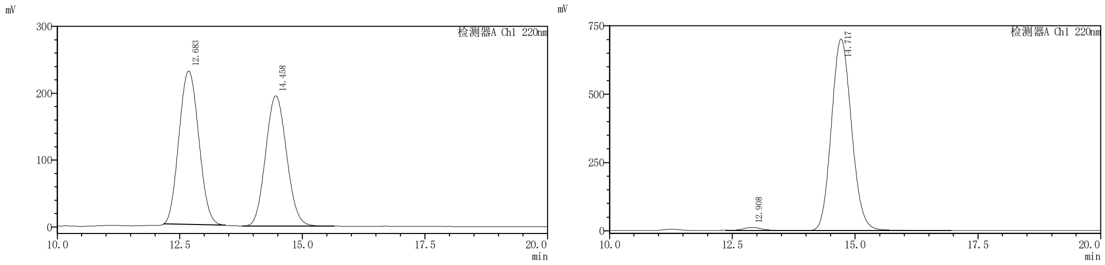


| No. | Time | Area | Area (%) | No. | Time | Area | Area (%) |
|---|---|---|---|---|---|---|---|
| 1 | 12.683 | 6464218 | 52.668 | 1 | 12.908 | 307972 | 1.513 |
| 2 | 14.458 | 5809372 | 47.332 | 2 | 14.717 | 20046794 | 98.487 |

**Supplementary Figure 67. HPLC data of 60.**

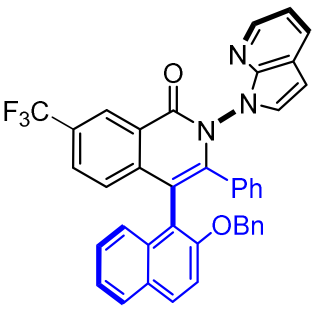

**(*R,S*)-4-(2-(benzyloxy)naphthalen-1-yl)-3-phenyl-2-(1H-pyrrolo[2,3-b]pyridin-1-yl)-7-(trifluoromethyl)isoquinolin-1(2H)-one (*dia*-60)**

White solid (40.1 mg, 63%), $^1$H NMR (600 MHz, $CDCl_3$) δ 8.85 (d, *J* = 2.1 Hz, 1H), 8.32 (dd, *J* = 4.8, 1.7 Hz, 1H), 7.78 – 7.70 (m, 3H), 7.68 (d, *J* = 8.2 Hz, 1H), 7.62 (dd, *J* = 8.5, 2.0 Hz, 1H), 7.40 (ddd, *J* = 8.4, 6.8, 1.4 Hz, 1H), 7.35 – 7.24 (m, 5H), 7.23 – 7.11 (m, 4H), 7.06 – 7.02 (m, 2H), 7.00 (d, *J* = 8.3 Hz, 1H), 6.88 (d, *J* = 7.9 Hz, 1H), 6.84 (t, *J* = 7.6 Hz, 1H), 6.74 (td, *J* = 7.5, 1.5 Hz, 1H), 6.40 (t, *J* = 7.7 Hz, 1H), 6.34 – 6.28 (m, 1H), 5.14 (s, 2H).

$^{13}$C NMR (151 MHz, $CDCl_3$) δ 159.94, 153.19, 145.63, 144.38, 143.07, 139.21, 136.04, 132.78, 130.77, 129.36, 128.40, 128.29, 127.78, 127.51, 127.46, 127.11, 126.93, 126.88, 126.13, 125.68, 125.58, 125.54, 125.50, 125.30, 124.45, 123.90, 122.90, 117.56, 116.88, 116.20, 112.93, 111.47, 99.65, 69.52.

**HRMS** (ESI-TOF) (m/z): Calcd for $C_{40}H_{27}F_3N_3O_2^+$, ([M + H]$^+$), 638.2050, found 638.2045. **[α]$_D$$^{20}$** = +27 (c = 0.1, $CHCl_3$).

**HPLC** conditions: Daicel Chiralpak IC column (90: 10 hexane: 2-propanol, 0.8 mL/min, 40 °C, 254 nm); tr (major) = 11.3 min, tr (minor) = 12.4 min, 93% e.e.. dr = 1:5 determined by crude NMR.

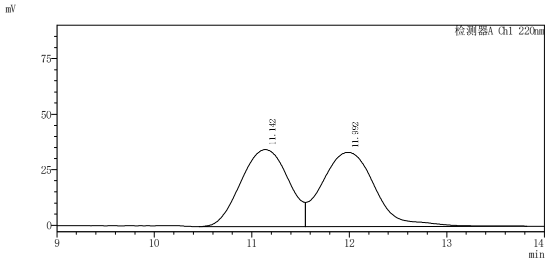


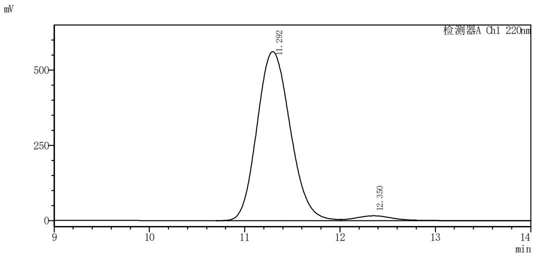


| No. | Time | Area | Area (%) | No. | Time | Area | Area (%) |
|---|---|---|---|---|---|---|---|
| 1 | 11.142 | 1173667 | 48.413 | 1 | 11.292 | 13642053 | 96.682 |
| 2 | 11.992 | 1250634 | 51.587 | 2 | 12.350 | 468233 | 3.318 |

**Supplementary Figure 68. HPLC data of *dia*-60.**

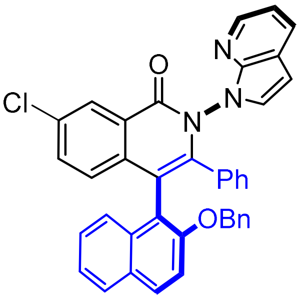

**(*S,S*)-4-(2-(benzyloxy)naphthalen-1-yl)-7-chloro-3-phenyl-2-(1H-pyrrolo[2,3-b]pyridin-1-yl)isoquinolin-1(2H)-one (61)**

White solid (36.8 mg, 61%), $^1$H NMR (600 MHz, $CDCl_3$) δ 8.52 (t, *J* = 1.8 Hz, 1H), 8.32 (dd, *J* = 4.7, 1.8 Hz, 1H), 7.79 – 7.67 (m, 3H), 7.58 (d, *J* = 8.5 Hz, 1H), 7.45 – 7.35 (m, 2H), 7.35 – 7.24 (m, 6H), 7.20 – 7.15 (m, 1H), 7.11 (dd, *J* = 9.1, 1.4 Hz, 1H), 7.08 (dd, *J* = 3.9, 1.4 Hz, 1H), 7.07 – 7.02 (m, 1H), 6.85 (ddd, *J* = 16.7, 8.2, 1.6 Hz, 2H), 6.77 (td, *J* = 7.6, 1.4 Hz, 1H), 6.65 (t, *J* = 7.6 Hz, 1H), 6.58 (t, *J* = 7.7 Hz, 1H), 6.32 (dd, *J* = 3.9, 1.4 Hz, 1H), 5.14 – 5.02 (m, 2H).

$^{13}$C NMR (151 MHz, $CDCl_3$) δ 160.54, 154.14, 146.93, 144.27, 143.57, 137.12, 136.06, 134.26, 133.66, 133.17, 132.10, 130.17, 129.21, 128.71, 128.55, 128.43, 128.30, 128.21, 128.10, 127.95, 127.52, 127.44, 127.01, 126.64, 126.54, 126.50, 124.67, 123.70, 118.41, 118.00, 117.12, 114.45, 112.35, 100.64, 70.28.

**HRMS** (ESI-TOF) (m/z): Calcd for $C_{39}H_{27}ClN_3O_2^+$, ([M + H]$^+$), 604.1786, found 604.1787. **[α]$_D$$^{20}$** = -33 (c = 0.1, $CHCl_3$).

**HPLC** conditions: Daicel Chiralpak IC column (80: 20 hexane: 2-propanol, 0.8 mL/min, 40 °C, 254 nm); tr (major) = 17.6 min, tr (minor) = 15.9 min, 92% e.e.. >20:1 dr determined by crude NMR.

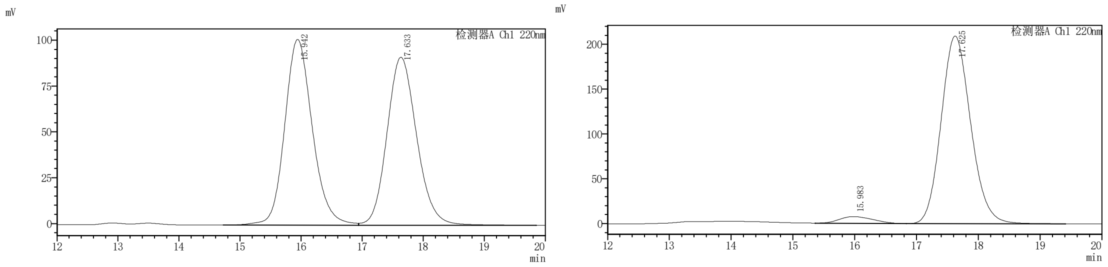


| No. | Time | Area | Area (%) | No. | Time | Area | Area (%) |
|---|---|---|---|---|---|---|---|
| 1 | 15.942 | 3193699 | 50.717 | 1 | 15.983 | 280739 | 3.876 |
| 2 | 17.633 | 3103369 | 49.283 | 2 | 17.625 | 6962001 | 96.124 |

**Supplementary Figure 69. HPLC data of 61.**

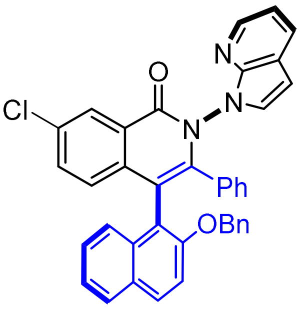

**(*R,S*)-4-(2-(benzyloxy)naphthalen-1-yl)-7-chloro-3-phenyl-2-(1H-pyrrolo[2,3-b]pyridin-1-yl)isoquinolin-1(2H)-one (*dia*-61)**

White solid (42.2 mg, 70%), $^{1}$H NMR (600 MHz, $CDCl_3$) δ 8.53 (d, $J$ = 2.4 Hz, 1H), 8.33 (dd, $J$ = 4.8, 1.5 Hz, 1H), 7.81 – 7.65 (m, 4H), 7.40 (ddd, $J$ = 8.6, 6.5, 1.7 Hz, 2H), 7.32 – 7.26 (m, 4H), 7.23 – 7.14 (m, 4H), 7.08 – 6.99 (m, 2H), 6.79-6.89 (m, 3H), 6.77 – 6.71 (m, 1H), 6.39 (t, $J$ = 7.7 Hz, 1H), 6.31 (d, $J$ = 3.9 Hz, 1H), 5.14 (s, 2H).

$^{13}$C NMR (151 MHz, $CDCl_3$) δ 160.64, 154.18, 146.69, 144.09, 143.40, 137.19, 136.12, 133.86, 133.63, 133.16, 132.00, 130.21, 129.31, 128.78, 128.56, 128.31, 128.28, 128.09, 128.02, 127.88, 127.34, 127.05, 126.73, 126.62, 126.51, 126.50, 125.08, 123.86, 118.53, 118.26, 117.14, 113.97, 112.45, 100.50, 70.49.

**HRMS** (ESI-TOF) (m/z): Calcd for $C_{39}H_{27}ClN_3O_2^+$, ([M + H]$^+$), 604.1786, found 604.1768. **$[\alpha]_D^{20}$** = +42 (c = 0.1, $CHCl_3$).

**HPLC** conditions: Daicel Chiralpak OD-3 column (80: 20 hexane: 2-propanol, 0.8 mL/min, 40 °C, 254 nm); tr (major) = 11.3 min, tr (minor) = 9.5 min, 96% e.e.. dr = 1:9 determined by crude NMR.

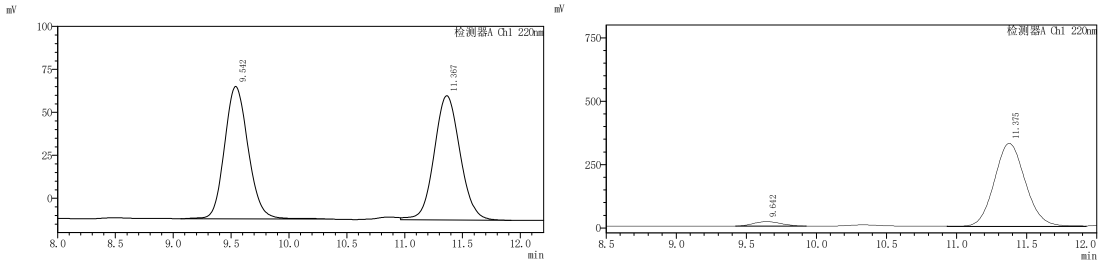


| No. | Time | Area | Area (%) | No. | Time | Area | Area (%) |
|---|---|---|---|---|---|---|---|
| 1 | 9.542 | 1104212 | 49.355 | 1 | 9.642 | 235391 | 2.831 |
| 2 | 11.367 | 1133078 | 50.645 | 2 | 11.375 | 8079374 | 97.169 |

**Supplementary Figure 70. HPLC data of dia-61.**

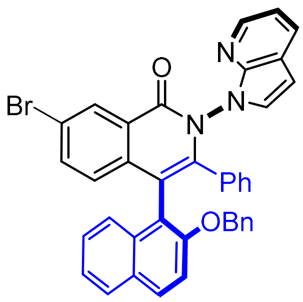

**(*S,S*)-4-(2-(benzyloxy)naphthalen-1-yl)-7-bromo-3-phenyl-2-(1H-pyrrolo[2,3-b]pyridin-1-yl)isoquinolin-1(2H)-one (62)**

White solid (34.3 mg, 53%), $^{1}H$ NMR (600 MHz, $CDCl_3$) δ 8.60 (t, *J* = 1.7 Hz, 1H), 8.24 (dd, *J* = 4.8, 1.5 Hz, 1H), 7.70 – 7.58 (m, 3H), 7.50 (d, *J* = 8.5 Hz, 1H), 7.42 (dd, *J* = 8.8, 2.0 Hz, 1H), 7.37 – 7.30 (m, 1H), 7.28 – 7.13 (m, 7H), 7.10 (dd, *J* = 7.7, 1.9 Hz, 1H), 7.06 – 6.98 (m, 2H), 6.96 (ddd, *J* = 7.8, 4.8, 1.2 Hz, 1H), 6.84 – 6.76 (m, 1H), 6.70 – 6.65 (m, 2H), 6.56 (t, *J* = 7.6 Hz, 1H), 6.49 (t, *J* = 7.7 Hz, 1H), 6.23 (dd, *J* = 3.9, 1.2 Hz, 1H), 4.99 (d, *J* = 3.3 Hz, 2H).

$^{13}C$ NMR (151 MHz, $CDCl_3$) δ 159.38, 153.11, 145.81, 143.14, 142.72, 136.07, 135.38, 135.35, 133.21, 131.06, 130.18, 129.17, 128.27, 127.68, 127.52, 127.35, 127.30, 127.28, 127.19, 126.88, 126.52, 126.51, 125.99, 125.85, 125.52, 123.62, 122.69, 120.06, 117.44, 116.89, 116.11, 113.42, 111.41, 99.64, 69.28.

**HRMS** (ESI-TOF) (m/z): Calcd for $C_{39}H_{27}BrN_3O_2^+$, ([M + H]$^+$), 648.1281, found 648.1285. **$[\alpha]_D^{20}$** = -30 (c = 0.1, $CHCl_3$).

**HPLC** conditions: Daicel Chiralpak IC column (80: 20 hexane: 2-propanol, 0.8 mL/min, 40 °C, 254 nm); tr (major) = 19.0 min, tr (minor) = 16.7 min, 97% e.e.. >20:1 dr determined by crude NMR.

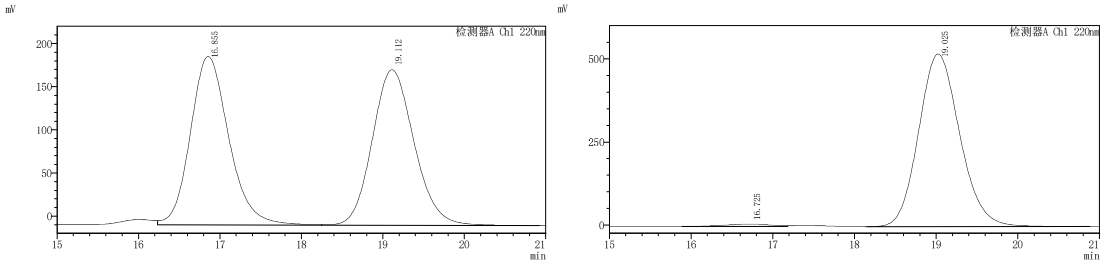


| No. | Time | Area | Area (%) | No. | Time | Area | Area (%) |
|---|---|---|---|---|---|---|---|
| 1 | 16.855 | 6437274 | 50.023 | 1 | 16.725 | 294189 | 1.593 |
| 2 | 19.112 | 6431367 | 49.977 | 2 | 19.025 | 18172935 | 98.407 |

**Supplementary Figure 71. HPLC data of 62.**

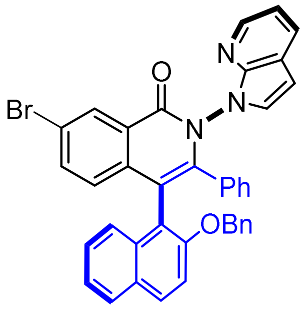

**(*R,S*)-4-(2-(benzyloxy)naphthalen-1-yl)-7-bromo-3-phenyl-2-(1H-pyrrolo[2,3-b]pyridin-1-yl)isoquinolin-1(2H)-one (*dia*-62)**

White solid (49.8 mg, 77%), $^{1}H$ NMR (600 MHz, $CDCl_3$) δ 8.69 (d, *J* = 2.3 Hz, 1H), 8.37 – 8.28 (m, 1H), 7.79 – 7.65 (m, 4H), 7.57 – 7.48 (m, 1H), 7.43 – 7.37 (m, 1H), 7.36 – 7.24 (m, 5H), 7.21 – 7.16 (m, 3H), 7.08 – 7.00 (m, 2H), 6.92 – 6.80 (m, 2H), 6.79 – 6.69 (m, 2H), 6.38 (t, *J* = 7.7 Hz, 1H), 6.34 – 6.27 (m, 1H), 5.13 (s, 2H).

$^{13}C$ NMR (151 MHz, $CDCl_3$) δ 160.49, 154.18, 146.65, 144.07, 143.60, 137.17, 136.44, 136.38, 136.35, 133.85, 131.99, 131.13, 130.21, 129.31, 128.54, 128.31, 128.23, 127.88, 127.44, 127.05, 126.96, 126.62, 126.50, 126.48, 125.04, 123.85, 121.02, 118.52, 118.18, 117.13, 113.97, 112.49, 100.63, 100.50, 70.48.

**HRMS** (ESI-TOF) (m/z): Calcd for $C_{39}H_{27}BrN_3O_2^+$, ([M + H]$^+$), 648.1281, found 648.1281 . **$[\alpha]_D^{20}$** = +41 (c = 0.1, $CHCl_3$).

**HPLC** conditions: Daicel Chiralpak IC column (90: 10 hexane: 2-propanol, 0.8 mL/min, 40 °C, 254 nm); tr (major) = 27.8 min, tr (minor) = 25.3 min, 93% e.e.. <1:20 dr determined by crude NMR.

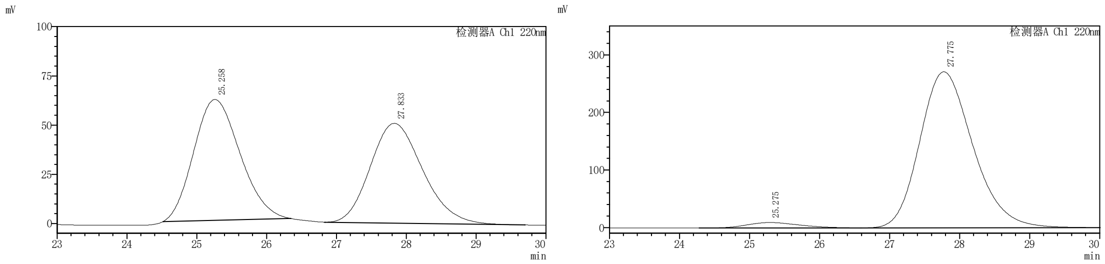


| No. | Time | Area | Area (%) | No. | Time | Area | Area (%) |
|---|---|---|---|---|---|---|---|
| 1 | 25.258 | 2832357 | 51.646 | 1 | 25.275 | 494744 | 3.344 |
| 2 | 27.833 | 2651842 | 48.354 | 2 | 27.775 | 14302329 | 96.656 |

**Supplementary Figure 72. HPLC data of dia-62.**

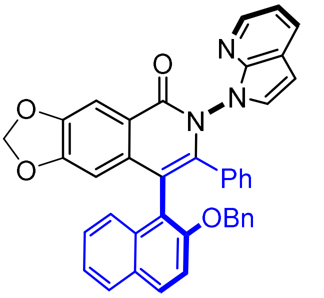


**(*S, S*)-8-(2-(benzyloxy)naphthalen-1-yl)-7-phenyl-6-(1H-pyrrolo[2,3-b]pyridin-1-yl)-[1,3]dioxolo[4,5-g]isoquinolin-5(6H)-one (63)**

White solid (20.8 mg, 34%), $^{1}$H NMR (600 MHz, $CDCl_3$) δ 8.32 (dd, *J* = 4.8, 1.6 Hz, 1H), 8.25 – 8.13 (m, 1H), 7.76 – 7.55 (m, 4H), 7.44 – 7.23 (m, 8H), 7.22 – 7.17 (m, 1H), 7.11 (dd, *J* = 3.9, 1.2 Hz, 1H), 7.06 – 6.99 (m, 3H), 6.88 – 6.82 (m, 1H), 6.73 (tt, *J* = 7.3, 1.3 Hz, 1H), 6.61 (t, *J* = 7.7 Hz, 1H), 6.58 – 6.52 (m, 1H), 6.31 (dd, *J* = 3.8, 1.1 Hz, 1H), 5.50 (d, *J* = 1.6 Hz, 1H), 5.36 (d, *J* = 1.5 Hz, 1H), 5.20 – 5.04 (m, 2H).

$^{13}$C NMR (151 MHz, $CDCl_3$) δ 160.95, 153.87, 151.47, 146.95, 144.15, 143.18, 142.50, 137.62, 134.81, 132.08, 129.42, 129.08, 128.65, 128.47, 128.45, 128.10, 128.08, 127.91, 127.42, 126.57, 126.36, 126.28, 124.82, 124.59, 123.20, 122.11, 120.47, 120.16, 118.38, 116.98, 114.28, 109.00, 108.47, 101.69, 100.30, 70.30.

**HRMS** (ESI-TOF) (m/z): Calcd for $C_{40}H_{28}N_3O_4^+$, ([M + H]$^+$), 614.2074, found 614.2074. **$[\alpha]_D^{20}$** = -25 (c = 0.1, $CHCl_3$).

**HPLC** conditions: Daicel Chiralpak IC column (70: 30 hexane: 2-propanol, 0.8 mL/min, 40 °C, 254 nm); tr (major) = 16.1 min, tr (minor) = 28.6 min, 88% e.e.. >20:1 dr determined by crude NMR.

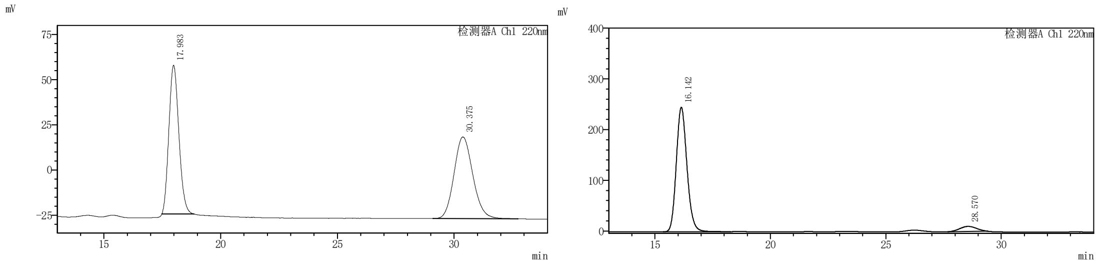


| No. | Time | Area | Area (%) | No. | Time | Area | Area (%) |
|---|---|---|---|---|---|---|---|
| 1 | 17.983 | 2452755 | 49.116 | 1 | 16.142 | 7887628 | 94.009 |
| 2 | 30.375 | 2541035 | 50.884 | 2 | 28.570 | 502646 | 5.991 |

**Supplementary Figure 73. HPLC data of 63.**

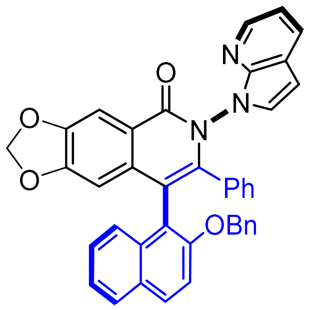


**($R,S$)-8-(2-(benzyloxy)naphthalen-1-yl)-7-phenyl-6-(1H-pyrrolo[2,3-b]pyridin-1-yl)-[1,3]dioxolo[4,5-g]isoquinolin-5(6H)-one (dia-63)**

White solid (32.5 mg, 53%), $^{1}$H NMR (600 MHz, $CDCl_3$) δ 8.36 (dt, $J$ = 4.8, 1.4 Hz, 1H), 8.24 (dd, $J$ = 8.4, 1.3 Hz, 1H), 7.89 (d, $J$ = 8.7 Hz, 1H), 7.74 (dd, $J$ = 7.8, 1.3 Hz, 1H), 7.63 (dd, $J$ = 13.9, 8.6 Hz, 2H), 7.38 (dd, $J$ = 8.5, 6.9 Hz, 1H), 7.33 – 7.27 (m, 3H), 7.26 – 7.22 (m, 3H), 7.20 – 7.16 (m, 1H), 7.13 (dd, $J$ = 9.0, 1.2 Hz, 1H), 7.09 – 6.99 (m, 3H), 6.87 (dd, $J$ = 7.8, 1.7 Hz, 1H), 6.80 (t, $J$ = 7.6 Hz, 1H), 6.70 (td, $J$ = 7.5, 1.4 Hz, 1H), 6.36 (t, $J$ = 7.7 Hz, 1H), 6.28 (dd, $J$ = 3.8, 1.2 Hz, 1H), 5.49 (d, $J$ = 1.4 Hz, 1H), 5.40 (d, $J$ = 1.3 Hz, 1H), 5.19 (s, 2H).

$^{13}$C NMR (151 MHz, CDCl3) δ 160.96, 154.19, 151.41, 146.85, 144.02, 142.97, 142.52, 137.65, 134.11, 131.99, 129.46, 129.28, 128.73, 128.53, 128.51, 128.47, 128.16, 128.11, 127.71, 127.59, 126.54, 126.39, 126.35, 126.28, 125.22, 124.80, 123.31, 122.16, 120.57, 120.27, 118.52, 117.01, 113.70, 108.97, 108.57, 101.66, 100.24, 70.42.

**HRMS** (ESI-TOF) (m/z): Calcd for $C_{40}H_{28}N_3O_4^+$, ([M + H]$^+$), 614.2074, found 614.2068. **$[\alpha]_D^{20}$** = +22 (c = 0.1, $CHCl_3$).

**HPLC** conditions: Daicel Chiralpak IC column (80: 20 hexane: 2-propanol, 0.8 mL/min, 40 °C, 254 nm); tr (major) = 22.2 min, tr (minor) = 34.1 min, 75% e.e.. <1:20 dr determined by crude NMR.

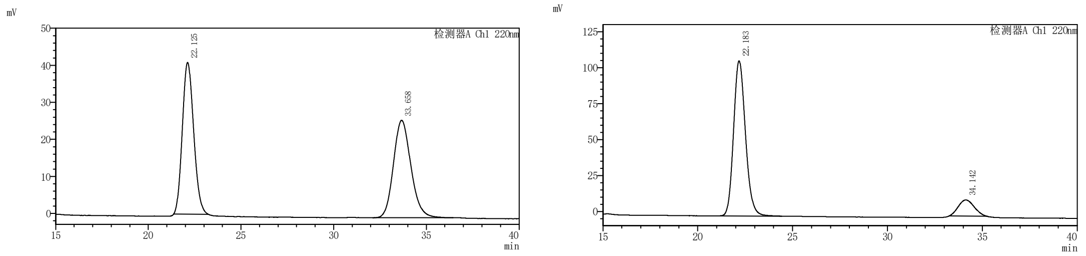


| No. | Time | Area | Area (%) | No. | Time | Area | Area (%) |
|---|---|---|---|---|---|---|---|
| 1 | 22.125 | 1746748 | 50.274 | 1 | 22.183 | 4709399 | 87.795 |
| 2 | 33.658 | 1727707 | 49.726 | 2 | 34.142 | 654678 | 12.205 |

**Supplementary Figure 74. HPLC data of dia-63.**

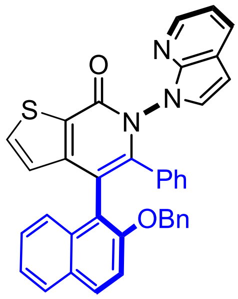


**($S,S$)-4-(2-(benzyloxy)naphthalen-1-yl)-5-phenyl-6-(1H-pyrrolo[2,3-b]pyridin-1-yl)thieno[2,3-c]pyridin-7(6H)-one (64)**

White solid (23.6 mg, 41%), $^{1}$H NMR (600 MHz, $CDCl_3$) δ 8.30 (dd, $J$ = 4.9, 1.5 Hz, 1H), 7.71 (dd, $J$ = 7.8, 1.5 Hz, 1H), 7.67 – 7.63 (m, 1H), 7.60 (d, $J$ = 9.1 Hz, 1H), 7.56 (d, $J$ = 8.5 Hz, 1H), 7.52 (d, $J$ = 5.2 Hz, 1H), 7.36 (ddd, $J$ = 8.3, 6.8, 1.3 Hz, 1H), 7.28 – 7.21 (m, 4H), 7.18 (dd, $J$ = 5.3, 2.1 Hz, 1H), 7.16 – 7.14 (m, 2H), 7.12 (dd, $J$ = 8.5, 3.8 Hz, 1H), 7.03 – 6.97 (m, 3H), 6.81 (d, $J$ = 7.8 Hz, 1H), 6.73 (tt, $J$ = 7.5, 1.3 Hz, 1H), 6.60 (t, $J$ = 7.7 Hz, 1H), 6.53 (t, $J$ = 7.6 Hz, 1H), 6.45 (d, $J$ = 5.2 Hz, 1H), 6.26 (d, $J$ = 3.8 Hz, 1H), 4.98 (s, 2H).

$^{13}$C NMR (151 MHz, $CDCl_3$) δ 156.37, 152.80, 146.24, 145.84, 143.79, 142.86, 136.16, 133.35, 132.91, 131.06, 129.14, 128.99, 128.59, 127.72, 127.61, 127.47, 127.29, 127.22, 127.13, 126.43, 125.88, 125.61, 125.43, 124.24, 123.71, 122.63, 117.65, 116.09, 113.61, 110.32, 99.68, 69.42.

**HRMS** (ESI-TOF) (m/z): Calcd for $C_{37}H_{26}N_3O_2S^+$, ([M + H]$^+$), 576.1740, found 576.1735. **$[\alpha]_D^{20}$** = -35 (c = 0.1, $CHCl_3$).

**HPLC** conditions: Daicel Chiralpak OD-3 column (80: 20 hexane: 2-propanol, 0.8 mL/min, 40 °C, 254 nm); tr (major) = 11.2 min, tr (minor) = 15.6 min, 95% e.e.. >20:1 dr determined by crude NMR.

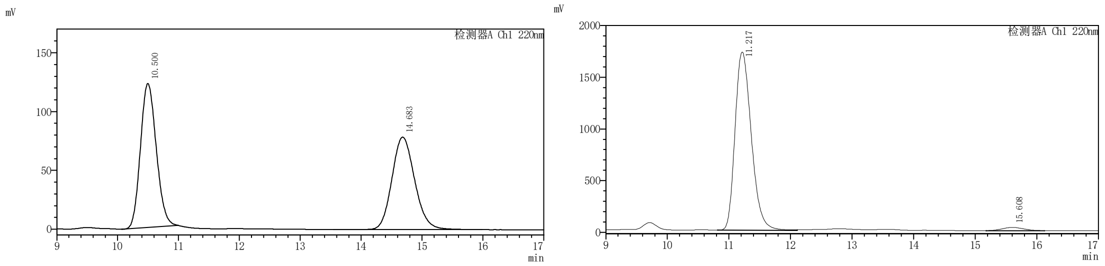


| No. | Time | Area | Area (%) | No. | Time | Area | Area (%) |
|---|---|---|---|---|---|---|---|
| 1 | 10.500 | 2213690 | 51.936 | 1 | 11.217 | 31743340 | 97.670 |
| 2 | 14.683 | 2048643 | 48.064 | 2 | 15.608 | 757140 | 2.330 |

**Supplementary Figure 75. HPLC data of 64.**

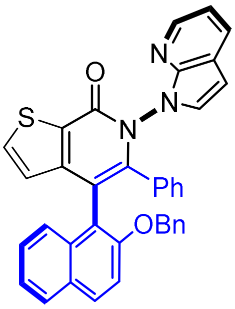


**(*R*,*S*)-4-(2-(benzyloxy)naphthalen-1-yl)-5-phenyl-6-(1H-pyrrolo[2,3-b]pyridin-1-yl)thieno[2,3-c]pyridin-7(6H)-one (*dia*-64)**

White solid (33.4 mg, 58%), $^1$H NMR (600 MHz, $CDCl_3$) δ 8.34 (dd, *J* = 4.8, 1.5 Hz, 1H), 7.81 (d, *J* = 8.5 Hz, 1H), 7.77 – 7.65 (m, 3H), 7.61 (d, *J* = 5.2 Hz, 1H), 7.41 (ddd, *J* = 8.4, 6.7, 1.3 Hz, 1H), 7.36 – 7.27 (m, 4H), 7.18-7.21 (m, 3H), 7.17 – 7.12 (m, 1H), 7.08 (d, *J* = 3.8 Hz, 1H), 7.05 (dd, *J* = 7.8, 4.8 Hz, 1H), 6.92 (d, *J* = 7.7 Hz, 1H), 6.81 (d, *J* = 7.9 Hz, 1H), 6.78 – 6.72 (m, 1H), 6.56 (d, *J* = 5.2 Hz, 1H), 6.43 (t, *J* = 7.7 Hz, 1H), 6.33 (d, *J* = 3.8 Hz, 1H).

$^{13}$C NMR (151 MHz, $CDCl_3$) δ 157.47, 153.94, 147.30, 146.90, 144.76, 144.06, 137.26, 134.30, 133.54, 131.99, 130.05, 129.27, 128.87, 128.82, 128.56, 128.54, 128.47, 128.26, 127.84, 127.82, 126.92, 126.59, 126.49, 125.25, 125.16, 123.83, 119.26, 118.57, 117.09, 114.14, 111.28, 100.42, 70.66.

**HRMS** (ESI-TOF) (m/z): Calcd for $C_{37}H_{26}N_3O_2S^+$, ([M + H]$^+$), 576.1740, found 576.1748. **$[\alpha]_D^{20}$** = +38 (c = 0.1, $CHCl_3$).

**HPLC** conditions: Daicel Chiralpak IC column (80: 20 hexane: 2-propanol, 0.8 mL/min, 40 °C, 254 nm); tr (major) = 23.2 min, tr (minor) = 49.2 min, 89% e.e.. <1:20 dr determined by crude NMR.

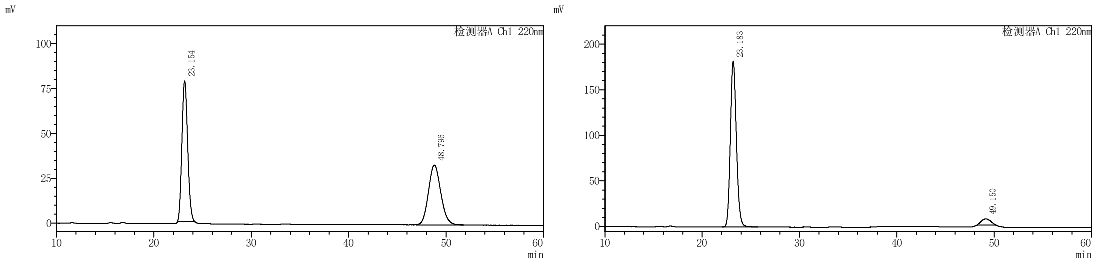


| No. | Time | Area | Area (%) | No. | Time | Area | Area (%) |
|---|---|---|---|---|---|---|---|
| 1 | 23.154 | 3228627 | 52.256 | 1 | 23.183 | 7846993 | 94.597 |
| 2 | 48.796 | 2949829 | 47.744 | 2 | 49.150 | 448163 | 5.403 |

**Supplementary Figure 76. HPLC data of dia-64.**

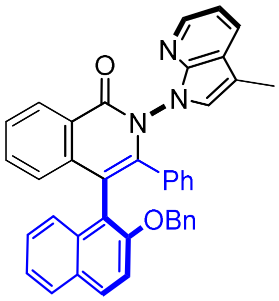


**(*S,S*)-4-(2-(benzyloxy)naphthalen-1-yl)-2-(3-methyl-1H-pyrrolo[2,3-b]pyridin-1-yl)-3-phenylisoquinolin-1(2H)-one (65)**

White solid (39.7 mg, 68%), $^1$H NMR (600 MHz, $CDCl_3$) δ 8.55 (dd, $J$ = 7.8, 1.7 Hz, 1H), 8.31 (dd, $J$ = 4.8, 1.5 Hz, 1H), 7.77 – 7.65 (m, 3H), 7.62 (d, $J$ = 8.5 Hz, 1H), 7.50 – 7.43 (m, 2H), 7.40 (ddd, $J$ = 8.3, 6.7, 1.3 Hz, 1H), 7.35 – 7.22 (m, 7H), 7.09 (d, $J$ = 9.0 Hz, 1H), 7.02 (dd, $J$ = 7.8, 4.8 Hz, 1H), 6.94 – 6.83 (m, 3H), 6.77 (tt, $J$ = 7.6, 1.4 Hz, 1H), 6.69 – 6.56 (m, 2H), 5.12 – 4.98 (m, 2H), 2.13 (s, 3H).

$^{13}$C NMR (151 MHz, $CDCl_3$) δ 161.65, 154.15, 147.70, 144.01, 143.38, 137.62, 137.32, 134.51, 133.18, 132.63, 129.86, 128.73, 128.68, 128.50, 128.10, 128.02, 127.39, 127.16, 126.99, 126.79, 126.45, 126.39, 125.72, 125.62, 125.50, 124.97, 123.56, 119.41, 118.67, 116.38, 114.56, 113.49, 112.68, 110.05, 70.24, 9.64.

**HRMS** (ESI-TOF) (m/z): Calcd for $C_{40}H_{30}N_3O_2^+$, ([M + H]$^+$), 584.2333, found 584.2338. **[α]$_D^{20}$** = -22 (c = 0.1, $CHCl_3$).

**HPLC** conditions: Daicel Chiralpak OD-3 column (80: 20 hexane: 2-propanol, 0.8 mL/min, 40 °C, 254 nm); tr (major) = 6.7 min, tr (minor) = 8.6 min, 90% e.e.. >20:1 dr determined by crude NMR.

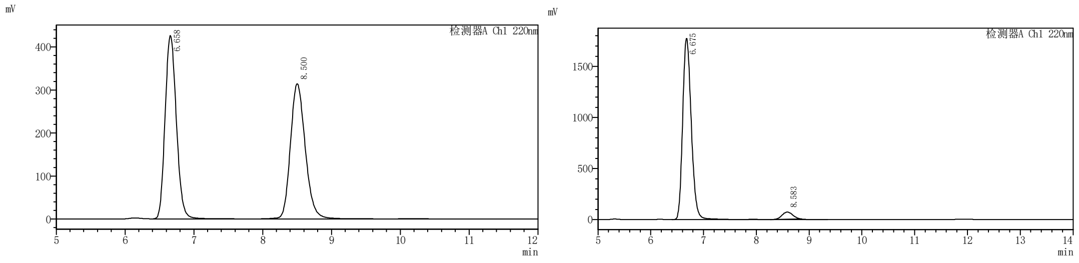


| No. | Time | Area | Area (%) | No. | Time | Area | Area (%) |
|---|---|---|---|---|---|---|---|
| 1 | 6.658 | 4880672 | 50.468 | 1 | 6.675 | 20361812 | 95.332 |
| 2 | 8.500 | 4790154 | 49.532 | 2 | 8.583 | 997005 | 4.668 |

**Supplementary Figure 77. HPLC data of 65.**

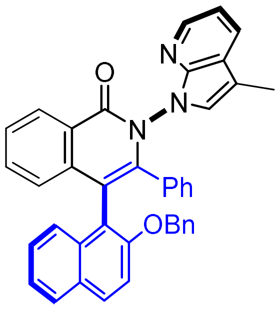


**(*R,S*)-4-(2-(benzyloxy)naphthalen-1-yl)-2-(3-methyl-1H-pyrrolo[2,3-b]pyridin-1-yl)-3-phenylisoquinolin-1(2H)-one (*dia*-65)**

White solid (43.7 mg, 75%), $^1$H NMR (600 MHz, $CDCl_3$) δ 8.62 – 8.47 (m, 1H), 8.31 (dd, $J$ = 4.8, 1.5 Hz, 1H), 7.81 (dd, $J$ = 8.5, 1.2 Hz, 1H), 7.74 – 7.65 (m, 3H), 7.53 – 7.43 (m, 2H), 7.38 (ddd, $J$ = 8.3, 6.8, 1.3 Hz, 1H), 7.34 – 7.11 (m, 8H), 7.02 (dd, $J$ = 7.8, 4.8 Hz, 1H), 6.98 (d, $J$ = 7.8 Hz, 1H), 6.92 (dd, $J$ = 7.8, 1.5 Hz, 1H), 6.87 – 6.77 (m, 2H), 6.73 (tt, $J$ = 7.5, 1.3 Hz, 1H), 6.42 (t, $J$ = 7.7 Hz, 1H), 5.14 (d, $J$ = 2.3 Hz, 2H), 2.12 (s, 3H).

$^{13}$C NMR (151 MHz, $CDCl_3$) δ 161.73, 154.26, 147.45, 143.83, 143.21, 137.66, 137.36, 134.00, 133.18, 132.52, 129.91, 128.81, 128.64, 128.49, 128.35, 128.04, 127.76, 127.73, 127.27, 127.00, 126.84, 126.53, 126.37, 126.00, 125.60, 125.49, 125.39, 123.75, 119.53, 118.98, 116.40, 114.05, 112.74, 109.89, 70.48, 9.60.

**HRMS** (ESI-TOF) (m/z): Calcd for $C_{40}H_{30}N_3O_2^+$, ([M + H]$^+$), 584.2333, found 584.2326. **[α]$_D^{20}$** = +32 (c = 0.1, $CHCl_3$).

**HPLC** conditions: Daicel Chiralpak OD-3 column (80: 20 hexane: 2-propanol, 0.8 mL/min, 40 °C, 254 nm); tr (major) = 11.1 min, tr (minor) = 9.0 min, 92% e.e.. <1:20 dr determined by crude NMR.

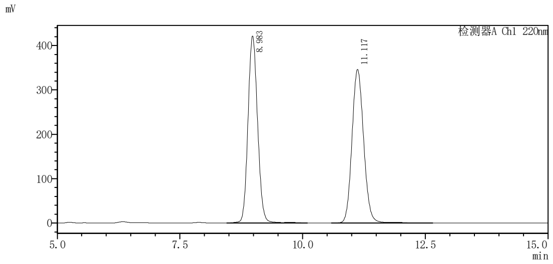

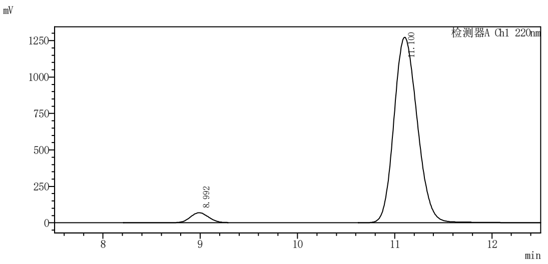

| No. | Time | Area | Area (%) | No. | Time | Area | Area (%) |
|---|---|---|---|---|---|---|---|
| 1 | 8.983 | 5731451 | 49.953 | 1 | 8.992 | 878348 | 3.951 |
| 2 | 11.117 | 5742134 | 50.047 | 2 | 11.100 | 21352648 | 96.049 |

**Supplementary Figure 78. HPLC data of dia-65.**

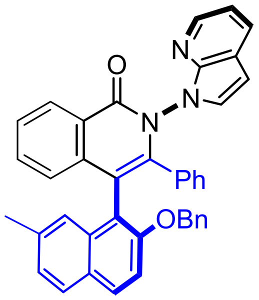

**(*S,S*)-4-(2-(benzyloxy)-7-methylnaphthalen-1-yl)-3-phenyl-2-(1H-pyrrolo[2,3-b]pyridin-1-yl)isoquinolin-1(2H)-one (66)**

White solid (35.0 mg, 60%), $^1$H NMR (600 MHz, $CDCl_3$) δ 8.58 (dd, *J* = 7.7, 1.7 Hz, 1H), 8.32 (dd, *J* = 4.8, 1.6 Hz, 1H), 7.75 (dd, *J* = 7.8, 1.6 Hz, 1H), 7.62 (t, *J* = 9.3 Hz, 2H), 7.48 (dtd, *J* = 19.5, 7.1, 1.5 Hz, 2H), 7.38 – 7.22 (m, 6H), 7.21 – 7.17 (m, 1H), 7.16 – 7.10 (m, 2H), 7.08 – 6.99 (m, 2H), 6.95 – 6.91 (m, 1H), 6.90 – 6.84 (m, 1H), 6.76 (td, *J* = 7.5, 1.4 Hz, 1H), 6.65 (t, *J* = 7.6 Hz, 1H), 6.57 (t, *J* = 7.7 Hz, 1H), 6.32 (dd, *J* = 3.9, 1.1 Hz, 1H), 5.10 – 4.98 (m, 2H), 2.42 (s, 3H).

$^{13}$C NMR (151 MHz, $CDCl_3$) δ 161.64, 154.25, 147.04, 144.20, 143.03, 137.66, 137.35, 136.59, 134.65, 133.29, 132.46, 129.61, 129.13, 128.66, 128.54, 128.49, 128.11, 128.05, 127.93, 127.37, 127.10, 127.02, 126.48, 126.46, 125.96, 125.77, 125.50, 123.76, 118.42, 117.90, 117.00, 113.48, 113.01, 100.43, 70.20, 22.16.

**HRMS** (ESI-TOF) (m/z): Calcd for $C_{40}H_{30}N_3O_2^+$, ([M + H]$^+$), 584.2333, found 584.2334. **$[\alpha]_D^{20}$** = -34 (c = 0.1, $CHCl_3$).

**HPLC** conditions: Daicel Chiralpak IC column (80: 20 hexane: 2-propanol, 0.8 mL/min, 40 °C, 254 nm); tr (major) = 16.5 min, tr (minor) = 32.1 min, 96% e.e.. >20:1 dr determined by crude NMR.

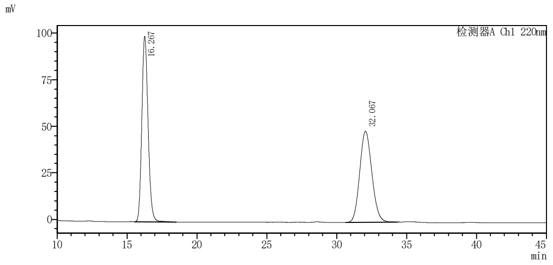

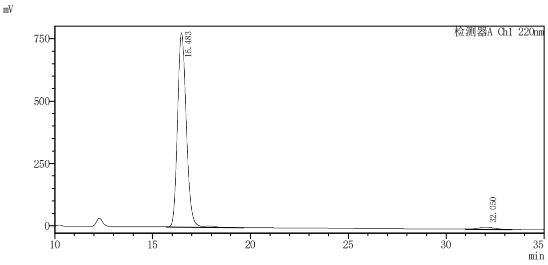

| No. | Time | Area | Area (%) | No. | Time | Area | Area (%) |
|---|---|---|---|---|---|---|---|
| 1 | 16.267 | 3109499 | 51.291 | 1 | 16.483 | 24340504 | 98.085 |
| 2 | 32.067 | 2952930 | 48.709 | 2 | 32.050 | 475095 | 1.915 |

**Supplementary Figure 79. HPLC data of 66.**

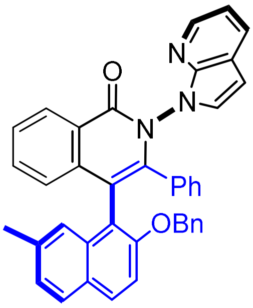


**(*R*,*S*)-4-(2-(benzyloxy)-7-methylnaphthalen-1-yl)-3-phenyl-2-(1H-pyrrolo[2,3-b]pyridin-1-yl)isoquinolin-1(2H)-one (*dia*-66)**

White solid (43.2 mg, 74%), $^{1}$H NMR (600 MHz, $CDCl_3$) δ 8.62 – 8.54 (m, 1H), 8.35 (dt, *J* = 4.8, 1.3 Hz, 1H), 7.73 (dt, *J* = 7.7, 1.3 Hz, 1H), 7.65 (d, *J* = 9.0 Hz, 1H), 7.60 – 7.58 (m, 1H), 7.56 (d, *J* = 8.3 Hz, 1H), 7.53 – 7.45 (m, 2H), 7.31 – 7.25 (m, 3H), 7.20 – 7.07 (m, 5H), 7.06 – 7.01 (m, 2H), 6.99 – 6.95 (m, 1H), 6.88 (d, *J* = 7.9 Hz, 1H), 6.81 (t, *J* = 7.6 Hz, 1H), 6.72 (tt, *J* = 7.5, 1.2 Hz, 1H), 6.40 (t, *J* = 7.6 Hz, 1H), 6.30 (dd, *J* = 3.9, 1.0 Hz, 1H), 5.11 (s, 2H), 2.43 (s, 3H).

$^{13}$C NMR (151 MHz, $CDCl_3$) δ 161.72, 154.35, 146.85, 144.01, 142.86, 137.75, 137.39, 136.65, 134.19, 133.26, 132.42, 129.65, 129.23, 128.80, 128.67, 128.47, 128.10, 127.70, 127.60, 127.12, 127.06, 126.53, 126.42, 126.38, 126.14, 125.62, 124.12, 118.54, 118.17, 117.01, 116.99, 113.13, 112.98, 100.24, 70.41, 22.02.

**HRMS** (ESI-TOF) (m/z): Calcd for $C_{40}H_{30}N_3O_2^+$, ([M + H]$^+$), 584.2333, found 584.2330. **[α]$_D$$^{20}$** = +52 (c = 0.1, $CHCl_3$).

**HPLC** conditions: Daicel Chiralpak IC column (80: 20 hexane: 2-propanol, 0.8 mL/min, 40 °C, 254 nm); tr (major) = 12.3 min, tr (minor) = 23.5 min, 88% e.e.. dr = 1:3.6 determined by crude NMR.

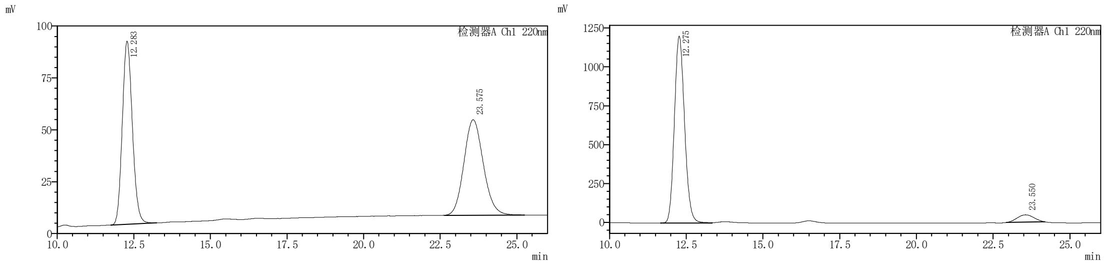


| No. | Time | Area | Area (%) | No. | Time | Area | Area (%) |
|---|---|---|---|---|---|---|---|
| 1 | 12.283 | 2016948 | 49.886 | 1 | 12.275 | 27649652 | 93.937 |
| 2 | 23.575 | 2026164 | 50.114 | 2 | 23.550 | 1784714 | 6.063 |

**Supplementary Figure 80. HPLC data of dia-66.**

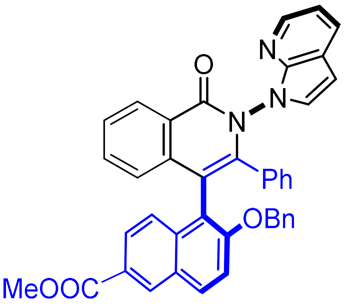


**(*S*,*S*)-methyl 6-(benzyloxy)-5-(1-oxo-3-phenyl-2-(1H-pyrrolo[2,3-b]pyridin-1-yl)-1,2-dihydroisoquinolin-4-yl)-2-naphthoate (67)**

White solid (40.8 mg, 65%), $^{1}$H NMR (600 MHz, $CDCl_3$) δ 8.60 (dt, *J* = 7.6, 1.5 Hz, 1H), 8.48 (d, *J* = 1.9 Hz, 1H), 8.38 (dd, *J* = 4.8, 1.5 Hz, 1H), 7.98 (dt, *J* = 9.0, 1.6 Hz, 1H), 7.81 – 7.75 (m, 2H), 7.67 (d, *J* = 8.9 Hz, 1H), 7.53 (tdd, *J* = 8.6, 6.4, 3.6 Hz, 2H), 7.30 – 7.17 (m, 6H), 7.15 – 7.10 (m, 2H), 7.08 (ddd, *J* = 7.8, 4.8, 1.3 Hz, 1H), 6.91 (dd, *J* = 7.5, 1.8 Hz, 1H), 6.87 – 6.83 (m, 1H), 6.78 (td, *J* = 7.5, 1.4 Hz, 1H), 6.64 (t, *J* = 7.6 Hz, 1H), 6.60 (t, *J* = 7.7 Hz, 1H), 6.35 (dd, *J* = 3.8, 1.3 Hz, 1H), 5.14 – 5.01 (m, 2H), 3.94 (s, 3H).

$^{13}$C NMR (151 MHz, $CDCl_3$) δ 167.24, 161.52, 156.16, 146.72, 143.87, 143.29, 137.37, 136.82, 136.72, 133.38, 132.19, 131.59, 131.47, 129.57, 128.89, 128.60, 128.54, 128.39, 128.32, 127.94, 127.55, 127.32, 126.60, 126.28, 126.23, 125.68, 125.47, 125.23, 125.06, 118.65, 118.51, 117.10, 115.00, 112.39, 100.63, 70.17, 52.16.

**HRMS** (ESI-TOF) (m/z): Calcd for $C_{41}H_{30}N_3O_4^+$, ([M + H]$^+$), 628.2231, found 628.2221. **[α]$_D^{20}$** = -40 (c = 0.1, $CHCl_3$).

**HPLC** conditions: Daicel Chiralpak OD-3 column (80: 20 hexane: 2-propanol, 0.8 mL/min, 40 °C, 254 nm); tr (major) = 27.6 min, tr (minor) = 25.0 min, 96% e.e.. >20:1 dr determined by crude NMR.

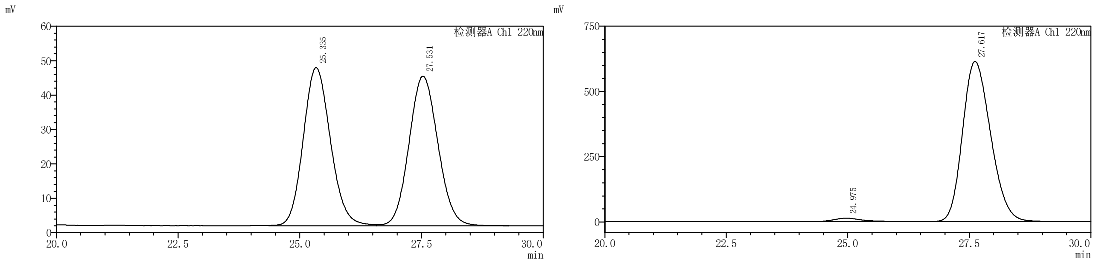


| No. | Time | Area | Area (%) | No. | Time | Area | Area (%) |
|---|---|---|---|---|---|---|---|
| 1 | 25.335 | 1797370 | 49.903 | 1 | 24.975 | 515235 | 2.045 |
| 2 | 27.531 | 1804345 | 50.097 | 2 | 27.617 | 24683808 | 97.955 |

**Supplementary Figure 81. HPLC data of 67.**

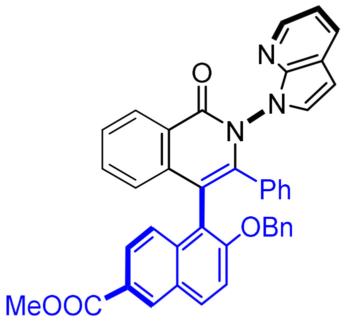


**(*R,S*)-methyl 6-(benzyloxy)-5-(1-oxo-3-phenyl-2-(1H-pyrrolo[2,3-b]pyridin-1-yl)-1,2-dihydroisoquinolin-4-yl)-2-naphthoate (*dia*-67)**

White solid (42.6 mg, 68%), $^1$H NMR (600 MHz, $CDCl_3$) δ 8.59 (dd, *J* = 7.7, 1.7 Hz, 1H), 8.44 (d, *J* = 1.8 Hz, 1H), 8.35 (dd, *J* = 4.8, 1.6 Hz, 1H), 7.93 (dd, *J* = 8.9, 1.8 Hz, 1H), 7.87 (d, *J* = 8.9 Hz, 1H), 7.82 (d, *J* = 9.1 Hz, 1H), 7.75 (dd, *J* = 7.8, 1.6 Hz, 1H), 7.51 (dtd, *J* = 18.1, 7.2, 1.5 Hz, 2H), 7.36 – 7.23 (m, 5H), 7.14-7.18 (m, 3H), 7.09 – 7.02 (m, 2H), 6.94 – 6.89 (m, 1H), 6.88 (d, *J* = 7.8 Hz, 1H), 6.82 (t, *J* = 7.6 Hz, 1H), 6.73 (t, *J* = 7.5 Hz, 1H), 6.39 (t, *J* = 7.7 Hz, 1H), 6.31 (d, *J* = 3.8 Hz, 1H), 5.18 (s, 2H), 3.92 (s, 3H).

$^{13}$C NMR (151 MHz, $CDCl_3$) δ 167.24, 161.59, 156.14, 146.75, 144.04, 143.19, 137.39, 136.86, 136.34, 133.37, 132.15, 131.64, 131.19, 129.33, 128.81, 128.71, 128.59, 128.29, 128.23, 128.10, 127.94, 127.70, 127.27, 126.56, 126.52, 126.21, 125.67, 125.52, 125.40, 125.31, 118.92, 118.57, 117.11, 114.55, 112.37, 70.43, 52.10.

**HRMS** (ESI-TOF) (m/z): Calcd for $C_{41}H_{30}N_3O_4^+$, ([M + H]$^+$), 628.2231, found 628.2228. **[α]$_D^{20}$** = +27 (c = 0.1, $CHCl_3$).

**HPLC** conditions: Daicel Chiralpak IC column (80: 20 hexane: 2-propanol, 0.8 mL/min, 40 °C, 254 nm); tr (major) = 13.9 min, tr (minor) = 21.0 min, 92% e.e.. dr = 1:9 determined by crude NMR.

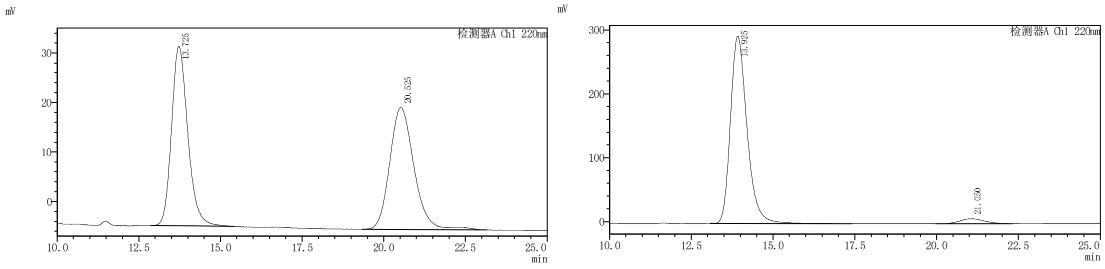


| No. | Time | Area | Area (%) | No. | Time | Area | Area (%) |
|---|---|---|---|---|---|---|---|
| 1 | 13.725 | 1258647 | 49.721 | 1 | 13.925 | 10333271 | 96.176 |

| | | | | | | | |
|---|---|---|---|---|---|---|---|
| 2 | 20.525 | 1272755 | 50.279 | 2 | 21.050 | 410836 | 3.824 |

**Supplementary Figure 82. HPLC data of dia-67.**

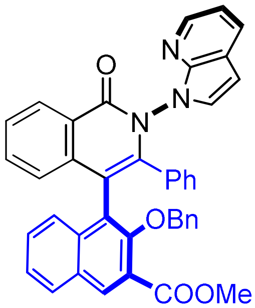


**(*S,S*)-methyl-3-(benzyloxy)-4-(1-oxo-3-phenyl-2-(1H-pyrrolo[2,3-b]pyridin-1-yl)-1,2-dihydroisoquinolin-4-yl)-2-naphthoate (68)**

White solid (20.0 mg, 32%), $^1$H NMR (600 MHz, $CDCl_3$) δ 8.59 – 8.52 (m, 1H), 8.36 (dd, $J$ = 4.8, 1.5 Hz, 1H), 8.30 (s, 1H), 7.85 (d, $J$ = 8.1 Hz, 1H), 7.76 (dt, $J$ = 7.8, 1.2 Hz, 1H), 7.69 – 7.64 (m, 1H), 7.56 – 7.48 (m, 5H), 7.46 (ddd, $J$ = 8.2, 7.0, 1.3 Hz, 1H), 7.43 – 7.38 (m, 2H), 7.35 (dt, $J$ = 7.9, 1.6 Hz, 1H), 7.30 (td, $J$ = 7.3, 1.3 Hz, 1H), 7.11 – 7.08 (m, 1H), 7.07 (ddd, $J$ = 7.8, 4.7, 1.0 Hz, 1H), 7.04 – 6.99 (m, 1H), 6.81 (dd, $J$ = 7.8, 1.6 Hz, 1H), 6.75 (td, $J$ = 7.5, 1.2 Hz, 1H), 6.67 – 6.56 (m, 2H), 6.33 (dd, $J$ = 3.8, 1.0 Hz, 1H), 5.00 (d, $J$ = 9.7 Hz, 1H), 4.72 (d, $J$ = 9.8 Hz, 1H), 3.84 (s, 3H).

$^{13}$C NMR (151 MHz, $CDCl_3$) δ 166.69, 161.45, 153.57, 147.05, 144.29, 144.26, 137.72, 136.94, 135.87, 133.52, 133.40, 131.88, 129.33, 129.26, 129.02, 128.95, 128.94, 128.72, 128.51, 128.38, 128.35, 127.88, 127.60, 127.32, 127.05, 126.39, 126.34, 125.67, 125.61, 125.48, 125.42, 125.31, 118.32, 117.17, 111.65, 100.46, 76.43, 52.37.

**HRMS** (ESI-TOF) (m/z): Calcd for $C_{41}H_{30}N_3O_4^+$, ([M + H]$^+$), 628.2231, found 628.2229. **[α]$_D^{20}$** = -31 (c = 0.1, $CHCl_3$).

**HPLC** conditions: Daicel Chiralpak IC column (80: 20 hexane: 2-propanol, 0.8 mL/min, 40 °C, 254 nm); tr (major) = 15.6 min, tr (minor) = 22.9 min, 92% e.e.. >20:1 dr determined by crude NMR.

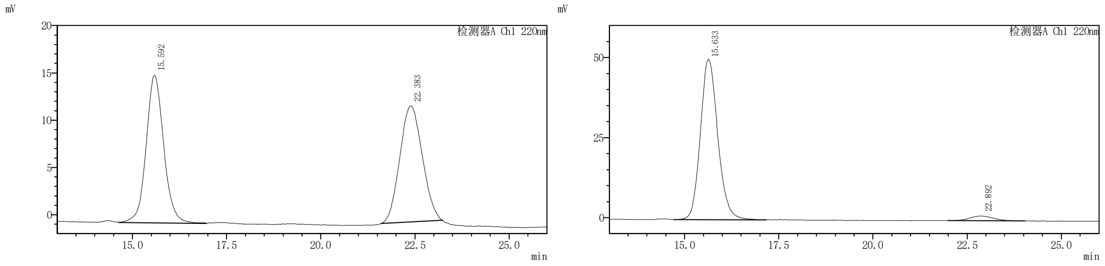


| No. | Time | Area | Area (%) | No. | Time | Area | Area (%) |
|---|---|---|---|---|---|---|---|
| 1 | 15.592 | 509029 | 49.950 | 1 | 15.633 | 1613607 | 96.120 |
| 2 | 22.383 | 510048 | 50.050 | 2 | 22.892 | 65140 | 3.880 |

**Supplementary Figure 83. HPLC data of 68.**

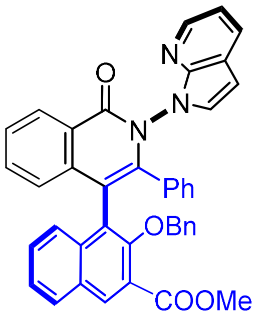


**(*R,S*)-methyl-3-(benzyloxy)-4-(1-oxo-3-phenyl-2-(1H-pyrrolo[2,3-b]pyridin-1-yl)-1,2-dihydroisoquinolin-4-yl)-2-naphthoate (*dia*-68)**

White solid (42.0 mg, 67%), $^1$H NMR (600 MHz, $CDCl_3$) δ 8.57 (ddt, $J$ = 7.1, 4.9, 2.5 Hz, 1H), 8.33 (s, 1H), 8.26 (dd, $J$ = 4.8, 1.5 Hz, 1H), 7.83 (dd, $J$ = 17.3, 8.3 Hz, 2H), 7.71 (dt, $J$ = 7.8, 1.4 Hz, 1H), 7.57 – 7.49 (m, 3H), 7.45 – 7.40 (m, 1H), 7.35 – 7.29 (m, 3H), 7.25 – 7.22 (m, 1H), 7.10 – 7.02 (m, 2H), 6.99 (dtd, $J$ = 9.2, 4.5, 1.6 Hz, 2H), 6.90 (dd, $J$ = 3.9, 1.3 Hz, 1H), 6.86 – 6.78 (m, 2H), 6.70 (td, $J$ = 7.5, 1.4 Hz, 1H), 6.39 – 6.27 (m, 2H), 5.16 (d, $J$ = 10.5 Hz, 1H), 4.48 (d, $J$ = 10.5 Hz, 1H), 3.91 (s, 3H).

$^{13}C$ NMR (151 MHz, $CDCl_3$) δ 166.53, 161.25, 153.59, 146.47, 144.02, 143.86, 137.84, 137.59, 135.85, 133.61, 133.49, 131.67, 129.33, 129.22, 129.05, 128.98, 128.85, 128.46, 128.38, 128.29, 128.07, 127.96, 127.57, 127.31, 126.80, 126.66, 126.17, 126.04, 125.77, 125.56, 124.69, 118.51, 117.02, 111.65, 100.40, 76.11, 52.35.

**HRMS** (ESI-TOF) (m/z): Calcd for $C_{41}H_{30}N_3O_4^+$, ($[M + H]^+$), 628.2231, found 628.2234. **$[\alpha]_D^{20}$** = +21 (c = 0.1, $CHCl_3$).

**HPLC** conditions: Daicel Chiralpak IC column (80: 20 hexane: 2-propanol, 0.8 mL/min, 40 °C, 254 nm); tr (major) = 12.1 min, tr (minor) = 27.5 min, 98% e.e.. <1:20 dr was determined by crude NMR.

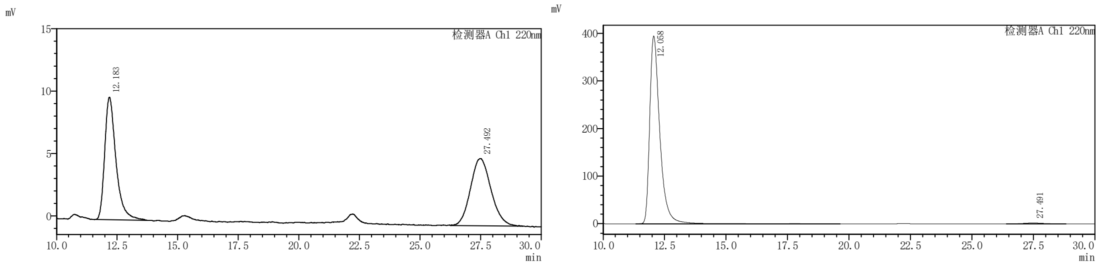


| No. | Time | Area | Area (%) | No. | Time | Area | Area (%) |
|---|---|---|---|---|---|---|---|
| 1 | 12.183 | 318994 | 49.911 | 1 | 12.058 | 11380638 | 98.919 |
| 2 | 27.492 | 320125 | 50.089 | 2 | 27.491 | 124323 | 1.081 |

**Supplementary Figure 84. HPLC data of dia-68.**

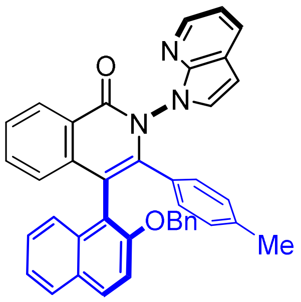


**(*S*, *S*)-4-(2-(benzyloxy)naphthalen-1-yl)-2-(1H-pyrrolo[2,3-b]pyridin-1-yl)-3-(p-tolyl)isoquinolin-1(2H)-one (69)**

White solid (36.7 mg, 63%), $^1H$ NMR (600 MHz, $CDCl_3$) δ 8.61 – 8.50 (m, 1H), 8.33 (dd, $J$ = 4.7, 1.5 Hz, 1H), 7.77 (dd, $J$ = 7.8, 1.5 Hz, 1H), 7.74 (dd, $J$ = 8.1, 1.3 Hz, 1H), 7.70 (d, $J$ = 9.0 Hz, 1H), 7.62 (dd, $J$ = 8.5, 1.2 Hz, 1H), 7.51 – 7.43 (m, 2H), 7.40 (ddd, $J$ = 8.3, 6.8, 1.3 Hz, 1H), 7.34 – 7.26 (m, 5H), 7.14 – 7.07 (m, 3H), 7.05 (dd, $J$ = 7.8, 4.8 Hz, 1H), 6.92 – 6.85 (m, 1H), 6.75 (dd, $J$ = 7.9, 2.0 Hz, 1H), 6.44 (d, $J$ = 7.9 Hz, 1H), 6.40 – 6.36 (m, 1H), 6.34 (d, $J$ = 3.8 Hz, 1H), 5.12 – 5.02 (m, 2H), 1.92 (s, 3H).

$^{13}C$ NMR (151 MHz, $CDCl_3$) δ 161.61, 154.13, 147.15, 144.19, 143.36, 137.76, 137.68, 137.34, 134.52, 133.23, 129.79, 129.55, 129.10, 128.70, 128.56, 128.48, 128.35, 128.08, 127.89, 127.38, 127.22, 126.98, 126.78, 126.48, 125.64, 125.43, 125.03, 123.57, 118.80, 118.47, 116.96, 114.67, 112.75, 100.47, 70.29, 21.06.

**HRMS** (ESI-TOF) (m/z): Calcd for $C_{40}H_{30}N_3O_2^+$, ($[M + H]^+$), 584.2333, found 584.2324. **$[\alpha]_D^{20}$** = -24 (c = 0.1, $CHCl_3$).

**HPLC** conditions: Daicel Chiralpak IC column (80: 20 hexane: 2-propanol, 0.8 mL/min, 40 °C, 254 nm); tr (major) = 17.7 min, tr (minor) = 31.4 min, 97% e.e.. >20:1 dr was determined by crude NMR.

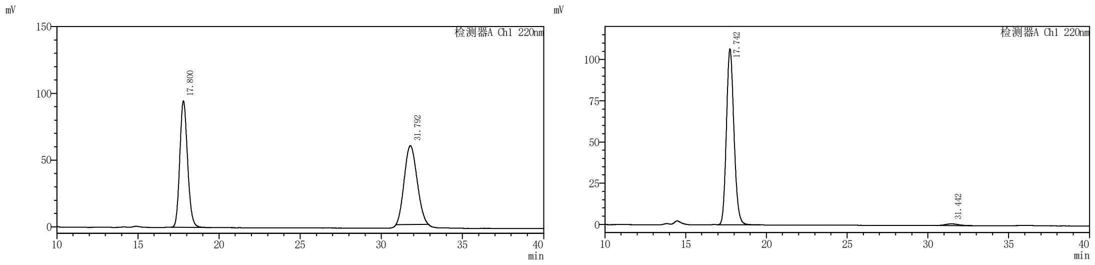


| No. | Time | Area | Area (%) | No. | Time | Area | Area (%) |
|---|---|---|---|---|---|---|---|
| 1 | 17.800 | 3152317 | 49.333 | 1 | 17.742 | 3472627 | 98.338 |
| 2 | 31.792 | 3237556 | 50.667 | 2 | 31.442 | 58687 | 1.662 |

**Supplementary Figure 85. HPLC data of 69.**

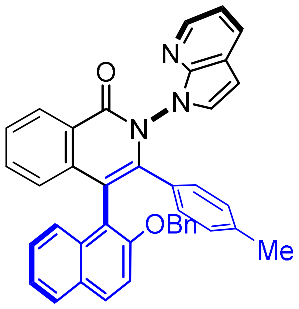


**(*R, S*)-4-(2-(benzyloxy)naphthalen-1-yl)-2-(1H-pyrrolo[2,3-b]pyridin-1-yl)-3-(p-tolyl)isoquinolin-1(2H)-one (*dia*-69)**

White solid (46.1 mg, 79%), $^{1}$H NMR (600 MHz, $CDCl_3$) δ 8.56 (dd, *J* = 7.8, 1.7 Hz, 1H), 8.34 (dd, *J* = 4.8, 1.7 Hz, 1H), 7.81 (d, *J* = 8.5 Hz, 1H), 7.77 – 7.69 (m, 2H), 7.68 (d, *J* = 8.1 Hz, 1H), 7.52 – 7.42 (m, 2H), 7.38 (ddd, *J* = 8.5, 6.8, 1.4 Hz, 1H), 7.31 – 7.25 (m, 4H), 7.21 (dd, *J* = 9.1, 1.9 Hz, 1H), 7.18 – 7.13 (m, 2H), 7.10 – 7.00 (m, 3H), 6.90 (dd, *J* = 8.0, 1.5 Hz, 1H), 6.78 (d, *J* = 8.0 Hz, 1H), 6.61 (d, *J* = 7.9 Hz, 1H), 6.32 (dd, *J* = 4.0, 1.7 Hz, 1H), 6.19 (d, *J* = 8.0 Hz, 1H), 5.14 (d, *J* = 1.9 Hz, 2H), 1.87 (s, 3H).

$^{13}$C NMR (151 MHz, $CDCl_3$) δ 161.69, 154.24, 146.90, 144.00, 143.16, 137.76, 137.73, 137.36, 134.03, 133.22, 129.85, 129.44, 129.21, 128.86, 128.64, 128.48, 128.24, 127.95, 127.73, 127.28, 127.23, 126.99, 126.82, 126.55, 125.53, 125.50, 125.40, 123.74, 119.08, 118.60, 116.98, 114.11, 112.85, 100.31, 70.49, 21.02.

**HRMS** (ESI-TOF) (m/z): Calcd for $C_{40}H_{30}N_3O_2^+$, ($[M + H]^+$), 584.2333, found 584.2324. **$[\alpha]_D^{20}$** = +22 (c = 0.1, $CHCl_3$).

**HPLC** conditions: Daicel Chiralpak IC column (80: 20 hexane: 2-propanol, 0.8 mL/min, 40 °C, 254 nm); tr (major) = 13.8 min, tr (minor) = 23.2 min, 93% e.e.. <1:20 dr determined by crude NMR.

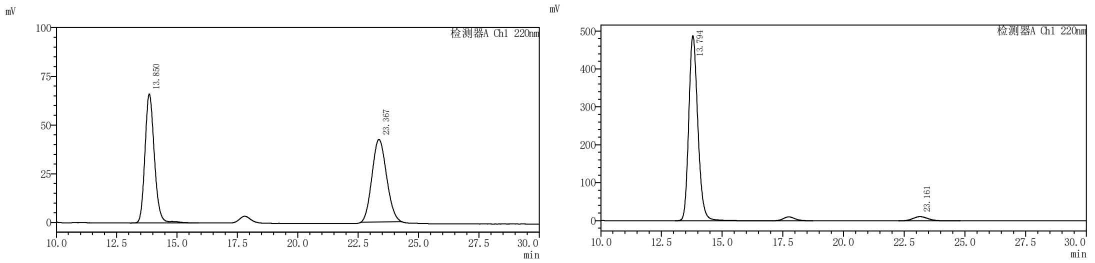


| No. | Time | Area | Area (%) | No. | Time | Area | Area (%) |
|---|---|---|---|---|---|---|---|
| 1 | 13.850 | 1803709 | 50.362 | 1 | 13.794 | 13036308 | 96.545 |
| 2 | 23.367 | 1777796 | 49.638 | 2 | 23.161 | 466591 | 3.455 |

**Supplementary Figure 86. HPLC data of dia-69.**

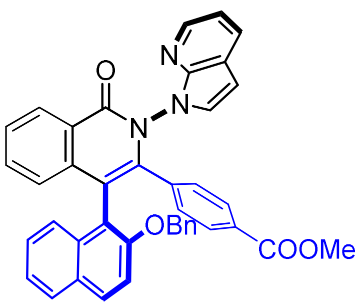

**(*S*, *S*)-methyl 4-(4-(2-(benzyloxy)naphthalen-1-yl)-1-oxo-2-(1H-pyrrolo[2,3-b]pyridin-1-yl)-1,2-dihydroisoquinolin-3-yl)benzoate (70)**

White solid (41.4 mg, 66%), $^1$H NMR (600 MHz, $CDCl_3$) δ 8.57 (dd, *J* = 7.9, 1.6 Hz, 1H), 8.33 (dd, *J* = 4.8, 1.5 Hz, 1H), 7.77 (dd, *J* = 7.8, 1.5 Hz, 1H), 7.74 – 7.71 (m, 1H), 7.69 (d, *J* = 9.1 Hz, 1H), 7.60 (d, *J* = 8.5 Hz, 1H), 7.51 (dtd, *J* = 20.7, 7.3, 1.4 Hz, 2H), 7.41 (ddd, *J* = 8.3, 6.7, 1.3 Hz, 1H), 7.35 – 7.23 (m, 10H), 7.13 – 7.04 (m, 3H), 6.95 (ddd, *J* = 16.3, 8.0, 1.5 Hz, 2H), 6.34 (d, *J* = 3.8 Hz, 1H), 5.07 (s, 2H), 3.68 (s, 3H).

$^{13}$C NMR (151 MHz, $CDCl_3$) δ 166.39, 161.44, 154.15, 146.88, 144.20, 142.02, 137.40, 137.07, 137.04, 134.28, 133.42, 130.25, 129.61, 129.47, 128.77, 128.70, 128.53, 128.35, 128.23, 128.11, 127.87, 127.81, 127.53, 127.46, 127.04, 126.49, 125.79, 125.62, 124.66, 123.72, 118.52, 117.85, 117.25, 114.40, 113.13, 100.90, 70.24, 51.93.

**HRMS** (ESI-TOF) (m/z): Calcd for $C_{41}H_{30}N_3O_4^+$, ([M + H]$^+$), 628.2231, found 628.2221. **$[\alpha]_D^{20}$** = -47 (c = 0.1, $CHCl_3$).

**HPLC** conditions: Daicel Chiralpak IC column (60: 40 hexane: 2-propanol, 0.8 mL/min, 40 °C, 254 nm); tr (major) = 12.0 min, tr (minor) = 18.9 min, 94% e.e.. >20:1 dr determined by crude NMR.

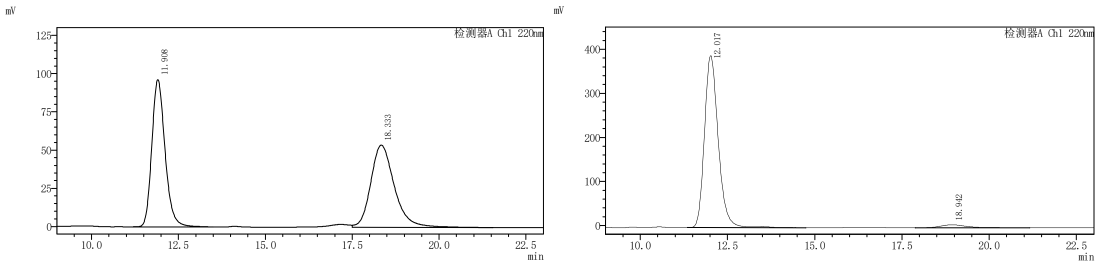


| No. | Time | Area | Area (%) | No. | Time | Area | Area (%) |
|---|---|---|---|---|---|---|---|
| 1 | 11.908 | 2557340 | 50.042 | 1 | 12.017 | 10726828 | 96.947 |
| 2 | 18.333 | 2553003 | 49.958 | 2 | 18.942 | 337795 | 3.053 |

**Supplementary Figure 87. HPLC data of 70.**

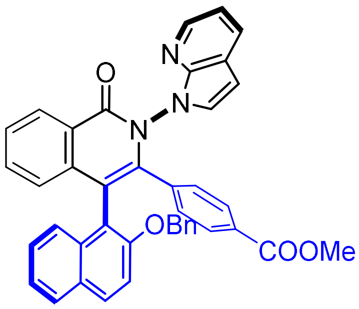

**(*R*, *S*)-methyl 4-(4-(2-(benzyloxy)naphthalen-1-yl)-1-oxo-2-(1H-pyrrolo[2,3-b]pyridin-1-yl)-1,2-dihydroisoquinolin-3-yl)benzoate (*dia*-70)**

White solid (50.2 mg, 80%), $^1$H NMR (600 MHz, $CDCl_3$) δ 8.58 (dd, *J* = 7.9, 1.6 Hz, 1H), 8.32 (dd, *J* = 4.8, 1.5 Hz, 1H), 7.79 (d, *J* = 8.5 Hz, 1H), 7.76 – 7.70 (m, 2H), 7.68 (d, *J* = 8.2 Hz, 1H), 7.49-7.53 (m, 3H), 7.40 (ddd, *J* = 8.4, 6.8, 1.3 Hz, 1H), 7.33 – 7.24 (m, 5H), 7.19 (d, *J* = 9.0 Hz, 1H), 7.16 (dd, *J* = 6.6, 2.8 Hz, 2H), 7.11 – 6.99 (m, 4H), 6.94 (dd, *J* = 8.0, 1.3 Hz, 1H), 6.32 (d, *J* = 3.8 Hz, 1H), 5.13 (s, 2H), 3.66 (s, 3H).

$^{13}$C NMR (151 MHz, $CDCl_3$) δ 166.37, 161.51, 154.21, 146.75, 144.14, 141.93, 137.46, 137.17, 136.99, 133.93, 133.42, 130.29, 129.58, 129.41, 128.81, 128.72, 128.61, 128.55, 128.39, 127.94, 127.88, 127.81, 127.77, 127.43, 127.12, 126.62, 125.66, 125.08, 123.90, 118.57, 118.23, 117.24, 114.02, 113.10, 100.71, 70.52, 51.90.

**HRMS** (ESI-TOF) (m/z): Calcd for $C_{41}H_{30}N_3O_4^+$, ([M + H]$^+$), 628.2231, found 628.2219. **[α]$_D^{20}$** = +41 (c = 0.1, $CHCl_3$).

**HPLC** conditions: Daicel Chiralpak OD-3 column (60: 40 hexane: 2-propanol, 0.8 mL/min, 40 °C, 254 nm); tr (major) = 7.4 min, tr (minor) = 18.6 min, 93% e.e.. <1:20 dr determined by crude NMR.

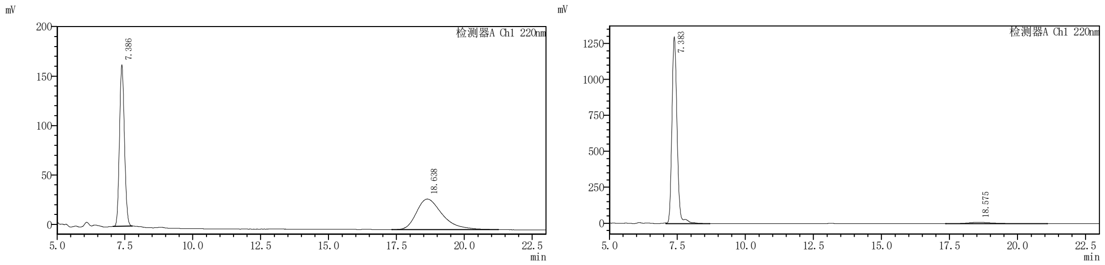


| No. | Time | Area | Area (%) | No. | Time | Area | Area (%) |
|---|---|---|---|---|---|---|---|
| 1 | 7.386 | 2078178 | 50.394 | 1 | 7.383 | 17084758 | 96.376 |
| 2 | 18.638 | 2045650 | 49.606 | 2 | 18.575 | 642456 | 3.624 |

**Supplementary Figure 88. HPLC data of dia-70.**

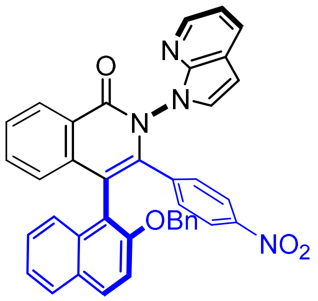


**(*S,S*)-4-(2-(benzyloxy)naphthalen-1-yl)-3-(4-nitrophenyl)-2-(1H-pyrrolo[2,3-b]pyridin-1-yl)isoquinolin-1(2H)-one (71)**

Yellow solid (31.9 mg, 52%), $^1$H NMR (600 MHz, $CDCl_3$) δ 8.50 (dd, *J* = 8.0, 1.7 Hz, 1H), 8.24 (dd, *J* = 4.8, 1.6 Hz, 1H), 7.67-7.72 (m, 3H), 7.55 – 7.31 (m, 7H), 7.29 – 7.16 (m, 6H), 7.10 – 7.03 (m, 2H), 6.99 (ddd, *J* = 13.1, 8.1, 3.3 Hz, 2H), 6.88 – 6.83 (m, 1H), 6.31 (d, *J* = 3.7 Hz, 1H), 5.03 (s, 2H).

$^{13}$C NMR (151 MHz, $CDCl_3$) δ 160.25, 153.09, 146.23, 145.95, 143.44, 139.76, 137.98, 136.11, 135.83, 133.12, 132.55, 129.59, 129.02, 128.50, 127.93, 127.81, 127.73, 127.59, 127.38, 127.16, 126.78, 126.68, 126.28, 125.58, 124.84, 124.67, 123.38, 122.91, 120.79, 120.67, 117.46, 116.47, 113.39, 112.44, 100.27, 69.31.

**HRMS** (ESI-TOF) (m/z): Calcd for $C_{39}H_{26}N_4O_4^+$, ([M + H]$^+$), 615.2027, found 615.2023. **[α]$_D^{20}$** = -22 (c = 0.1, $CHCl_3$).

**HPLC** conditions: Daicel Chiralpak IC column (80: 20 hexane: 2-propanol, 0.8 mL/min, 40 °C, 254 nm); tr (major) = 24.1 min, tr (minor) = 32.2 min, 94% e.e.. >20:1 dr determined by crude NMR.

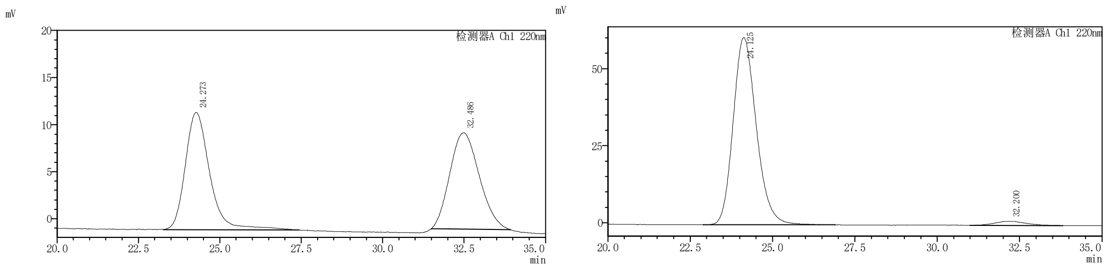


| No. | Time | Area | Area (%) | No. | Time | Area | Area (%) |
|---|---|---|---|---|---|---|---|
| 1 | 24.273 | 652161 | 50.243 | 1 | 24.125 | 2976855 | 97.138 |
| 2 | 32.486 | 645862 | 49.757 | 2 | 32.200 | 87718 | 2.862 |

**Supplementary Figure 89. HPLC data of 71.**

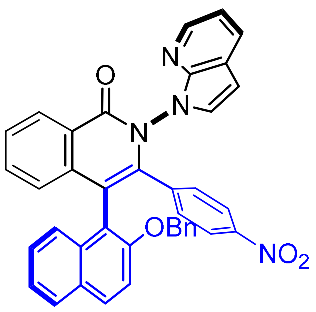

**(*R*,*S*)-4-(2-(benzyloxy)naphthalen-1-yl)-3-(4-nitrophenyl)-2-(1H-pyrrolo[2,3-b]pyridin-1-yl)isoquinolin-1(2H)-one (*dia*-71)**

White solid (35.6 mg, 58%)$^{1}$H NMR (600 MHz, $CDCl_3$) δ 8.58 (dd, *J* = 8.0, 1.6 Hz, 1H), 8.32 (dd, *J* = 4.8, 1.6 Hz, 1H), 7.85 – 7.74 (m, 3H), 7.71 (d, *J* = 8.2 Hz, 1H), 7.67 (dd, *J* = 8.5, 2.4 Hz, 1H), 7.56 (t, *J* = 7.6 Hz, 1H), 7.51 (td, *J* = 7.6, 7.2, 1.6 Hz, 1H), 7.43 (dd, *J* = 8.5, 6.9 Hz, 1H), 7.36 – 7.24 (m, 6H), 7.22 (d, *J* = 8.9 Hz, 1H), 7.14-7.16 (m, 3H), 7.10 – 7.08 (m, 1H), 7.08 – 7.04 (m, 1H), 6.94 (d, *J* = 8.1 Hz, 1H), 6.40 – 6.33 (m, 1H), 5.20 – 5.03 (m, 2H).

$^{13}$C NMR (151 MHz, $CDCl_3$) δ 161.36, 154.16, 147.22, 146.70, 144.29, 140.68, 138.92, 137.19, 136.97, 133.83, 133.58, 130.64, 129.61, 129.52, 128.84, 128.78, 128.60, 128.47, 128.14, 128.04, 127.79, 127.40, 126.74, 125.78, 124.82, 124.10, 121.76, 121.70, 118.59, 117.76, 117.51, 114.10, 113.46, 101.10, 70.64.

**HRMS** (ESI-TOF) (m/z): Calcd for $C_{39}H_{26}N_4O_4^+$, ([M + H]$^+$), 615.2027, found 615.2015. $[\alpha]_D^{20}$ = +30 (c = 0.1, $CHCl_3$).

**HPLC** conditions: Daicel Chiralpak IC column (80: 20 hexane: 2-propanol, 0.8 mL/min, 40 °C, 254 nm); tr (major) = 19.3 min, tr (minor) = 25.9 min, 91% e.e.. <1:20 dr determined by crude NMR.

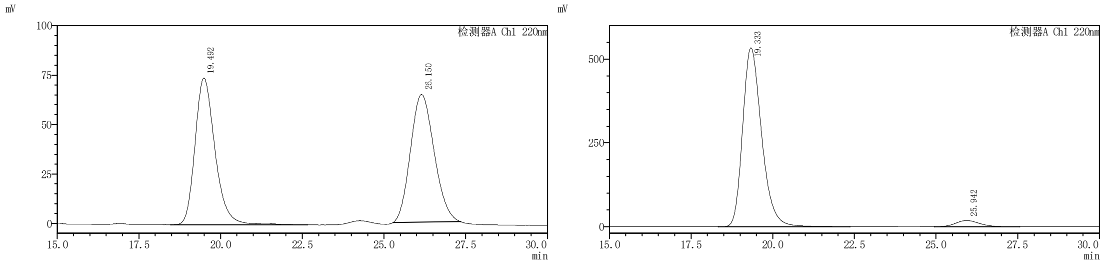


| No. | Time | Area | Area (%) | No. | Time | Area | Area (%) |
|---|---|---|---|---|---|---|---|
| 1 | 19.492 | 3172455 | 49.358 | 1 | 19.333 | 21614476 | 95.748 |
| 2 | 26.150 | 3254954 | 50.642 | 2 | 25.942 | 959909 | 4.252 |

**Supplementary Figure 90. HPLC data of dia-71.**

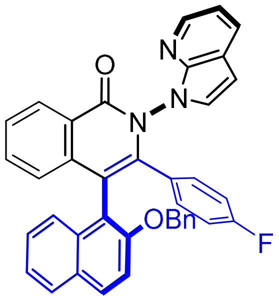

**(*S*,*S*)-4-(2-(benzyloxy)naphthalen-1-yl)-3-(4-fluorophenyl)-2-(1H-pyrrolo[2,3-b]pyridin-1-yl)isoquinolin-1(2H)-one (72)**

White solid (36.4 mg, 62%), $^{1}$H NMR (600 MHz, $CDCl_3$) δ 8.57 (dd, *J* = 7.9, 1.6 Hz, 1H), 8.33 (dt, *J* = 4.8, 1.6 Hz, 1H), 7.79 (dt, *J* = 7.8, 1.5 Hz, 1H), 7.73 (dd, *J* = 15.0, 8.6 Hz, 2H), 7.59 (d, *J* = 8.5 Hz, 1H), 7.55 – 7.45 (m, 2H), 7.40 (tt, *J* = 6.8, 1.4 Hz, 1H), 7.36 – 7.29 (m, 3H), 7.27 – 7.24 (m, 3H), 7.21 (ddd, *J* = 8.7, 5.5, 2.0 Hz, 1H), 7.16 – 7.10 (m, 2H), 7.07 (ddd, *J* = 7.9, 4.7, 1.4 Hz, 1H), 6.97 – 6.89 (m, 1H), 6.85 (ddd, *J* = 8.6, 5.1, 2.0 Hz, 1H), 6.37 (dd, *J* = 3.9, 1.4 Hz, 1H), 6.33 (ddt, *J* = 9.9, 8.5, 1.9 Hz, 1H), 6.27 (tt, *J* = 8.8, 1.9 Hz, 1H), 5.08 (s, 2H).

$^{13}$C NMR (151 MHz, $CDCl_3$) δ 161.54, 161.35 (d, *J* = 246.4 Hz), 154.06, 146.90, 144.17, 142.13, 137.45, 137.13, 134.30, 133.37, 130.65, 130.08 (d, *J* = 9.4 Hz), 129.86 (d, *J* = 8.3 Hz), 129.39, 128.76, 128.53, 128.42, 128.22, 127.52, 127.31, 126.97, 126.50, 125.74, 124.71, 123.70, 118.54, 118.31, 117.17, 114.51, 113.83, 113.71, 113.56, 113.30, 100.74, 70.26.

**HRMS** (ESI-TOF) (m/z): Calcd for $C_{39}H_{27}FN_3O_2^+$, ($[M + H]^+$), 588.2082, found 588.2077. **$[\alpha]_D^{20}$** = -24 (c = 0.1, $CHCl_3$).

**HPLC** conditions: Daicel Chiralpak IC column (80: 20 hexane: 2-propanol, 0.8 mL/min, 40 °C, 254 nm); tr (major) = 14.9 min, tr (minor) = 24.2 min, 95% e.e.. >20:1 dr determined by crude NMR.

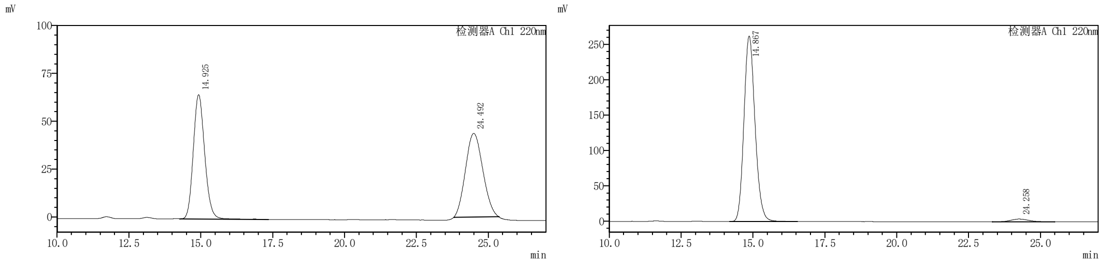


| No. | Time | Area | Area (%) | No. | Time | Area | Area (%) |
|---|---|---|---|---|---|---|---|
| 1 | 14.925 | 1794056 | 49.442 | 1 | 14.867 | 7017651 | 97.576 |
| 2 | 24.492 | 1834540 | 50.558 | 2 | 24.258 | 174348 | 2.424 |

**Supplementary Figure 91. HPLC data of 72.**

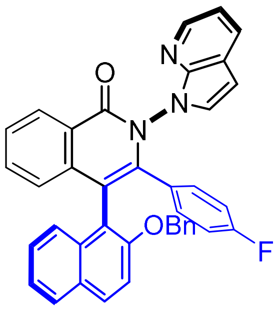


**(*R,S*)-4-(2-(benzyloxy)naphthalen-1-yl)-3-(4-fluorophenyl)-2-(1H-pyrrolo[2,3-b]pyridin-1-yl)isoquinolin-1(2H)-one (*dia*-72)**

White solid (45.2 mg, 77%), $^1$H NMR (600 MHz, $CDCl_3$) δ 8.57 (dt, $J$ = 7.9, 2.1 Hz, 1H), 8.32 (dq, $J$ = 4.3, 2.0 Hz, 1H), 7.83 – 7.67 (m, 4H), 7.55 – 7.44 (m, 2H), 7.39 (ddd, $J$ = 8.3, 6.6, 1.5 Hz, 1H), 7.33 – 7.25 (m, 4H), 7.25 – 7.20 (m, 1H), 7.13-7.17 (m, 3H), 7.08 – 7.01 (m, 2H), 6.91 (ddt, $J$ = 16.7, 8.3, 2.1 Hz, 2H), 6.49 (td, $J$ = 8.7, 2.6 Hz, 1H), 6.34 (dd, $J$ = 4.1, 2.4 Hz, 1H), 6.09 (td, $J$ = 8.7, 2.6 Hz, 1H), 5.13 (t, $J$ = 2.3 Hz, 2H).

$^{13}$C NMR (151 MHz, $CDCl_3$) δ 161.60, 161.30 (d, $J$ = 246.8 Hz), 154.14, 146.74, 144.08, 141.99, 137.50, 137.19, 133.90, 133.36, 130.27, 130.16, 130.12 (d, $J$ = 9.6 Hz), 129.38 (d, $J$ = 8.5 Hz), 128.85, 128.69, 128.65, 128.52, 128.38, 127.91, 127.86, 127.29, 127.03, 126.62, 125.63, 125.61, 125.14, 123.87, 118.67, 117.16, 114.10, 113.58, 113.32, 100.56, 70.54.

**HRMS** (ESI-TOF) (m/z): Calcd for $C_{39}H_{27}FN_3O_2^+$, ($[M + H]^+$), 588.2082, found 588.2081. **$[\alpha]_D^{20}$** = +32 (c = 0.1, $CHCl_3$).

**HPLC** conditions: Daicel Chiralpak IC column (80: 20 hexane: 2-propanol, 0.8 mL/min, 40 °C, 254 nm); tr (major) = 11.6 min, tr (minor) = 18.9 min, 93% e.e.. <1:20 dr determined by crude NMR.

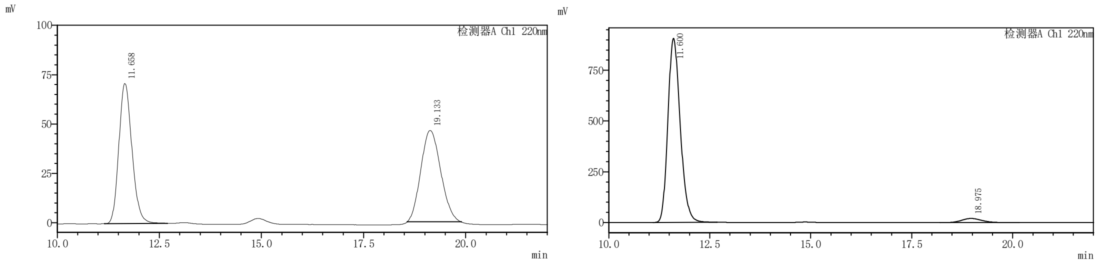


| No. | Time | Area | Area (%) | No. | Time | Area | Area (%) |
|---|---|---|---|---|---|---|---|
| 1 | 11.658 | 1586686 | 50.235 | 1 | 11.600 | 19317250 | 96.439 |
| 2 | 19.133 | 1571839 | 49.765 | 2 | 18.975 | 713263 | 3.561 |

**Supplementary Figure 92. HPLC data of dia-72.**

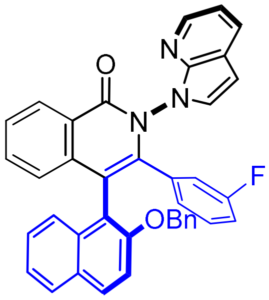

**(*S,S*)-4-(2-(benzyloxy)naphthalen-1-yl)-3-(3-fluorophenyl)-2-(1H-pyrrolo[2,3-b]pyridin-1-yl)isoquinolin-1(2H)-one (73)**

White solid (37.0 mg, 63%), $^1$H NMR (600 MHz, $CDCl_3$) δ 8.57 (dd, *J* = 8.5, 3.7 Hz, 1H), 8.31 (dd, *J* = 9.8, 4.8 Hz, 1H), 7.73-7.76 (m, 3H), 7.58 (dd, *J* = 8.4, 2.8 Hz, 1H), 7.50 (dt, *J* = 21.8, 7.4 Hz, 2H), 7.41 (dt, *J* = 22.3, 7.7 Hz, 1H), 7.36 – 7.09 (m, 9H), 7.05 (ddd, *J* = 12.3, 7.8, 4.8 Hz, 1H), 6.99 – 6.69 (m, 3H), 6.53 (dt, *J* = 35.1, 7.9 Hz, 1H), 6.37 (dd, *J* = 26.5, 3.8 Hz, 1H), 5.12 (dd, *J* = 23.7, 14.2 Hz, 2H).

$^{13}$C NMR (151 MHz, $CDCl_3$) δ 161.48, 153.94, 153.85, 146.85, 144.34, 144.12, 140.11, 137.42, 137.19, 133.97, 133.83, 133.42, 130.28, 129.37, 128.73, 128.62, 128.54, 128.44, 128.38, 127.91, 127.84, 127.43, 126.58, 126.45, 125.66, 125.08, 124.97, 123.80, 118.60, 118.13, 117.32, 116.22, 113.95, 113.29, 100.72, 70.55.

**HRMS** (ESI-TOF) (m/z): Calcd for $C_{39}H_{27}FN_3O_2^+$, ([M + H]$^+$), 588.2082, found 588.2083. $[\alpha]_D^{20}$ = -42 (c = 0.1, $CHCl_3$).

**HPLC** conditions: Daicel Chiralpak IC column (80: 20 hexane: 2-propanol, 0.8 mL/min, 40 °C, 254 nm); tr (major) = 14.5 min, tr (minor) = 22.7 min, 96% e.e.. >20:1 dr determined by crude NMR.

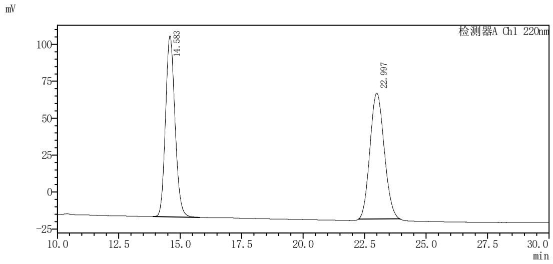


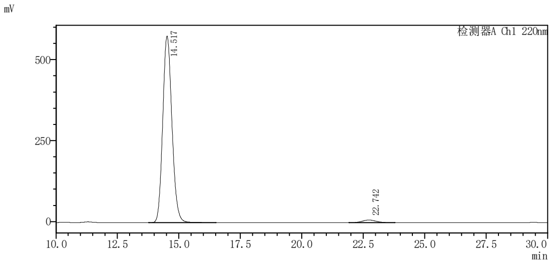


| No. | Time | Area | Area (%) | No. | Time | Area | Area (%) |
|---|---|---|---|---|---|---|---|
| 1 | 14.583 | 3291023 | 48.859 | 1 | 14.517 | 15009559 | 97.866 |
| 2 | 22.997 | 3444780 | 51.141 | 2 | 22.742 | 327237 | 2.134 |

**Supplementary Figure 93. HPLC data of 73.**

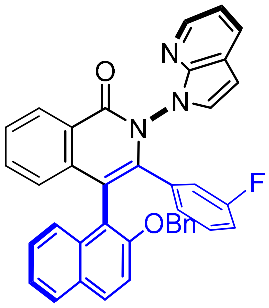

**(*R,S*)-4-(2-(benzyloxy)naphthalen-1-yl)-3-(3-fluorophenyl)-2-(1H-pyrrolo[2,3-b]pyridin-1-yl)isoquinolin-1(2H)-one (*dia*-73)**

White solid (44.6 mg, 76%), $^1$H NMR (600 MHz, $CDCl_3$) δ 8.58 (t, *J* = 6.8 Hz, 1H), 8.35 (dd, *J* = 35.5, 4.8 Hz, 1H), 7.85 – 7.65 (m, 4H), 7.57 – 7.45 (m, 2H), 7.41 (dt, *J* = 21.3, 7.7 Hz, 1H), 7.36 – 7.18 (m, 7H), 7.17 – 7.11 (m, 1H), 7.10 – 6.91 (m, 4H), 6.85 – 6.65 (m, 2H), 6.37 – 6.31 (m, 1H), 5.21 – 5.12 (m, 2H).

$^{13}$C NMR (151 MHz, $CDCl_3$) δ 161.47, 154.34, 146.82, 144.39, 144.08, 141.42, 137.42, 137.19, 133.97, 133.39, 132.18, 130.27, 129.36, 128.71, 128.67, 128.60, 128.52, 128.44, 128.36, 127.89, 127.83, 127.41, 127.02, 126.58, 126.45, 125.69, 125.65, 125.08, 123.80, 118.61, 118.16, 117.16, 113.97, 113.30, 100.70, 70.57.

**HRMS** (ESI-TOF) (m/z): Calcd for $C_{39}H_{27}FN_3O_2^+$, ([M + H]$^+$), 588.2082, found 588.2077. $[\alpha]_D^{20}$ = +50 (c = 0.1, $CHCl_3$).

**HPLC** conditions: Daicel Chiralpak IC column (80: 20 hexane: 2-propanol, 0.8 mL/min, 40 °C, 254 nm); tr (major) = 11.3 min, tr (minor) = 16.9 min, 92% e.e.. <1:20 dr determined by crude NMR.

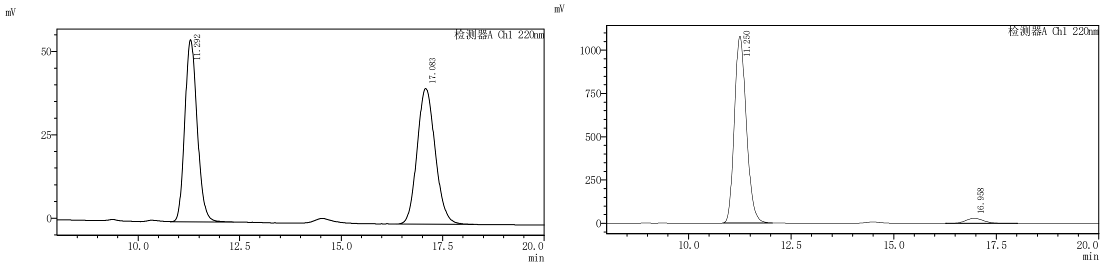


| No. | Time | Area | Area (%) | No. | Time | Area | Area (%) |
|---|---|---|---|---|---|---|---|
| 1 | 11.292 | 1165552 | 47.970 | 1 | 11.250 | 21890197 | 96.116 |
| 2 | 17.083 | 1264216 | 52.030 | 2 | 16.958 | 884504 | 3.884 |

**Supplementary Figure 94. HPLC data of dia-73.**

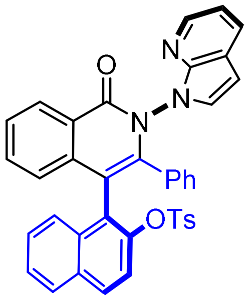


**(*S, S*)-3-phenyl-2-(1H-pyrrolo[2,3-b]pyridin-1-yl)-4-(2-tosylnaphthalen-1-yl)isoquinolin-1(2H)-one (74)**

White solid (15.2 mg, 24%), $^{1}H$ NMR (600 MHz, $CDCl_3$) δ 8.42 (dd, *J* = 4.7, 1.5 Hz, 1H), 8.25 (ddd, *J* = 8.1, 1.5, 0.6 Hz, 1H), 7.86 – 7.64 (m, 5H), 7.46 – 7.27 (m, 6H), 7.16 (ddd, *J* = 8.4, 7.2, 1.5 Hz, 1H), 7.10 (dd, *J* = 7.8, 4.7 Hz, 1H), 6.89 – 6.81 (m, 3H), 6.79 (tt, *J* = 7.3, 1.5 Hz, 1H), 6.73 – 6.68 (m, 1H), 6.63 – 6.54 (m, 2H), 6.40 (dt, *J* = 8.2, 0.9 Hz, 1H), 6.25 (d, *J* = 3.8 Hz, 1H), 2.10 (s, 3H).

$^{13}C$ NMR (151 MHz, $CDCl_3$) δ 160.01, 146.38, 144.77, 143.36, 143.28, 142.99, 135.68, 132.51, 132.25, 131.72, 130.83, 130.61, 129.13, 128.67, 128.33, 127.65, 127.53, 127.39, 127.19, 126.78, 126.58, 126.17, 125.71, 125.53, 125.33, 125.30, 125.04, 123.98, 123.33, 121.83, 117.53, 116.35, 109.43, 99.70, 20.67.

**HRMS** (ESI-TOF) (m/z): Calcd for $C_{39}H_{28}N_3O_4S^+$, ($[M + H]^+$), 634.1795,found 634.1798. $[\alpha]_D^{20}$ = -22 (c = 0.1, $CHCl_3$).

**HPLC** conditions: Daicel Chiralpak OD-3 column (80: 20 hexane: 2-propanol, 1.0 mL/min, 40 °C, 254 nm); tr (major) = 6.8 min, tr (minor) = 7.5 min, 94% e.e.. >20:1 dr determined by crude NMR.

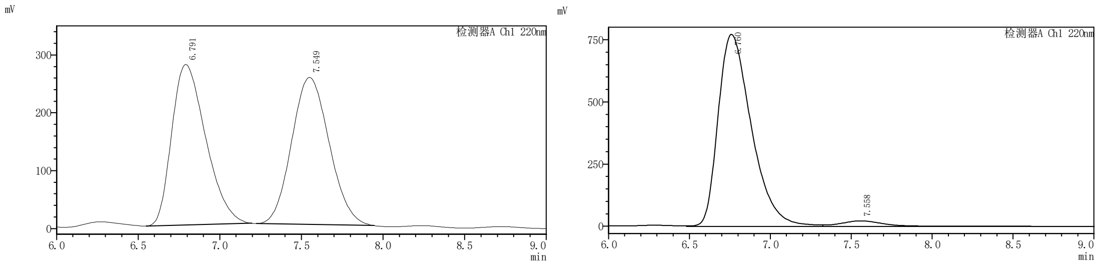


| No. | Time | Area | Area (%) | No. | Time | Area | Area (%) |
|---|---|---|---|---|---|---|---|
| 1 | 6.791 | 4018318 | 49.644 | 1 | 6.760 | 13333822 | 96.984 |
| 2 | 7.549 | 4076012 | 50.356 | 2 | 7.558 | 414654 | 3.016 |

**Supplementary Figure 95. HPLC data of 74.**

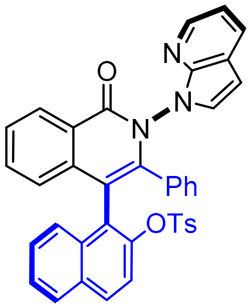


**(*R,S*)-3-phenyl-2-(1H-pyrrolo[2,3-b]pyridin-1-yl)-4-(2-tosylnaphthalen-1-yl)isoquinolin-1(2H)-one (*dia*-74)**

White solid (50.0 mg, 79%), $^{1}$H NMR (600 MHz, $CDCl_3$) δ 8.40 (ddd, *J* = 8.1, 1.5, 0.6 Hz, 1H), 8.17 – 8.11 (m, 1H), 7.75 – 7.68 (m, 3H), 7.64 (dd, *J* = 7.8, 1.5 Hz, 1H), 7.45 – 7.26 (m, 8H), 7.01 (d, *J* = 3.8 Hz, 1H), 6.98 – 6.93 (m, 2H), 6.93 – 6.84 (m, 2H), 6.72 – 6.63 (m, 2H), 6.58 (dt, *J* = 8.2, 0.9 Hz, 1H), 6.36 – 6.27 (m, 2H), 2.30 (s, 3H).

$^{13}$C NMR (151 MHz, $CDCl_3$) δ 160.17, 145.28, 144.80, 144.12, 142.26, 135.97, 132.66, 132.55, 132.10, 130.52, 129.25, 128.52, 128.18, 127.76, 127.47, 127.41, 127.33, 127.20, 127.10, 126.68, 126.42, 126.24, 125.91, 125.66, 125.42, 125.30, 125.18, 124.10, 123.73, 119.89, 117.43, 116.06, 109.59, 99.45, 20.67.

**HRMS** (ESI-TOF) (m/z): Calcd for $C_{39}H_{28}N_3O_4S^{+}$, ($[M + H]^{+}$), 634.1795, found 634.1798. $[\alpha]_D^{20}$ = +42 (c = 0.1, $CHCl_3$).

**HPLC** conditions: Daicel Chiralpak IC column (80: 20 hexane: 2-propanol, 1.0 mL/min, 40 °C, 254 nm); tr (major) = 9.6 min, tr (minor) = 12.0 min, 88% e.e.. dr = 1:7 determined by crude NMR.

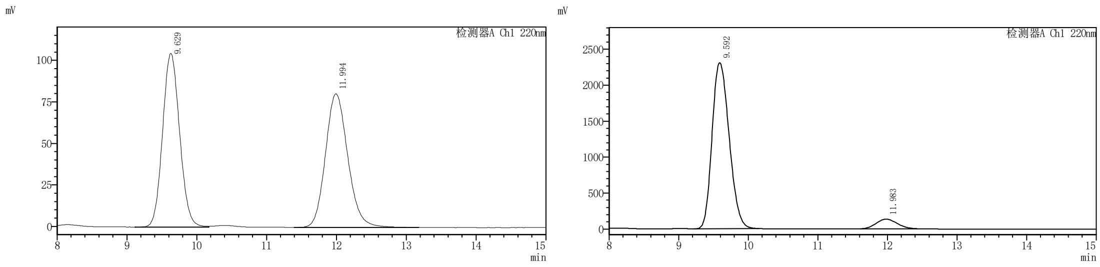


| No. | Time | Area | Area (%) | No. | Time | Area | Area (%) |
|---|---|---|---|---|---|---|---|
| 1 | 9.629 | 1776223 | 49.795 | 1 | 9.592 | 39429992 | 93.421 |
| 2 | 11.994 | 1790832 | 50.205 | 2 | 11.983 | 2776967 | 6.579 |

**Supplementary Figure 96. HPLC data of dia-74.**

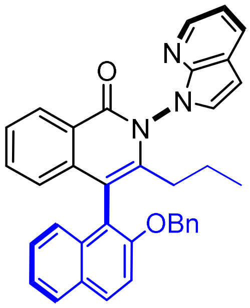


**(*R,S*)-4-(2-(benzyloxy)naphthalen-1-yl)-3-propyl-2-(1H-pyrrolo[2,3-b]pyridin-1-yl)isoquinolin-1(2H)-one (*dia*-75)**

White solid (34.8 mg, 65%), $^{1}$H NMR (600 MHz, $CDCl_3$) δ 8.48 – 8.41 (m, 1H), 8.41 – 8.32 (m, 1H), 8.01 – 7.93 (m, 2H), 7.90 – 7.83 (m, 1H), 7.78 (d, *J* = 8.4 Hz, 1H), 7.47 – 7.36 (m, 5H), 7.31 (d, *J* = 3.8 Hz, 1H), 7.23-7.26 (m, 3H), 7.18 – 7.07 (m, 3H), 6.85 – 6.76 (m, 1H), 6.68 (d, *J* = 3.8 Hz, 1H), 5.16 (q, *J* = 12.2 Hz, 2H), 2.14 – 2.02 (m, 2H), 1.36 – 1.27 (m, 1H), 1.22 – 1.14 (m, 1H), 0.28 (t, *J* = 7.4 Hz, 3H).

$^{13}$C NMR (151 MHz, $CDCl_3$) δ 162.11, 154.43, 147.27, 144.50, 144.02, 137.84, 137.18, 134.15, 133.07, 130.19, 129.61, 129.58, 129.34, 128.43, 128.01, 127.77, 127.26, 126.69, 126.37, 125.33, 125.09, 124.91, 124.16, 119.26, 119.05, 117.48, 114.66, 111.30, 111.28, 100.95, 70.67, 32.61, 22.12, 13.45.

**HRMS** (ESI-TOF) (m/z): Calcd for $C_{36}H_{30}N_3O_2^{+}$, ($[M + H]^{+}$), 536.2333, found 536.2347. $[\alpha]_D^{20}$ = +33 (c = 0.1, $CHCl_3$).

**HPLC** conditions: Daicel Chiralpak IC column (80: 20 hexane: 2-propanol, 1.0 mL/min, 40 °C, 254 nm); tr (major) = 12.2 min, tr (minor) = 19.0 min, 95% e.e.. <1:20 dr determined by crude NMR.

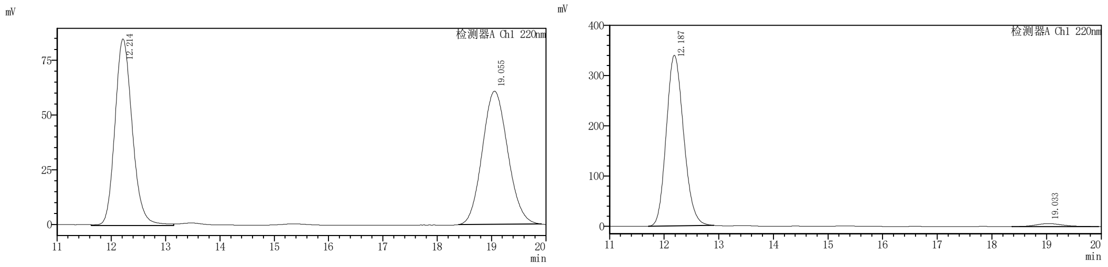

| No. | Time | Area | Area (%) | No. | Time | Area | Area (%) |
| --- | --- | --- | --- | --- | --- | --- | --- |
| 1 | 12.214 | 1892972 | 48.856 | 1 | 12.187 | 7804580 | 97.573 |
| 2 | 19.055 | 1981624 | 51.144 | 2 | 19.033 | 194104 | 2.427 |

**Supplementary Figure 97. HPLC data of dia-75.**

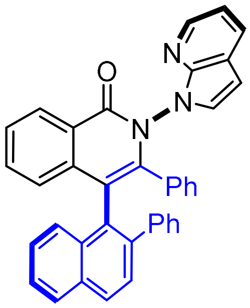


**(*R,S*)-3-phenyl-4-(2-phenylnaphthalen-1-yl)-2-(1H-pyrrolo[2,3-b]pyridin-1-yl)isoquinolin-1(2H)-one (*dia*-76)**

White solid (32.4 mg, 60%), $^1$H NMR (600 MHz, $CDCl_3$) δ 8.51 (dd, $J$ = 7.4, 2.0 Hz, 1H), 8.07 (dd, $J$ = 4.9, 1.5 Hz, 1H), 7.96 (d, $J$ = 8.3 Hz, 1H), 7.81 – 7.70 (m, 2H), 7.56 (dd, $J$ = 7.8, 1.5 Hz, 1H), 7.52 – 7.39 (m, 4H), 7.30 (d, $J$ = 8.5 Hz, 1H), 7.25 – 7.19 (m, 1H), 7.13 (q, $J$ = 7.6, 6.9 Hz, 2H), 7.04 (dd, $J$ = 7.3, 1.8 Hz, 1H), 6.95 (d, $J$ = 3.9 Hz, 1H), 6.88 – 6.78 (m, 3H), 6.64 – 6.52 (m, 3H), 6.28-6.21 (m, 2H), 5.93 – 5.82 (m, 1H).

$^{13}$C NMR (151 MHz, $CDCl_3$) δ 160.82, 142.54, 141.91, 140.34, 138.92, 138.32, 133.03, 132.74, 131.41, 130.32, 129.28, 128.64, 128.35, 128.12, 127.86, 127.73, 127.54, 127.38, 127.25, 126.99, 126.96, 126.64, 126.30, 126.25, 125.99, 125.94, 125.54, 125.17, 124.87, 123.92, 117.41, 115.90, 114.56, 99.00.

**HRMS** (ESI-TOF) (m/z): Calcd for $C_{38}H_{26}N_3O^+$, ($[M + H]^+$), 540.2070, found 540.2077. **$[\alpha]_D^{20}$** = +36 (c = 0.1, $CHCl_3$).

**HPLC** conditions: Daicel Chiralpak IC column (80: 20 hexane: 2-propanol, 0.8 mL/min, 40 °C, 254 nm); tr (major) = 10.8 min, tr (minor) = 14.8 min, 87% e.e.. dr = 1:11 determined by crude NMR.

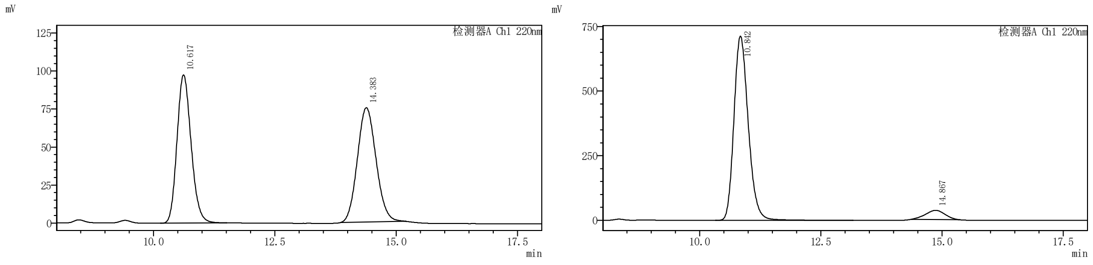

| No. | Time | Area | Area (%) | No. | Time | Area | Area (%) |
| --- | --- | --- | --- | --- | --- | --- | --- |
| 1 | 10.617 | 97603 | 49.488 | 1 | 10.842 | 14676126 | 93.663 |
| 2 | 14.383 | 75344 | 50.512 | 2 | 14.867 | 992871 | 6.337 |

**Supplementary Figure 98. HPLC data of dia-76.**

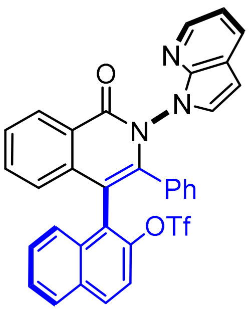


**(*R,S*)-1-(1-oxo-3-phenyl-2-(1H-pyrrolo[2,3-b]pyridin-1-yl)-1,2-dihydroisoquinolin-4-yl)naphthalen-2-yl trifluoromethanesulfonate *(dia*-77)**

White solid (44.0 mg, 72%), $^{1}$H NMR (600 MHz, $CDCl_3$) δ 8.61 – 8.53 (m, 1H), 8.23 (dd, *J* = 4.8, 1.5 Hz, 1H), 8.03 – 7.91 (m, 2H), 7.91 – 7.81 (m, 2H), 7.73 (dd, *J* = 7.8, 1.5 Hz, 1H), 7.62 – 7.49 (m, 4H), 7.40 (p, *J* = 3.7, 3.2 Hz, 1H), 7.36 – 7.30 (m, 2H), 7.03 – 6.93 (m, 2H), 6.91 – 6.86 (m, 1H), 6.84 – 6.75 (m, 2H), 6.46 – 6.39 (m, 2H).

$^{13}$C NMR (151 MHz, $CDCl_3$) δ 162.30, 145.91, 145.28, 144.53, 143.87, 136.62, 133.88, 133.34, 132.09, 131.48, 131.07, 129.51, 129.26, 129.23, 128.76, 128.61, 128.55, 128.35, 128.27, 128.17, 127.71, 127.66, 127.15, 127.05, 126.77, 126.64, 126.16, 126.07, 125.35, 119.01, 118.59, 117.13, 109.84, 100.58.

**HRMS** (ESI-TOF) (m/z): Calcd for $C_{30}H_{21}F_3N_3O_4S^+$, ($[M + H]^+$), 612.1199, found 612.1197. $[\alpha]_D^{20}$ = +30 (c = 0.1, $CHCl_3$).

**HPLC** conditions: Daicel Chiralpak IC column (80: 20 hexane: 2-propanol, 0.8 mL/min, 40 °C, 254 nm); tr (major) = 7.7 min, tr (minor) = 10.2 min, 91% e.e.. <1:20 dr determined by crude NMR.

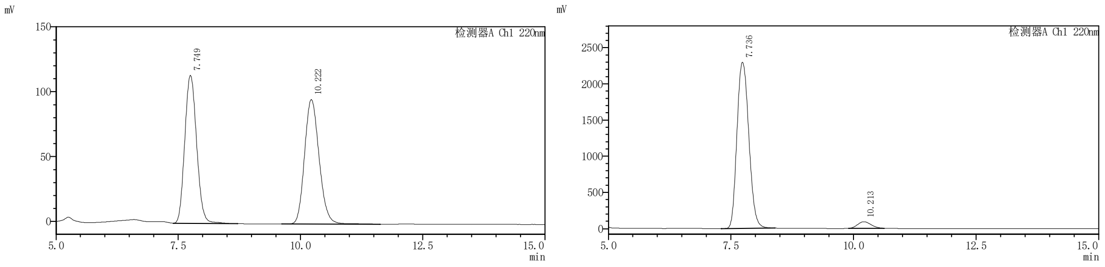


| No. | Time | Area | Area (%) | No. | Time | Area | Area (%) |
|---|---|---|---|---|---|---|---|
| 1 | 7.749 | 1931972 | 49.159 | 1 | 7.736 | 38800552 | 95.765 |
| 2 | 10.222 | 1998061 | 50.841 | 2 | 10.213 | 1715773 | 4.235 |

**Supplementary Figure 99. HPLC data of dia-77.**

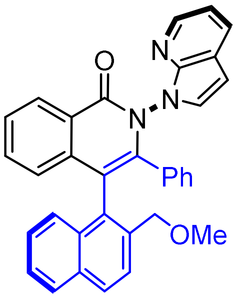


**(*R,S*)-4-(2-(methoxymethyl)naphthalen-1-yl)-3-phenyl-2-(1H-pyrrolo[2,3-b]pyridin-1-yl)isoquinolin-1(2H)-one *(dia*-78)**

White solid (29.4 mg, 58%), $^{1}$H NMR (600 MHz, $CDCl_3$) δ 8.50 (dd, *J* = 7.8, 1.7 Hz, 1H), 8.23 (dd, *J* = 4.8, 1.6 Hz, 1H), 7.90 (d, *J* = 8.4 Hz, 1H), 7.75 – 7.63 (m, 3H), 7.49 – 7.29 (m, 5H), 7.22 – 7.15 (m, 1H), 7.07 (d, *J* = 7.7 Hz, 1H), 6.95 (dd, *J* = 7.8, 4.8 Hz, 1H), 6.77-6.80 (m, 3H), 6.65 (td, *J* = 7.5, 1.3 Hz, 1H), 6.30 (dd, *J* = 10.4, 5.7 Hz, 2H), 4.40 (d, *J* = 12.3 Hz, 1H), 4.30 (d, *J* = 12.3 Hz, 1H), 3.20 (s, 3H).

$^{13}$C NMR (151 MHz, $CDCl_3$) δ 161.67, 147.13, 143.87, 142.81, 137.25, 135.45, 133.62, 133.23, 132.82, 131.76, 130.52, 129.41, 128.97, 128.77, 128.67, 128.49, 128.36, 127.97, 127.57, 126.66, 126.64, 126.60, 125.82, 125.80, 125.46, 125.02, 118.62, 117.14, 114.39, 101.22, 72.64, 57.87.

**HRMS** (ESI-TOF) (m/z): Calcd for $C_{34}H_{26}N_3O_2^+$, ($[M + H]^+$), 508.2020, found 508.2025. $[\alpha]_D^{20}$ = +27 (c = 0.1, $CHCl_3$).

**HPLC** conditions: Daicel Chiralpak IC column (80: 20 hexane: 2-propanol, 0.8 mL/min, 40 °C, 254 nm); tr (major) = 11.3 min, tr (minor) = 15.7 min, 92% e.e.. <1:20 dr determined by crude NMR.

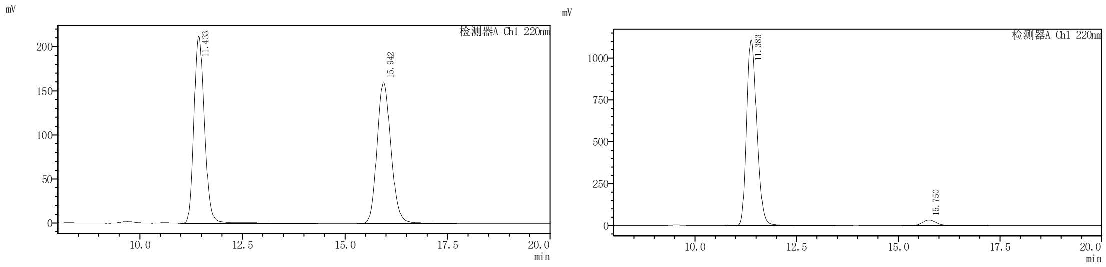

| No. | Time | Area | Area (%) | No. | Time | Area | Area (%) |
|---|---|---|---|---|---|---|---|
| 1 | 11.433 | 3809452 | 50.151 | 1 | 11.383 | 19422053 | 96.012 |
| 2 | 15.942 | 3786454 | 49.849 | 2 | 15.750 | 806749 | 3.988 |

**Supplementary Figure 100. HPLC data of dia-78.**

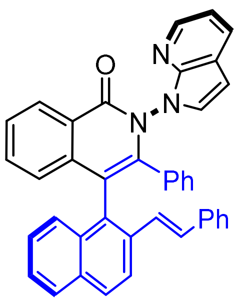

**(*R,S*)-(E)-3-phenyl-2-(1H-pyrrolo[2,3-b]pyridin-1-yl)-4-(2-styrylnaphthalen-1-yl)isoquinolin-1(2H)-one *(dia*-79)**

White solid (35.6 mg, 63%), $^1$H NMR (600 MHz, $CDCl_3$) δ 8.62 (dd, *J* = 8.0, 1.6 Hz, 1H), 8.34 (dd, *J* = 4.8, 1.6 Hz, 1H), 7.92 (d, *J* = 8.4 Hz, 1H), 7.81 – 7.66 (m, 4H), 7.51 (dtd, *J* = 23.8, 7.2, 1.4 Hz, 2H), 7.42 (ddd, *J* = 8.4, 6.8, 1.5 Hz, 1H), 7.40 – 7.18 (m, 7H), 7.14 (d, *J* = 3.9 Hz, 1H), 7.10 (d, *J* = 16.3 Hz, 1H), 7.08 – 7.01 (m, 2H), 6.94 (dd, *J* = 8.0, 1.4 Hz, 1H), 6.86 (d, *J* = 8.0 Hz, 1H), 6.77 (t, *J* = 7.6 Hz, 1H), 6.69 (td, *J* = 7.5, 1.3 Hz, 1H), 6.36 (t, *J* = 7.7 Hz, 1H), 6.34 – 6.30 (m, 1H).

$^{13}$C NMR (151 MHz, $CDCl_3$) δ 161.71, 146.72, 144.08, 143.20, 137.40, 137.30, 134.62, 133.80, 133.45, 132.81, 131.80, 131.16, 130.52, 129.33, 128.86, 128.66, 128.62, 128.28, 128.20, 128.09, 127.97, 127.88, 127.62, 126.94, 126.80, 126.67, 126.52, 126.49, 126.05, 125.91, 125.37, 122.81, 118.47, 117.13, 114.74, 100.51.

**HRMS** (ESI-TOF) (m/z): Calcd for $C_{40}H_{28}N_3O^+$, ([M + H]$^+$), 566.2227, found 566.2233. **[α]$_D$$^{20}$** = +47 (c = 0.1, $CHCl_3$).

**HPLC** conditions: Daicel Chiralpak IC column (80: 20 hexane: 2-propanol, 0.8 mL/min, 40 °C, 254 nm); tr (major) = 10.2 min, tr (minor) = 16.6 min, 96% e.e.. <1:20 dr determined by crude NMR.

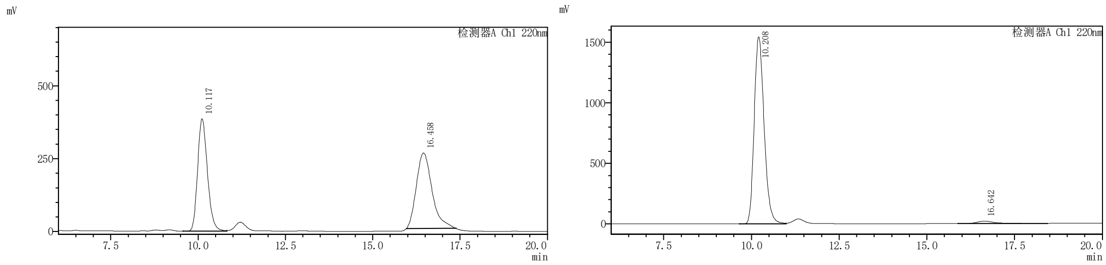

| No. | Time | Area | Area (%) | No. | Time | Area | Area (%) |
|---|---|---|---|---|---|---|---|
| 1 | 10.117 | 7578393 | 48.356 | 1 | 10.208 | 30342898 | 97.763 |
| 2 | 16.458 | 8093714 | 51.644 | 2 | 16.642 | 694248 | 2.237 |

**Supplementary Figure 101. HPLC data of dia-79.**

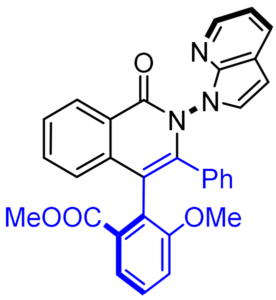


**(*R*,*S*)-methyl-3-methoxy-2-(1-oxo-3-phenyl-2-(1H-pyrrolo[2,3-b]pyridin-1-yl)-1,2-dihydroisoquinolin-4-yl)benzoate *(dia*-80)**

White solid (37.1 mg, 74%), $^1$H NMR (600 MHz, $CDCl_3$) δ 8.50 (dd, *J* = 8.0, 1.5 Hz, 1H), 8.33 (dd, *J* = 4.8, 1.5 Hz, 1H), 7.75 (dt, *J* = 7.8, 1.4 Hz, 1H), 7.56 (ddt, *J* = 8.4, 7.1, 1.4 Hz, 1H), 7.51 – 7.41 (m, 2H), 7.29 – 7.23 (m, 1H), 7.16 – 7.08 (m, 3H), 7.07 – 6.98 (m, 2H), 6.93 – 6.83 (m, 3H), 6.68 (td, *J* = 7.3, 1.7 Hz, 1H), 6.33 (dd, *J* = 3.8, 1.3 Hz, 1H), 3.66 (s, 3H), 3.64 (s, 3H).

$^{13}$C NMR (151 MHz, $CDCl_3$) δ 167.14, 161.38, 157.87, 146.86, 143.94, 140.66, 137.54, 133.06, 132.67, 129.24, 129.15, 128.98, 128.70, 128.63, 128.24, 126.80, 126.65, 126.47, 125.46, 125.32, 124.52, 121.94, 118.59, 116.95, 114.28, 113.88, 100.36, 55.76, 52.03.

**HRMS** (ESI-TOF) (m/z): Calcd for $C_3H_{24}N_3O_4^+$, ($[M + H]^+$), 502.1761, found 502.1780. **$[\alpha]_D^{20}$** = +21 (c = 0.1, $CHCl_3$).

**HPLC** conditions: Daicel Chiralpak IC column (70: 30 hexane: 2-propanol, 0.8 mL/min, 40 °C, 254 nm); tr (major) = 22.6 min, tr (minor) = 38.7 min, 88% e.e.. <1:20 dr determined by crude NMR.

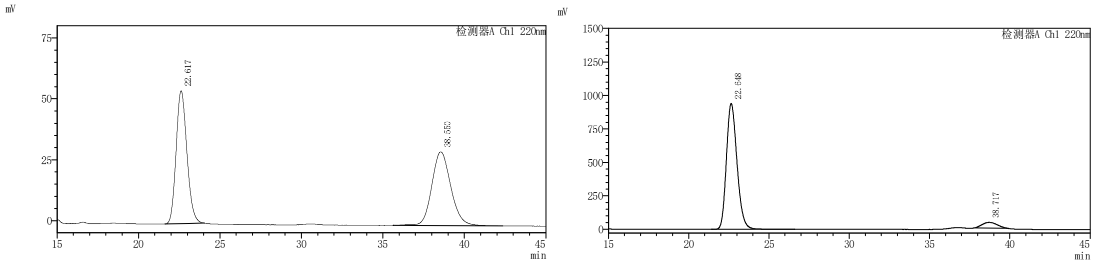


| No. | Time | Area | Area (%) | No. | Time | Area | Area (%) |
|---|---|---|---|---|---|---|---|
| 1 | 22.617 | 2512384 | 50.499 | 1 | 22.648 | 43248038 | 93.847 |
| 2 | 38.550 | 2462778 | 49.501 | 2 | 38.717 | 2835540 | 6.153 |

**Supplementary Figure 102. HPLC data of dia-80.**

## 2.3 Reductive Cleavage of the Directing Group toward Enantiodivergence.

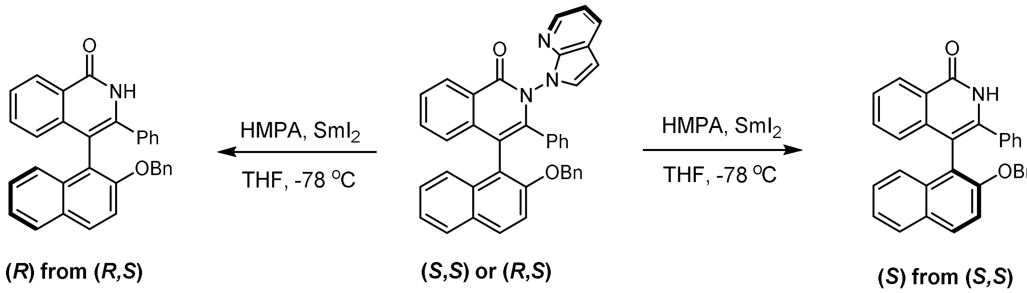


In a $N_2$-filled glove box, A scew-cap vial (8 mL) was charged with the Biaryl Products with Twofold Chiral Axes. ( 0.1 mmol, 1.0 equiv), in THF (1.0 mL). The resulted mixture was stirred at -78 °C, then HMPA (100 μL) was added slowly, then $SmI_2$ was added slowly until the solution turned blue. The reaction was stirred for 30min. After the reaction was complete (monitored by TLC), the solvent was removed under reduced pressure. Then the residue was purified by silica gel column chromatography (PE/EA = 2:1) to afford the desired product as a white solid. Products **82-86** were known compounds and the NMR spectra and HPLC chromatograms agree with the literature report.[3]

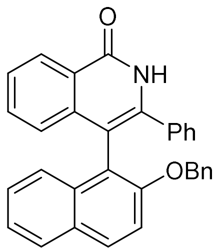


**4-(2-(benzyloxy)naphthalen-1-yl)-3-phenylisoquinolin-1(2H)-one**

***(S)*-82** White solid (39.0 mg, 86%)

***(R)*-82** White solid (39.9 mg, 88%)

$^{1}$H NMR (600 MHz, $CDCl_3$) δ 9.97 (s, 1H), 8.50 (dd, $J$ = 7.9, 1.5 Hz, 1H), 7.84 – 7.70 (m, 2H), 7.51 – 7.37 (m, 3H), 7.34 – 7.28 (m, 2H), 7.23 – 7.11 (m, 7H), 7.07 (t, $J$ = 7.6 Hz, 2H), 6.95 (p, $J$ = 3.8 Hz, 2H), 6.89 (d, $J$ = 8.0 Hz, 1H), 4.99 (d, $J$ = 12.7 Hz, 1H), 4.91 (s, 1H).

$^{13}$C NMR (151 MHz, $CDCl_3$) δ 163.47, 154.52, 139.00, 138.67, 137.15, 135.18, 134.76, 132.69, 129.93, 128.91, 128.66, 128.34, 128.16, 128.13, 128.04, 127.53, 127.45, 126.98, 126.44, 125.61, 125.15, 125.05, 124.29, 123.76, 118.78, 114.73, 110.83, 70.44. **HRMS** (ESI-TOF) (m/z): Calcd for $C_{32}H_{24}NO_2^+$, ([M + H]$^+$), 454.1802, found 454.1804.

**HPLC** conditions: Daicel Chiralpak OD-3 column (80: 20 hexane: 2-propanol, 0.8 mL/min, 40 ºC, 254 nm); ***(S)*-82** tr (major) = 9.1 min, tr (minor) = 12.8 min, 96% e.e.. ***(R)*-82** tr (major) = 12.8 min, tr (minor) = 9.1 min, 88% e.e..

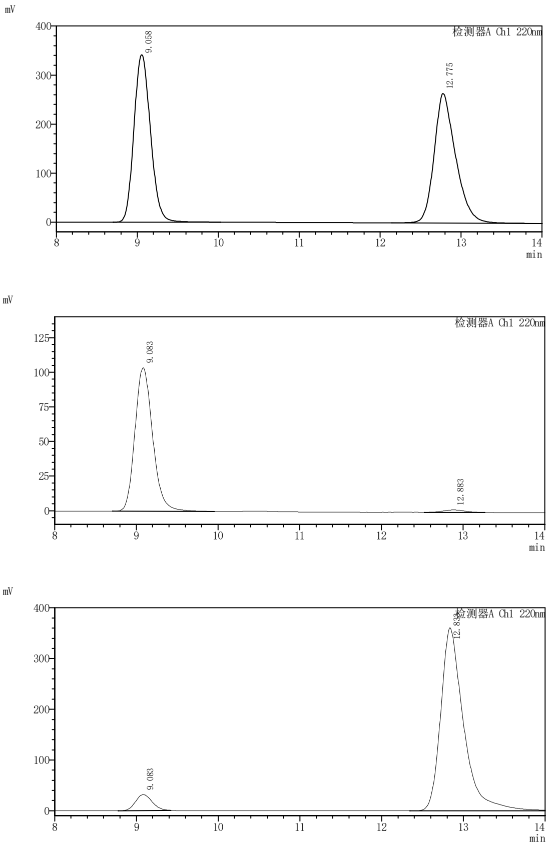


| No. | Time | Area | Area (%) | No. | Time | Area | Area (%) |
|---|---|---|---|---|---|---|---|
| 1 | 9.058 | 4855619 | 49.811 | 1 | 9.083 | 1568758 | 97.926 |
| 2 | 12.775 | 4892425 | 50.189 | 2 | 12.883 | 33228 | 2.074 |
| | | | | 3 | 9.083 | 444041 | 5.936 |
| | | | | 4 | 12.833 | 7036893 | 94.064 |

**Supplementary Figure 103. HPLC data of 82.**

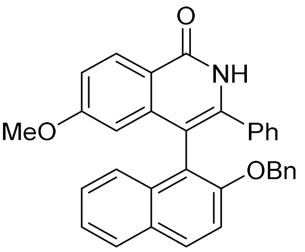


**4-(2-(benzyloxy)naphthalen-1-yl)-6-methoxy-3-phenylisoquinolin-1(2H)-one**

***(S)*-83** White solid (45.9 mg, 95%).

***(R)*-83** White solid (44.5 mg, 92%).

$^1$H NMR (600 MHz, $CDCl_3$) δ 9.03 (s, 1H), 8.45 (d, *J* = 8.9 Hz, 1H), 7.83 – 7.74 (m, 2H), 7.55 – 7.45 (m, 1H), 7.32 (tt, *J* = 6.8, 5.1 Hz, 2H), 7.23 – 7.11 (m, 7H), 7.10 – 7.02 (m, 3H), 6.99 (dd, *J* = 6.4, 2.8 Hz, 2H), 6.23 (d, *J* = 2.5 Hz, 1H), 5.01 (d, *J* = 12.7 Hz, 1H), 4.92 (d, *J* = 12.7 Hz, 1H), 3.49 (s, 3H).

$^{13}$C NMR (151 MHz, $CDCl_3$) δ 162.08, 161.70, 153.41, 140.12, 138.13, 136.15, 134.39, 133.60, 128.96, 128.60, 127.91, 127.68, 127.34, 127.11, 127.09, 126.87, 126.55, 125.96, 125.47, 124.02, 122.75, 118.07, 117.77, 114.07, 113.68, 109.38, 106.66, 69.44, 54.08.

**HRMS** (ESI-TOF) (m/z): Calcd for $C_{33}H_{26}NO_3^+$, ($[M + H]^+$), 484.1907, found 484.1905.

**HPLC** conditions: Daicel Chiralpak IC column (80: 20 hexane: 2-propanol, 0.8 mL/min, 40 °C, 254 nm); ***(S)*-83** tr (major) = 14.6 min, tr (minor) = 13.5 min, 92% e.e.. ***(R)*-83** tr (major) = 13.2 min, tr (minor) = 14.1 min, 90% e.e..

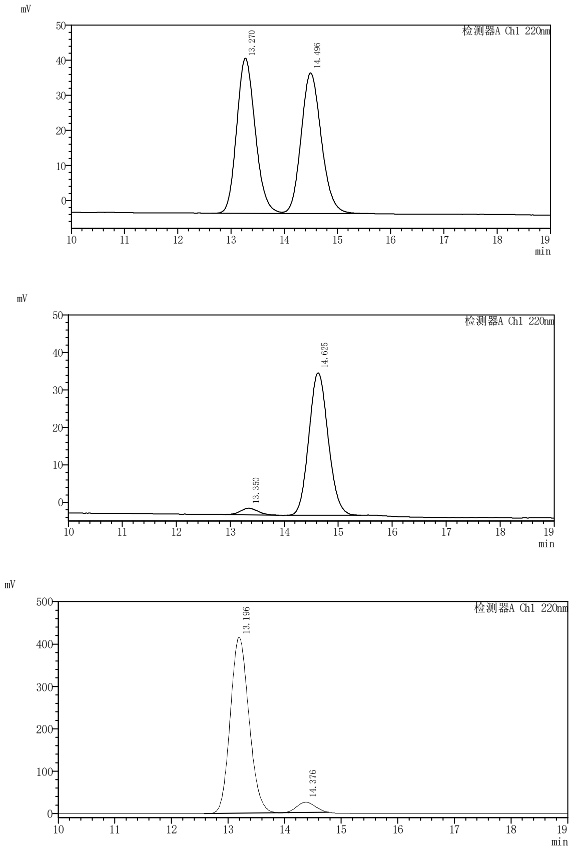


| No. | Time | Area | Area (%) | No. | Time | Area | Area (%) |
|---|---|---|---|---|---|---|---|
| 1 | 13.270 | 1073448 | 50.322 | 1 | 13.350 | 39598 | 4.009 |
| 2 | 14.496 | 1059728 | 49.678 | 2 | 14.625 | 948127 | 95.991 |
| | | | | 3 | 13.196 | 9542225 | 94.657 |
| | | | | 4 | 14.376 | 538604 | 5.343 |

**Supplementary Figure 104. HPLC data of 83.**

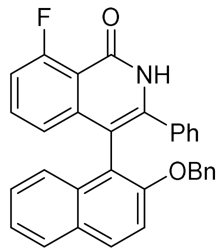


**4-(2-(benzyloxy)naphthalen-1-yl)-8-fluoro-3-phenylisoquinolin-1(2H)-one**
***(S)*-84** White solid (41.9 mg, 89%).
***(R)*-84** White solid (42.4 mg, 90%).
$^1$H NMR (600 MHz, $CDCl_3$) δ 9.42 (s, 1H), 7.84 – 7.73 (m, 2H), 7.48 – 7.44 (m, 1H), 7.36 – 7.29 (m, 3H), 7.24 – 7.18 (m, 4H), 7.14-7.16 (m, 3H), 7.11 – 7.04 (m, 3H), 6.98 (ddd, *J* = 5.0, 3.1, 1.5 Hz, 2H), 6.66 (d, *J* = 8.2 Hz, 1H), 5.01 (d, *J* = 12.7 Hz, 1H), 4.92 (d, *J* = 12.7 Hz, 1H).
$^{13}$C NMR (151 MHz, $CDCl_3$) δ 163.61, 161.86, 160.73, 154.51, 141.75, 139.91, 137.05, 134.72, 134.62, 133.47, 133.40, 130.15, 128.95, 128.92, 128.41, 128.21, 128.15, 127.91, 127.63, 127.14, 126.47, 126.07, 125.45, 124.82, 123.84, 121.48, 118.53, 116.04, 114.62, 114.51, 113.30, 113.16, 70.43. **HRMS** (ESI-TOF) (m/z): Calcd for $C_{32}H_{23}FNO_2^+$, ([M + H]$^+$), 472.1707, found.472.1720.
**HPLC** conditions: Daicel Chiralpak OD-3 column (80: 20 hexane: 2-propanol, 0.8 mL/min, 40 ºC, 254 nm); ***(S)*-84** tr (major) = 8.0 min, tr (minor) = 9.4 min, 97% e.e.. ***(R)*-84** tr (major) = 9.4 min, tr (minor) = 8.0 min, 90% e.e..

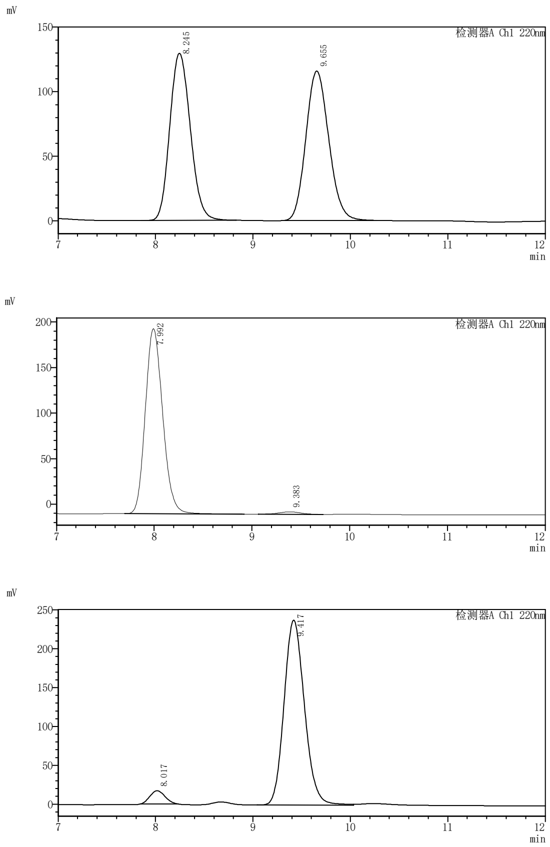


| No. | Time | Area | Area (%) | No. | Time | Area | Area (%) |
|---|---|---|---|---|---|---|---|
| 1 | 8.245 | 1898450 | 50.065 | 1 | 7.992 | 2519183 | 98.346 |
| 2 | 9.655 | 1893487 | 49.935 | 2 | 9.383 | 42359 | 1.654 |
| | | | | 3 | 8.017 | 186848 | 5.100 |
| | | | | 4 | 9.417 | 3476679 | 94.900 |

**Supplementary Figure 105. HPLC data of 84.**

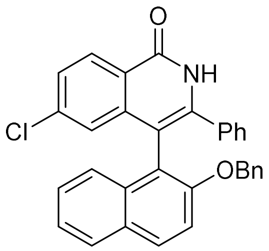

**4-(2-(benzyloxy)naphthalen-1-yl)-6-chloro-3-phenylisoquinolin-1(2H)-one**

***(S)*-85** White solid (42.4 mg, 87%).

***(R)*-85** White solid (43.4 mg, 89%).

$^1$H NMR (600 MHz, $CDCl_3$) δ 9.63 (s, 1H), 8.42 (d, $J$ = 8.6 Hz, 1H), 7.87 – 7.76 (m, 2H), 7.46 – 7.39 (m, 2H), 7.36 – 7.30 (m, 2H), 7.24 – 7.19 (m, 4H), 7.19 – 7.14 (m, 3H), 7.11 – 7.04 (m, 2H), 7.00 (dd, $J$ = 6.6, 3.0 Hz, 2H), 6.85 (d, $J$ = 1.9 Hz, 1H), 5.05 (d, $J$ = 12.8 Hz, 1H), 4.97 (d, $J$ = 12.8 Hz, 1H).

$^{13}$C NMR (151 MHz, $CDCl_3$) δ 162.64, 154.52, 140.43, 139.99, 139.45, 137.00, 134.87, 134.45, 130.32, 129.29, 128.96, 128.91, 128.45, 128.26, 128.16, 127.96, 127.67, 127.20, 127.10, 126.52, 124.91, 124.66, 123.86, 123.58, 117.73, 114.47, 110.00, 70.38. **HRMS** (ESI-TOF) (m/z): Calcd for $C_{32}H_{23}ClNO_2^+$, ([M + H]$^+$), 488.1412, found.488.1403.

**HPLC** conditions: Daicel Chiralpak OD-3 column (80: 20 hexane: 2-propanol, 0.8 mL/min, 40 ºC, 254 nm); ***(S)*-85** tr (major) = 8.0 min, tr (minor) = 9.4 min, 93% e.e.. ***(R)*-85** tr (major) = 9.4 min, tr (minor) = 8.0 min, 90% e.e..

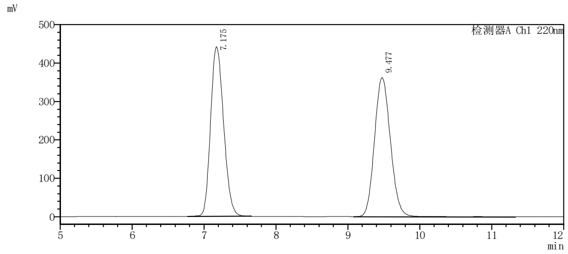

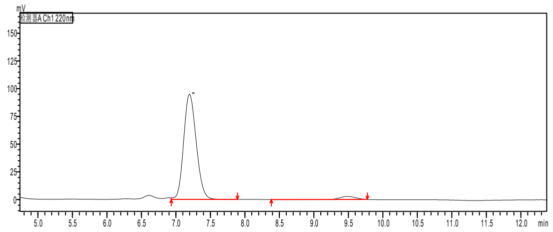

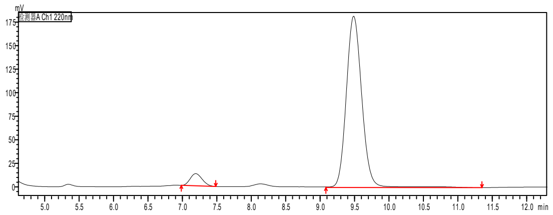

| No. | Time | Area | Area (%) | No. | Time | Area | Area (%) |
|---|---|---|---|---|---|---|---|
| 1 | 7.175 | 5564600 | 49.642 | 1 | 7.200 | 1202981 | 96.616 |
| 2 | 9.477 | 5644755 | 50.358 | 2 | 9.492 | 42138 | 3.384 |
| | | | | 3 | 7.192 | 155711 | 5.162 |
| | | | | 4 | 9.487 | 2860765 | 94.838 |

**Supplementary Figure 106. HPLC data of 86.**

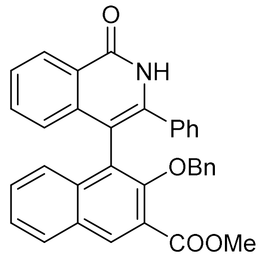

**methyl 3-(benzyloxy)-4-(1-oxo-3-phenyl-1,2-dihydroisoquinolin-4-yl)-2-naphthoate**

***(S)*-86** White solid (46.0 mg, 90%).

***(R)*-86** White solid (45.0 mg, 88%).

$^1$H NMR (600 MHz, $CDCl_3$) δ 9.38 (d, $J$ = 31.3 Hz, 1H), 8.49 (dq, $J$ = 6.7, 3.0, 2.4 Hz, 1H), 8.43 (d, $J$ = 1.7 Hz, 1H), 7.93 (dt, $J$ = 7.6, 1.6 Hz, 1H), 7.55 (dd, $J$ = 7.4, 2.3 Hz, 1H), 7.52 – 7.41 (m, 4H), 7.18-7.21 (m, 3H), 7.14 (ddt, $J$ = 6.8, 4.8, 1.8 Hz, 1H), 7.10 – 7.01 (m, 4H), 6.97 – 6.89 (m, 3H), 4.84 (dd, $J$ = 10.6, 1.7 Hz, 1H), 4.43 (dd, $J$ = 10.5, 1.8 Hz, 1H), 3.86 (s, 3H).

$^{13}$C NMR (151 MHz, $CDCl_3$) δ 166.63, 162.86, 153.80, 139.21, 139.05, 137.11, 136.57, 134.79, 133.55, 133.04, 129.46, 129.39, 129.02, 129.00, 128.32, 128.19, 127.81, 127.75, 127.72, 127.42, 126.74, 126.61, 125.80, 125.70, 125.55, 125.36, 125.02, 109.62, 75.83, 52.39.

**HRMS** (ESI-TOF) (m/z): Calcd for $C_{35}H_{25}NO_4^+$, ([M + H]$^+$), 512.1856, found 512.1858.

**HPLC** conditions: Daicel Chiralpak OD-3 column (80: 20 hexane: 2-propanol, 0.8 mL/min, 40 ºC, 254 nm); ***(S)*-86** tr (major) = 7.3 min, tr (minor) = 8.4 min, 92% e.e.. ***(R)*-86** tr (major) = 8.4 min, tr (minor) = 7.3 min, 95% e.e..

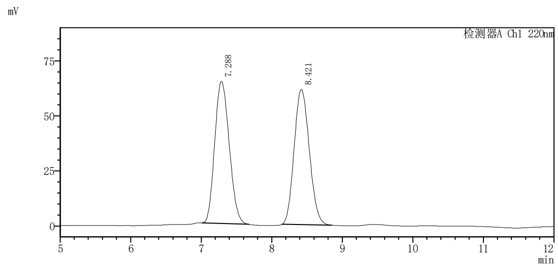


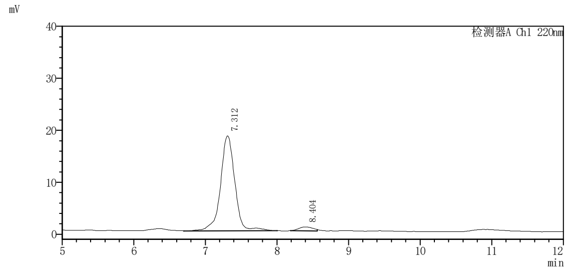


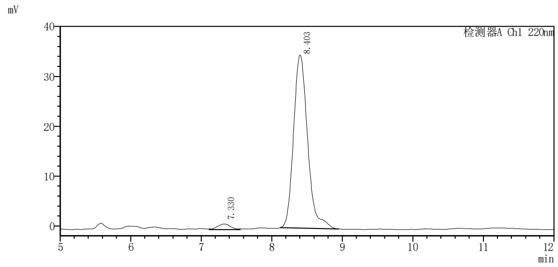


| No. | Time | Area | Area (%) | No. | Time | Area | Area (%) |
|---|---|---|---|---|---|---|---|
| 1 | 7.288 | 923174 | 50.145 | 1 | 7.312 | 253913 | 96.083 |
| 2 | 8.421 | 917832 | 49.855 | 2 | 8.404 | 10350 | 3.917 |
| | | | | 3 | 7.330 | 14553 | 2.942 |

|  |  |  |  | 4 | 8.403 | 480119 | 97.058 |
|---|---|---|---|---|---|---|---|

**Supplementary Figure 107. HPLC data of 86.**

## 2.4 Synthetic applications

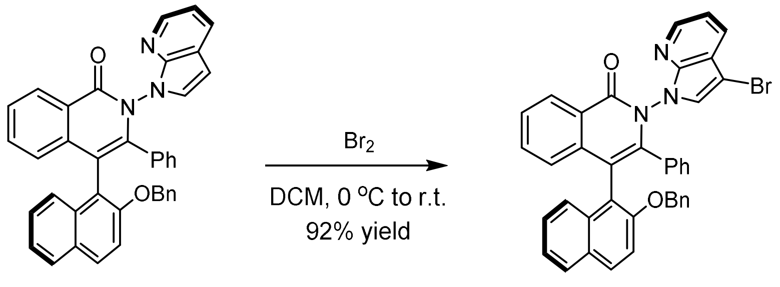


(*dia*)-**49** (92% ee) **87** (92% ee)

A scew-cap vial (8 mL) was charged with ***(dia)*-49** (56.9 mg, 0.1 mmol, 1.0 equiv) in DCM (1.0 mL). The resulted mixture was stirred at 0 °C, then $Br_2$ (19.2 mg, 0. 12 mmol, 1.2 equiv) was added slowly. The reaction was allowed to warm to room temperature and stirred for 12 h. After the reaction was complete (monitored by TLC), the solvent was removed under reduced pressure. Then the residue was purified by silica gel column chromatography (PE/EA = 2:1) to afford the desired product **87** (59.5 mg, 92% yield) as a white solid.

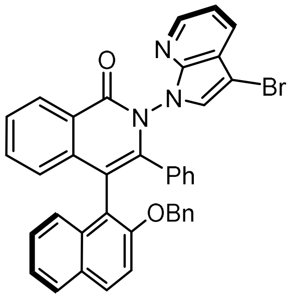

**4-(2-(benzyloxy)naphthalen-1-yl)-2-(3-bromo-1H-pyrrolo[2,3-b]pyridin-1-yl)-3-phenylisoquinolin-1(2H)-one (87)**

White solid (59.5 mg, 92%), $^{1}$H NMR (600 MHz, $CDCl_3$) δ 8.58 – 8.53 (m, 1H), 8.37 (dd, *J* = 4.8, 1.5 Hz, 1H), 7.78 (dt, *J* = 8.6, 1.0 Hz, 1H), 7.75 – 7.70 (m, 2H), 7.70 – 7.67 (m, 1H), 7.56 – 7.47 (m, 2H), 7.39 (ddd, *J* = 8.4, 6.8, 1.3 Hz, 1H), 7.31 – 7.26 (m, 4H), 7.19 (dd, *J* = 10.5, 8.1 Hz, 2H), 7.17 – 7.10 (m, 4H), 6.95 – 6.92 (m, 1H), 6.91 – 6.84 (m, 2H), 6.76 (tt, *J* = 7.5, 1.3 Hz, 1H), 6.42 (td, *J* = 7.7, 1.3 Hz, 1H), 5.14 (d, *J* = 1.8 Hz, 2H).

$^{13}$C NMR (151 MHz, $CDCl_3$) δ 162.35, 154.86, 146.27, 145.20, 141.93, 137.64, 137.27, 133.97, 133.46, 132.05, 130.04, 128.80, 128.68, 128.53, 128.38, 128.33, 128.13, 127.99, 127.91, 127.83, 127.26, 126.91, 126.70, 126.58, 125.66, 125.43, 125.21, 123.78, 118.63, 118.56, 117.69, 114.07, 113.13, 89.37, 70.52.

**HRMS** (ESI-TOF) (m/z): Calcd for $C_{39}H_{27}BrN_3O_2^+$, ([M + H]$^+$), 648.1281, found 648.1274. **$[\alpha]_D^{20}$** = +34 (c = 0.1, $CHCl_3$).

**HPLC** conditions: Daicel Chiralpak IC column (90: 10 hexane: 2-propanol, 0.8 mL/min, 40 °C, 254 nm); tr (major) = 20.0 min, tr (minor) = 17.9 min, 92% e.e.. >20:1 dr determined by crude NMR.

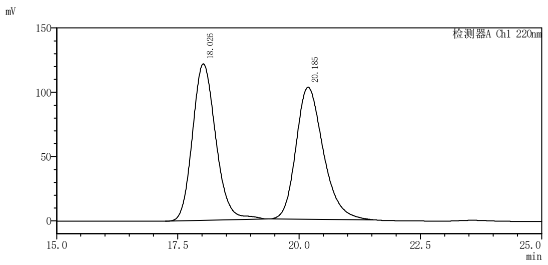


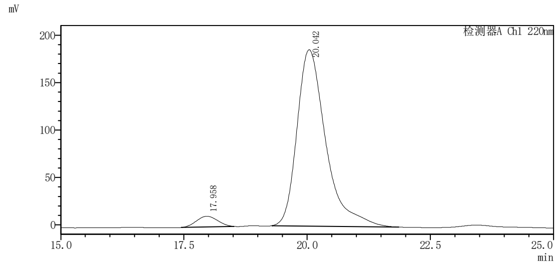


| No. | Time | Area | Area (%) | No. | Time | Area | Area (%) |
|---|---|---|---|---|---|---|---|
| 1 | 18.026 | 4071052 | 50.258 | 1 | 17.958 | 338724 | 4.291 |
| 2 | 20.185 | 4029189 | 49.742 | 2 | 20.042 | 7554885 | 95.709 |

**Supplementary Figure 108. HPLC data of 87.**

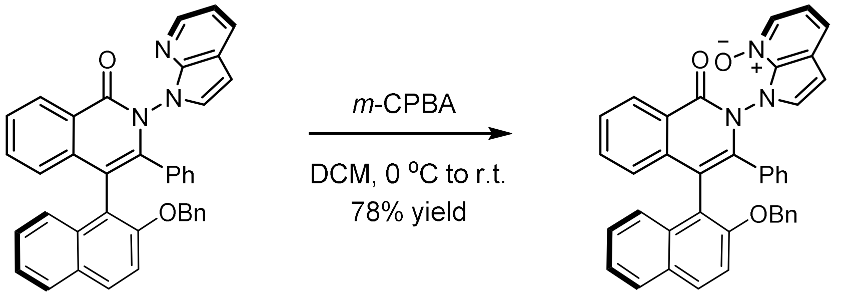


**(*dia*)-49** (92% ee) **88** (92% ee)

A scew-cap vial (8 mL) was charged with **(*dia*)-49** (56.9 mg, 0.1 mmol, 1.0 equiv), in DCM (1.0 mL). The resulted mixture was stirred at 0 °C, then *m*-CPBA (20.6 mg, 0.12 mmol, 1.2 equiv) was added slowly. The reaction was allowed to warm to room temperature and stirred for 12 h. After the reaction was complete (monitored by TLC), the solvent was removed under reduced pressure. Then the residue was purified by silica gel column chromatography (PE/EA = 1:1) to afford the desired product **88** (45.6 mg, 78% yield) as a white solid.

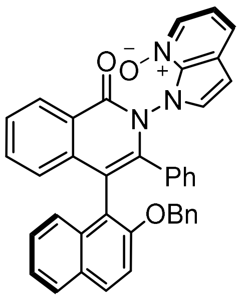

**1-(4-(2-(benzyloxy)naphthalen-1-yl)-1-oxo-3-phenylisoquinolin-2(1H)-yl)-1H-pyrrolo[2,3-b]pyridine 7-oxide (88)**

White solid (45.6 mg, 78%), $^{1}$H NMR (600 MHz, $CD_2Cl_2$) δ 8.41 – 8.35 (m, 1H), 8.00 (d, *J* = 6.2 Hz, 1H), 7.77 (d, *J* = 8.5 Hz, 1H), 7.66 (d, *J* = 9.1 Hz, 1H), 7.61 (d, *J* = 8.2 Hz, 1H), 7.38-7.43 (m, 2H), 7.35 – 7.27 (m, 2H), 7.24 (dt, *J* = 7.8, 1.5 Hz, 1H), 7.17-7.21 (m, 6H), 7.15 – 7.10 (m, 2H), 6.94 – 6.84 (m, 3H), 6.79 (dd, *J* = 7.6, 1.6 Hz, 1H), 6.77 – 6.72 (m, 1H), 6.51 (td, *J* = 7.7, 1.4 Hz, 1H), 6.30 (d, *J* = 3.7 Hz, 1H), 5.13 – 5.05 (m, 2H).

$^{13}$C NMR (151 MHz, $CD_2Cl_2$) δ 161.61, 154.48, 142.67, 137.81, 137.59, 134.83, 134.16, 133.45, 133.21, 130.85, 130.01, 129.47, 129.07, 128.95, 128.58, 128.46, 128.13, 127.86, 127.79, 127.35, 127.30, 127.14, 127.04, 126.90, 125.58, 125.52, 124.73, 124.42, 123.93, 118.70, 118.09, 114.10, 112.19, 101.93, 70.64.

**HRMS** (ESI-TOF) (m/z): Calcd for $C_{39}H_{28}N_3O_3^+$, ([M + H]$^+$), 586.2125, found 586.2125. **$[\alpha]_D^{20}$** = +20 (c = 0.1, $CHCl_3$).

**HPLC** conditions: Daicel Chiralpak OD-3 column (80: 20 hexane: 2-propanol, 0.8 mL/min, 40 °C, 254 nm); tr (major) = 13.1 min, tr (minor) = 31.7 min, 92% e.e..

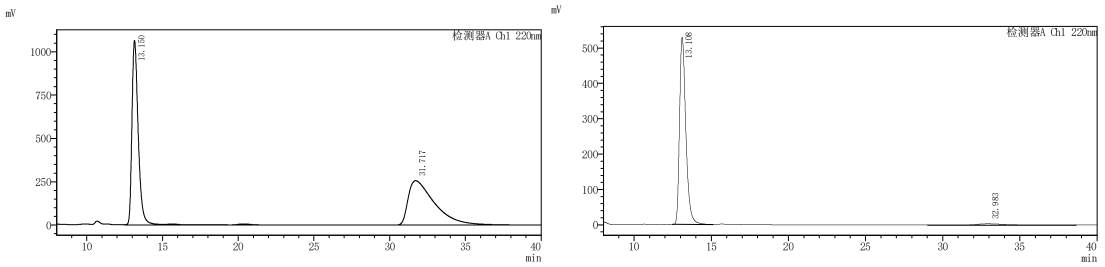


| No. | Time | Area | Area (%) | No. | Time | Area | Area (%) |
|---|---|---|---|---|---|---|---|
| 1 | 13.150 | 31464741 | 50.209 | 1 | 13.108 | 15055871 | 96.109 |
| 2 | 31.717 | 31202292 | 49.791 | 2 | 32.983 | 609600 | 3.891 |

**Supplementary Figure 109. HPLC data of 88.**

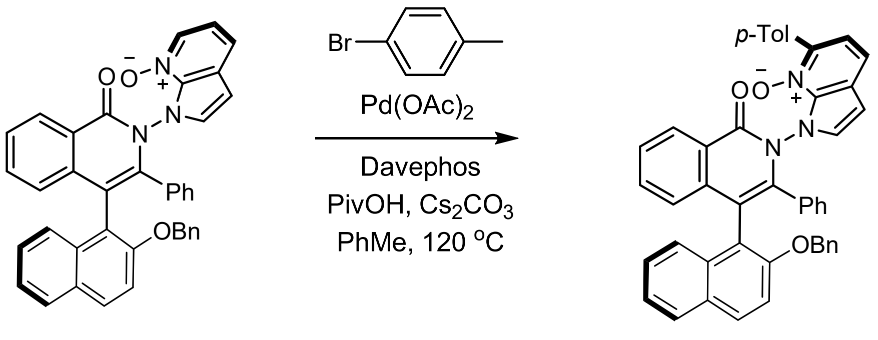


**88** (92% ee) **89**, 58% (90% ee)

Product **88** was prepared by following a literature report.[4] In a $N_2$-filled glove box, a scew-cap vial (8 mL) was charged with **88** (58.5 mg, 0.1 mmol, 1.0 equiv), 1-bromo-4-methylbenzene (17 mg, 0.1 mmol, 1.0 equiv), $Pd(OAc)_2$ (1.1 mg, 0.005 mmol, 5 mol %), Davephos (7.9 mg, 0.02 mmol, 20 mol %), PivOH (3 mg, 0.03 mmol, 30 mol%) and $Cs_2CO_3$ (65.2 mg, 0.2 mmol, 2.0 equiv) in toluene (1.0 mL). The reaction mixture was then stirred at 120 °C in a preset oil bath for 12 h. After the reaction was complete (monitored by TLC), the reaction mixture was allowed to cool to room temperature. The resulting mixture was then concentrated and purified by silica gel chromatography using PE:EA (3:1) as the eluent to yield compound **89** (39.2 mg, 58% yield) as a white solid.

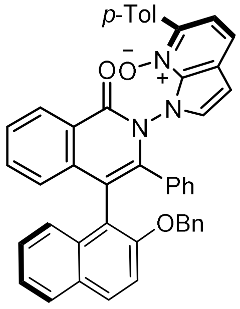


**1-(1-oxo-3,4-diphenylisoquinolin-2(1H)-yl)-6-(p-tolyl)-1H-pyrrolo[2,3-b]pyridine 7-oxide (89)**

White solid (39.2 mg, 58%), $^{1}H$ NMR (600 MHz, $CDCl_3$) δ 8.50 (dd, $J$ = 7.8, 1.7 Hz, 1H), 8.09 (d, $J$ = 8.5 Hz, 1H), 7.84 – 7.77 (m, 2H), 7.69 (dd, $J$ = 15.7, 8.6 Hz, 2H), 7.40-7.48 (m, 3H), 7.35 (dt, $J$ = 7.8, 1.6 Hz, 1H), 7.33 – 7.25 (m, 7H), 7.22 (dd, $J$ = 7.9, 1.7 Hz, 1H), 7.19 – 7.14 (m, 3H), 7.11 (d, $J$ = 8.3 Hz, 1H), 6.98 (d, $J$ = 3.7 Hz, 1H), 6.91 – 6.85 (m, 2H), 6.79 (td, $J$ = 7.5, 1.4 Hz, 1H), 6.57 (td, $J$ = 7.6, 1.3 Hz, 1H), 6.32 (d, $J$ = 3.6 Hz, 1H), 5.13 (s, 2H), 2.40 (s, 3H).

$^{13}C$ NMR (151 MHz, $CDCl_3$) δ 162.07, 154.10, 144.46, 142.68, 139.02, 137.83, 137.48, 136.66, 134.44, 133.14, 130.56, 130.09, 129.83, 129.54, 129.23, 128.85, 128.69, 128.46, 127.96, 127.67, 127.49, 126.91, 126.85, 126.53, 126.39, 125.37, 124.89, 123.83, 122.63, 119.72, 119.38, 119.03, 114.02, 112.10, 101.43, 70.48, 21.43.

**HRMS** (ESI-TOF) (m/z): Calcd for $C_{46}H_{34}N_3O_3^+$, ([M + H]$^+$), 676.2595, found 676.2600. **$[\alpha]_D^{20}$** = 15 (c = 0.1, $CHCl_3$).

**HPLC** conditions: Daicel Chiralpak OD-3 column (70: 30 hexane: ethanol, 0.8 mL/min, 40 °C, 254 nm); tr (major) = 6.6 min, tr (minor) = 5.6 min, 90% e.e..

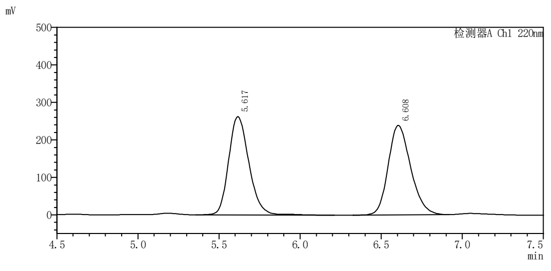


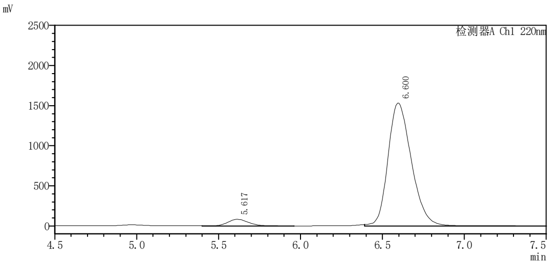

| No. | Time | Area | Area (%) | No. | Time | Area | Area (%) |
|---|---|---|---|---|---|---|---|
| 1 | 5.617 | 2286214 | 49.838 | 1 | 5.617 | 802151 | 5.001 |
| 2 | 6.608 | 2301091 | 50.162 | 2 | 6.600 | 15236226 | 94.999 |

**Supplementary Figure 110. HPLC data of 89.**

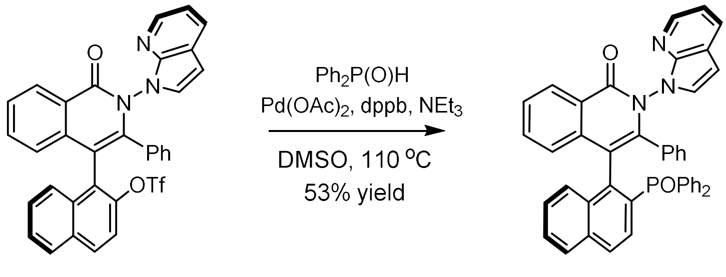


**(*dia*)-77** (91% ee) **90** (91% ee)

In a $N_2$-filled glove box, a scew-cap vial (8 mL) was charged with **(dia)-77** (61.1 mg, 0.1 mmol, 1.0 equiv), $Ph_2P(O)H$ (80.8 mg, 0.4 mmol, 4.0 equiv), $Pd(OAc)_2$ (2.2 mg, 0.01 mmol, 10 mol %), dppb (5.1 mg, 0.012 mmol, 12 mol %), $NEt_3$ (60.6 mg, 0.6 mmol, 6.0 equiv) in DMSO (1.0 mL). The reaction mixture was then stirred at 110 °C in a preset oil bath for 12 h. After the reaction was complete (monitored by TLC), the reaction mixture was allowed to cool to room temperature. The resulting mixture was then concentrated and purified by silica gel chromatography using PE:EA (1:1) as the eluent to yield compound **90** (35.1 mg, 53% yield) as a white solid.

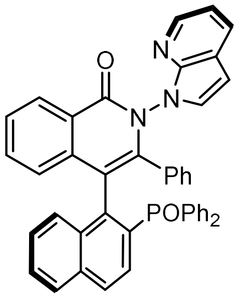

**4-(2-(diphenylphosphoryl)naphthalen-1-yl)-3-phenyl-2-(1H-pyrrolo[2,3-b]pyridin-1-yl)isoquinolin-1(2H)-one (90)**

White solid (35.1 mg, 53%), $^1$H NMR (600 MHz, $CDCl_3$) δ 8.44 (dd, $J$ = 8.1, 1.5 Hz, 1H), 8.28 (dt, $J$ = 4.8, 1.4 Hz, 1H), 8.21 – 8.11 (m, 1H), 8.09 – 7.98 (m, 1H), 7.80 – 7.74 (m, 1H), 7.73 – 7.67 (m, 2H), 7.56 – 7.50 (m, 3H), 7.50 – 7.39 (m, 5H), 7.35 (ddd, $J$ = 8.8, 6.7, 1.4 Hz, 1H), 7.34 – 7.29 (m, 2H), 7.29 – 7.24 (m, 2H), 7.21 – 7.15 (m, 2H), 6.99 (ddd, $J$ = 7.8, 4.8, 1.2 Hz, 1H), 6.94 (d, $J$ = 7.8 Hz, 1H), 6.90 – 6.84 (m, 2H), 6.74 (td, $J$ = 7.5, 1.3 Hz, 1H), 6.39 (t, $J$ = 7.7 Hz, 1H), 6.33 (dd, $J$ = 3.8, 1.2 Hz, 1H), 6.16 (d, $J$ = 8.2 Hz, 1H). $^{13}$C NMR (151 MHz, $CDCl_3$) δ 162.75, 146.58, 144.19, 143.05, 140.64, 137.01, 134.25, 133.77, 132.38, 132.13, 132.03, 131.98, 131.94, 131.74, 131.70, 131.24 (d, $J$ = 6.4 Hz), 130.81, 129.88, 129.04, 128.57, 128.49, 128.43, 128.24, 128.06, 127.97, 127.88, 127.66 (d, $J$ = 3.2 Hz), 127.49, 126.44, 126.27, 125.18, 118.64 (d, $J$ = 68 Hz), 116.80 (d, $J$ = 118 Hz), 100.68.

$^{31}$P NMR (243 MHz, $CDCl_3$) δ 29.31.

**HRMS** (ESI-TOF) (m/z): Calcd for $C_{44}H_{31}N_3O_2P^+$, ([M + H]$^+$), 664.2148, found 664.2133. **$[\alpha]_D^{20}$** = 42 (c = 0.1, $CHCl_3$).

**HPLC** conditions: Daicel Chiralpak OD-3 column (80: 20 hexane: 2-propanol, 0.8 mL/min, 40 °C, 254 nm); tr (major) = 6..035 min, tr (minor) = 6.908 min, 91% e.e. >20:1 dr determined by crude NMR.

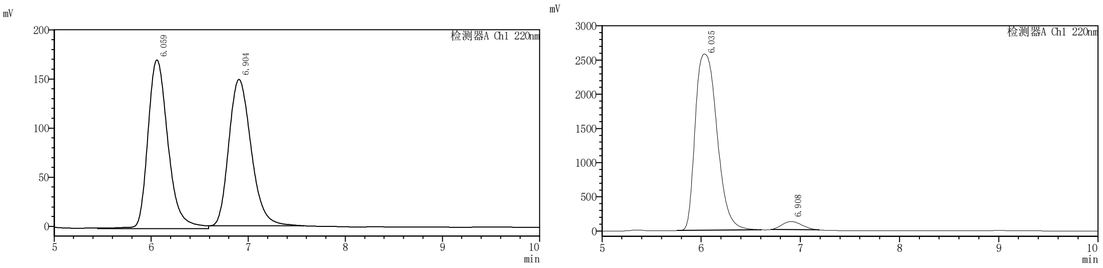


| No. | Time | Area | Area (%) | No. | Time | Area | Area (%) |
|---|---|---|---|---|---|---|---|
| 1 | 6.059 | 2489968 | 51.129 | 1 | 6.035 | 39463693 | 95.991 |
| 2 | 6.904 | 2379975 | 48.871 | 2 | 6.908 | 1648152 | 4.009 |

**Supplementary Figure 111. HPLC data of 90.**

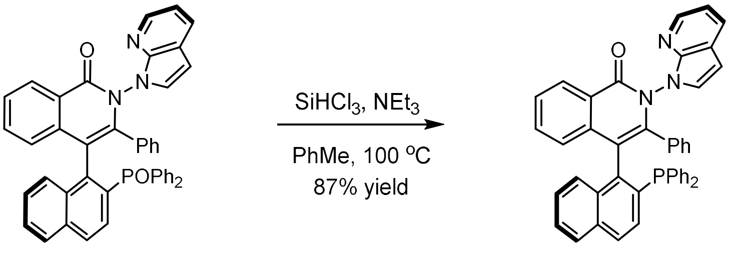


**90** (91% ee)

**91** (90% ee, 5:1 dr)

In a $N_2$-filled glove box, a scew-cap vial (8 mL) was charged with **90** (66.3 mg, 0.1 mmol) and HSiCl3 (40.6 mg, 0.3 mmol) in 1 mL toluene was stirred at 100 °C heated by an oil bath for 12 h. After removal of the solvent under reduced pressure, the residue was purified by flash column chromatography to give the product **91** (56.3 mg, 87% yield) as a white solid.

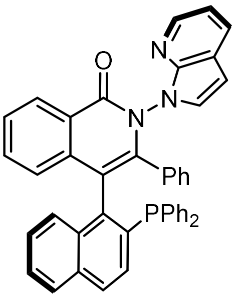


**4-(2-(diphenylphosphaneyl)naphthalen-1-yl)-3-phenyl-2-(1H-pyrrolo[2,3-b]pyridin-1-yl)isoquinolin-1(2H)-one (91)**

White solid (56.3 mg, 87%). $^1$H NMR (600 MHz, $CD_2Cl_2$) δ 8.37 – 8.33 (m, 1H), 8.19 (dd, $J$ = 4.8, 1.5 Hz, 1H), 7.99 (d, $J$ = 8.1 Hz, 1H), 7.75 – 7.68 (m, 2H), 7.64 (d, $J$ = 8.5 Hz, 1H), 7.44 (dqd, $J$ = 8.3, 6.9, 1.5 Hz, 2H), 7.39 – 7.34 (m, 1H), 7.33 – 7.28 (m, 2H), 7.21 – 7.12 (m, 5H), 7.06 – 6.99 (m, 5H), 6.97 (dd, $J$ = 7.7, 4.6 Hz, 1H), 6.86 (td, $J$ = 7.3, 6.7, 1.4 Hz, 2H), 6.82 – 6.77 (m, 1H), 6.76 – 6.68 (m, 2H), 6.36 – 6.28 (m, 2H), 6.25 – 6.21 (m, 1H).

$^{13}$C NMR (151 MHz, $CD_2Cl_2$) δ 161.76, 144.50, 142.46, 140.52, 137.73, 134.48, 133.90, 133.62 (d, $J$ = 19.4 Hz,), 132.84 (d, $J$ = 16.9 Hz), 132.64, 130.38, 129.91, 129.12, 128.97, 128.49, 128.45, 128.40, 128.33, 128.10, 127.98 (d, $J$ = 116 Hz), 127.19, 127.11, 126.82, 126.54, 126.42, 125.14, 118.63, 117.22, 115.61, 99.44.

$^{31}$P NMR (243 MHz, $CD_2Cl_2$) δ -14.45.

**HRMS** (ESI-TOF) (m/z): Calcd for $C_{44}H_{31}N_3OP^+$, ([M + H]$^+$), 648.2199, found 648.2182. **[α]$_D^{20}$** = 64 (c = 0.1, $CHCl_3$).

**HPLC** conditions: Daicel Chiralpak IC column (80: 20 hexane: 2-propanol, 0.8 mL/min, 40 °C, 254 nm); tr (major) = 8.8 min, tr (minor) = 13.2 min, 90% e.e. 5:1 dr was determined by crude NMR.

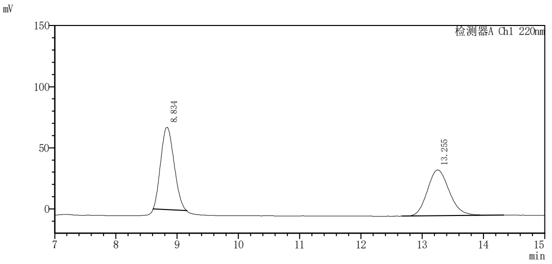


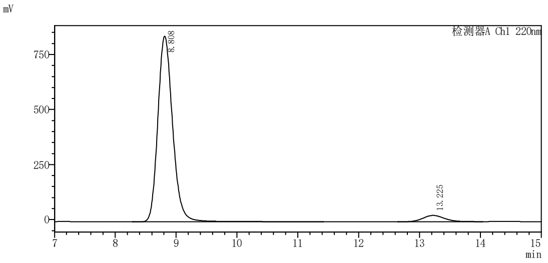


| No. | Time | Area | Area (%) | No. | Time | Area | Area (%) |
|---|---|---|---|---|---|---|---|
| 1 | 8.834 | 1021520 | 52.193 | 1 | 8.808 | 14257276 | 95.250 |
| 2 | 13.255 | 935684 | 47.807 | 2 | 13.225 | 711016 | 4.750 |

**Supplementary Figure 112. HPLC data of 91.**

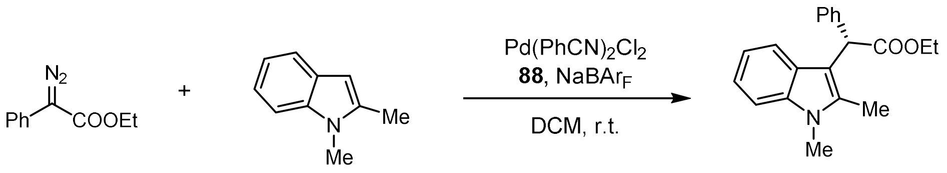


**92**, 67%, 59% ee

A scew-cap vial (8 mL) was charged with ethyl 2-diazo-2-phenylacetate (19.0 mg, 0.1 mmol, 1.0 equiv), 1,2-dimethyl-1H-indole (14.5 mg, 0.1 mmol, 1.0 equiv), $Pd(PhCN)_2Cl_2$ (2.6 mg, 10 mol%), **88** (4.3 mg, 10 mol%) and $NaBAr_F$ (21.3 mg, 24 mol%) in DCM (1 mL) was stirred in a vial at 25 °C for 12 h. The reaction mixture was evaporated under vacuum and the residue was purified by preparative TLC to give the corresponding product **92** (20.6 mg, 67% yield) as a white solid.

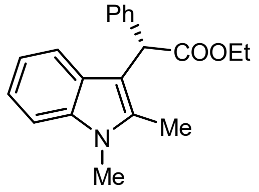


**ethyl (S)-2-(1,2-dimethyl-1H-indol-3-yl)-2-phenylacetate (92)**

White solid (20.6 mg, 67%), $^1H$ NMR (600 MHz, $CDCl_3$) δ 7.49 (dt, $J$ = 8.0, 1.0 Hz, 1H), 7.30 – 7.23 (m, 5H), 7.22 – 7.18 (m, 1H), 7.14 (ddd, $J$ = 8.1, 7.0, 1.2 Hz, 1H), 7.02 (ddd, $J$ = 8.0, 6.9, 1.0 Hz, 1H), 5.29 (s, 1H), 4.23 (dqd, $J$ = 10.8, 7.1, 1.1 Hz, 1H), 4.17 (dqd, $J$ = 10.9, 7.1, 1.0 Hz, 1H), 3.66 (s, 3H), 2.35 (s, 3H), 1.25 – 1.20 (m, 3H).

$^{13}C$ NMR (151 MHz, $CDCl_3$) δ 173.23, 139.06, 136.65, 134.73, 128.22, 126.91, 126.62, 120.71, 119.45, 119.18, 108.62, 107.93, 61.00, 48.40, 29.61, 14.24, 10.76.

**HRMS** (ESI-TOF) (m/z): Calcd for $C_{20}H_{22}NO_2^+$, ($[M + H]^+$), 308.1645, found 308.1638. $[\alpha]_D^{20}$ = 42 (c = 0.1, $CHCl_3$).

**HPLC** conditions: Daicel Chiralpak IC column (80: 20 hexane: 2-propanol, 0.8 mL/min, 40 °C, 254 nm); tr (major) = 6.4 min, tr (minor) = 7.2 min, 59% ee.

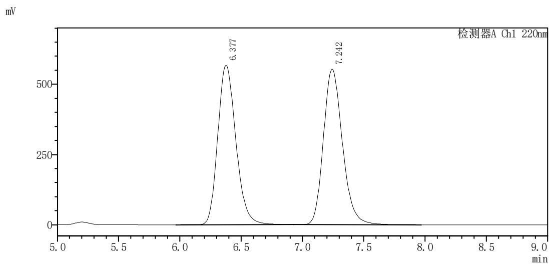


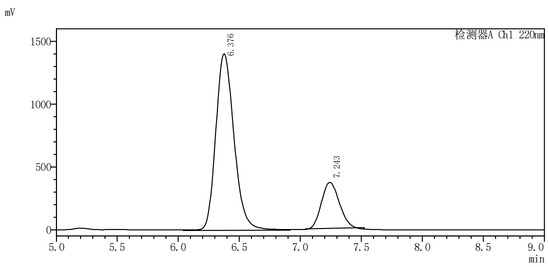

| No. | Time | Area | Area (%) | No. | Time | Area | Area (%) |
|---|---|---|---|---|---|---|---|
| 1 | 6.377 | 5860337 | 49.473 | 1 | 6.376 | 14700656 | 79.986 |
| 2 | 7.242 | 5985096 | 50.527 | 2 | 7.243 | 3678405 | 20.014 |

**Supplementary Figure 113. HPLC data of 92.**

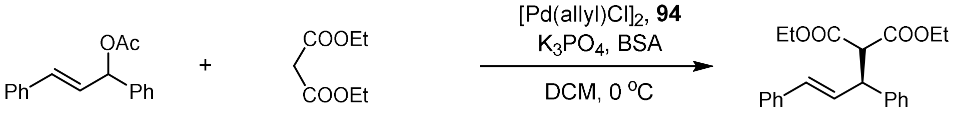


**93**

To a suspension of the desired chiral ligand **94** (3.9 mg, 6 mol%), $[Pd(allyl)Cl]_2$ (0.9 mg, 2.5 mol%) in DCM (2.0 mL) were stirred at r.t. for 30 min under $N_2$. Then the reaction solution was cooled to 0 °C, 1,3-diphenyl-2- propenyl acetate (25.2 mg, 0.1 mmol, 1 equiv), dimethyl malonate (32.0 mg, 0.2 mmol, 2.0 equiv), $K_3PO_4$ (42.4 mg, 0.2 mmol, 2.0 equiv) and BSA (50.8 mg, 0.25 mmol, 2.5 equiv) were subsequently added. After the reaction time the reaction mixture was diluted with EtOAc (5 mL). Saturated $NH_4Cl$ (aq) (10 mL) was then added, the mixture was extracted with EtOAc (3 × 10 mL), and the extract was dried over $MgSO_4$. The residue was purified by preparative TLC to give the corresponding product **93**.

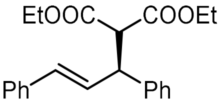


**diethyl (S,E)-2-(1,3-diphenylallyl)malonate (93)**

Yellow oil (21.1 mg, 60%), $^1$H NMR (600 MHz, $CDCl_3$ ) δ 7.35 – 7.28 (m, 6H), 7.26 (t, $J$ = 7.6 Hz, 2H), 7.24 – 7.16 (m, 2H), 6.47 (d, $J$ = 15.7 Hz, 1H), 6.34 (dd, $J$ = 15.7, 8.6 Hz, 1H), 4.26 (ddd, $J$ = 10.9, 8.6, 0.9 Hz, 1H), 4.17 (q, $J$ = 7.1 Hz, 2H), 3.97 (qt, $J$ = 7.1, 3.5 Hz, 2H), 3.92 (d, $J$ = 11.0 Hz, 1H), 1.20 (t, $J$ = 7.1 Hz, 3H), 1.01 (t, $J$ = 7.1 Hz, 3H).

$^{13}$C NMR (151 MHz, $CDCl_3$) δ 167.85, 167.42, 140.32, 136.86, 131.68, 129.36, 128.65, 128.46, 128.00, 127.51, 127.09, 126.35, 61.58, 61.37, 57.79, 49.22, 14.14, 13.78.

**HRMS** (ESI-TOF) (m/z): Calcd for $C_{22}H_{25}O_4^+$, ([M + H]$^+$), 353.1747, found 353.1756.

**HPLC** conditions: Daicel Chiralpak AD-H column (95:5 hexane: 2-propanol, 1.0 mL/min, 40 °C, 254 nm); tr (major) = 11.76 min, tr (minor) = 9.32 min, 86% ee.

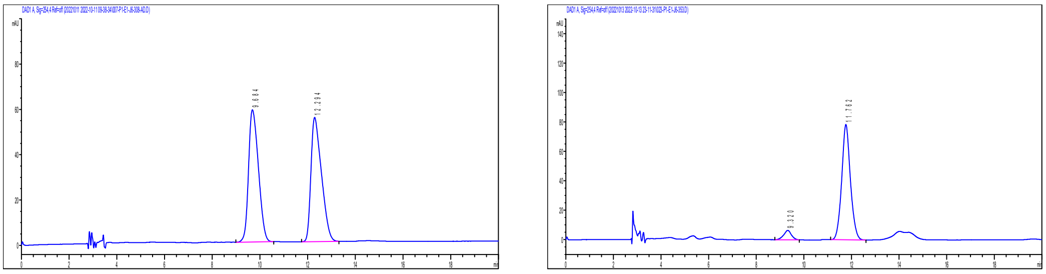

| No. | Time | Area | Area (%) | No. | Time | Area | Area (%) |
|---|---|---|---|---|---|---|---|
| 1 | 9.684 | 1674.7 | 50.00 | 1 | 9.320 | 144.9 | 6.79 |
| 2 | 12.294 | 1674.7 | 50.00 | 2 | 11.762 | 1988.9 | 93.21 |

**Supplementary Figure 114. HPLC data of 93.**

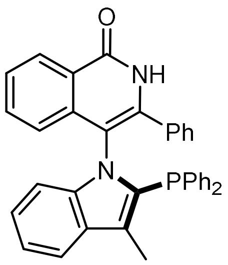

**4-(2-(diphenylphosphaneyl)-3-methyl-1H-indol-1-yl)-3-phenylisoquinolin-1(2H)-one (94)**

$^{1}H$ NMR (600 MHz, $CDCl_3$) $\delta$ 9.66 (s, 1H), 8.39 (d, J = 7.9 Hz, 1H), 7.63 (d, J = 7.9 Hz, 1H), 7.43 (t, J = 7.6 Hz, 1H), 7.37 (t, J = 7.6 Hz, 1H), 7.27 – 7.10 (m, 10H), 7.09 – 6.97 (m, 6H), 6.70 (t, J = 7.7 Hz, 2H), 6.49 (d, J = 8.0 Hz, 1H), 1.78 (s, 3H). $^{13}C$ NMR (150 MHz, $CDCl_3$) $\delta$ 163.0, 141.1(d, $J$ = 4.3 Hz), 140.9(d, $J$ =2.3 Hz), 137.9, 135.6 (d, $J$ = 7.8 Hz), 133.3, 132.7, 132.5, 132.4, 132.0 (d, $J$ = 18.5 Hz), 129.6, 129.6, 129.2, 128.7, 128.3 (d, $J$ = 2.9 Hz), 128.3 (d, $J$ = 2.7 Hz), 128.1 (d, $J$ = 118 Hz), 127.9, 128.8 (d, $J$ = 121.5 Hz), 127.7, 125.4, 125.2, 124.1, 123.0, 122.1 (d, $J$ = 7.8 Hz), 120.1, 119.5, 114.3, 111.1, 10.6. $^{31}P$ NMR (243 MHz, $CDCl_3$) $\delta$ -29.27.

**HRMS** (ESI) calculated for $C_{36}H_{27}N_2NaOP^+$ $[M+Na]^+$: 557.1753, found: 557.1759, $[\alpha]_D^{20}$ = 84 (c = 0.1, $CHCl_3$).

**HPLC conditions:** Daicel Chiralpak IE column (95:5 hexane: 2-propanol, 1.0 mL/min, 40 °C, 254nm); tr (major) = 16.76 min, tr (minor) = 19.20 min, 95% ee.

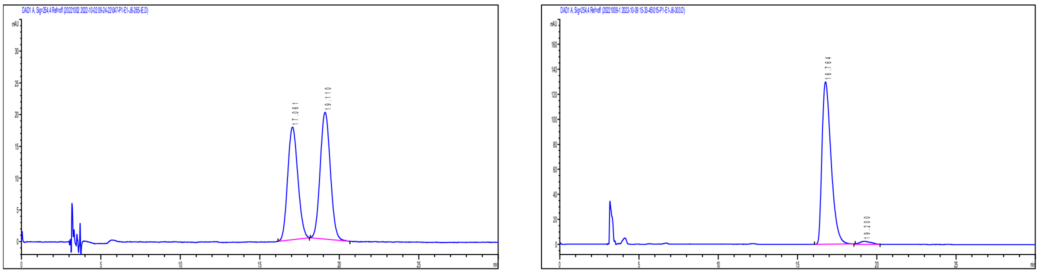

| No. | Time | Area | Area (%) | No. | Time | Area | Area (%) |
|---|---|---|---|---|---|---|---|
| 1 | 17.061 | 789.8 | 47.03 | 1 | 16.764 | 5174.7 | 97.69 |
| 2 | 19.110 | 889.7 | 52.97 | 2 | 19.200 | 122.3 | 2.31 |

**Supplementary Figure 115. HPLC data of 93.**

## 2.5 Determination of Rotation Barrier for Products 3, 37, 81.

The enantiomerisation barrier, corresponding to the barrier to rotation for the following atropisomers, was obtained by kinetic of racemisation of an enantiomer. The slope of the first order kinetic line gives the racemisation constant ($k_{racemisation}$ =2 × $k_{enantiomerisation}$). Eyring equation gives the enantiomerisation barrier ($\Delta G^{\ddagger}_{enaniomerization}$) from enantiomerisation constant ($k_{enantiomerisation}$), R = 8.31451 $J \cdot K^{-1} \cdot mol^{-1}$, $h$ = 6.62608 × $10^{-34}$ Js and $k_B$ = 1.38066 × $10^{-23}$ J/K. Reactions were conducted at 1 mg/mL concentration. The diastereomeric ratio (dr) and diastereomeirc excess (de) value were determined by HPLC.

$$\Delta G^{\ddagger}_{enantiomerization} = RT \times \ln \frac{k_B \times T}{h \times k_{enantiomerisation}}$$

**Supplementary Table 1.** Racemization of **3** in mesitylene at 120 °C.

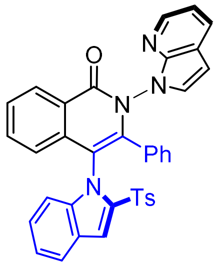


| T/s | dr | de | ln ($de_0/de_t$) |
|---|---|---|---|
| 0 | 99.5:0.5 | 99 | 0.000000 |
| 900 | 97.2:2.8 | 94.4 | 0.047578 |
| 1800 | 95.3:4.7 | 90.6 | 0.088666 |
| 3600 | 93.4:6.6 | 86.8 | 0.131513 |
| 5400 | 89.8:10.2 | 79.6 | 0.218091 |
| 7200 | 87.4:12.6 | 74.8 | 0.280230 |
| 9000 | 84.7:15.3 | 69.4 | 0.355224 |

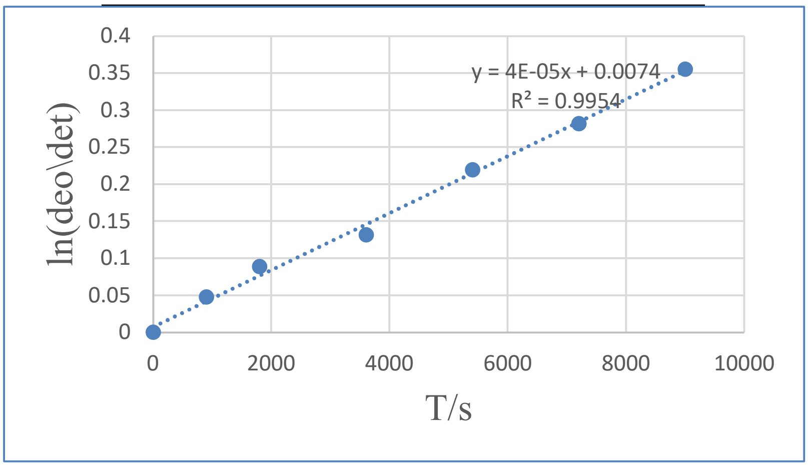


$k_{racemization}$ (120 °C) = $4.0\times10^{-5}$ $s^{-1}$

$k_{enantiomerization}$ (120 °C) = $2.0\times10^{-5}$ $s^{-1}$

Employing the Eyring equation: $\Delta G^{\ddagger}_{enantiomerization} = RT\times \ln\frac{k_B\times T}{hk_{enantiomerisation}}$

$$\Delta G^{\neq} = 8.314\ J \bullet K^{-1} \bullet mol^{-1} \times 393.15\ K \times \ln\frac{1.381\times10^{-23} J \bullet K^{-1} \times 393.15\ K}{2.0\times10^{-5}\ s^{-1} \times 6.626\times10^{-34}\ J \bullet s}$$

$$\Delta G^{\neq} = 132.6\ kJ \bullet mol^{-1} = 31.7\ kcal \bullet mol^{-1}$$

**Supplementary Table 2.** Racemization of **37** in *i*-PrOH at 60 °C.

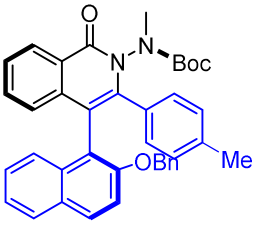

**37.** $\Delta G^{\ddagger}$ = 28.7 kcal/mol (60 °C, *i*-PrOH)

| T/s | dr | de | ln ($de_0/de_t$) |
|---|---|---|---|
| 0 | 95:5 | 90 | 0.000000 |
| 3600 | 93.6:6.4 | 87.2 | 0.031605 |
| 7200 | 93:7 | 86 | 0.045462 |
| 14400 | 92.5:7.5 | 85 | 0.057158 |
| 21600 | 92.3:7.7 | 84.6 | 0.061875 |
| 25200 | 92:8 | 84 | 0.068993 |
| 28800 | 91.7:8.3 | 83.4 | 0.076161 |
| 36000 | 91.3:8.7 | 82.6 | 0.085800 |
| 72000 | 88.7:11.3 | 77.4 | 0.150823 |
| 86400 | 88:12 | 76 | 0.169076 |

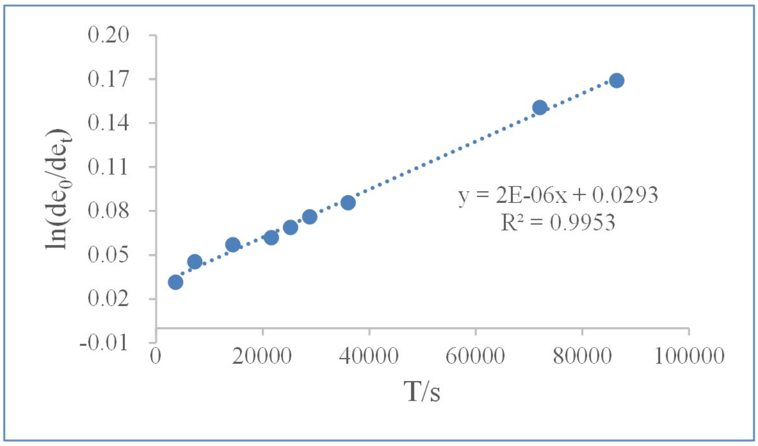


$k_{racemization}$ (60 °C) = $2.0\times10^{-6}$ $s^{-1}$

$k_{enantiomerization}$ (60 °C) = $1.0\times10^{-6}$ $s^{-1}$

Employing the Eyring equation: $\Delta G^{\ddagger}_{enantiomerization} = RT\times \ln\frac{k_B\times T}{hk_{enantiomerisation}}$

$$\Delta G^{\neq} = 8.314\ J\bullet K^{-1}\bullet mol^{-1} \times 333.15\ K \times \ln\frac{1.381\times10^{-23} J\bullet K^{-1} \times 333.15\ K}{1.0\times10^{-6}\ S^{-1}\times 6.626\times10^{-34}\ J\bullet s}$$

$$\Delta G^{\neq} = 120.2\ kJ\bullet mol^{-1} = 28.7\ kcal\bullet mol^{-1}$$

**Supplementary Table 3.** Racemization of **81** in mesitylene at 120 °C.

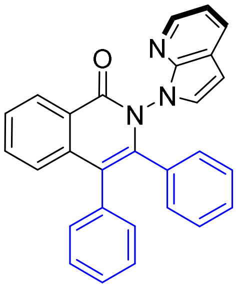


| T/s | er | ee | ln ($ee_0/ee_t$) |
|---|---|---|---|

| | | | |
|---|---|---|---|
| 0 | 96.6:3.4 | 93.2 | 0.000000 |
| 1800 | 96.3:3.7 | 92.6 | 0.006458 |
| 3600 | 94.8:5.2 | 89.6 | 0.039393 |
| 7200 | 94.3:5.7 | 88.6 | 0.050616 |
| 10800 | 92.9:7.1 | 85.8 | 0.083894 |
| 14400 | 91.3:8.7 | 82.6 | 0.121949 |
| 18000 | 90.0:10.0 | 80.0 | 0.153972 |
| 25200 | 87.2:12.8 | 74.4 | 0.225292 |

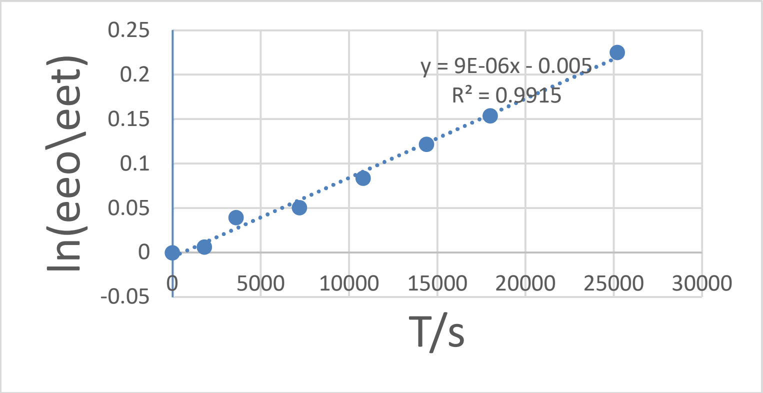


$k_{racemization}$ (120 °C) = $9.0\times10^{-6}$ $s^{-1}$

$k_{enantiomerization}$ (120 °C) = $4.5\times10^{-6}$ $s^{-1}$

Employing the Eyring equation: $\Delta G^{\ddagger}_{enantiomerization} = RT\times \ln\frac{k_B\times T}{hk_{enantiomerisation}}$

$$\Delta G^{\neq} = 8.314\ J \bullet K^{-1} \bullet mol^{-1} \times 393.15\ K \times \ln\frac{1.381\times10^{-23} J\bullet K^{-1} \times 393.15\ K}{4.5\times10^{-6}\ S^{-1}\times 6.626\times10^{-3}\ \ J\bullet s}$$

$$\Delta G^{\neq} = 137.4\ kJ \bullet mol^{-1} = 32.8\ kcal \bullet mol^{-1}$$

## 2.6 Mechanistic Studies.

**Deconvolution of the Three Selectivity Parameters.**[7]

$r_{CC}$ = (S-R)/(S+R)

$$ee_a = \frac{(1+r_1)(1+r_{cc})-(1-r_2)(1-r_{cc})}{(1+r_1)(1+r_{cc})+(1-r_2)(1-r_{cc})}$$

$$ee_b = \frac{(1-r_1)(1+r_{cc})-(1+r_2)(1-r_{cc})}{(1-r_1)(1+r_{cc})+(1+r_2)(1-r_{cc})}$$

**Supplementary Figure 116.** Derivation of the Three Selectivity Parameters.

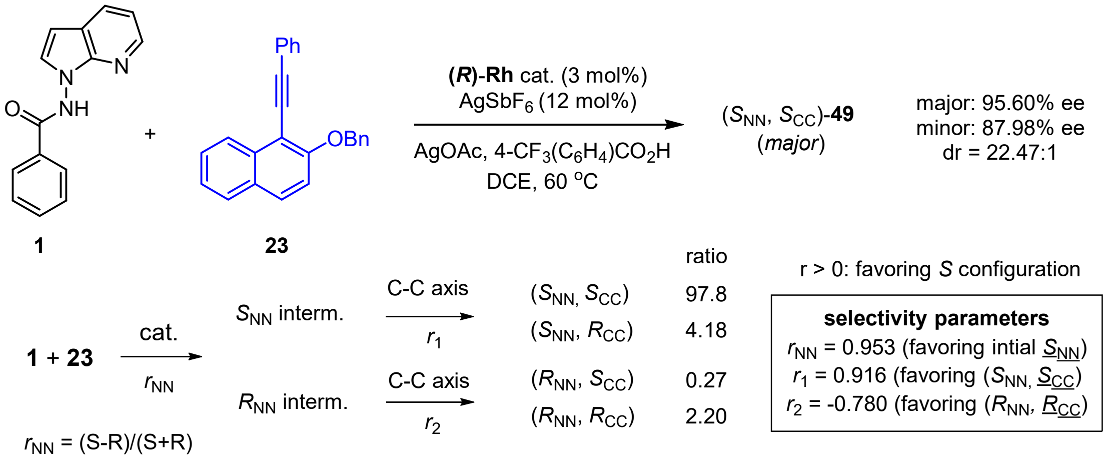


$r_{NN} = 0.953$

$$0.9560\ (\text{ee major}) = \frac{(1+r_1)(1+0.953)-(1-r_2)(1-0.953)}{(1+r_1)(1+0.953)+(1-r_2)(1-0.953)}$$

$$0.8798\ (\text{ee minor}) = \frac{(1-r_1)(1+0.953)-(1+r_2)(1-0.953)}{(1-r_1)(1+0.953)+(1+r_2)(1-0.953)}$$

$r_1 = 0.916$, $r_2 = -0.780$

----------------------------------------

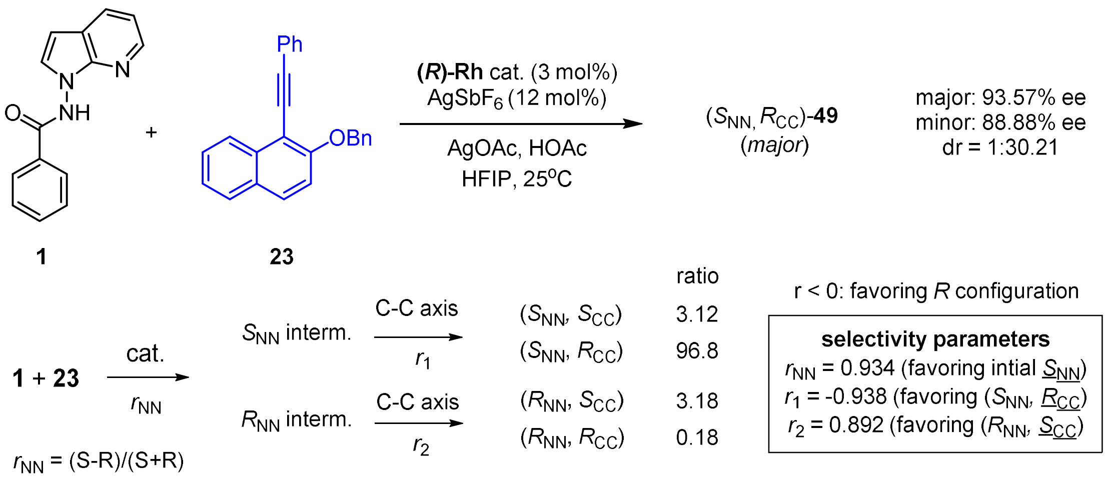


$r_{NN} = 0.934$

$$0.9357 = \frac{(1+r_1)(1+0.934)-(1-r_2)(1-0.934)}{(1+r_1)(1+0.934)+(1-r_2)(1-0.934)}$$

$$0.8888 = \frac{(1-r_1)(1+0.934)-(1+r_2)(1-0.934)}{(1-r_1)(1+0.934)+(1+r_2)(1-0.934)}$$

$r_1 = -0.938$, $r_2 = 0.892$

## Parallel Experiments for KIE.

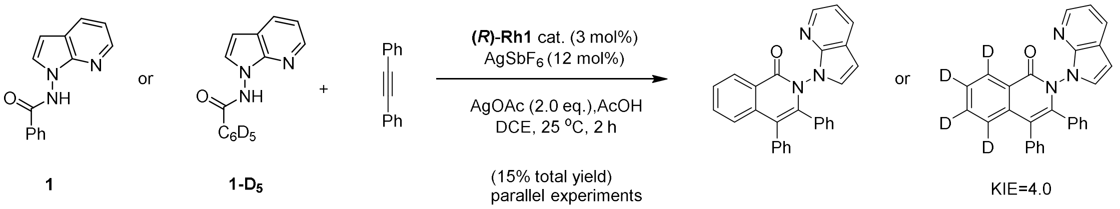


Two glass vials each was charged under $N_2$ with **1** (23.7 mg, 0.1 mmol, 1.0 equiv) or **1-D₅** (23.7 mg, 0.1 mmol, 1 equiv), 1,2-diphenylethyne (17.8 mg, 0.1 mmol, 1.0 equiv), **(*R*)-Rh-1** (3.5 mg, 3 mol%), $AgSbF_6$ (4.2 mg, 12 mol%), AgOAc (33.4 mg, 0.2 mmol, 2.0 equiv), and AcOH (6.0 mg, 0.1 mmol, 1.0 equiv). DCE (1 mL) was then added and the mixture was stirred at 25 °C for 2 h. The reaction tubes were quenched by cooling in ice-water. The two mixtures were combined and were rapidly evaporated under reduced pressure separately. The purification was performed by flash column chromatography on silica gel (eluent: PE/EA = 2:1) to afford the product(s). The KIE value was determined to be $k_H/k_D$ = 4.0 on the basis of $^1$H NMR analysis.

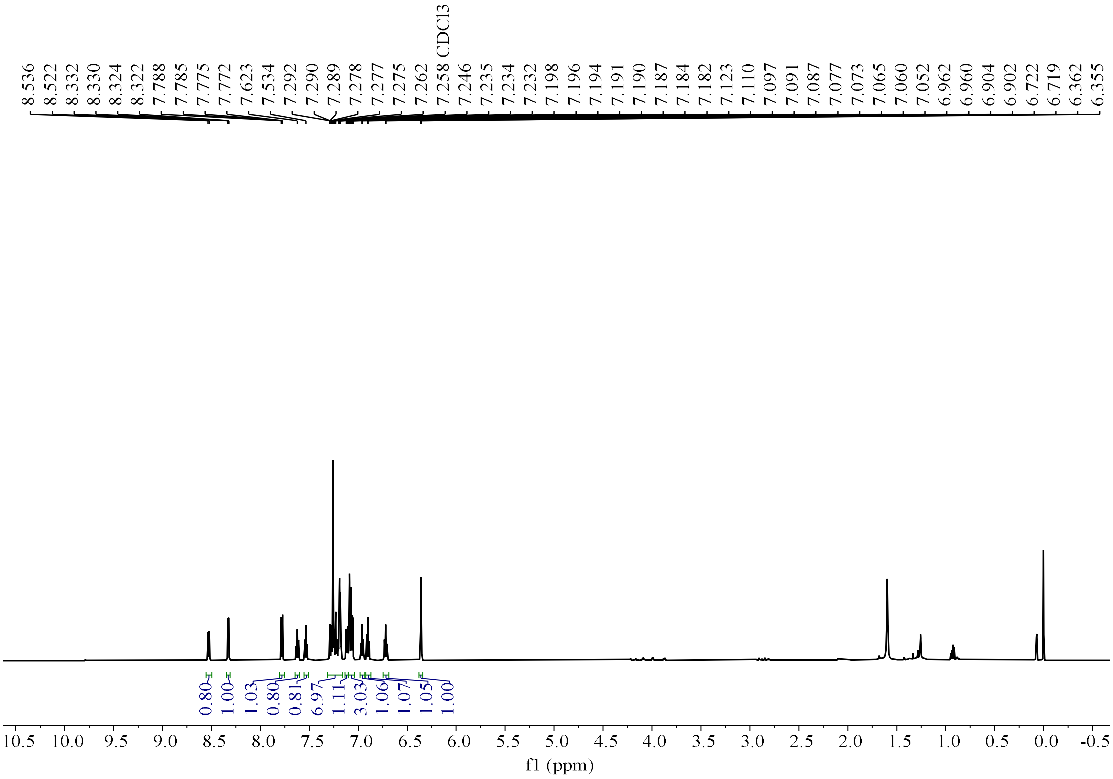

**Supplementary Figure 117. $^1$H NMR (600 MHz, $CDCl_3$) spectrum of KIE.**

## Rotamers of the amide bond (removal of Boc)

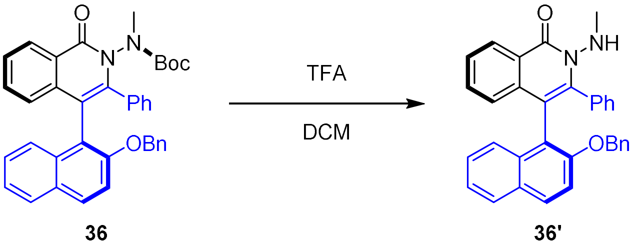


A scew-cap vial (8 mL) was charged with (*S,S*)-tert-butyl (4-(2-(benzyloxy)naphthalen-1-yl)-1-oxo-3-phenylisoquinolin-2(1H)-yl)(methyl)carbamate **36** (0.1 mmol, 1.0 equiv), TFA (1.0 mmol, 10.0 equiv),

and DCM (2 mL) was then added and the mixture was stirred at 30 °C for 12 h. The reaction mixture was evaporated under vacuum and the residue was purified by preparative TLC to give the product **36'** with the Boc removed. **$^1$H NMR (600 MHz, $CDCl_3$)** δ 8.59 (d, *J* = 8.1 Hz, 1H), 7.71 (d, *J* = 8.8 Hz, 2H), 7.50 (t, *J* = 7.4 Hz, 1H), 7.47 – 7.40 (m, 2H), 7.35 – 7.26 (m, 4H), 7.25 - 7.21 (m, 3H), 7.14 (d, *J* = 9.0 Hz, 1H), 7.12 – 7.03 (m, 4H), 6.99 (s, 1H), 6.87 (d, *J* = 8.2 Hz, 2H), 5.04 (s, 2H), 2.53 (s, 3H).
**HRMS (ESI)**: calcd. for $C_{33}H_{26}N_2NaO_2^+$ $[M+Na]^+$ : 505.1886, found : 505.1884 . No signal broaden was observed except the NH.

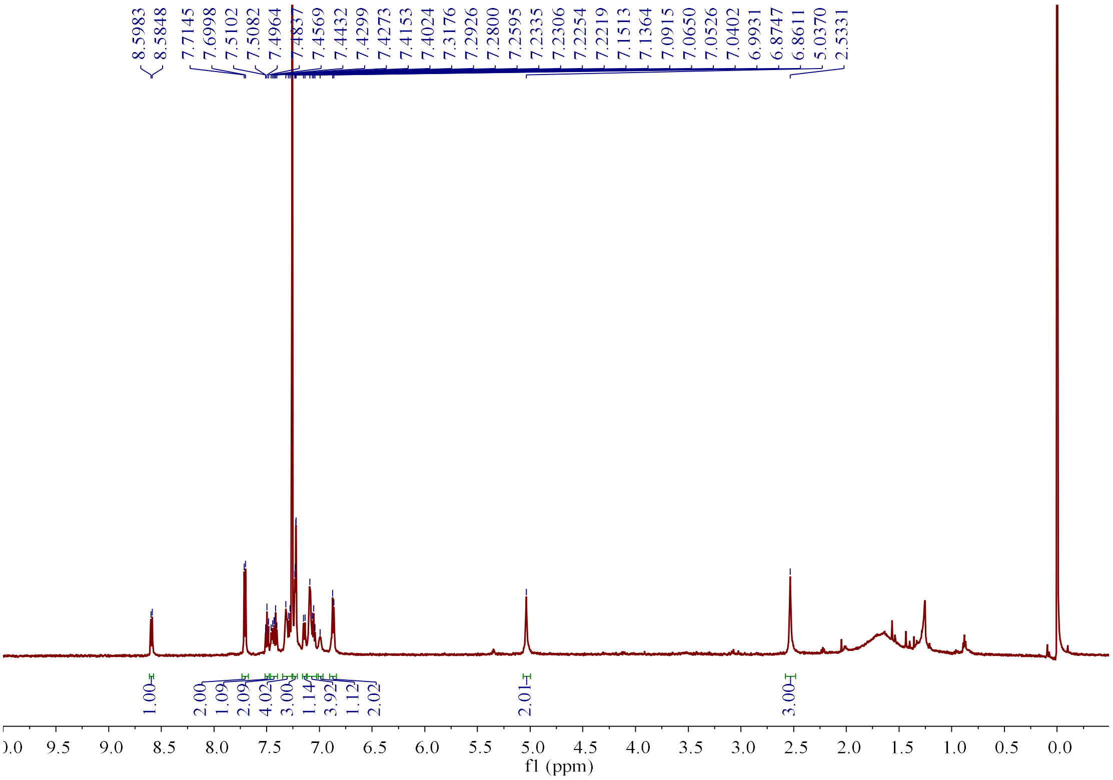


**Supplementary Figure 118.$^1$H NMR (600 MHz, $CDCl_3$) spectrum of 36'.**

Furthermore, variable-temperature (VT) NMR analysis of compound **24** were conducted to gain more insight into the dynamic exchange process for the different two rotamers. As shown below, two sets of signals of δ 8.50 - 8.46 ppm, 5.05 – 4.98 ppm broaden when measured at 328K.

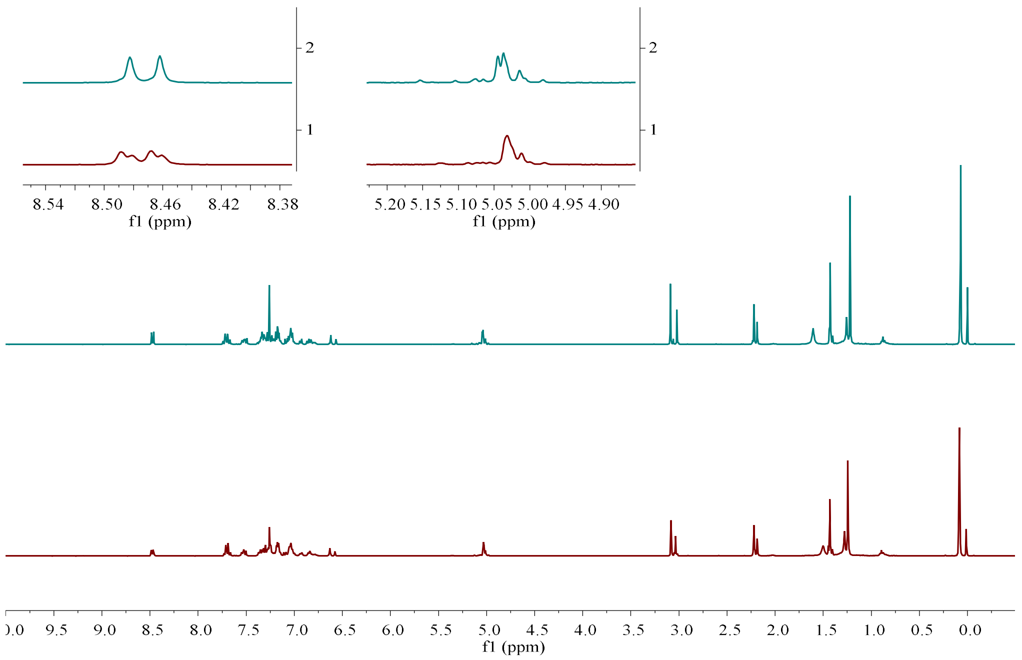


**Supplementary Figure 119. $^1$H NMR (400 MHz, CDCl$_3$) spectra of product 24 in 298 K (up) and 328 K (down).**

## Control experiment

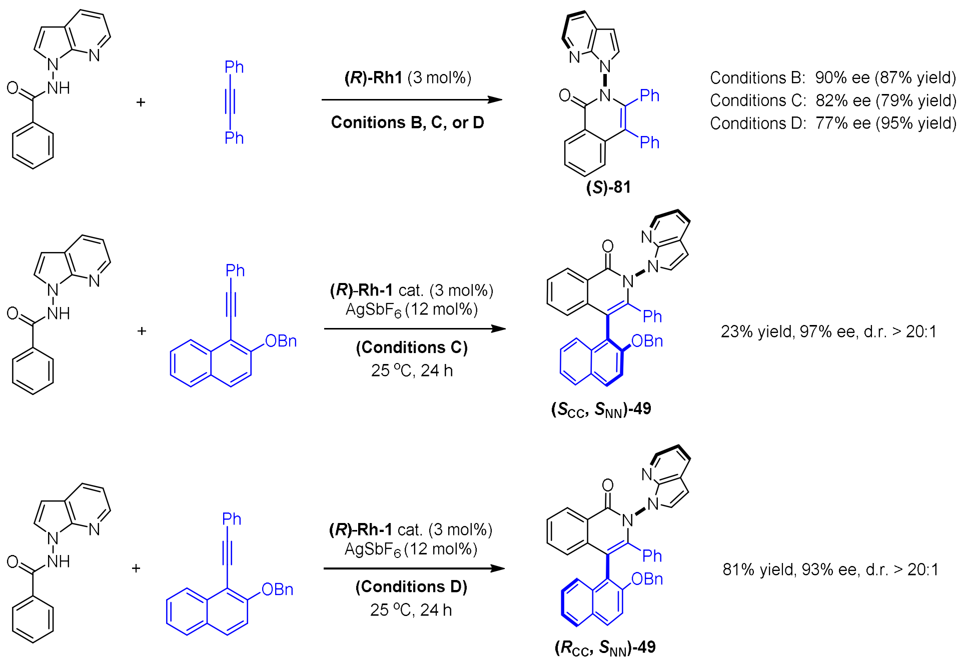


**Supplementary Figure 120. Control Experiments to Explore the Axial Chirality**

**(1) Diphenylacetylene was used as an alternative coupling reagent.**

**Conditions B.** A scew-cap vial (8 mL) was charged with N-(7-azaindol-1-yl)benzamide **1** (23.7 mg, 0.1 mmol, 1.0 equiv), diphenylacetylene (0.1 mmol, 1.0 equiv), (R)-Rh-1 (3 mol%), AgOAc (0.2 mmol, 2.0 equiv), HOPiv (0.2 mmol, 2.0 equiv) and MeOH (2 mL) was then added, and the mixture was stirred at 40 °C for 48 h under air. The reaction mixture was evaporated under vacuum and the residue was purified by preparative TLC to give the corresponding product. The enantiomeric excess was determined by chiral HPLC analysis.

**Conditions C.** A scew-cap vial (8 mL) was charged with N-(7-azaindol-1-yl)benzamide **1** (23.7 mg, 0.1 mmol, 1.0 equiv), diphenylacetylene (0.1 mmol, 1.0 equiv), **(*R*)-Rh-1** (3.5 mg, 3 mol%), $AgSbF_6$ ( 4.2 mg, 12 mol%), AgOAc (33.4 mg, 0.2 mmol, 2.0 equiv), and 4-$CF_3$-$C_6H_4CO_2H$ (19.0 mg, 0.1 mmol, 1.0 equiv). DCE (1 mL) was then added, and the mixture was stirred at 60 °C for 48 h. The reaction mixture was evaporated under vacuum and the residue was purified by preparative TLC to give the corresponding product.

**Conditions D.** A scew-cap vial (8 mL) was charged with N-(7-azaindol-1-yl)benzamide **1** (23.7 mg, 0.1 mmol, 1.0 equiv), diphenylacetylene (0.1 mmol, 1.0 equiv), **(*R*)-Rh-1** (3.5 mg, 3 mol%), $AgSbF_6$ ( 4.2 mg, 12 mol%), AgOAc (33.4 mg, 0.2 mmol, 2.0 equiv), and AcOH (6.0 mg, 0.1 mmol, 1.0 equiv). HFIP (1 mL) was then added and the mixture was stirred at 25 °C for 24 h. The reaction mixture was evaporated under vacuum and the residue was purified by preparative TLC to give the corresponding product.

**Racemic Synthesis of the Above Biaryl Products**. A similar synthetic procedure was followed for synthesis of racemic products except that racemic **Rh-1** (3 mol %) was used.

**(*S*)-3,4-diphenyl-2-(1H-pyrrolo[2,3-b]pyridin-1-yl)isoquinolin-1(2H)-one (81)**

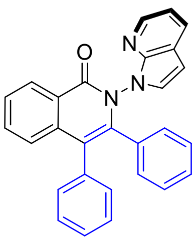

White solid (35.9 mg, 87%), $^1$H NMR (600 MHz, $CDCl_3$) δ 8.52 (dd, *J* = 8.0, 1.8 Hz, 1H), 8.32 (dd, *J* = 4.7, 1.9 Hz, 1H), 7.76 (dd, *J* = 7.8, 1.8 Hz, 1H), 7.63 – 7.56 (m, 1H), 7.51 (t, *J* = 7.7 Hz, 1H), 7.31 – 7.14 (m, 6H), 7.08-7.11 (m, 3H), 7.04 (ddd, *J* = 7.5, 4.7, 1.8 Hz, 1H), 6.95 (t, *J* = 7.6 Hz, 1H), 6.91 – 6.80 (m, 1H), 6.70 (t, *J* = 7.6 Hz, 1H), 6.34 (dd, *J* = 3.9, 1.8 Hz, 1H). $^{13}$C NMR (151 MHz, $CDCl_3$) δ 161.06, 147.29, 143.84, 142.01, 137.63, 135.61, 133.19, 132.23, 131.55, 131.31, 129.83, 129.74, 129.36, 128.76, 128.60, 128.23, 128.11, 127.92, 127.23, 127.16, 126.94, 126.92, 126.02, 125.34, 119.31, 118.58, 117.10, 100.53.

**HRMS** (ESI-TOF) (m/z): Calcd for $C_{28}H_{20}N_3O^+$, ($[M + H]^+$), 414.1601, found 414.1603. $[\alpha]_D^{20}$ = -12 (c = 0.1, $CHCl_3$).

**HPLC** conditions: Daicel Chiralpak OD-3 column (90: 10 hexane: 2-propanol, 0.8 mL/min, 40 °C, 254 nm)

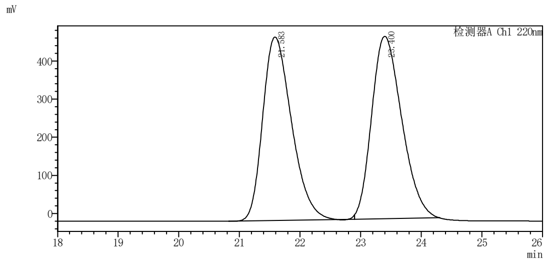

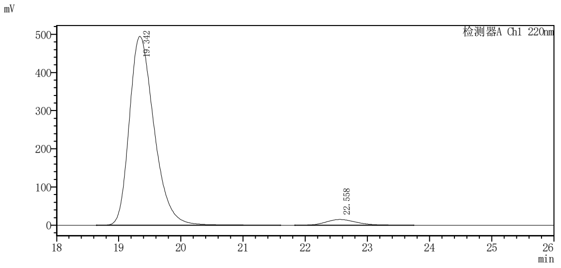

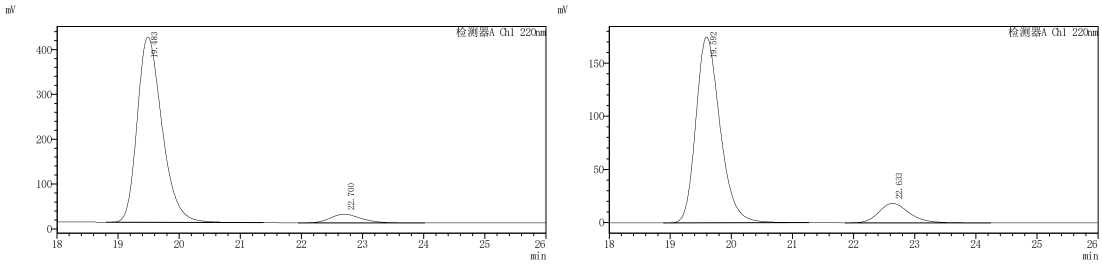

| No. | Time | Area | Area (%) | No. | Time | Area | Area (%) |
|---|---|---|---|---|---|---|---|
| 1 | 21.583 | 15941343 | 49.068 | 3 | 19.342 | 17785652 | 94.757 |
| 2 | 23.400 | 16547208 | 50.932 | 4 | 22.558 | 984098 | 5.243 |
| 5 | 19.483 | 13174452 | 90.924 | 7 | 19.592 | 7634329 | 88.768 |
| 6 | 22.700 | 1315069 | 9.076 | 8 | 22.633 | 965980 | 11.232 |

**Supplementary Figure 121. HPLC data of 81.**

### (2) Exclude the effect of temperature and reaction time

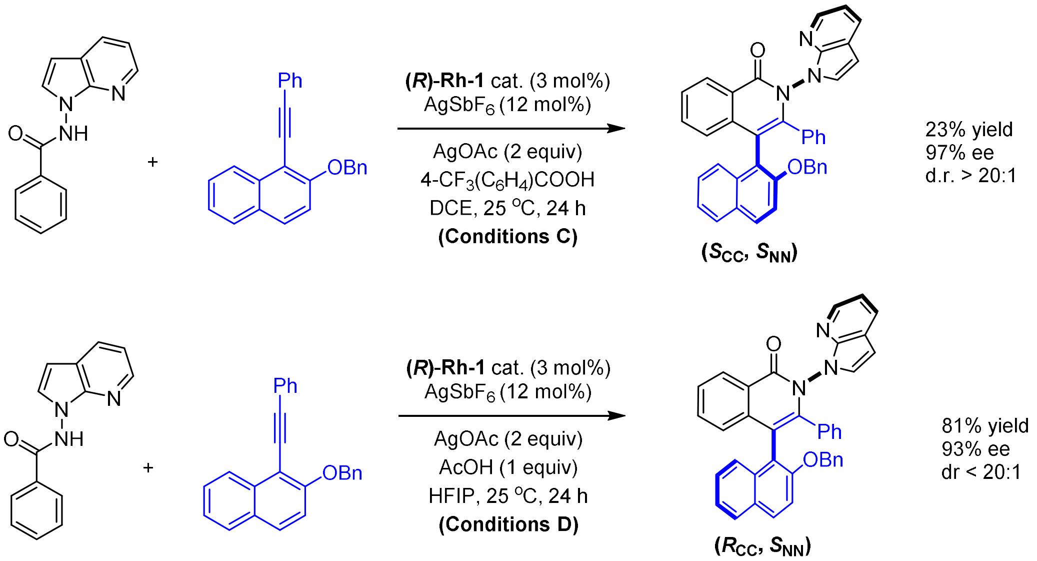


**Conditions C.** A scew-cap vial (8 mL) was charged with N-(7-azaindol-1-yl)benzamide **1** (23.7 mg, 0.1 mmol, 1.0 equiv), 2-substituted 1-alkynylnaphthalene **23** (0.1 mmol, 1.0 equiv), **(*R*)-Rh-1** (3.5 mg, 3 mol%), $AgSbF_6$ ( 4.2 mg, 12 mol%), AgOAc (33.4 mg, 0.2 mmol, 2.0 equiv), and 4-$CF_3$-$C_6H_4CO_2H$ (19.0 mg, 0.1 mmol, 1.0 equiv). DCE (1 mL) was then added, and the mixture was stirred at 25 °C for 24 h. The reaction mixture was evaporated under vacuum and the residue was purified by preparative TLC to give the corresponding product.

**Conditions D.** A scew-cap vial (8 mL) was charged with N-(7-azaindol-1-yl)benzamide **1** (23.7 mg, 0.1 mmol, 1.0 equiv), 2-substituted 1-alkynylnaphthalene **23** (37.1 mg, 0.1 mmol, 1.0 equiv), **(*R*)-Rh-1** (3.5 mg, 3 mol%), $AgSbF_6$ ( 4.2 mg, 12 mol%), AgOAc (33.4 mg, 0.2 mmol, 2.0 equiv), and AcOH (6.0 mg, 0.1 mmol, 1.0 equiv). HFIP (1 mL) was then added and the mixture was stirred at 25 °C for 24 h. The reaction mixture was evaporated under vacuum and the residue was purified by preparative TLC to give the corresponding product.

## Theoretical calculation

All the geometry optimizations were performed at the B3LYP-D3(BJ)[8]/def2-svp[9] level of theory using the Gaussian 09[10]. Frequencies were computed analytically at the same level of theory to confirm

whether the structures are minima (no imaginary frequencies) or transition states (only one imaginary frequency). Solvation effects (DCE, ε = 10.125 and HFIP, ε = 16.7) were taken into account by performing single-point calculations using the SMD model.[11] Since the solvent parameters for HFIP are not available in Gaussian, the solvent parameters of isopropanol were used and the dielectric constant of the solvent was modified to the dielectric constant of HFIP. Key transition-state structures were confirmed to connect the correct reactants and products by intrinsic reaction coordinate (IRC) calculations.[12] To obtain better accuracy, single-point energies for the optimized geometries were calculated at the PWPB95-D4[13]/def2-TZVPP[14] level of theory using ORCA 5.0.3[15] (with auxiliary basis set def2/J[16] and def2-TZVPP/C[17]). The RIJCOSX approximation was used to accelerate the calculations. To correct the Gibbs free energies under pressure of 1 atm to the standard state in solution (1 mol/L), a correction of RT $\ln(c_s/c_g)$ (1.9 kcal/mol) is added to energies of all species except HFIP. $c_s$ is the standard molar concentration in solution (1 mol/L), $c_g$ is the standard molar concentration in gas phase (0.0446 mol/L), and R is the gas constant. 3.2 kcal/mol free energy correction on HFIP solvent molecule was included based on the concentration of pure HFIP solvent under the standard state (9.45 mol/L). The final Gibbs free energies reported in the article are the single-point energies corrected by gas-phase Gibbs free energy correction (at 298.15 K), solvent effects and standard state corrections.

## Additional results for the reaction in DCE

## (a) C-H bond activation (R = 4-$CF_3C_6H_4$)

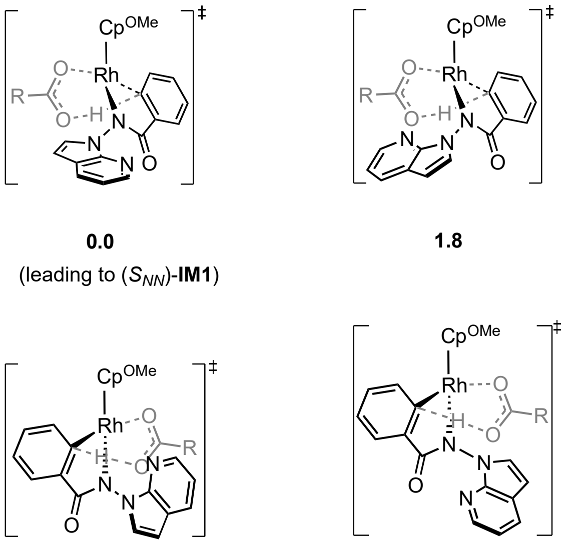


$\Delta\Delta G_{DCE}$ kcal/mol — **0.0** (leading to ($S_{NN}$)-**IM1**); **1.8**

**5.7** (leading to ($S_{NN}$)-**IM1'**); **4.8**

**Supplementary Figure 122.**

## (b) N-N axis rotation

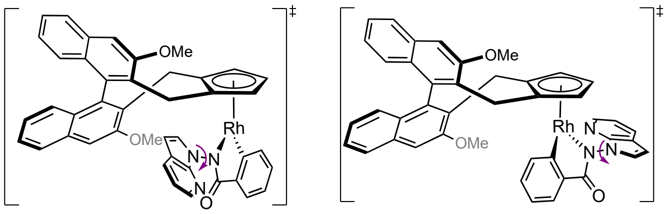


$\Delta G_{DCE}$ kcal/mol — **22.8**; **27.3**

**Supplementary Figure 123.**

## Direct insertion of alkyne 23 into the Rh-C bond of ($S_{NN}$)-IM1

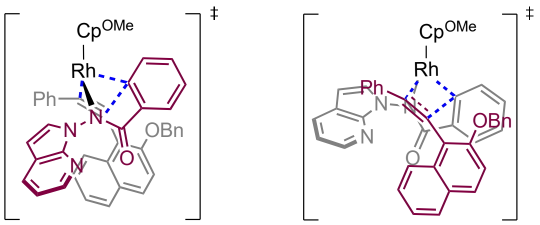


$\Delta G_{DCE}$ kcal/mol — **17.1**; **14.4**

**Supplementary Figure 124.**

## Additional results for the reaction in HFIP

## (a) C-H bond activation

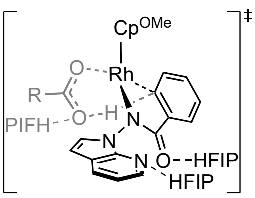


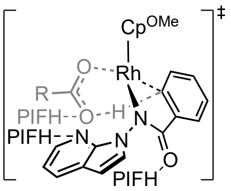


| | | |
|---|---|---|
| $\Delta\Delta G_{HFIP}$ kcal/mol | **0.0** (leading to ($S_{NN}$)-**IM2**) | **5.0** |

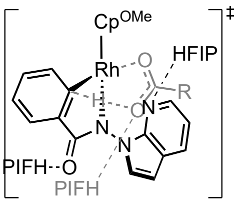


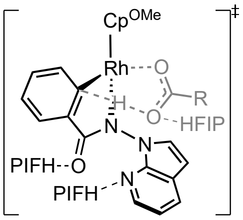


**6.1** **7.1**

**Supplementary Figure 125.**

## (c) Rotation along the Rh-Cp bond

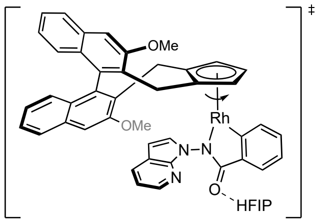


$\Delta G_{HFIP}$ kcal/mol **15.5**

**Supplementary Figure 126.**

## (c) N-N axis rotation

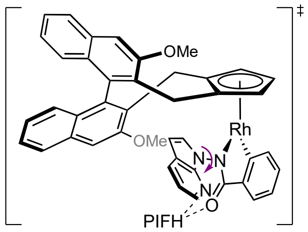


$\Delta G_{HFIP}$ kcal/mol **26.7**

**Supplementary Figure 127.**

## Comparisons of the Transition States Involving Lewis Acid Bindng or Hydrogen Bonding with Other Additives

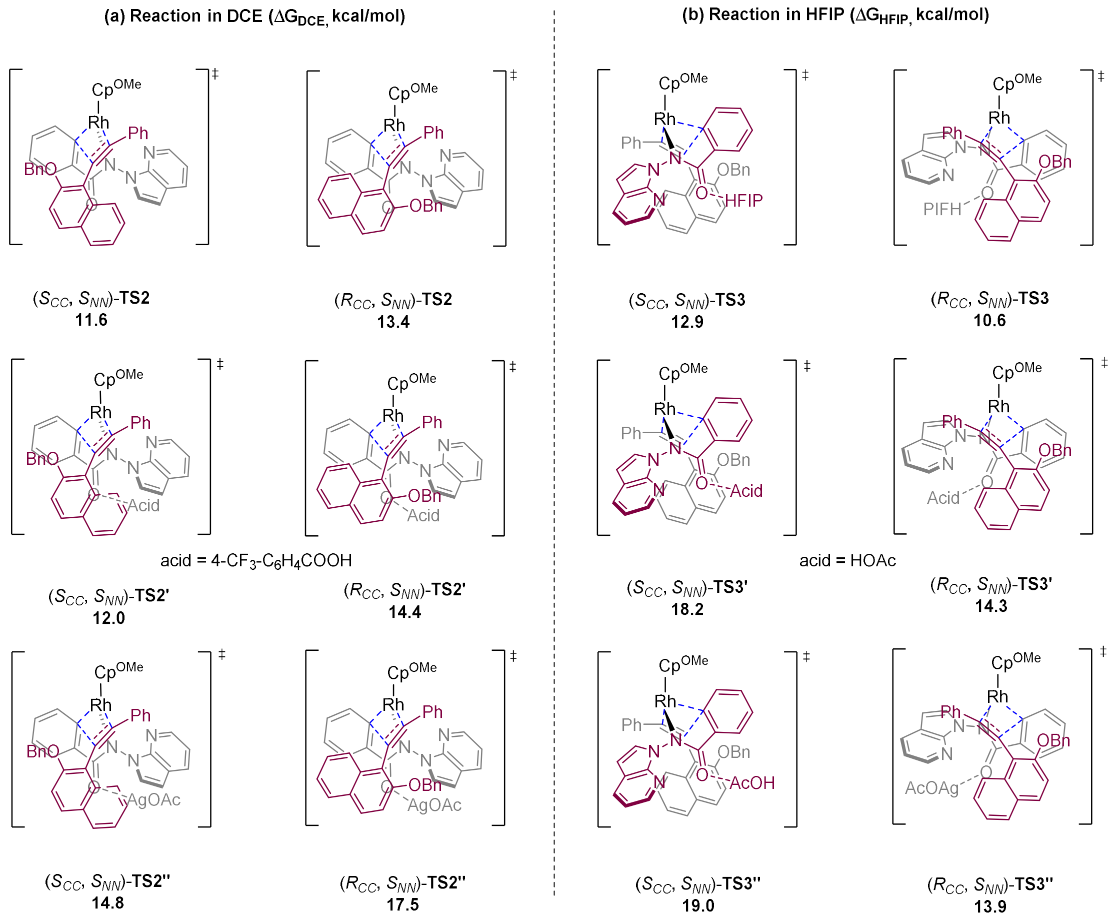


**Supplementary Figure 128. Comparisons of the Transition States Involving Lewis Acid Bindng or Hydrogen Bonding with Other Additives**

## Supplementary Table 4: Calculated energies and energy corrections

| Stationary point | Single-point energy PWPB95-D4 def2-TZVPP (a.u.) | Thermal correction to Gibbs free energy at 298.15 K (a.u.) | Solvent effect (a.u.) |
|---|---|---|---|
| **23** | -1038.299856 | 0.299315 | -0.029196 (DCE)<br>-0.023330 (HFIP) |
| ($S_{NN}$)-**TS1** | -2157.618159 | 0.574797 | -0.054373 |
| ($S_{NN}$)-**IM1** | -2157.644012 | 0.576137 | -0.049145 |
| ($S_{NN}$)-**IM1'** | -2157.637547 | 0.574548 | -0.049057 |
| ($S_{CC}$,$S_{NN}$)-**TS2** | -3195.966008 | 0.904265 | -0.063500 |
| ($S_{CC}$,$S_{NN}$)-**IM3** | -3195.988757 | 0.905470 | -0.060595 |
| ($S_{CC}$,$S_{NN}$)-**IM4** | -3196.003272 | 0.907017 | -0.061331 |

| | | | |
|---|---|---|---|
| ($R_{CC}$,$S_{NN}$)-**TS2** | -3195.965785 | 0.903863 | -0.060403 |
| ($R_{CC}$,$S_{NN}$)-**IM3** | -3195.990979 | 0.904139 | -0.061090 |
| ($R_{CC}$,$S_{NN}$)-**IM4** | -3196.004289 | 0.907204 | -0.060656 |
| ($S_{NN}$)-**IM2** | -3737.259347 | 0.679278 | -0.042862 |
| ($R_{CC}$,$S_{NN}$)-**TS3** | -3985.773650 | 0.954884 | -0.056275 |
| ($R_{CC}$,$S_{NN}$)-**IM5** | -3985.800367 | 0.957219 | -0.054823 |
| ($R_{CC}$,$S_{NN}$)-**IM6** | -3985.804252 | 0.956079 | -0.057322 |
| ($S_{CC}$,$S_{NN}$)-**TS3** | -3985.763878 | 0.952087 | -0.059700 |
| ($S_{CC}$,$S_{NN}$)-**IM5** | -3985.787927 | 0.952658 | -0.058446 |
| ($S_{CC}$,$S_{NN}$)-**IM6** | -3985.792238 | 0.957220 | -0.058352 |

## Photophysical properties of product

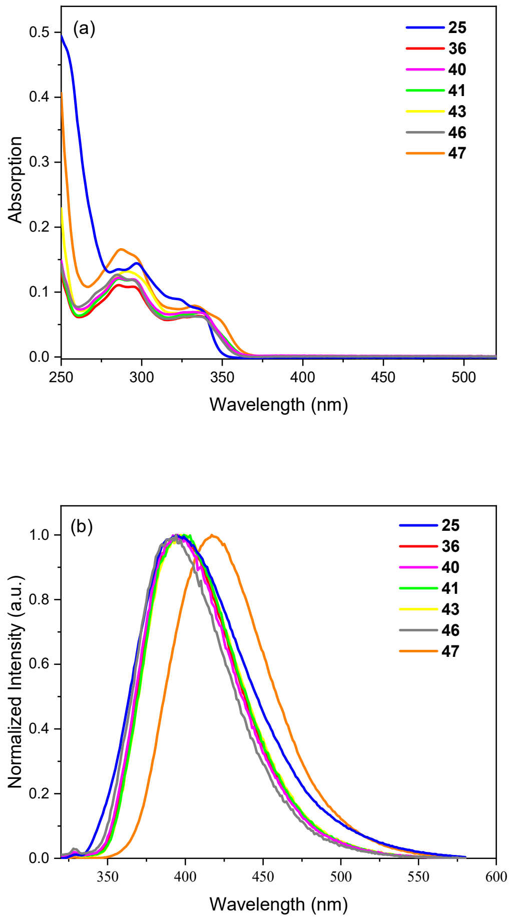


**Supplementary Figure 129.** [a] absorption (left) and [b] emission spectra (right) of **25**, **36**, **40**, **41**, **43**, **46** and **47** in DCM (1×10$^{-5}$ M).

**Supplementary Table 5**: Photophysical properties of selected products in DCM (1×10$^{-5}$ M)

| Compound | $\lambda_{abs}$[a][nm] | $\lambda_{em}$[b][nm] | $\Phi_F$[c] |
|---|---|---|---|
| **25** | 298, 324 (sh), 337 | 393 | 0.08 |
| **36** | 285, 296, 338 | 396 | 0.09 |
| **40** | 285, 296, 337 | 393 | 0.08 |
| **41** | 285, 296, 338 | 398 | 0.12 |
| **43** | 291, 337 | 398 | 0.12 |
| **46** | 284, 296, 334 | 394 | 0.10 |
| **47** | 287, 297 (sh), 333 | 418 | 0.47 |

[a] Absorption maxima. [b] Fluorescent emission maxima. [c] Absolute quantum yields (determined with an integrating sphere system).

## 2.7 X-Ray Crystallographic Data

## (1)X-ray crystal structure of (*S,S*)-8 (CCDC 2215068)

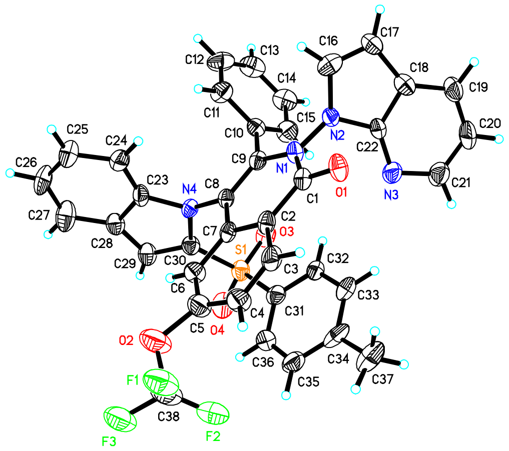


| | | |
|---|---|---|
| Identification code | **(*S,S*)-8** | |
| Empirical formula | C38 H25 F3 N4 O4 S | |
| Formula weight | 690.68 | |
| Temperature | 213.15 K | |
| Wavelength | 0.71073 Å | |
| Crystal system | Orthorhombic | |
| Space group | P 21 21 2 | |
| Unit cell dimensions | a = 26.0177(8) Å | $\alpha$= 90°. |
| | b = 28.5298(8) Å | $\beta$= 90°. |
| | c = 13.7719(4) Å | $\gamma$ = 90°. |
| Volume | 10222.6(5) $Å^3$ | |
| Z | 12 | |
| Density (calculated) | 1.346 $Mg/m^3$ | |
| Absorption coefficient | 0.158 $mm^{-1}$ | |
| F(000) | 4272 | |
| Crystal size | 0.2 x 0.16 x 0.12 $mm^3$ | |
| Theta range for data collection | 2.154 to 26.000°. | |
| Index ranges | -27<=h<=32, -32<=k<=35, -16<=l<=16 | |
| Reflections collected | 51819 | |
| Independent reflections | 20006 [R(int) = 0.0662] | |
| Completeness to theta = 25.242° | 99.8 % | |
| Absorption correction | Semi-empirical from equivalents | |
| Max. and min. transmission | 0.7456 and 0.5115 | |
| Refinement method | Full-matrix least-squares on $F^2$ | |

| | |
|---|---|
| Data / restraints / parameters | 20006 / 327 / 1444 |
| Goodness-of-fit on $F^2$ | 1.064 |
| Final R indices [I>2sigma(I)] | R1 = 0.0716, wR2 = 0.1700 |
| R indices (all data) | R1 = 0.1296, wR2 = 0.2072 |
| Absolute structure parameter | 0.00(5) |
| Extinction coefficient | n/a |
| Largest diff. peak and hole | 0.524 and -0.518 e.$Å^{-3}$ |

## (2)X-ray crystal structure of (*S,S*)-47 (CCDC 2213484)

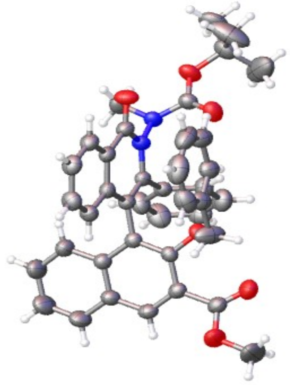

| | |
|---|---|
| Identification code | **47** |
| Empirical formula | C40H36N2O6 |
| Formula weight | 640.71 |
| Temperature/K | 213(2) |
| Crystal system | orthorhombic |
| Space group | P212121 |
| a/Å | 9.0675(5) |
| b/Å | 11.5772(6) |
| c/Å | 32.5606(17) |
| α/° | 90 |
| β/° | 90 |
| γ/° | 90 |
| Volume/Å3 | 3418.1(3) |
| Z | 4 |
| $\rho_{calc}$g/cm$^3$ | 1.245 |

| | |
|---|---|
| μ/mm$^{-1}$ | 0.678 |
| F(000) | 1352.0 |
| Crystal size/mm$^3$ | 0.2 × 0.16 × 0.13 |
| Radiation | CuKα (λ = 1.54178) |
| 2Θ range for data collection/° | 8.106 to 134.99 |
| Index ranges | -10 ≤ h ≤ 10, -13 ≤ k ≤ 13, -39 ≤ l ≤ 35 |
| Reflections collected | 32159 |
| Independent reflections | 6086 [$R_{int}$ = 0.0550, $R_{sigma}$ = 0.0412] |
| Data/restraints/parameters | 6086/0/439 |
| Goodness-of-fit on $F^2$ | 1.061 |
| Final R indexes [I>=2σ (I)] | $R_1$ = 0.0407, $wR_2$ = 0.1064 |
| Final R indexes [all data] | $R_1$ = 0.0412, $wR_2$ = 0.1071 |
| Largest diff. peak/hole / e Å$^{-3}$ | 0.17/-0.17 |
| Flack parameter | 0.04(6) |

## (3)X-ray crystal structure of *dia*-59 (CCDC 2215071)

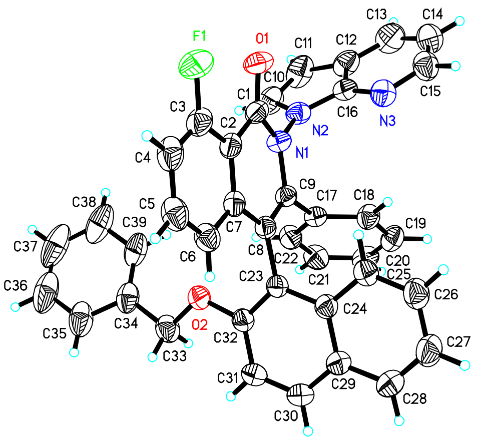


| | | |
|---|---|---|
| Identification code | cu_d8v22291_0m | |
| Empirical formula | C39 H26 F N3 O2 | |
| Formula weight | 587.63 | |
| Temperature | 293(2) K | |
| Wavelength | 1.54178 Å | |
| Crystal system | Orthorhombic | |
| Space group | P 21 21 21 | |
| Unit cell dimensions | a = 9.44380(10) Å | a= 90°. |
| | b = 16.8611(2) Å | b= 90°. |
| | c = 19.3329(3) Å | g = 90°. |
| Volume | 3078.43(7) $Å^3$ | |
| Z | 4 | |
| Density (calculated) | 1.268 $Mg/m^3$ | |
| Absorption coefficient | 0.669 $mm^{-1}$ | |
| F(000) | 1224 | |
| Crystal size | 0.190 x 0.150 x 0.120 $mm^3$ | |
| Theta range for data collection | 5.212 to 67.492°. | |
| Index ranges | -11<=h<=9, -18<=k<=20, -18<=l<=23 | |
| Reflections collected | 18982 | |
| Independent reflections | 5418 [R(int) = 0.0566] | |
| Completeness to theta = 67.679° | 97.6 % | |
| Absorption correction | Semi-empirical from equivalents | |
| Max. and min. transmission | 0.7533 and 0.4963 | |
| Refinement method | Full-matrix least-squares on $F^2$ | |
| Data / restraints / parameters | 5418 / 0 / 407 | |

| | |
|---|---|
| Goodness-of-fit on $F^2$ | 1.061 |
| Final R indices [I>2sigma(I)] | R1 = 0.0447, wR2 = 0.1121 |
| R indices (all data) | R1 = 0.0502, wR2 = 0.1182 |
| Absolute structure parameter | 0.04(12) |
| Extinction coefficient | 0.0207(18) |
| Largest diff. peak and hole | 0.117 and -0.136 e.Å$^{-3}$ |

## (4)X-ray crystal structure of *dia*-78 (CCDC 2215065)

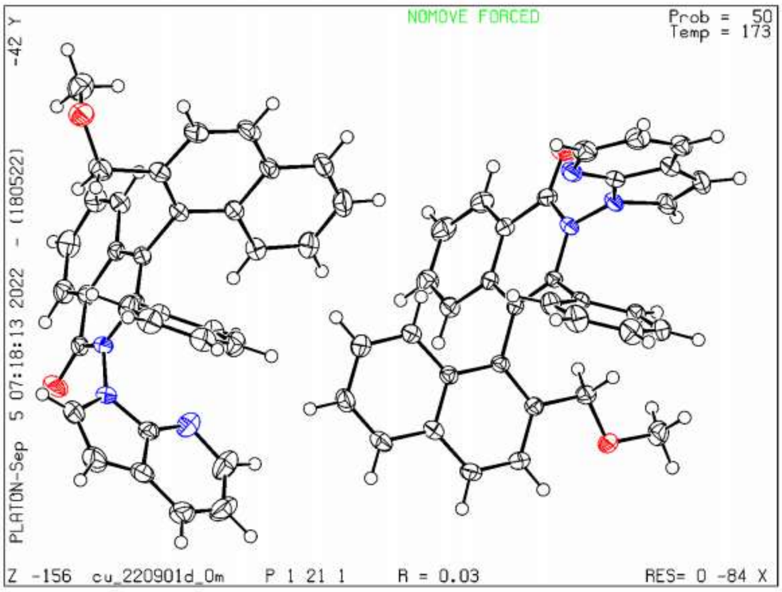


Bond precision: C-C = 0.0032 A Wavelength=1.54178

Cell: a=12.7440(16) b=12.8309(15) c=15.768(2)
alpha=90 beta=91.849(5) gamma=90

Temperature: 173 K

| | Calculated | Reported |
|---|---|---|
| Volume | 2577.0(5) | 2577.0(5) |
| Space group | P 21 | P 1 21 1 |
| Hall group | P 2yb | P 2yb |
| Moiety formula | C34 H25 N3 O2 | C34 H25 N3 O2 |
| Sum formula | C34 H25 N3 O2 | C34 H25 N3 O2 |
| Mr | 507.57 | 507.57 |
| Dx,g cm-3 | 1.308 | 1.308 |
| Z | 4 | 4 |
| Mu (mm-1) | 0.652 | 0.652 |
| F000 | 1064.0 | 1064.0 |
| F000' | 1067.05 | |
| h,k,lmax | 15,15,18 | 15,15,18 |
| Nref | 9095[ 4770] | 8329 |
| Tmin,Tmax | 0.937,0.949 | 0.656,0.753 |
| Tmin' | 0.937 | |

Correction method= # Reported T Limits: Tmin=0.656 Tmax=0.753
AbsCorr = MULTI-SCAN

Data completeness= 1.75/0.92 Theta(max)= 66.593

R(reflections)= 0.0297( 8012) wR2(reflections)= 0.0769( 8329)

S = 1.052 Npar= 705

## 2.8 ECD spectrum and DFT calculations of N-N single-axis product.

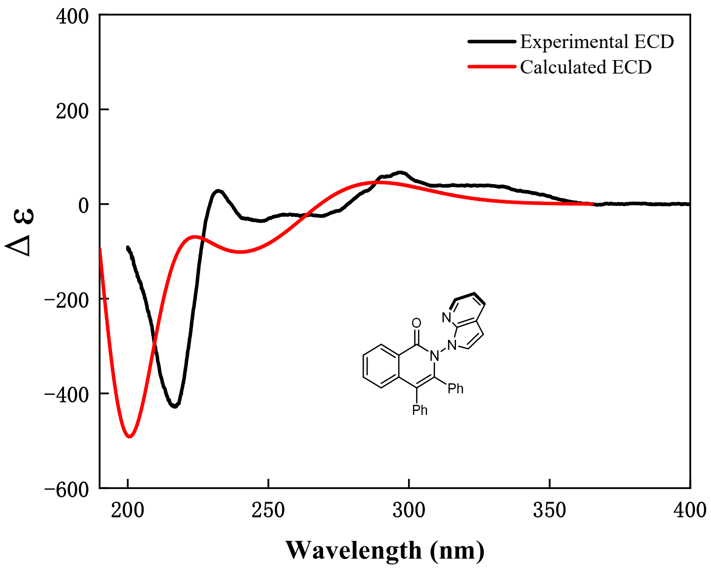


**Supplementary Figure 130.** Comparison of calculated (red line) and experimental (black line) ECD spectra of N-N single-axis product (*S*).

## 2.9 NMR Spectra

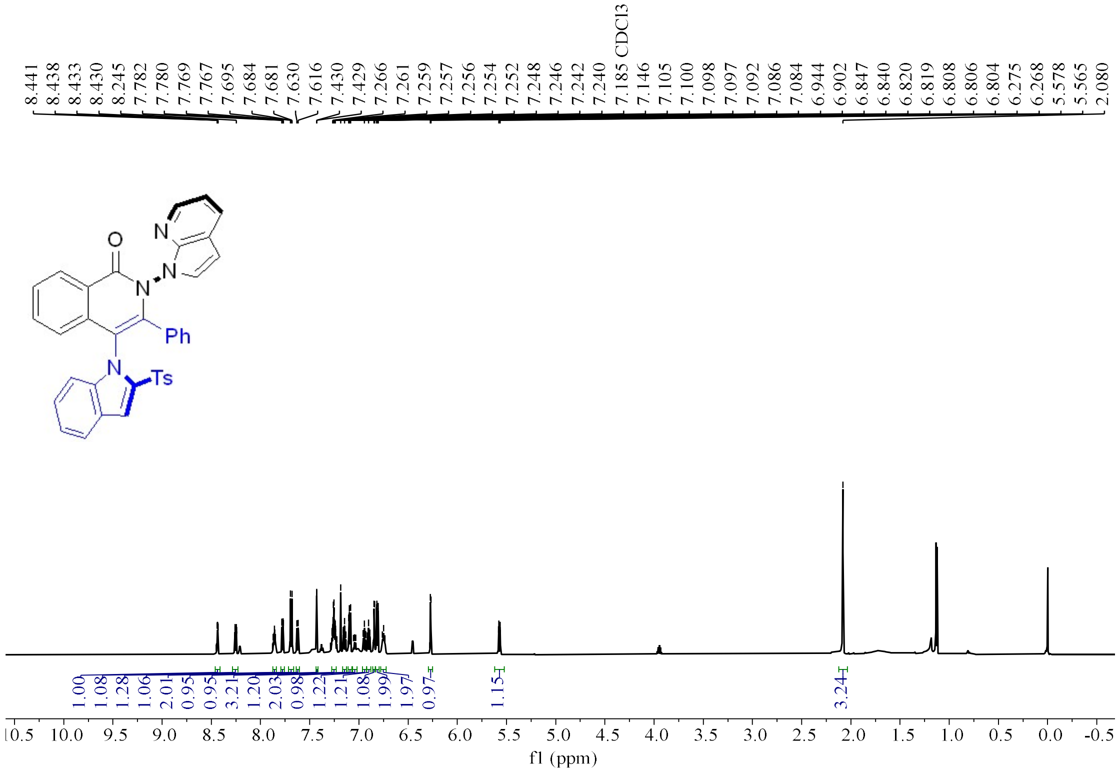


**Supplementary Figure 131. $^{1}H$ NMR (600 MHz, $CDCl_3$) spectrum of 3.**

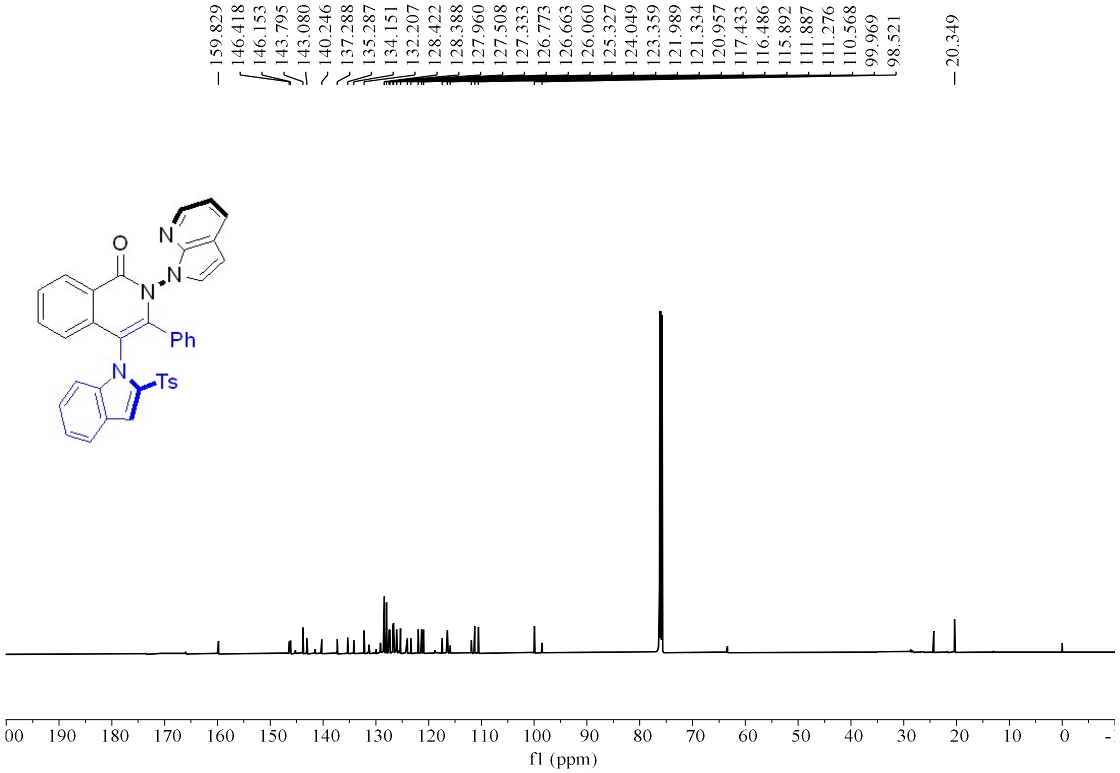


**Supplementary Figure 132. $^{13}C$ NMR (150 MHz, $CDCl_3$) spectrum of 3.**

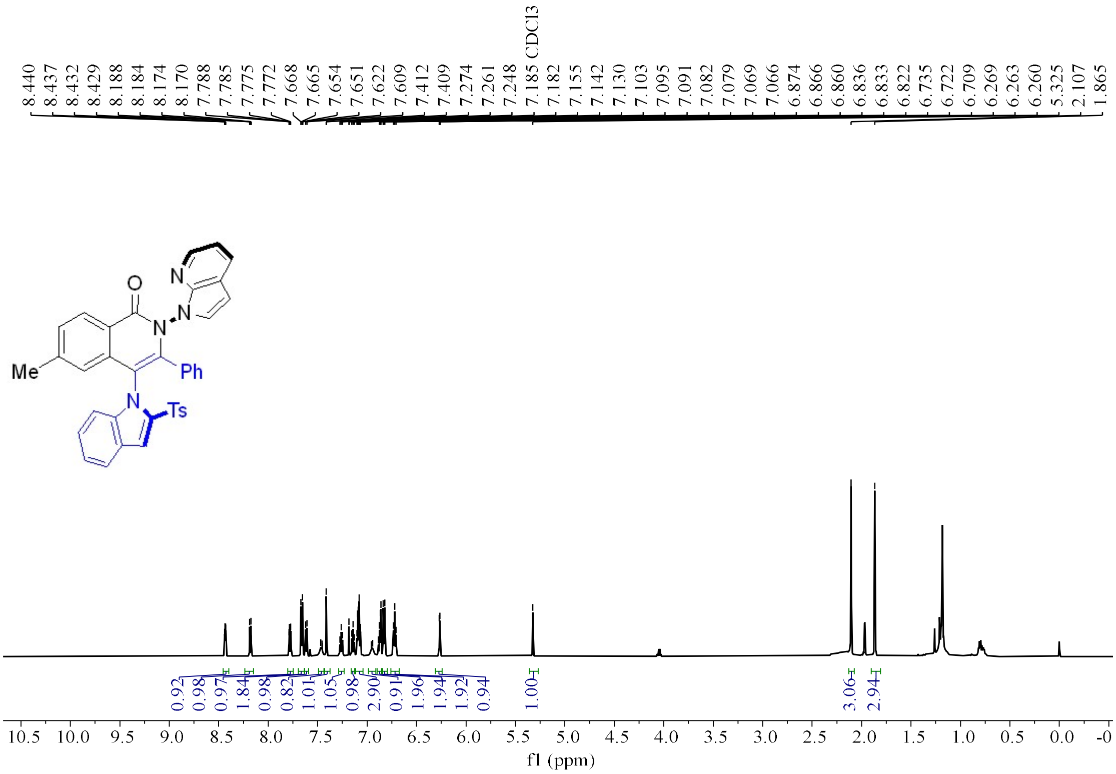


**Supplementary Figure 133. $^{1}$H NMR (600 MHz, $CDCl_3$) spectrum of 4.**

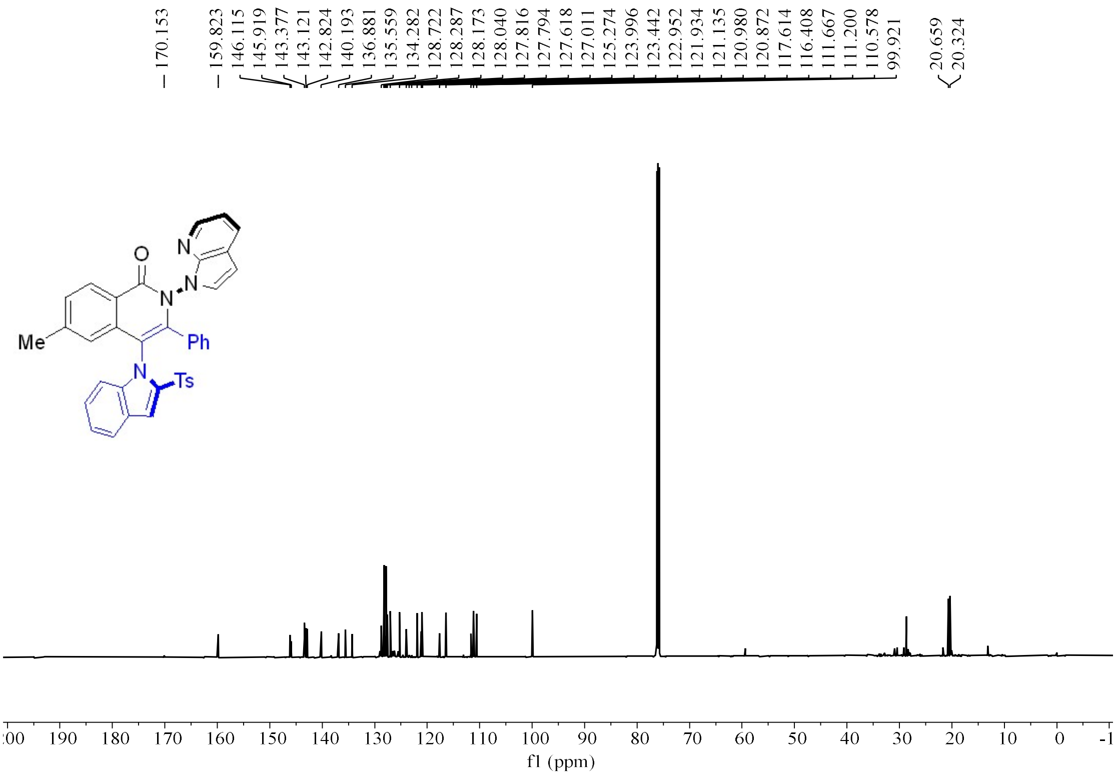


**Supplementary Figure 134. $^{13}$C NMR (150 MHz, $CDCl_3$) spectrum of 4.**

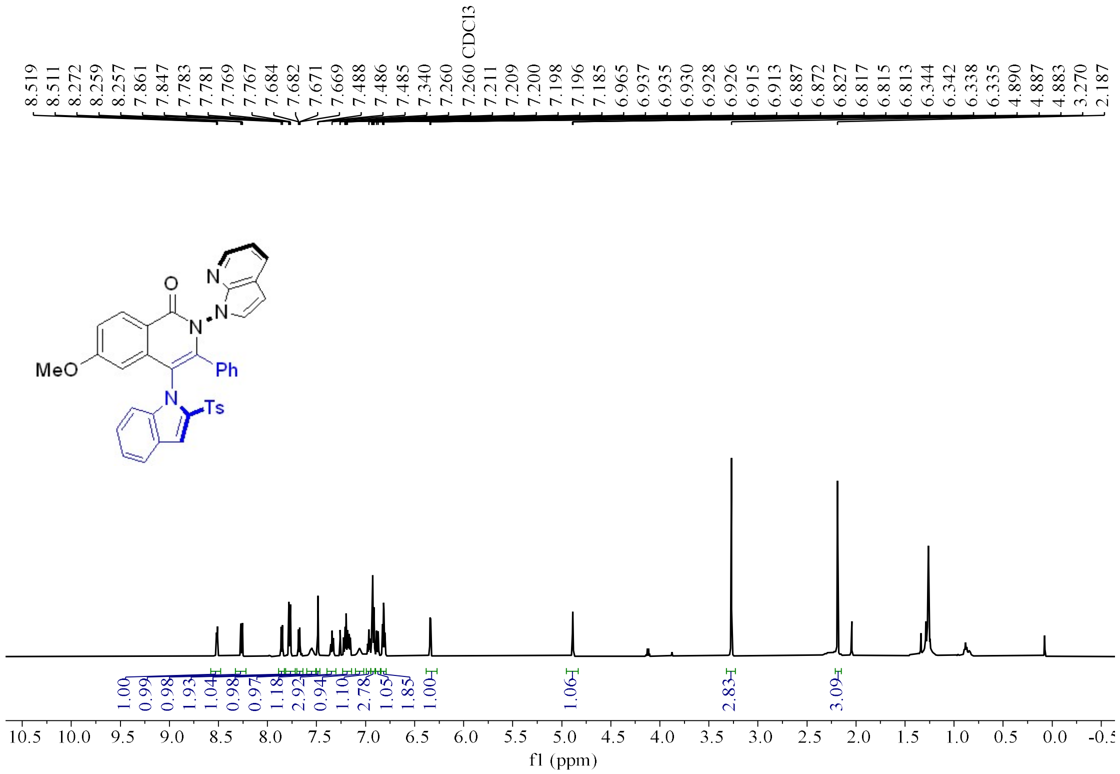


**Supplementary Figure 135. $^{1}$H NMR (600 MHz, $CDCl_3$) spectrum of 5.**

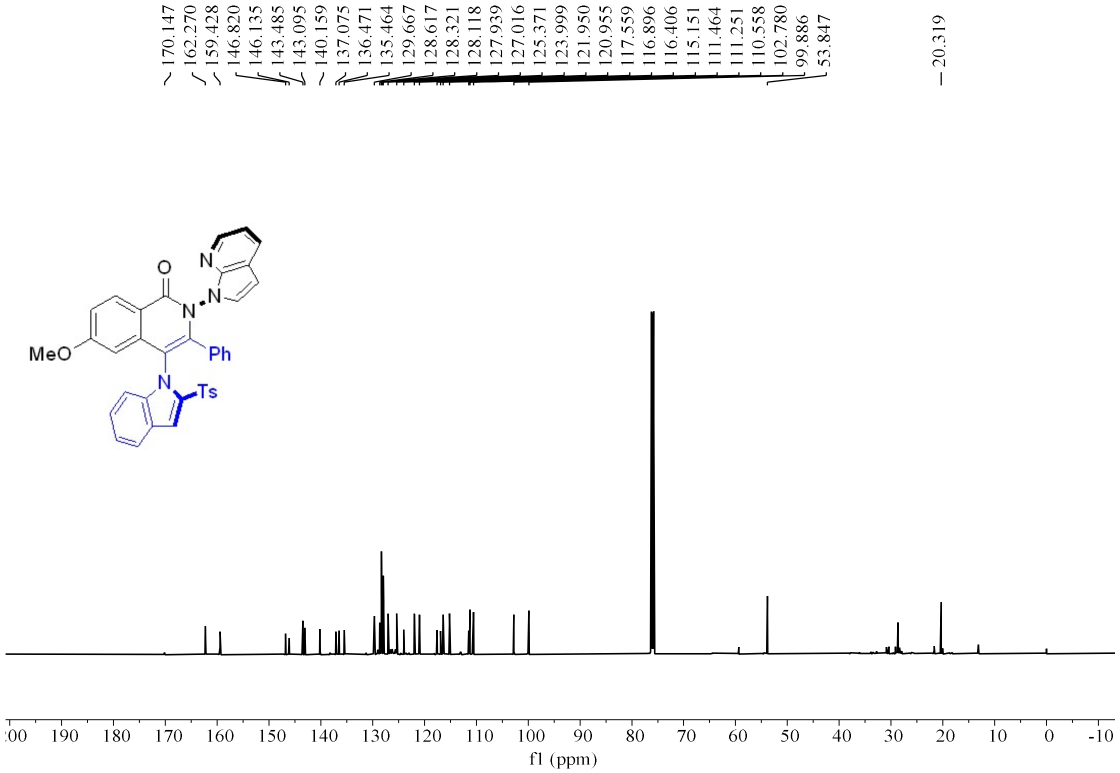


**Supplementary Figure 136. $^{13}$C NMR (150 MHz, $CDCl_3$) spectrum of 5.**

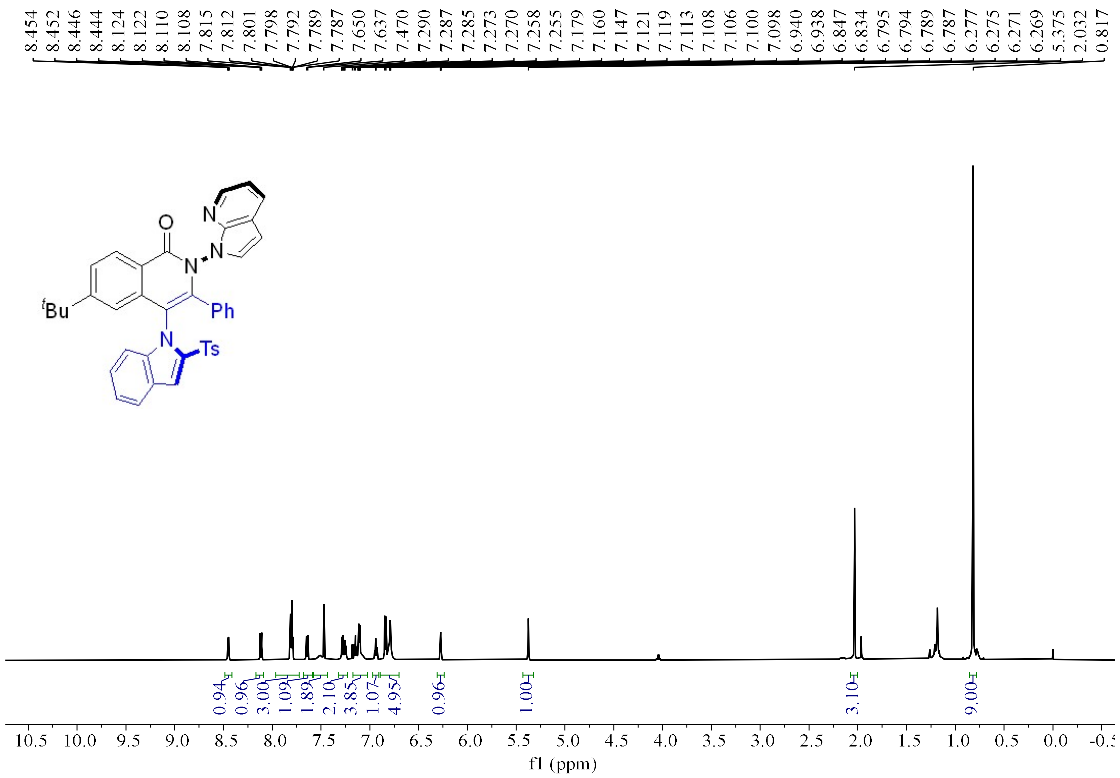

**Supplementary Figure 137. $^{1}$H NMR (600 MHz, $CDCl_3$) spectrum of 6.**

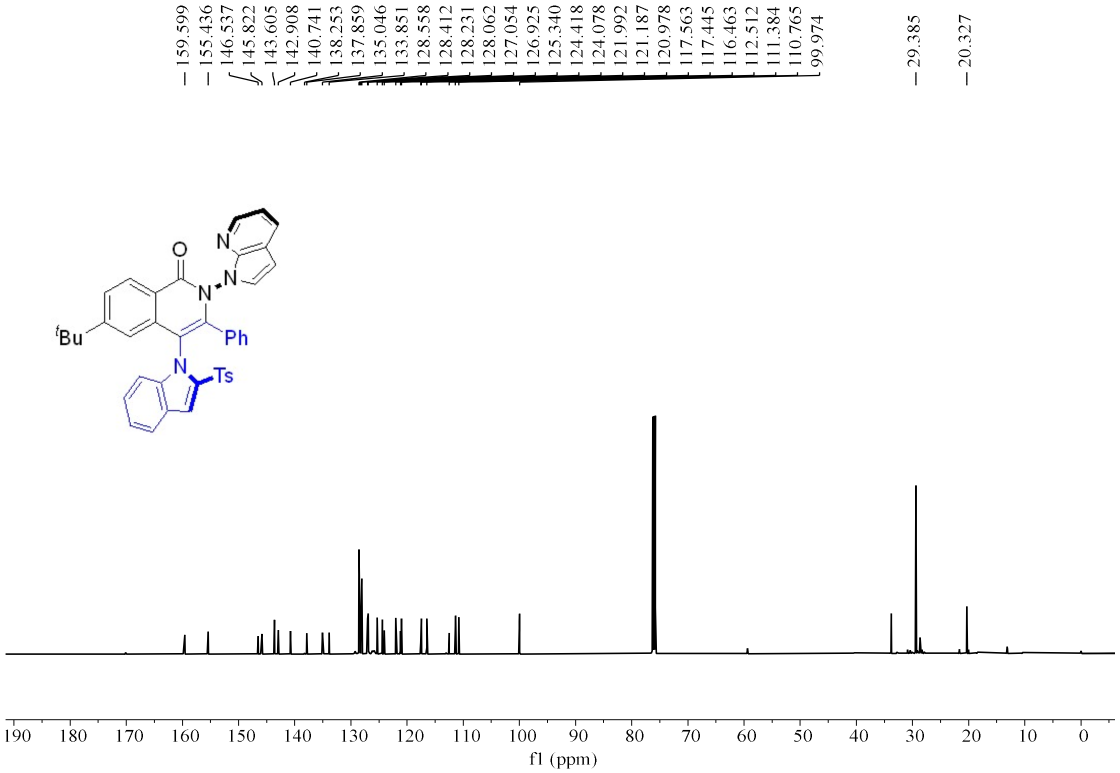

**Supplementary Figure 138. $^{13}$C NMR (150 MHz, $CDCl_3$) spectrum of 6.**

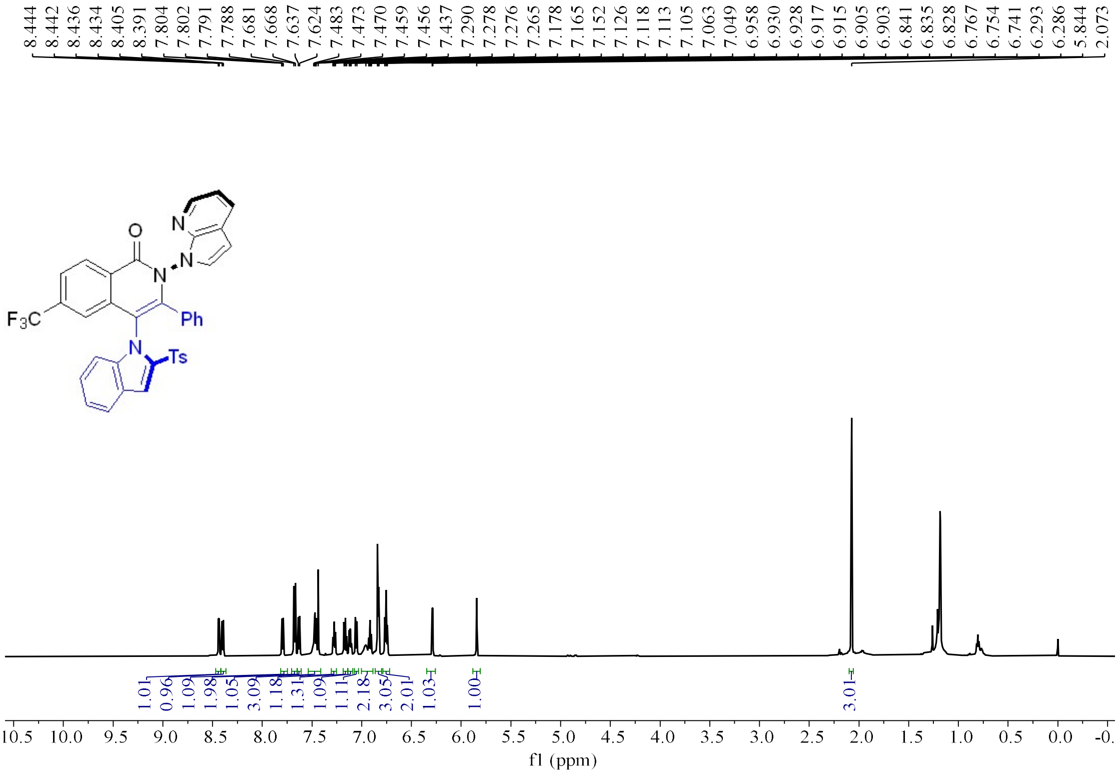


**Supplementary Figure 139.** $^{1}$**H NMR (600 MHz, CDCl$_3$) spectrum of 7.**

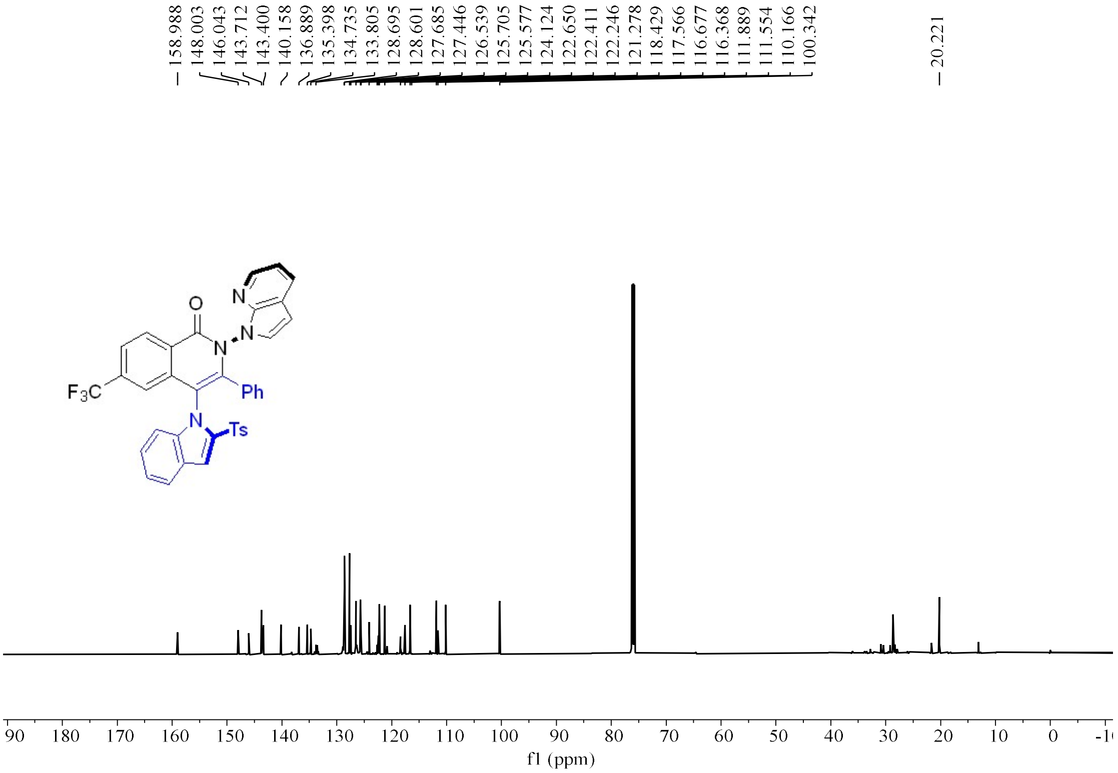


**Supplementary Figure 140.** $^{13}$**C NMR (150 MHz, CDCl$_3$) spectrum of 7.**

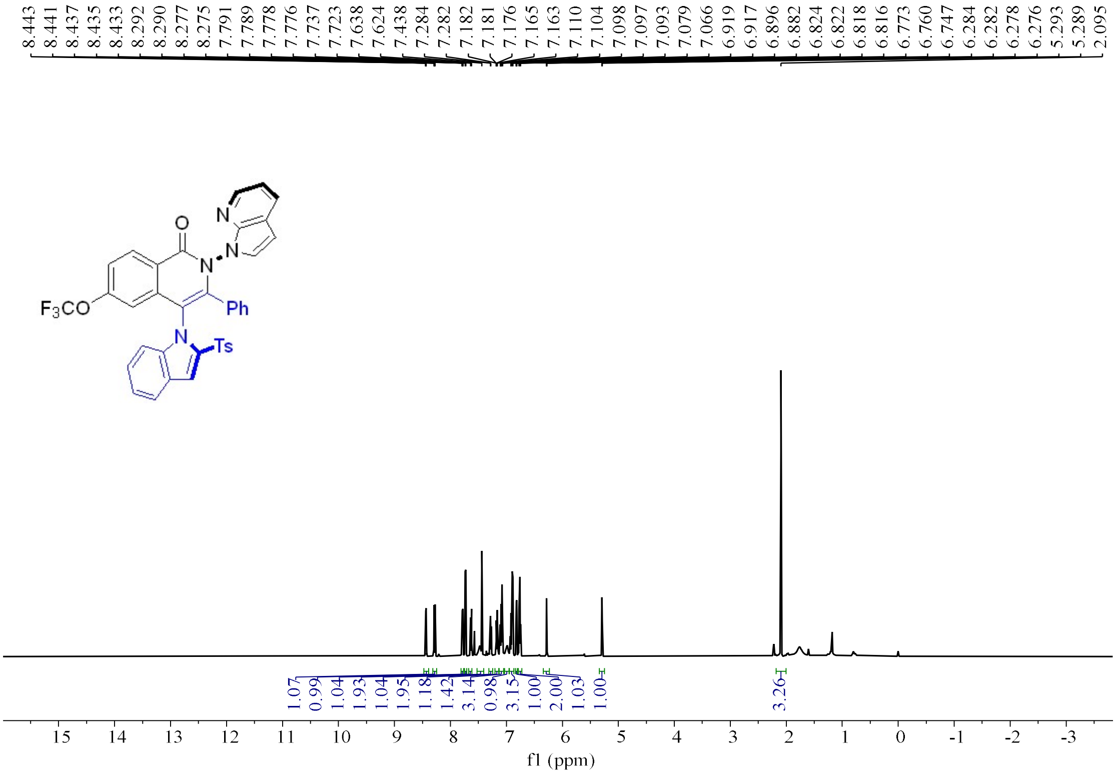


**Supplementary Figure 141.** **$^{1}$H NMR (600 MHz, $CDCl_3$) spectrum of 8.**

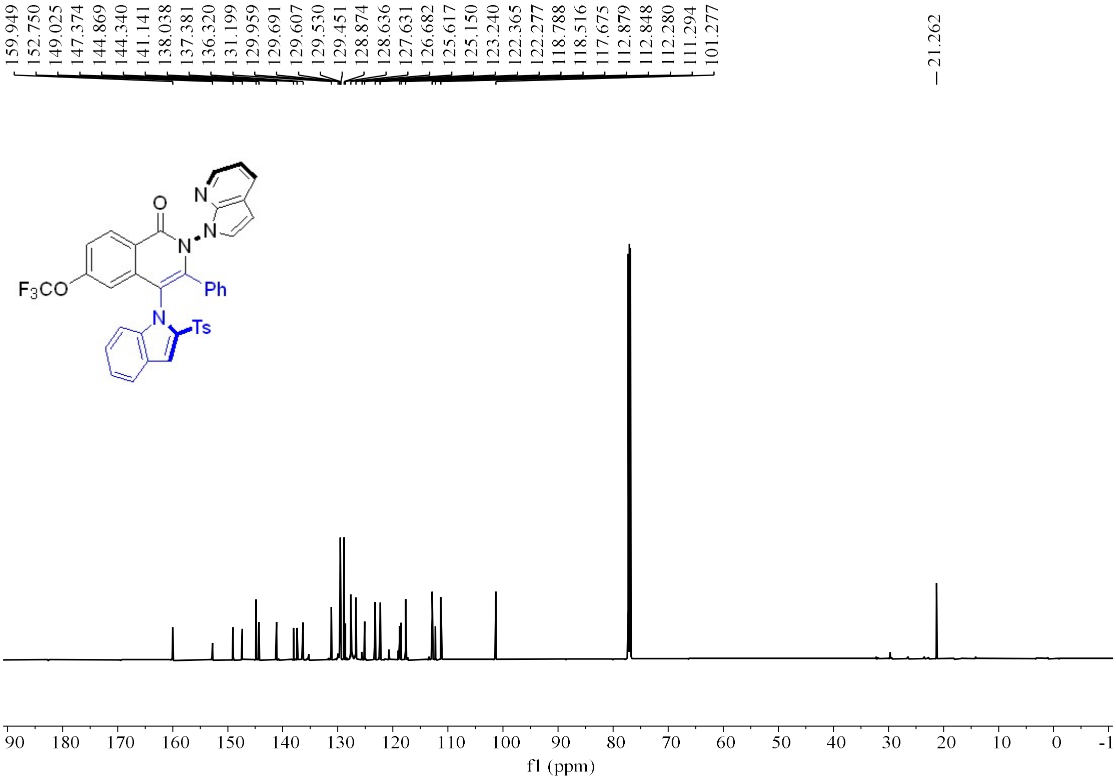


**Supplementary Figure 142.** **$^{13}$C NMR (150 MHz, $CDCl_3$) spectrum of 8.**

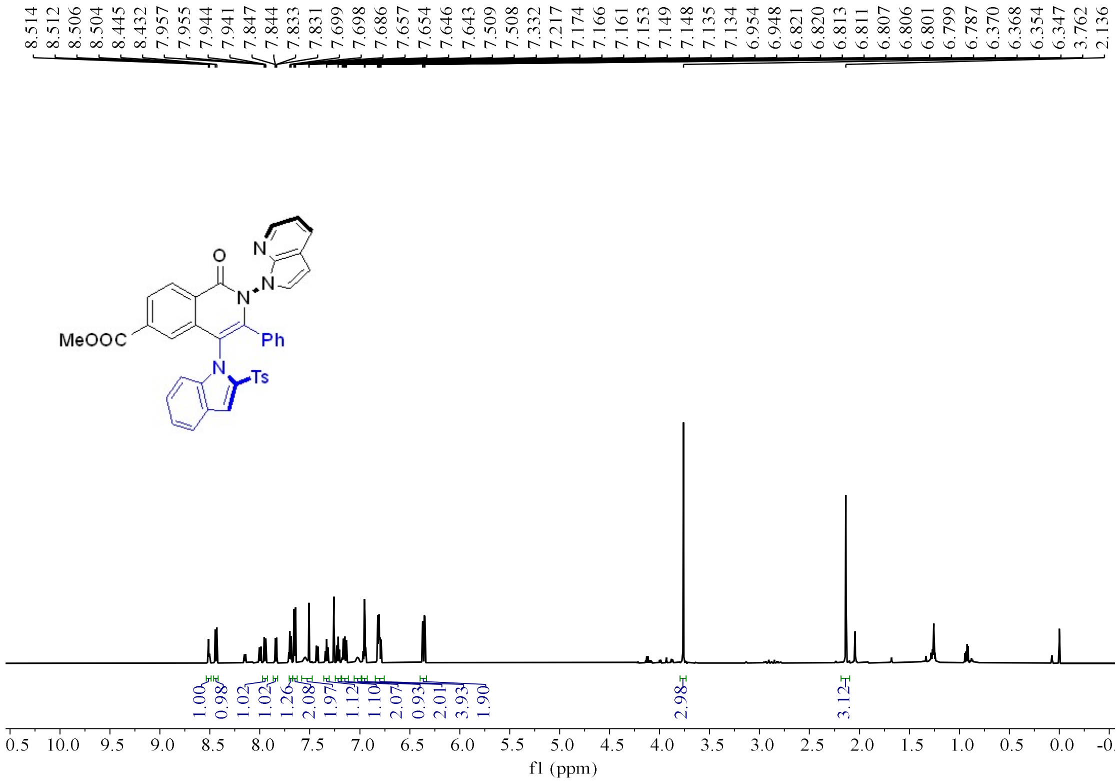


**Supplementary Figure 143.** **$^{1}$H NMR (600 MHz, $CDCl_3$) spectrum of 9.**

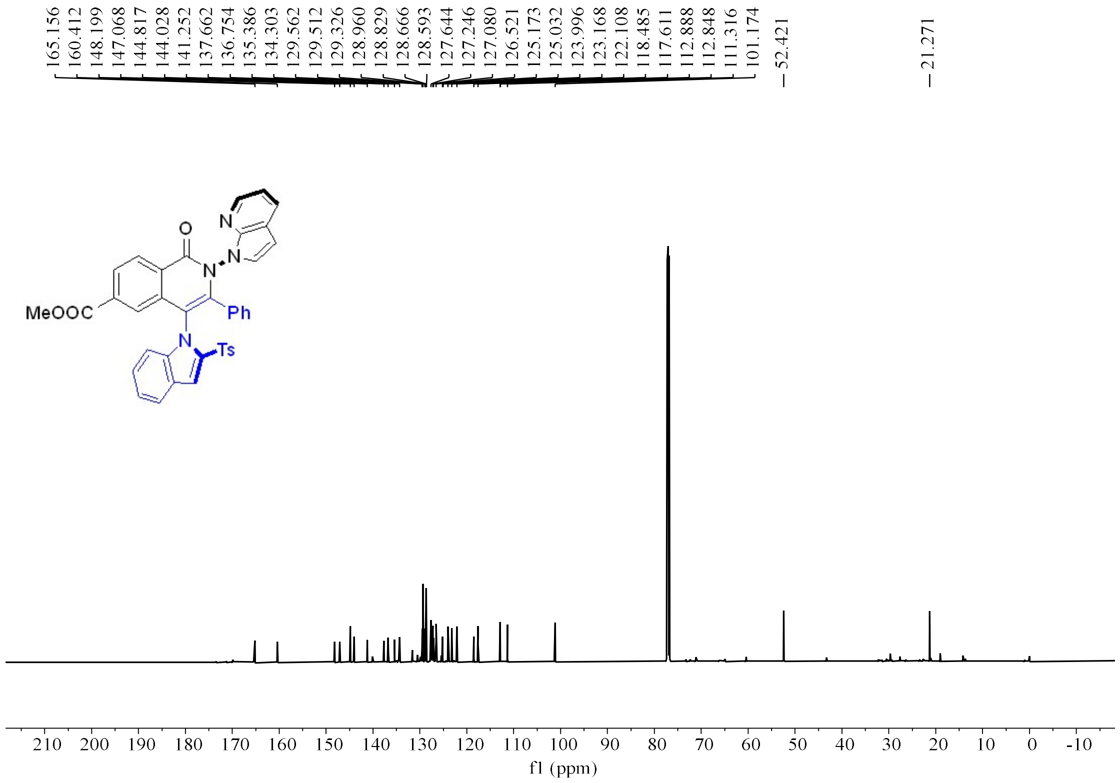


**Supplementary Figure 144.** **$^{13}$C NMR (150 MHz, $CDCl_3$) spectrum of 9.**

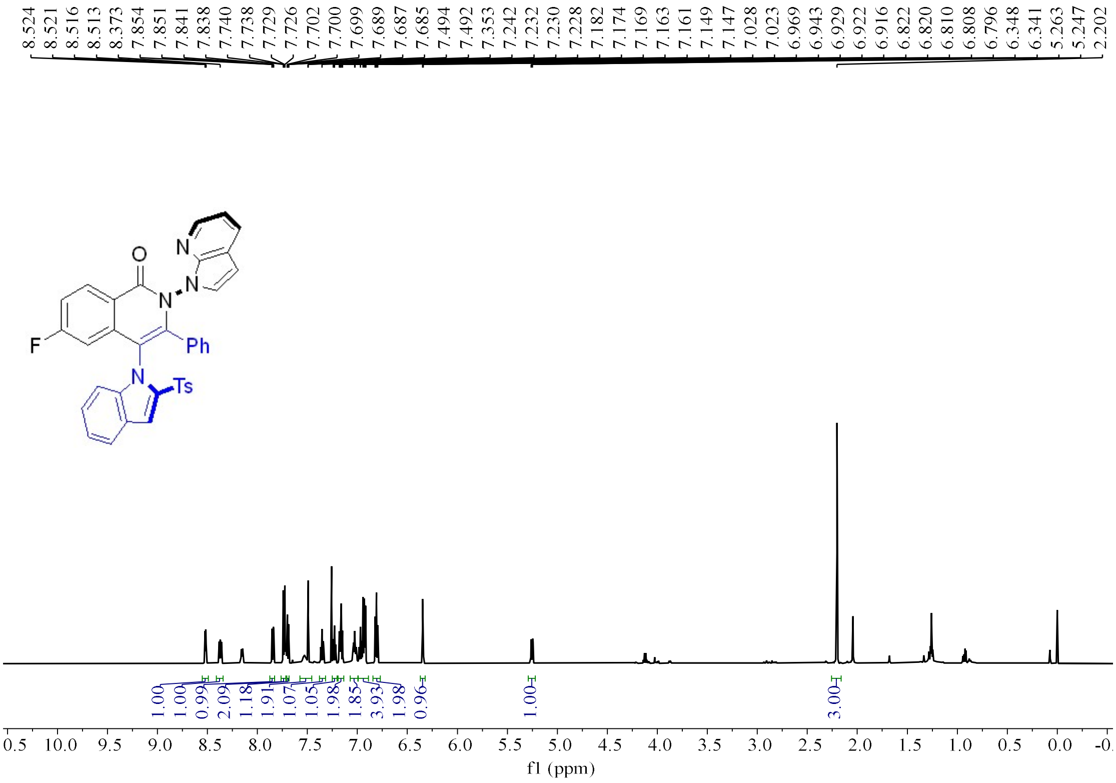


**Supplementary Figure 145.** $^{1}$**H NMR (600 MHz, CDCl$_3$) spectrum of 10.**

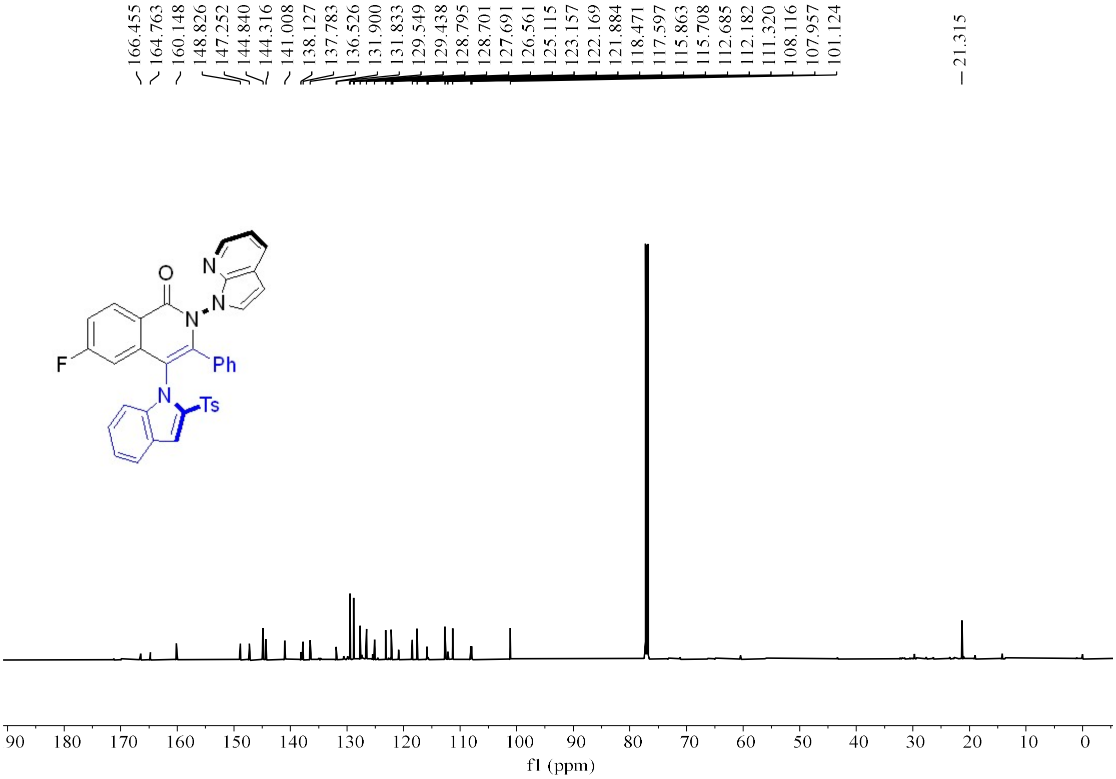


**Supplementary Figure 146.** $^{13}$**C NMR (150 MHz, CDCl$_3$) spectrum of 10.**

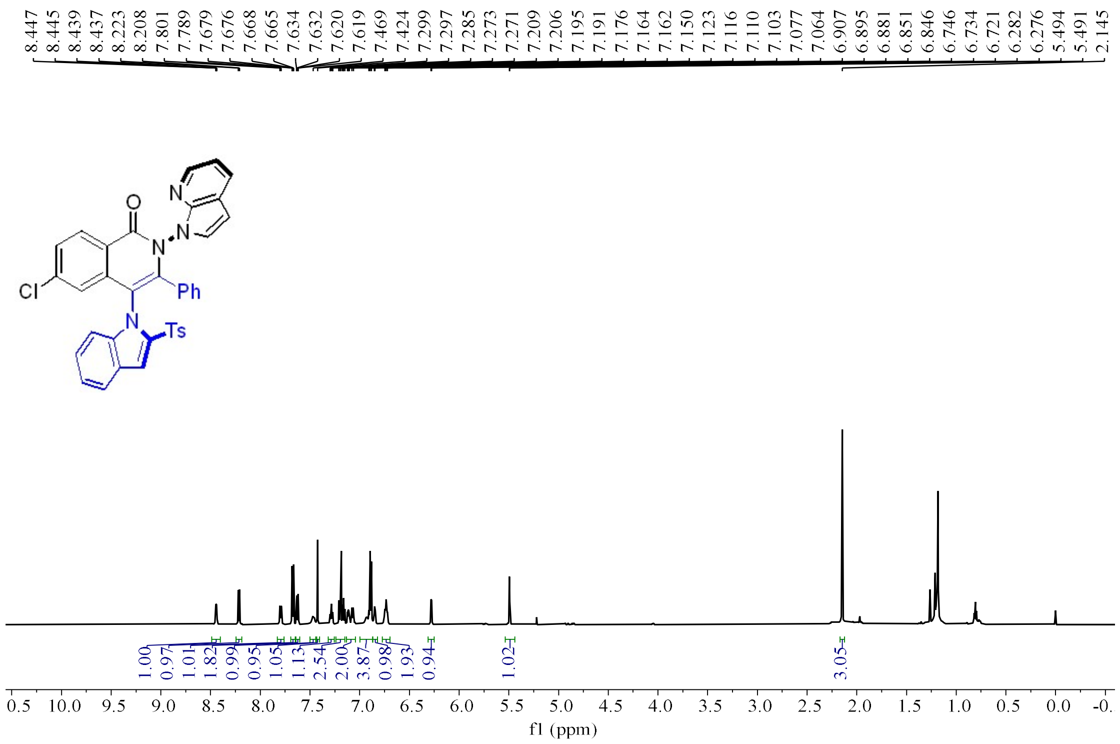


**Supplementary Figure 147. $^{1}H$ NMR (600 MHz, $CDCl_3$) spectrum of 11.**

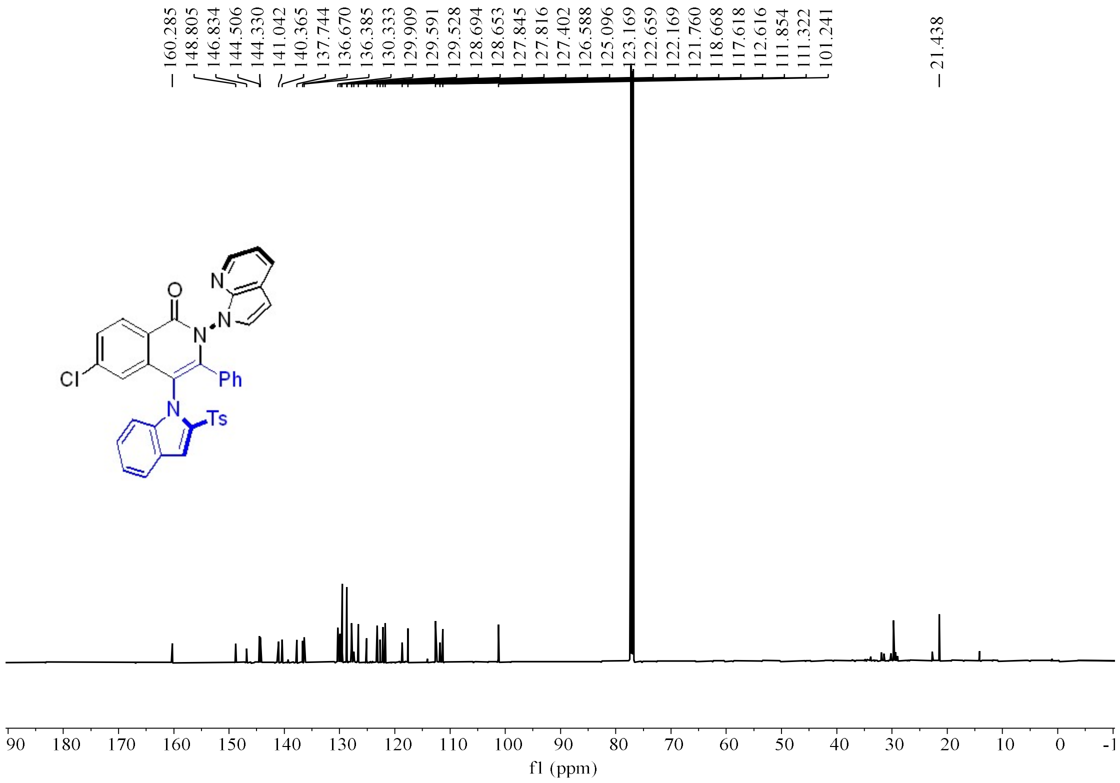


**Supplementary Figure 148. $^{13}C$ NMR (150 MHz, $CDCl_3$) spectrum of 11.**

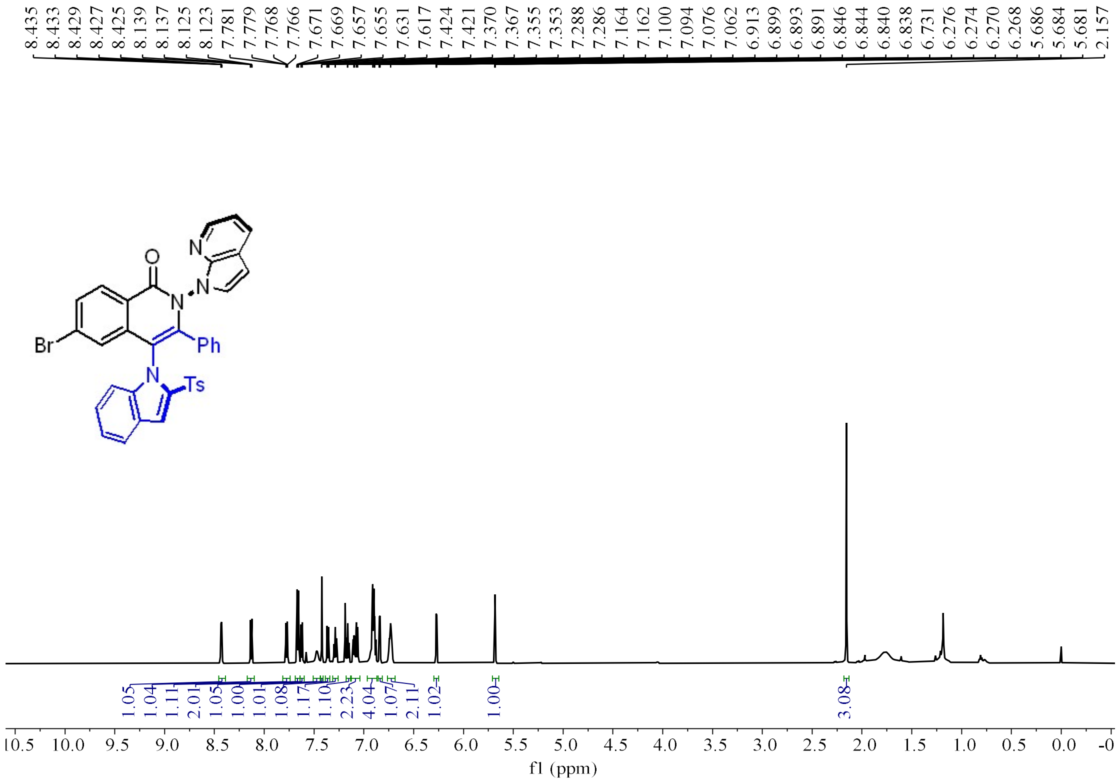


**Supplementary Figure 149. $^{1}$H NMR (600 MHz, $CDCl_3$) spectrum of 12.**

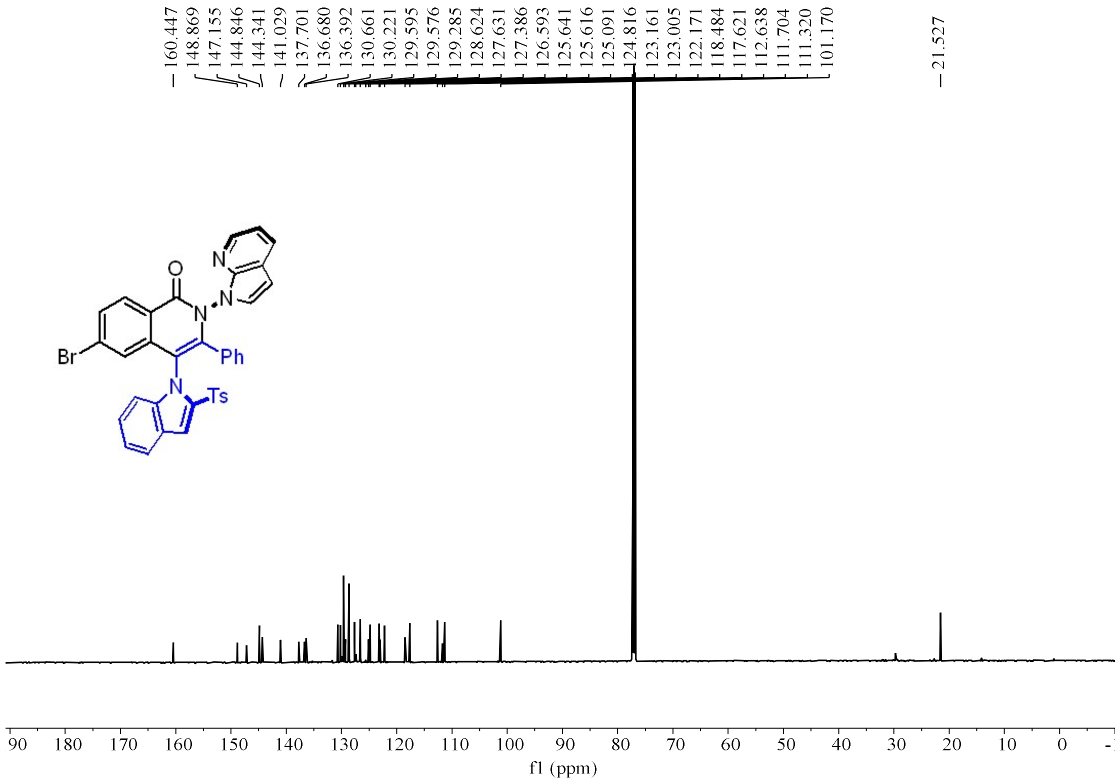


**Supplementary Figure 150. $^{13}$C NMR (150 MHz, $CDCl_3$) spectrum of 12.**

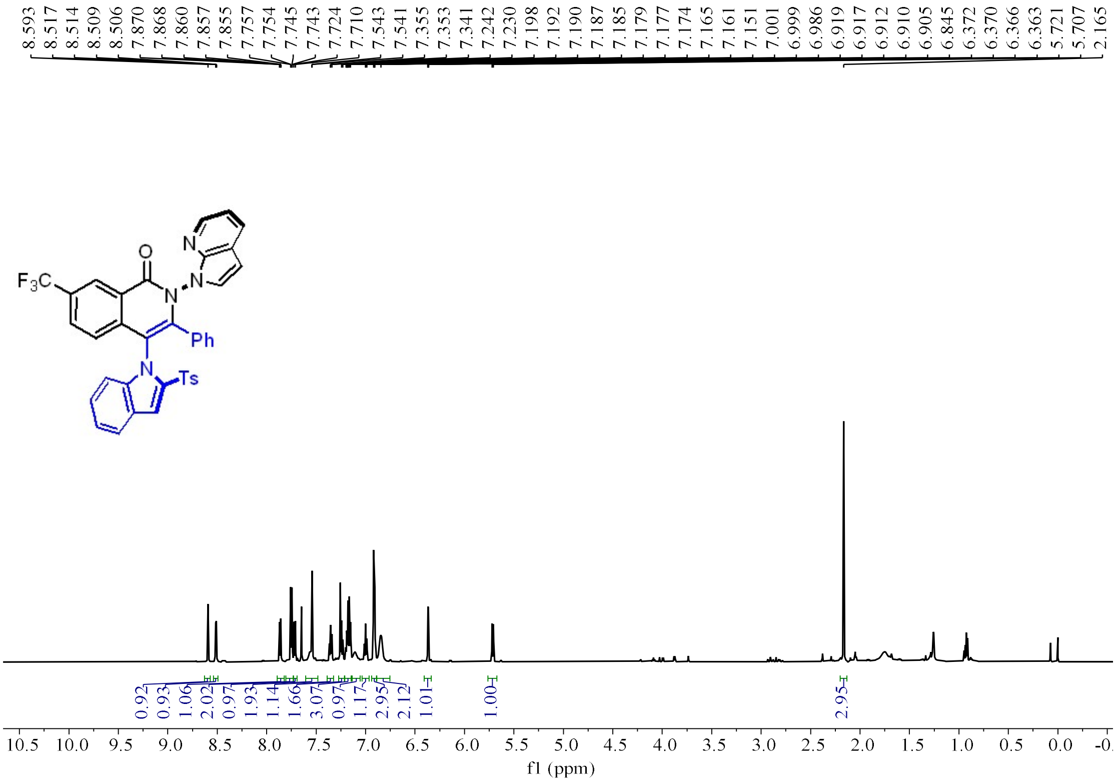


**Supplementary Figure 151. $^{1}$H NMR (600 MHz, CDCl$_3$) spectrum of 13.**

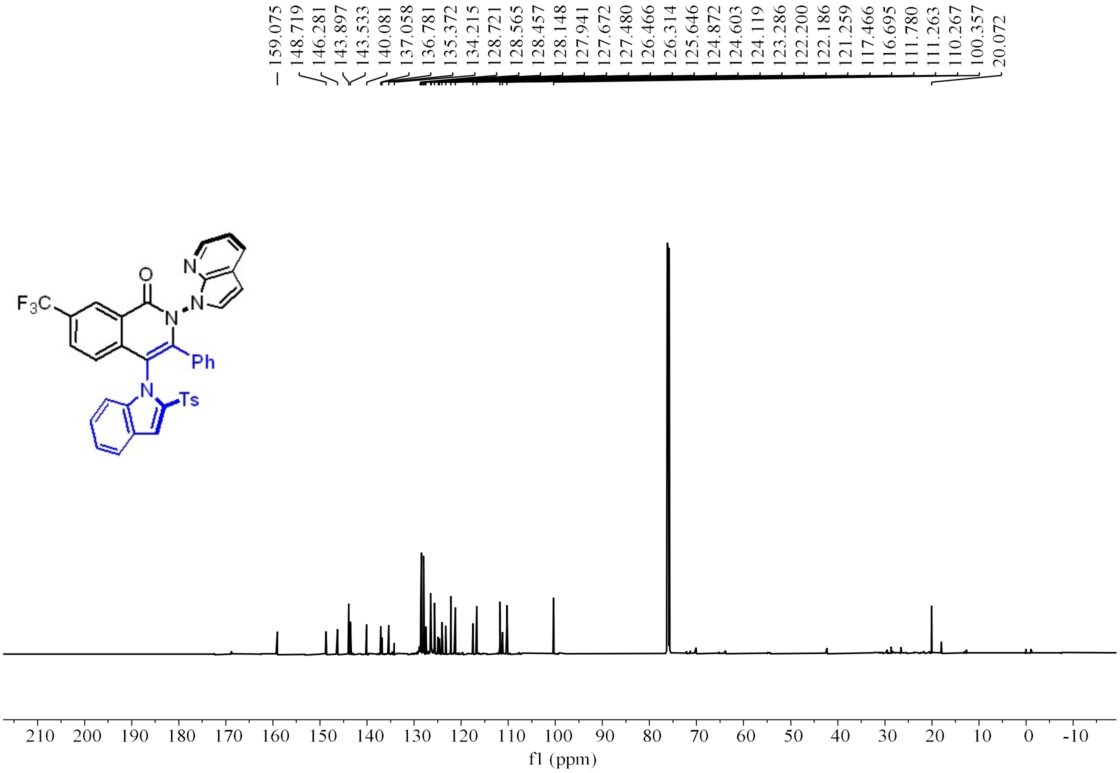


**Supplementary Figure 152. $^{13}$C NMR (150 MHz, CDCl$_3$) spectrum of 13.**

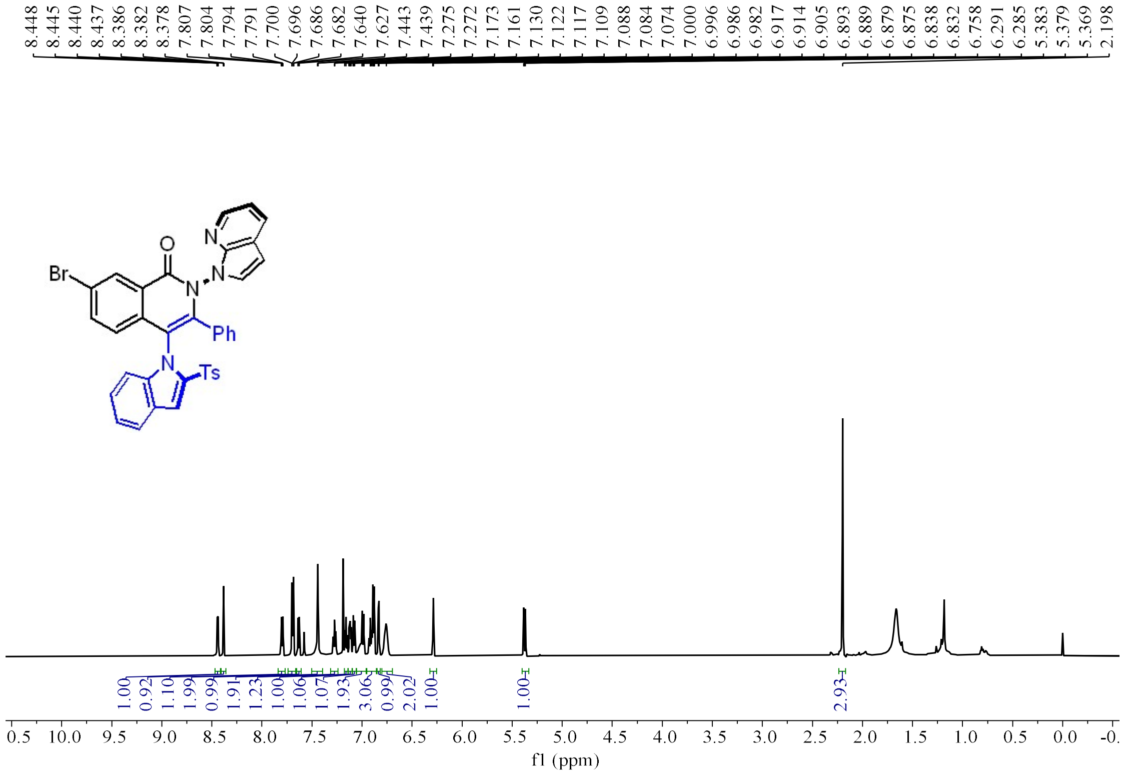

**Supplementary Figure 153. $^{1}$H NMR (600 MHz, $CDCl_3$) spectrum of 14.**

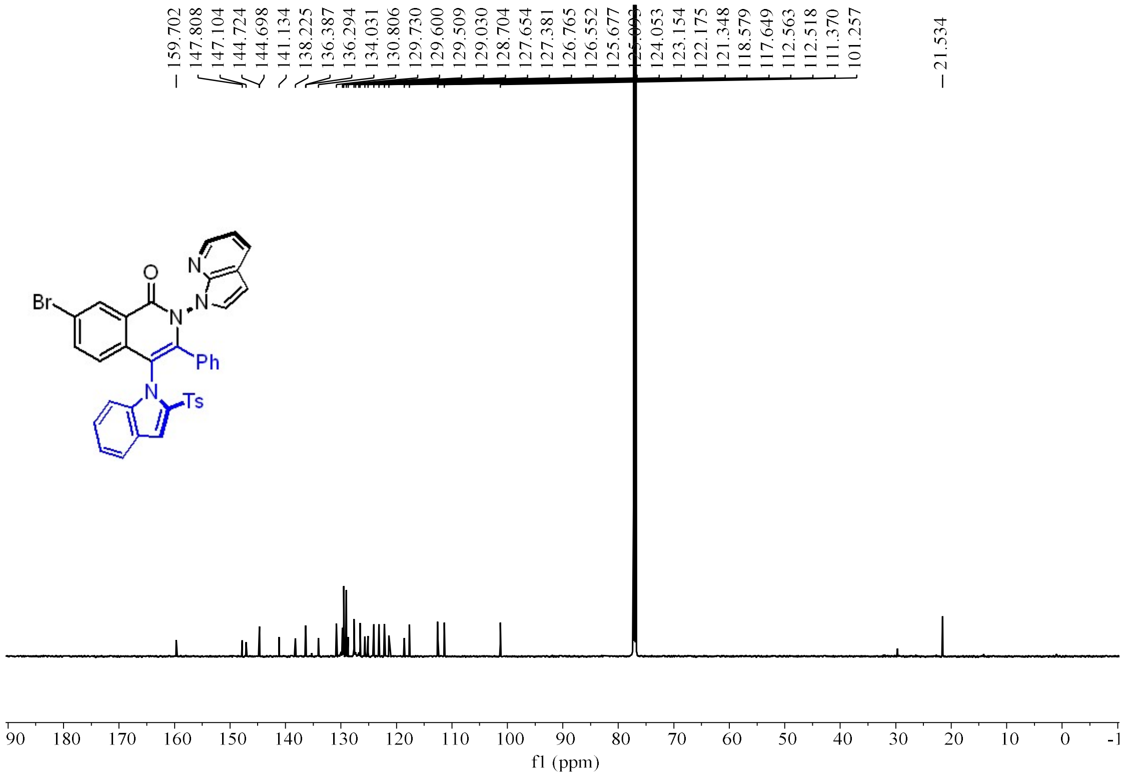

**Supplementary Figure 154. $^{13}$C NMR (150 MHz, $CDCl_3$) spectrum of 14.**

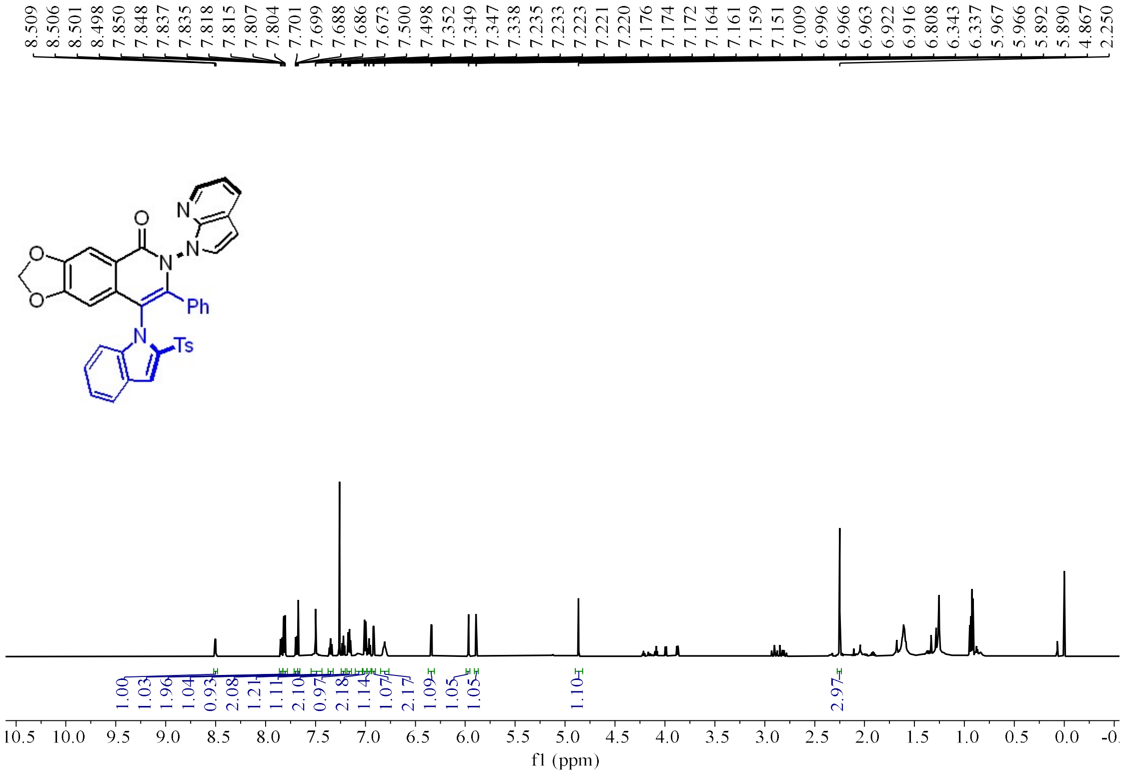


**Supplementary Figure 155. $^1$H NMR (600 MHz, $CDCl_3$) spectrum of 15.**

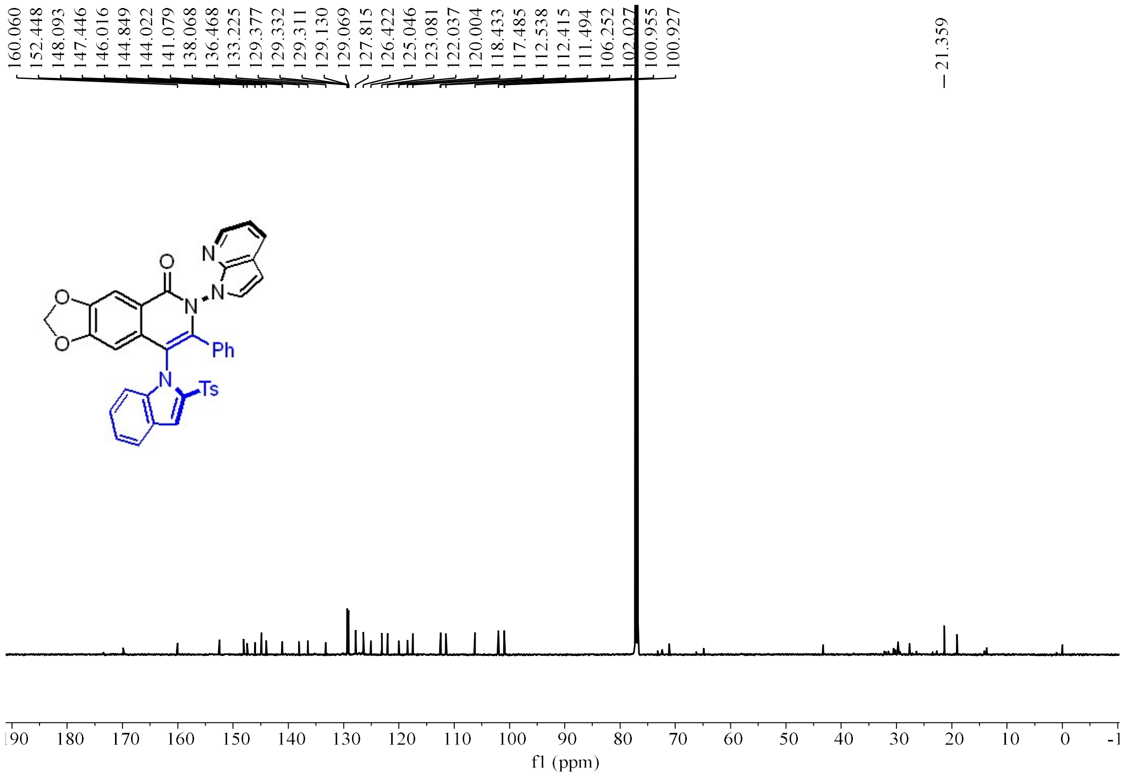


**Supplementary Figure 156. $^{13}C$ NMR (150 MHz, $CDCl_3$) spectrum of 15.**

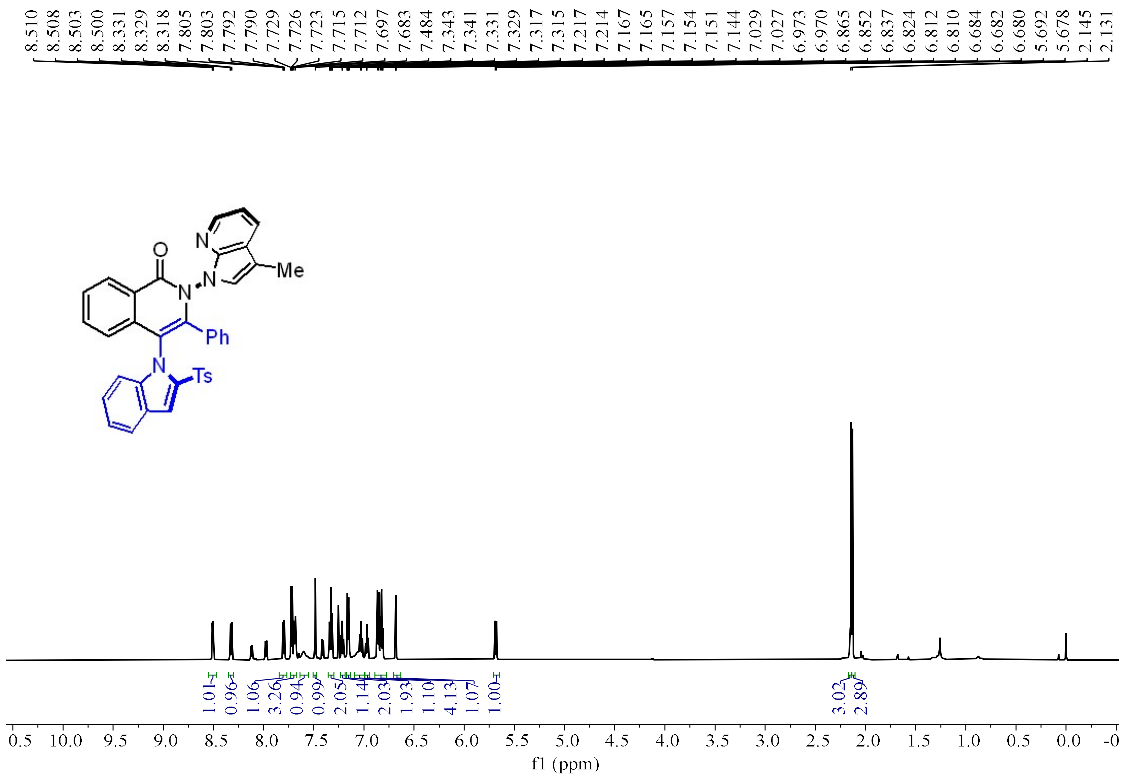

**Supplementary Figure 157. $^{1}$H NMR (600 MHz, $CDCl_3$) spectrum of 16.**

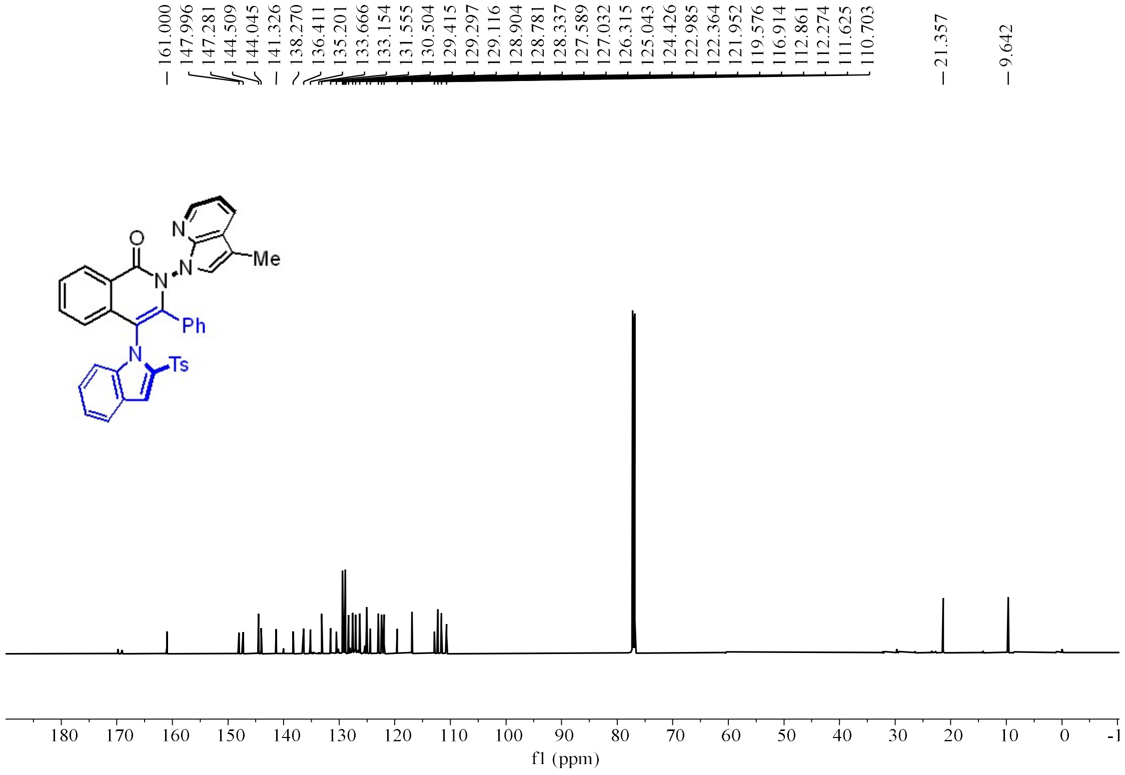

**Supplementary Figure 158. $^{13}$C NMR (150 MHz, $CDCl_3$) spectrum of 16.**

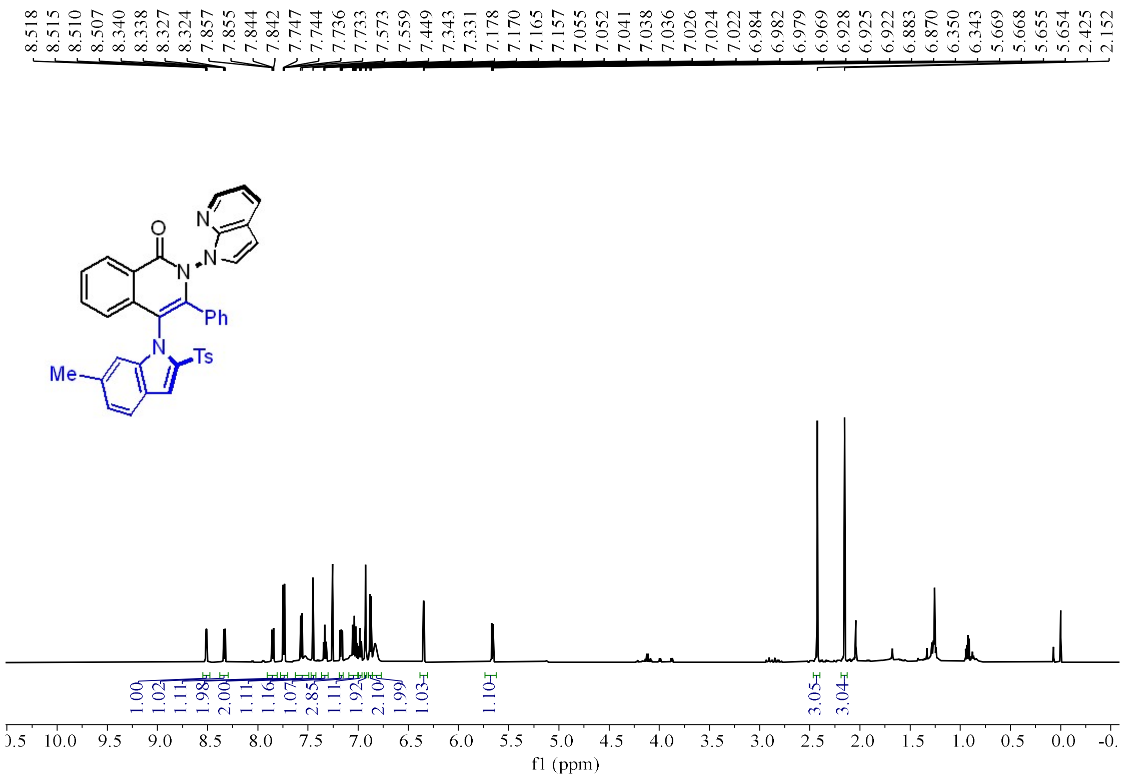


**Supplementary Figure 159. $^{1}$H NMR (600 MHz, $CDCl_3$) spectrum of 17.**

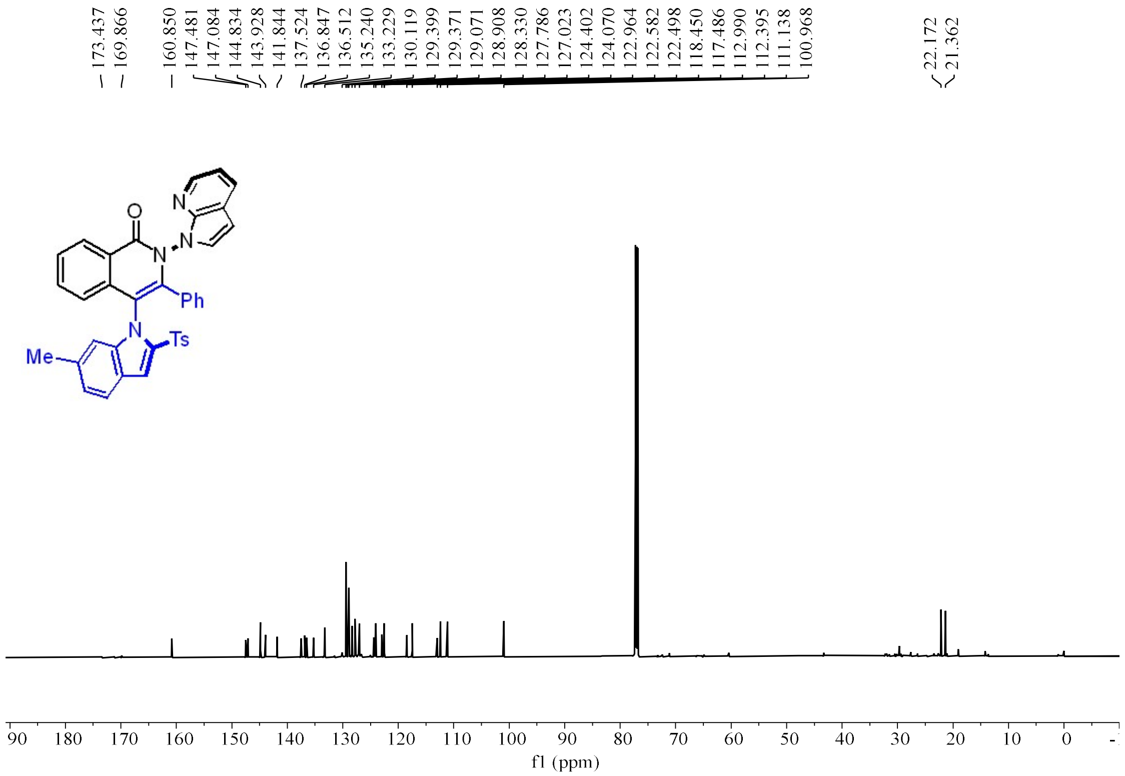


**Supplementary Figure 160. $^{13}$C NMR (150 MHz, $CDCl_3$) spectrum of 17.**

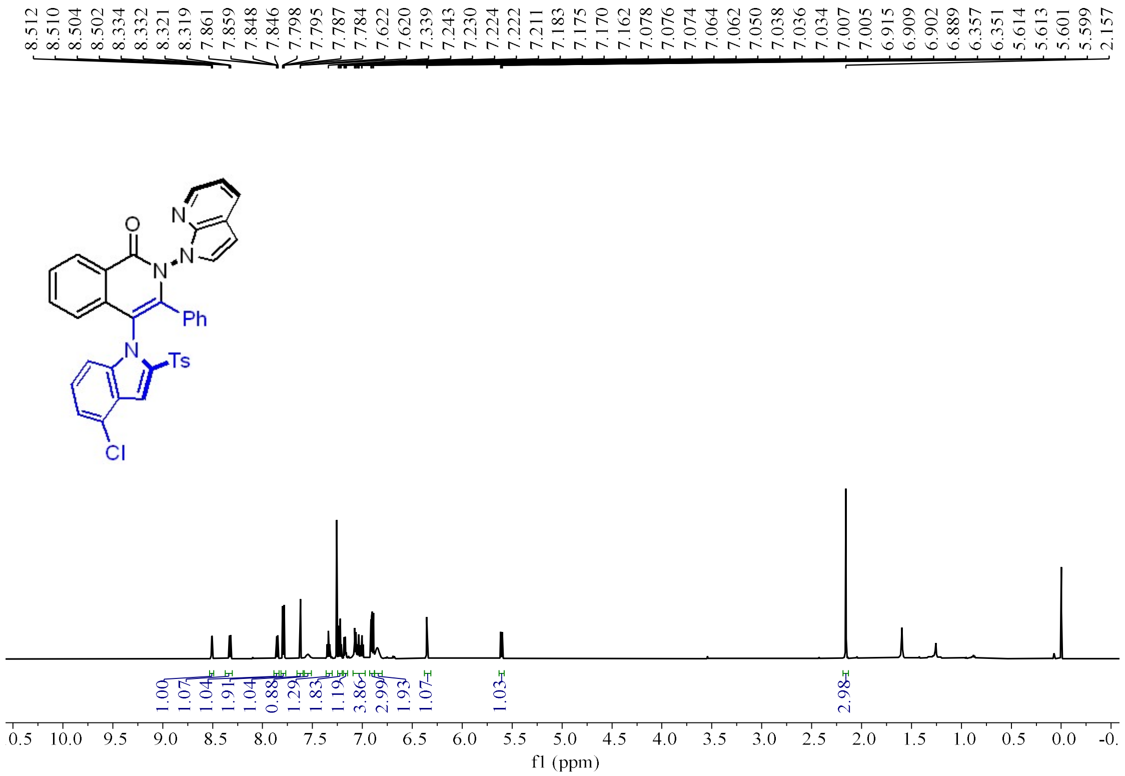


**Supplementary Figure 161. $^{1}$H NMR (600 MHz, $CDCl_3$) spectrum of 18.**

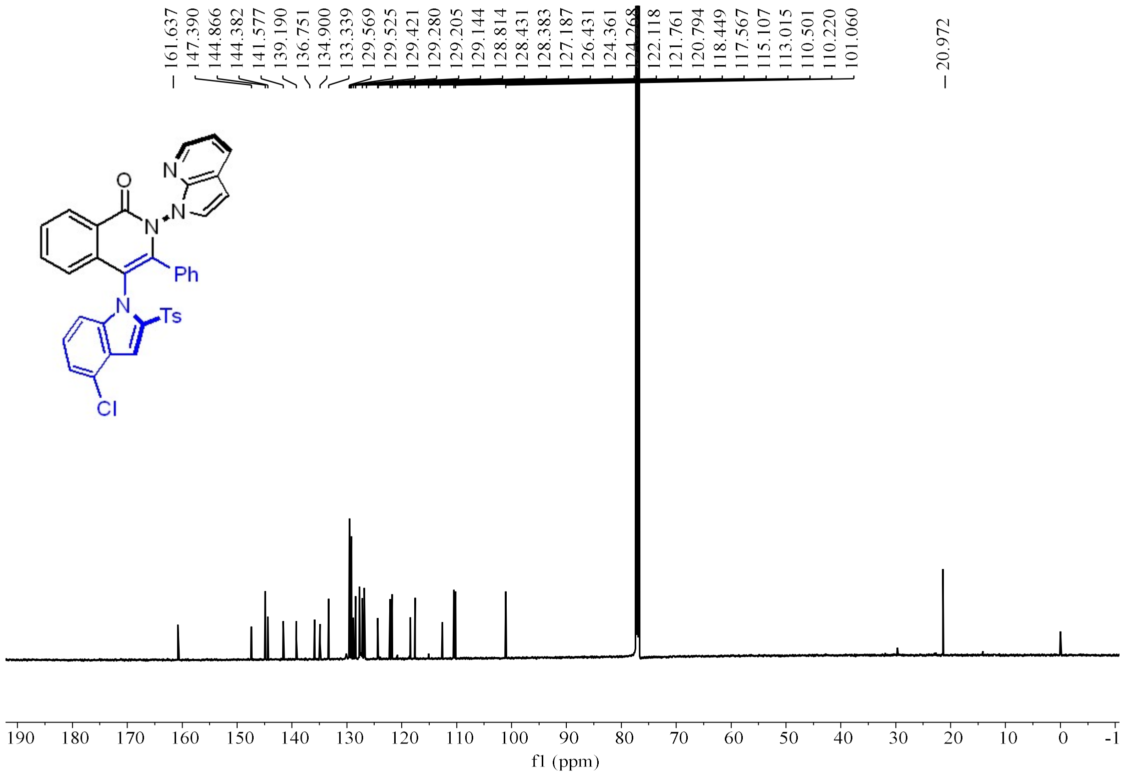


**Supplementary Figure 162. $^{13}$C NMR (150 MHz, $CDCl_3$) spectrum of 18.**

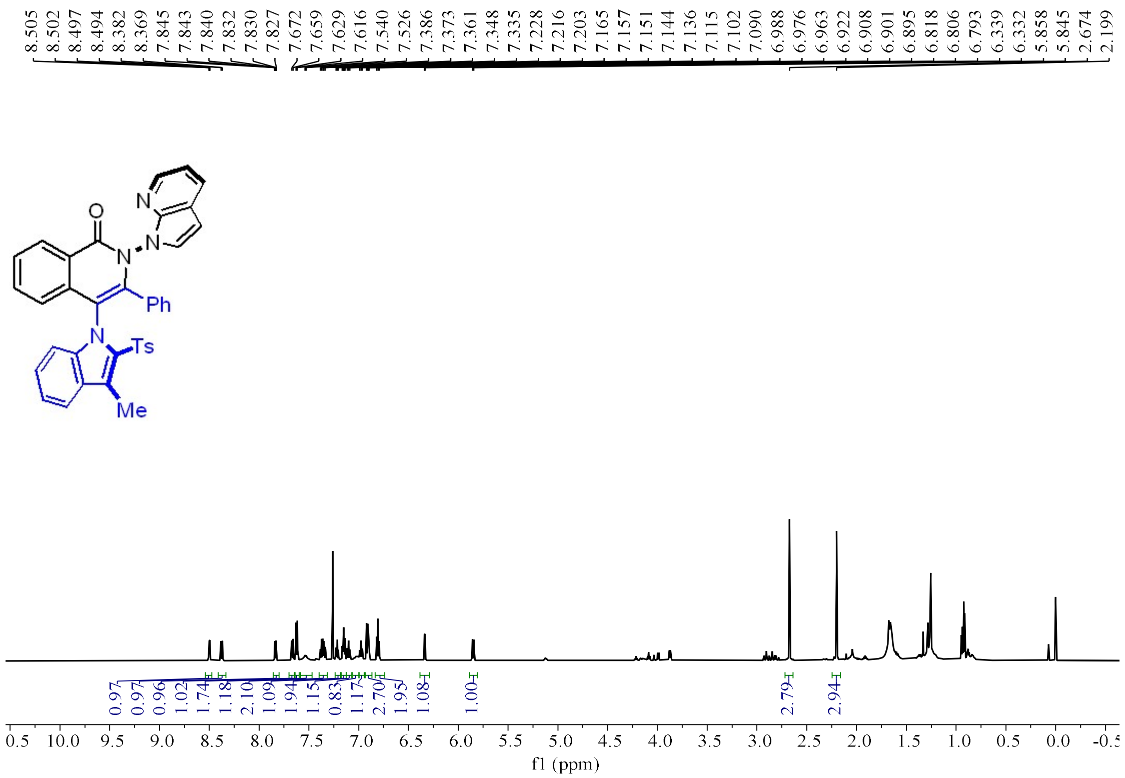


**Supplementary Figure 163.** **$^{1}$H NMR (600 MHz, $CDCl_3$) spectrum of 19.**

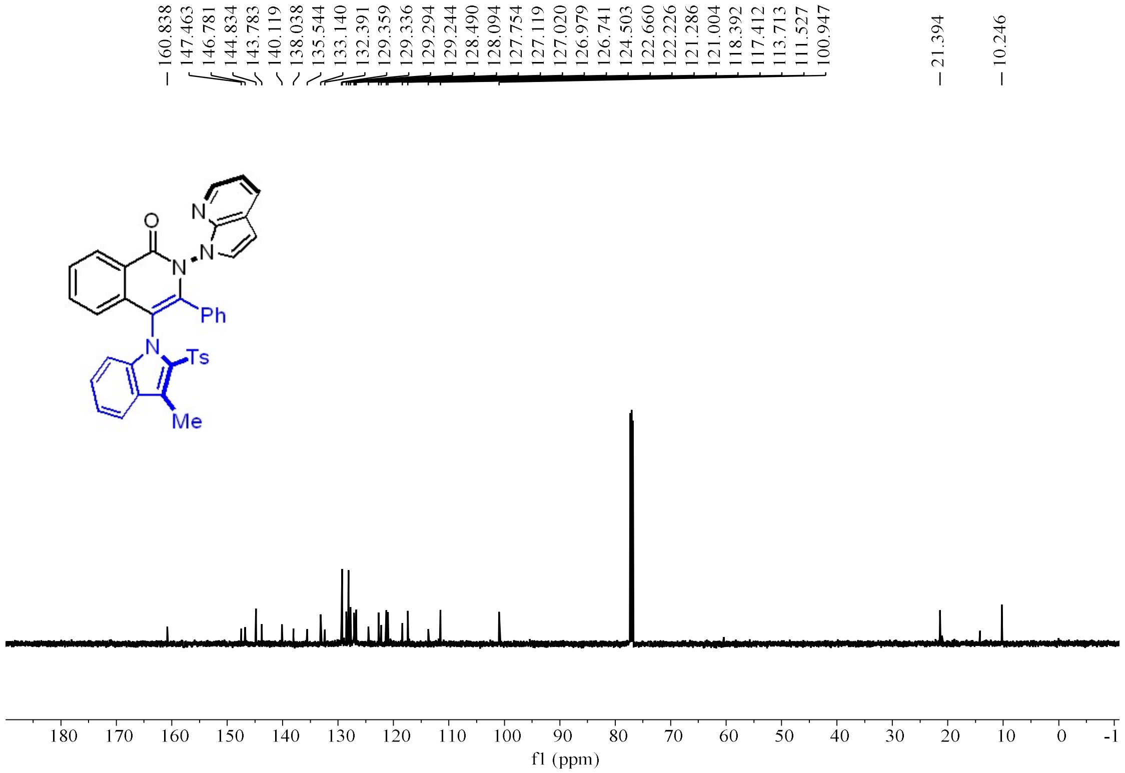


**Supplementary Figure 164.** **$^{13}$C NMR (150 MHz, $CDCl_3$) spectrum of 19.**

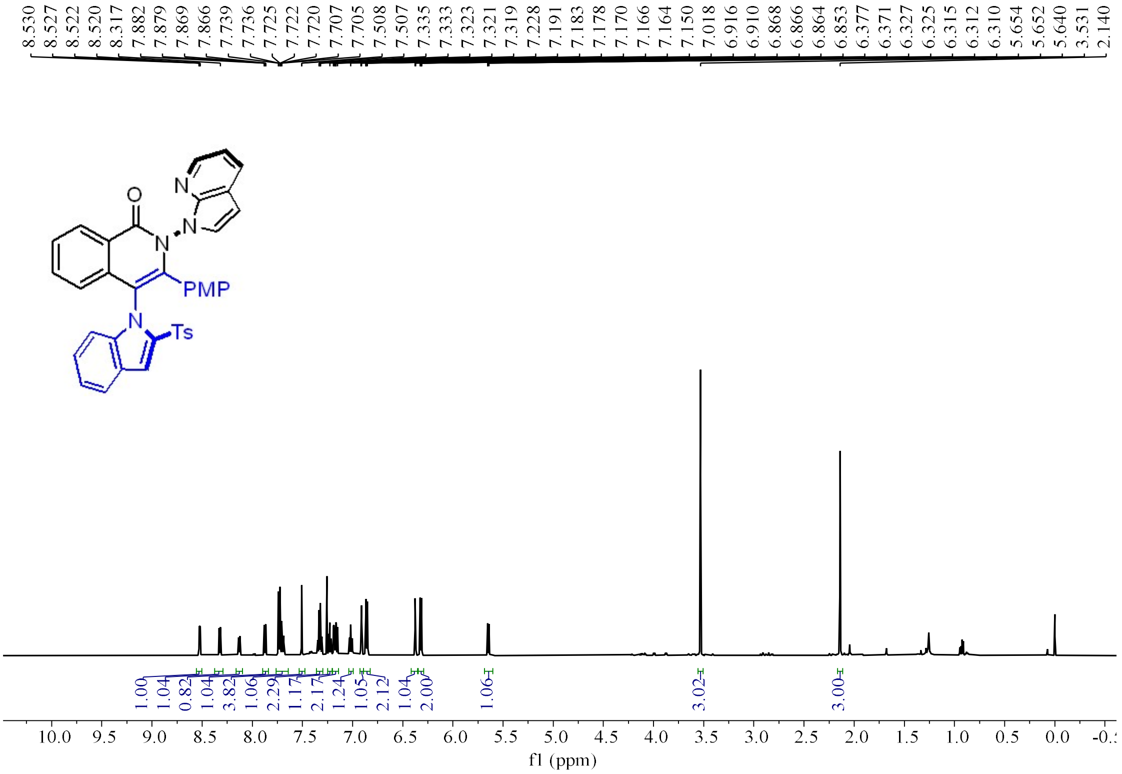


**Supplementary Figure 165. ¹H NMR (600 MHz, CDCl₃) spectrum of 20.**

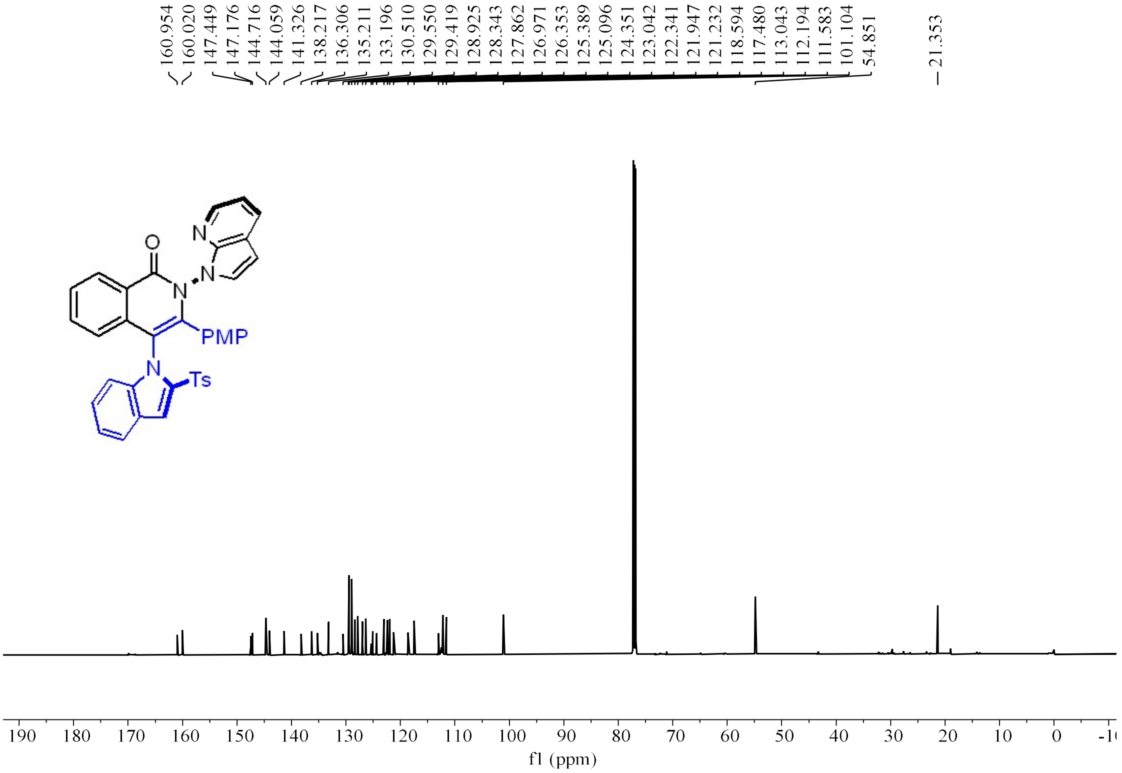


**Supplementary Figure 166. ¹³C NMR (150 MHz, CDCl₃) spectrum of 20.**

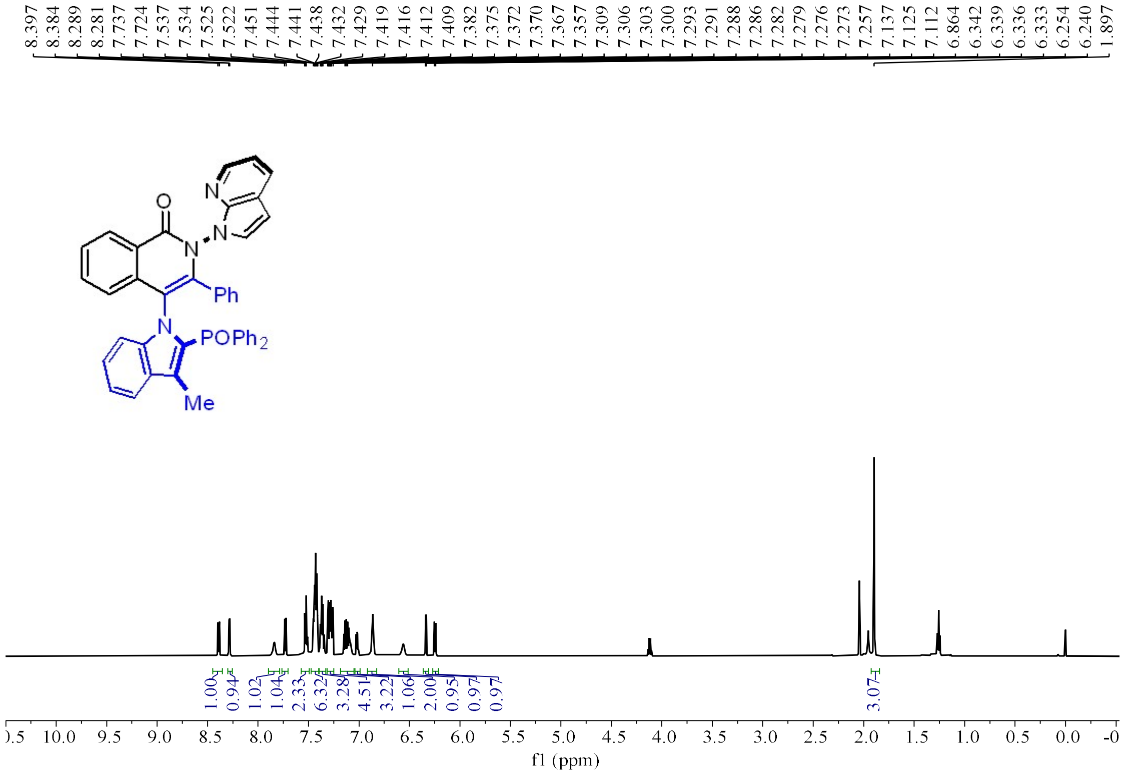

Supplementary Figure 167. $^1$H NMR (600 MHz, $CDCl_3$) spectrum of 21.

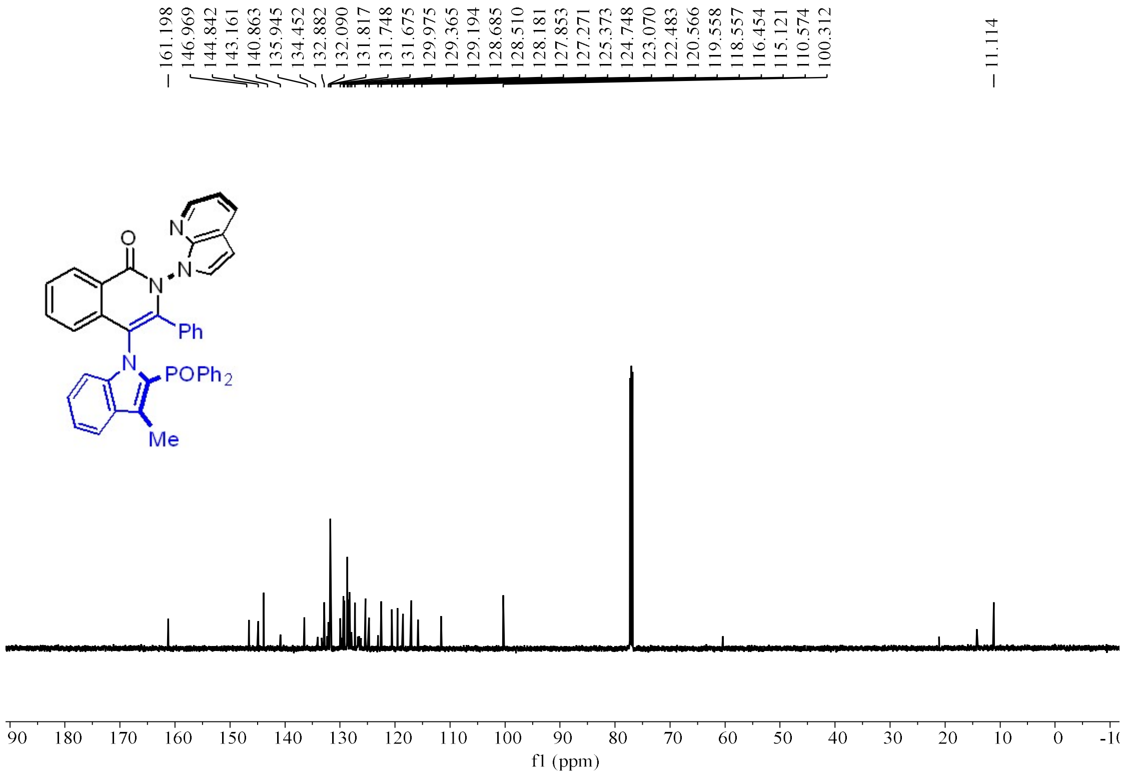

Supplementary Figure 168. $^{13}$C NMR (150 MHz, $CDCl_3$) spectrum of 21.

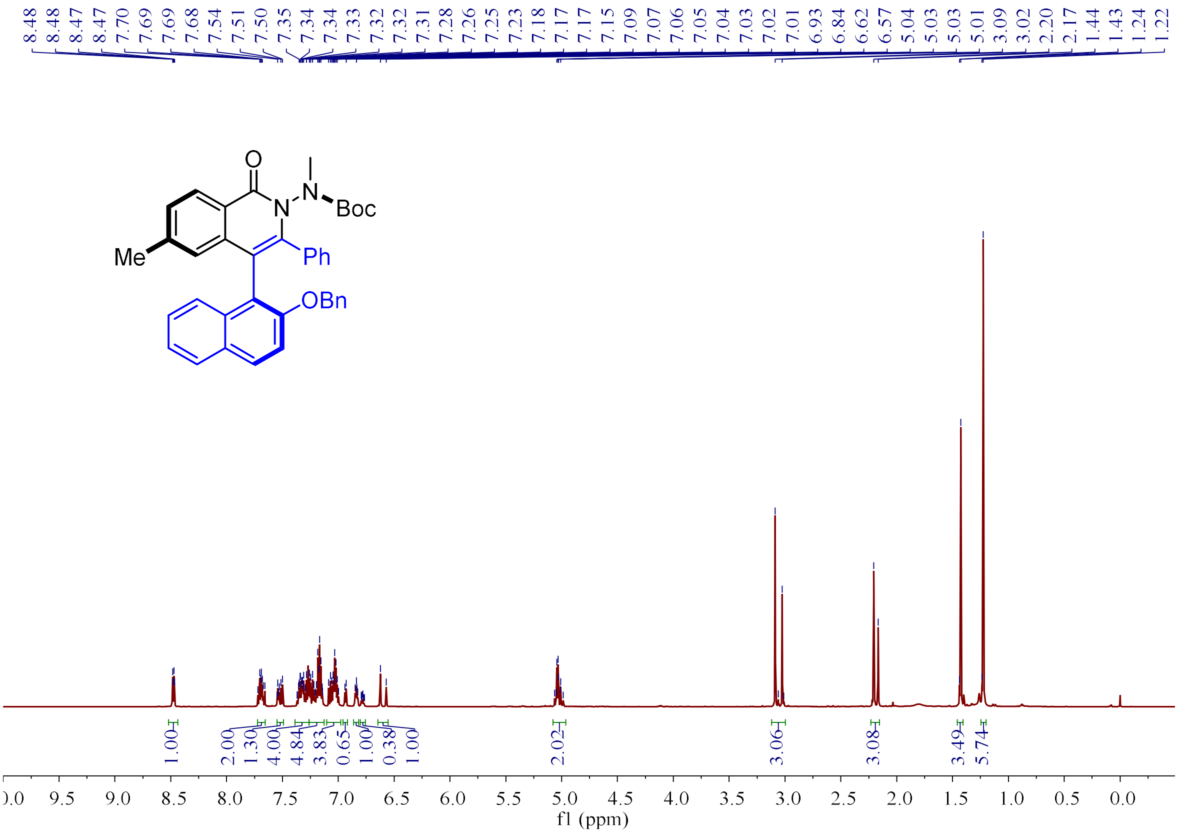


**Supplementary Figure 169. $^{1}$H NMR (600 MHz, CDCl$_3$) spectrum of 24.**

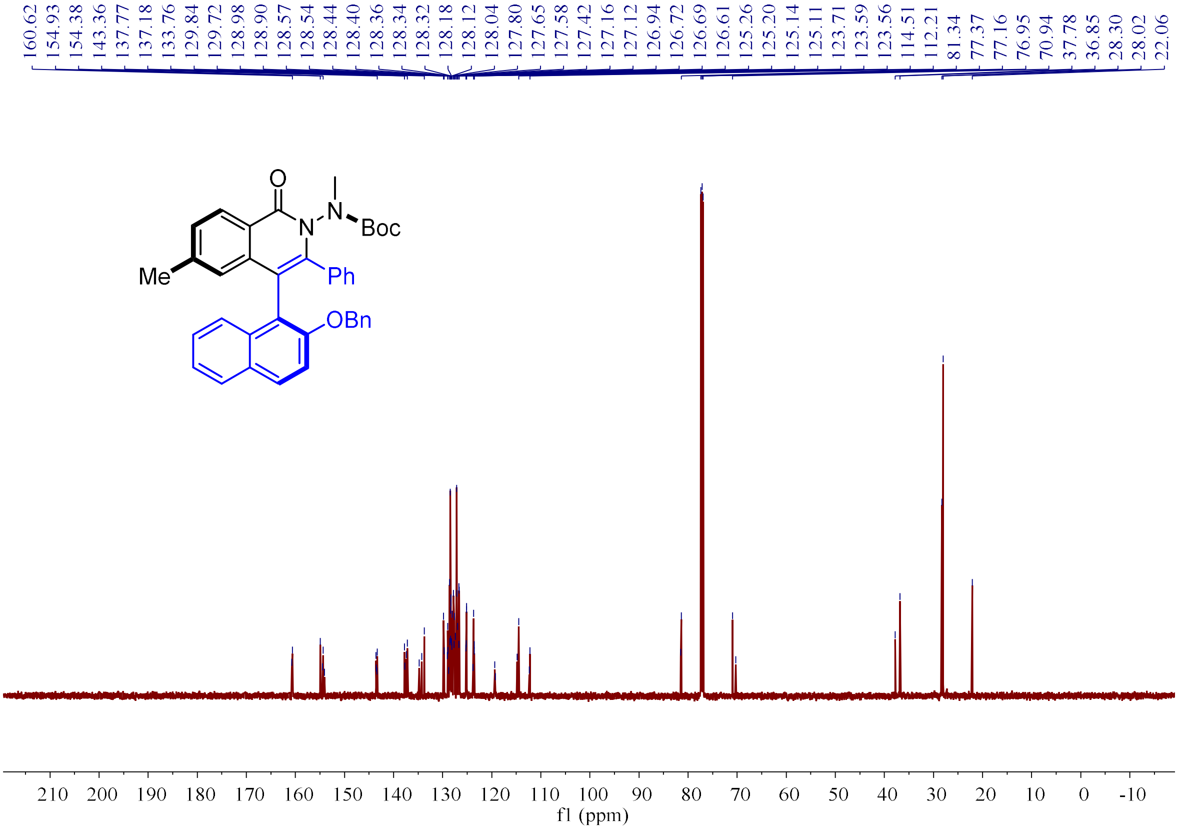


**Supplementary Figure 170. $^{13}$C NMR (150 MHz, CDCl$_3$) spectrum of 24.**

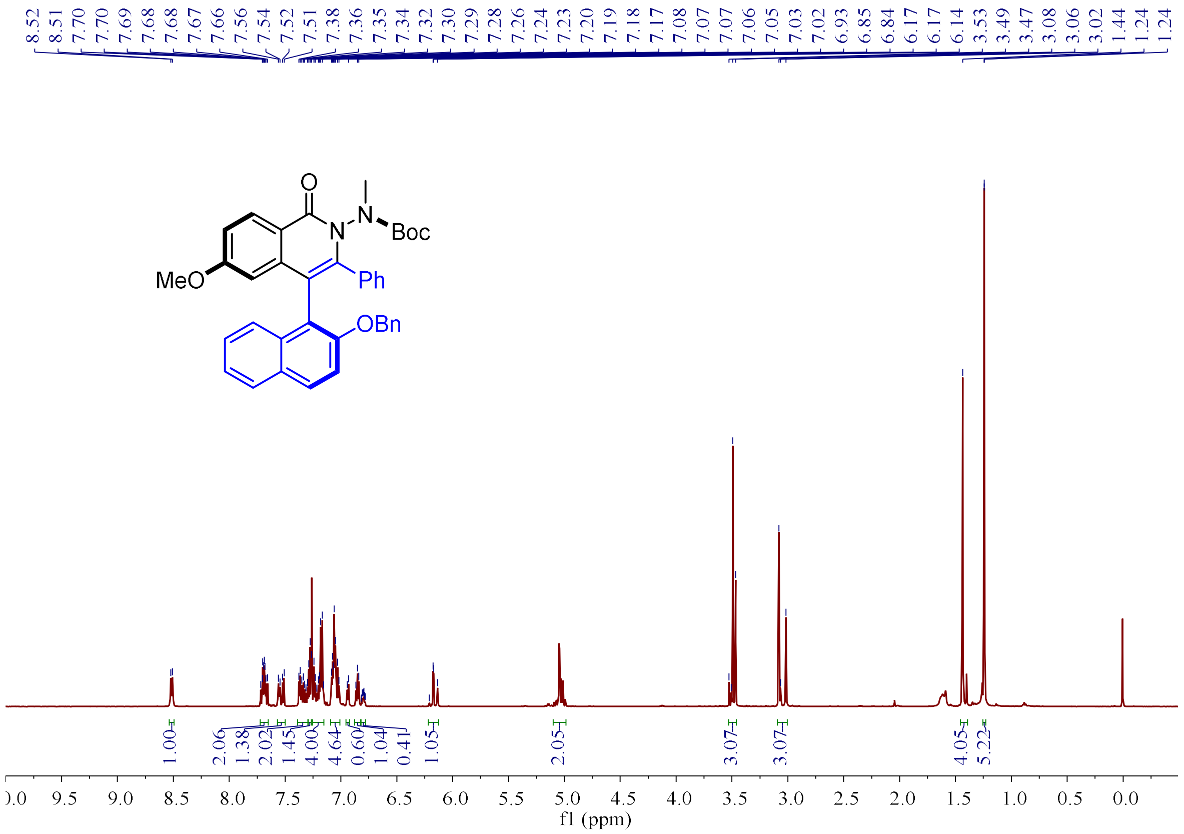

**Supplementary Figure 171. ¹H NMR (600 MHz, CDCl₃) spectrum of 25.**

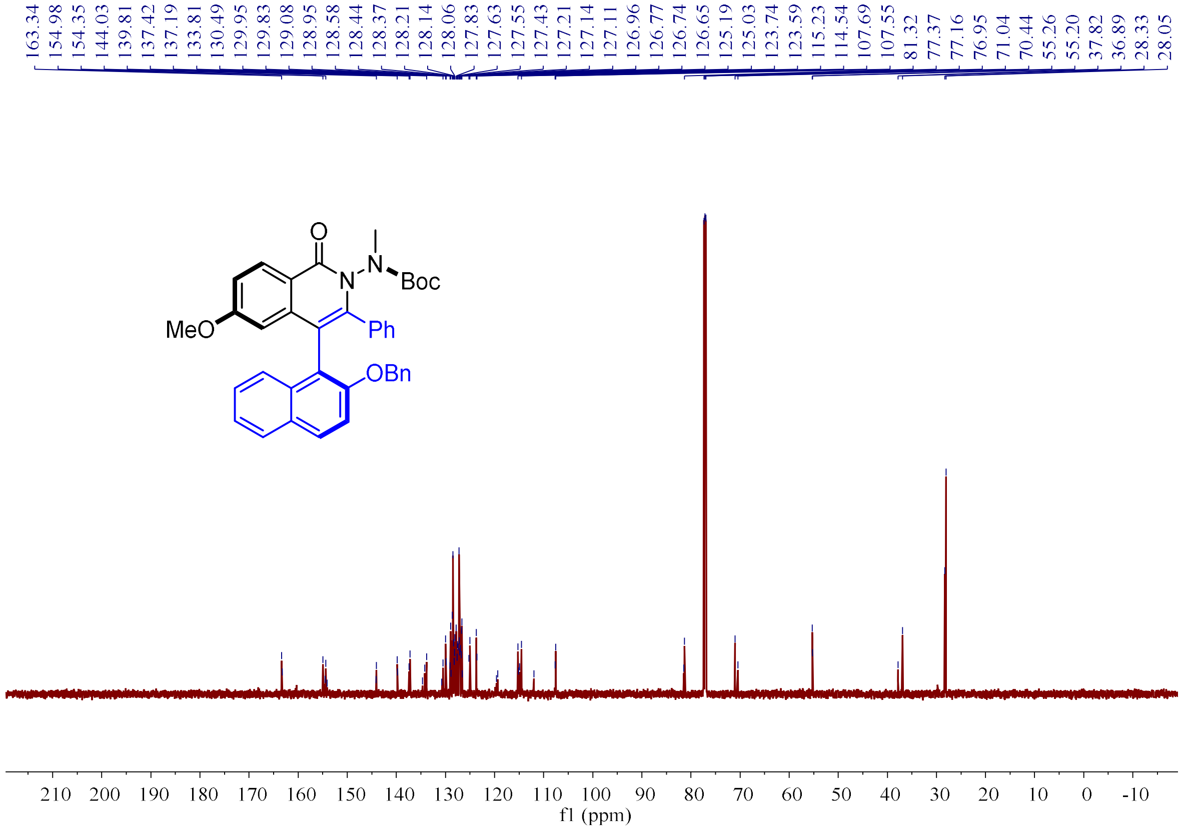

**Supplementary Figure 172. ¹³C NMR (150 MHz, CDCl₃) spectrum of 25.**

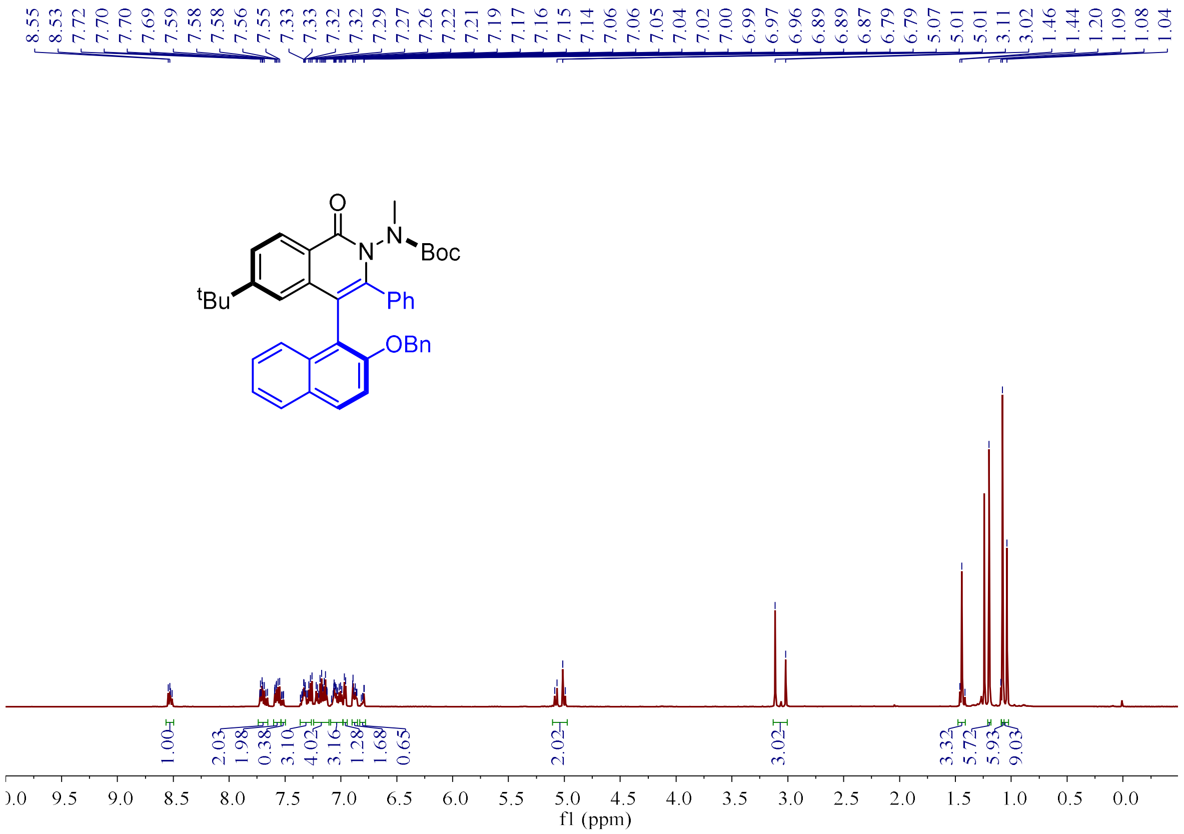

**Supplementary Figure 173. $^{1}$H NMR (600 MHz, CDCl$_3$) spectrum of 26.**

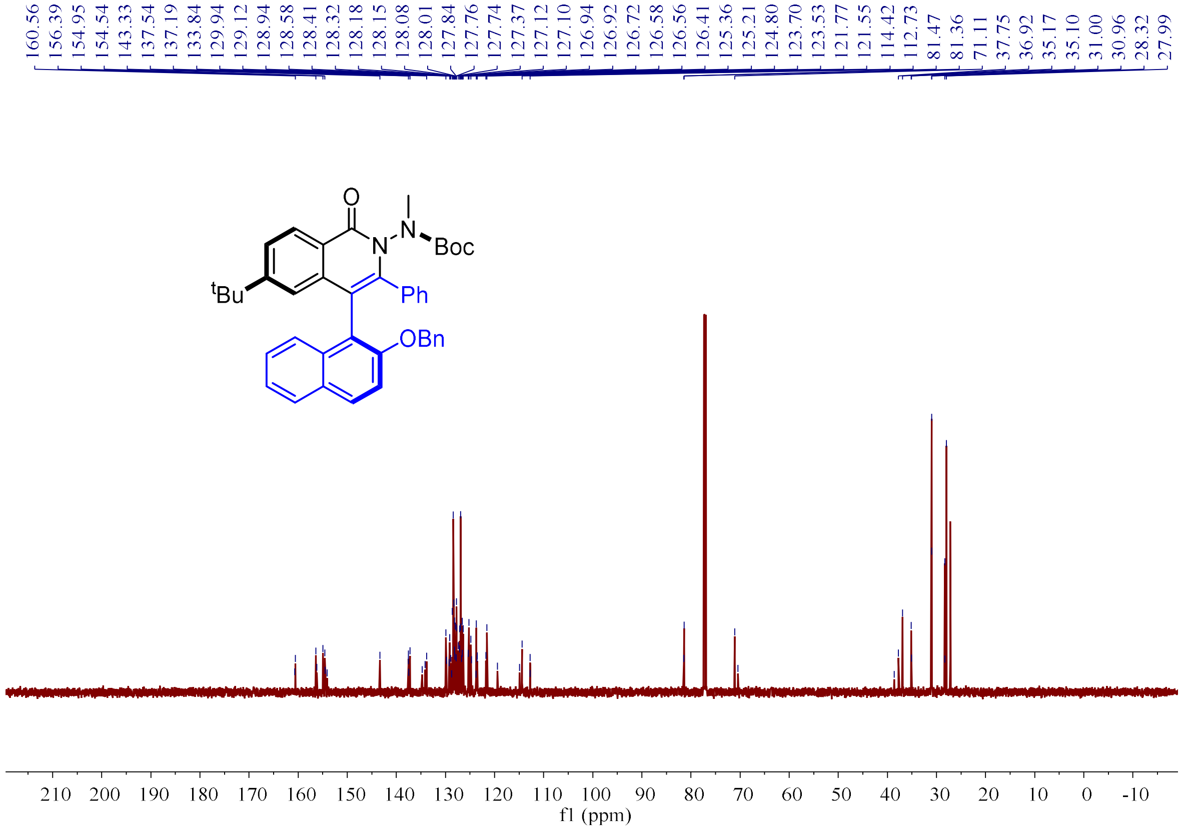

**Supplementary Figure 174. $^{13}$C NMR (150 MHz, CDCl$_3$) spectrum of 26.**

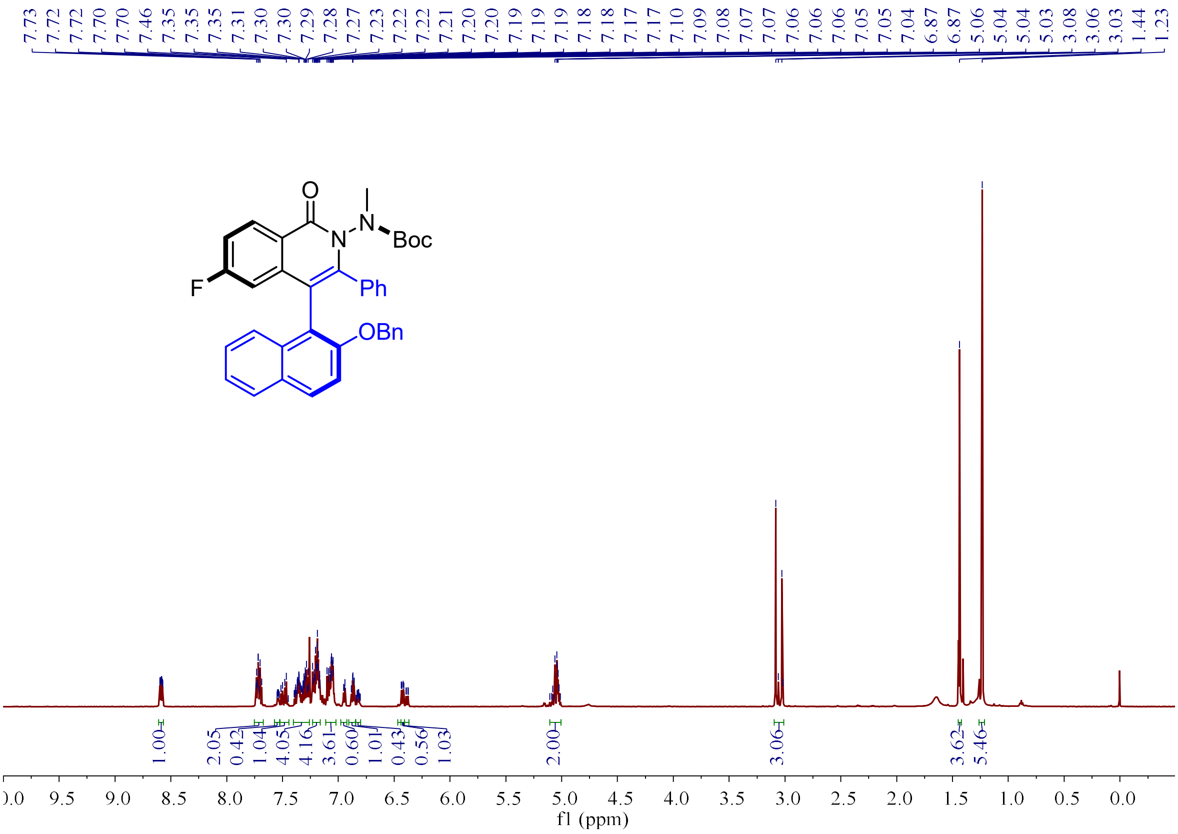


**Supplementary Figure 175. $^{1}$H NMR (600 MHz, CDCl$_3$) spectrum of 27.**

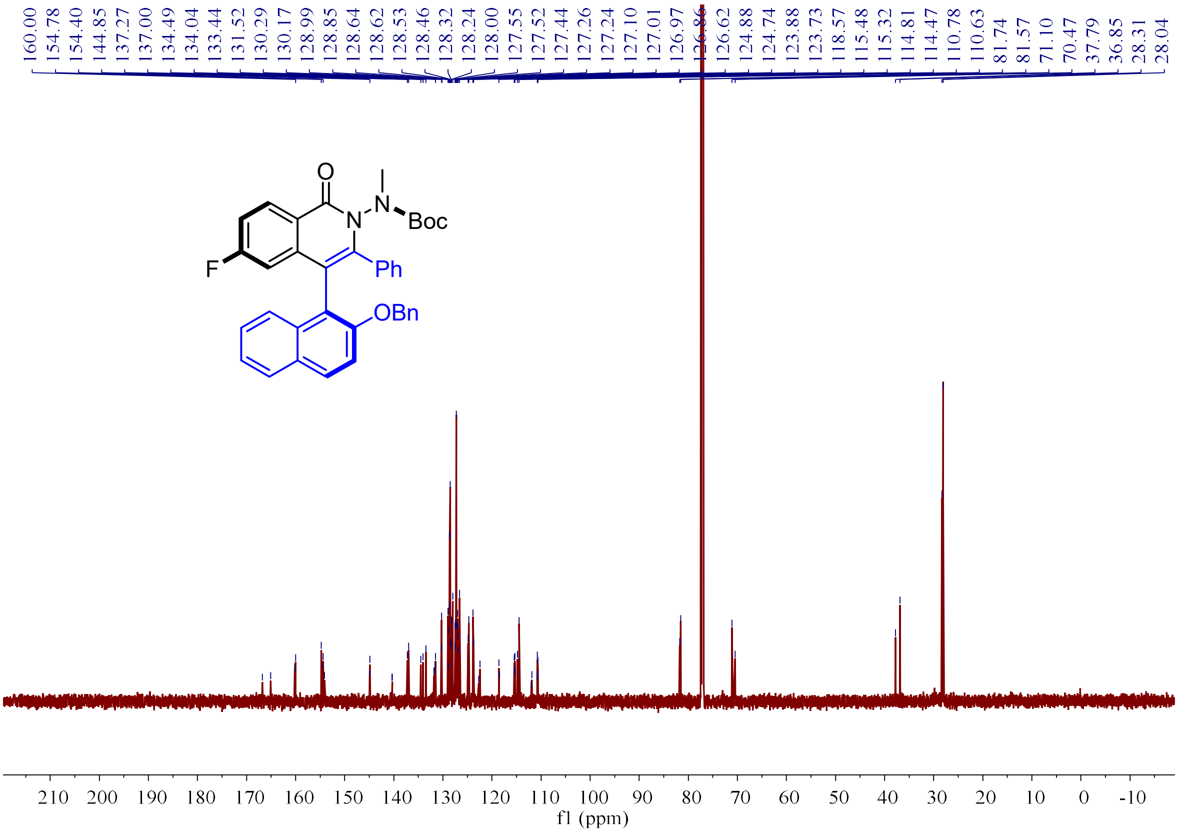


**Supplementary Figure 176. $^{13}$C NMR (150 MHz, CDCl$_3$) spectrum of 27.**

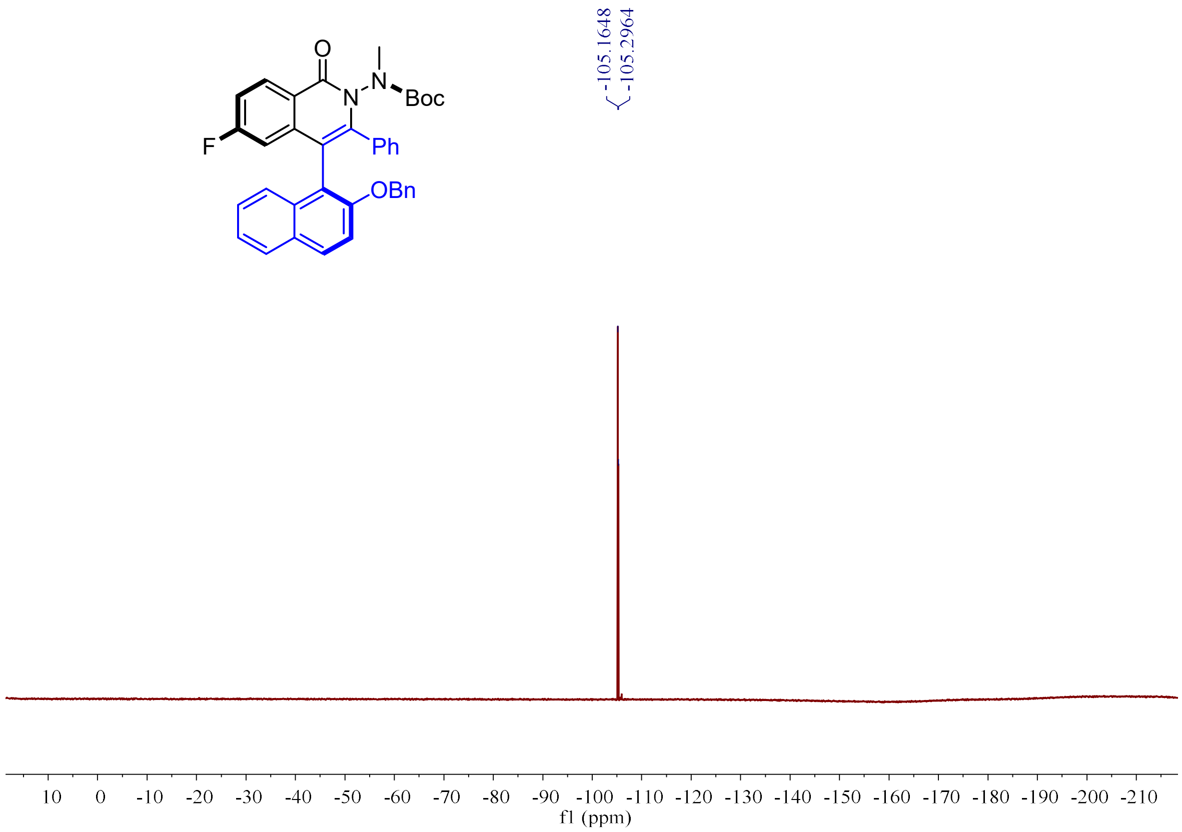


**Supplementary Figure 177. $^{19}F$ NMR (376 MHz, $CDCl_3$) spectrum of 27.**

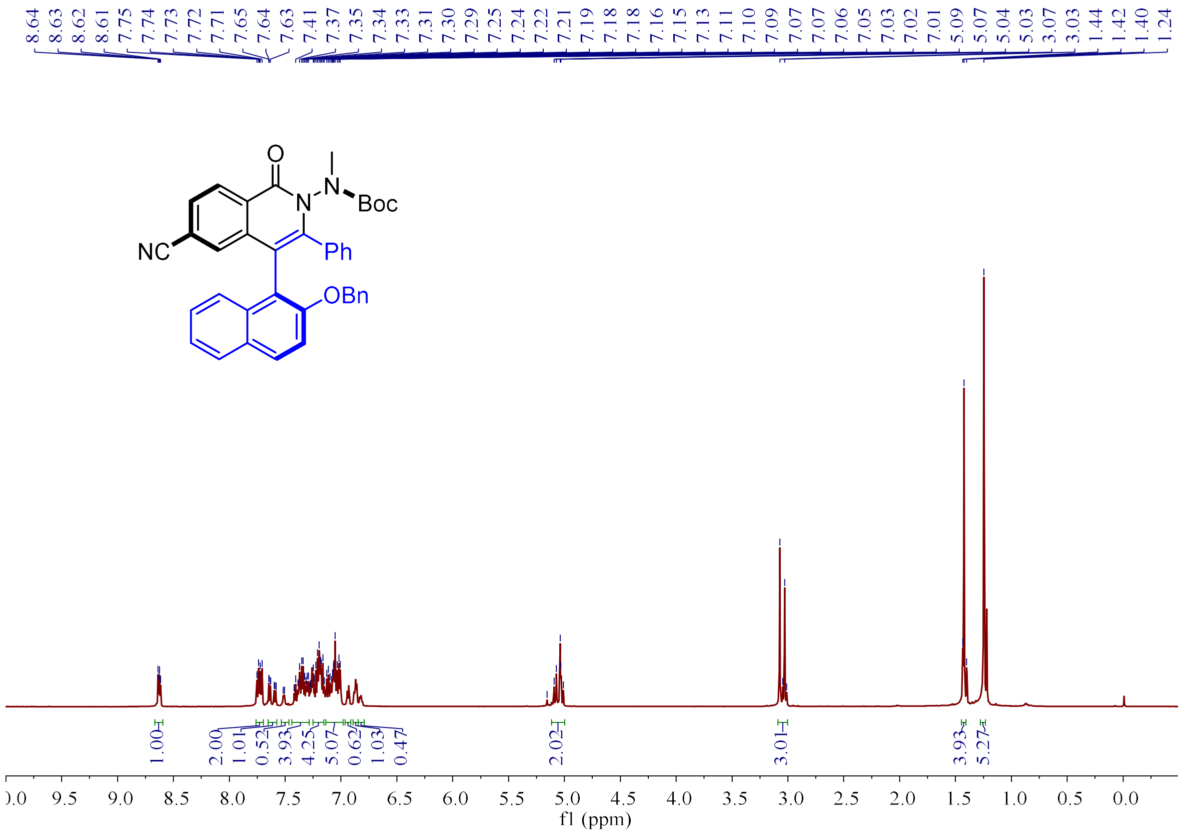


**Supplementary Figure 178. $^{1}$H NMR (600 MHz, $CDCl_3$) spectrum of 28.**

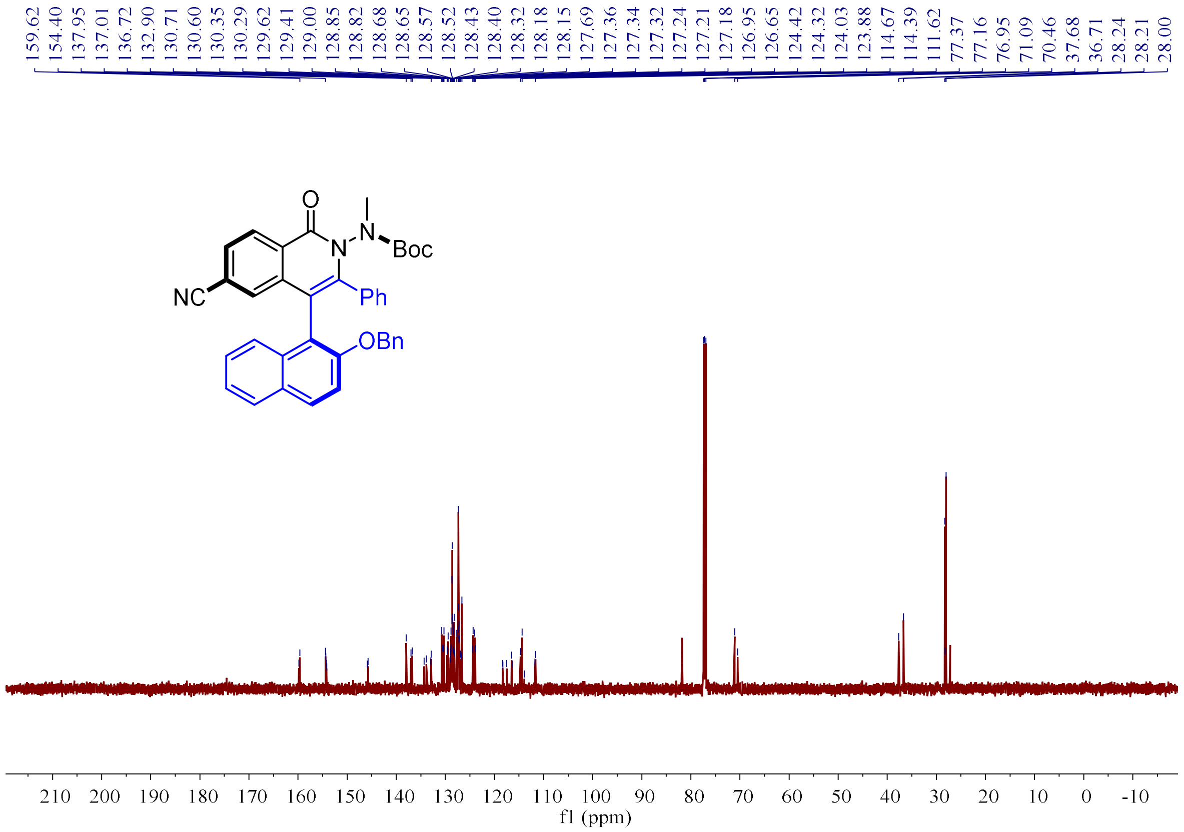


**Supplementary Figure 179. $^{13}$C NMR (150 MHz, $CDCl_3$) spectrum of 28.**

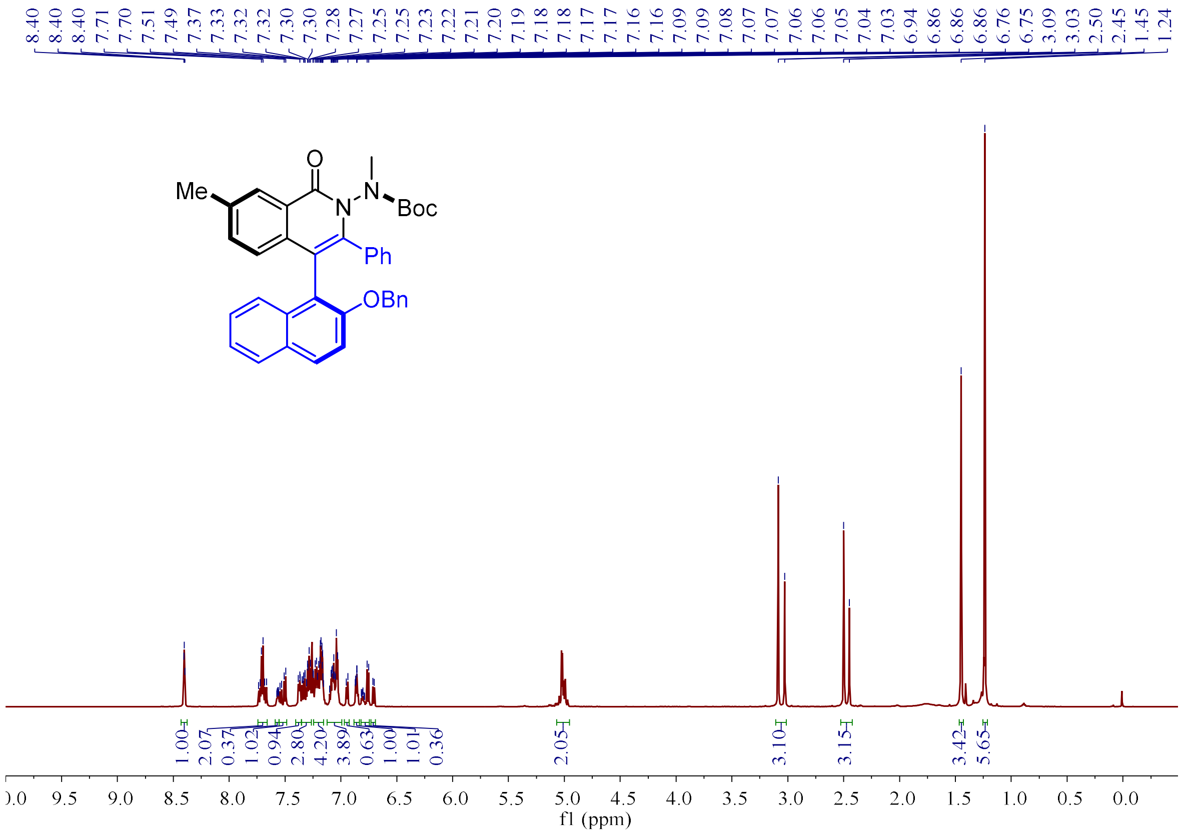

**Supplementary Figure 180. $^{1}$H NMR (600 MHz, CDCl$_3$) spectrum of 29.**

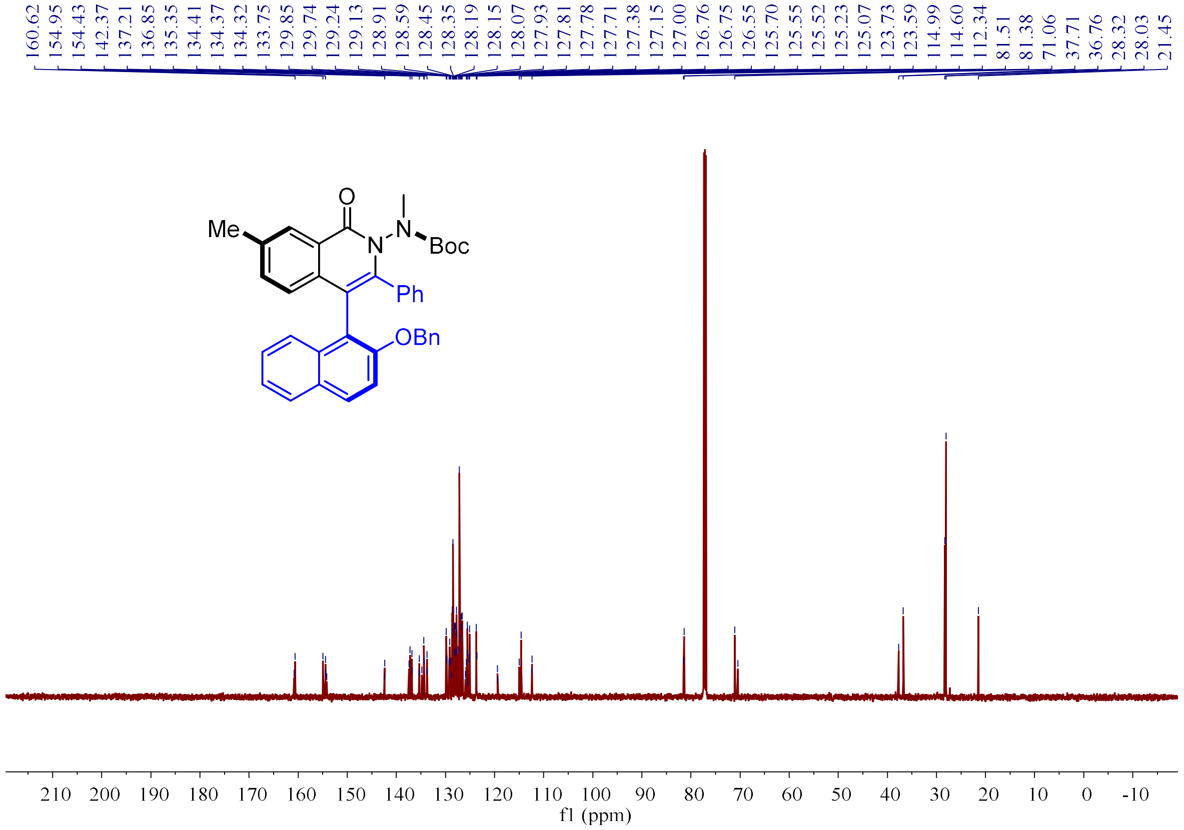

**Supplementary Figure 181. $^{13}$C NMR (150 MHz, CDCl$_3$) spectrum of 29.**

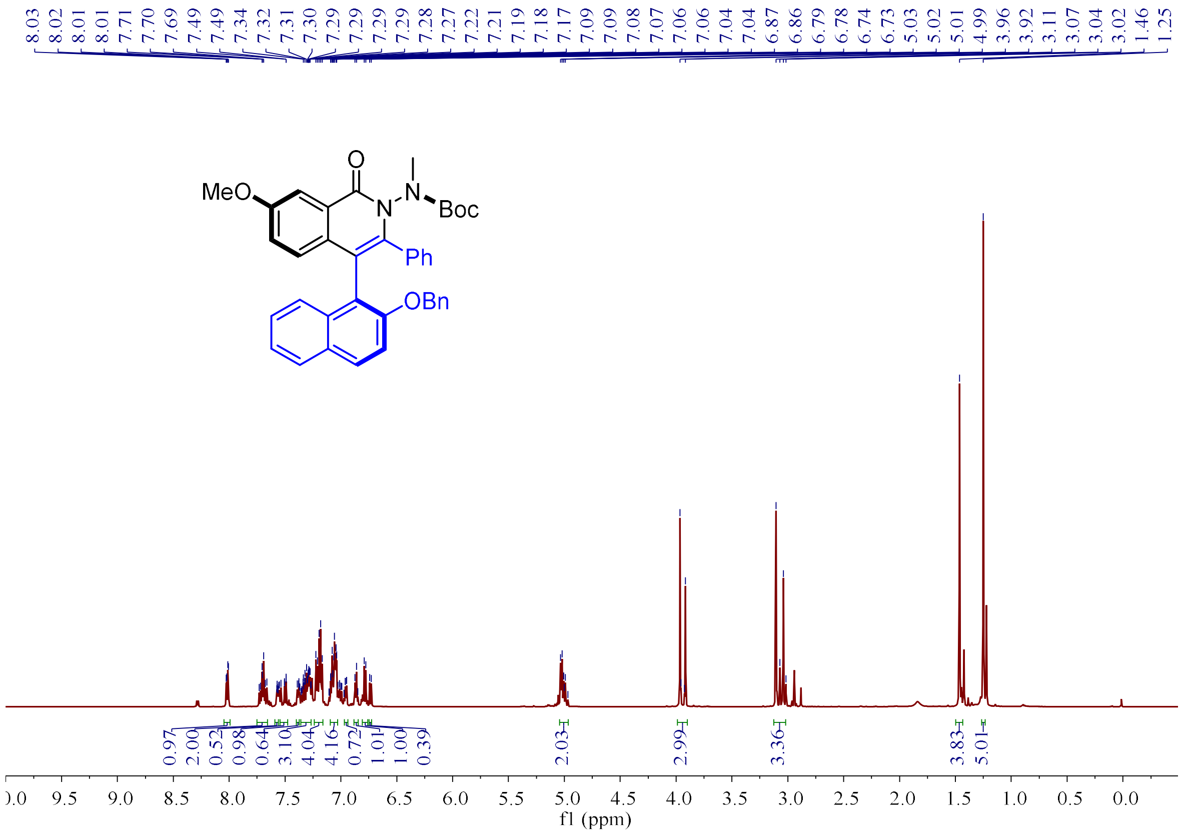


**Supplementary Figure 182. $^{1}$H NMR (600 MHz, $CDCl_3$) spectrum of 30.**

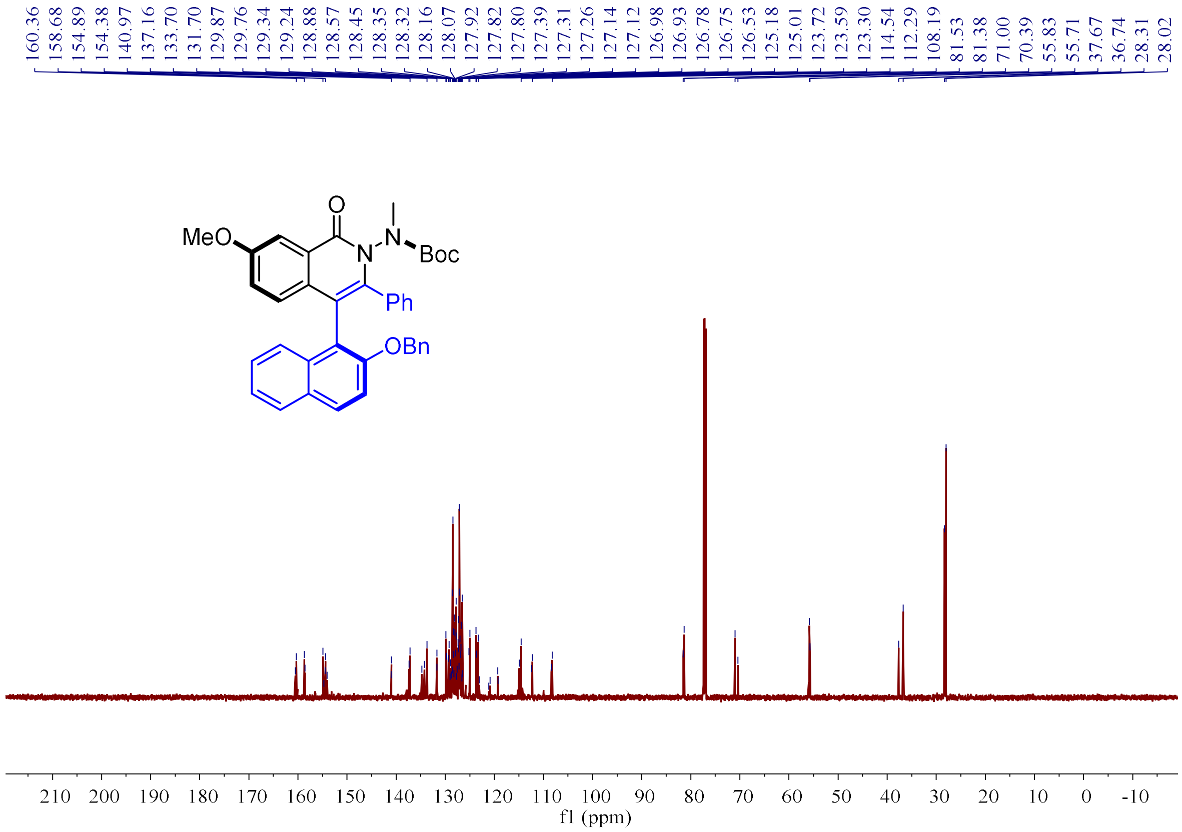


**Supplementary Figure 183. $^{13}$C NMR (150 MHz, $CDCl_3$) spectrum of 30.**

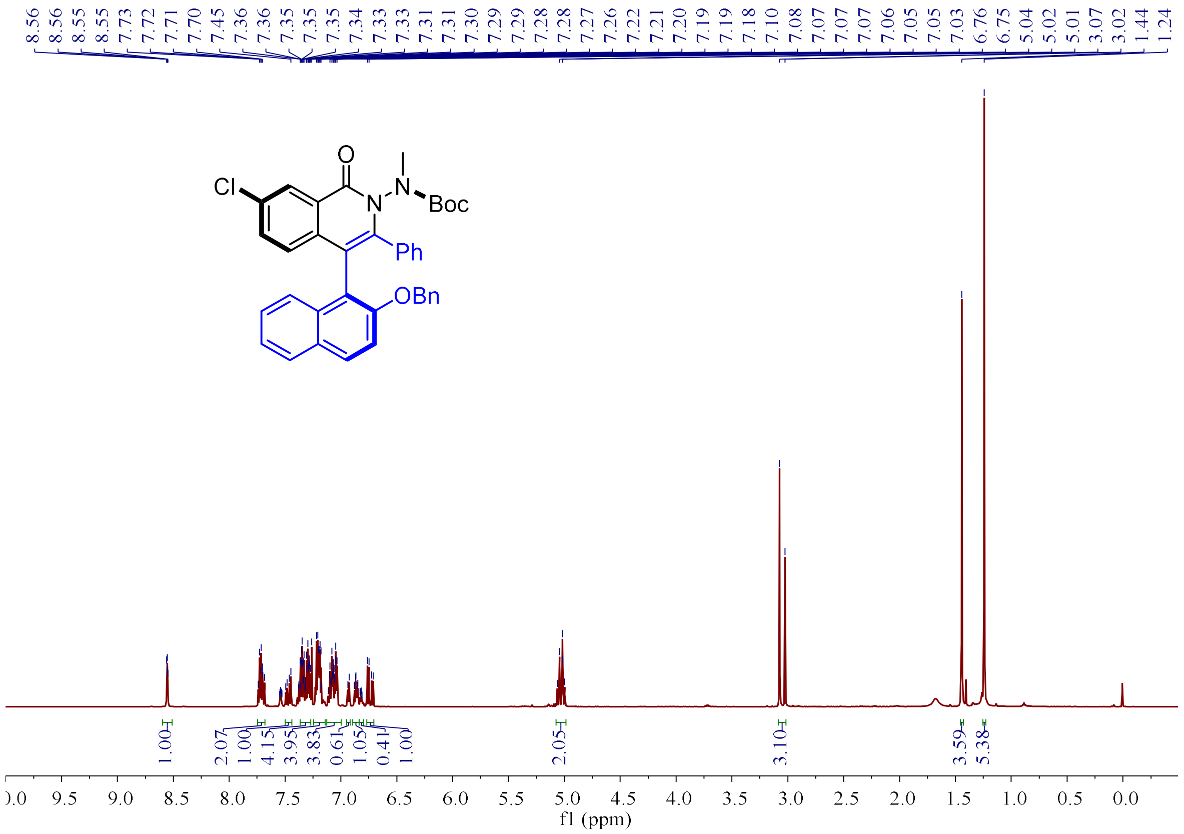


**Supplementary Figure 184. $^{1}$H NMR (600 MHz, CDCl$_3$) spectrum of 31.**

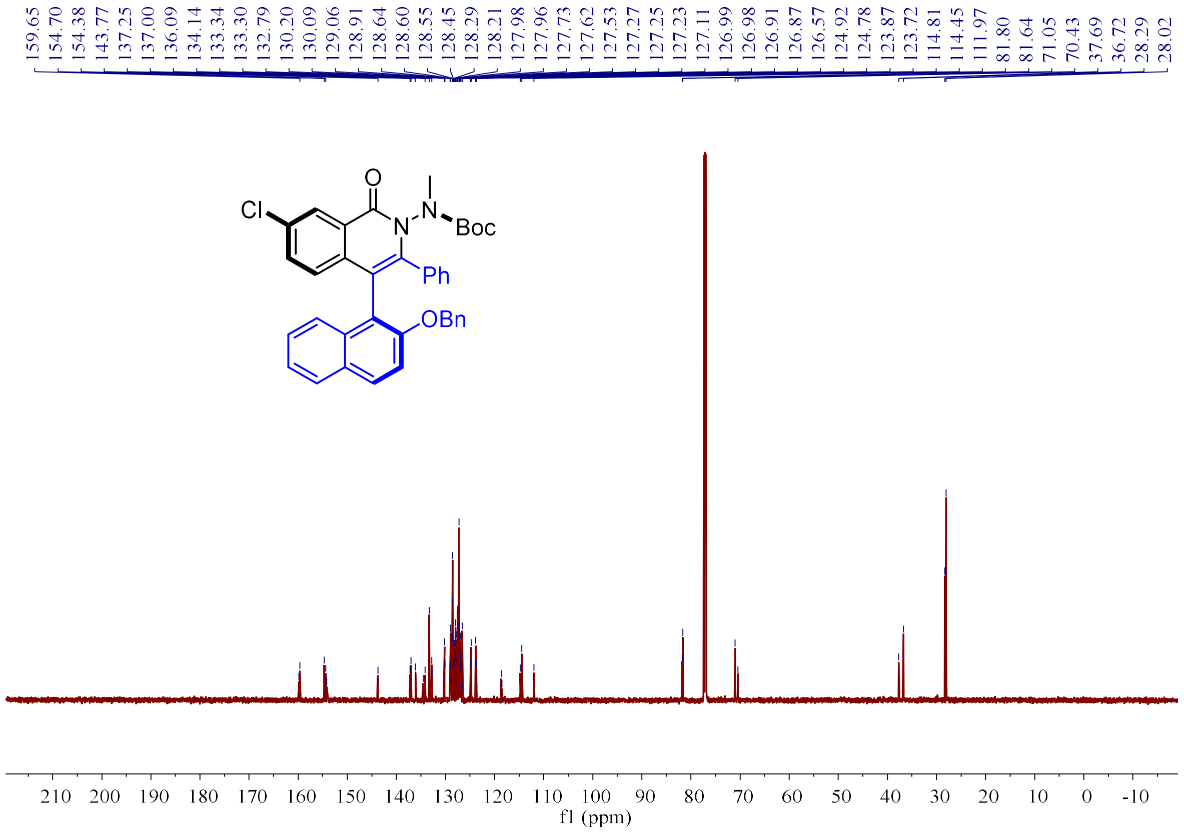


**Supplementary Figure 185. $^{13}$C NMR (150 MHz, CDCl$_3$) spectrum of 31.**

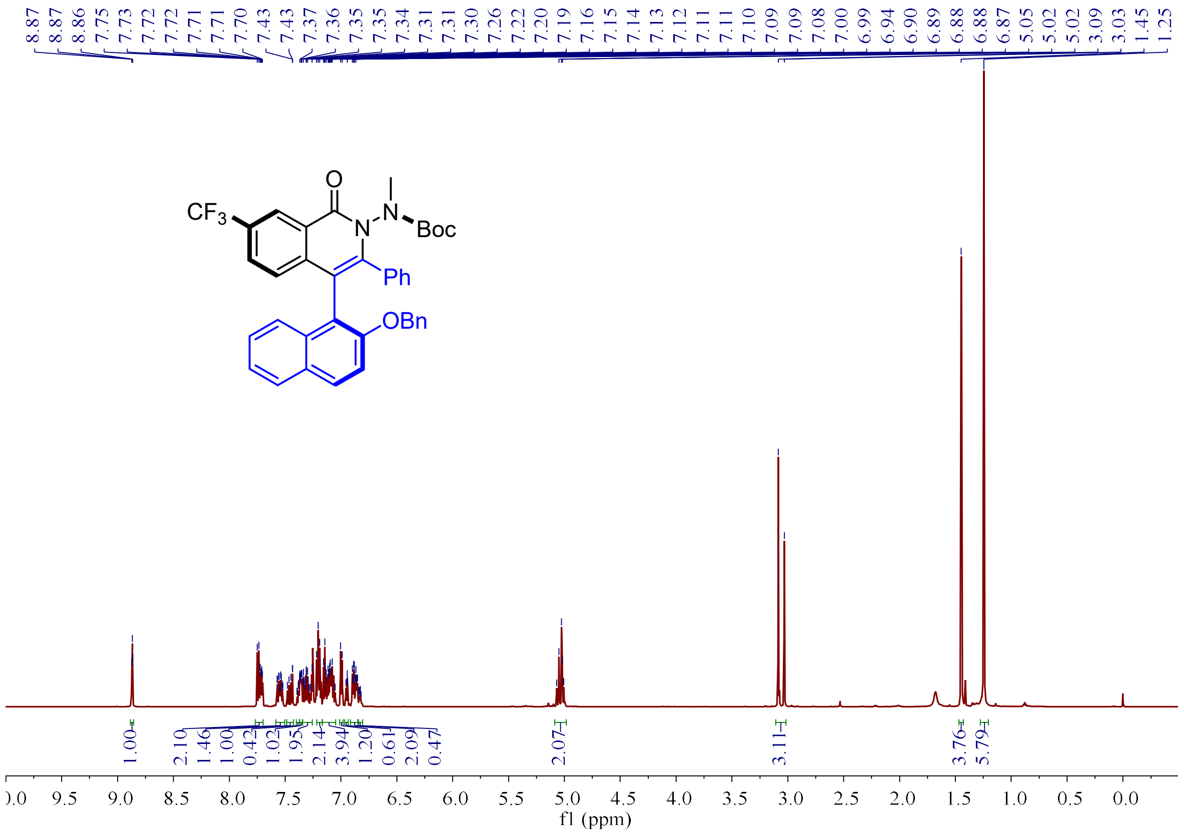

**Supplementary Figure 186. $^{1}$H NMR (600 MHz, $CDCl_3$) spectrum of 32.**

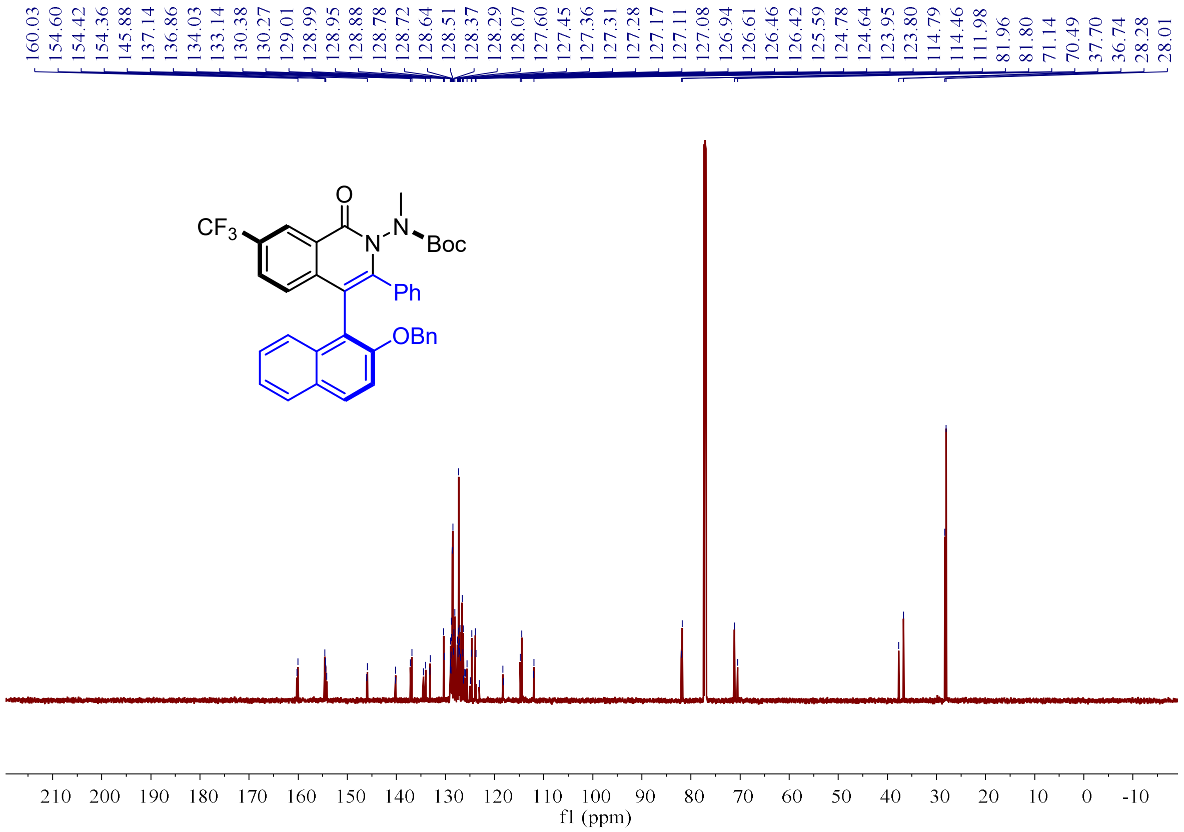

**Supplementary Figure 187. $^{13}$C NMR (150 MHz, $CDCl_3$) spectrum of 32.**

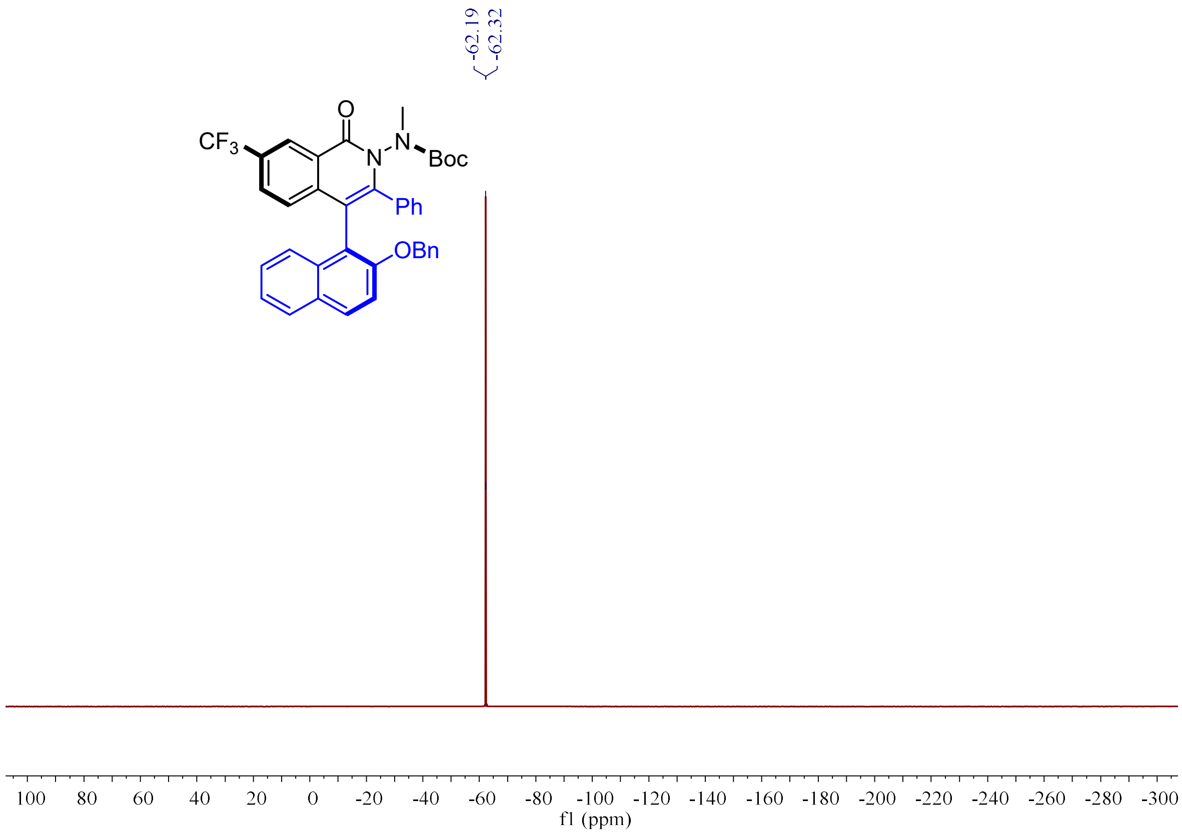


**Supplementary Figure 188. $^{19}$F NMR (376 MHz, CDCl$_3$) spectrum of 32.**

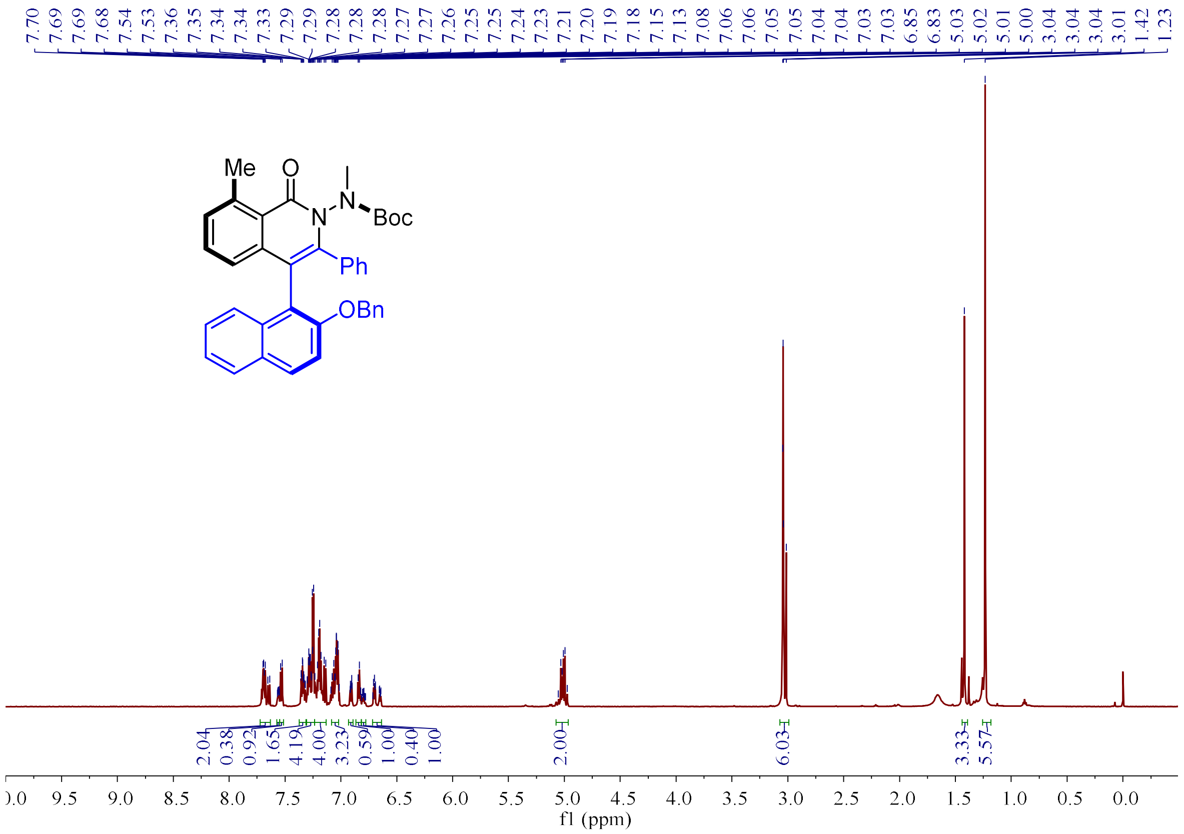


**Supplementary Figure 189.** $^{1}$**H NMR (600 MHz, CDCl**$_3$**) spectrum of 33.**

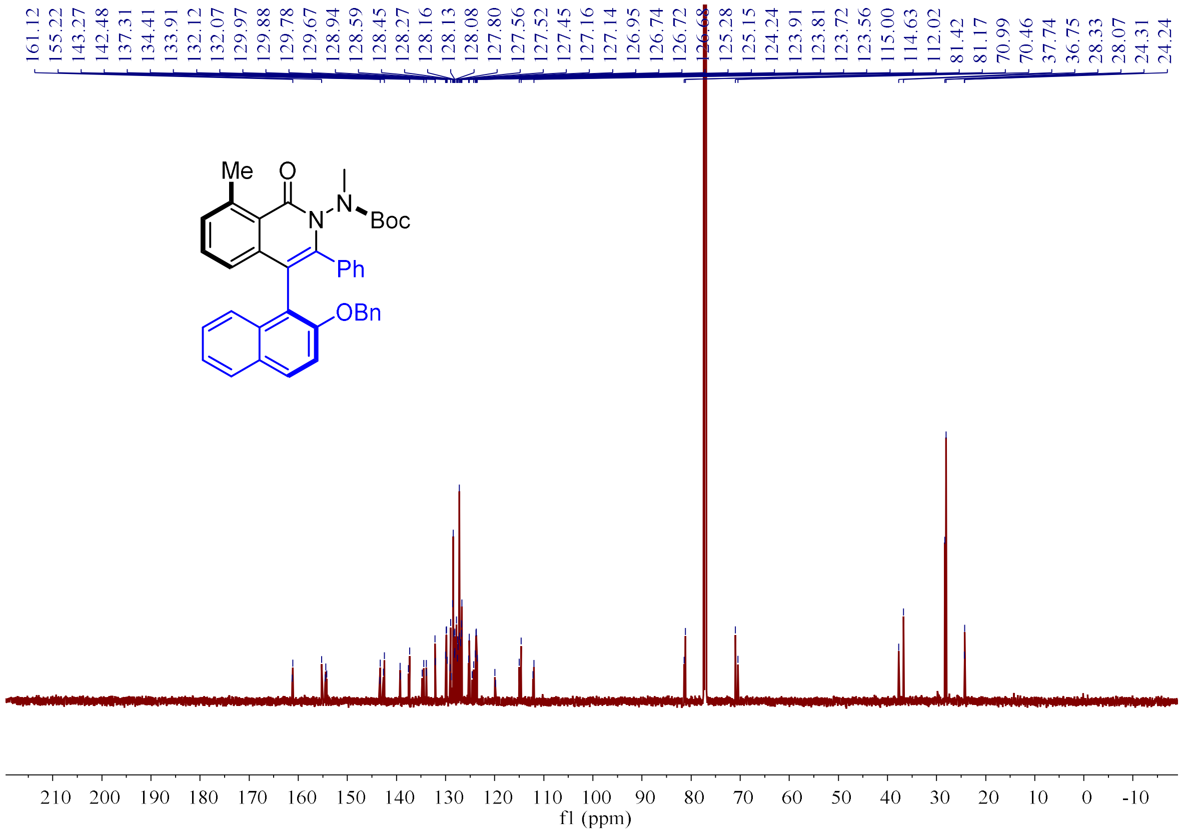


**Supplementary Figure 190.** $^{13}$**C NMR (150 MHz, CDCl**$_3$**) spectrum of 33.**

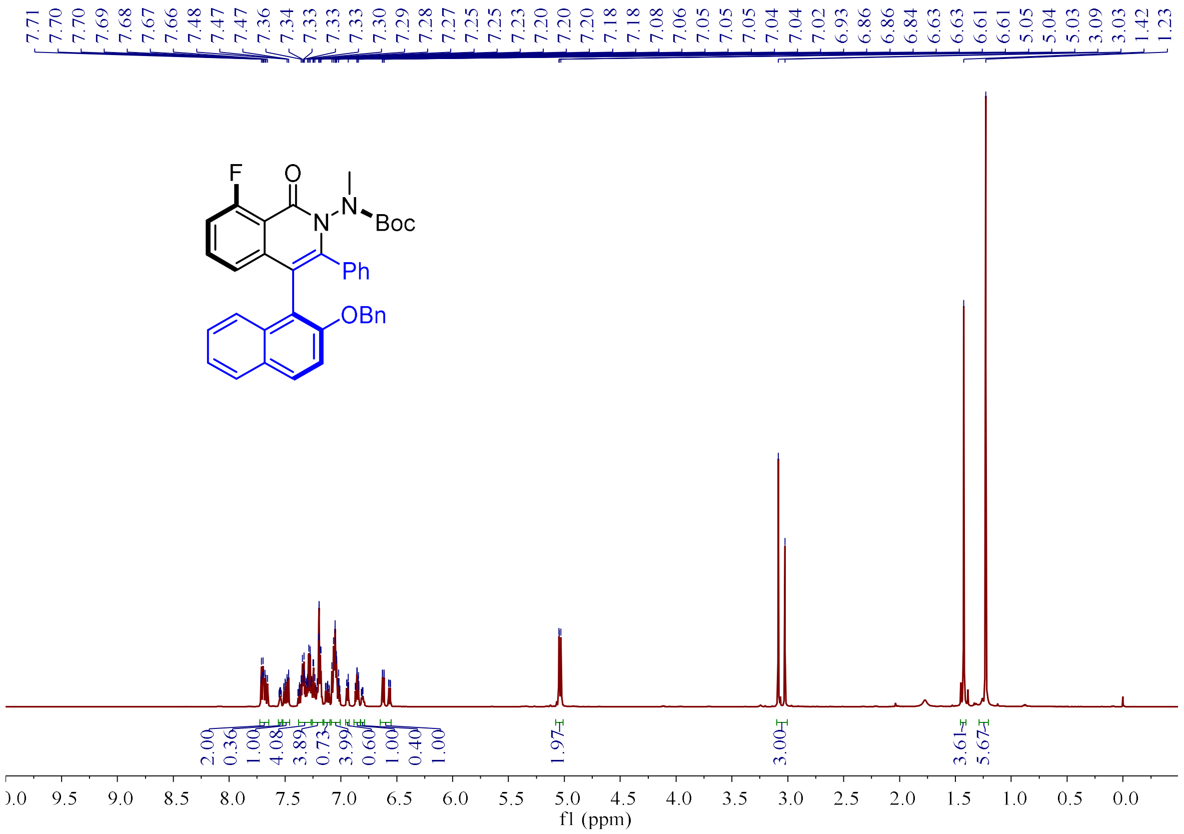


**Supplementary Figure 191. $^{1}H$ NMR (600 MHz, $CDCl_3$) spectrum of 34.**

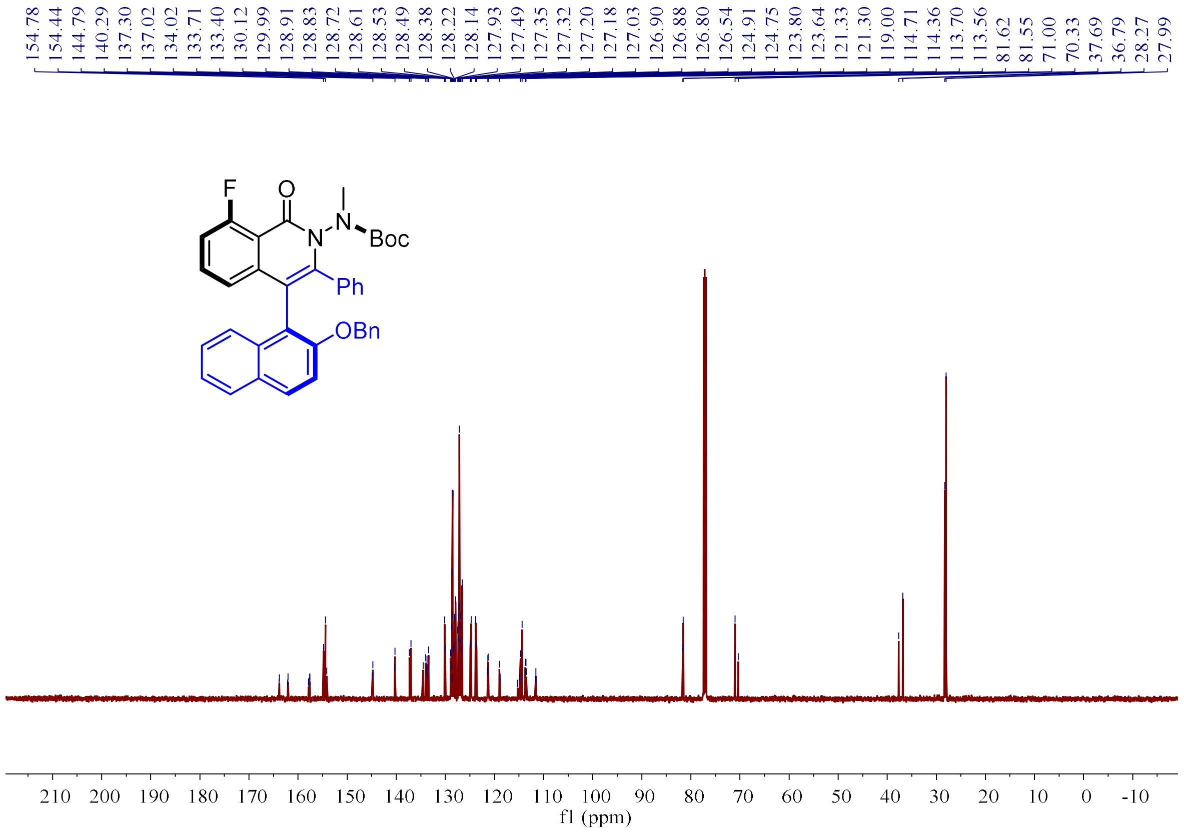


**Supplementary Figure 192. $^{13}C$ NMR (150 MHz, $CDCl_3$) spectrum of 34.**

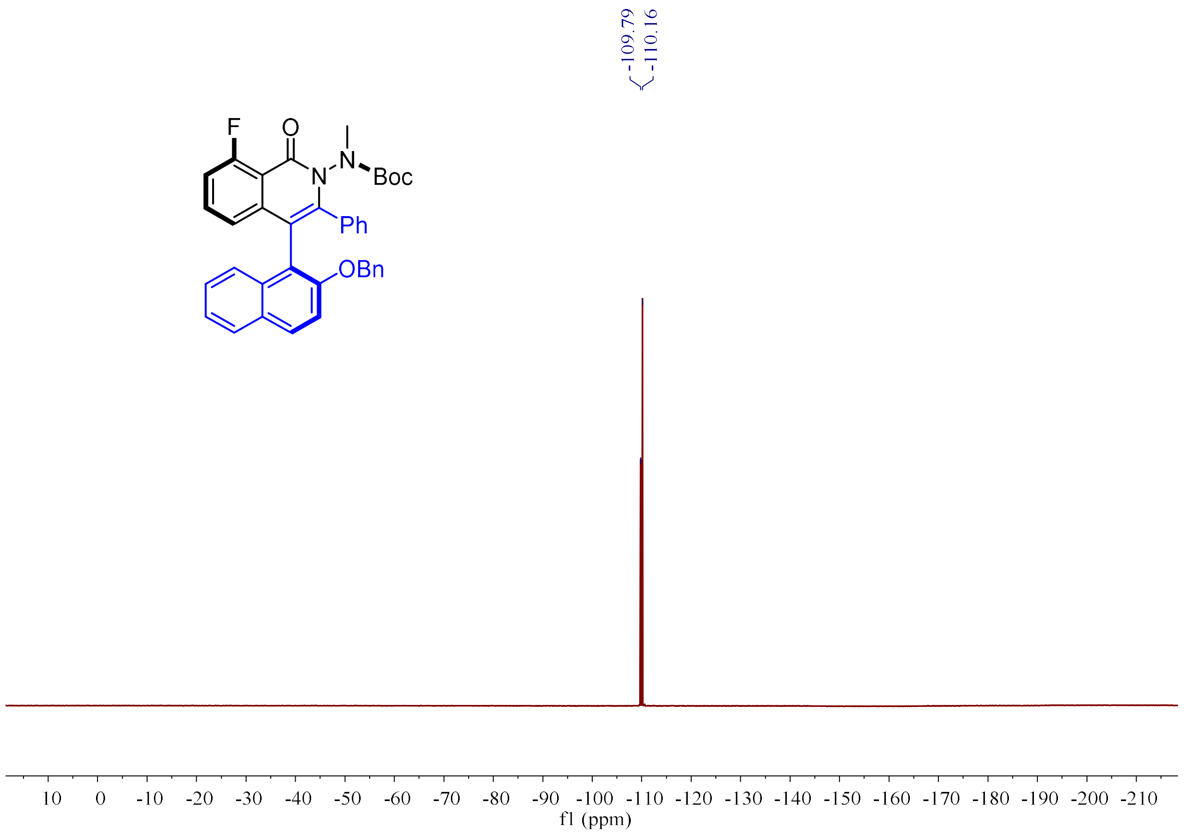


**Supplementary Figure 193. $^{19}$F NMR (376 MHz, $CDCl_3$) spectrum of 34.**

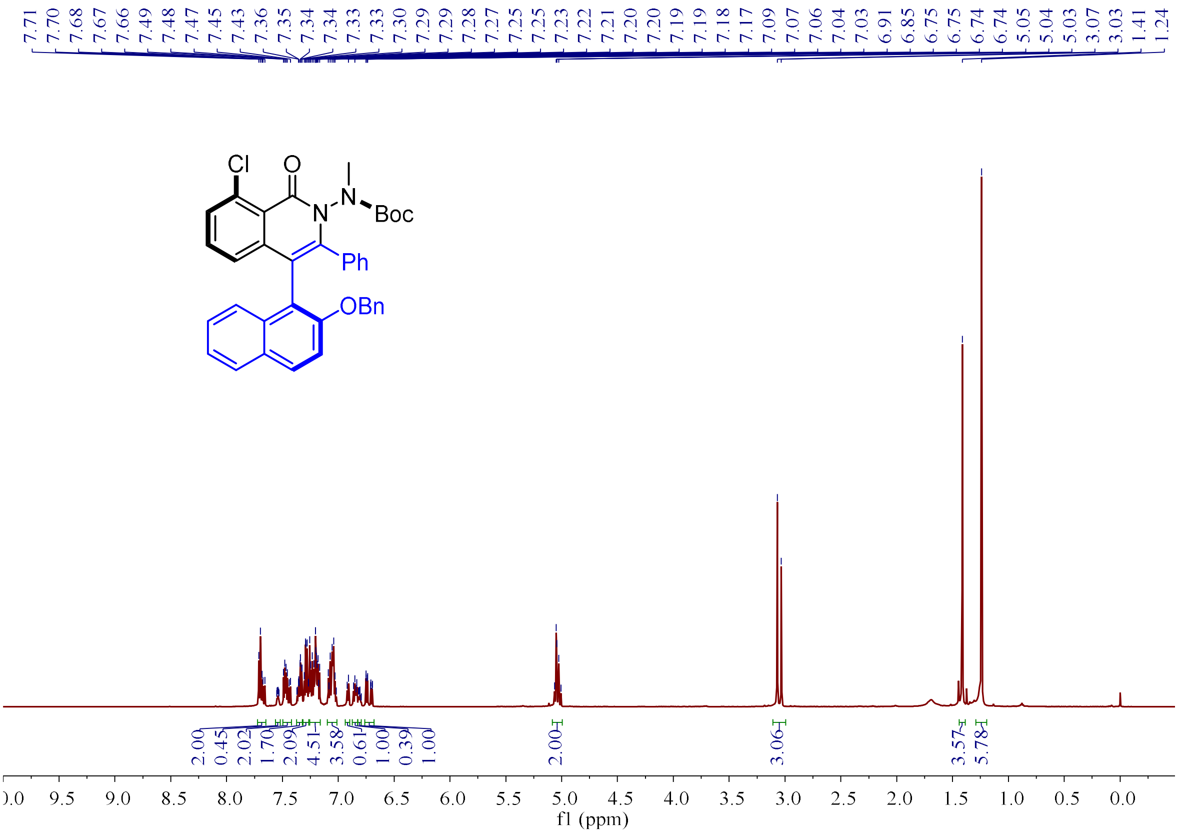


**Supplementary Figure 194. $^{1}$H NMR (600 MHz, $CDCl_3$) spectrum of 35.**

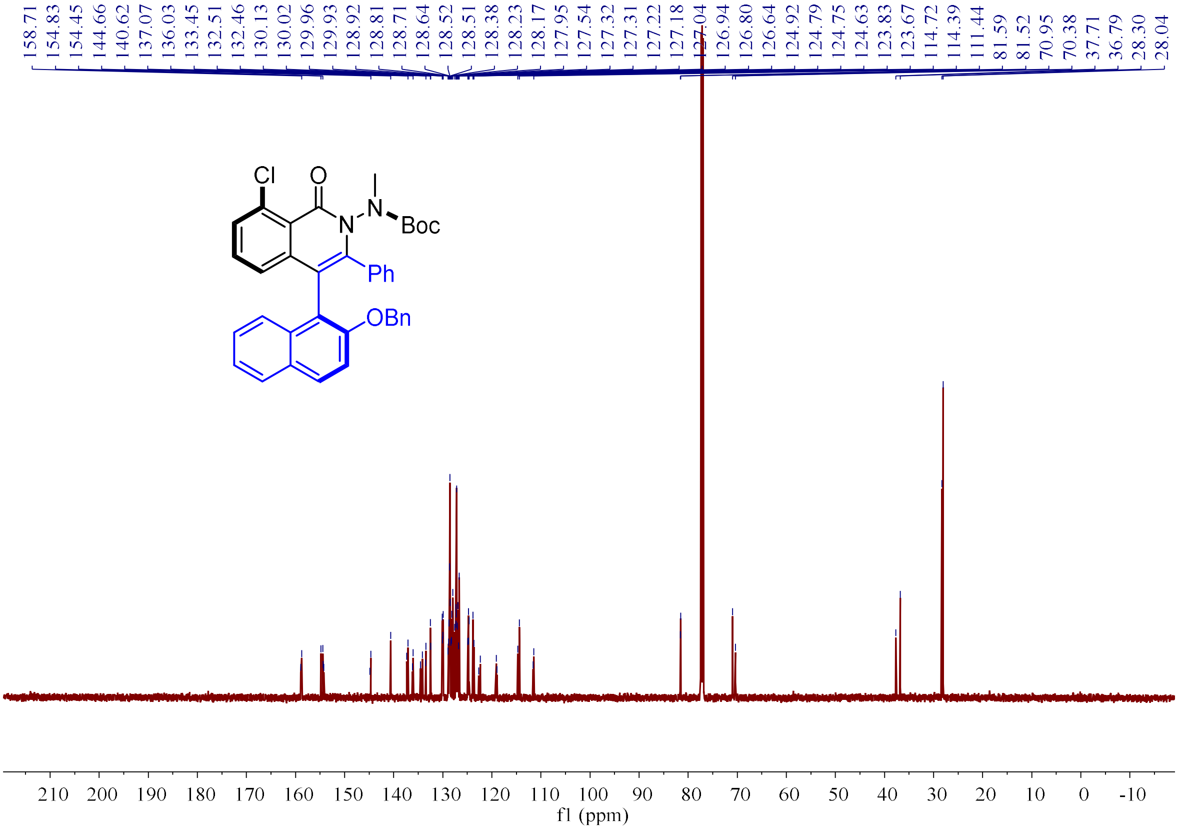


**Supplementary Figure 195. $^{13}$C NMR (150 MHz, $CDCl_3$) spectrum of 35.**

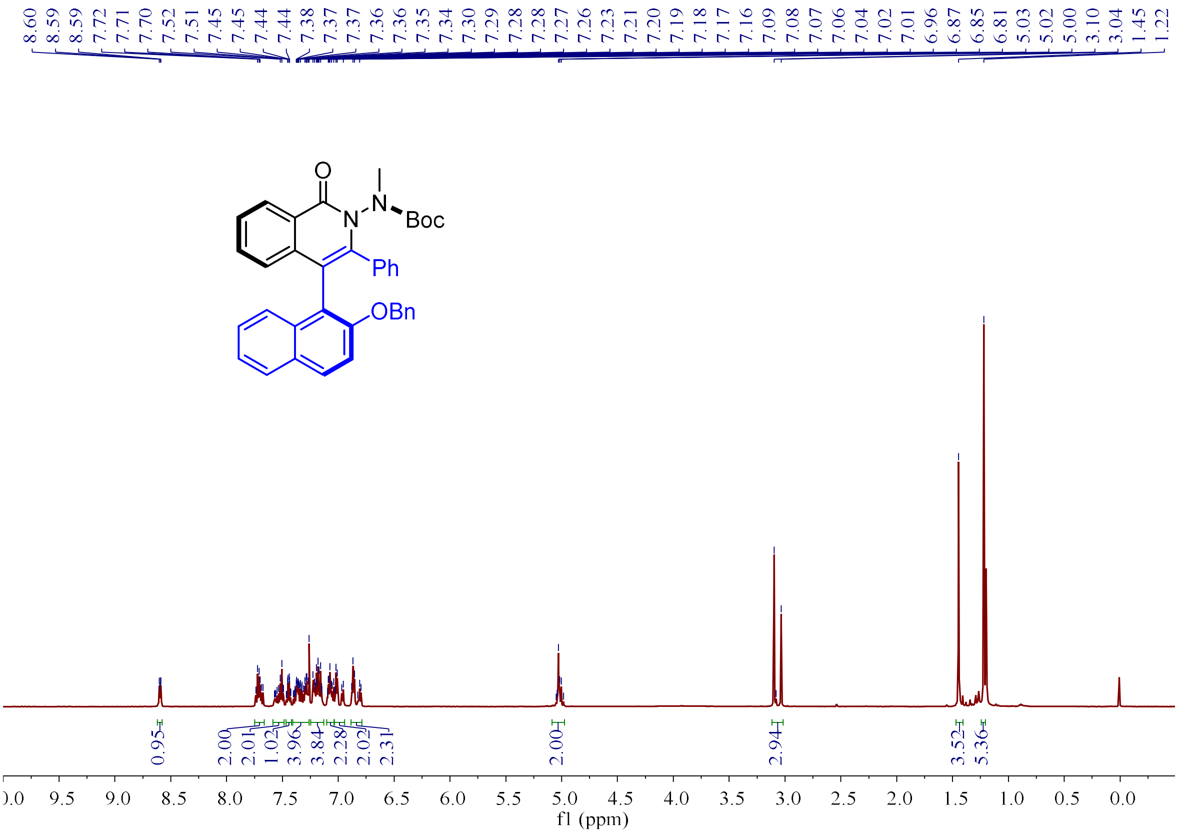


**Supplementary Figure 196.** **$^{1}$H NMR (600 MHz, $CDCl_3$) spectrum of 36.**

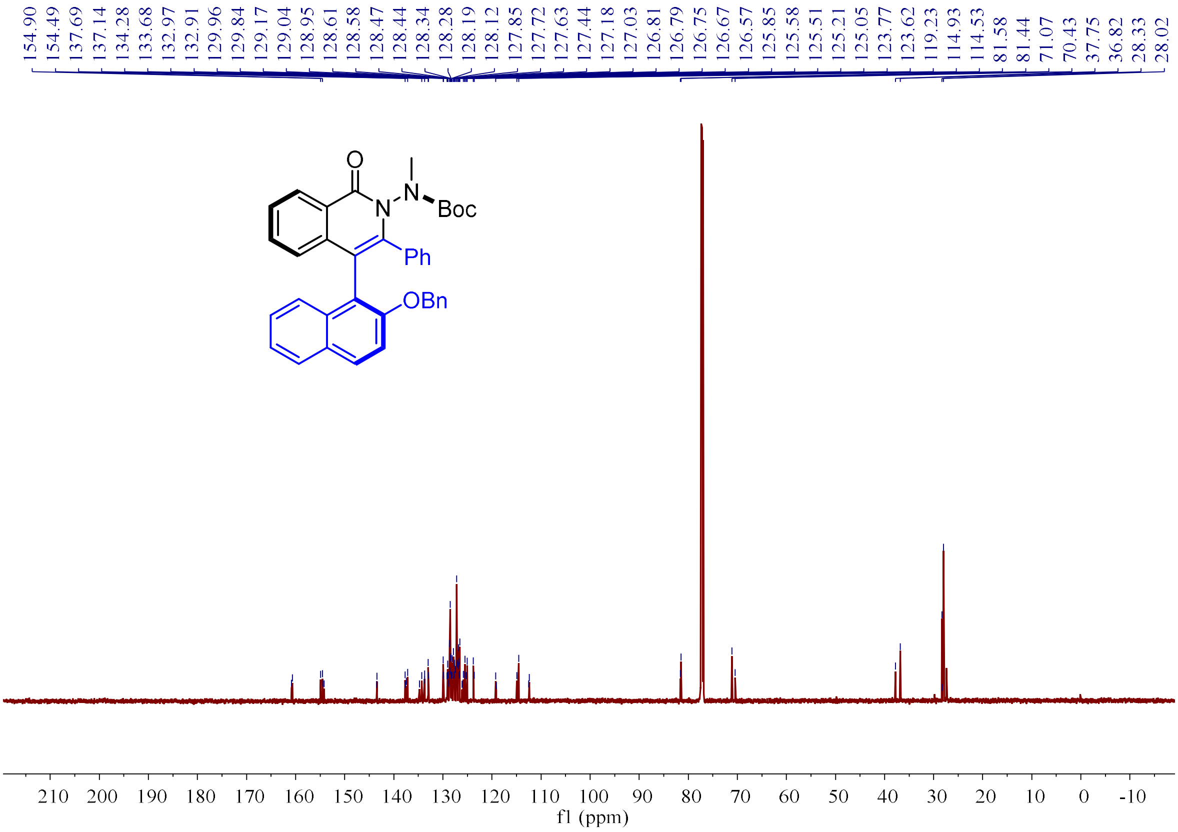


**Supplementary Figure 197.** **$^{13}$C NMR (150 MHz, $CDCl_3$) spectrum of 36.**

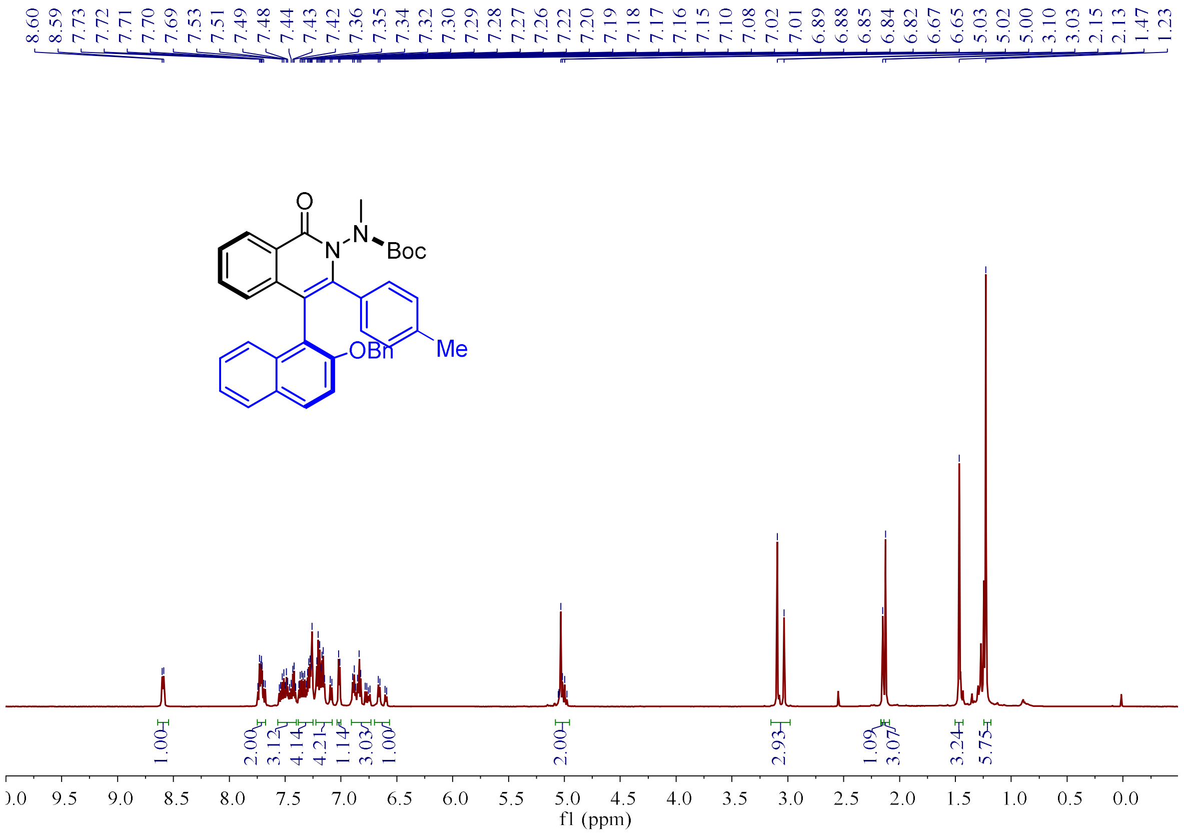

**Supplementary Figure 198. $^{1}$H NMR (600 MHz, CDCl$_3$) spectrum of 37.**

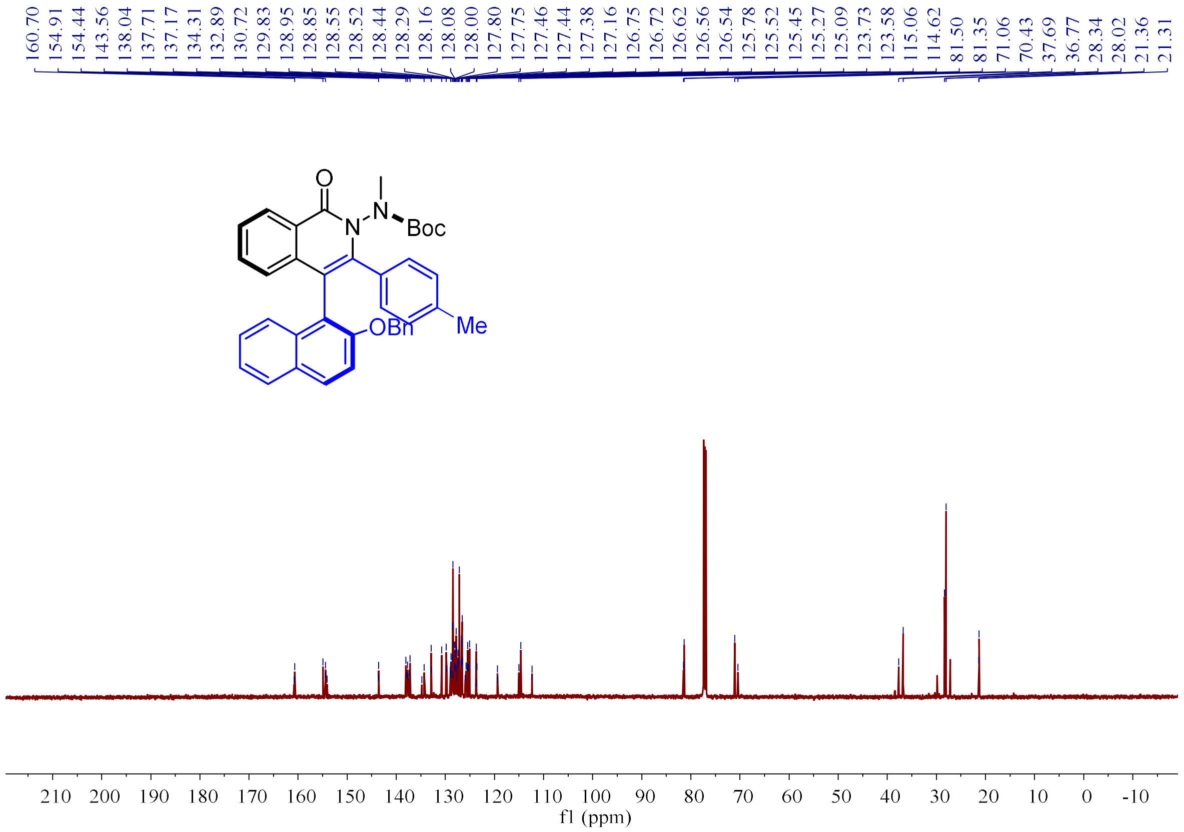

**Supplementary Figure 199. $^{13}$C NMR (150 MHz, CDCl$_3$) spectrum of 37.**

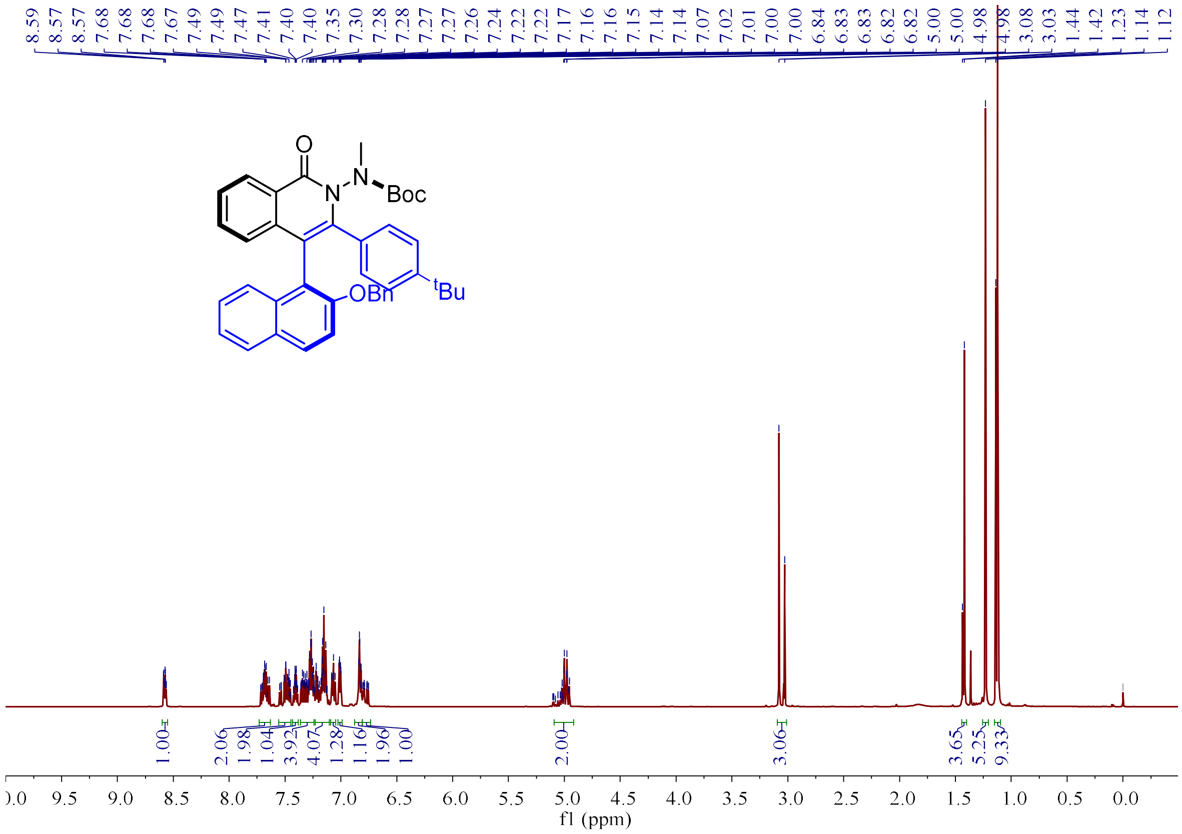


**Supplementary Figure 200.** $^{1}$**H NMR (600 MHz, CDCl$_3$) spectrum of 38.**

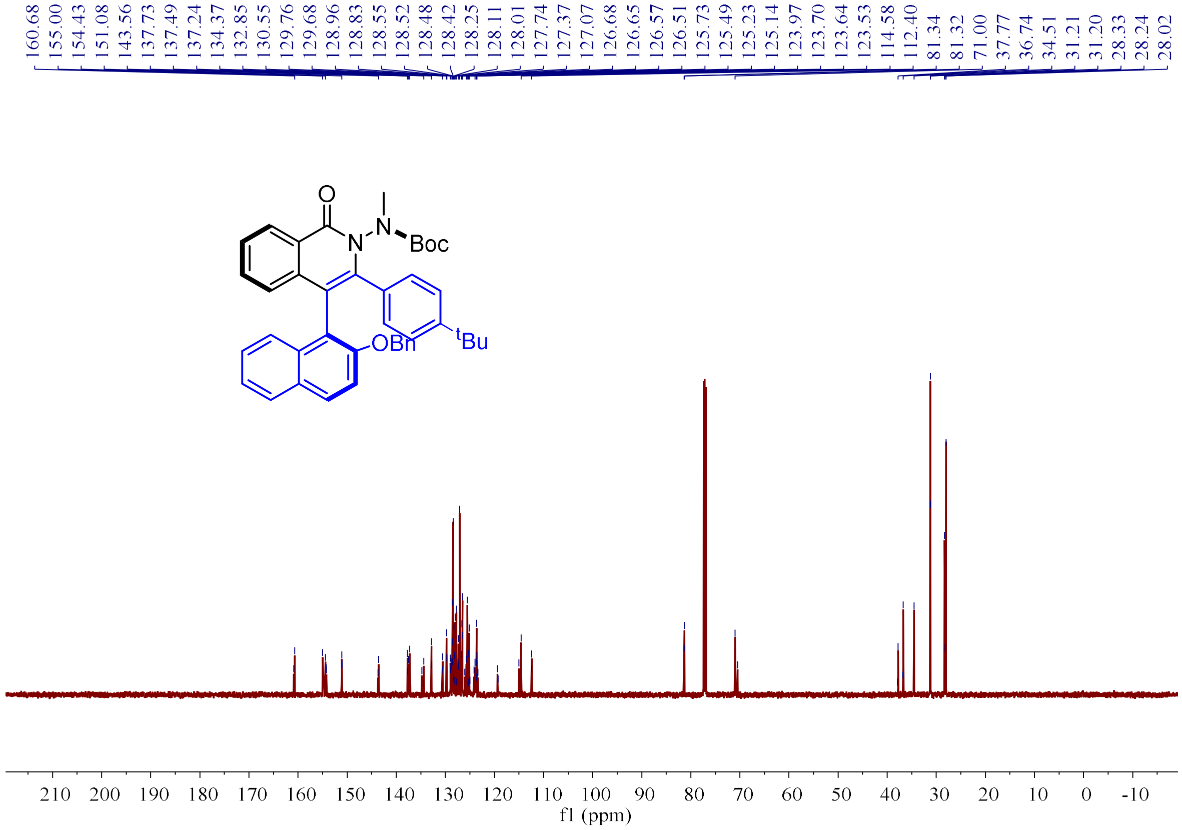


**Supplementary Figure 201.** $^{13}$**C NMR (150 MHz, CDCl$_3$) spectrum of 38.**

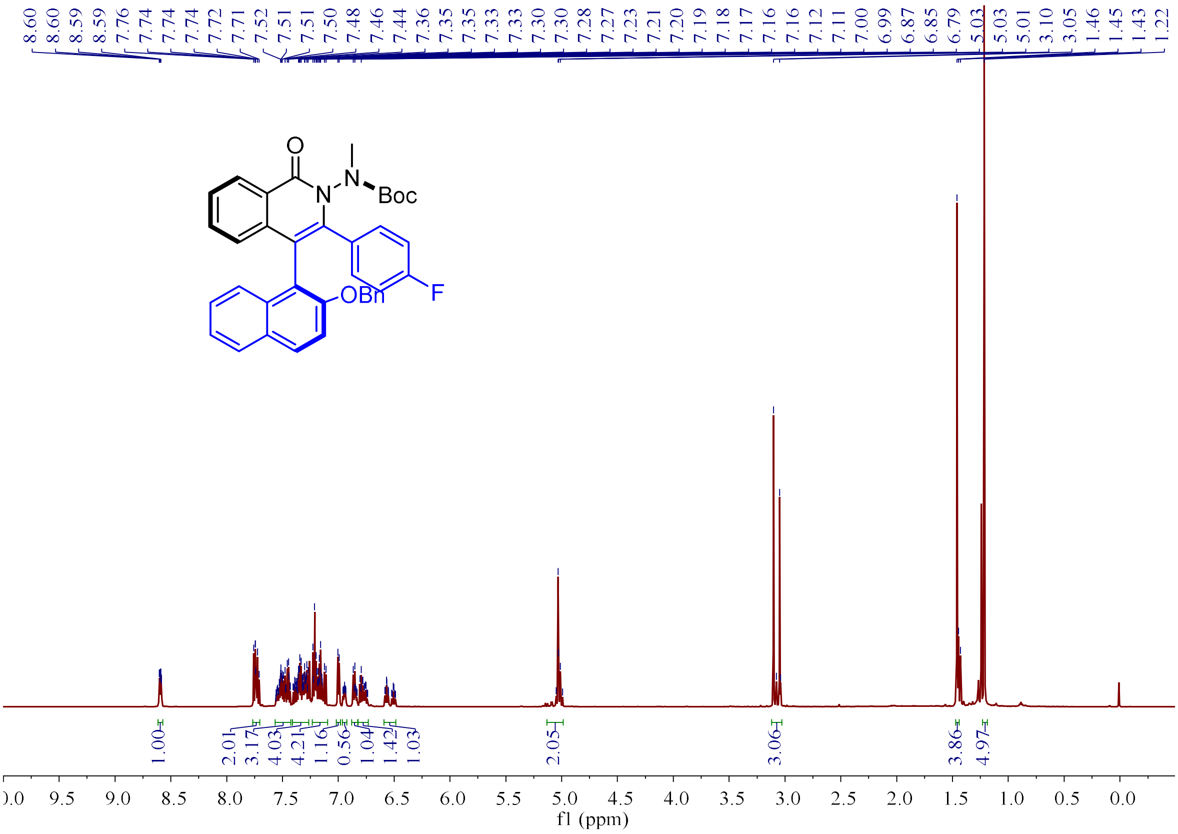


**Supplementary Figure 202. $^{1}$H NMR (600 MHz, $CDCl_3$) spectrum of 39.**

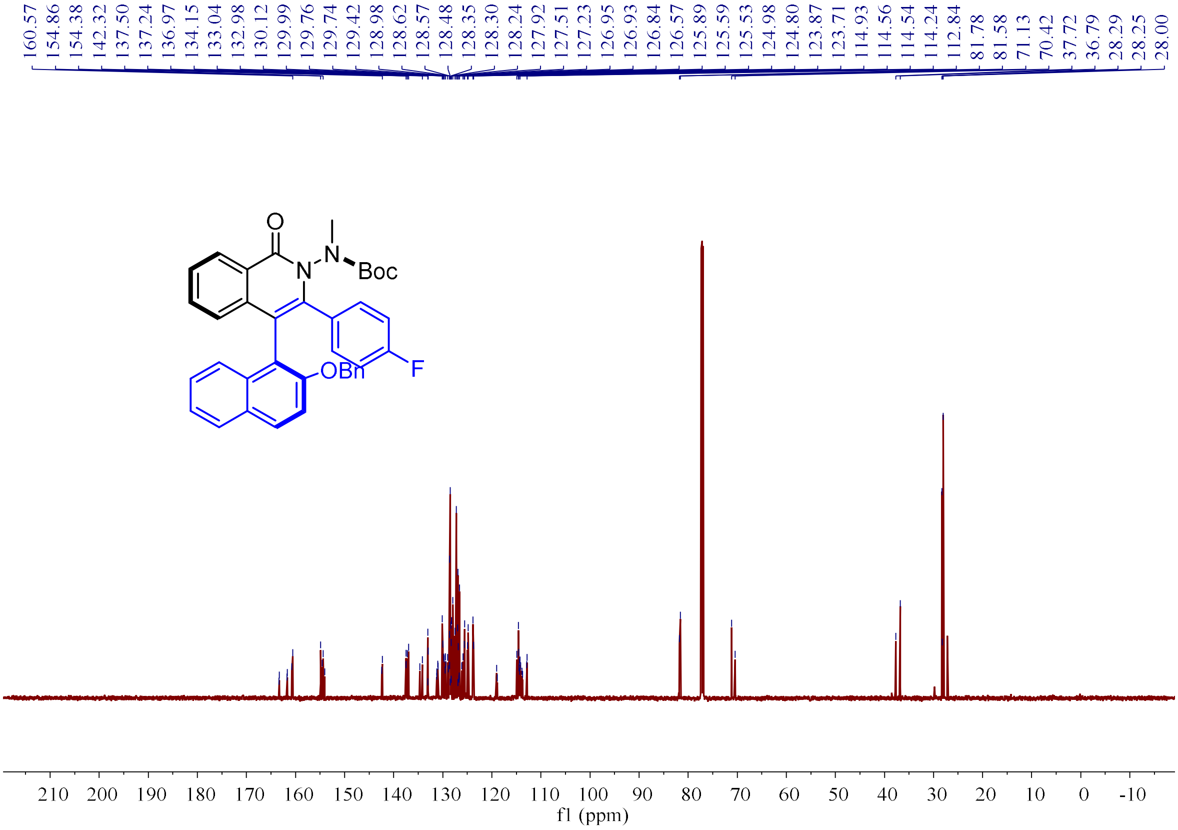


**Supplementary Figure 203. $^{13}$C NMR (150 MHz, $CDCl_3$) spectrum of 39.**

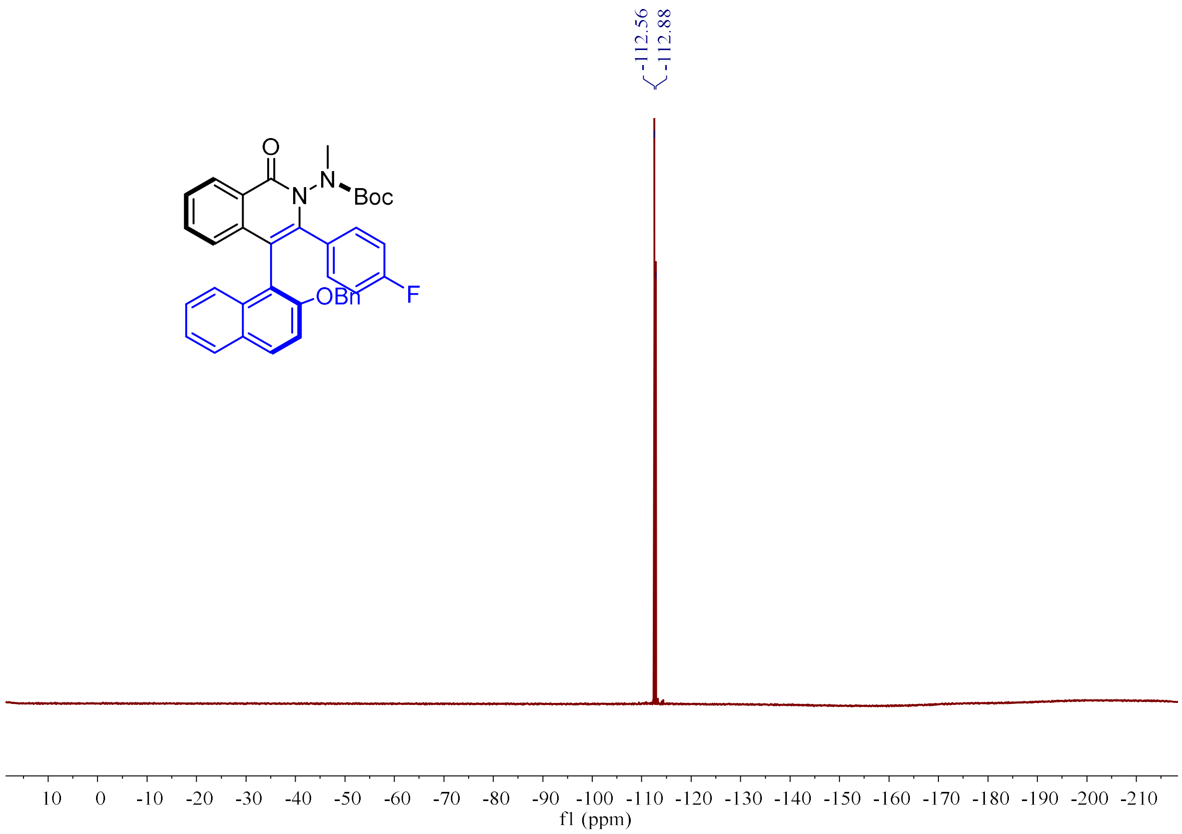


**Supplementary Figure 204.** **$^{19}$F NMR (376 MHz, $CDCl_3$) spectrum of 39.**

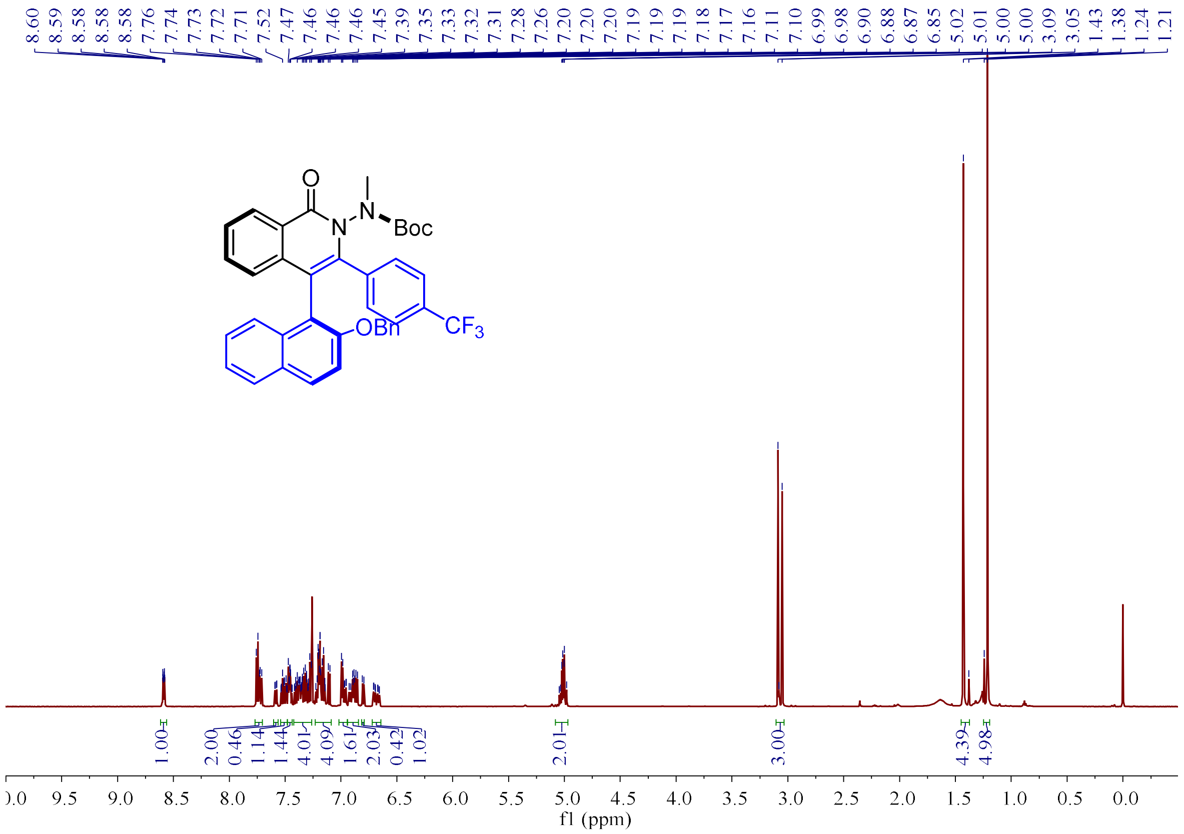

**Supplementary Figure 205. $^{1}$H NMR (600 MHz, CDCl₃) spectrum of 40.**

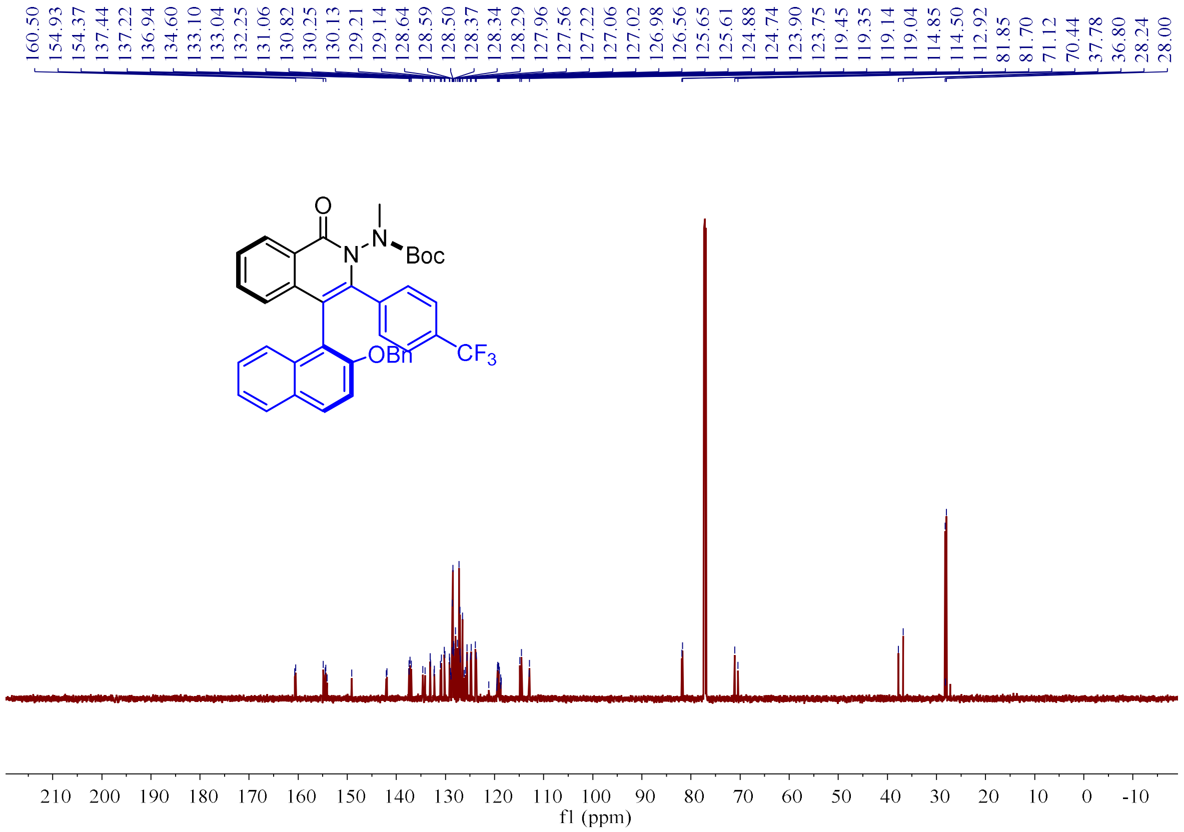

**Supplementary Figure 206. $^{13}$C NMR (150 MHz, CDCl₃) spectrum of 40.**

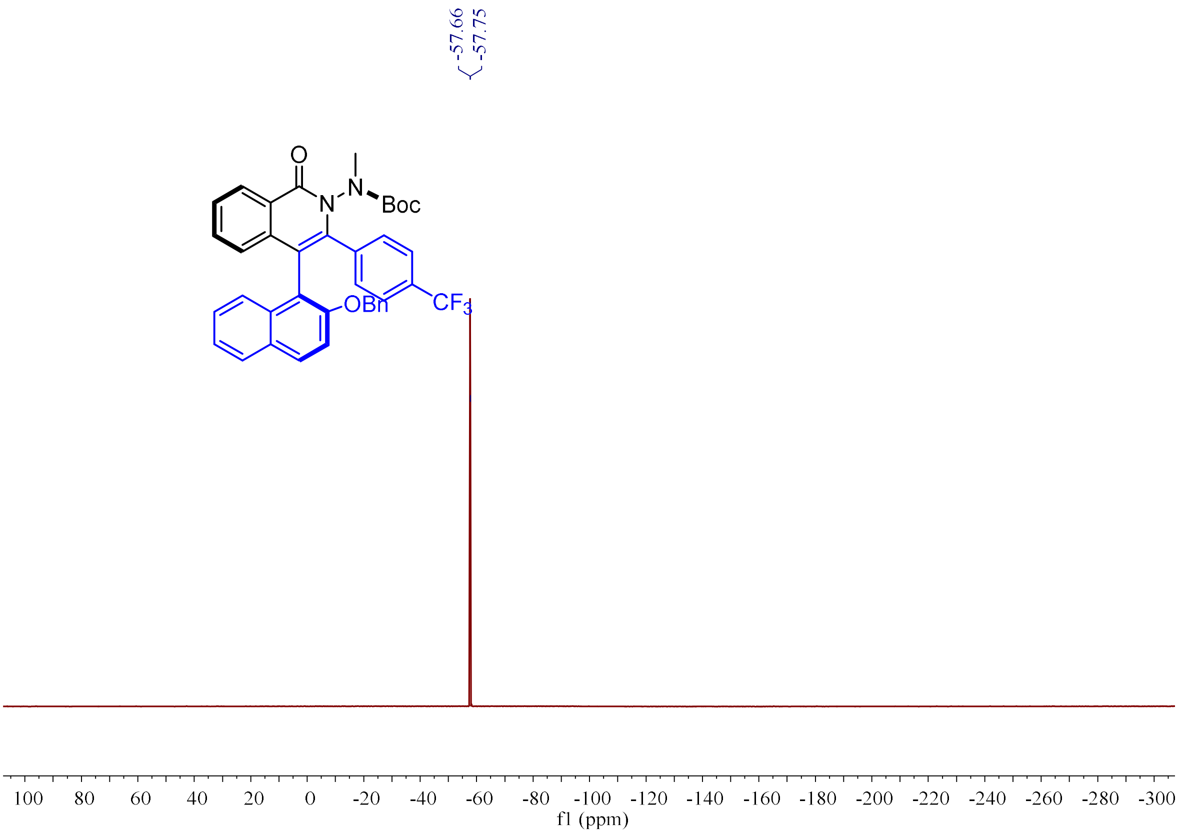


**Supplementary Figure 207.** $^{19}$**F NMR (376 MHz, CDCl**$_3$**) spectrum of 40.**

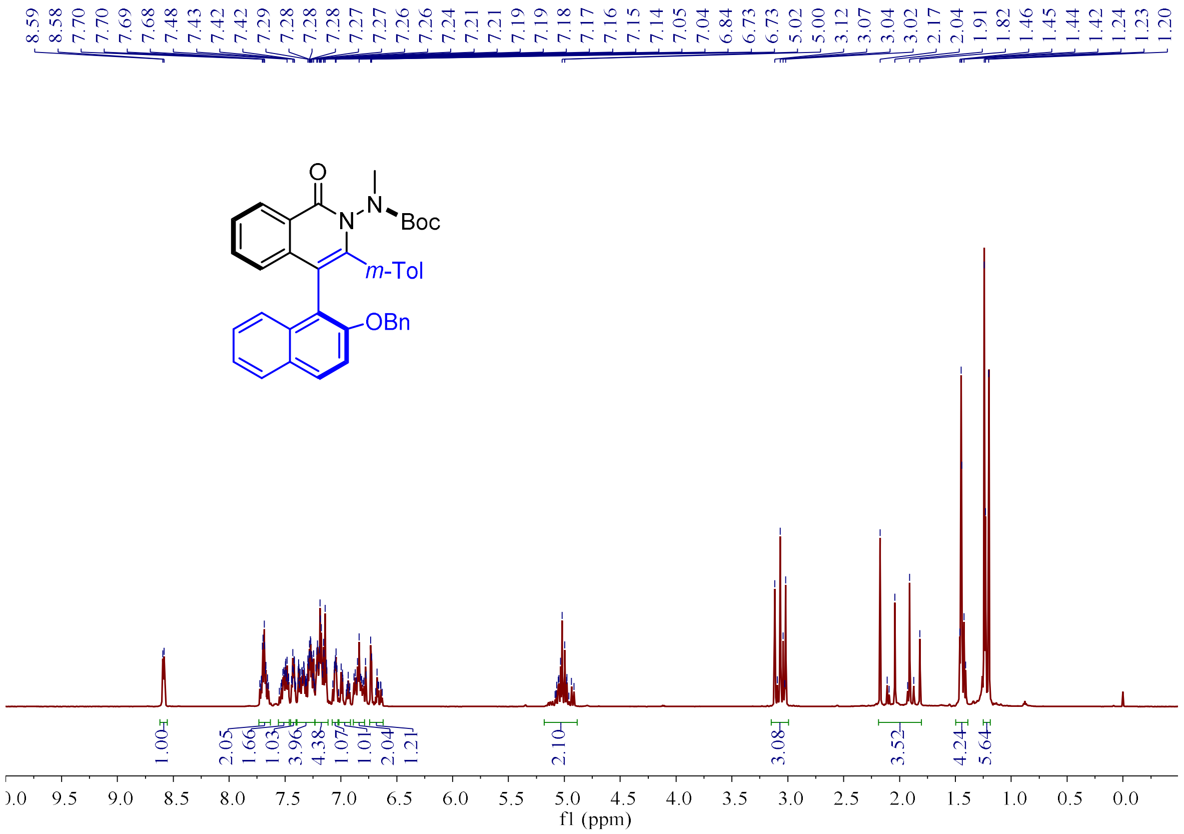


**Supplementary Figure 208. $^{1}$H NMR (600 MHz, $CDCl_3$) spectrum of 41.**

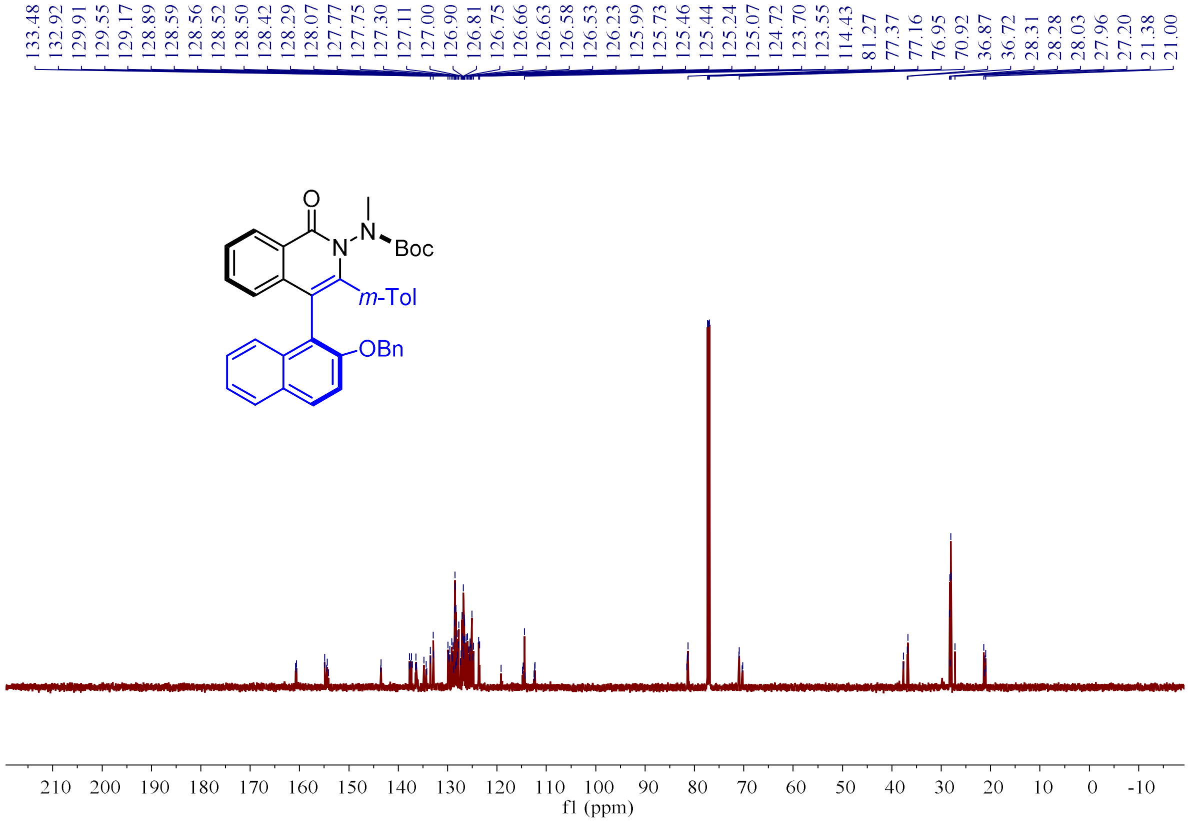


**Supplementary Figure 209. $^{13}$C NMR (150 MHz, $CDCl_3$) spectrum of 41.**

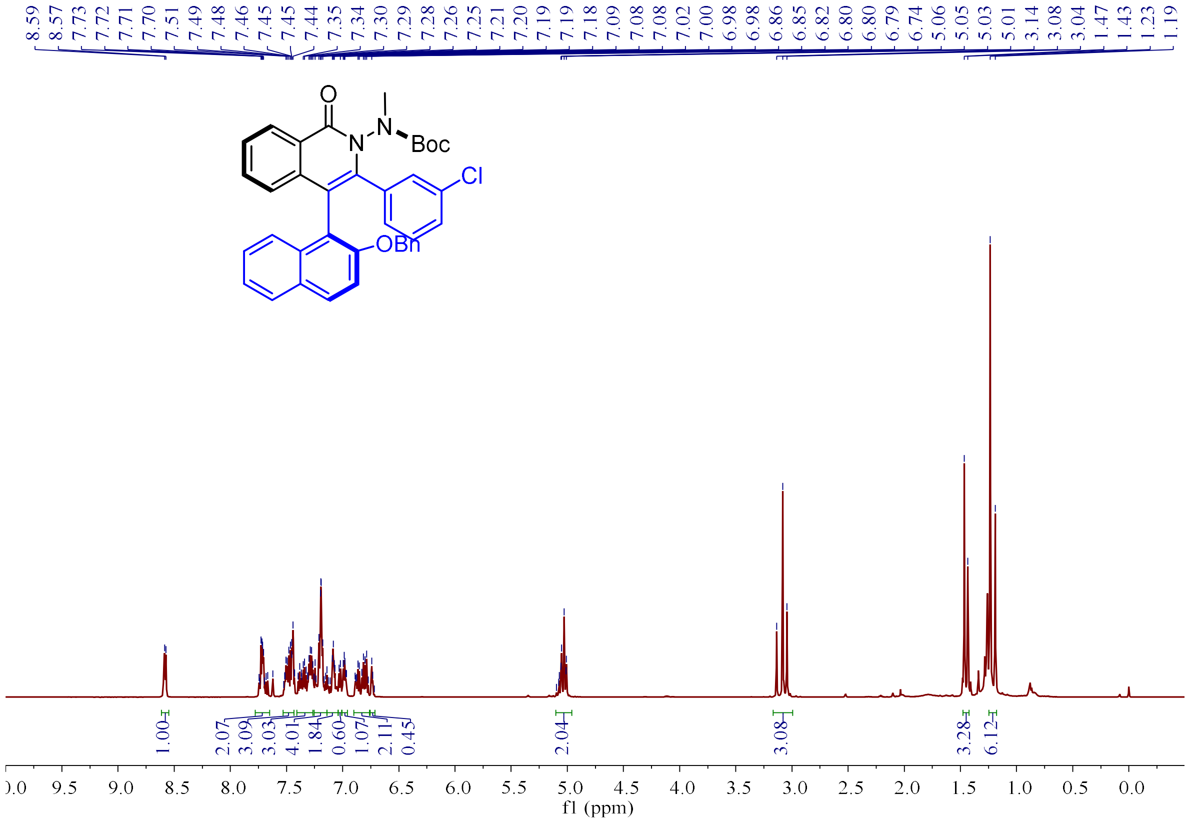

**Supplementary Figure 210.** $^{1}$**H NMR (600 MHz, CDCl$_3$) spectrum of 42.**

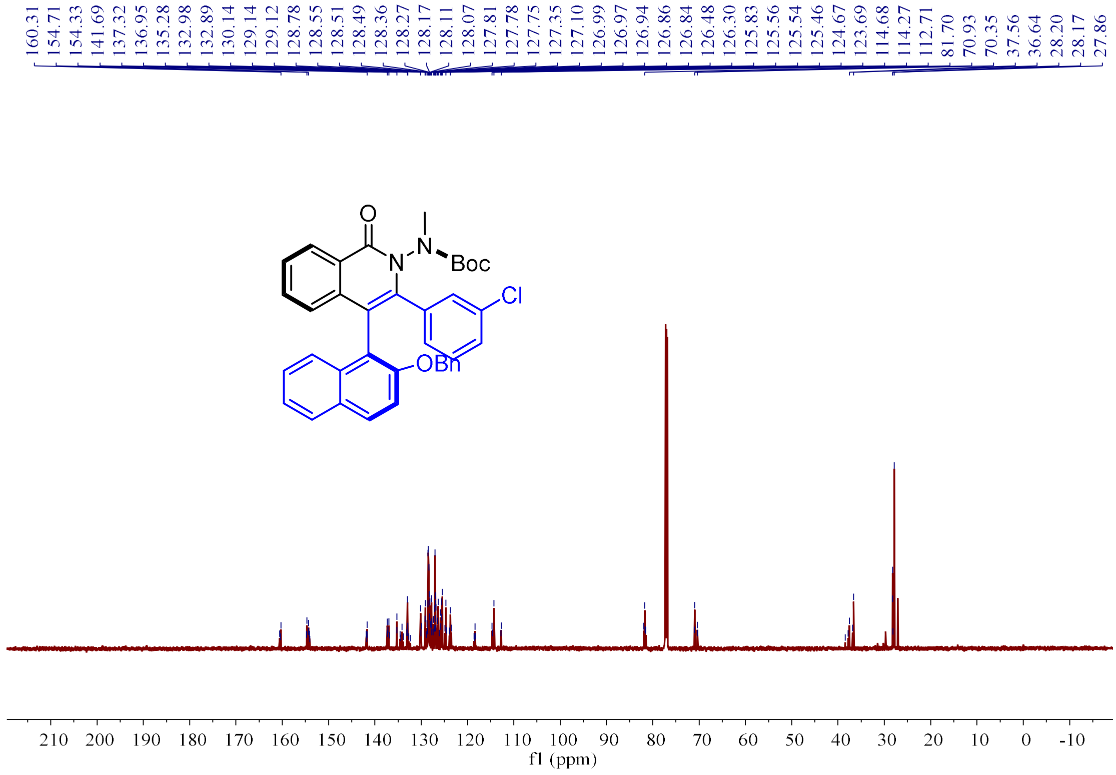

**Supplementary Figure 211.** $^{13}$**C NMR (150 MHz, CDCl$_3$) spectrum of 42.**

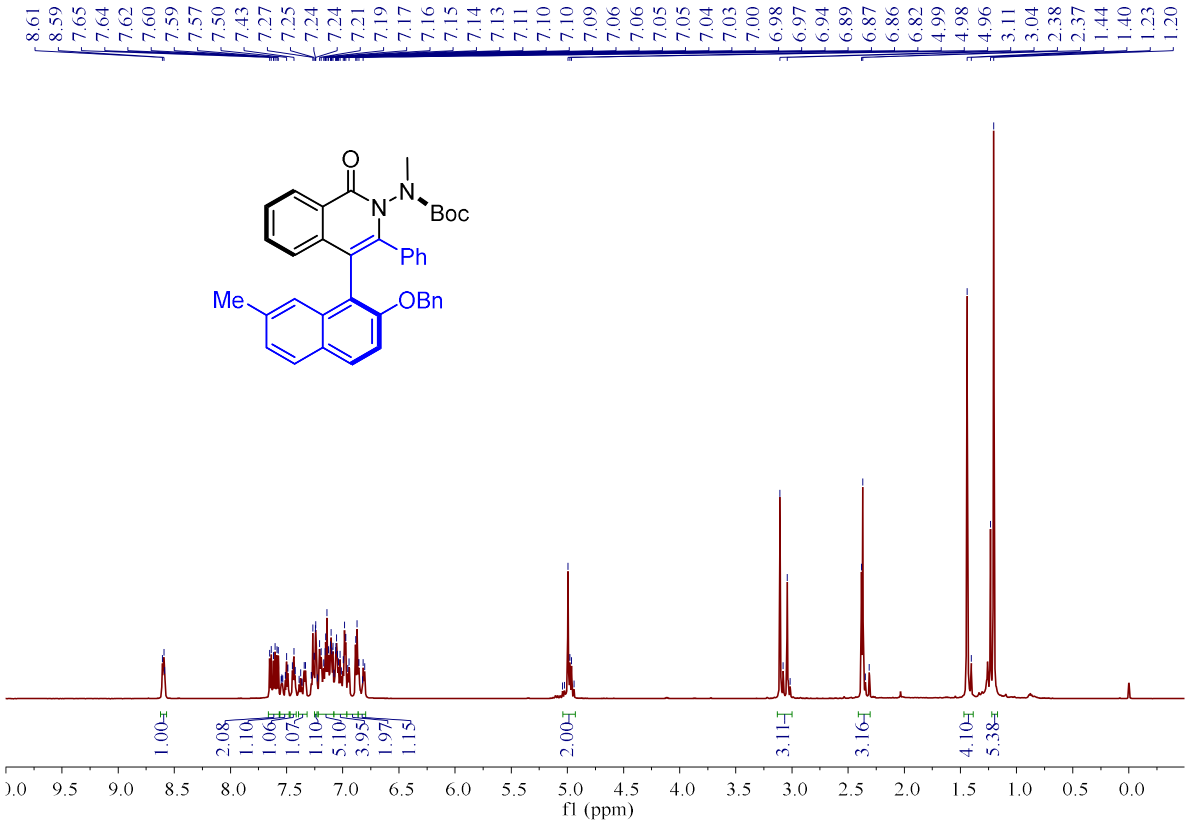


**Supplementary Figure 212.** **$^{1}$H NMR (600 MHz, $CDCl_3$) spectrum of 43.**

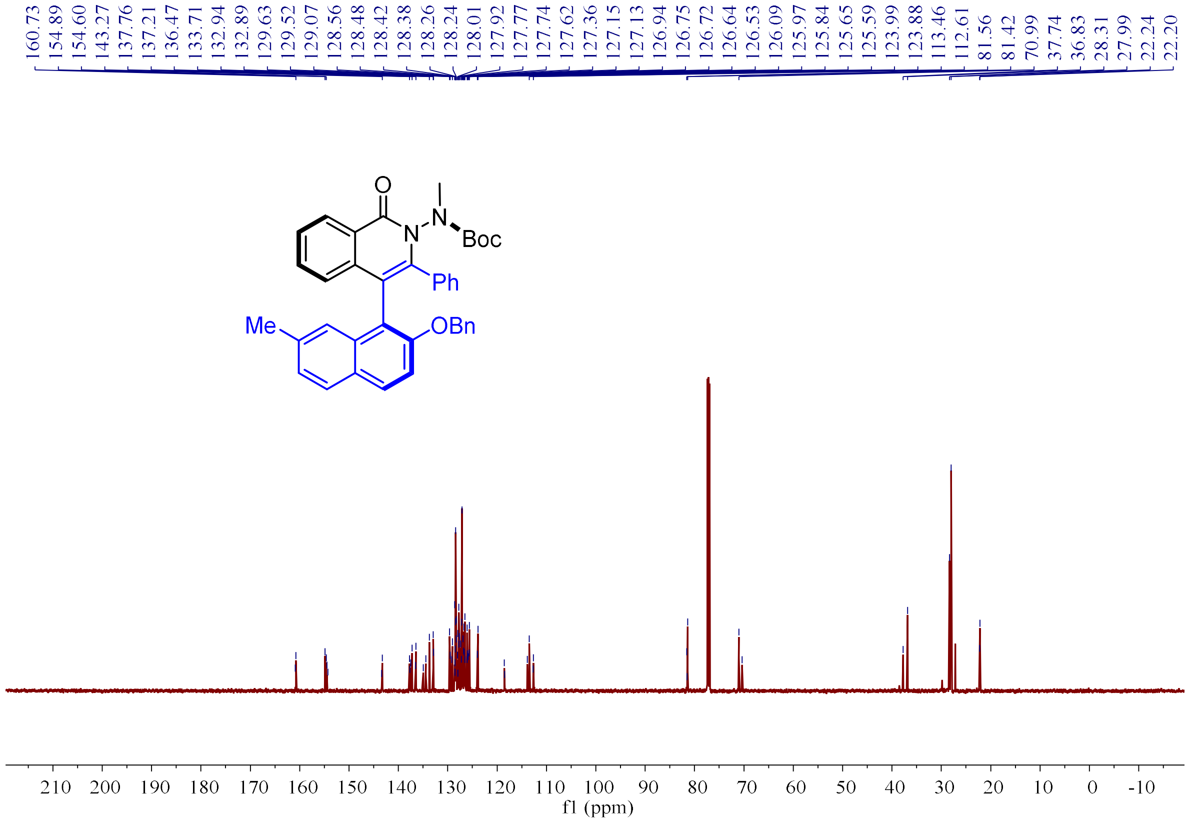


**Supplementary Figure 213.** **$^{13}$C NMR (150 MHz, $CDCl_3$) spectrum of 43.**

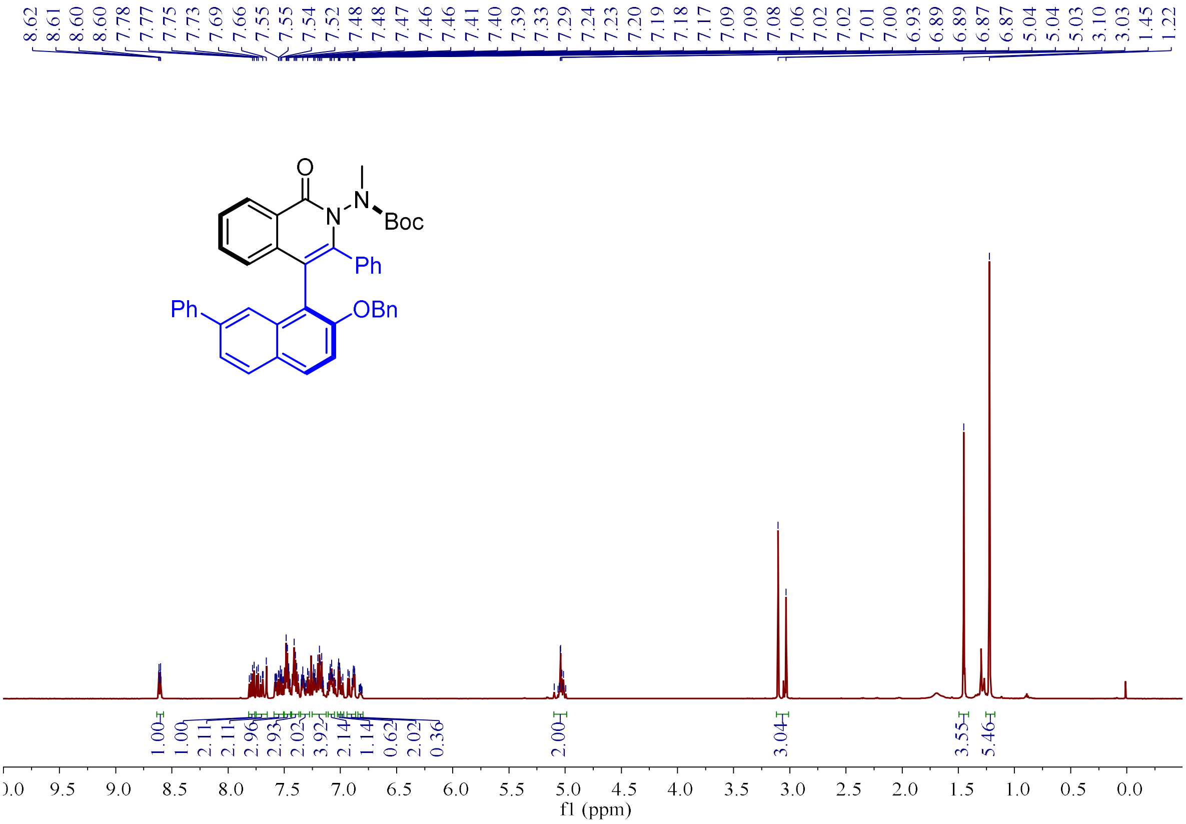

**Supplementary Figure 214. $^{1}$H NMR (600 MHz, $CDCl_3$) spectrum of 44.**

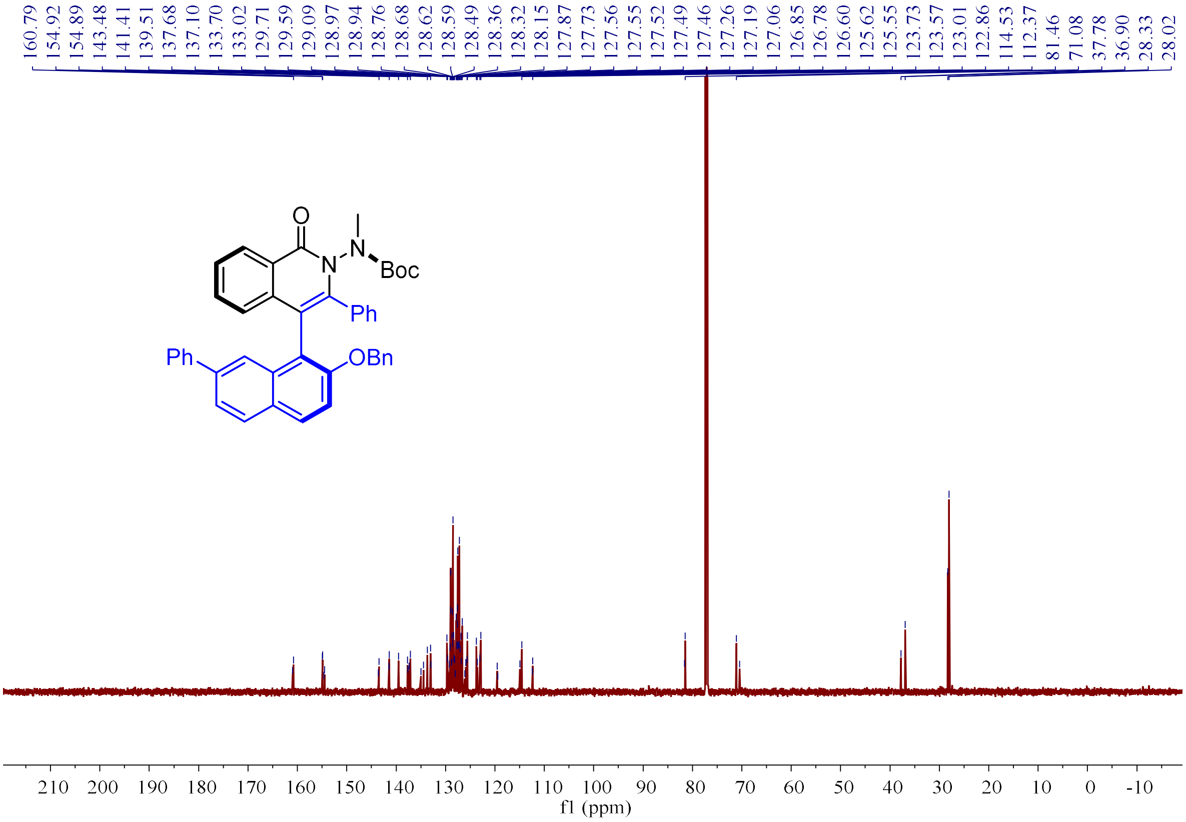

**Supplementary Figure 215. $^{13}$C NMR (150 MHz, $CDCl_3$) spectrum of 44.**

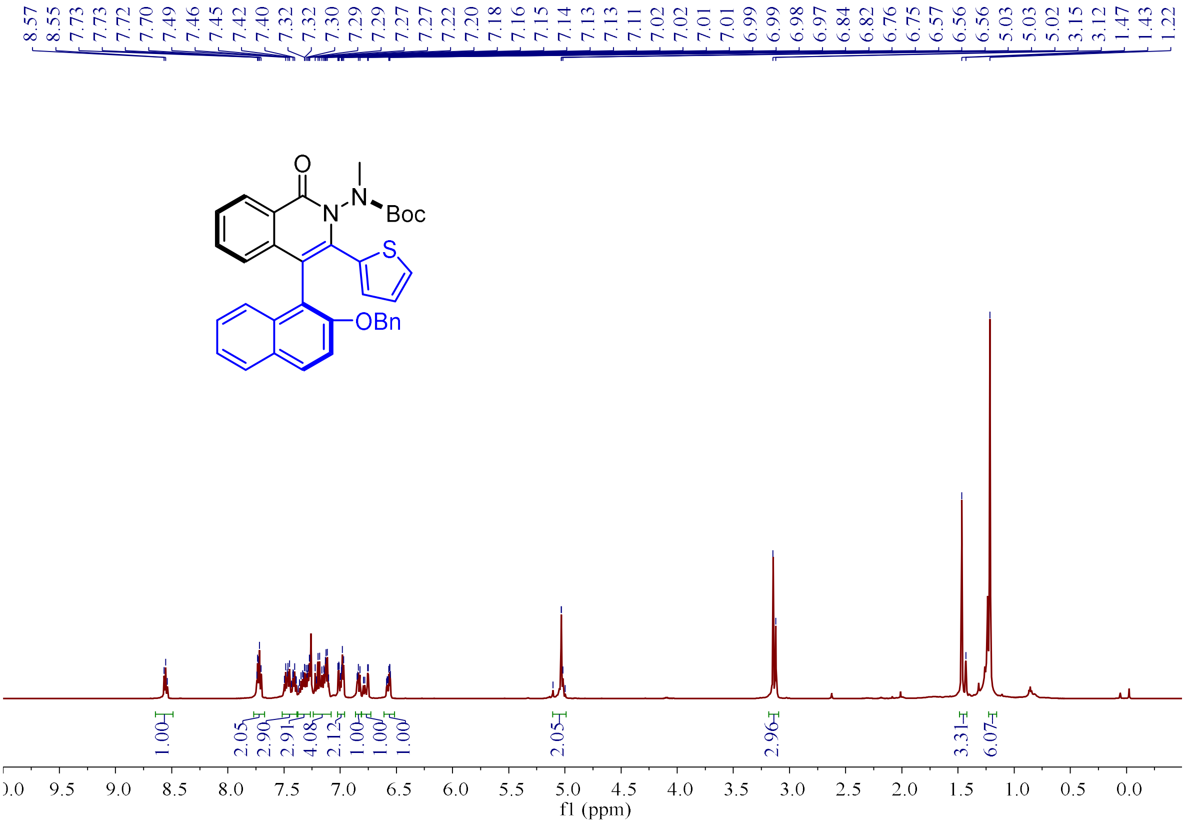


**Supplementary Figure 216. $^{1}$H NMR (600 MHz, CDCl$_3$) spectrum of 45.**

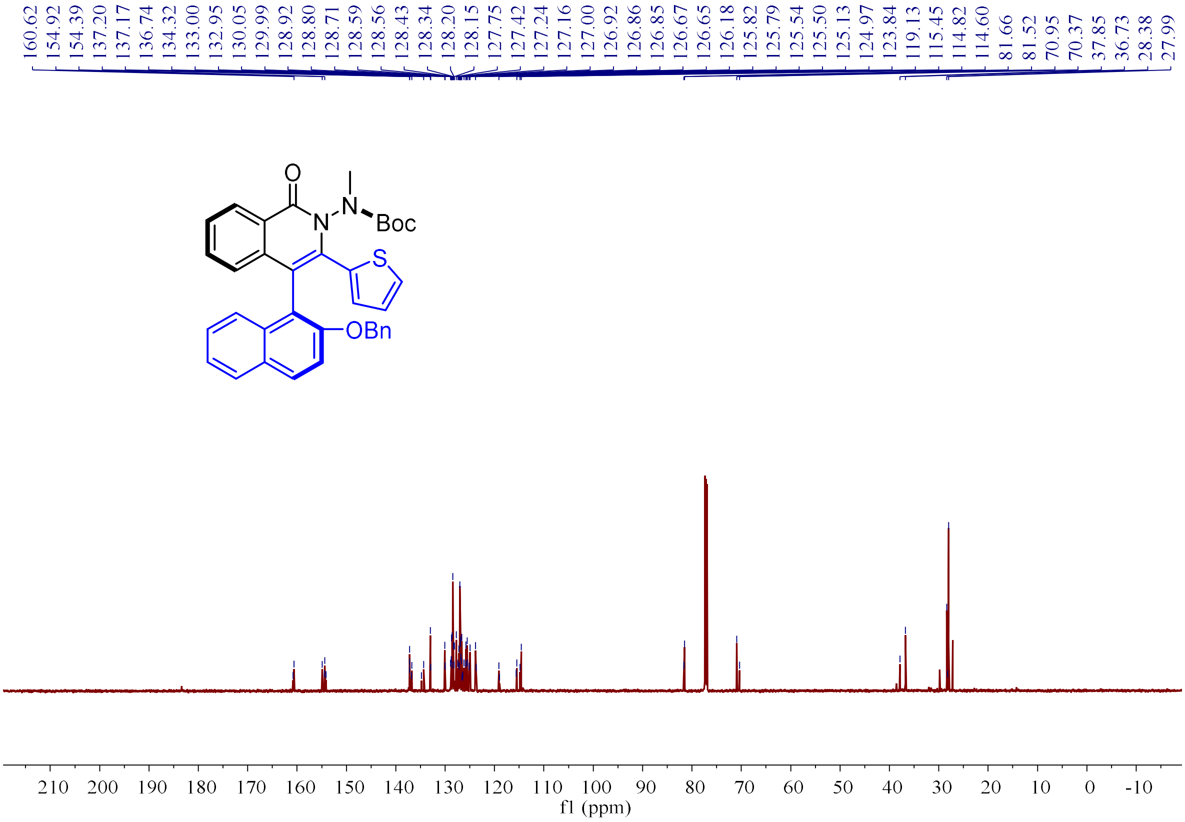


**Supplementary Figure 217. $^{13}$C NMR (150 MHz, CDCl$_3$) spectrum of 45.**

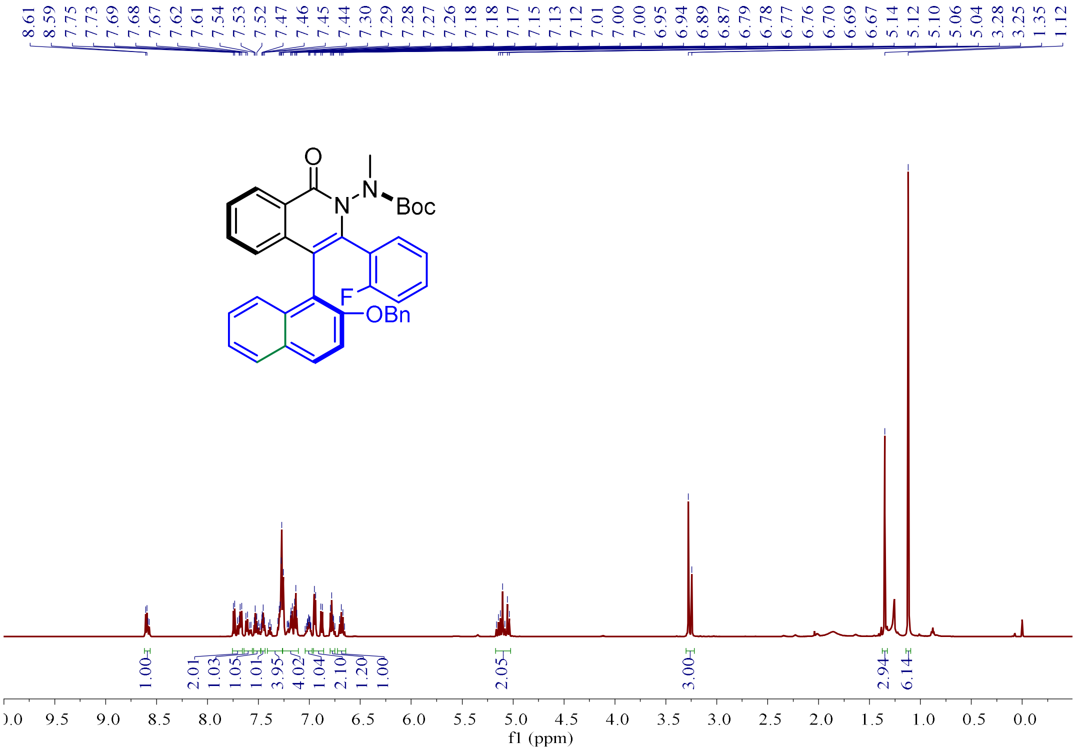


**Supplementary Figure 218. $^{1}H$ NMR (600 MHz, $CDCl_3$) spectrum of 46.**

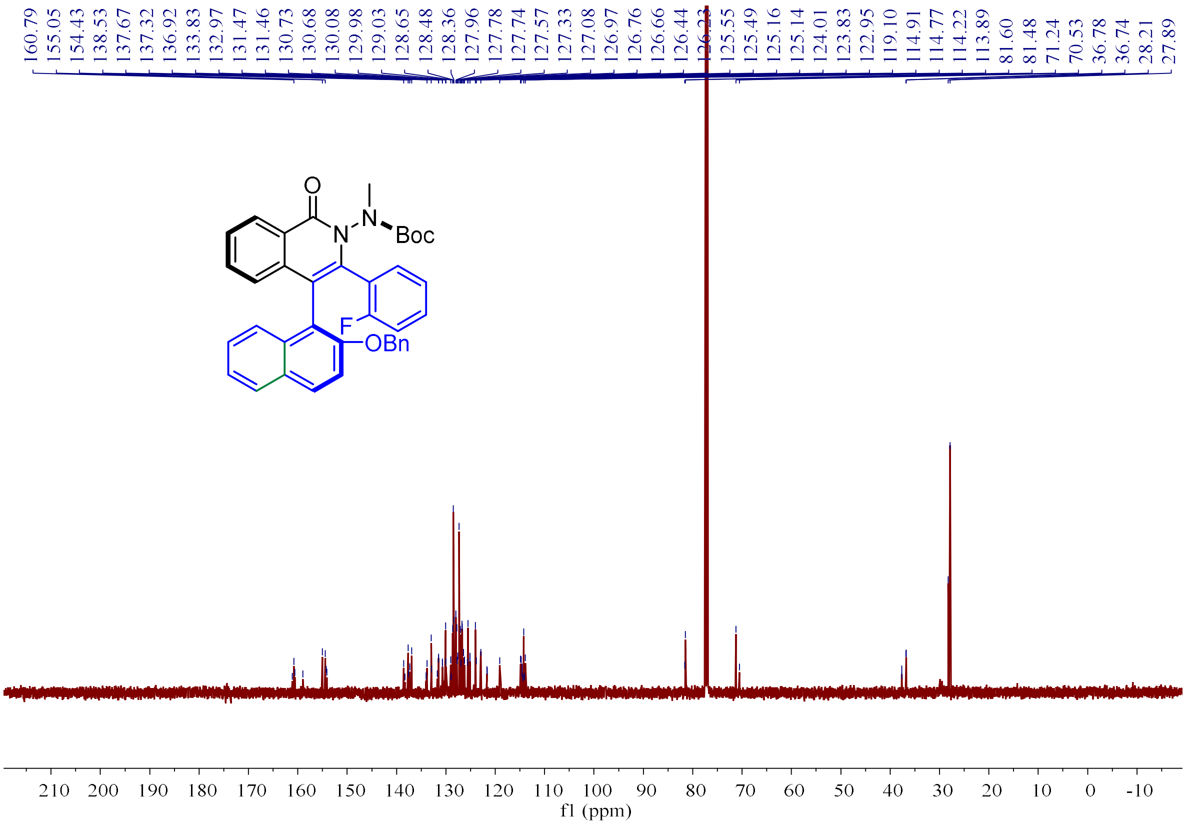


**Supplementary Figure 219. $^{13}C$ NMR (150 MHz, $CDCl_3$) spectrum of 46.**

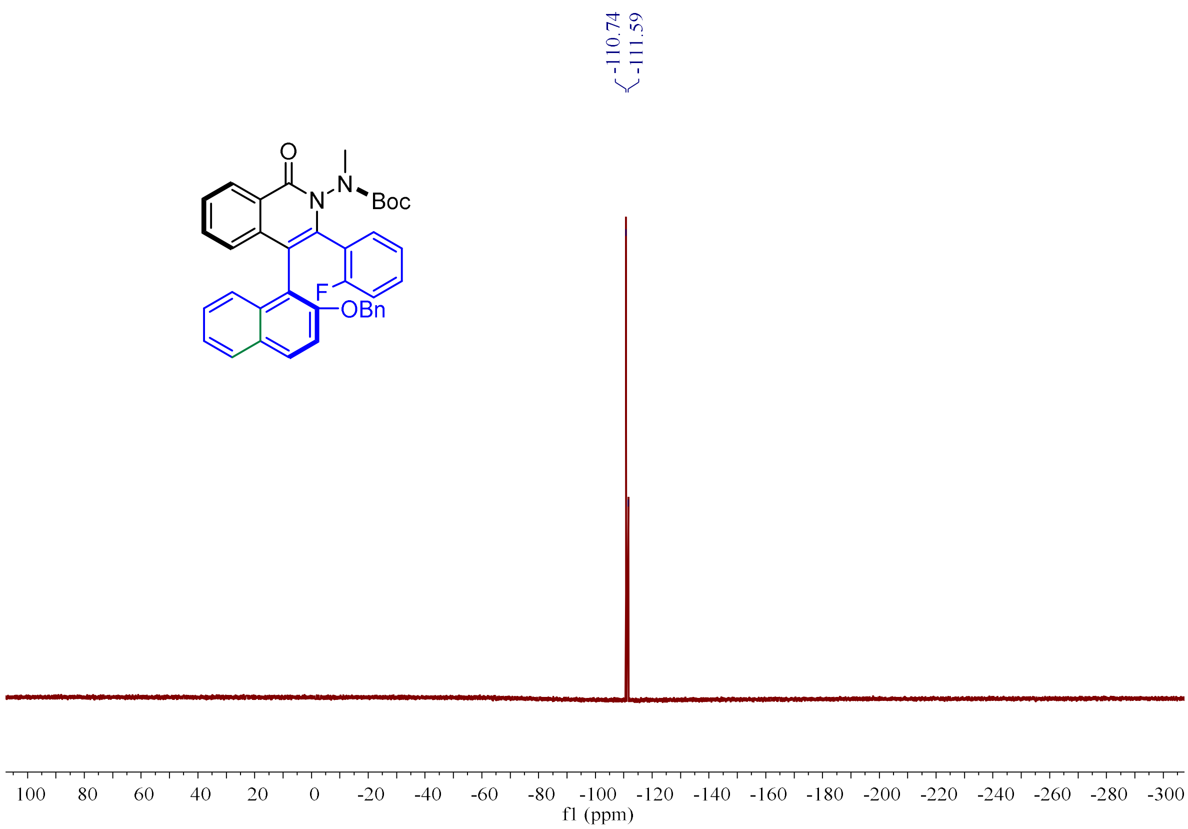


**Supplementary Figure 220.** $^{19}$**F NMR (376 MHz, CDCl**$_3$**) spectrum of 46.**

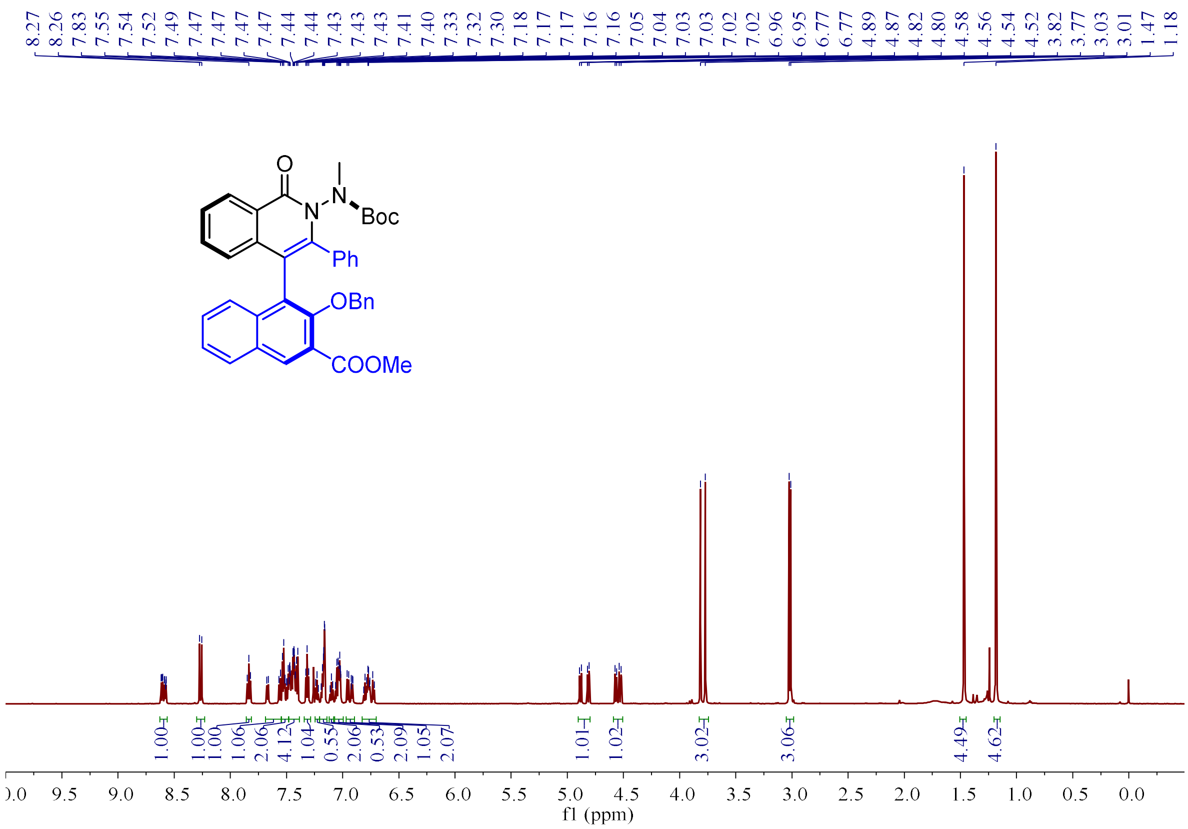

**Supplementary Figure 221. $^{1}$H NMR (600 MHz, $CDCl_3$) spectrum of 47.**

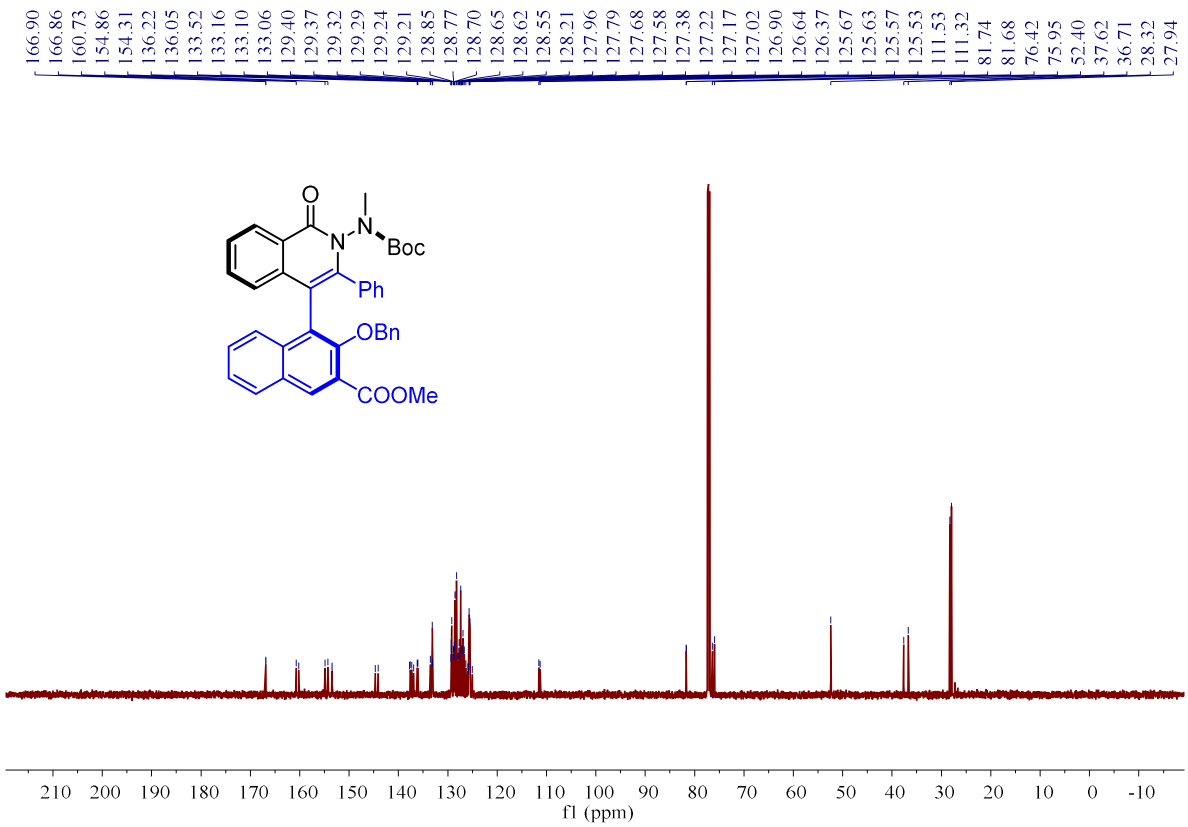

**Supplementary Figure 222. $^{13}$C NMR (150 MHz, $CDCl_3$) spectrum of 47.**

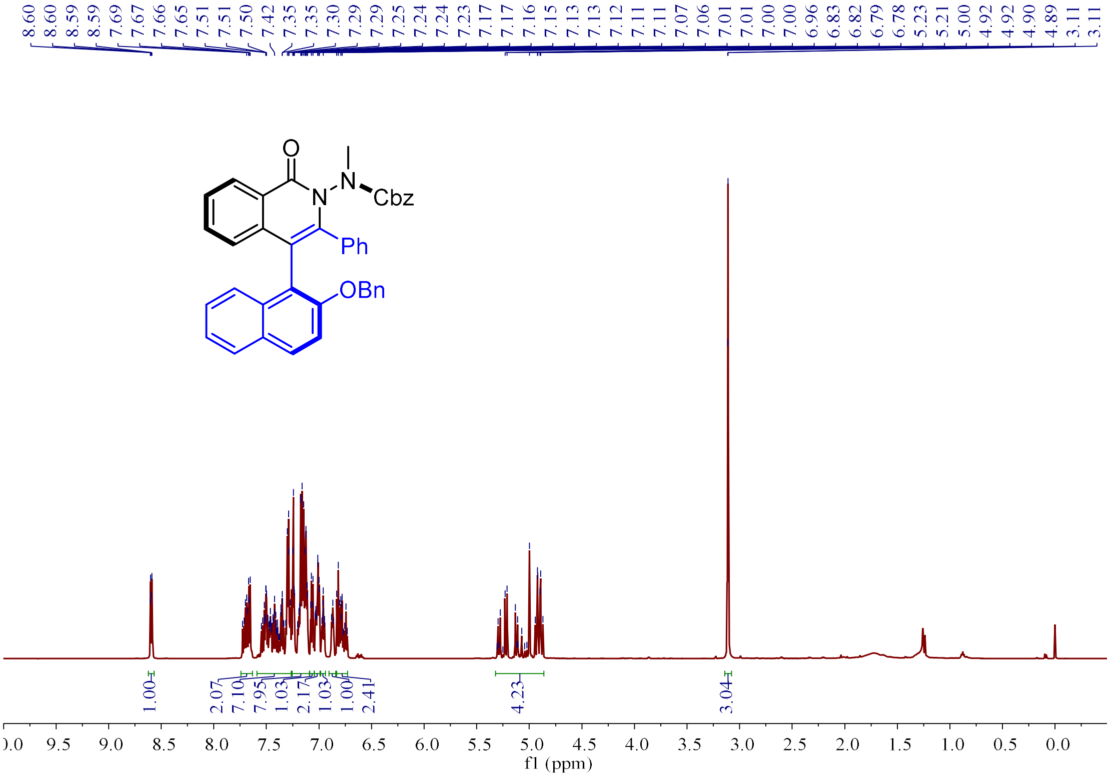

**Supplementary Figure 223. $^{1}$H NMR (600 MHz, CDCl$_3$) spectrum of 48.**

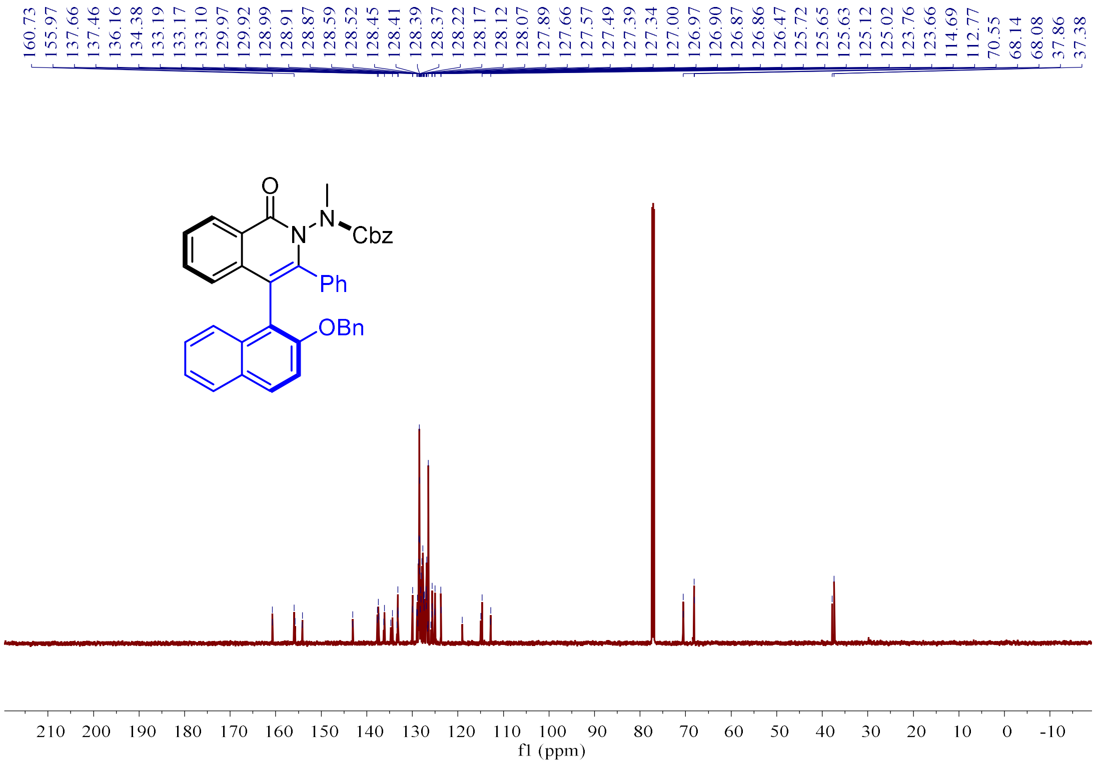

**Supplementary Figure 224. $^{13}$C NMR (150 MHz, CDCl$_3$) spectrum of 48.**

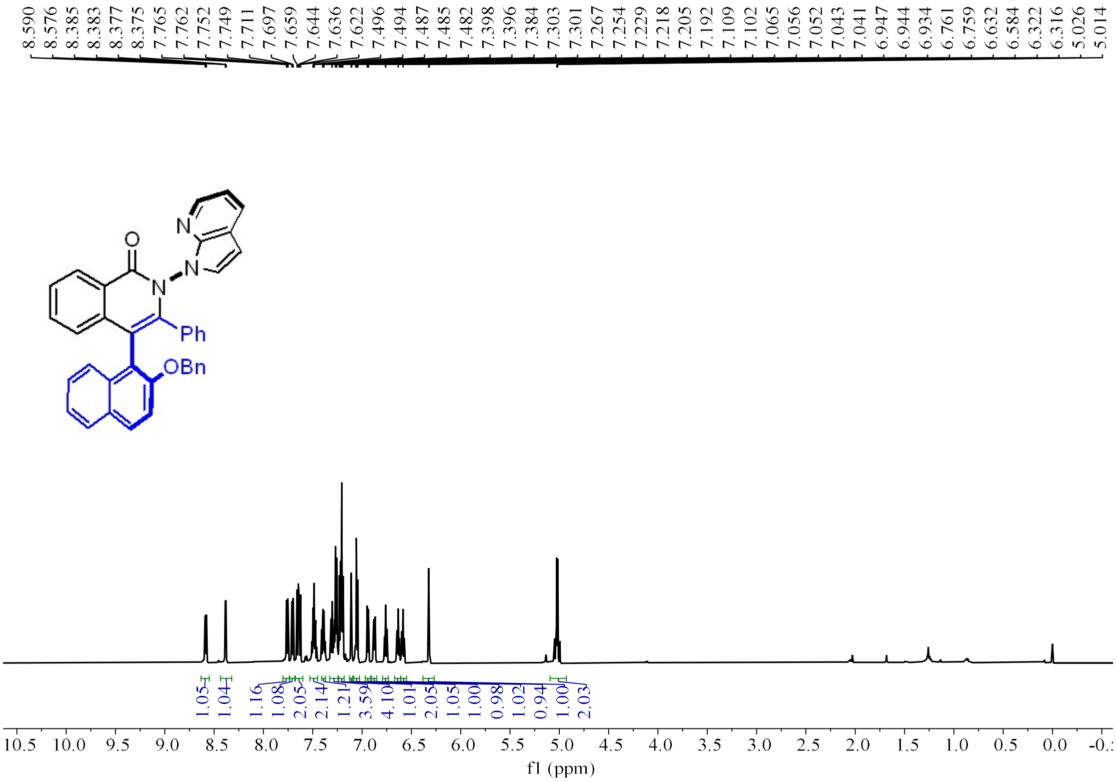


**Supplementary Figure 225.** **$^{1}$H NMR (600 MHz, $CDCl_3$) spectrum of 49.**

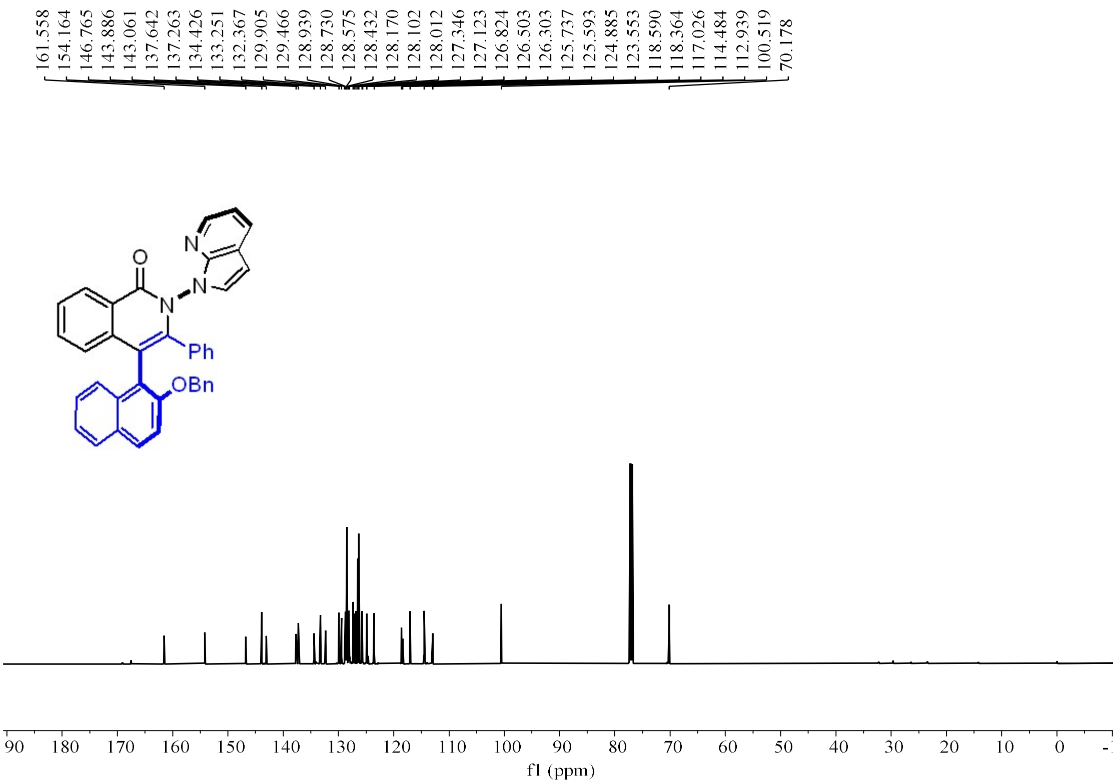


**Supplementary Figure 226.** **$^{13}$C NMR (150 MHz, $CDCl_3$) spectrum of 49.**

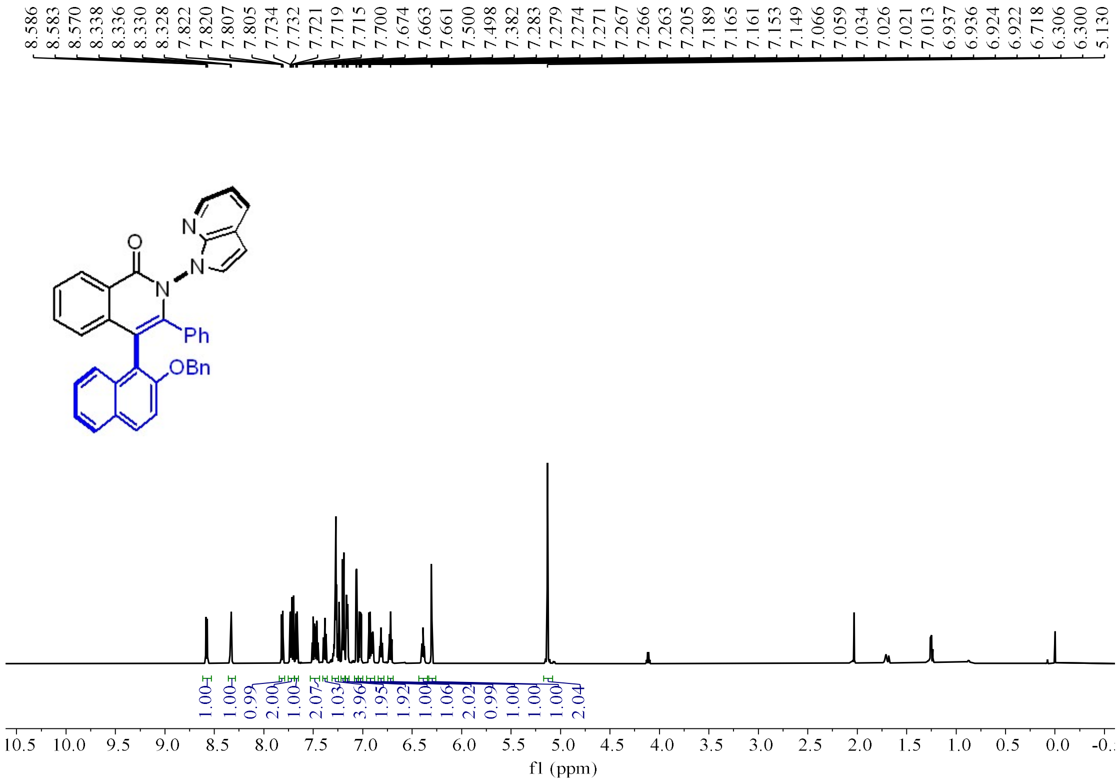


**Supplementary Figure 227. $^{1}$H NMR (600 MHz, $CDCl_3$) spectrum of dia-49.**

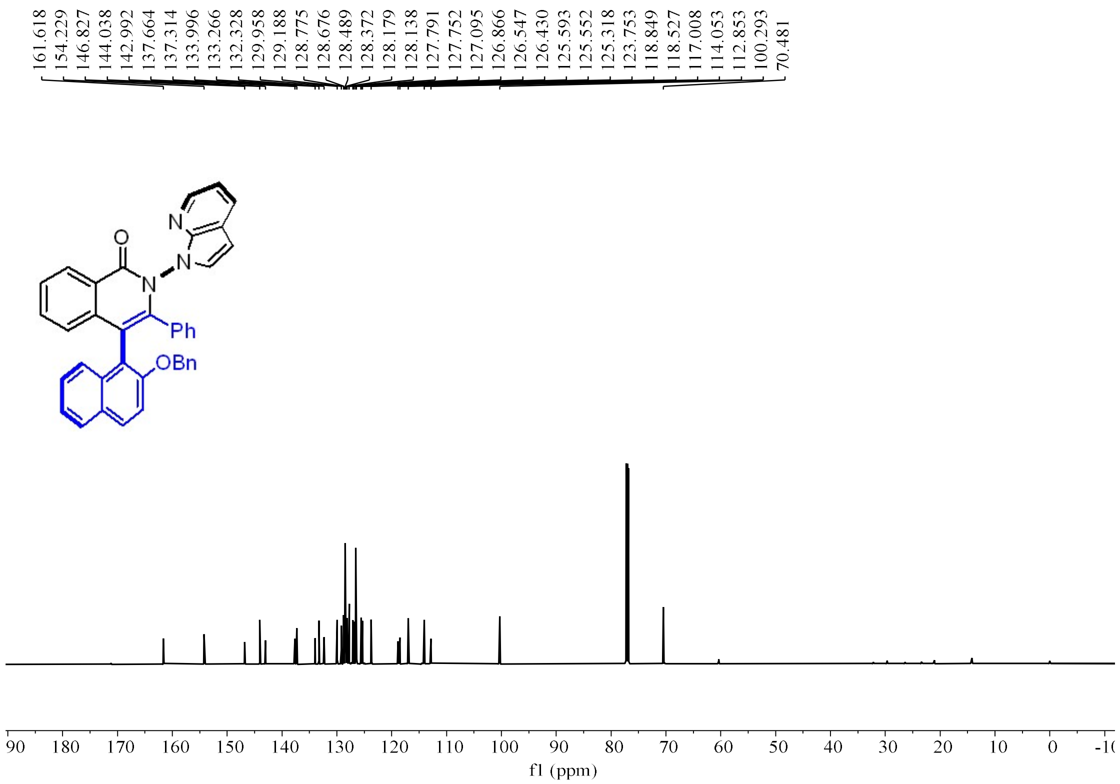


**Supplementary Figure 228. $^{13}$C NMR (150 MHz, $CDCl_3$) spectrum of dia-49.**

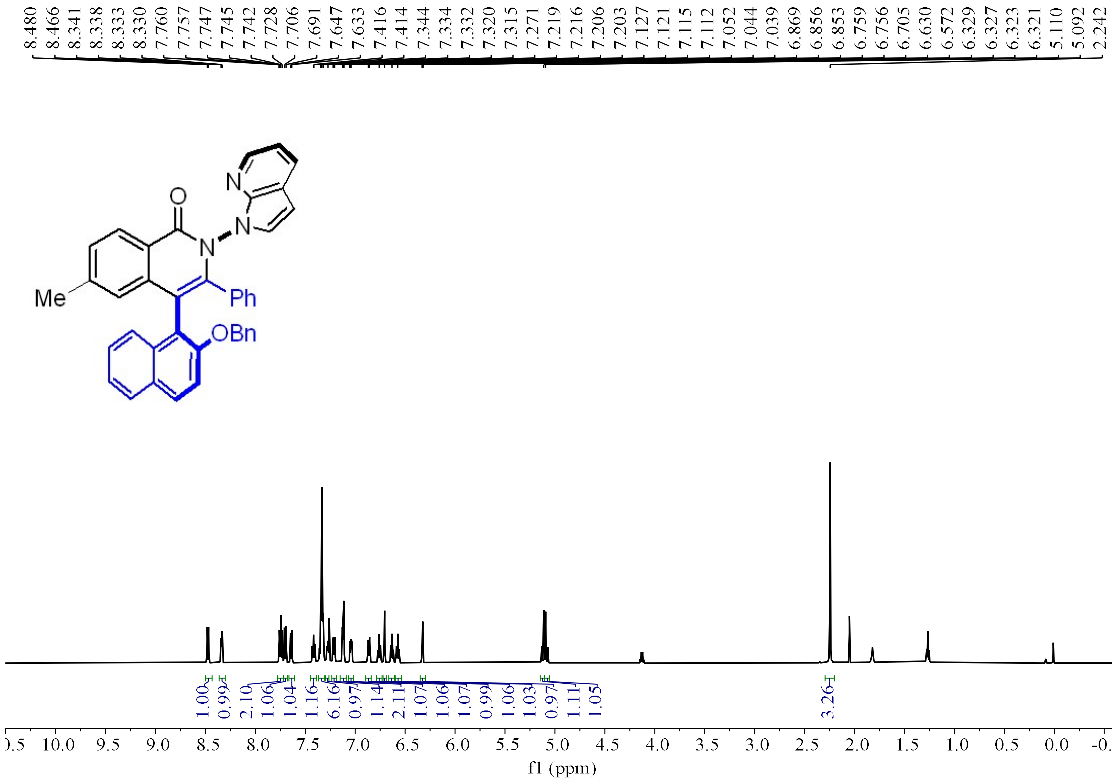


**Supplementary Figure 229.** $^{1}H$ **NMR (600 MHz, $CDCl_3$) spectrum of 50.**

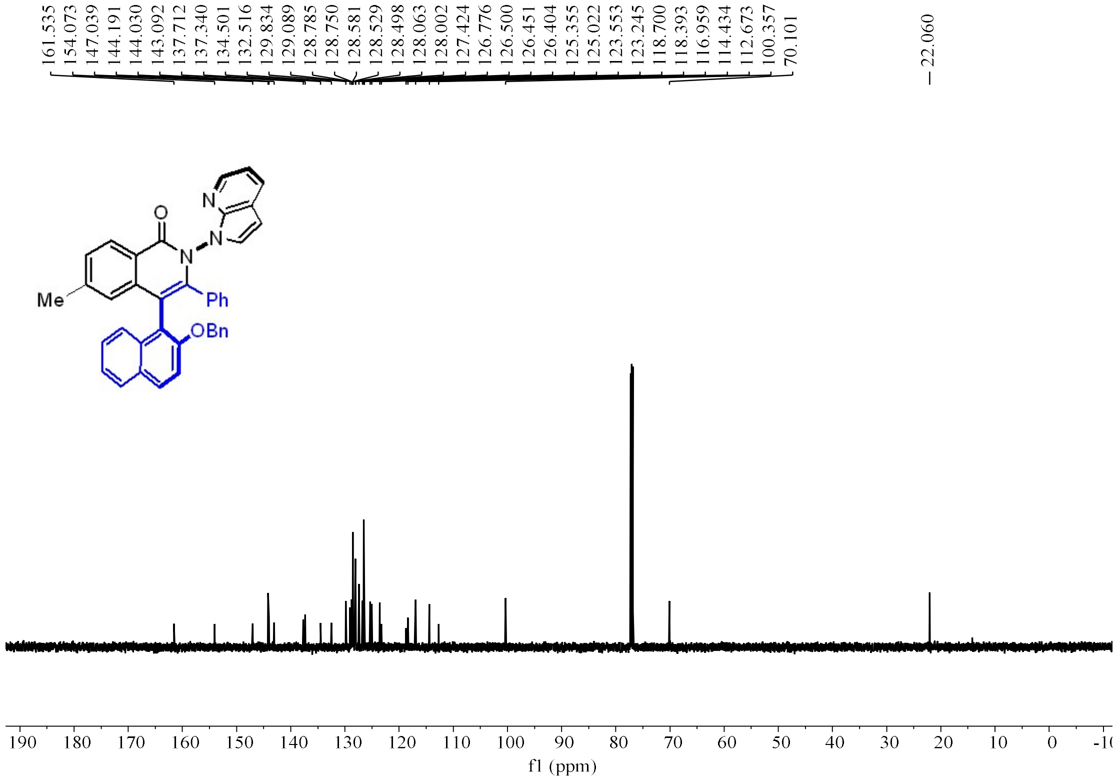


**Supplementary Figure 230.** $^{13}C$ **NMR (150 MHz, $CDCl_3$) spectrum of 50.**

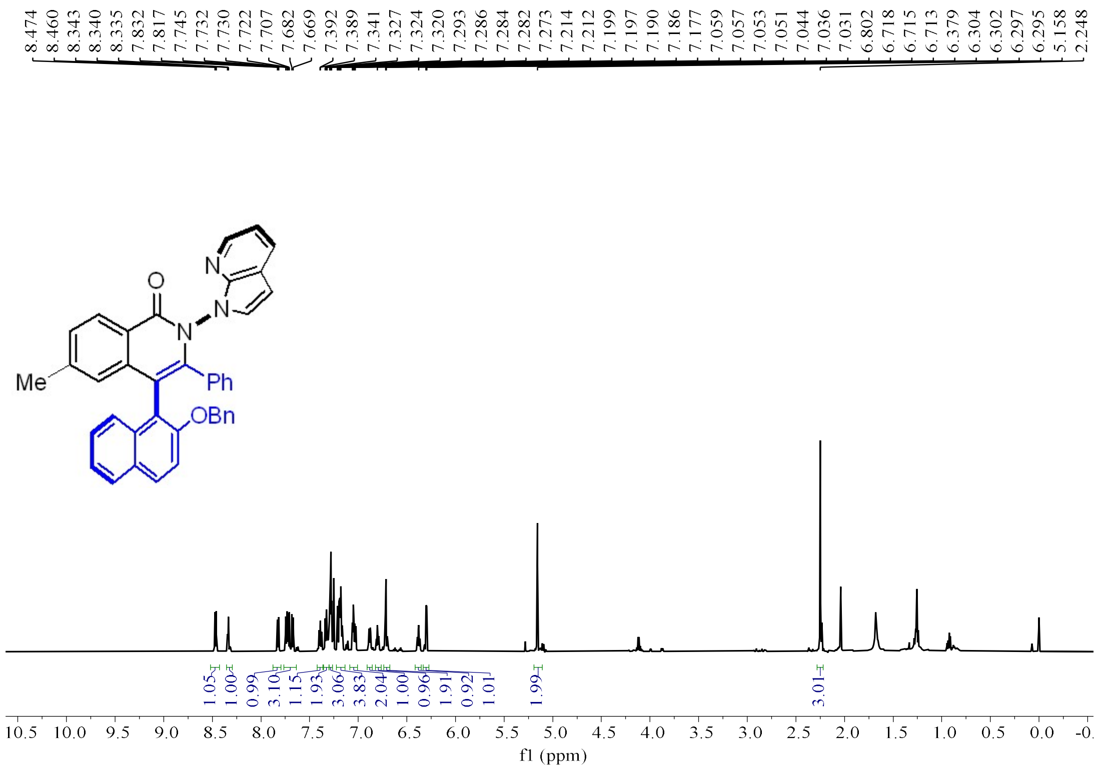


**Supplementary Figure 231.** **$^{1}$H NMR (600 MHz, $CDCl_3$) spectrum of dia-50.**

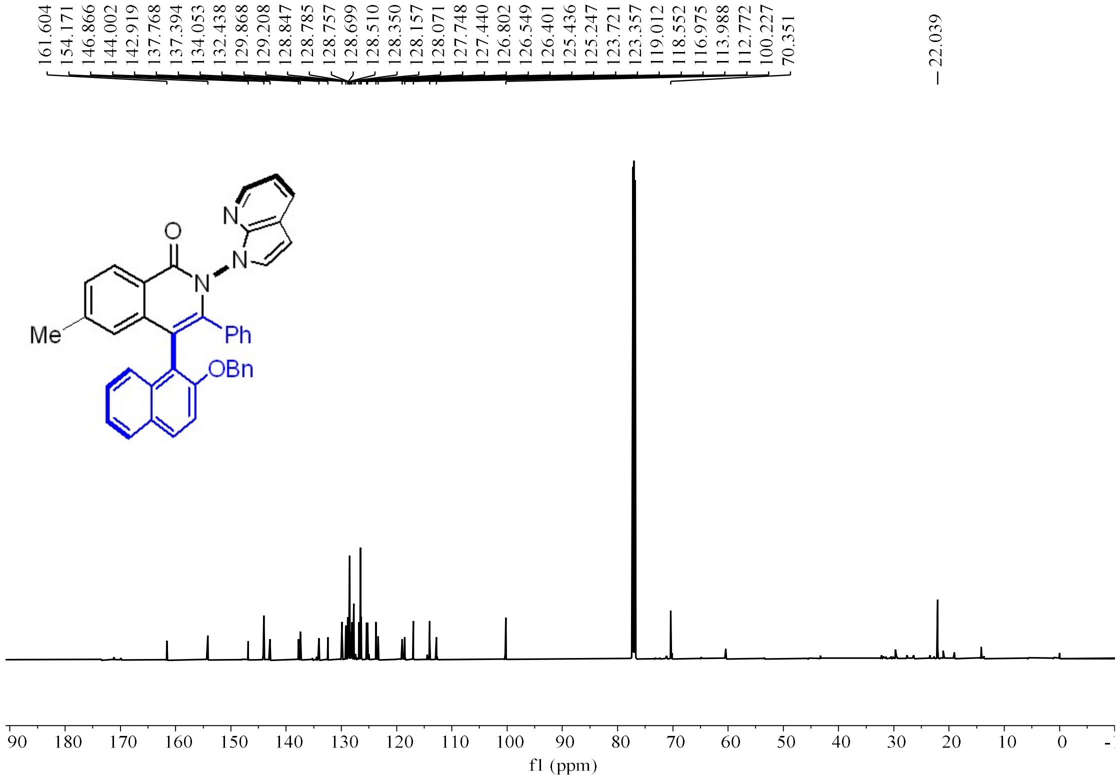


**Supplementary Figure 232.** **$^{13}$C NMR (150 MHz, $CDCl_3$) spectrum of dia-50.**

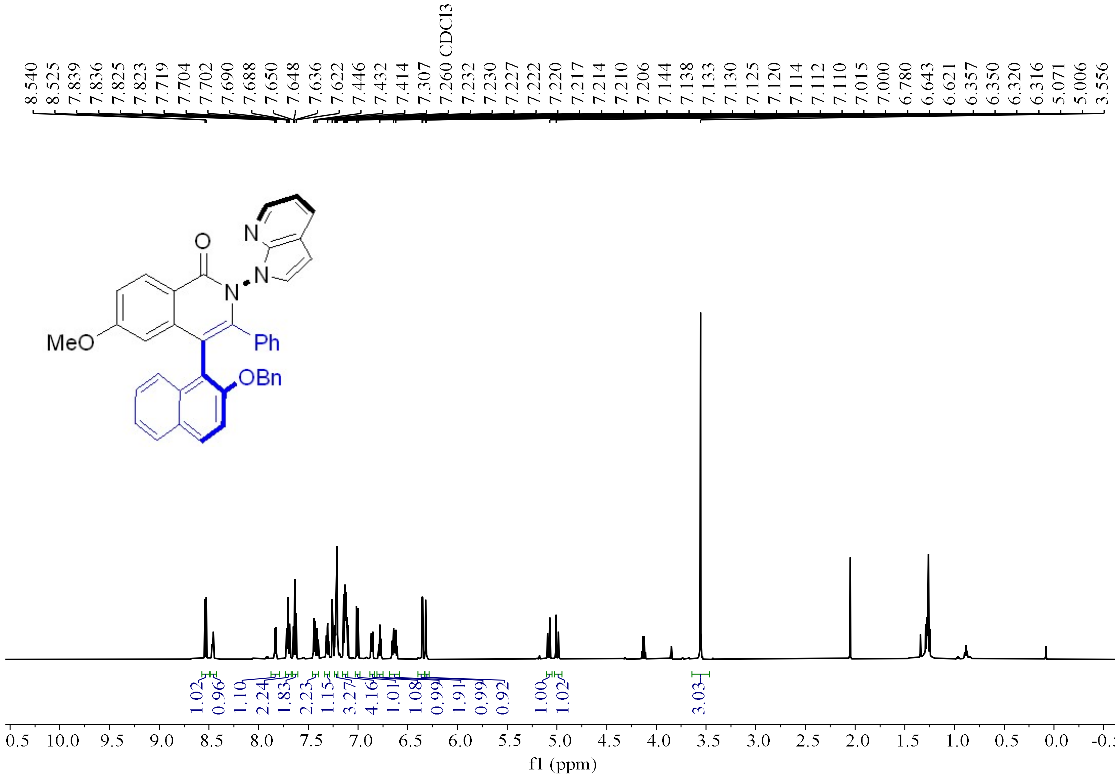

**Supplementary Figure 233. $^{1}$H NMR (600 MHz, CDCl$_3$) spectrum of 51.**

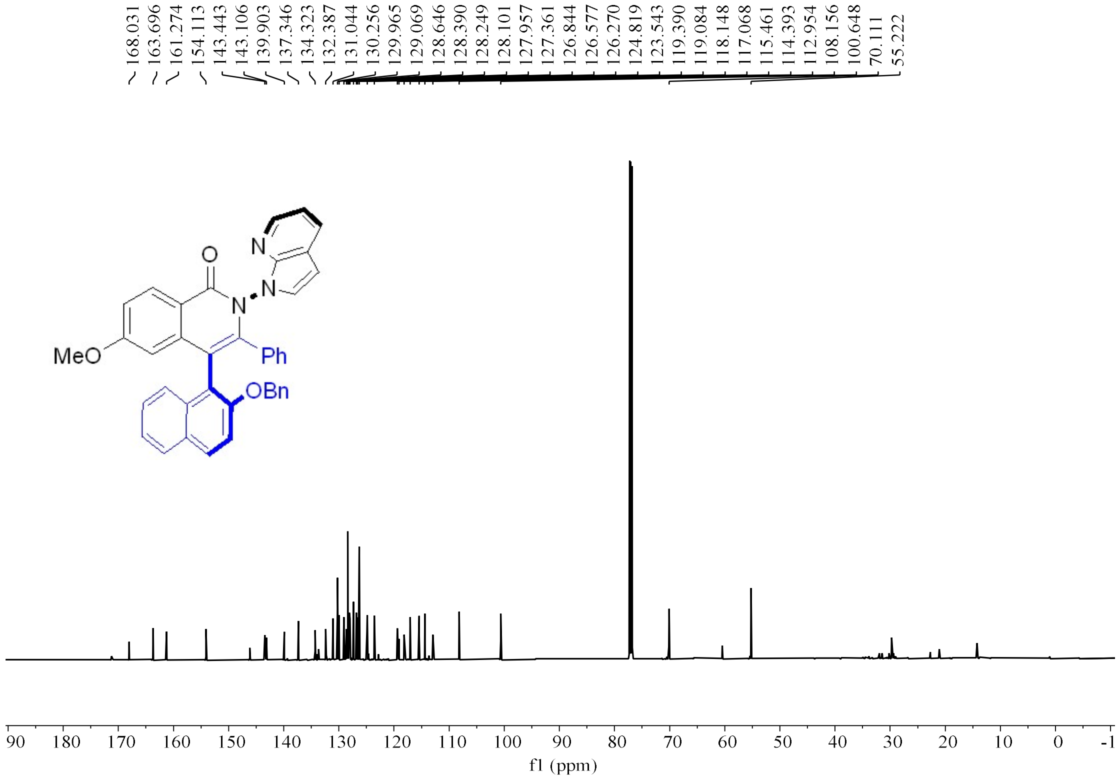

**Supplementary Figure 234. $^{13}$C NMR (150 MHz, CDCl$_3$) spectrum of 51.**

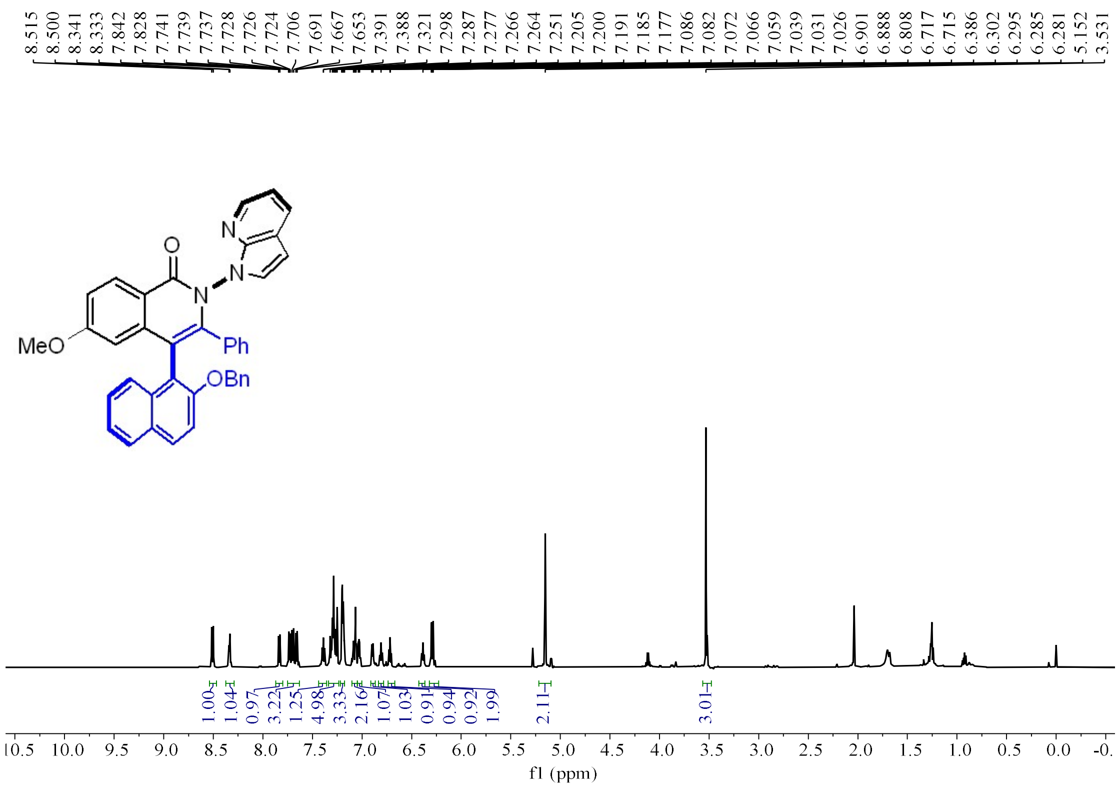


**Supplementary Figure 235.** $^{1}$**H NMR (600 MHz, CDCl$_3$) spectrum of dia-51.**

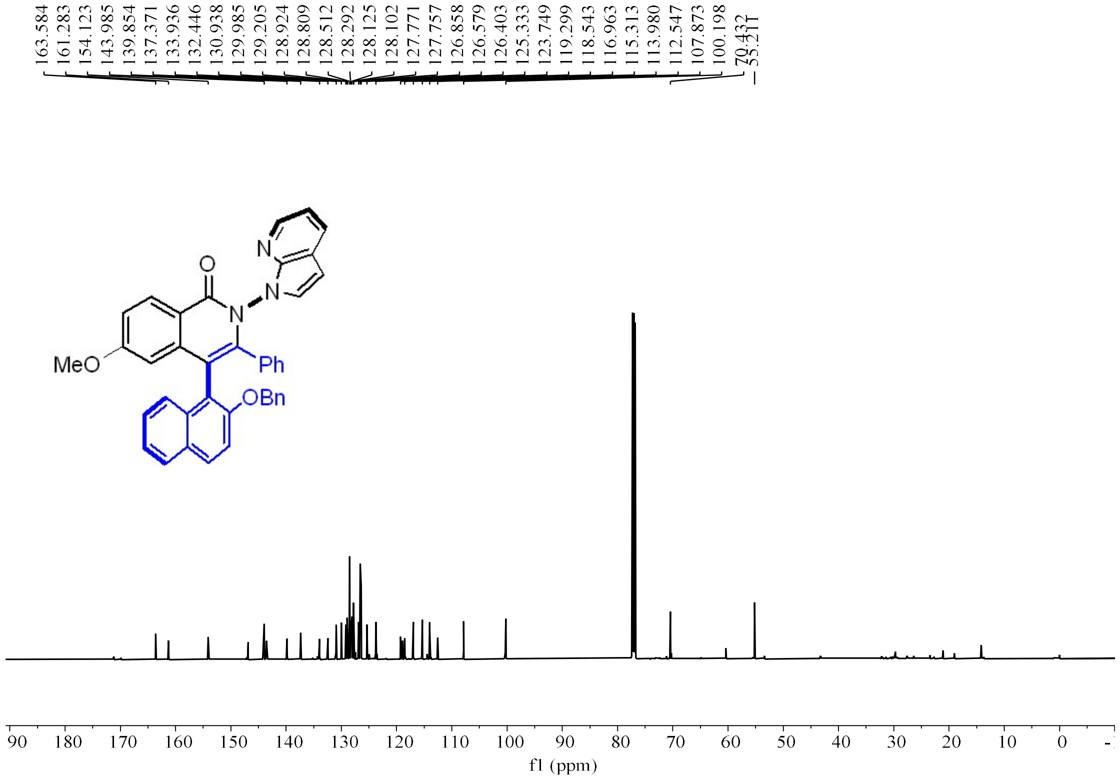


**Supplementary Figure 236.** $^{13}$**C NMR (150 MHz, CDCl$_3$) spectrum of dia-51.**

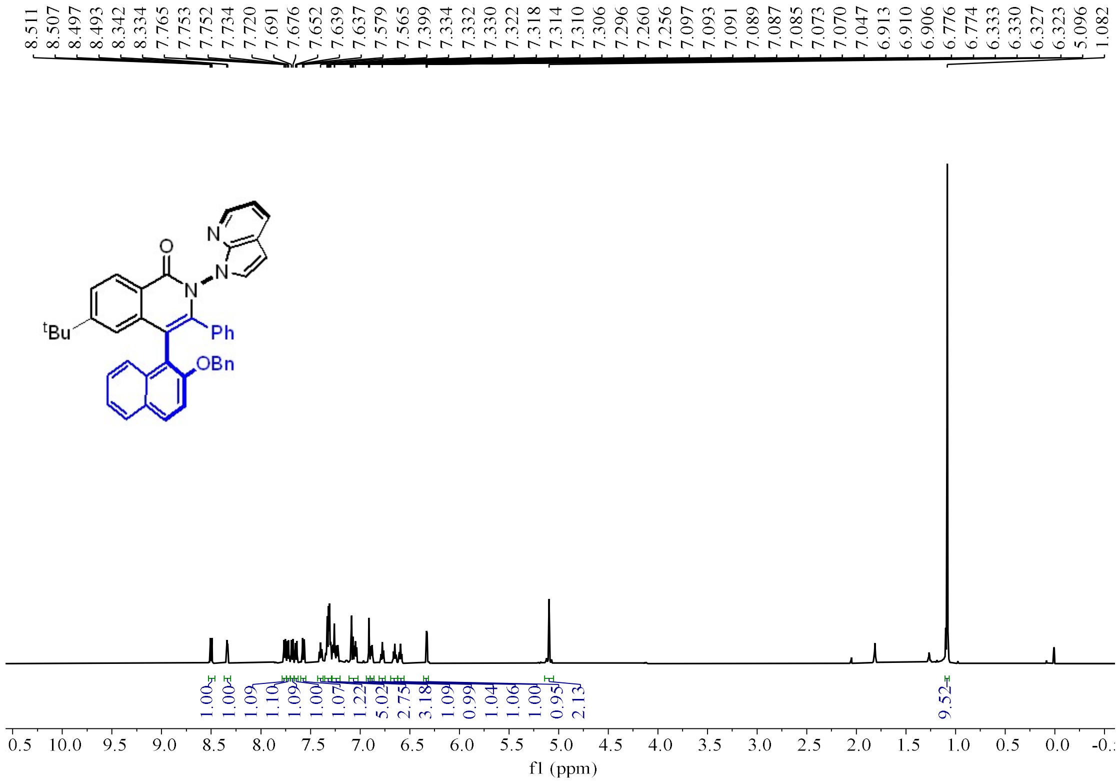


**Supplementary Figure 237. $^{1}$H NMR (600 MHz, $CDCl_3$) spectrum of 52.**

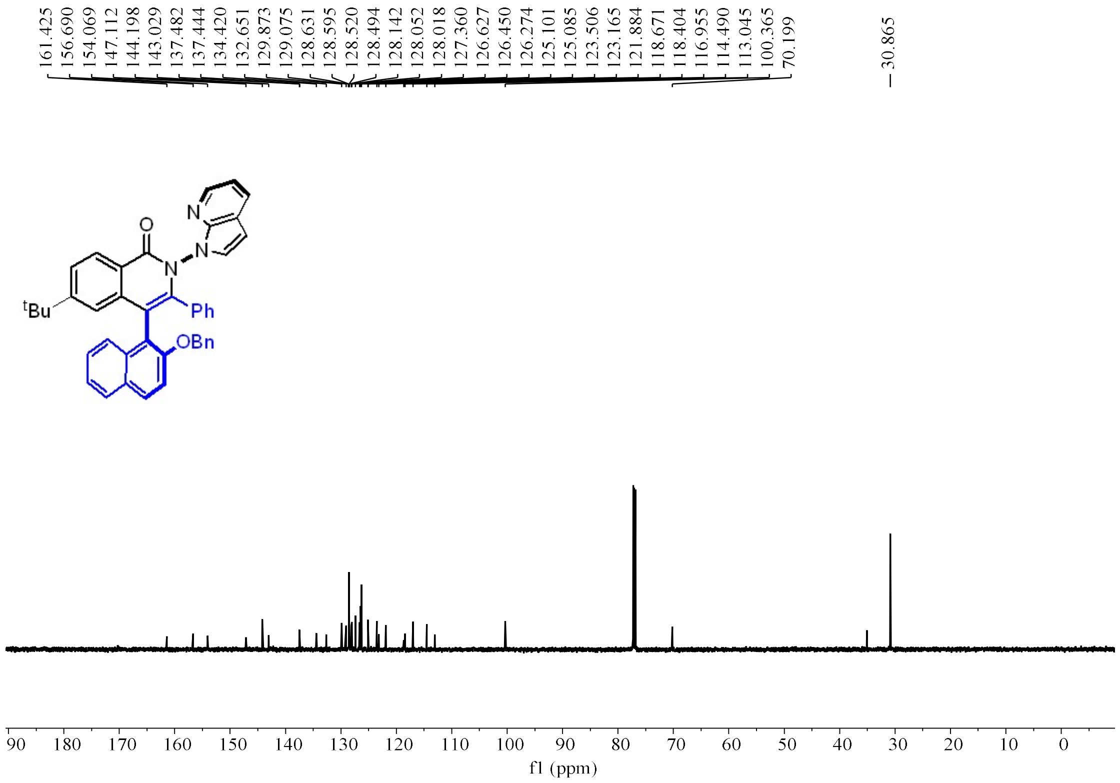


**Supplementary Figure 238. $^{13}$C NMR (150 MHz, $CDCl_3$) spectrum of 52.**

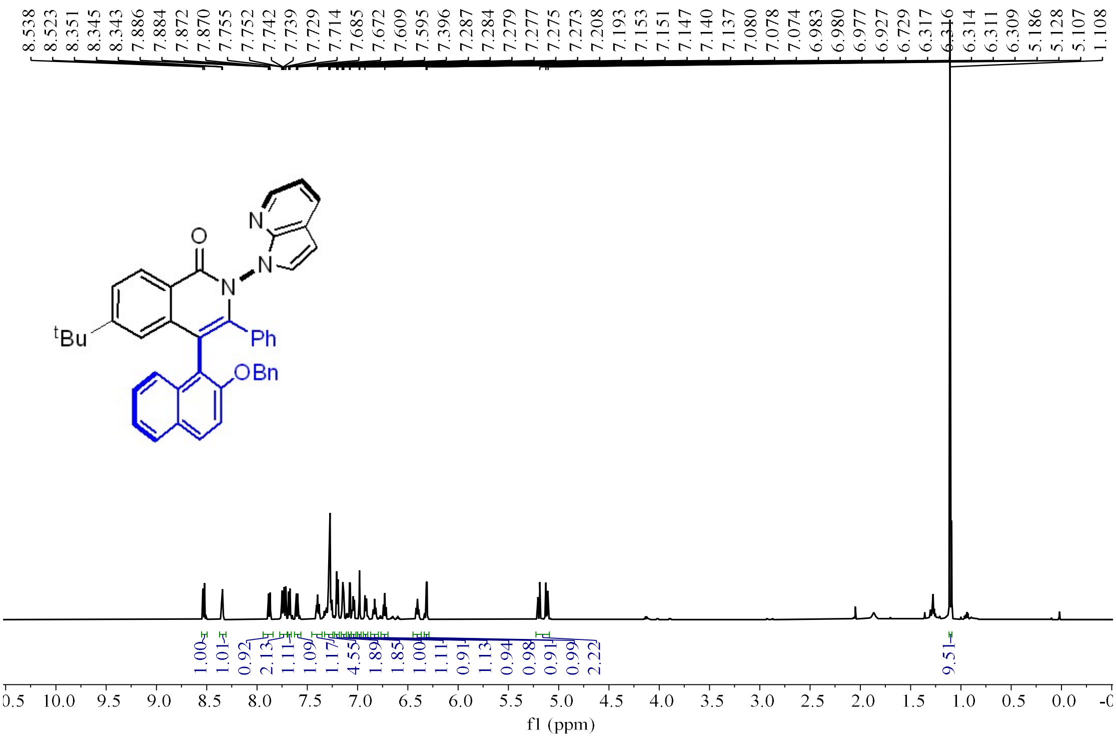

Supplementary Figure 239. $^{1}$H NMR (600 MHz, $CDCl_3$) spectrum of dia-52.

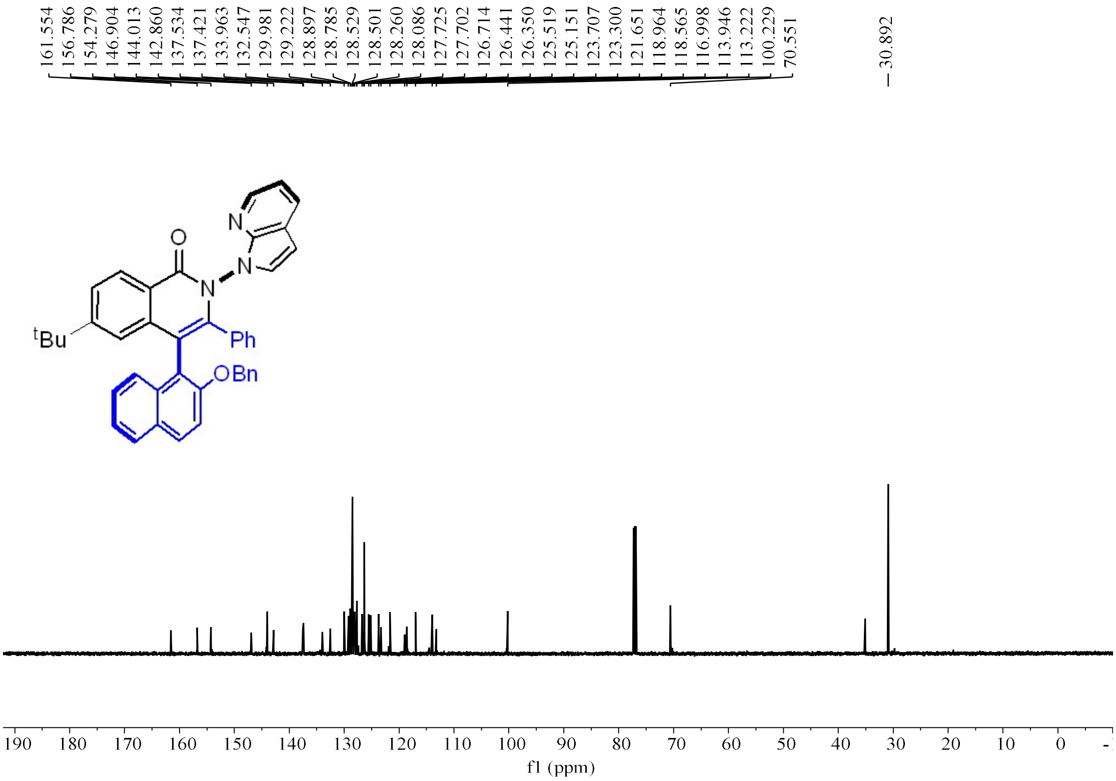

Supplementary Figure 240. $^{13}$C NMR (150 MHz, $CDCl_3$) spectrum of dia-52.

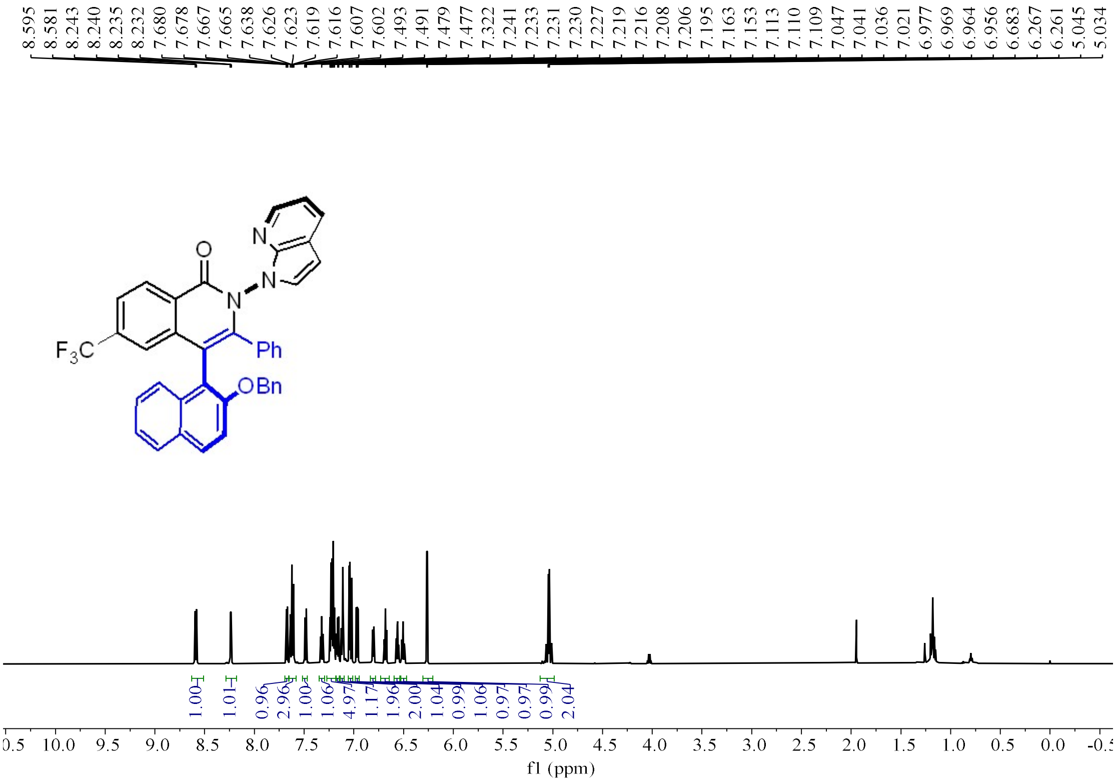


**Supplementary Figure 241.** $^{1}$**H NMR (600 MHz, CDCl3) spectrum of 53.**

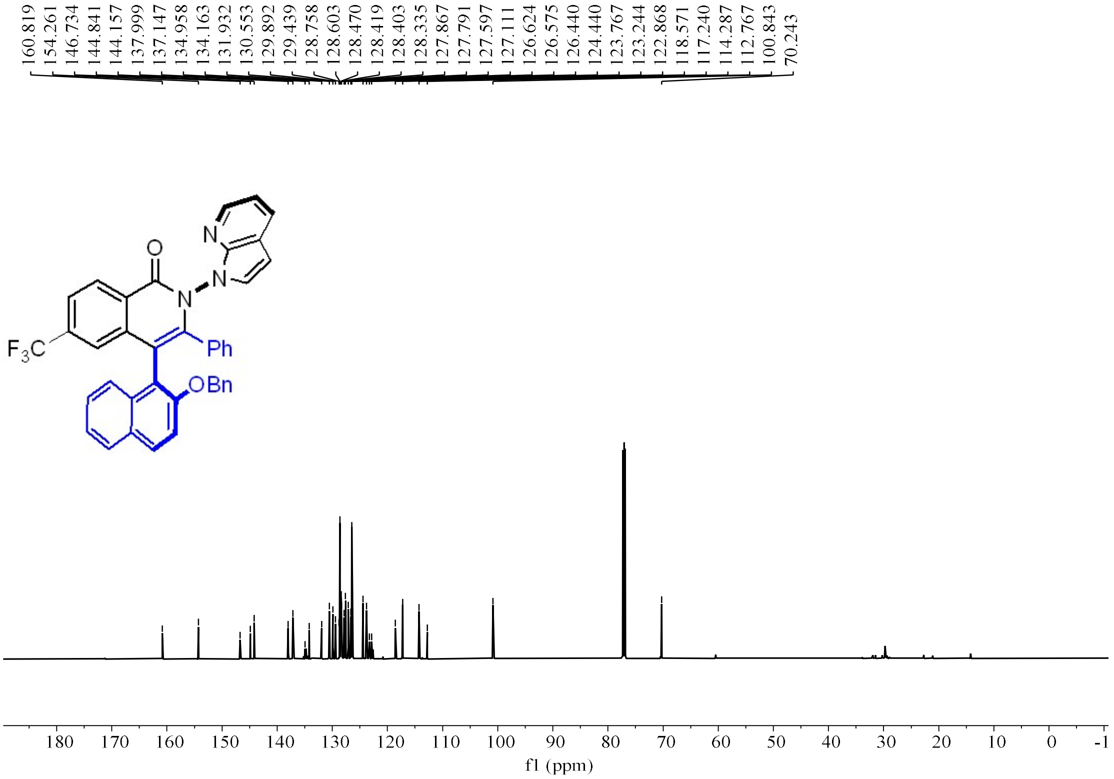


**Supplementary Figure 242.** $^{13}$**C NMR (150 MHz, CDCl3) spectrum of 53.**

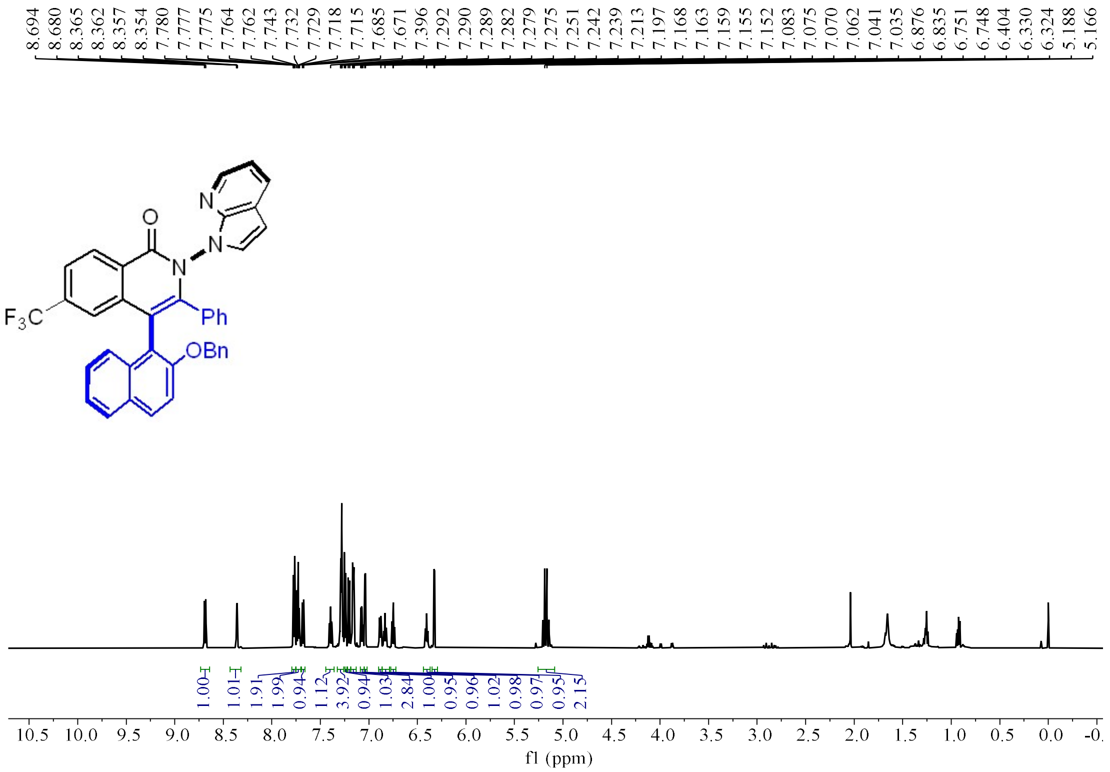


**Supplementary Figure 243.** $^{1}$**H NMR (600 MHz, CDCl$_3$) spectrum of dia-53.**

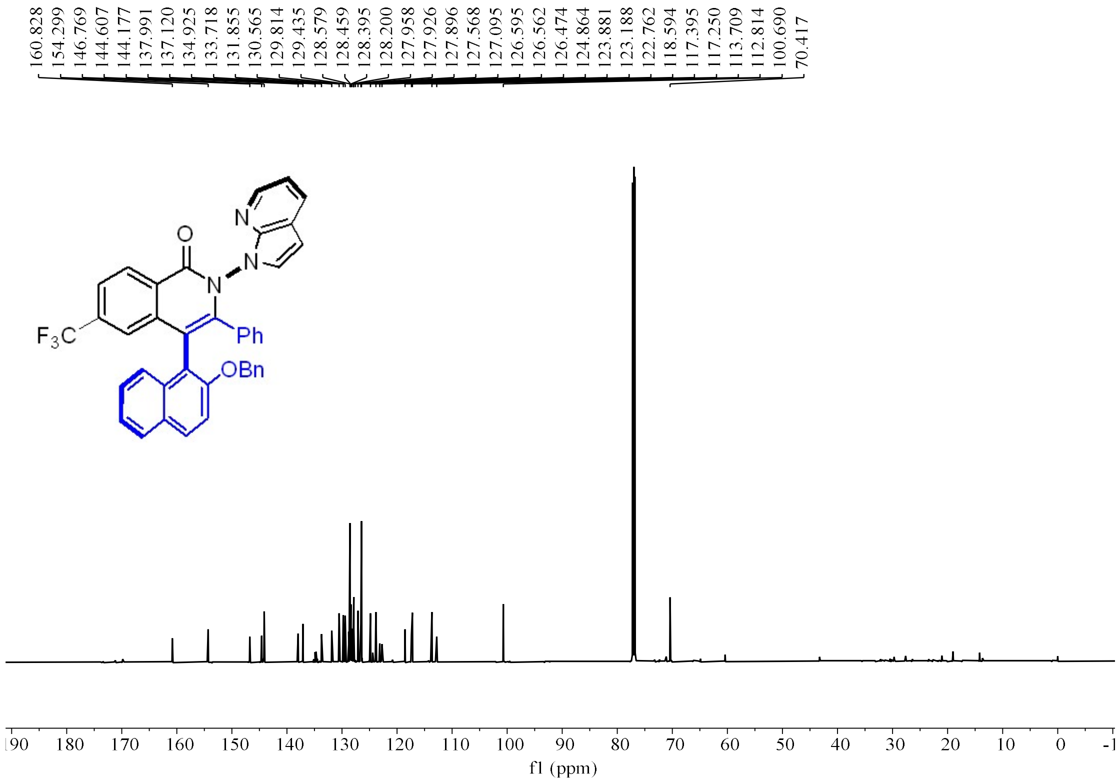


**Supplementary Figure 244.** $^{13}$**C NMR (150 MHz, CDCl$_3$) spectrum of dia-53.**

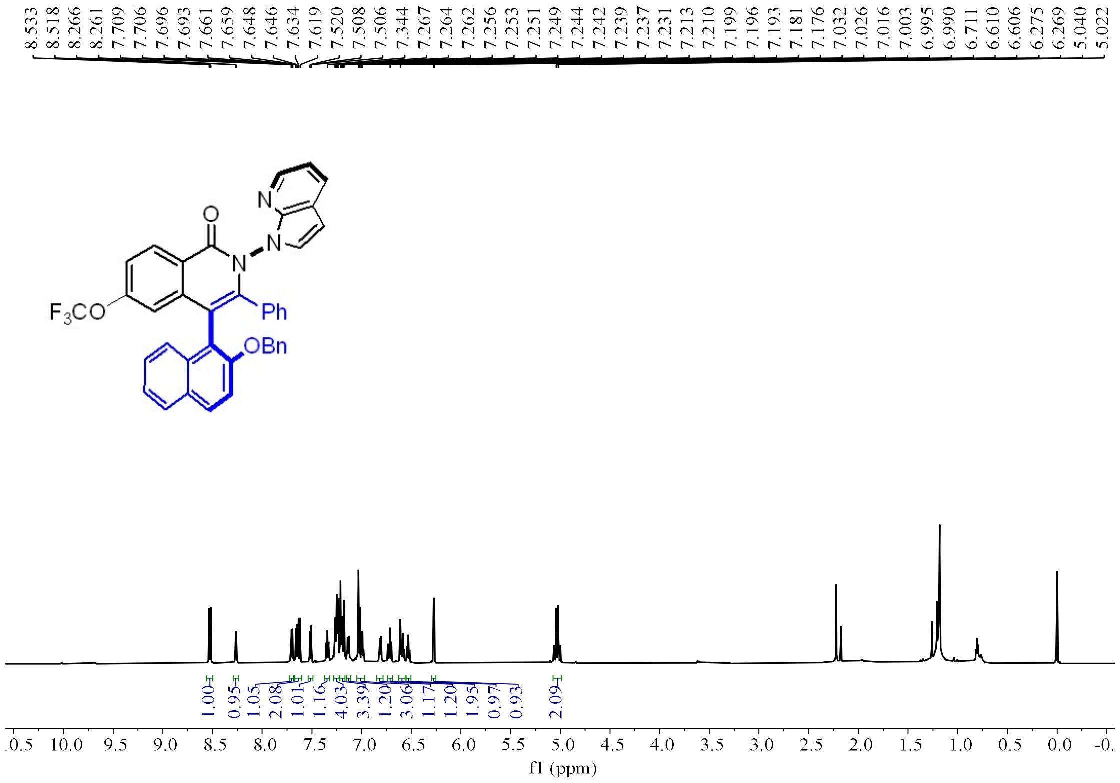


**Supplementary Figure 245. $^{1}H$ NMR (600 MHz, $CDCl_3$) spectrum of 54.**

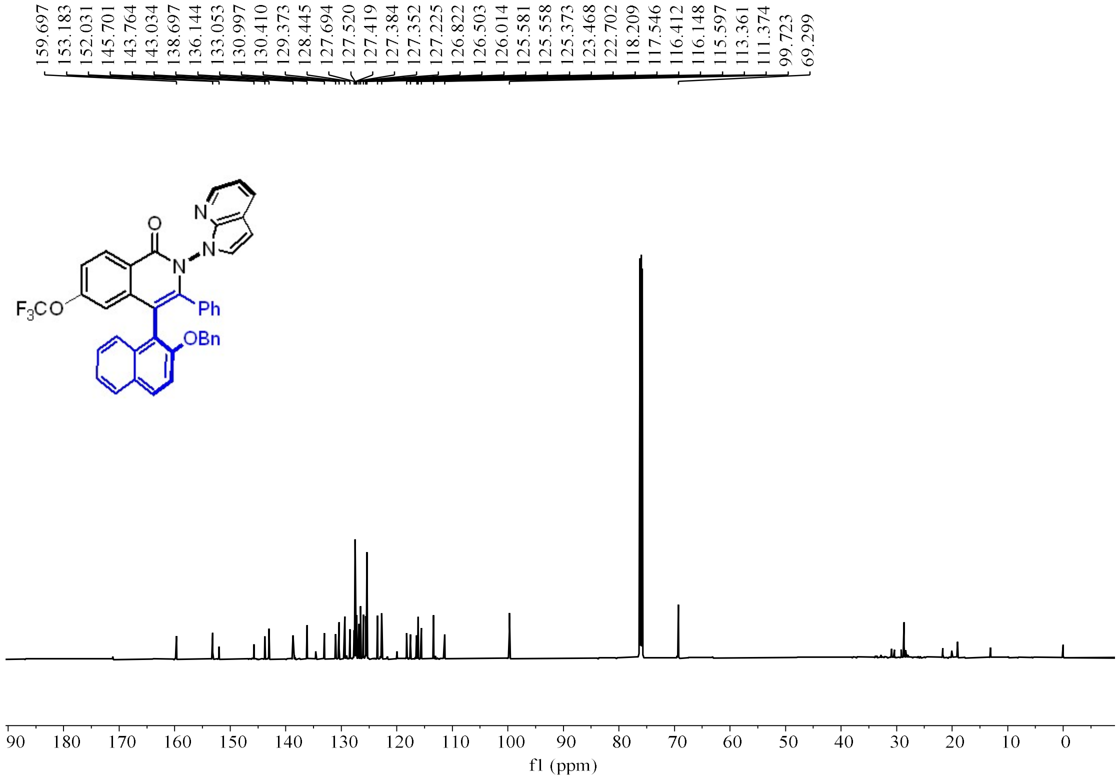


**Supplementary Figure 246. $^{13}C$ NMR (150 MHz, $CDCl_3$) spectrum of 54.**

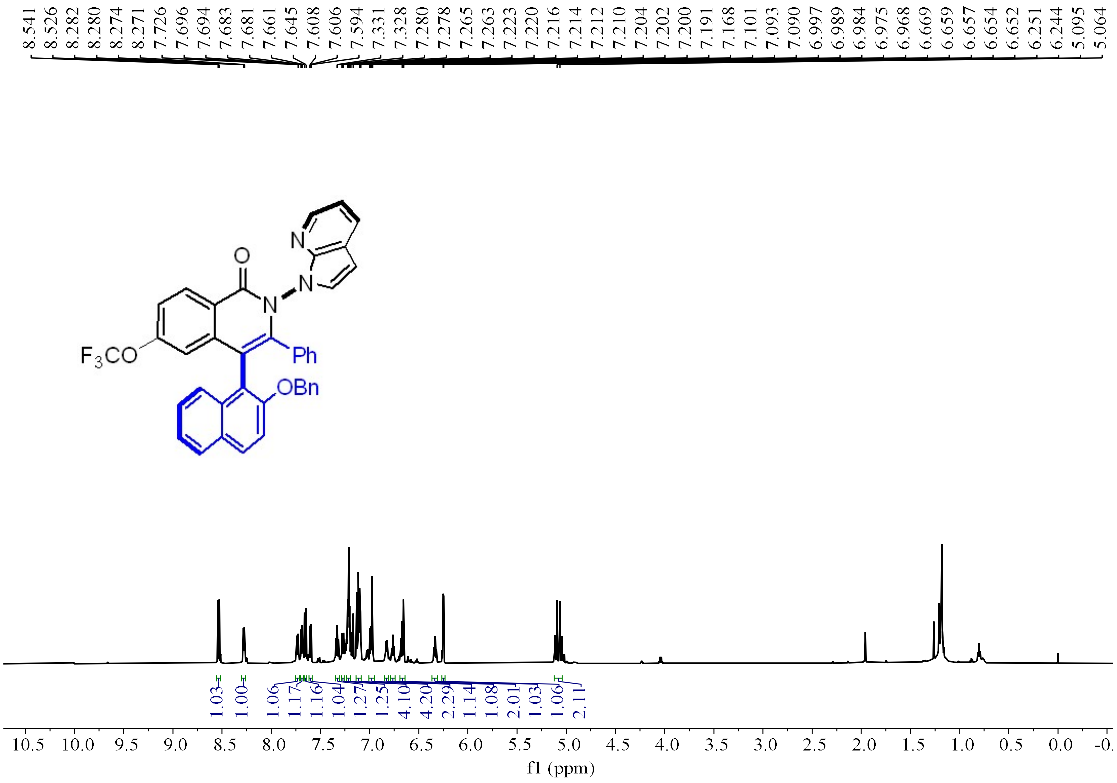


**Supplementary Figure 247. $^1$H NMR (600 MHz, $CDCl_3$) spectrum of dia-54.**

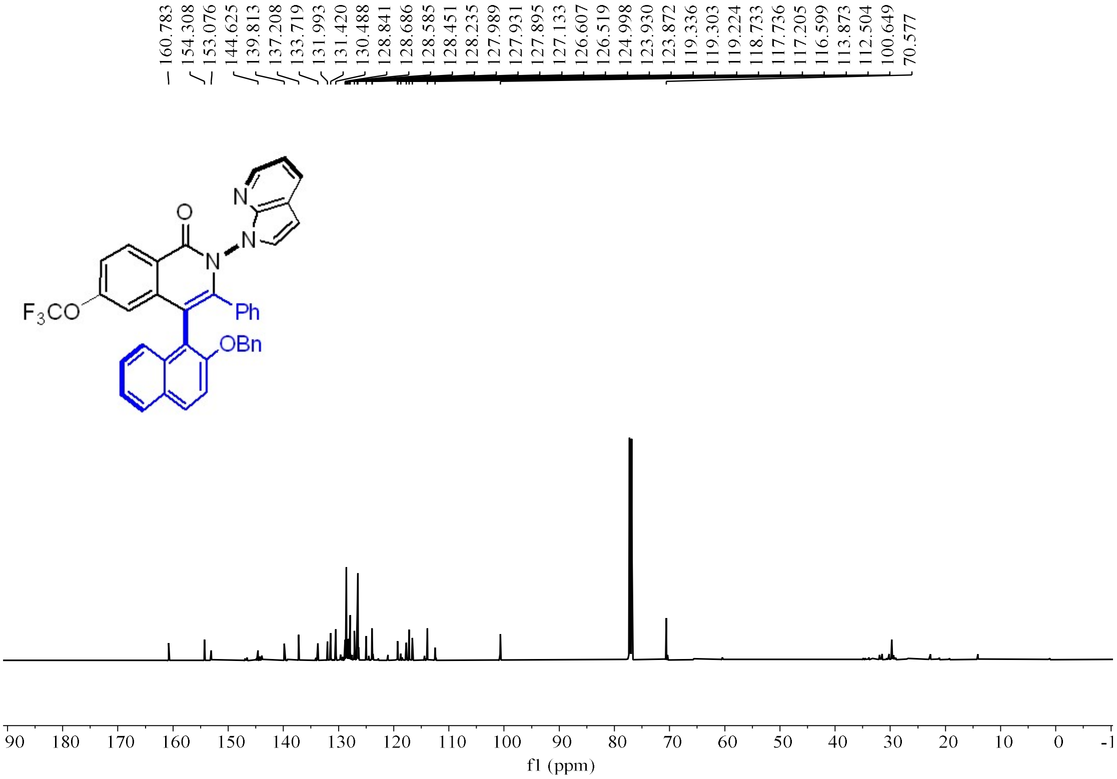


**Supplementary Figure 248. $^{13}C$ NMR (150 MHz, $CDCl_3$) spectrum of dia-54.**

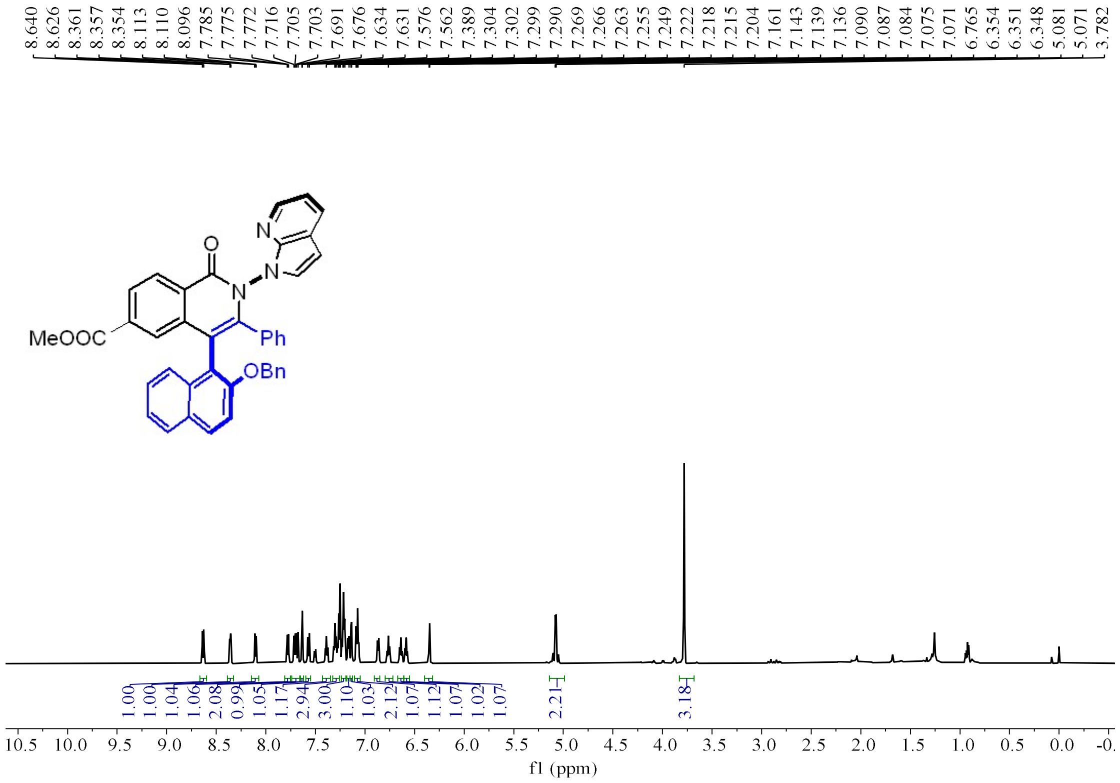

Supplementary Figure 249. $^{1}$H NMR (600 MHz, CDCl$_3$) spectrum of 55.

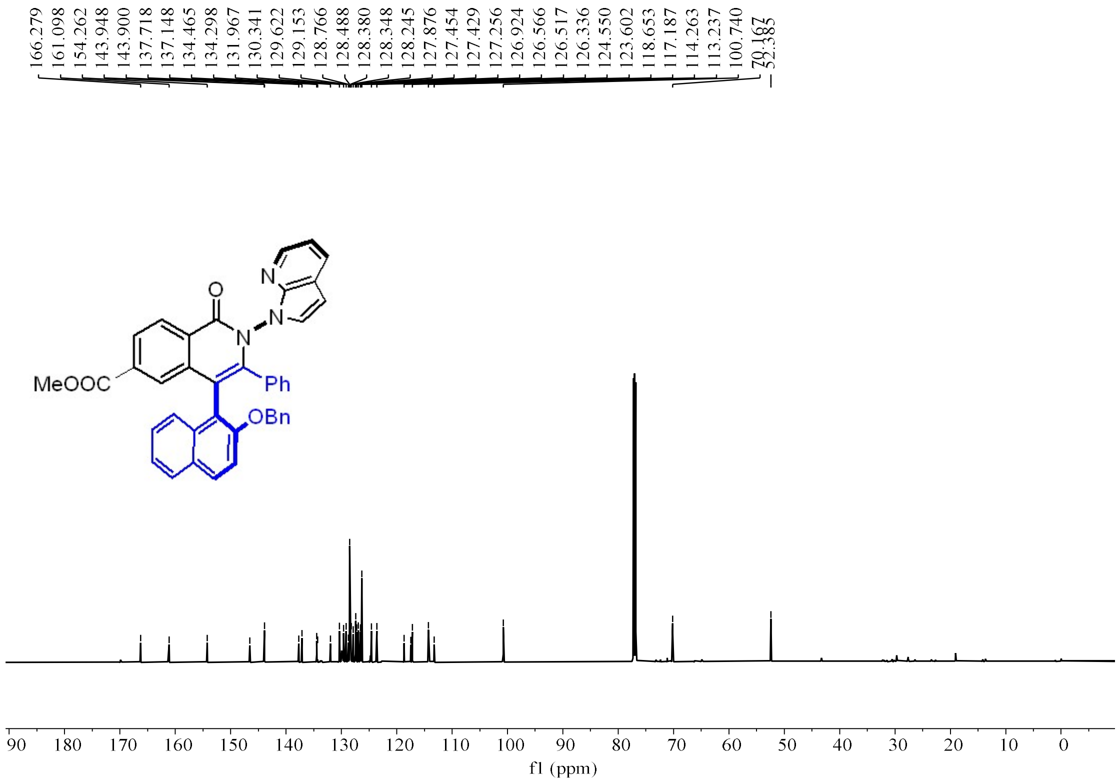

Supplementary Figure 250. $^{13}$C NMR (150 MHz, CDCl$_3$) spectrum of 55.

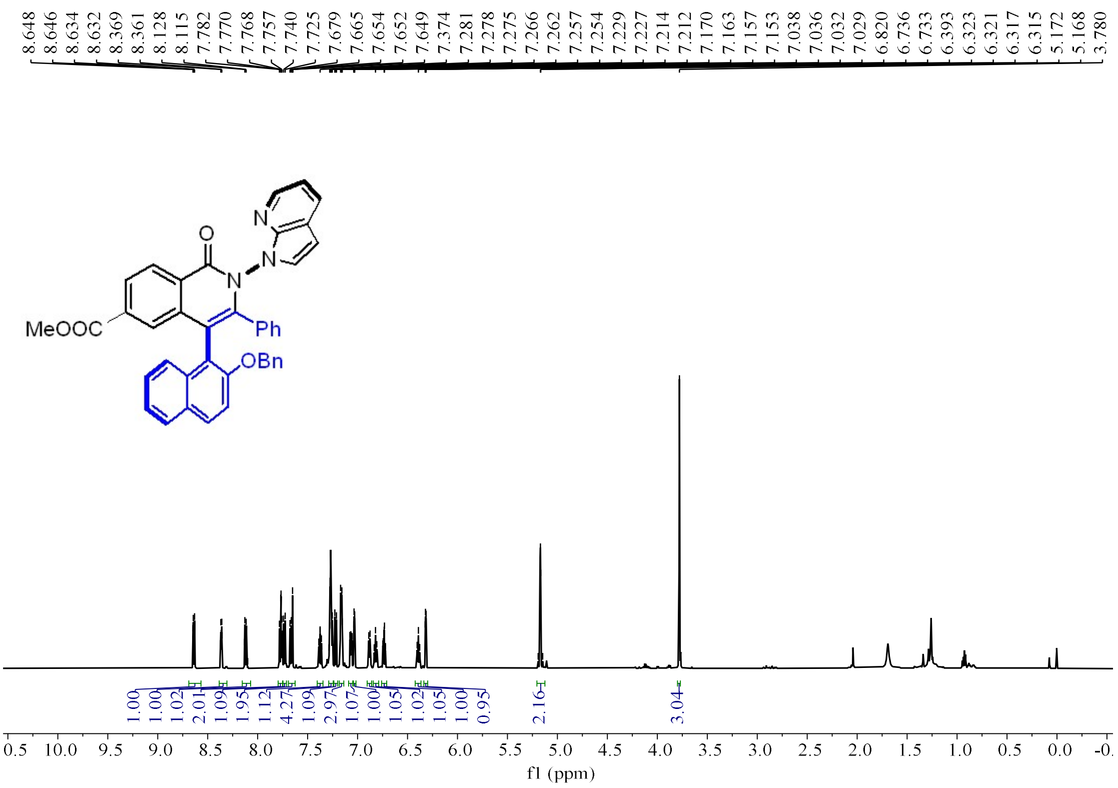


**Supplementary Figure 251. $^{1}$H NMR (600 MHz, $CDCl_3$) spectrum of dia-55.**

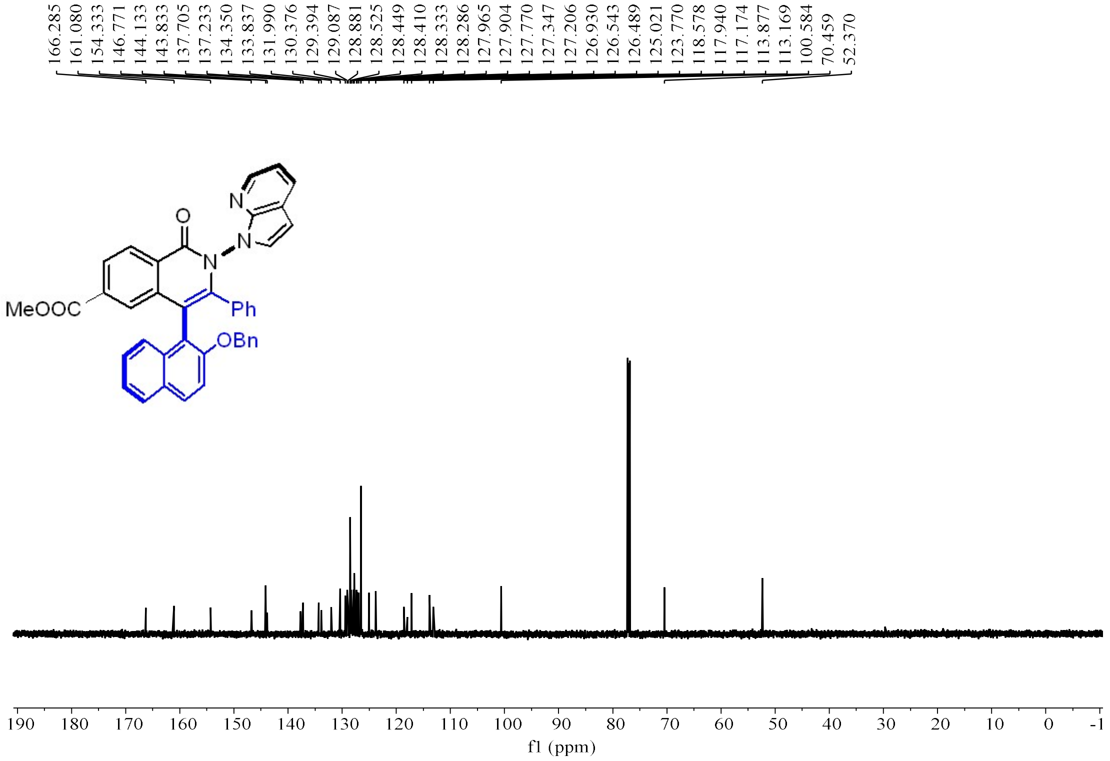


**Supplementary Figure 252. $^{13}$C NMR (150 MHz, $CDCl_3$) spectrum of dia-55.**

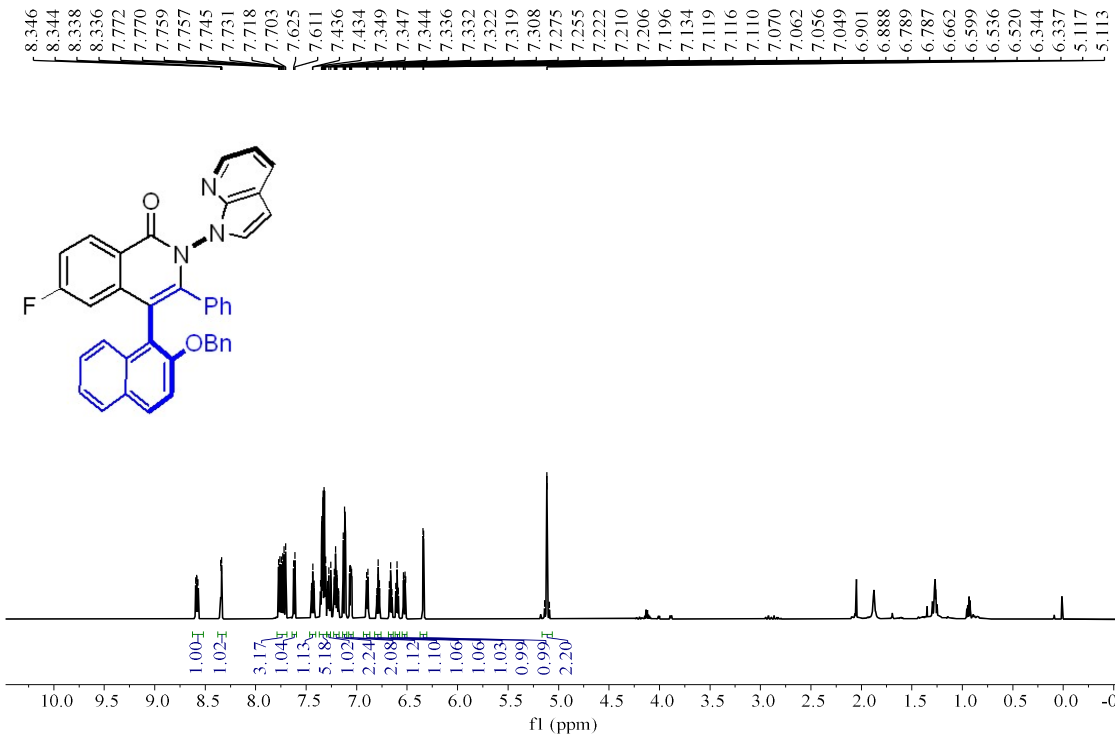


**Supplementary Figure 253.** $^{1}$**H NMR (600 MHz,** $CDCl_3$**) spectrum of 56.**

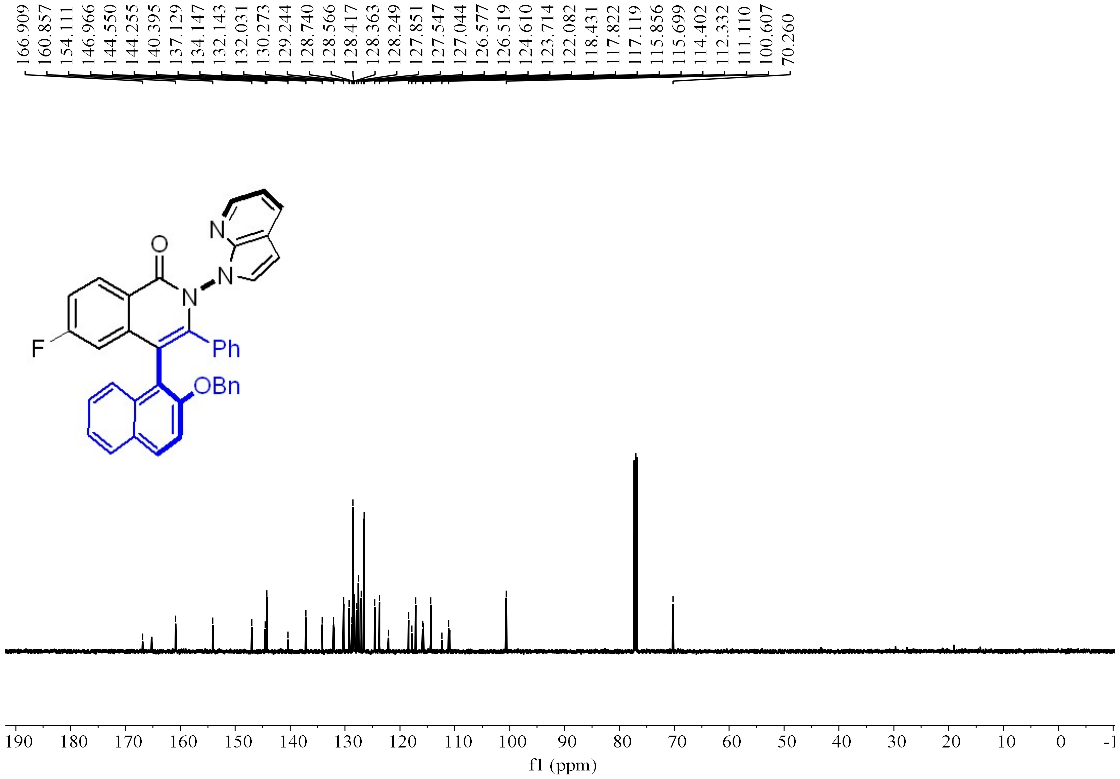


**Supplementary Figure 254.** $^{13}$**C NMR (150 MHz,** $CDCl_3$**) spectrum of 56.**

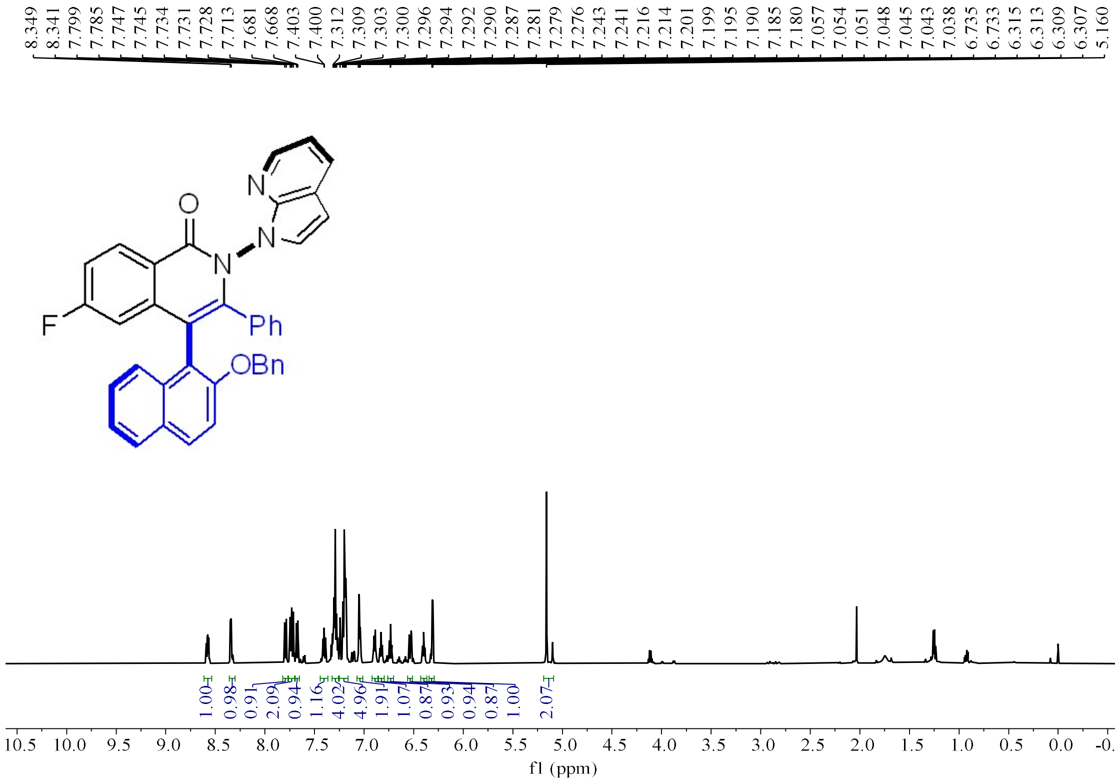


**Supplementary Figure 255. $^{1}$H NMR (600 MHz, $CDCl_3$) spectrum of dia-56.**

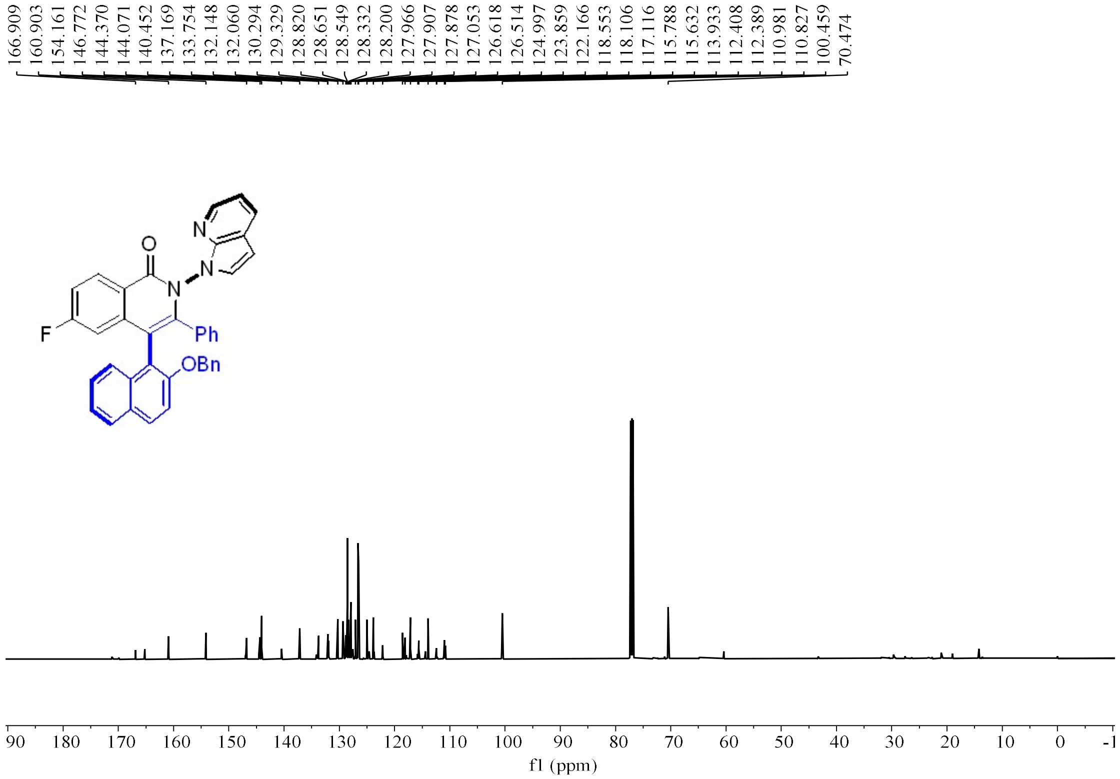


**Supplementary Figure 256. $^{13}$C NMR (150 MHz, $CDCl_3$) spectrum of dia-56.**

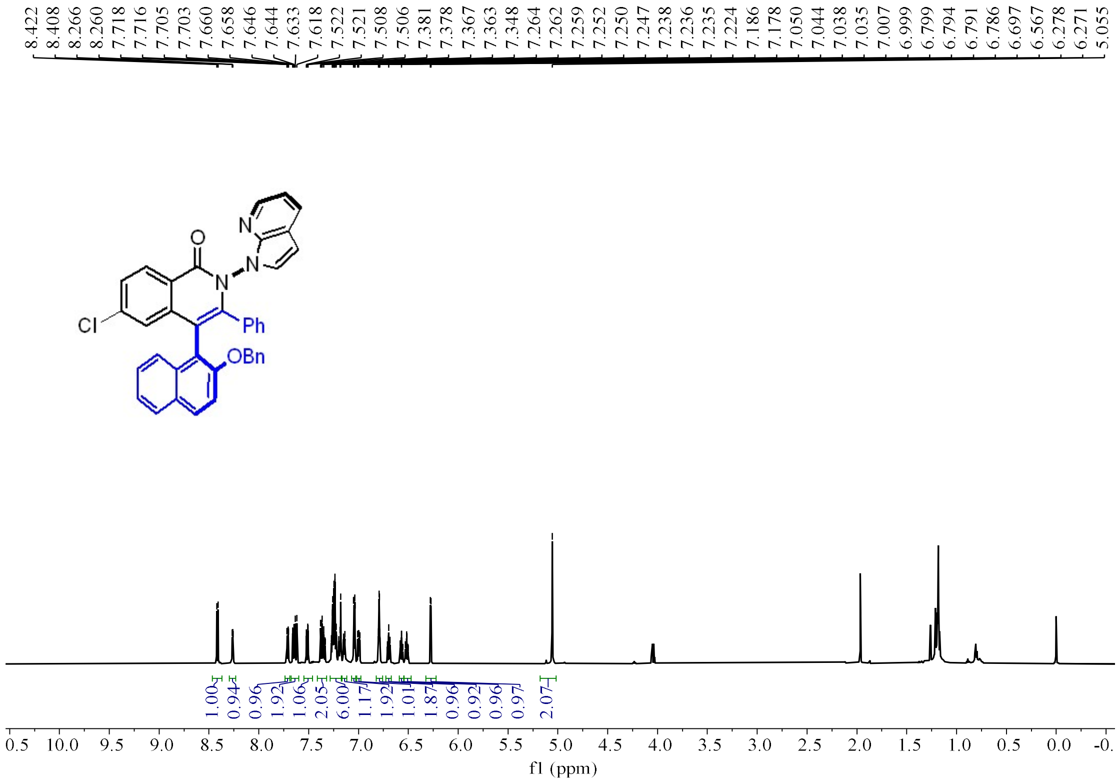

**Supplementary Figure 257. $^{1}$H NMR (600 MHz, $CDCl_3$) spectrum of 57.**

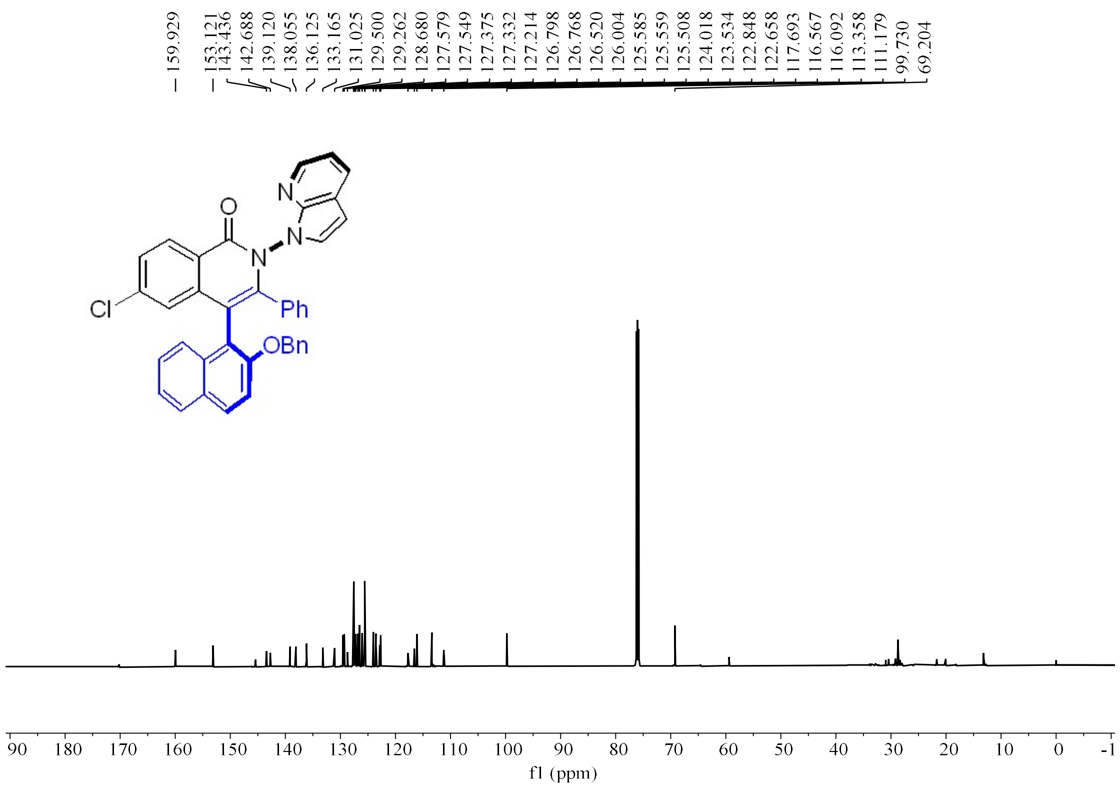

**Supplementary Figure 258. $^{13}$C NMR (150 MHz, $CDCl_3$) spectrum of 57.**

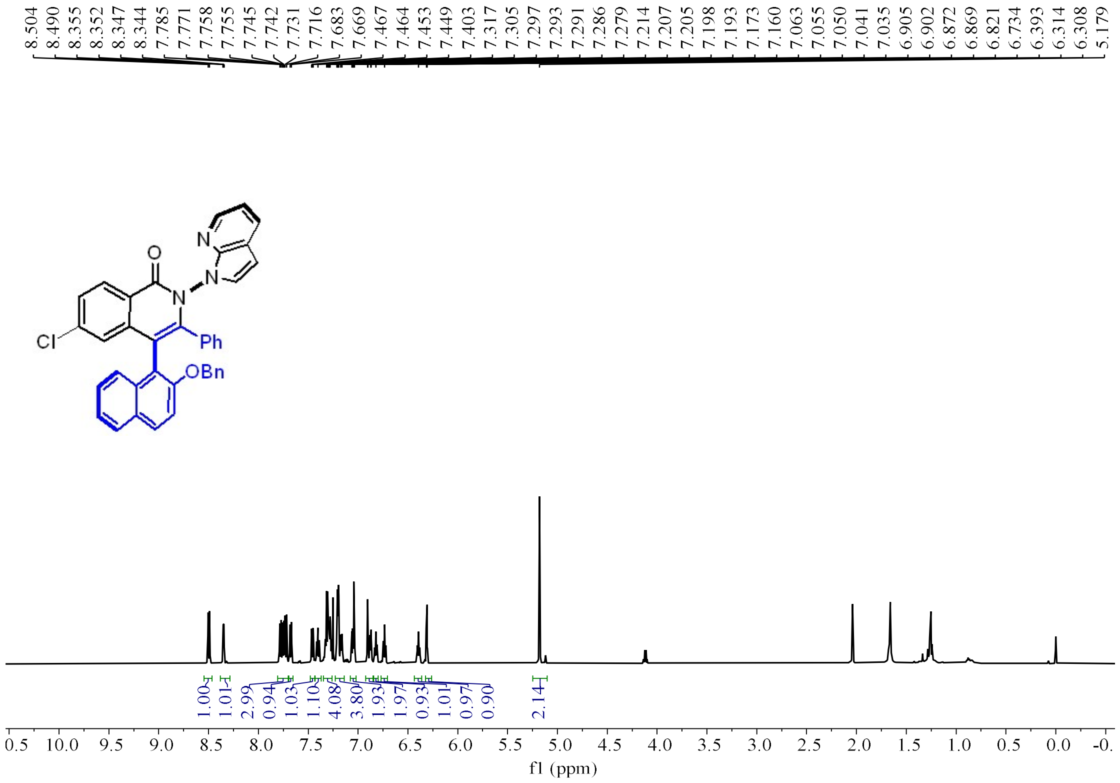


**Supplementary Figure 259.** $^{1}$**H NMR (600 MHz,** $CDCl_3$**) spectrum of dia-57.**

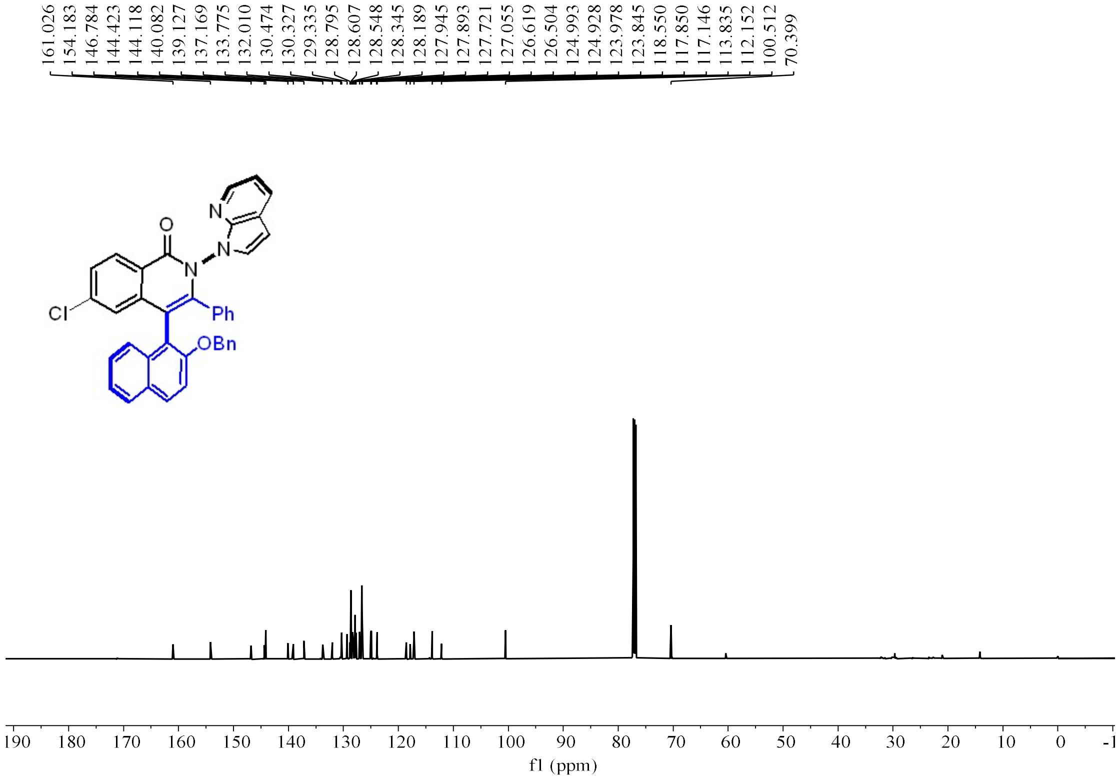


**Supplementary Figure 260.** $^{13}$**C NMR (150 MHz,** $CDCl_3$**) spectrum of diaa-57.**

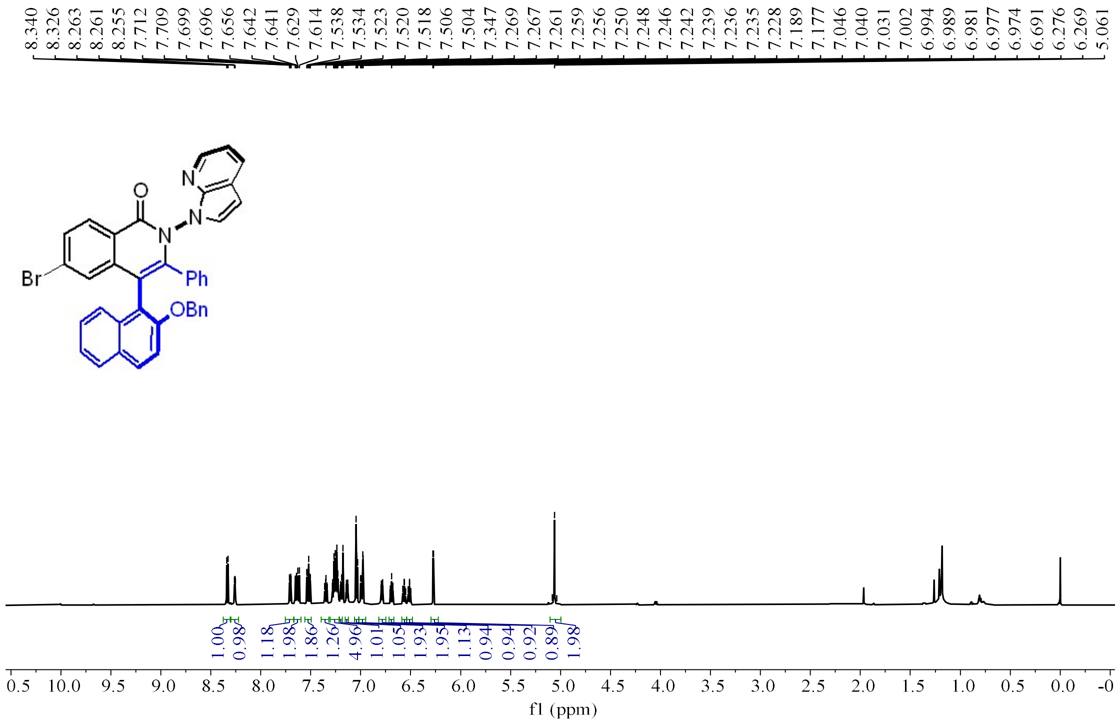

**Supplementary Figure 261. $^{1}H$ NMR (600 MHz, $CDCl_3$) spectrum of 58.**

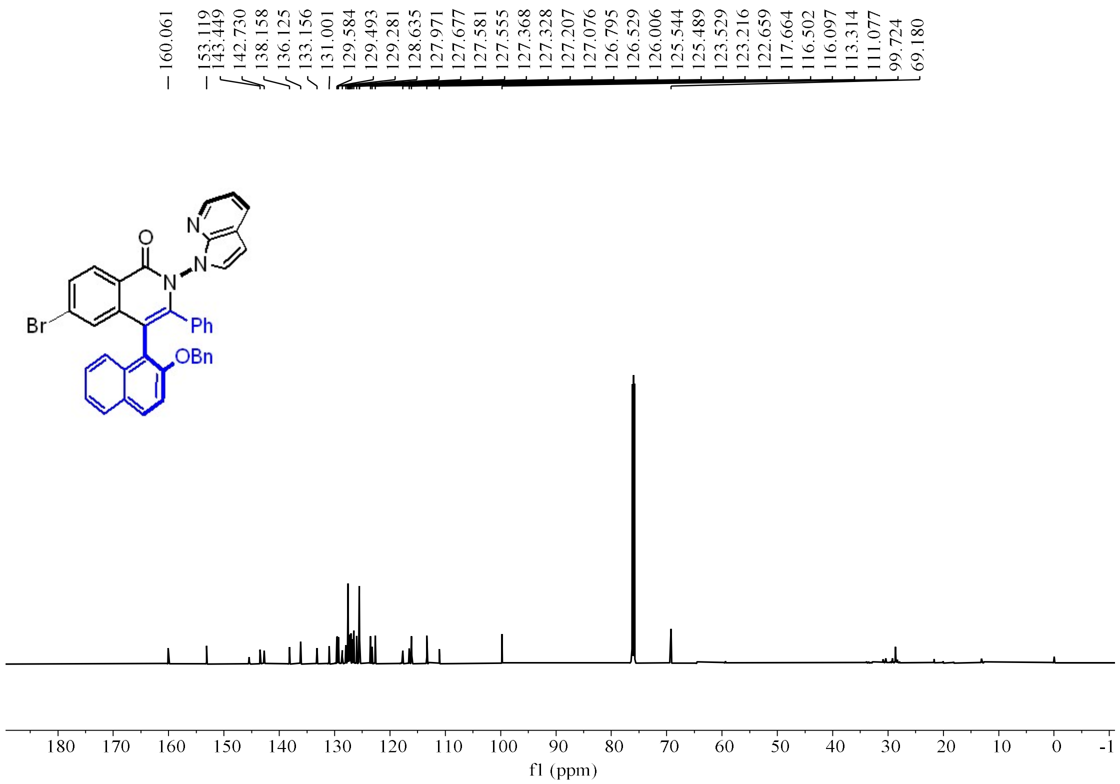

**Supplementary Figure 262. $^{13}C$ NMR (150 MHz, $CDCl_3$) spectrum of 58.**

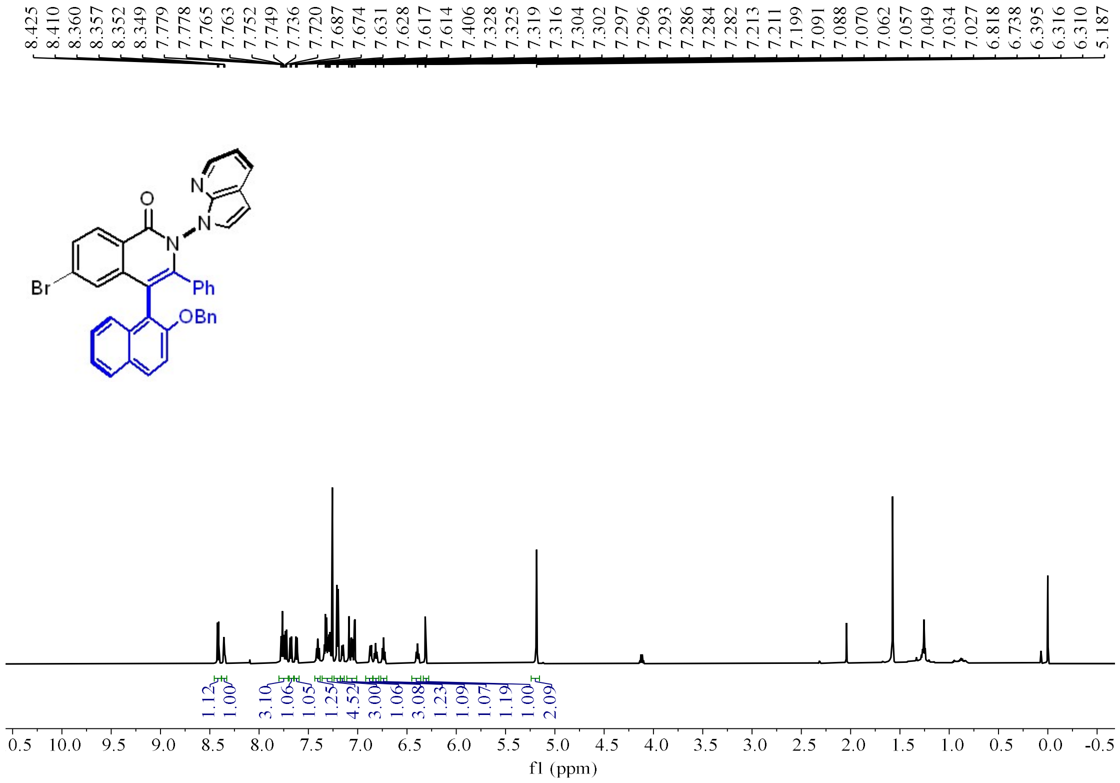


**Supplementary Figure 263. $^{1}$H NMR (600 MHz, CDCl$_3$) spectrum of dia-58.**

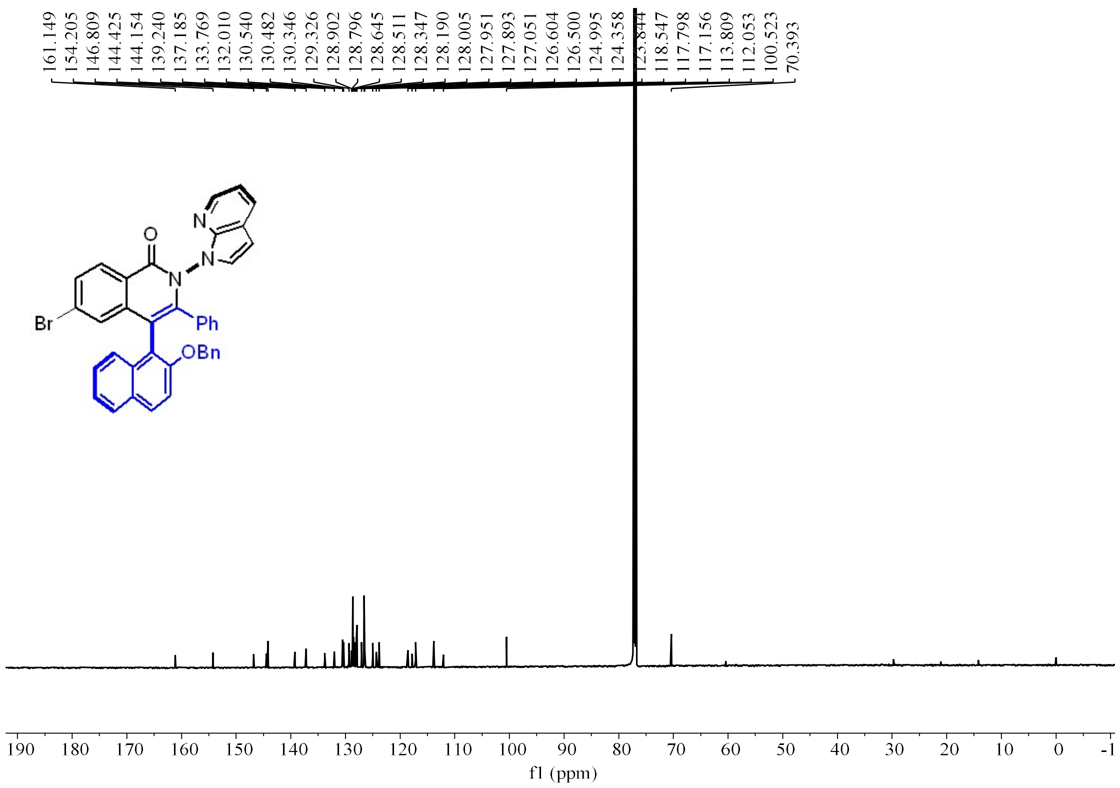


**Supplementary Figure 264. $^{13}$C NMR (150 MHz, CDCl$_3$) spectrum of dia-58.**

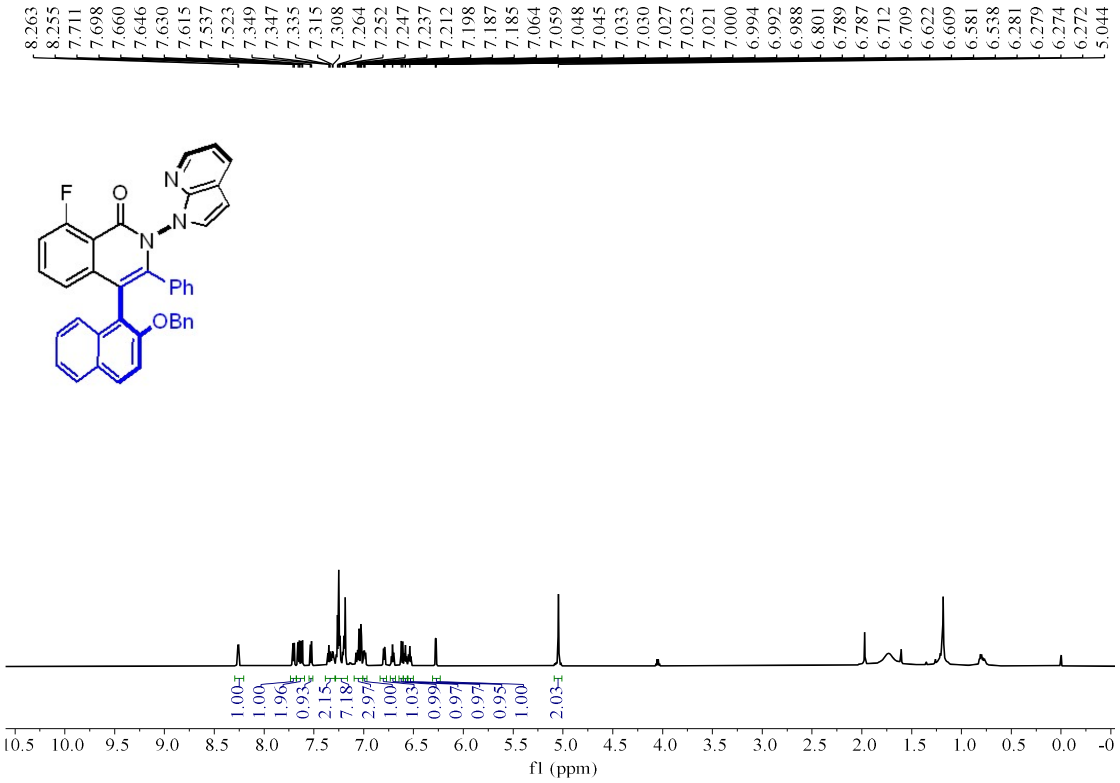


**Supplementary Figure 265.** $^{1}$**H NMR (600 MHz, CDCl**$_3$**) spectrum of 59.**

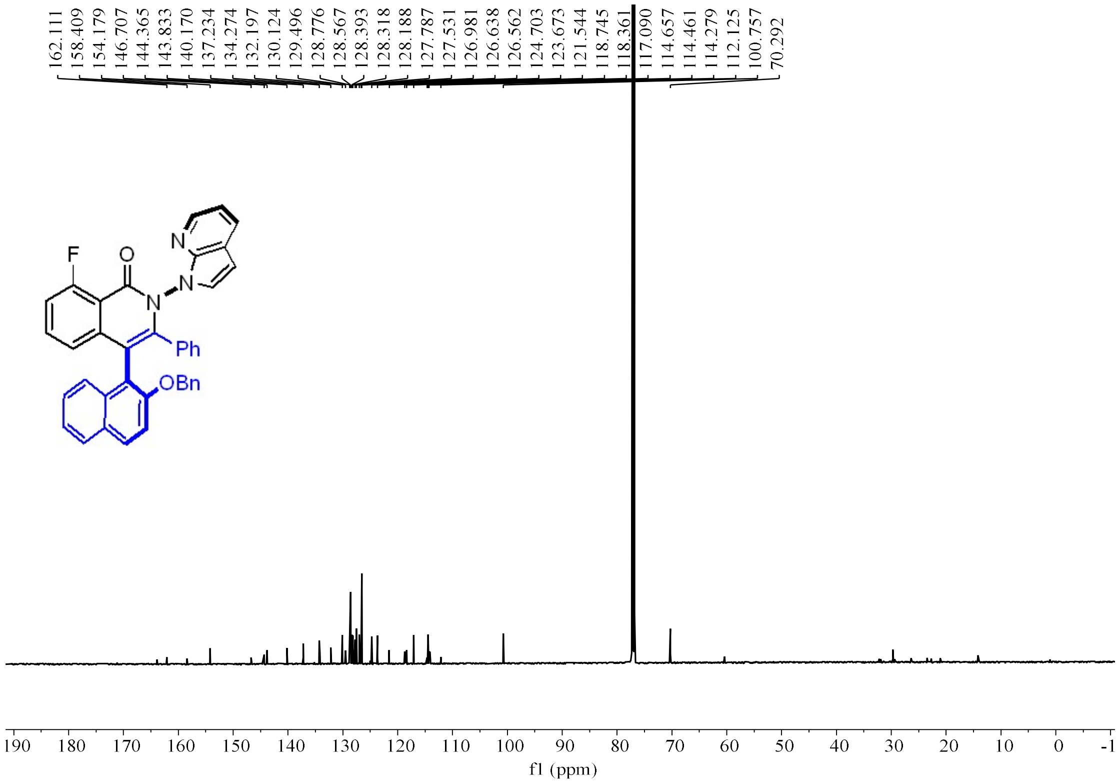


**Supplementary Figure 266.** $^{13}$**C NMR (150 MHz, CDCl**$_3$**) spectrum of 59.**

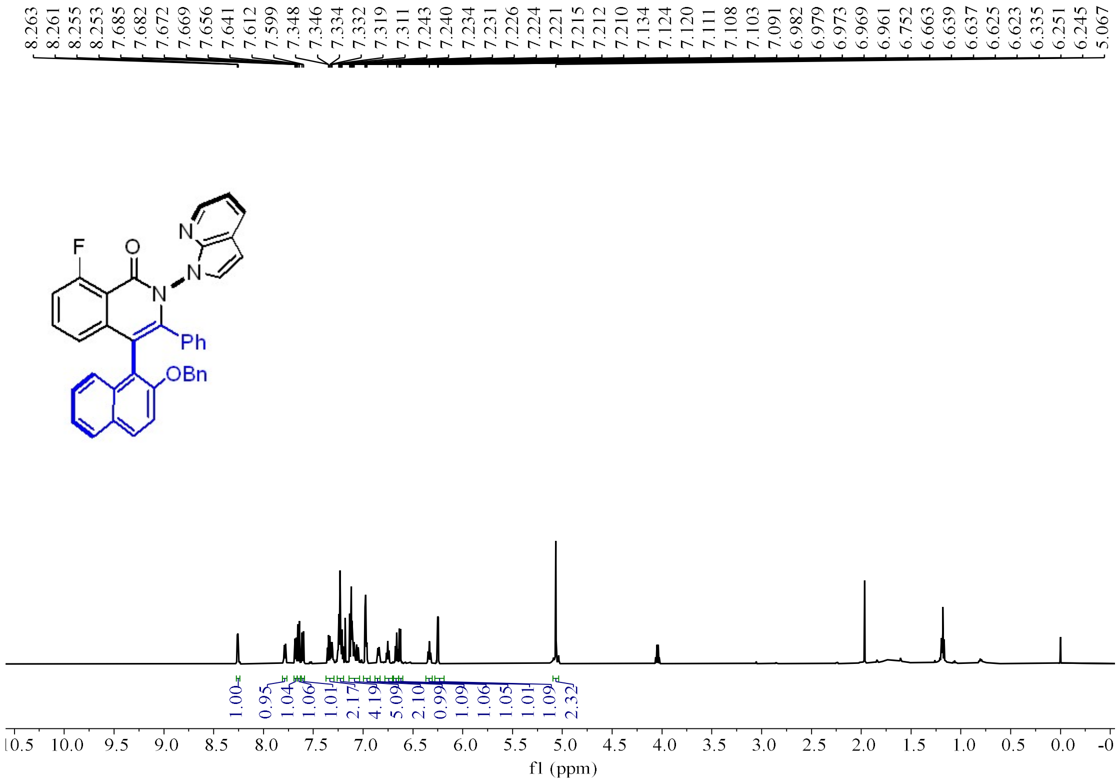


**Supplementary Figure 267. $^{1}$H NMR (600 MHz, $CDCl_3$) spectrum of dia-59.**

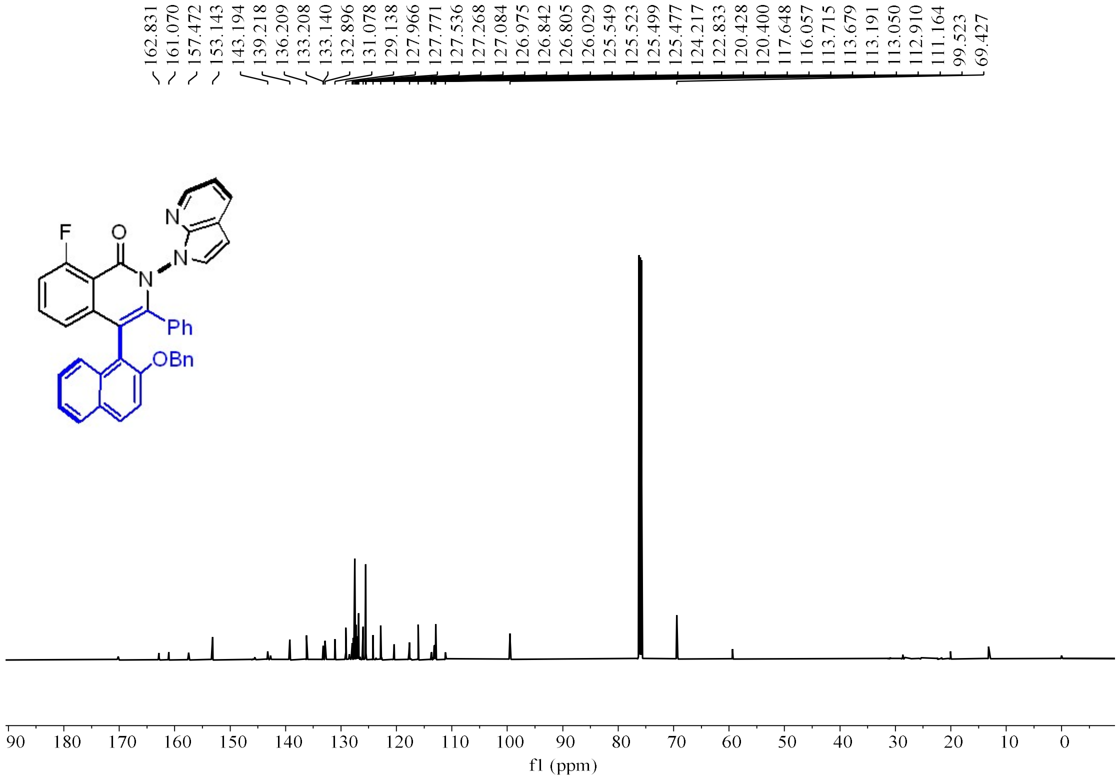


**Supplementary Figure 268. $^{13}$C NMR (150 MHz, $CDCl_3$) spectrum of dia-59.**

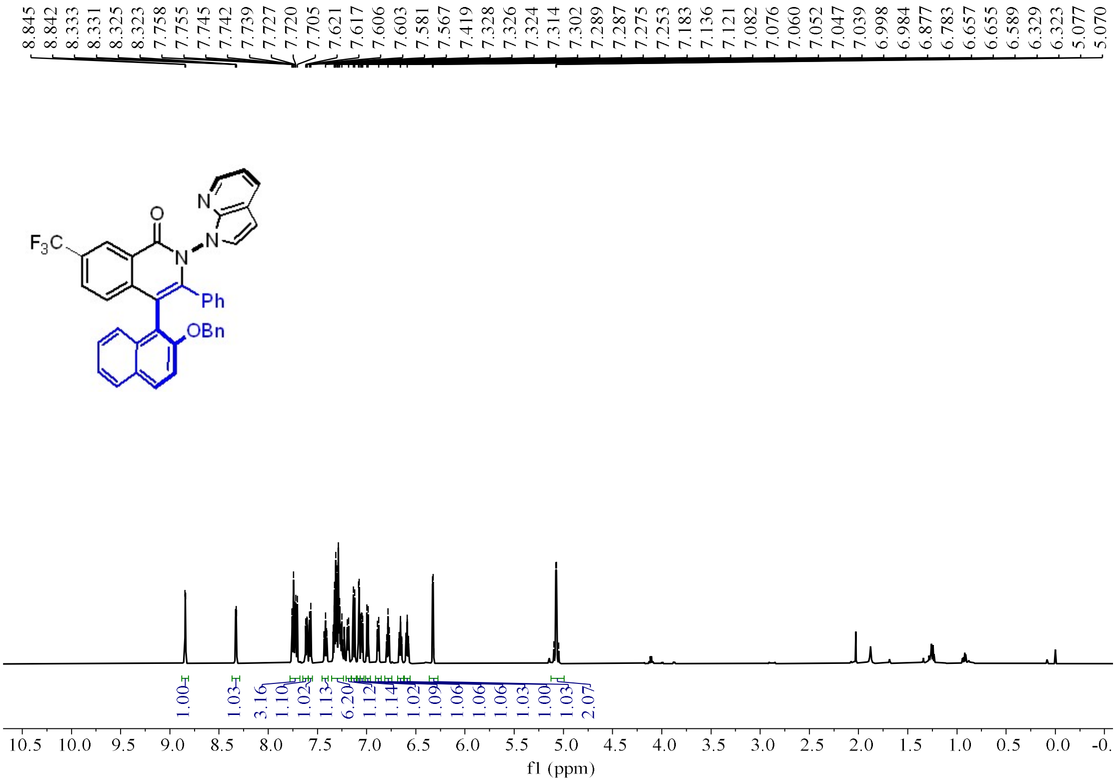


**Supplementary Figure 269.** $^{1}$**H NMR (600 MHz,** $CDCl_3$**) spectrum of 60.**

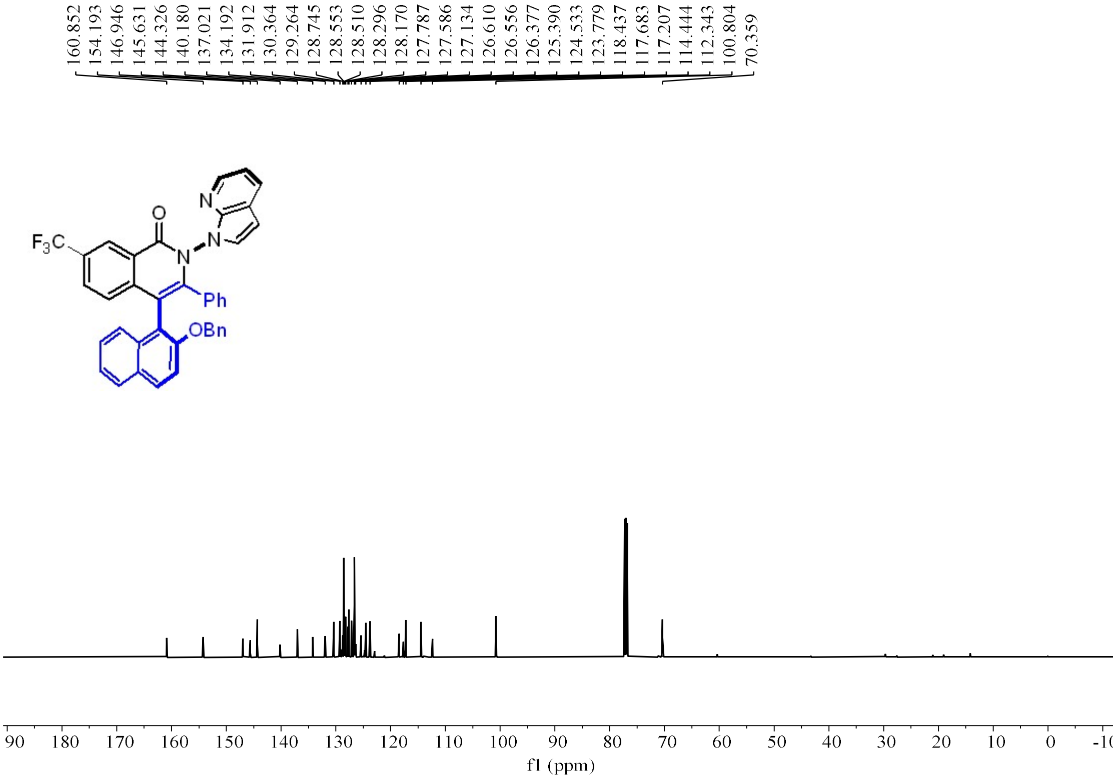


**Supplementary Figure 270.** $^{13}$**C NMR (150 MHz,** $CDCl_3$**) spectrum of 60.**

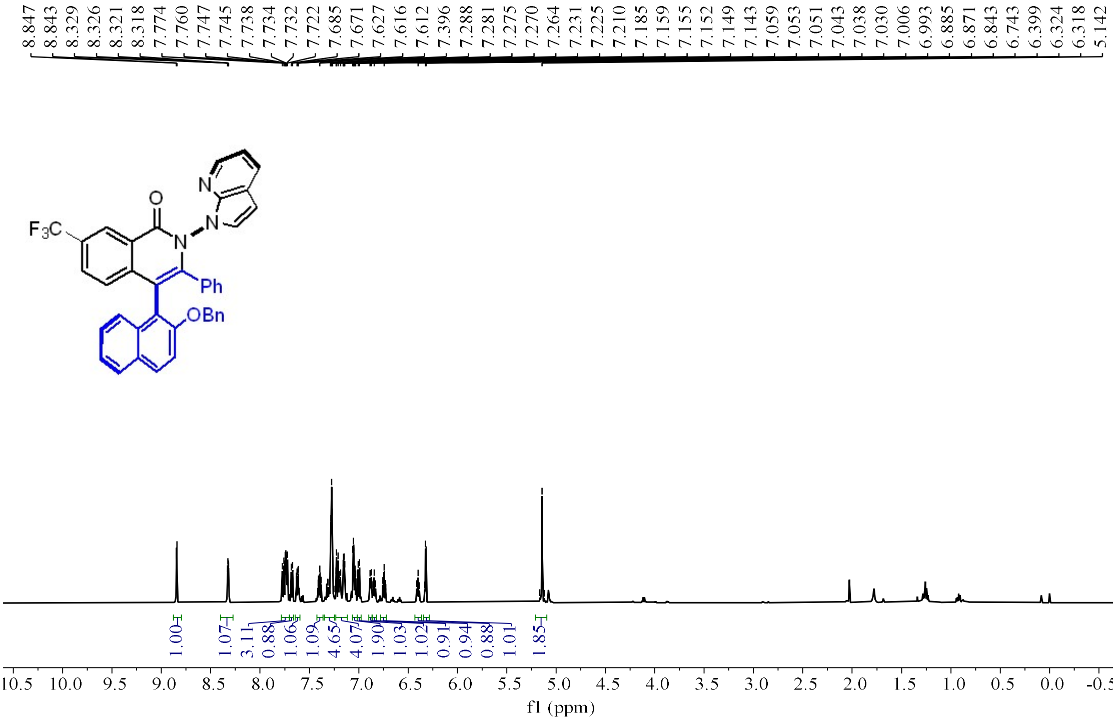


**Supplementary Figure 271.** $^{1}$**H NMR (600 MHz, CDCl₃) spectrum of dia-60.**

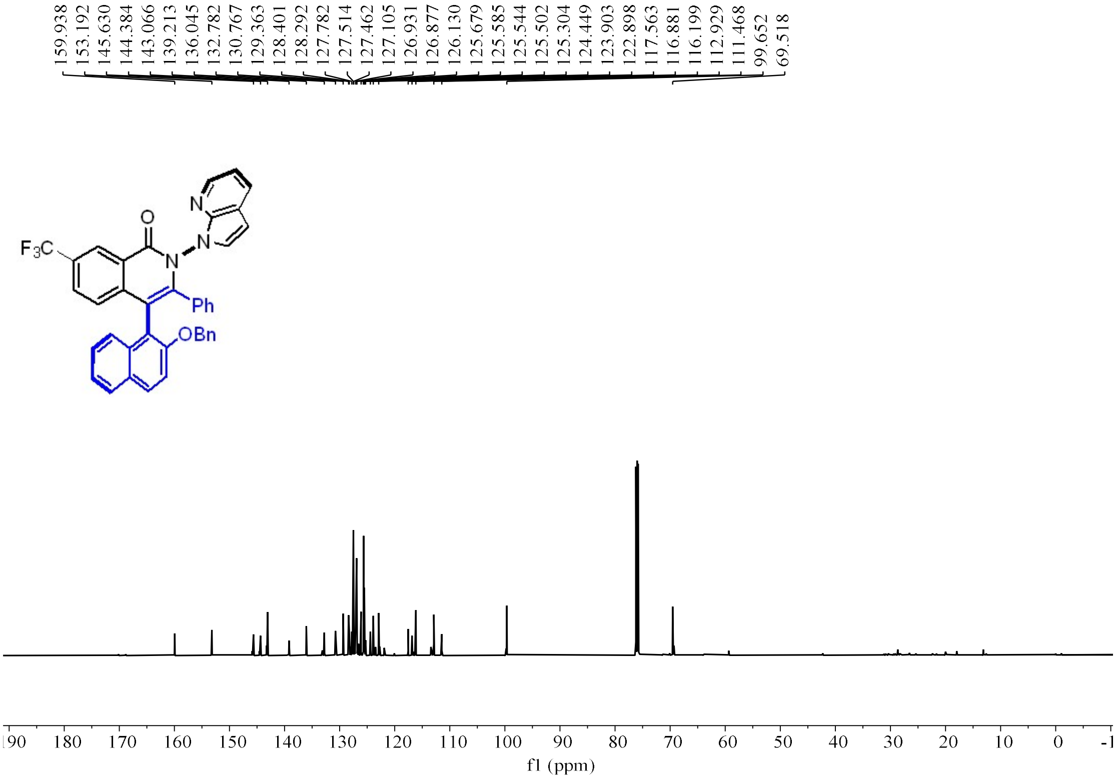


**Supplementary Figure 272.** $^{13}$**C NMR (150 MHz, CDCl₃) spectrum of dia-60.**

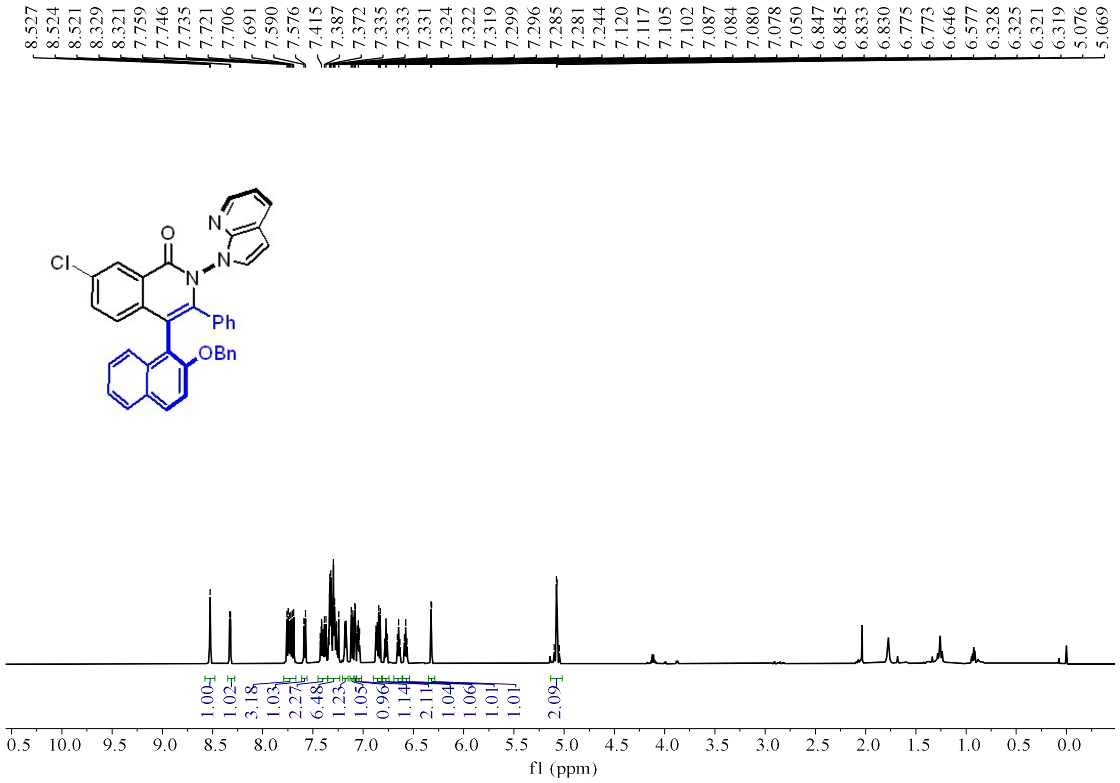


**Supplementary Figure 273.** $^{1}$**H NMR (600 MHz, CDCl$_3$) spectrum of 61.**

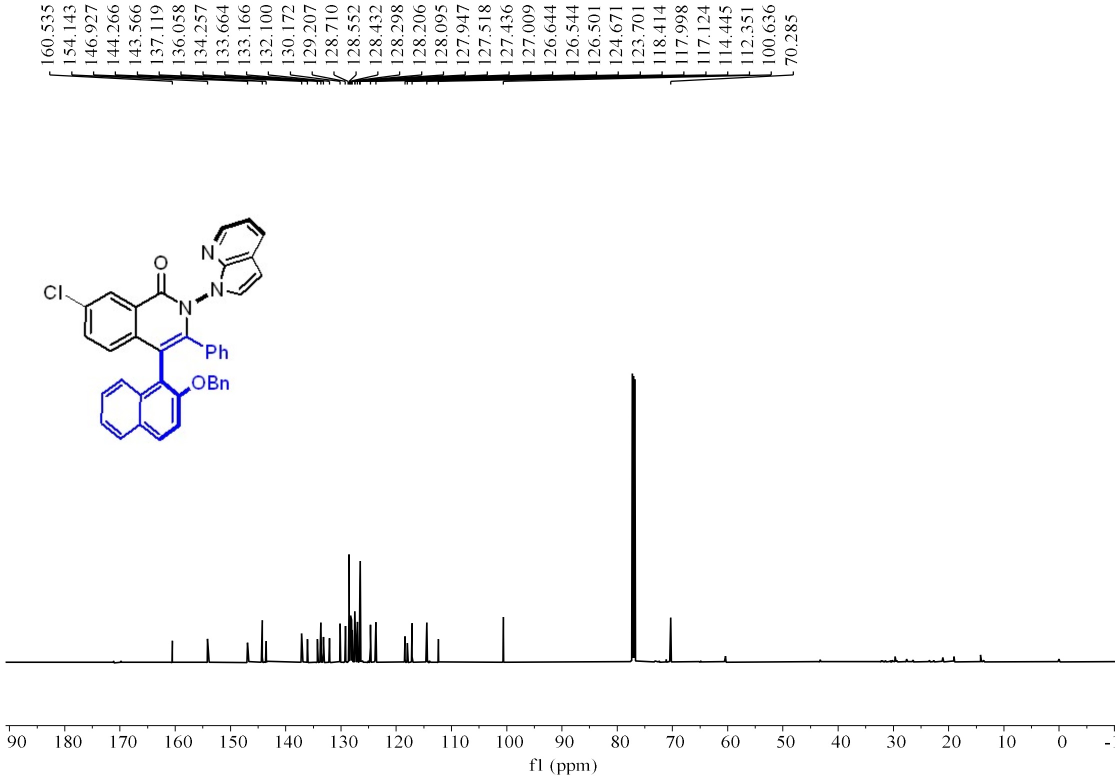


**Supplementary Figure 274.** $^{13}$**C NMR (150 MHz, CDCl$_3$) spectrum of 61.**

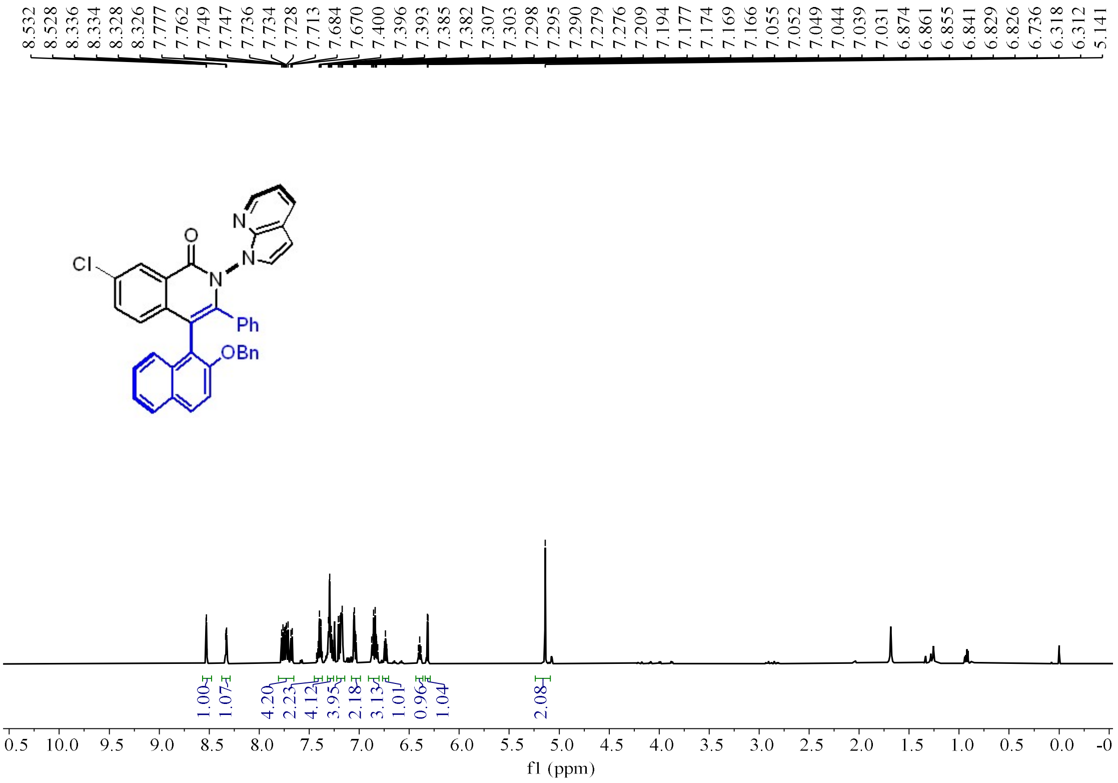


**Supplementary Figure 275.** **$^{1}$H NMR (600 MHz, CDCl$_3$) spectrum of dia-61.**

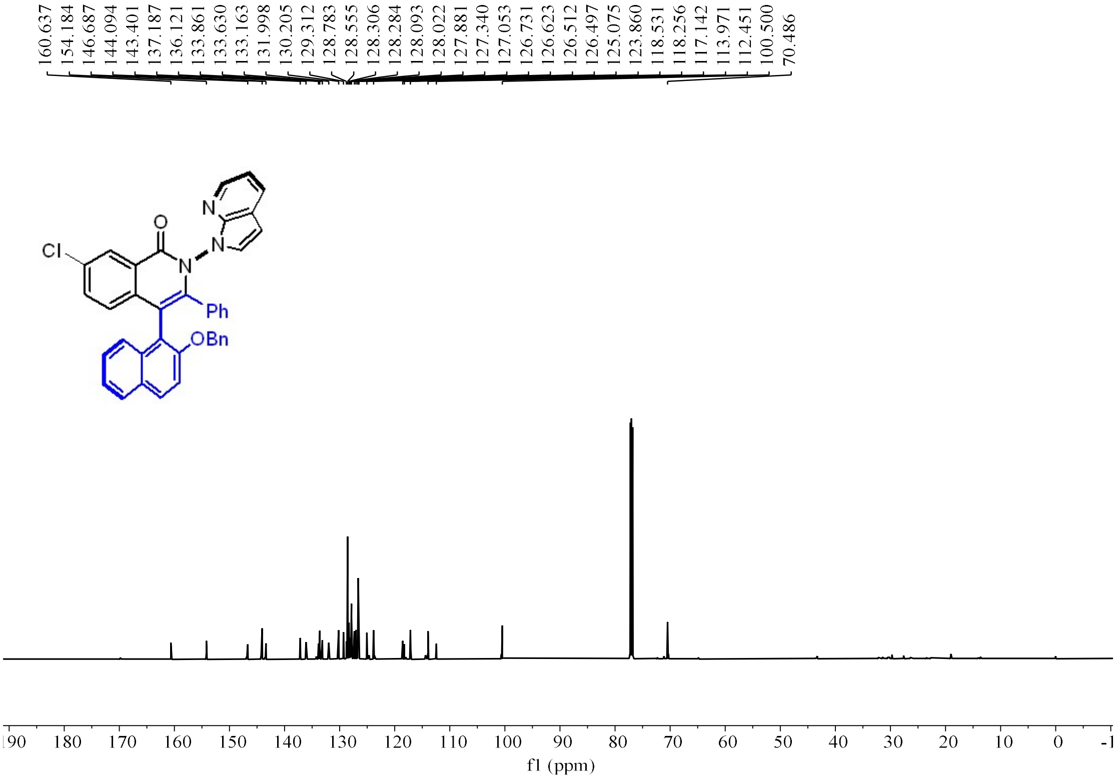


**Supplementary Figure 276.** **$^{13}$C NMR (150 MHz, CDCl$_3$) spectrum of dia-61.**

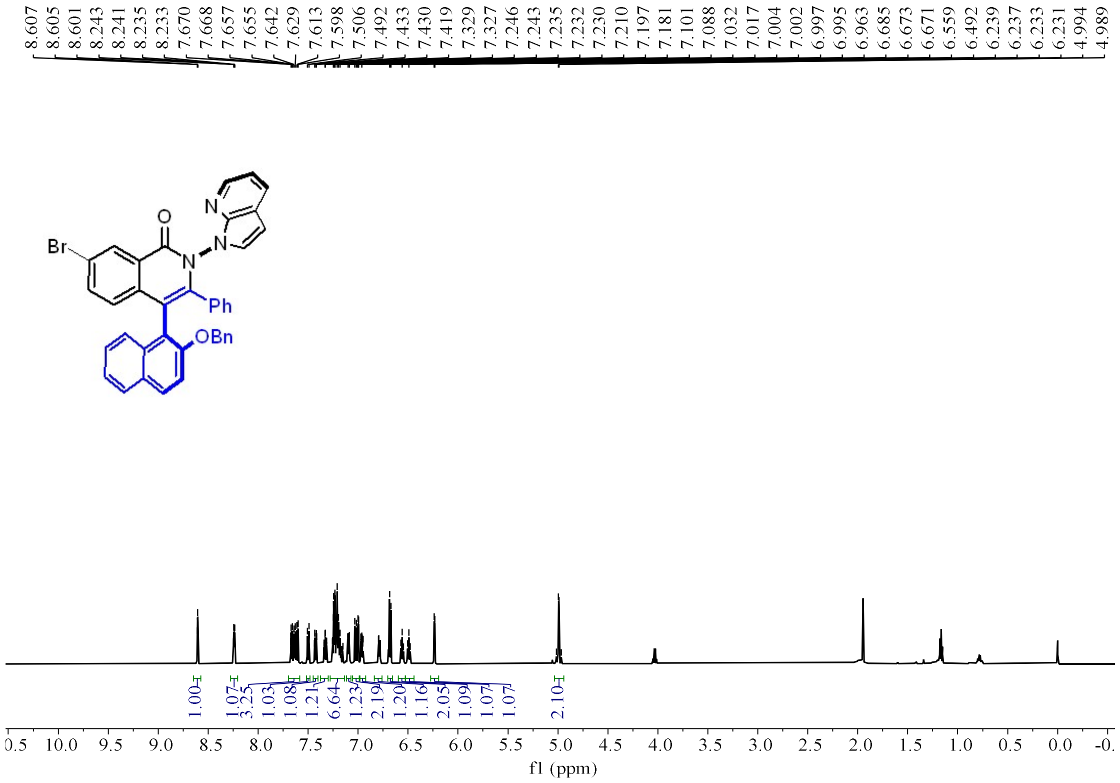


**Supplementary Figure 277.** **$^{1}$H NMR (600 MHz, $CDCl_3$) spectrum of 62.**

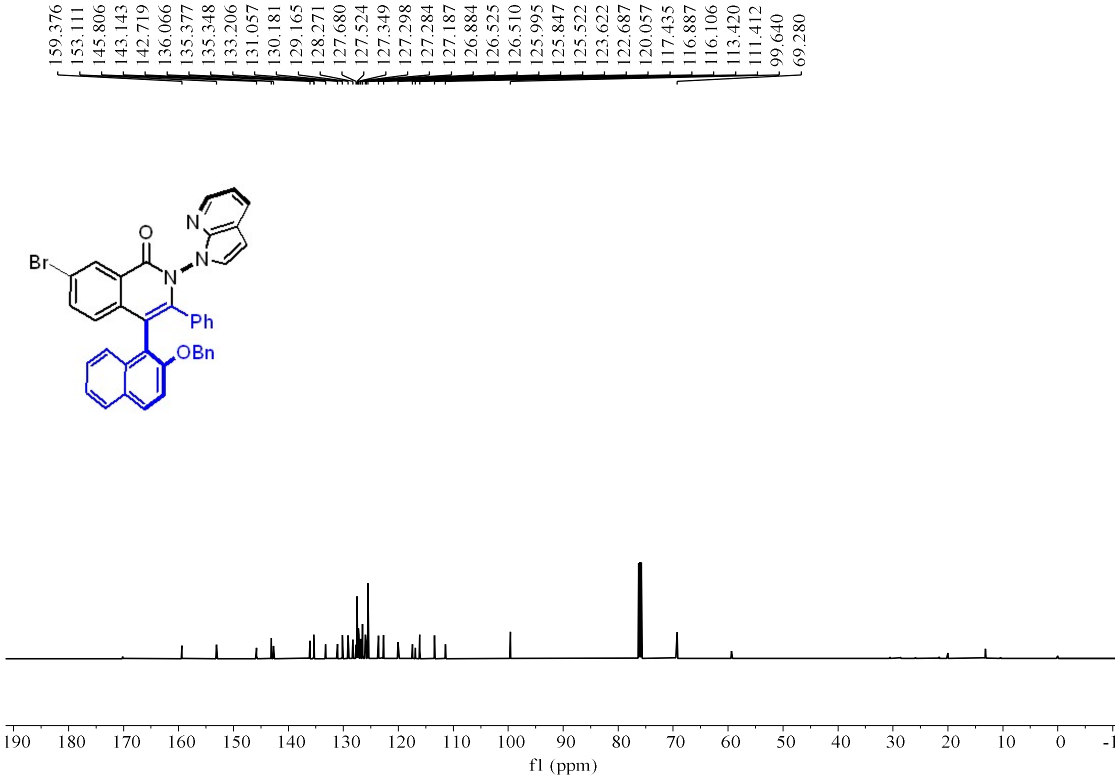


**Supplementary Figure 278.** **$^{13}$C NMR (150 MHz, $CDCl_3$) spectrum of 62.**

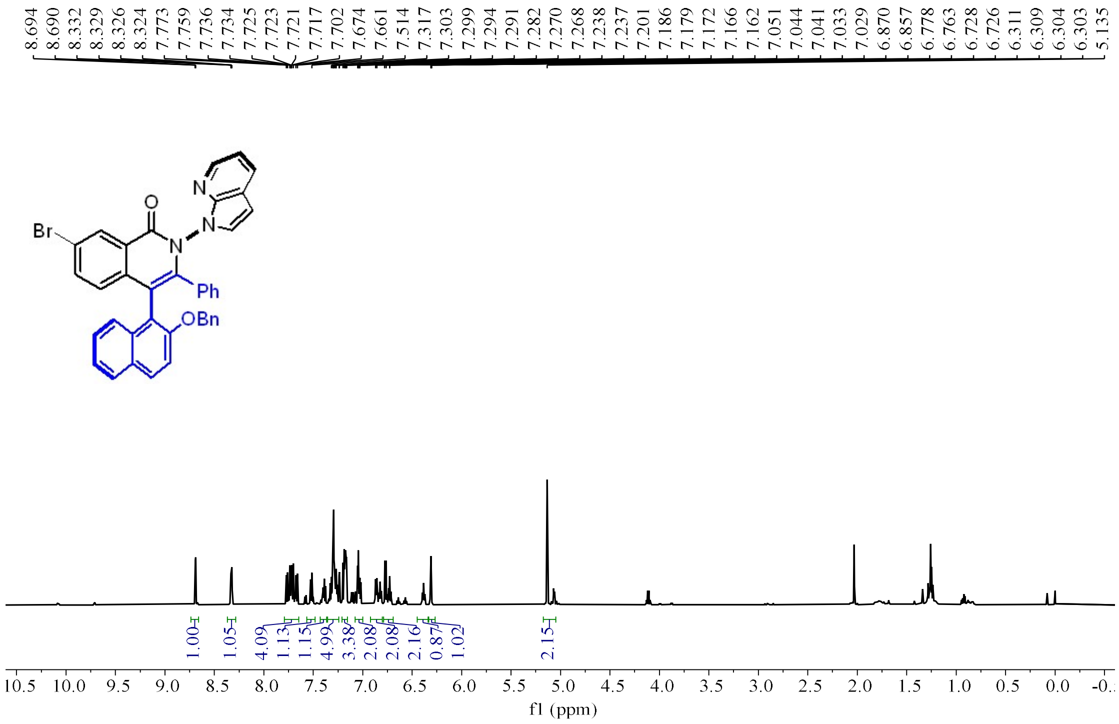


**Supplementary Figure 279.** $^{1}$**H NMR (600 MHz, CDCl₃) spectrum of dia-62.**

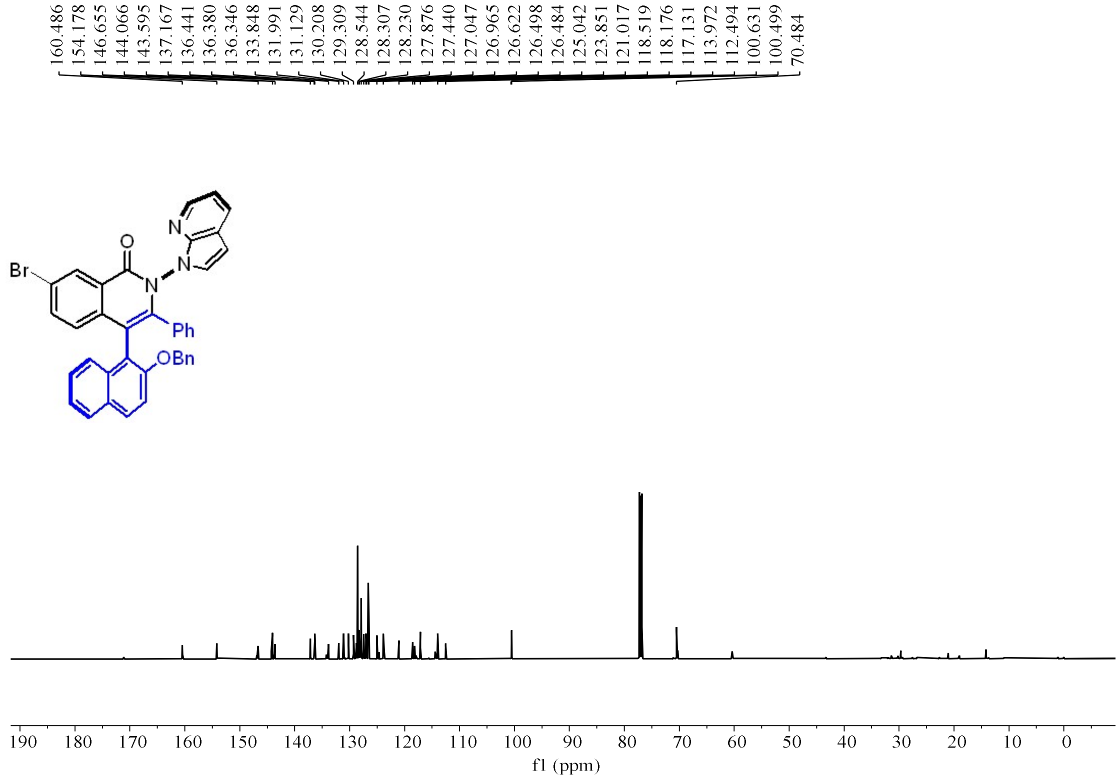


**Supplementary Figure 280.** $^{13}$**C NMR (150 MHz, CDCl₃) spectrum of dia-62.**

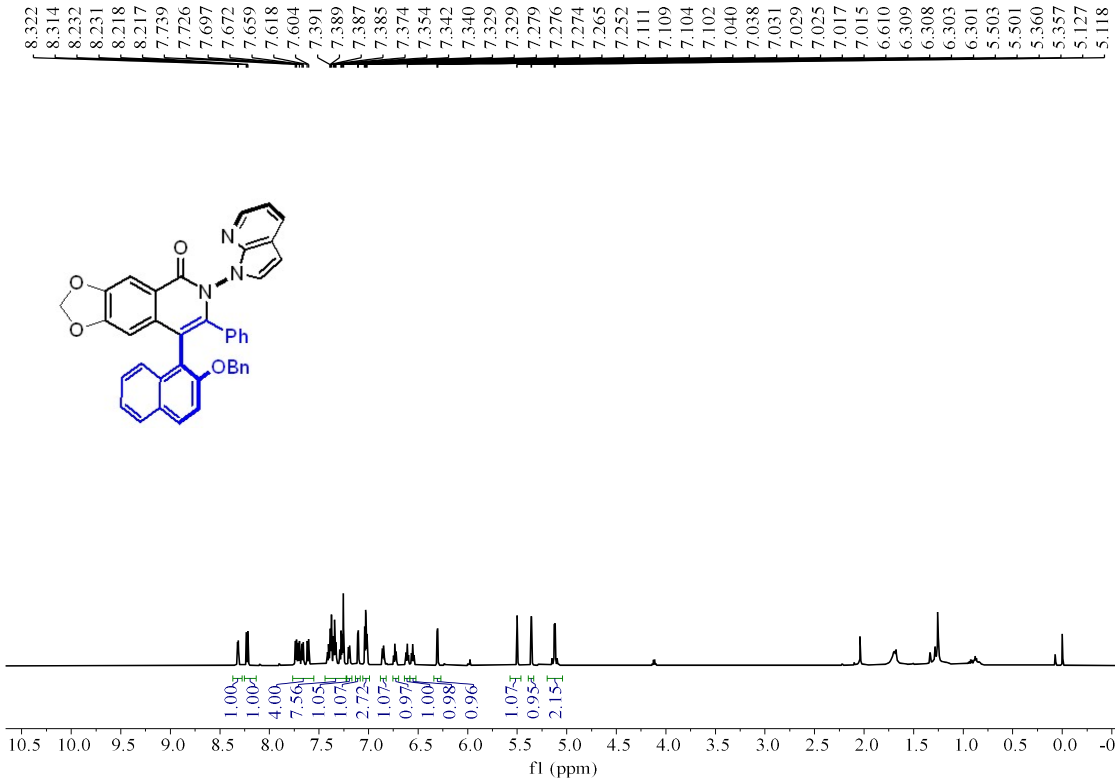

**Supplementary Figure 281.** **$^{1}$H NMR (600 MHz, CDCl$_3$) spectrum of 63.**

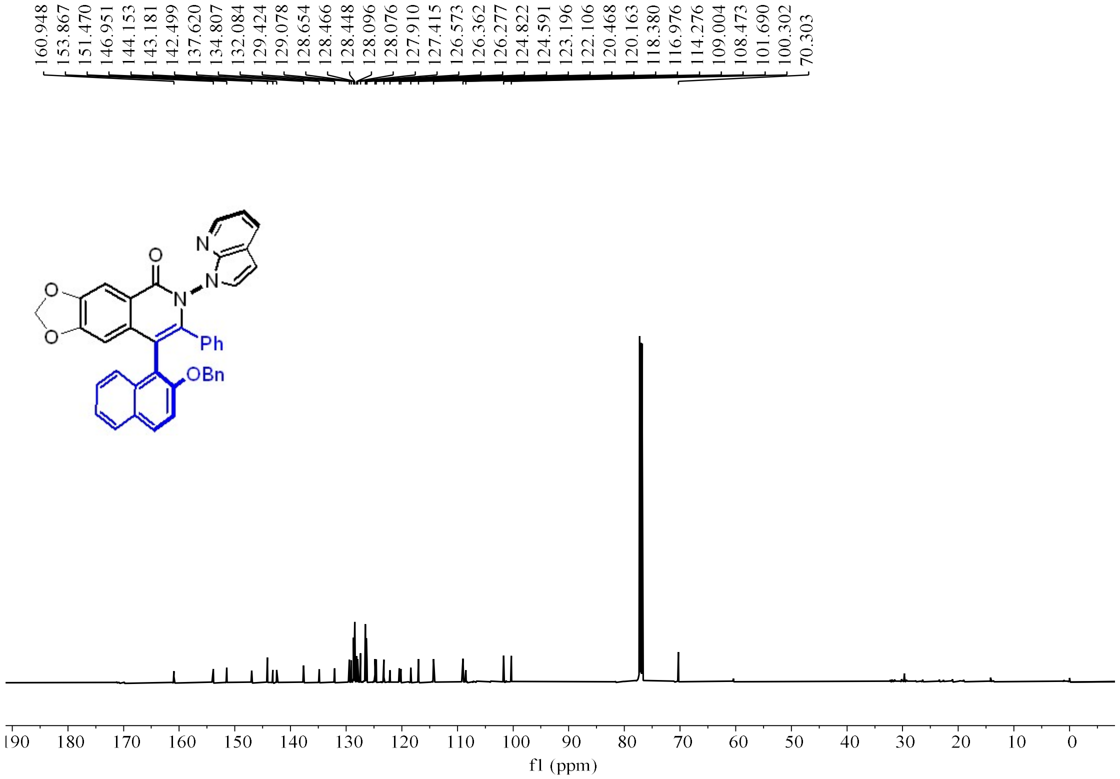

**Supplementary Figure 282.** **$^{13}$C NMR (150 MHz, CDCl$_3$) spectrum of 63.**

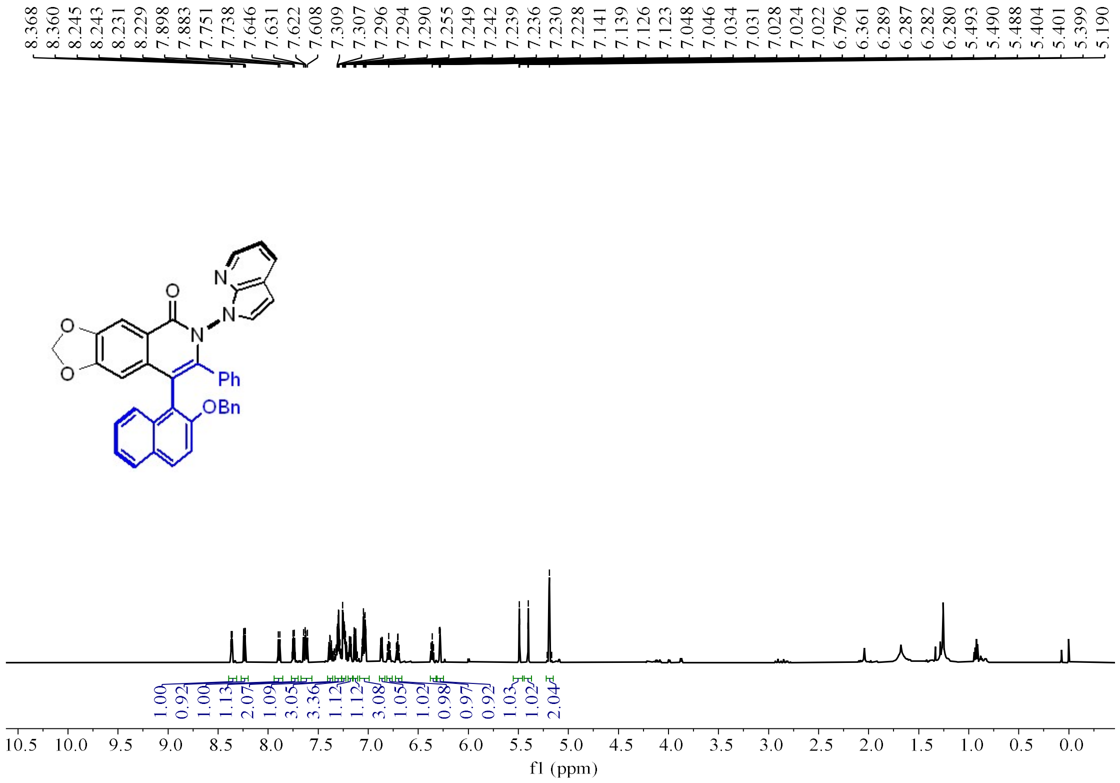


**Supplementary Figure 283.** **$^{1}$H NMR (600 MHz, $CDCl_3$) spectrum of dia-63.**

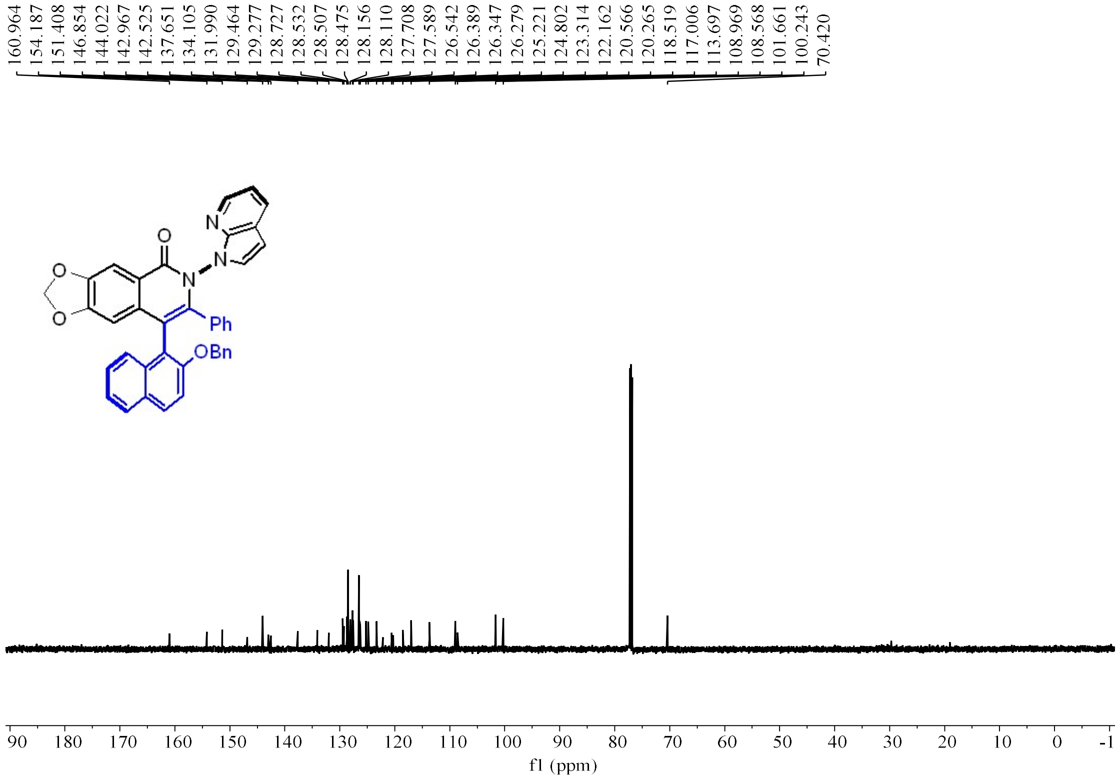


**Supplementary Figure 284.** **$^{13}$C NMR (150 MHz, $CDCl_3$) spectrum of dia-63.**

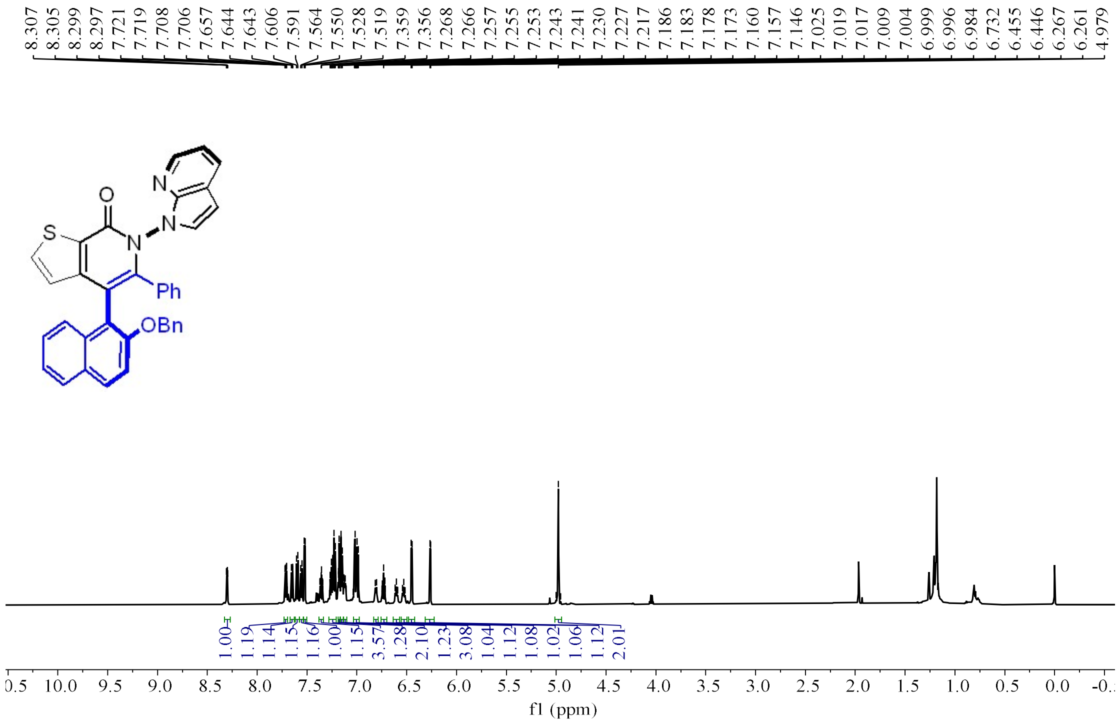

**Supplementary Figure 285. $^{1}$H NMR (600 MHz, $CDCl_3$) spectrum of 64.**

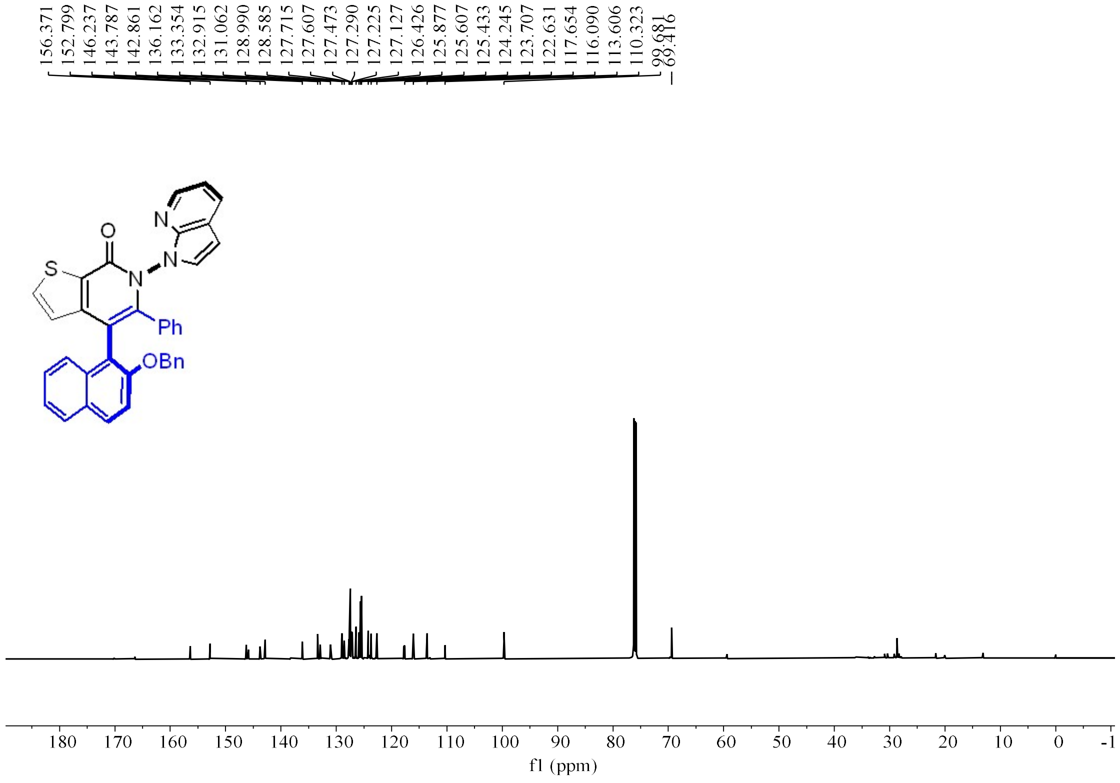

**Supplementary Figure 286. $^{13}$C NMR (150 MHz, $CDCl_3$) spectrum of 64.**

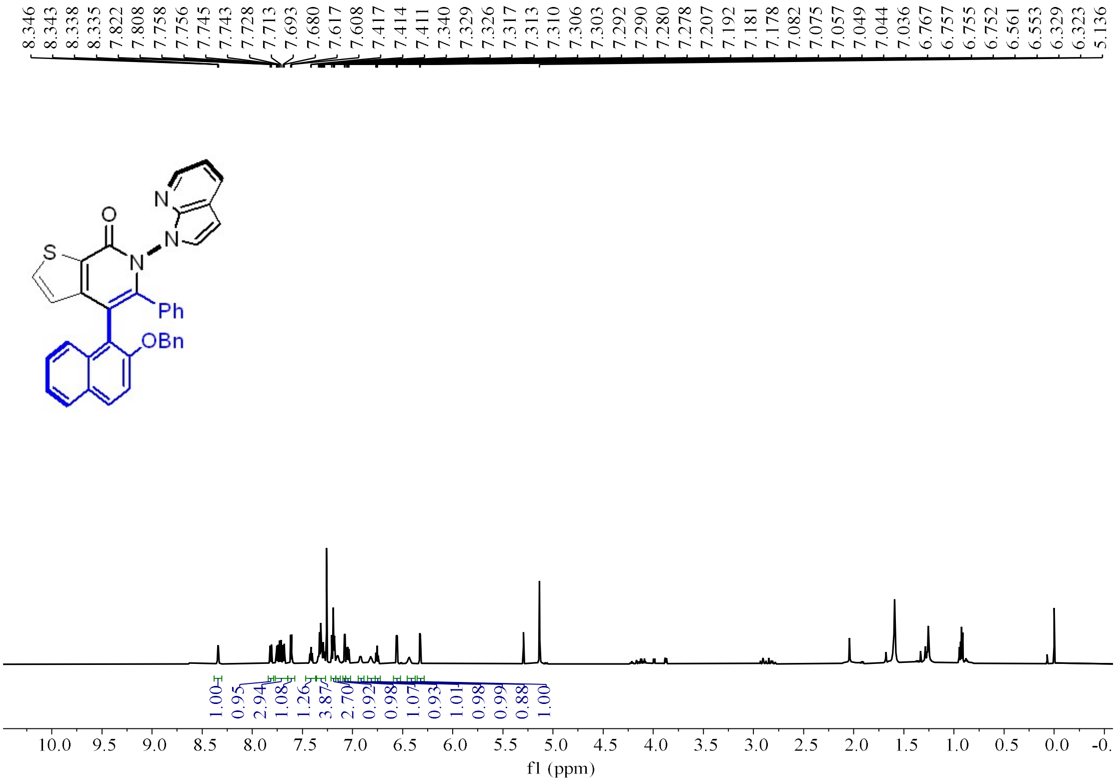


**Supplementary Figure 287.** $^{1}$**H NMR (600 MHz, CDCl**$_3$**) spectrum of dia-64.**

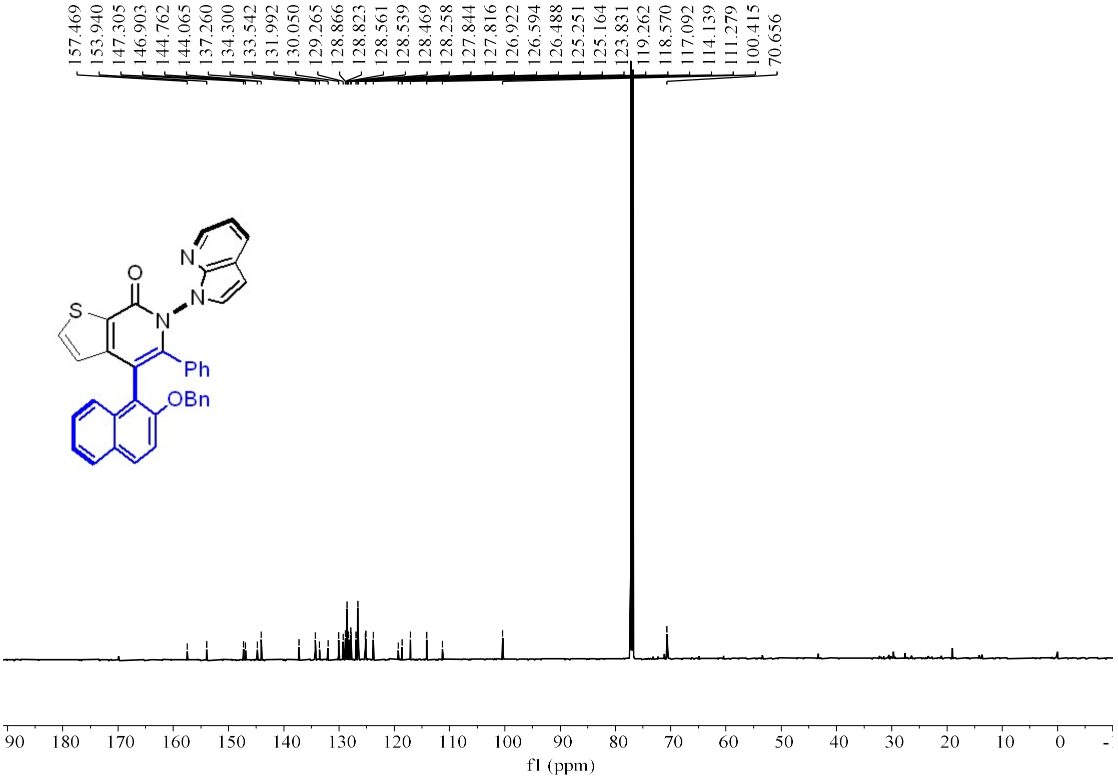


**Supplementary Figure 288.** $^{13}$**C NMR (150 MHz, CDCl**$_3$**) spectrum of dia-64.**

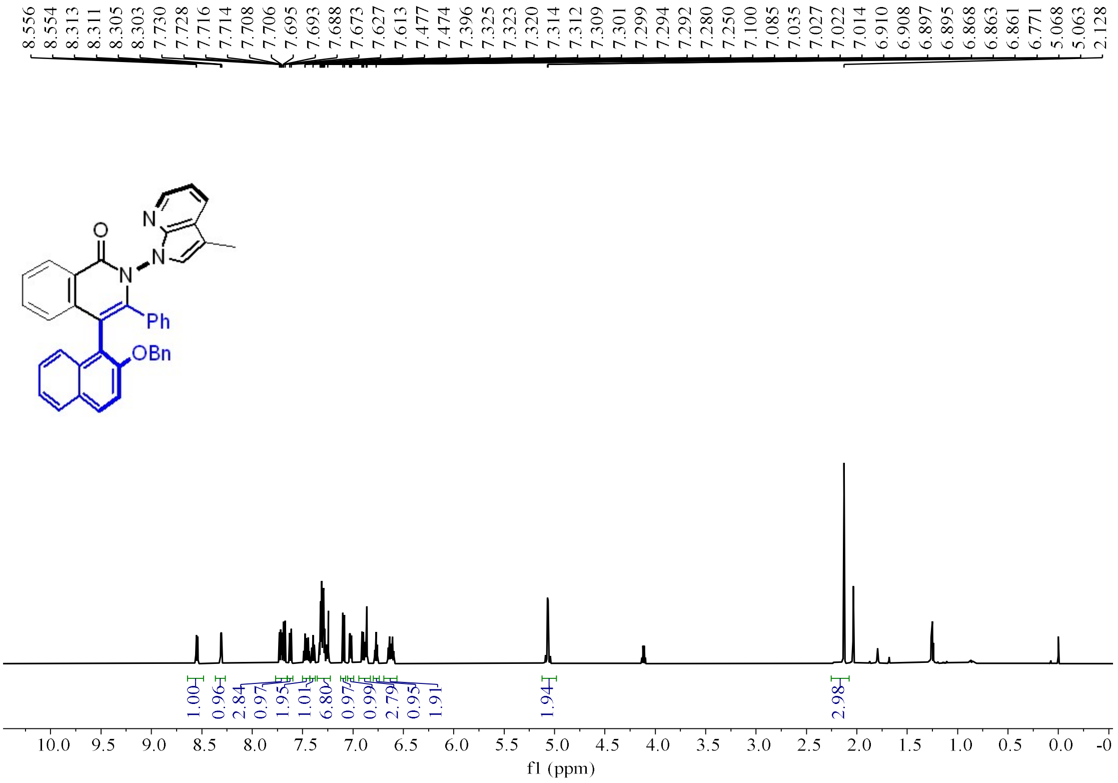


**Supplementary Figure 289.** $^{1}$**H NMR (600 MHz, CDCl$_3$) spectrum of 65.**

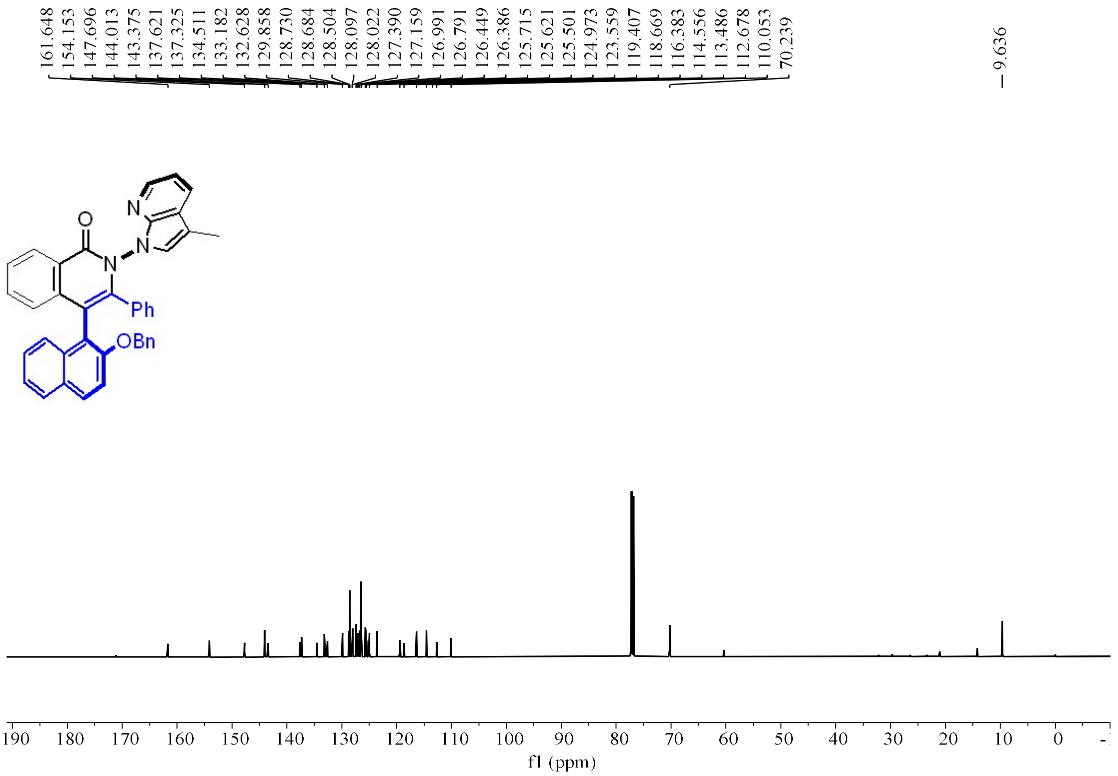


**Supplementary Figure 290.** $^{13}$**C NMR (150 MHz, CDCl$_3$) spectrum of 65.**

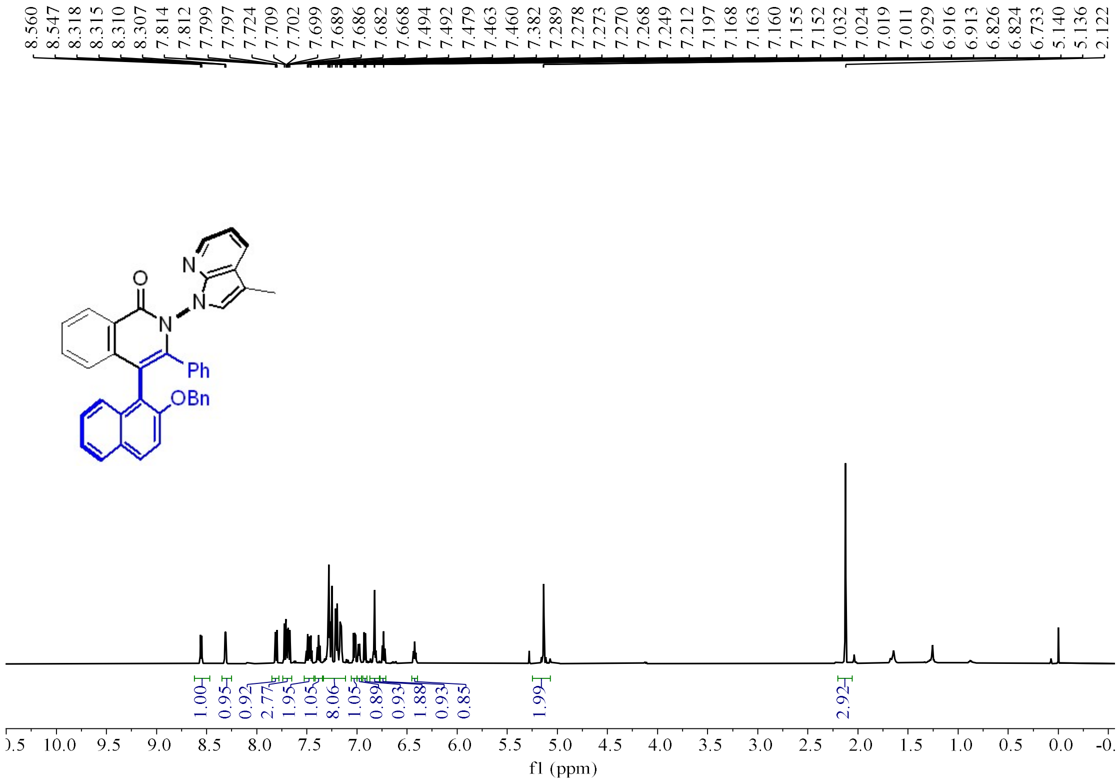


**Supplementary Figure 291.** $^{1}$**H NMR (600 MHz, CDCl$_3$) spectrum of dia-65.**

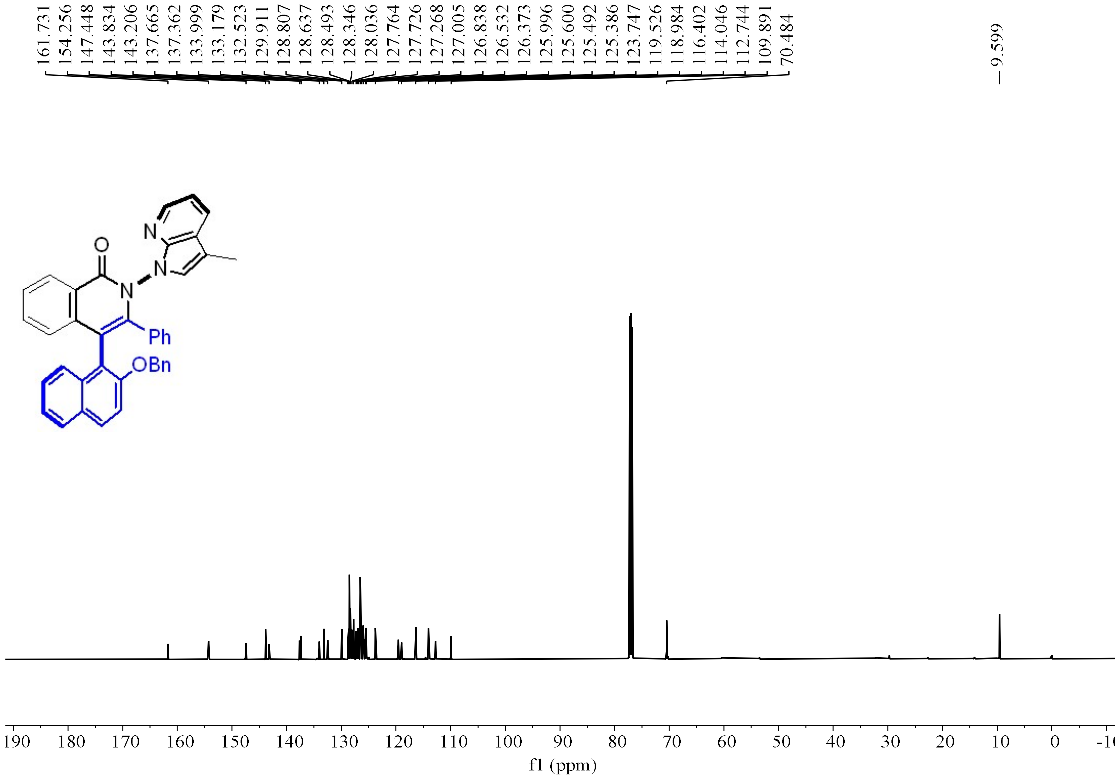


**Supplementary Figure 292.** $^{13}$**C NMR (150 MHz, CDCl$_3$) spectrum of dia-65.**

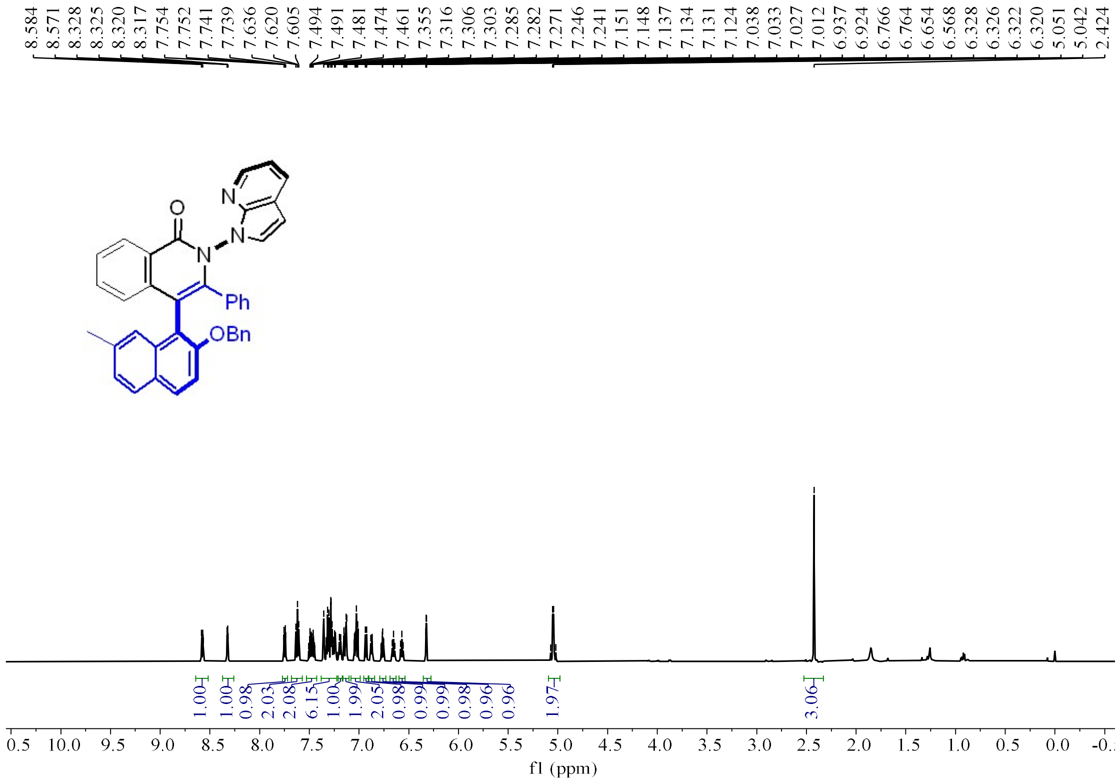


**Supplementary Figure 293.** $^{1}H$ **NMR (600 MHz, $CDCl_3$) spectrum of 66.**

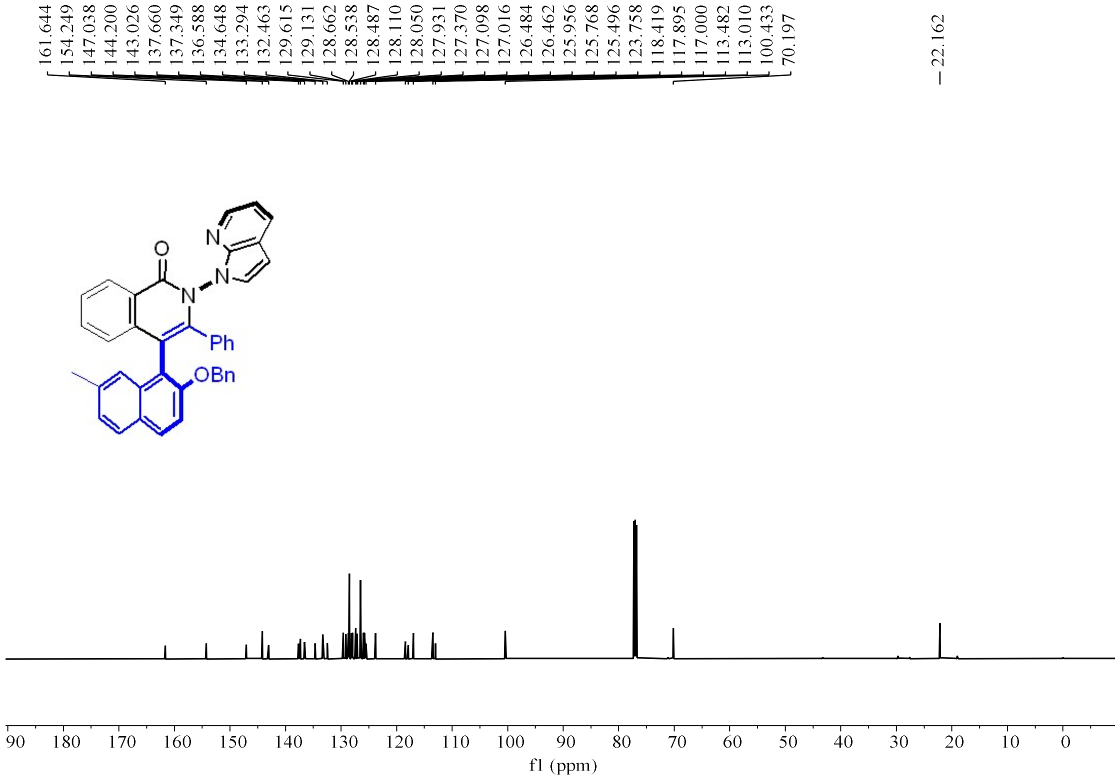


**Supplementary Figure 294.** $^{13}C$ **NMR (150 MHz, $CDCl_3$) spectrum of 66.**

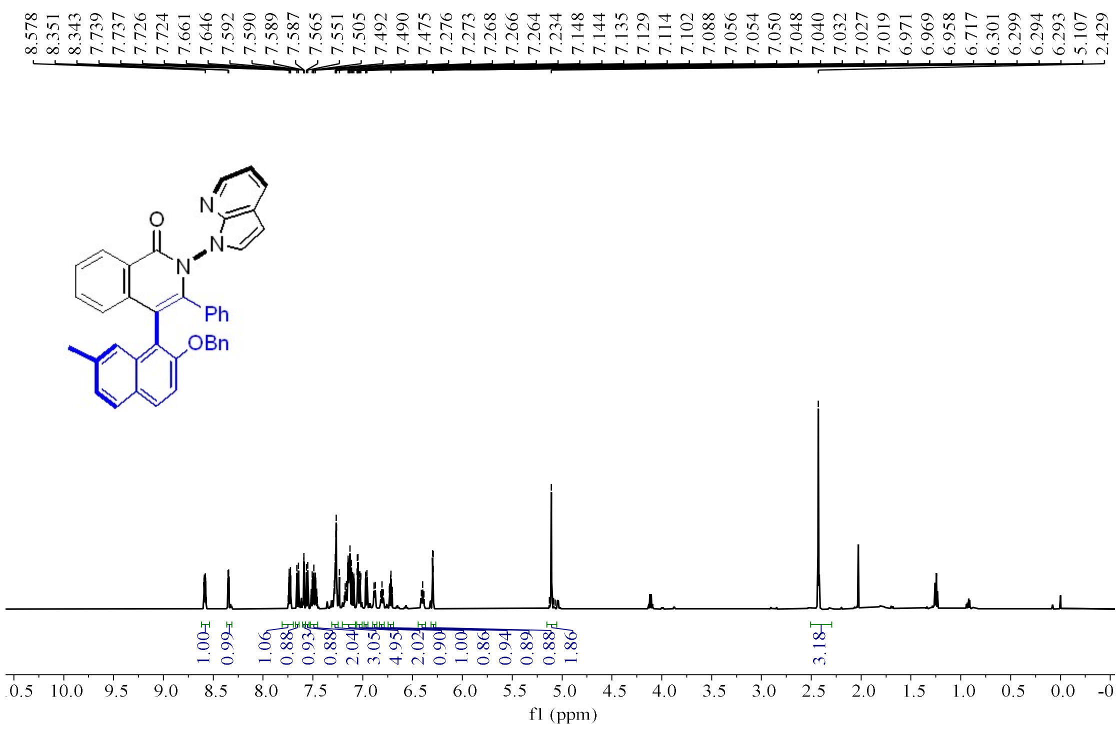


**Supplementary Figure 295.** **$^{1}$H NMR (600 MHz, CDCl$_3$) spectrum of dia-66.**

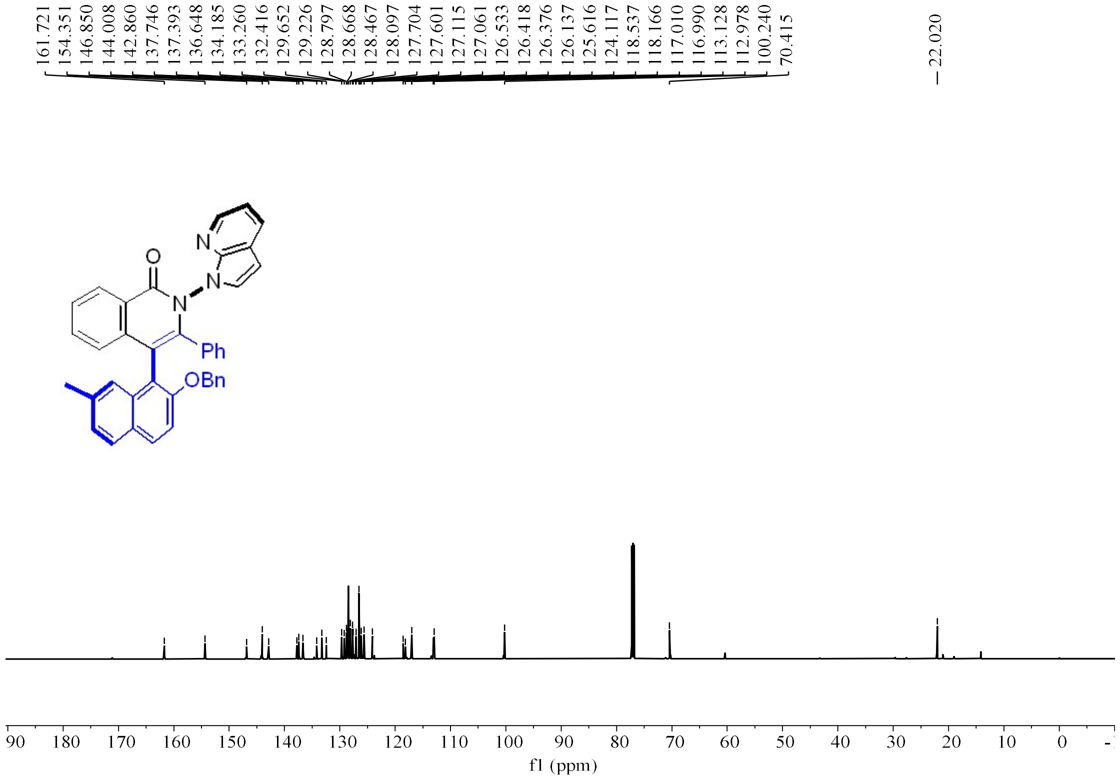


**Supplementary Figure 296.** **$^{13}$C NMR (150 MHz, CDCl$_3$) spectrum of dia-66.**

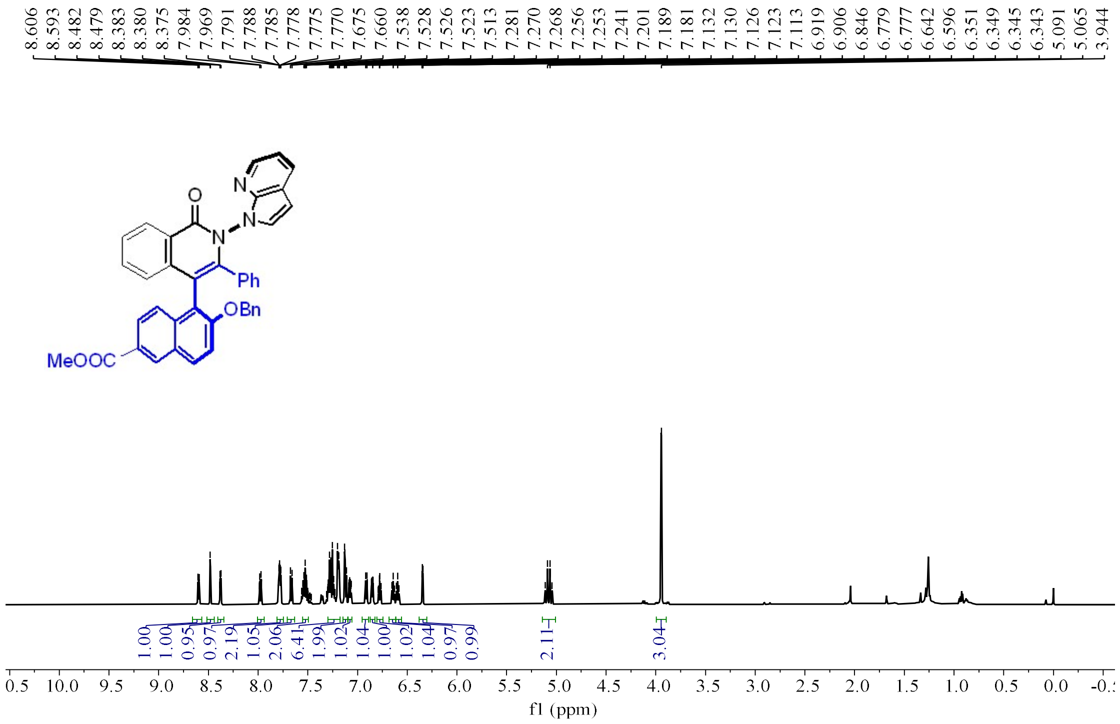


**Supplementary Figure 297.** $^{1}$**H NMR (600 MHz,** $CDCl_3$**) spectrum of 67.**

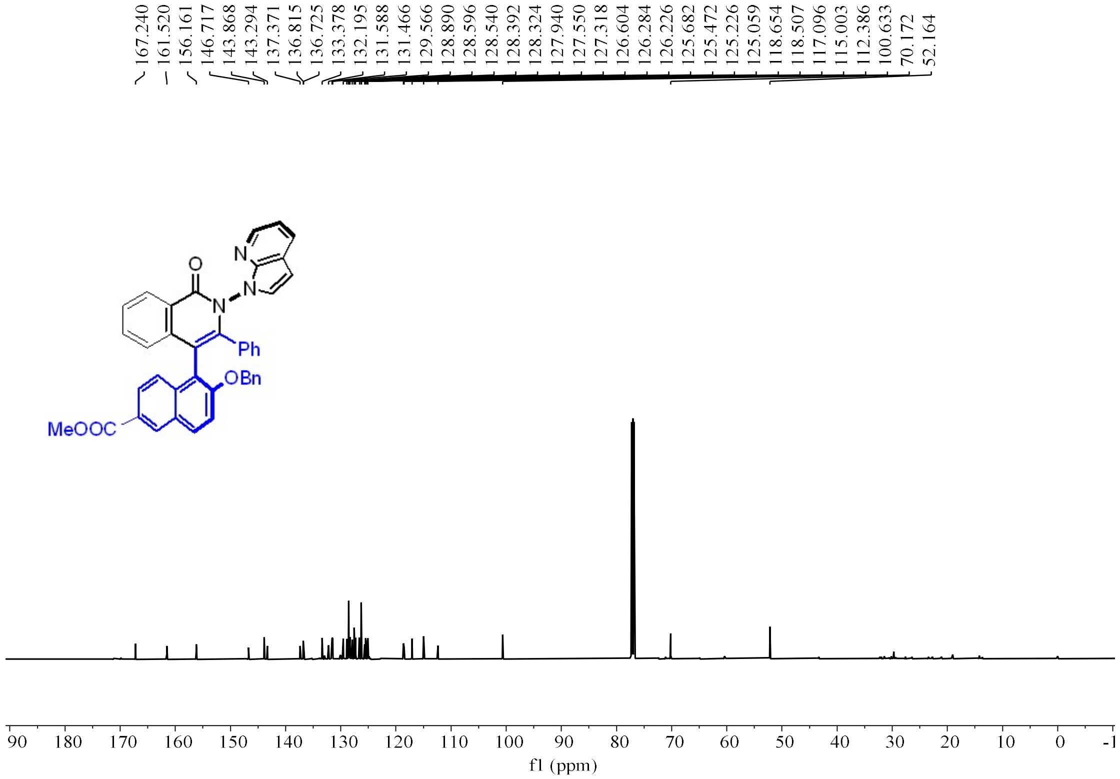


**Supplementary Figure 298.** $^{13}$**C NMR (150 MHz,** $CDCl_3$**) spectrum of 67.**

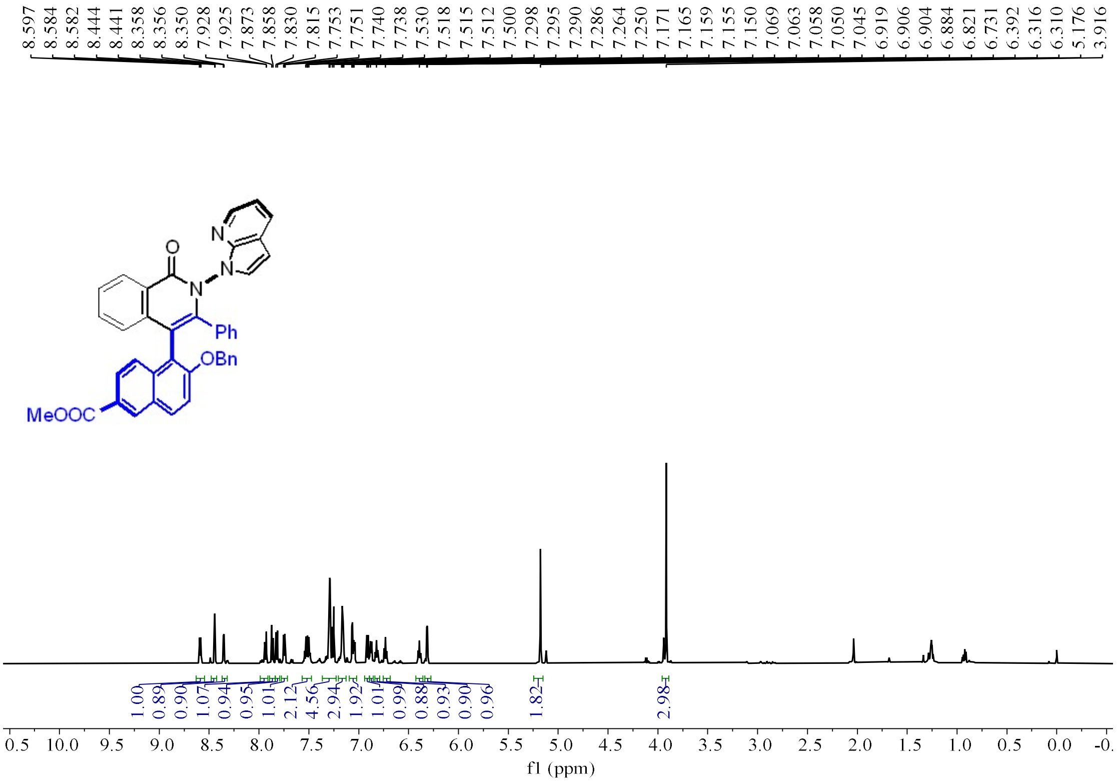


**Supplementary Figure 299. $^{1}$H NMR (600 MHz, $CDCl_3$) spectrum of dia-67.**

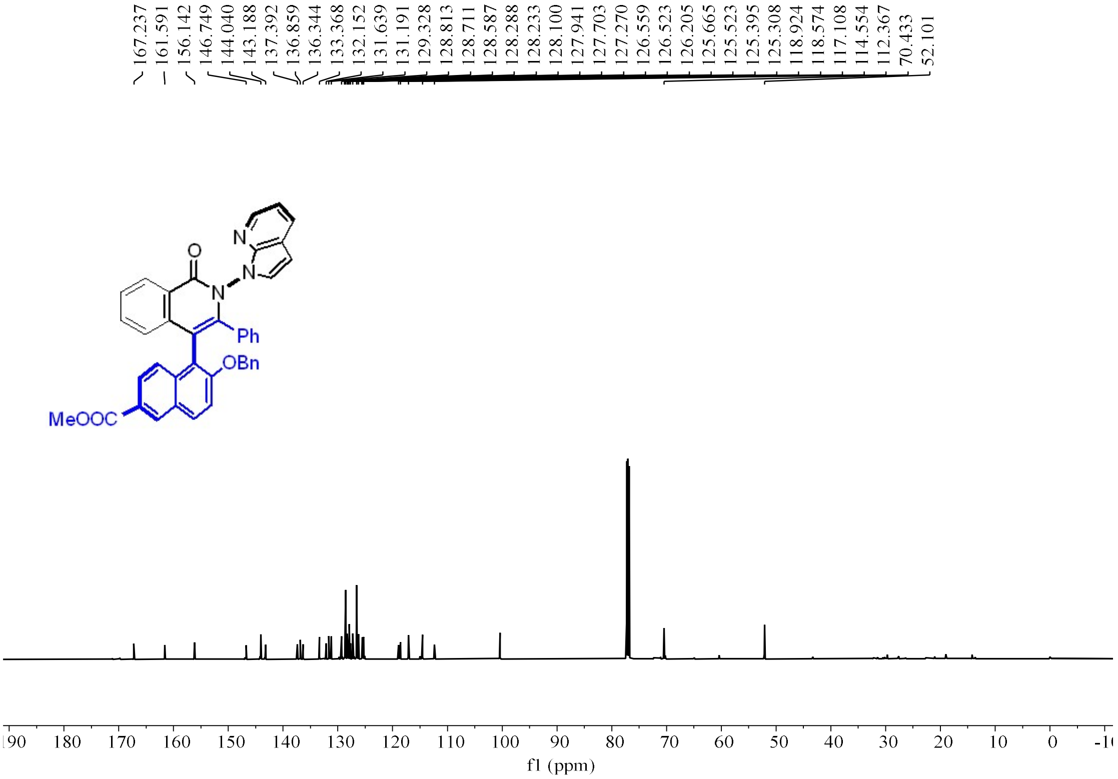


**Supplementary Figure 300. $^{13}$C NMR (150 MHz, $CDCl_3$) spectrum of dia-67.**

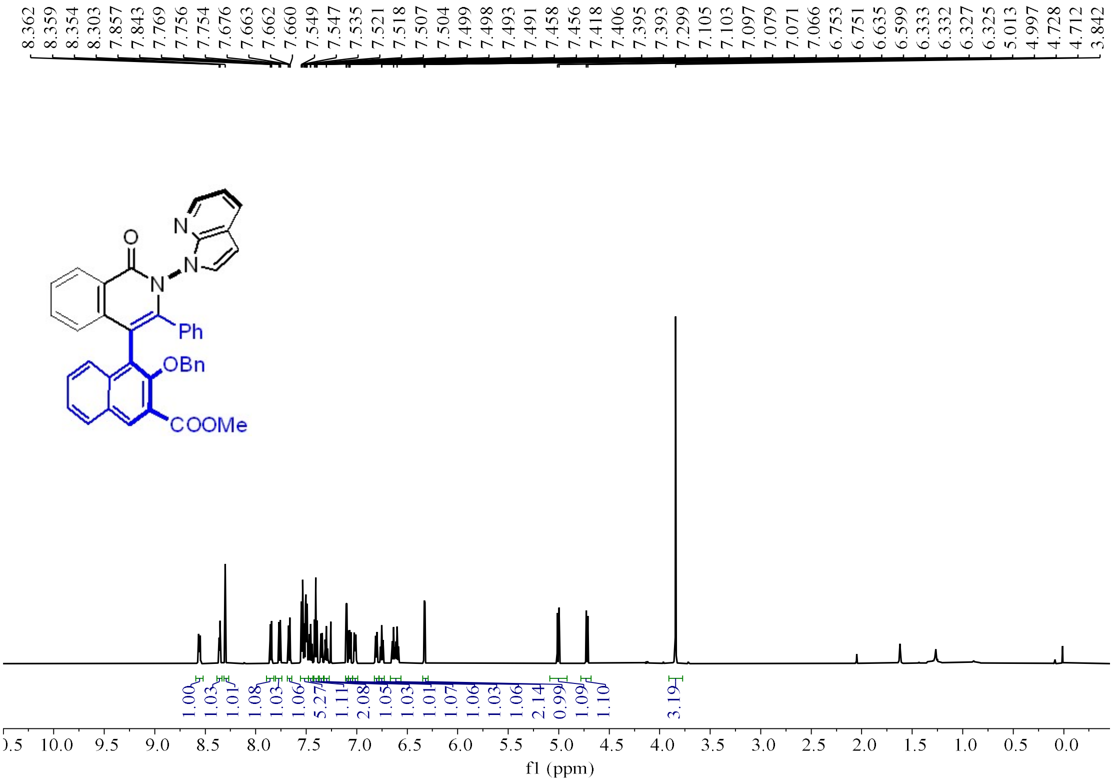


**Supplementary Figure 301. $^{1}H$ NMR (600 MHz, $CDCl_3$) spectrum of 68.**

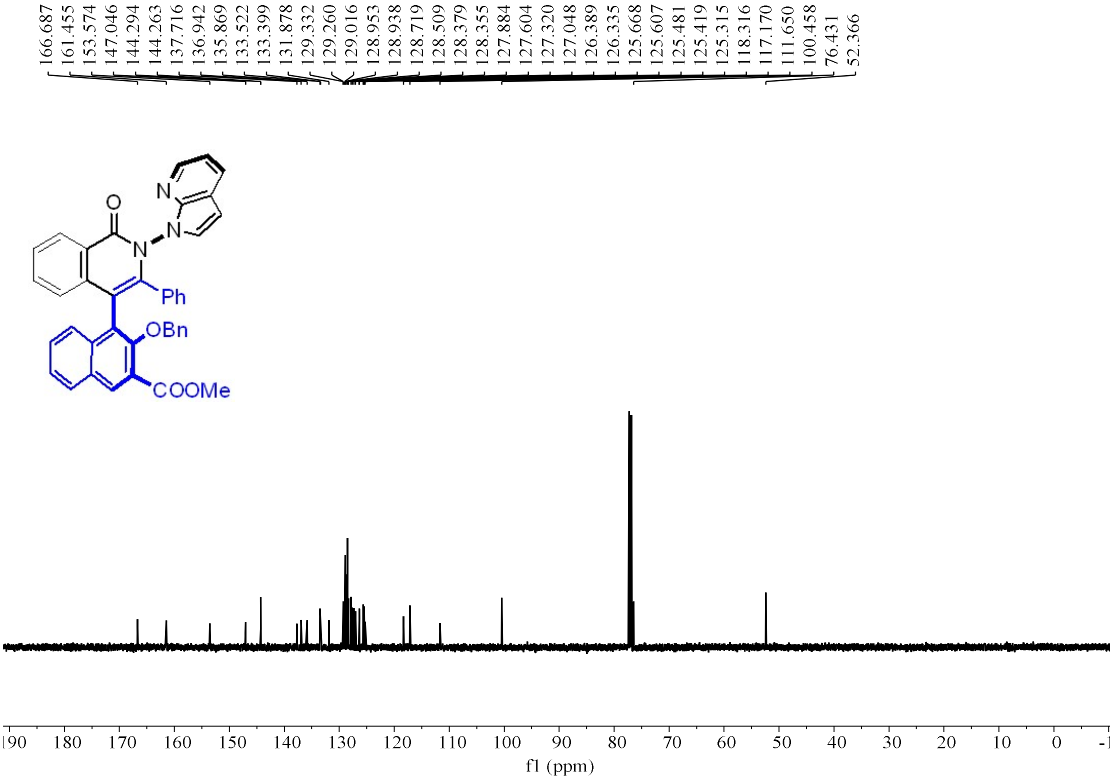


**Supplementary Figure 302. $^{13}C$ NMR (150 MHz, $CDCl_3$) spectrum of 68.**

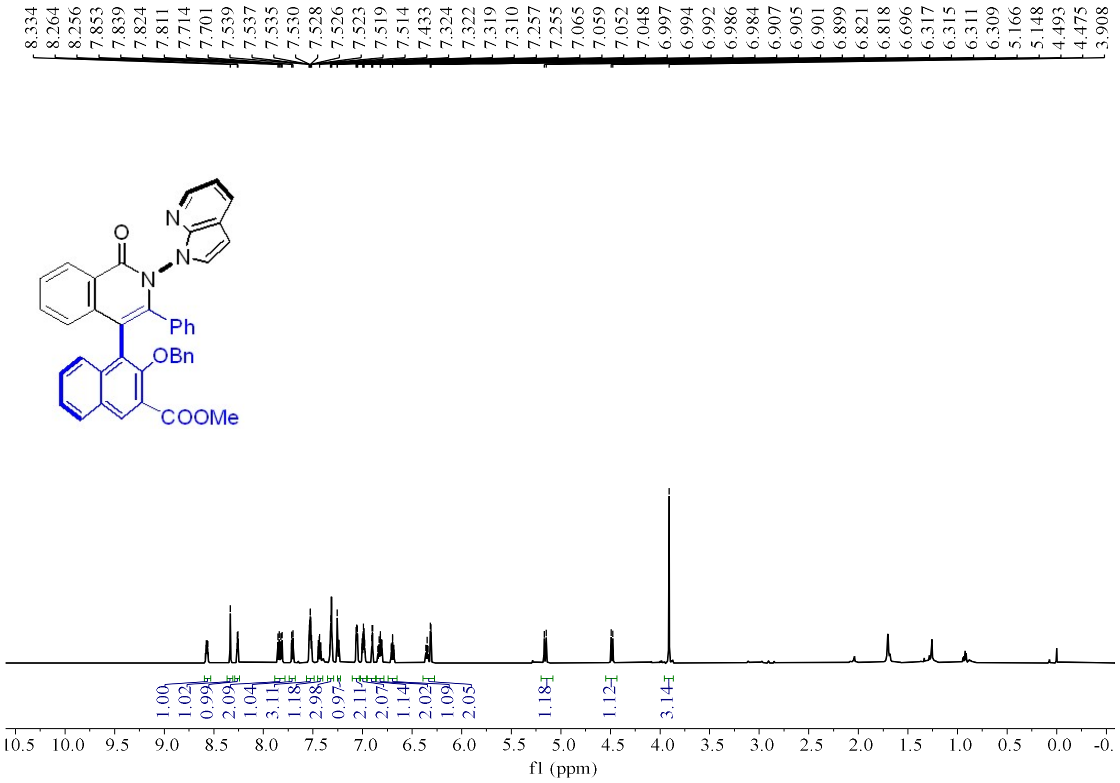


**Supplementary Figure 303. $^{1}H$ NMR (600 MHz, $CDCl_3$) spectrum of dia-68.**

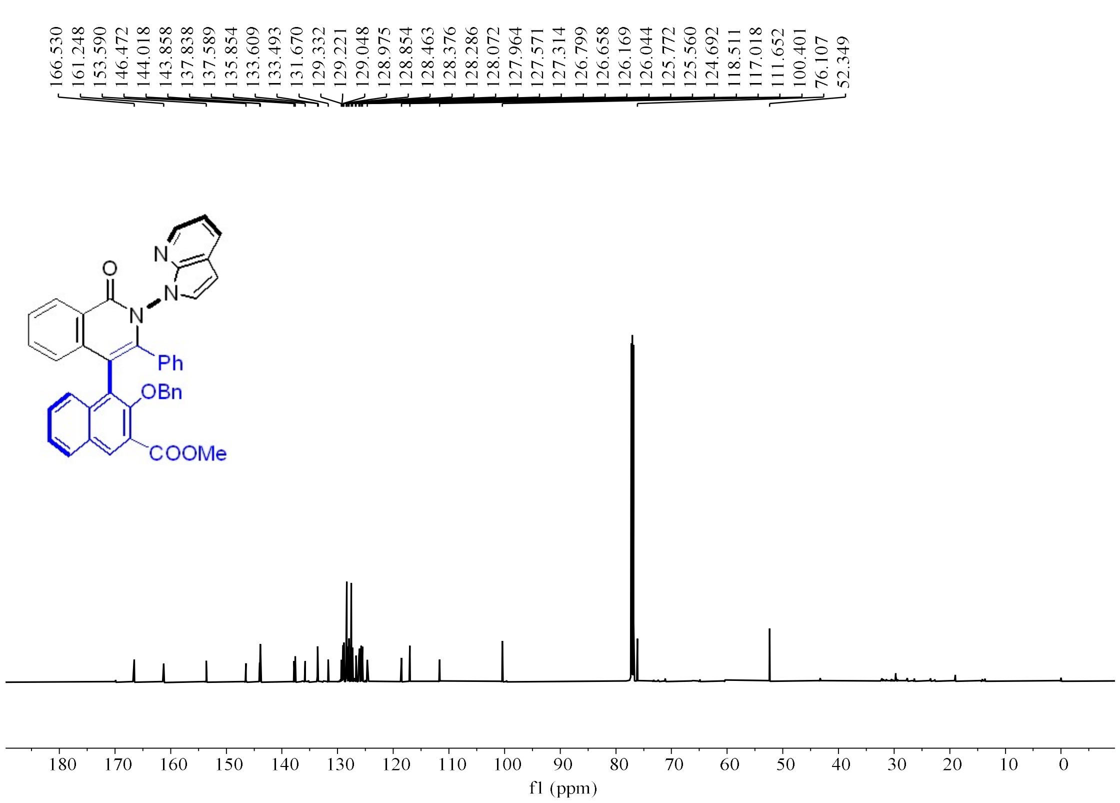


**Supplementary Figure 304. $^{13}C$ NMR (150 MHz, $CDCl_3$) spectrum of dia-68.**

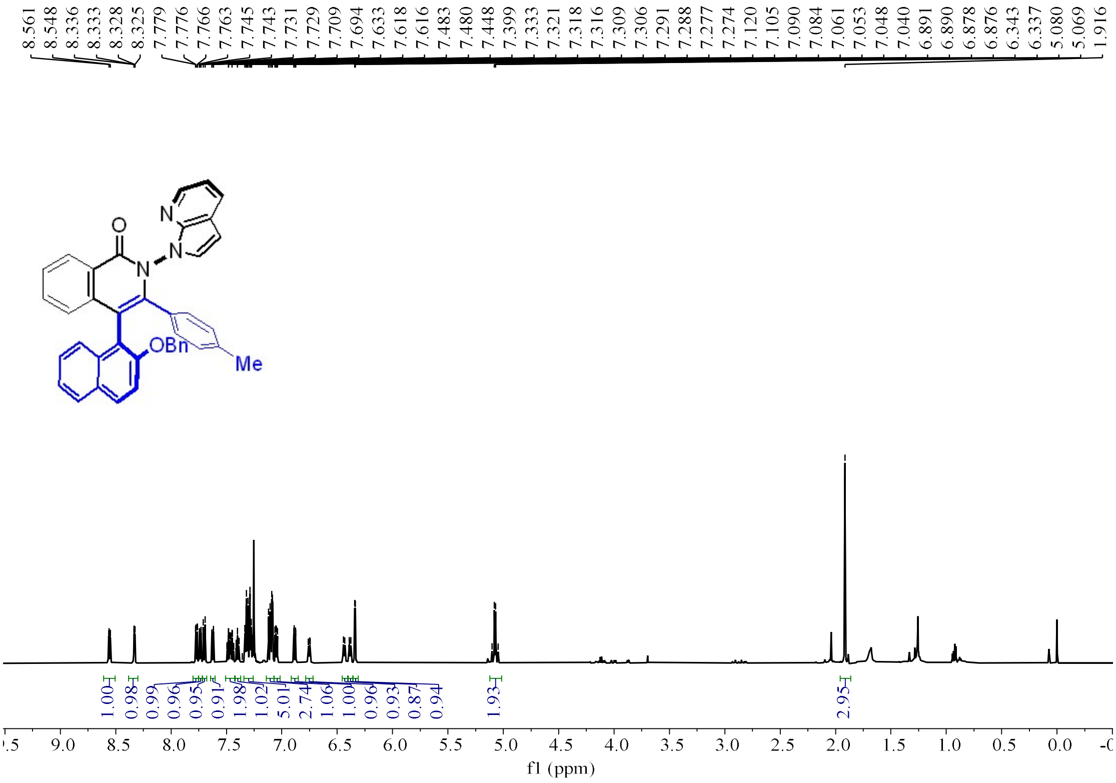


**Supplementary Figure 305. $^{1}H$ NMR (600 MHz, $CDCl_3$) spectrum of 69.**

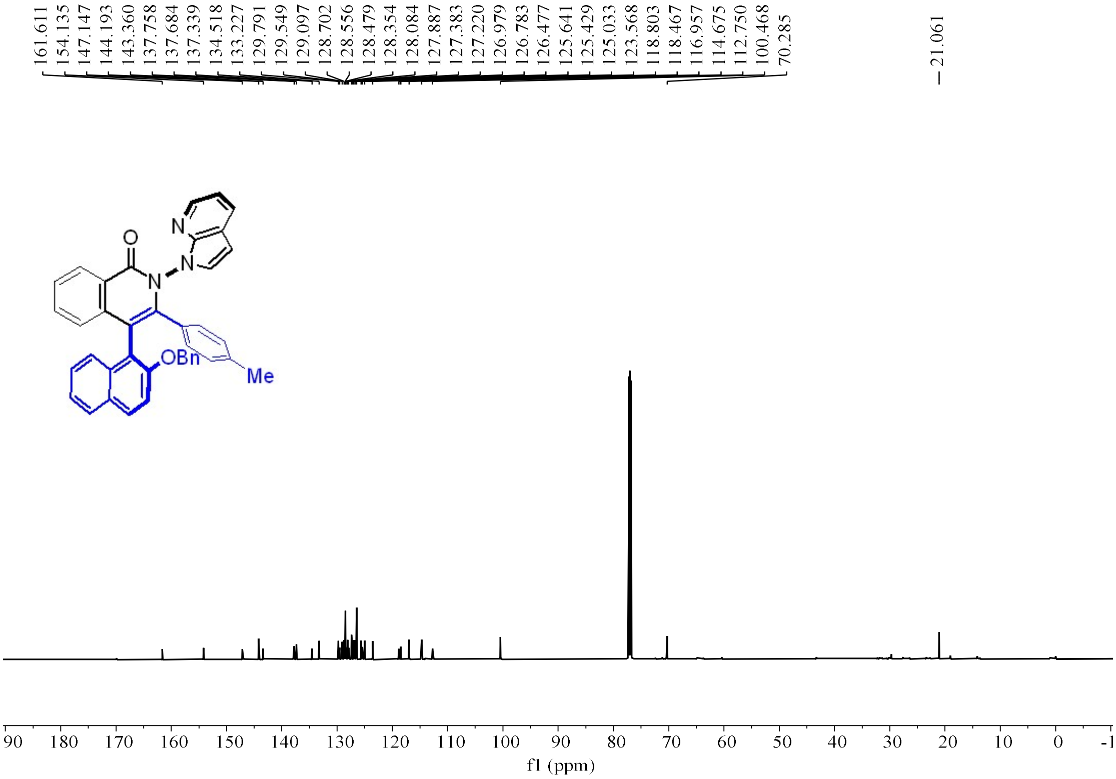


**Supplementary Figure 306. $^{13}C$ NMR (150 MHz, $CDCl_3$) spectrum of 69.**

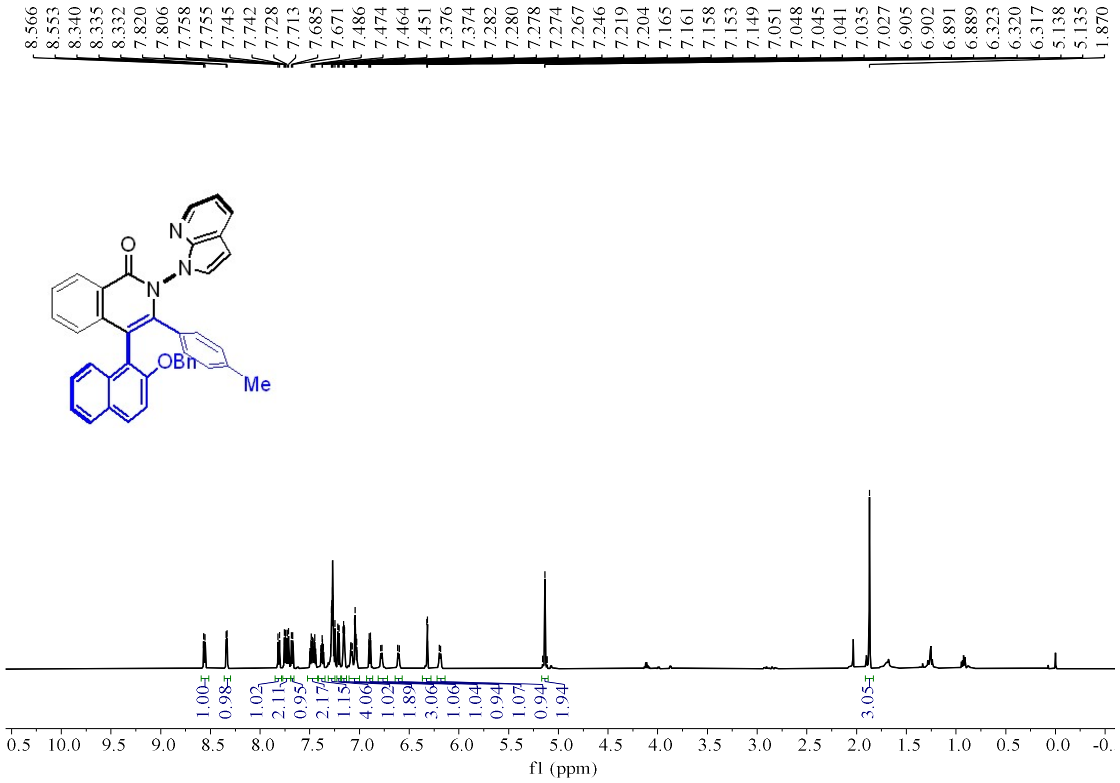


**Supplementary Figure 307.** $^{1}$**H NMR (600 MHz, CDCl$_3$) spectrum of dia-69.**

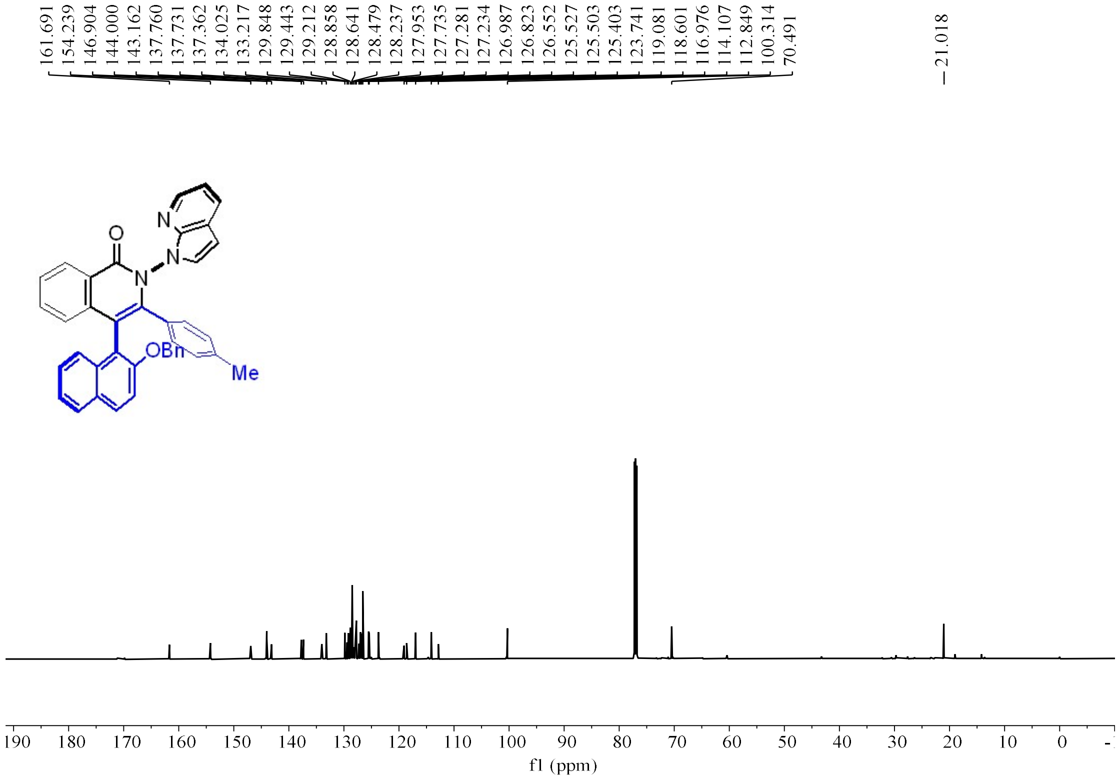


**Supplementary Figure 308.** $^{13}$**C NMR (150 MHz, CDCl$_3$) spectrum of dia-69.**

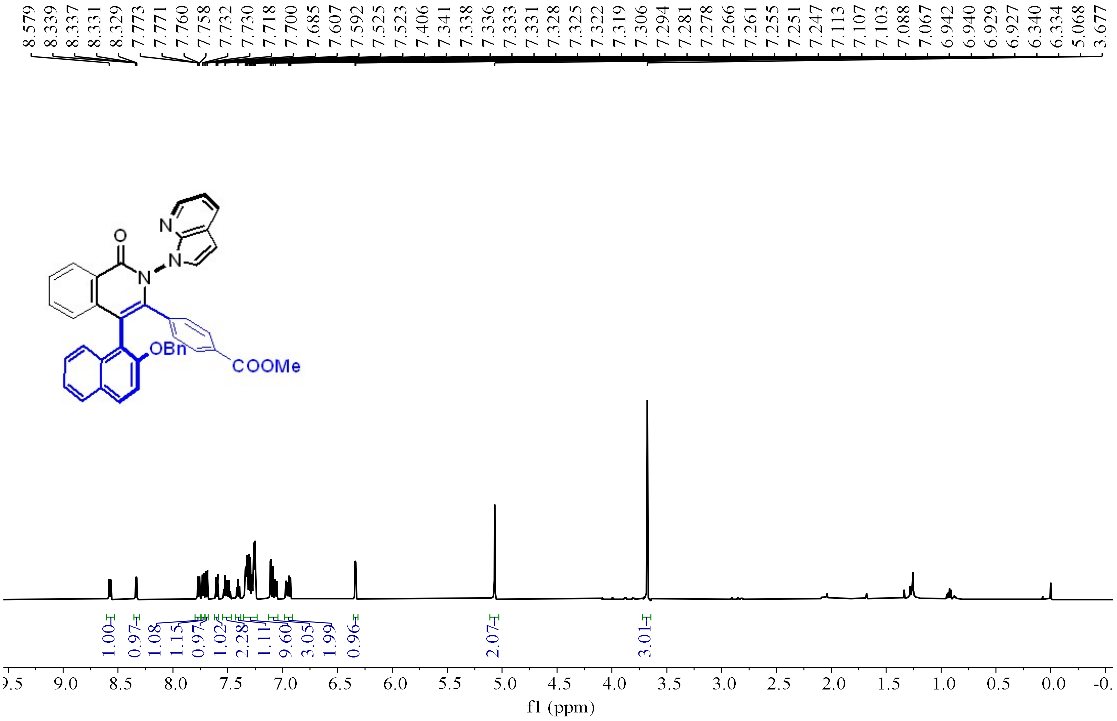


**Supplementary Figure 309. $^{1}$H NMR (600 MHz, $CDCl_3$) spectrum of 70.**

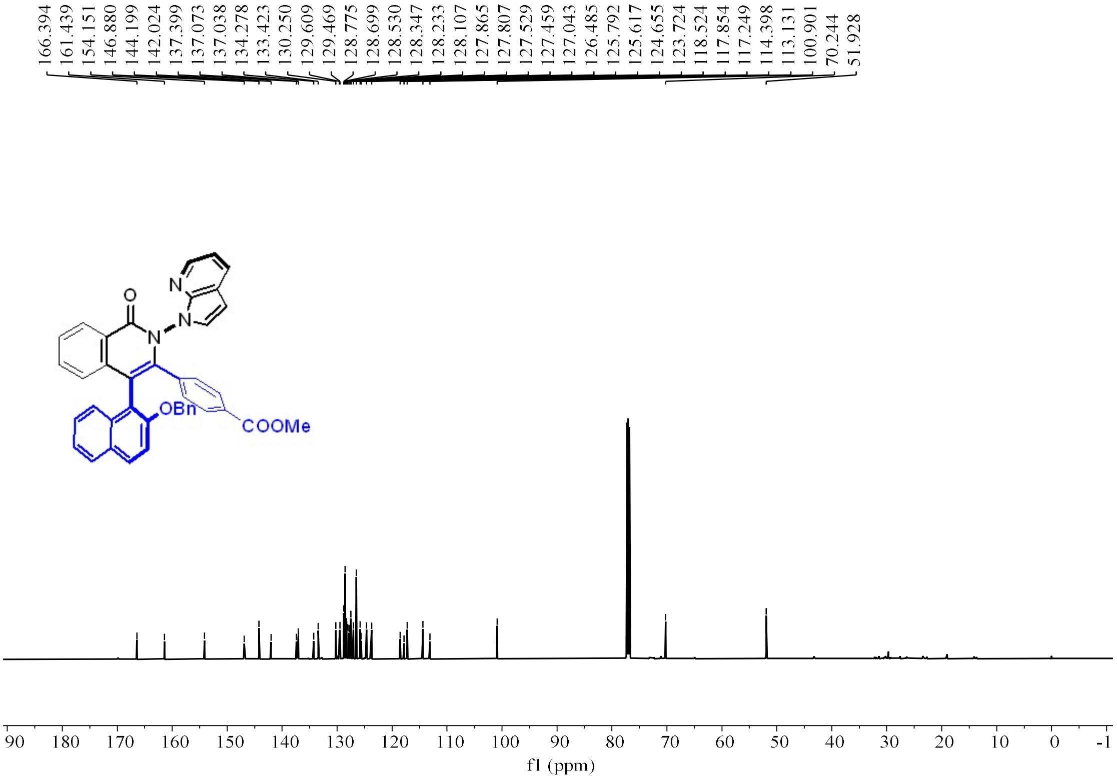


**Supplementary Figure 310. $^{13}$C NMR (150 MHz, $CDCl_3$) spectrum of 70.**

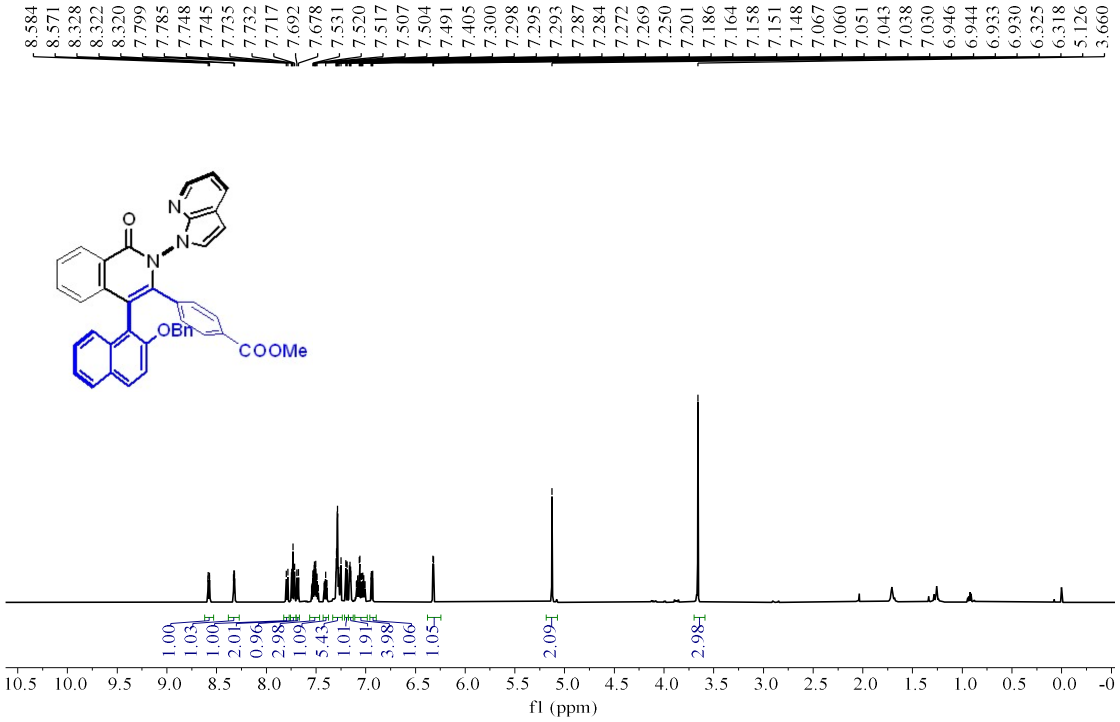


**Supplementary Figure 311. $^{1}$H NMR (600 MHz, $CDCl_3$) spectrum of dia-70.**

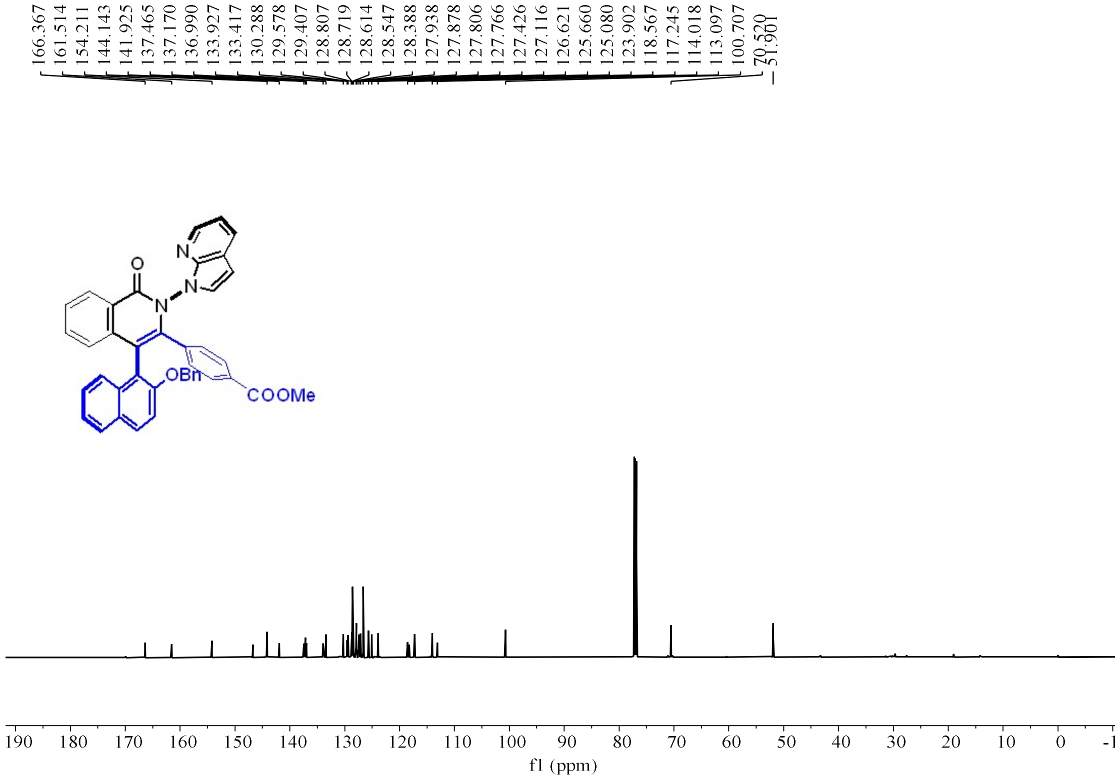


**Supplementary Figure 312. $^{13}$C NMR (150 MHz, $CDCl_3$) spectrum of dia-70.**

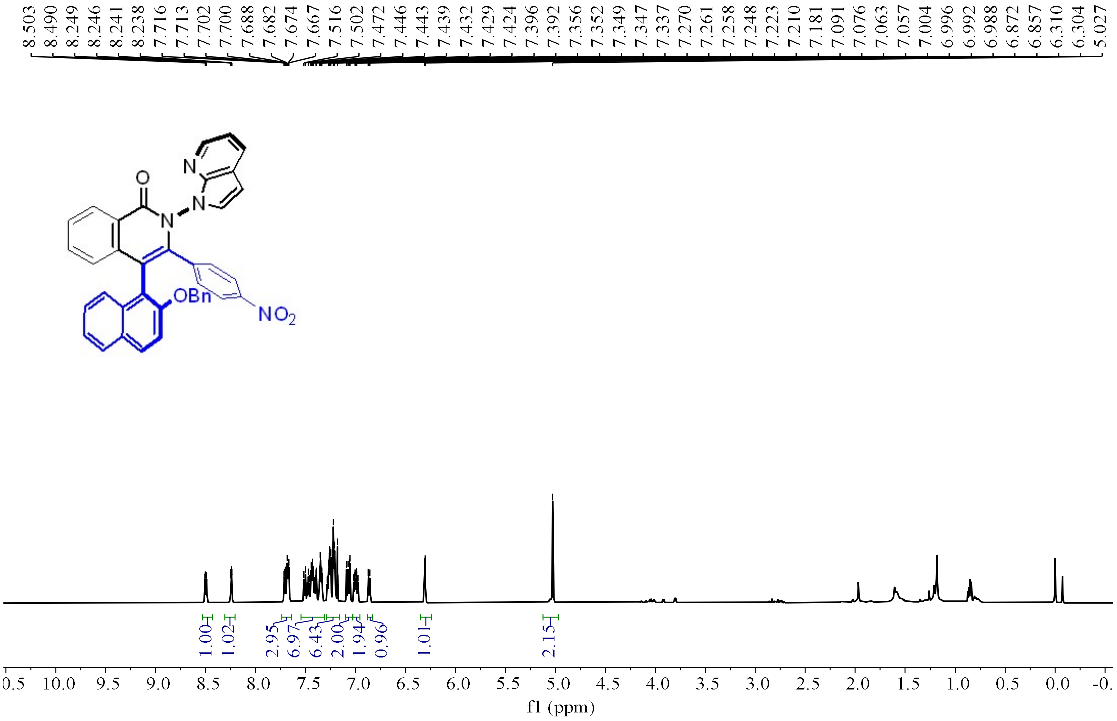


**Supplementary Figure 313. $^{1}H$ NMR (600 MHz, $CDCl_3$) spectrum of 71.**

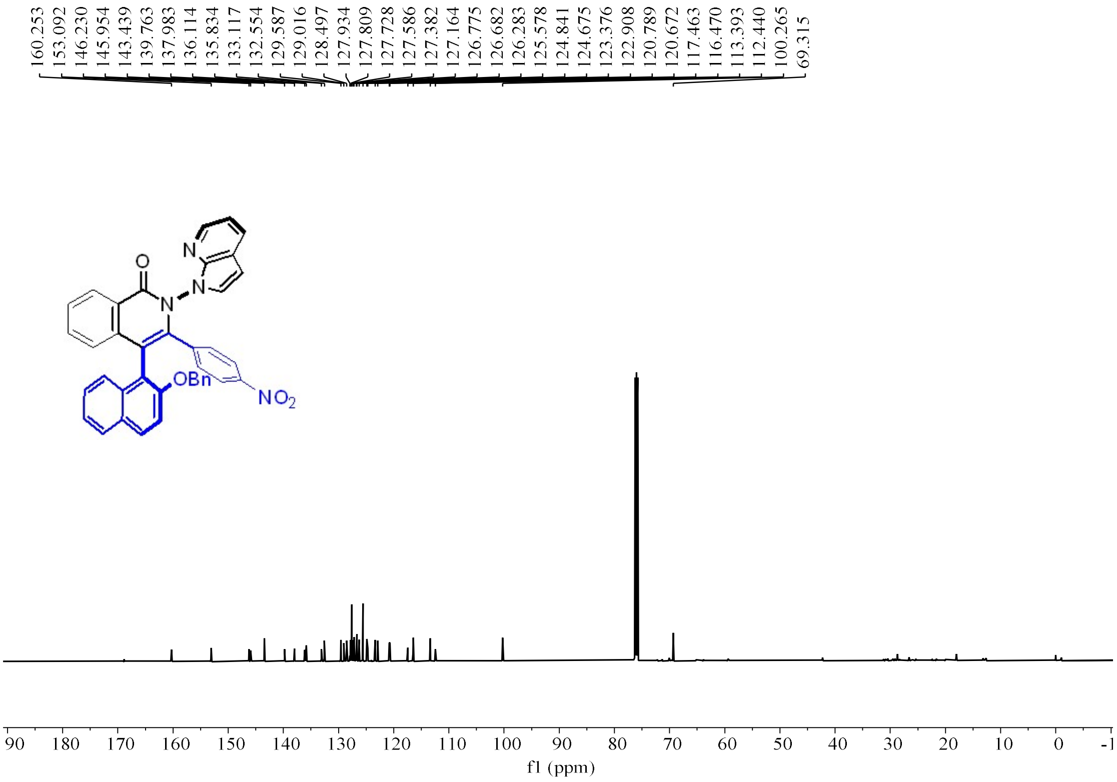


**Supplementary Figure 314. $^{13}C$ NMR (150 MHz, $CDCl_3$) spectrum of 71.**

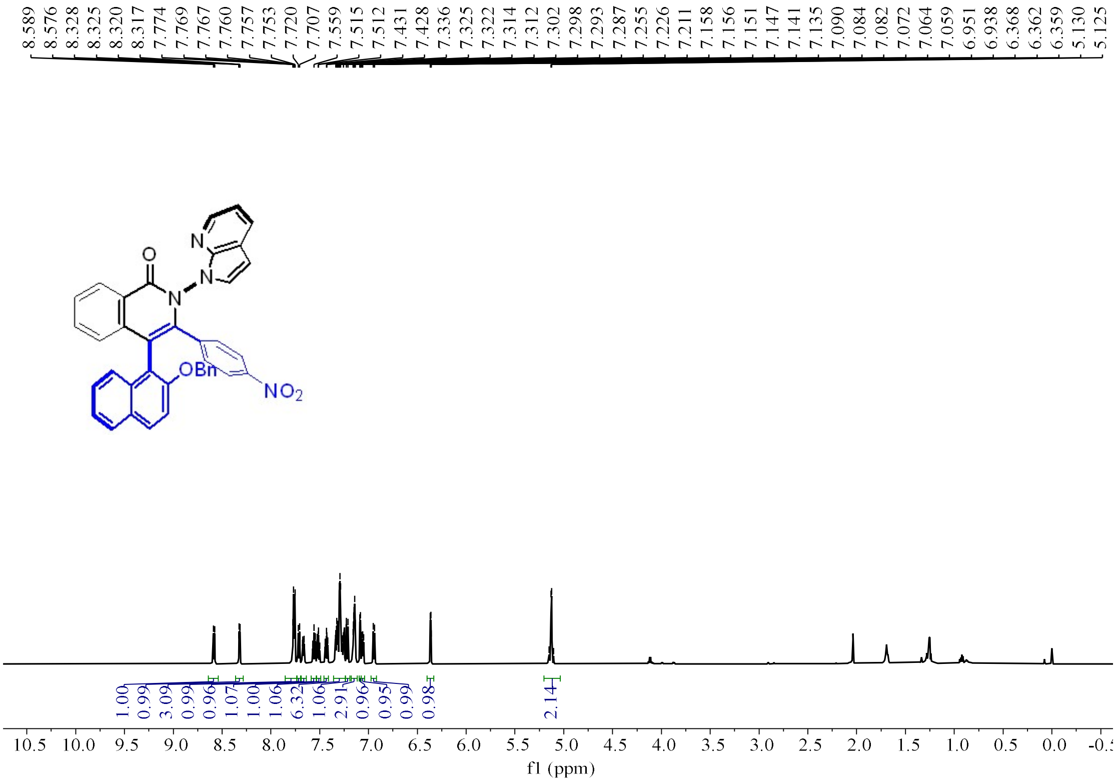


**Supplementary Figure 315. $^{1}$H NMR (600 MHz, $CDCl_3$) spectrum of dia-71.**

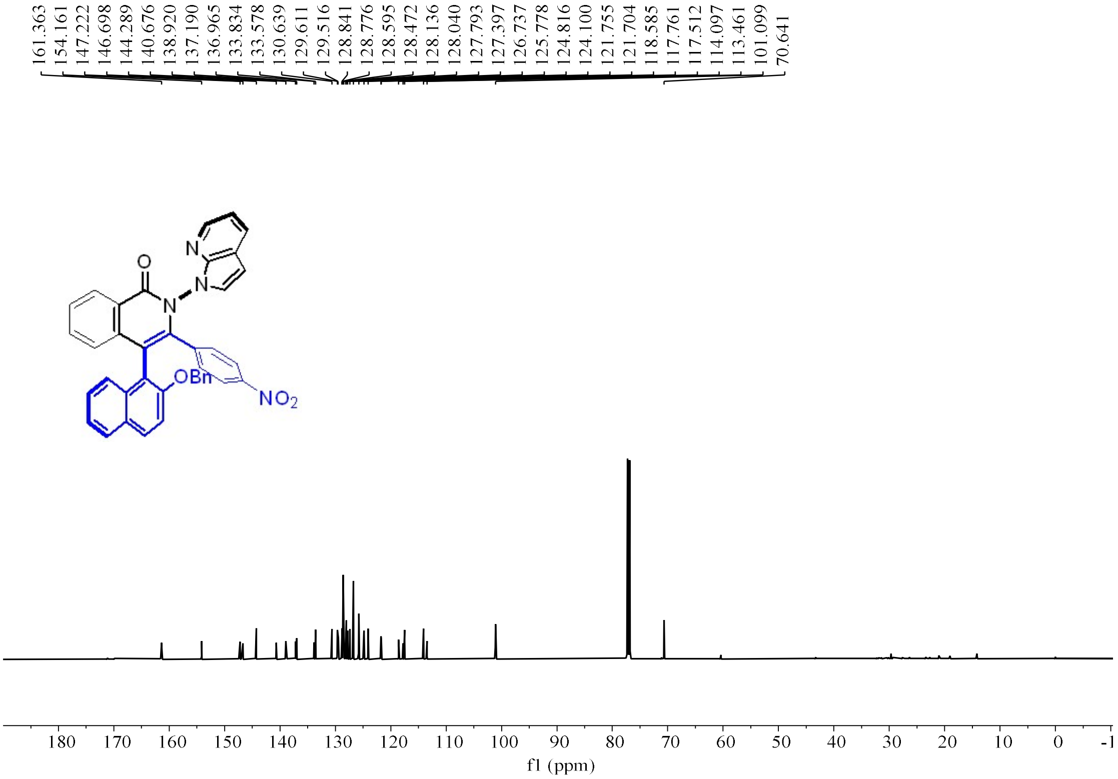


**Supplementary Figure 316. $^{13}$C NMR (150 MHz, $CDCl_3$) spectrum of dia-71.**

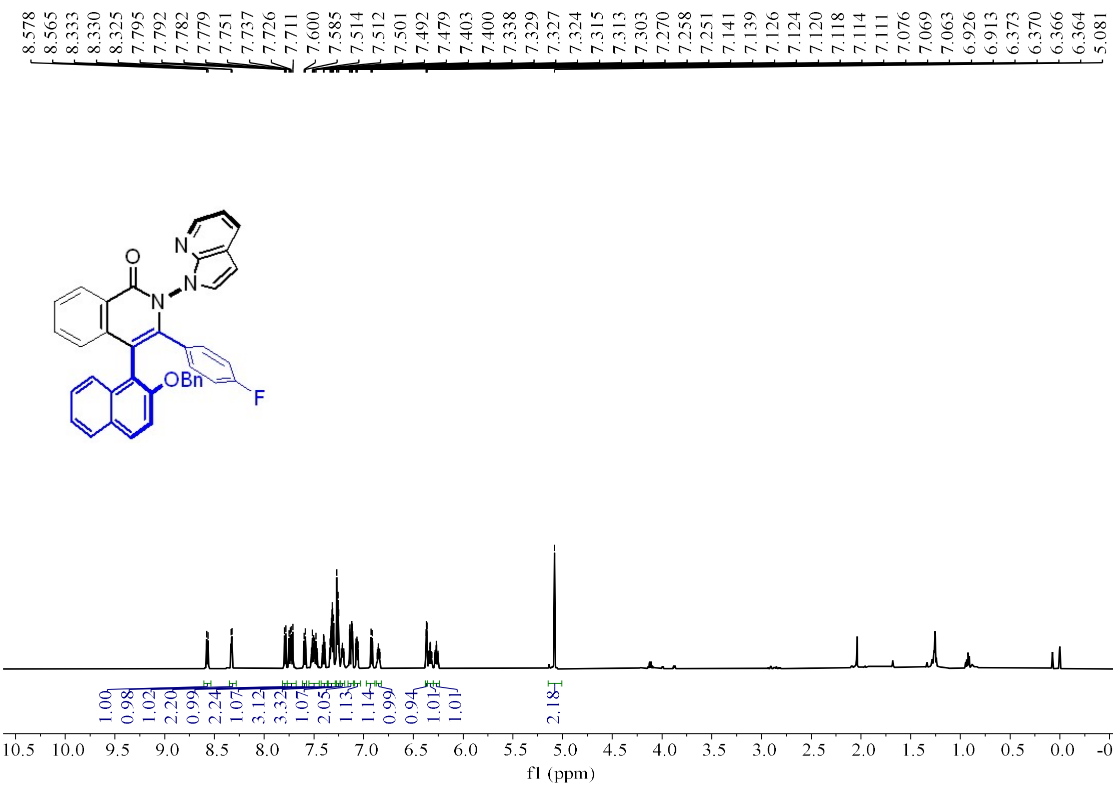


**Supplementary Figure 317.** $^{1}$**H NMR (600 MHz, CDCl**$_3$**) spectrum of 72.**

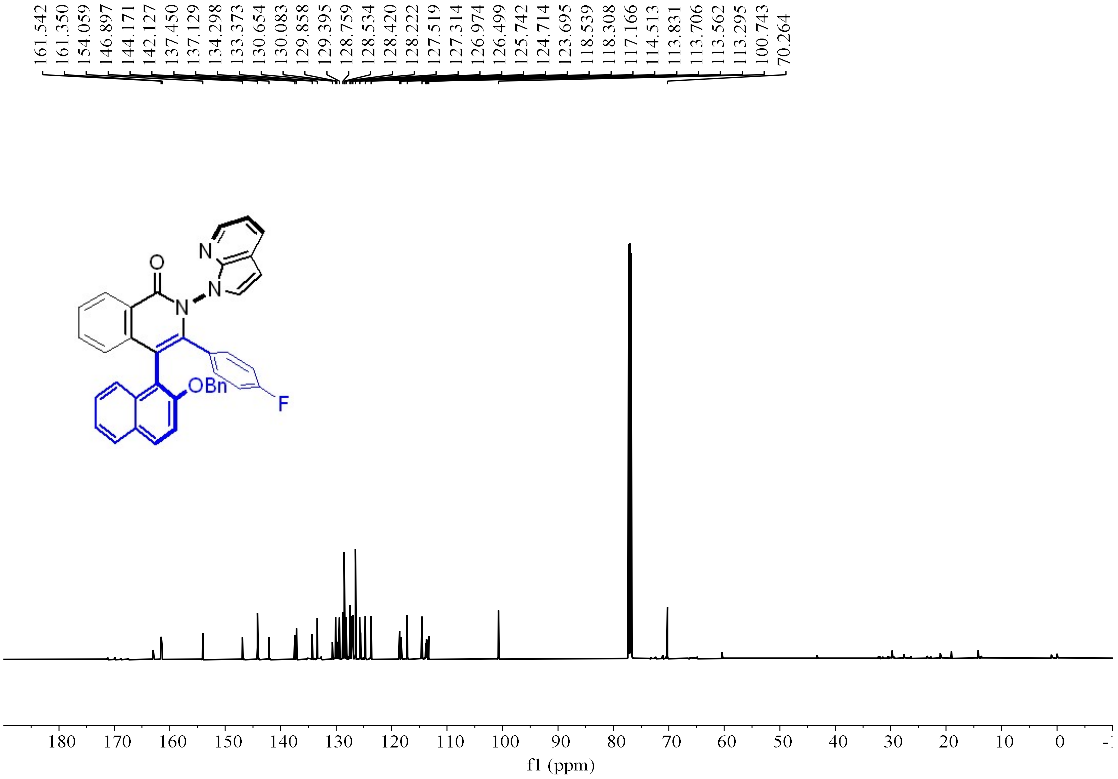


**Supplementary Figure 318.** $^{13}$**C NMR (150 MHz, CDCl**$_3$**) spectrum of 72.**

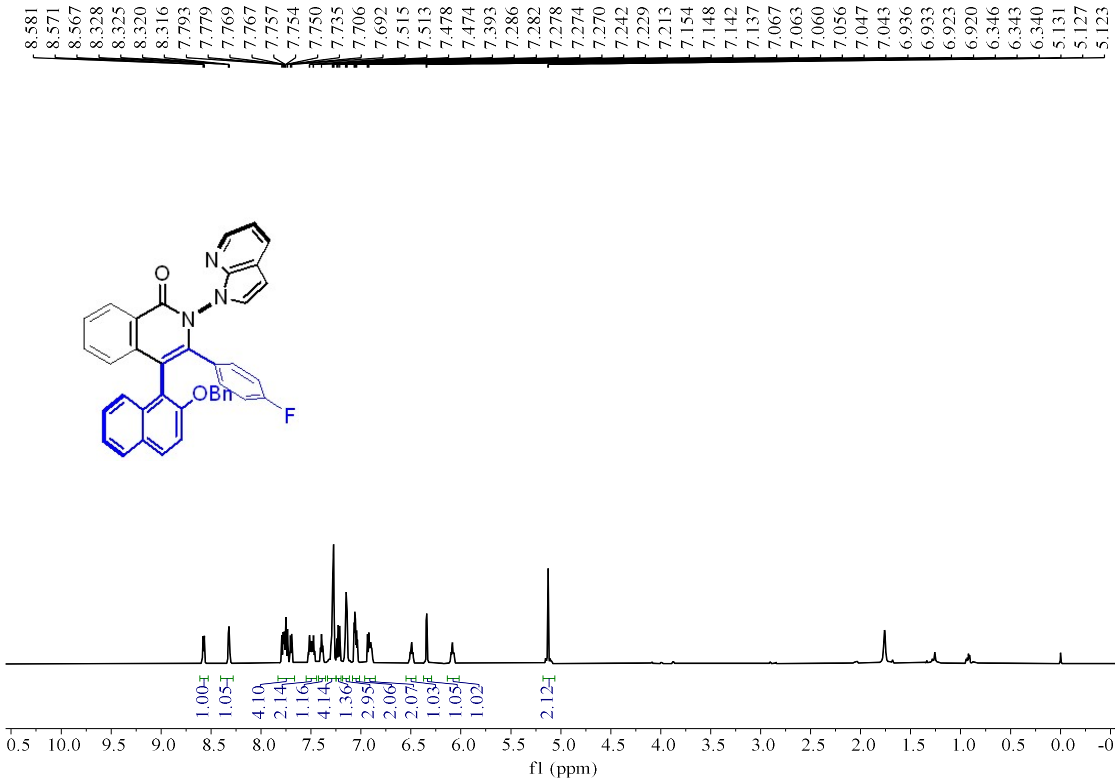


**Supplementary Figure 319.** $^{1}$**H NMR (600 MHz,** $CDCl_3$**) spectrum of dia-72.**

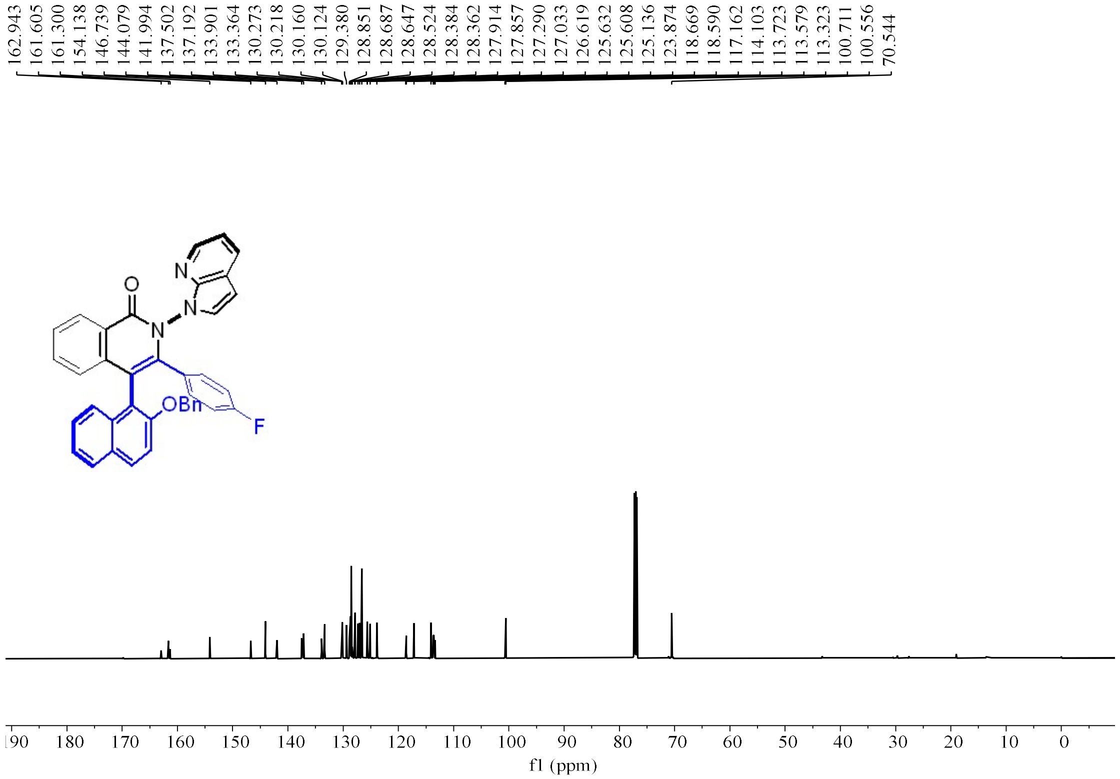


**Supplementary Figure 320.** $^{13}$**C NMR (150 MHz,** $CDCl_3$**) spectrum of dia-72.**

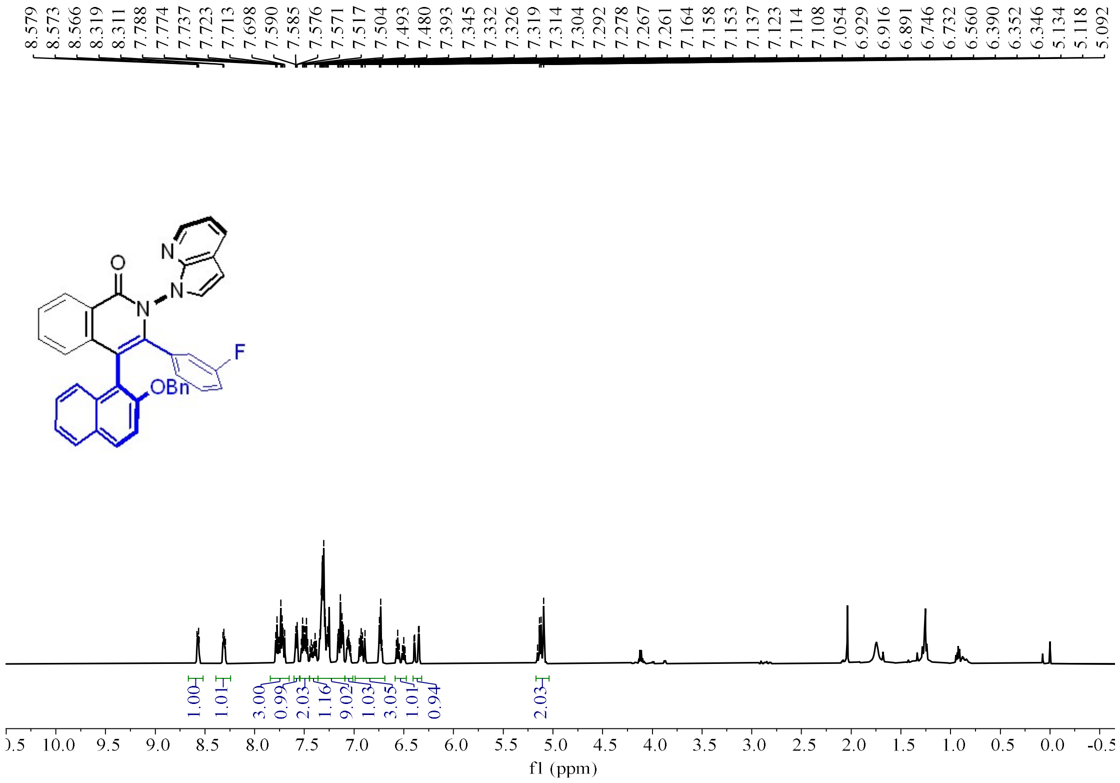


**Supplementary Figure 321. $^{1}$H NMR (600 MHz, $CDCl_3$) spectrum of 73.**

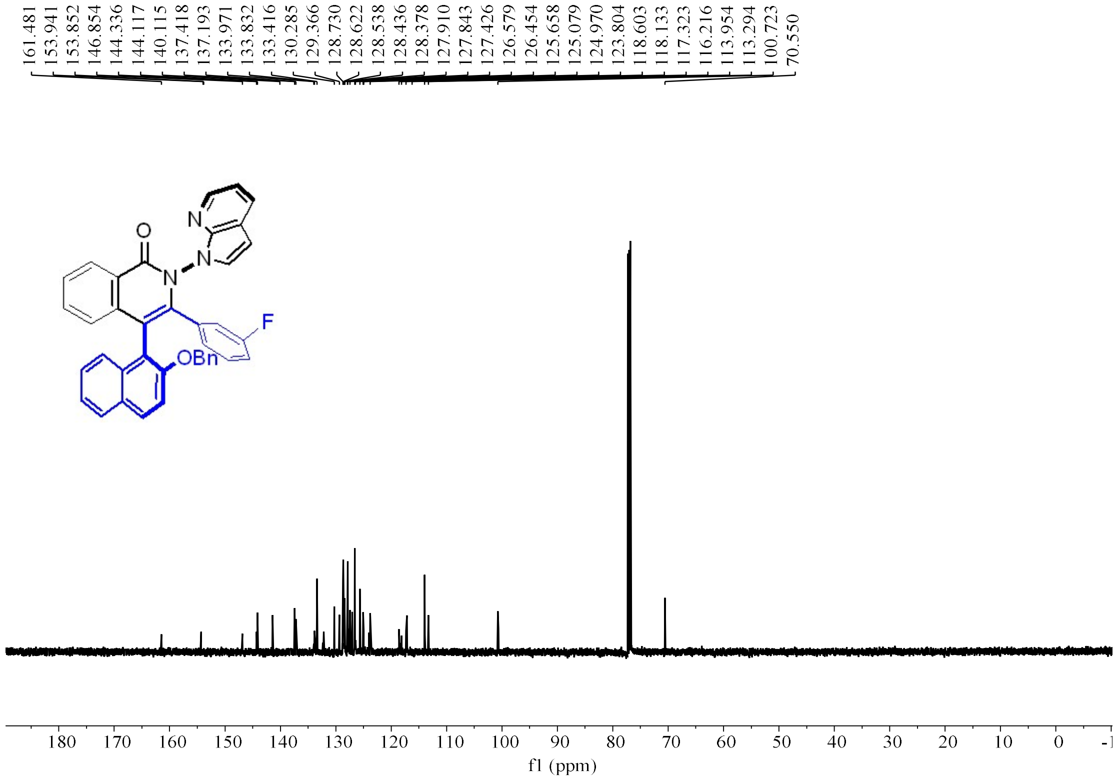


**Supplementary Figure 322. $^{13}$C NMR (150 MHz, $CDCl_3$) spectrum of 73.**

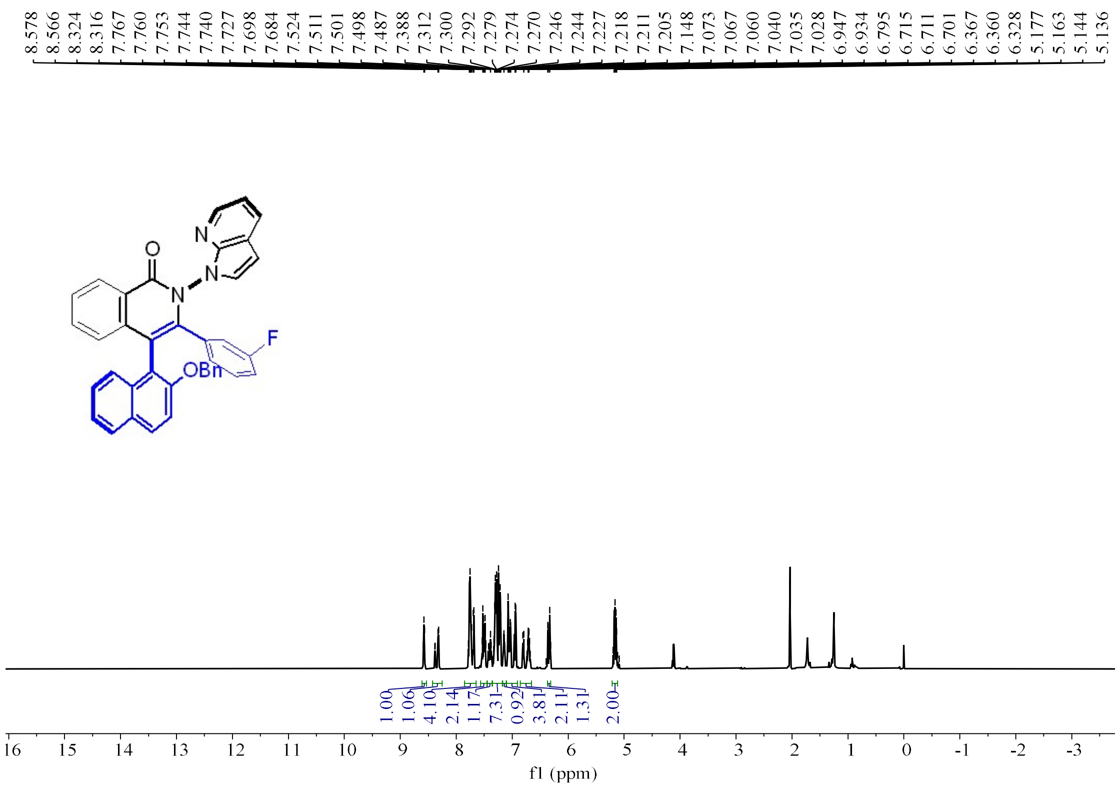


**Supplementary Figure 323.** $^{1}$**H NMR (600 MHz, CDCl$_3$) spectrum of dia-73.**

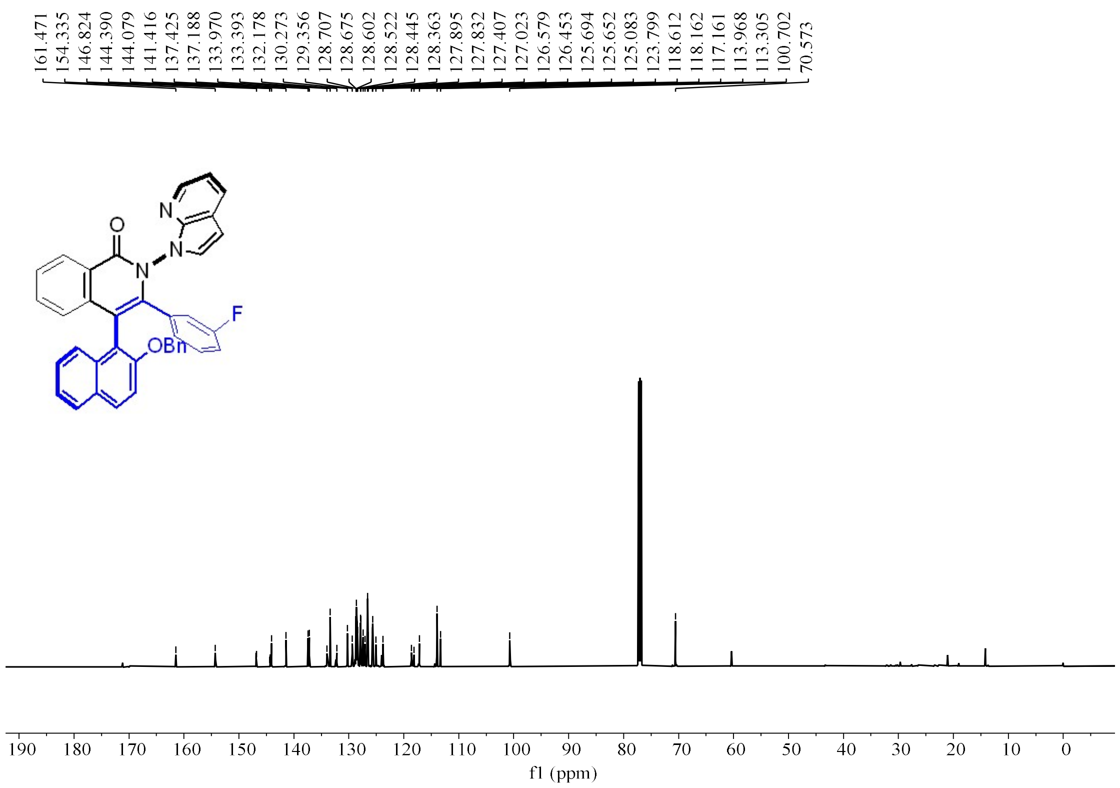


**Supplementary Figure 324.** $^{13}$**C NMR (150 MHz, CDCl$_3$) spectrum of dia-73.**

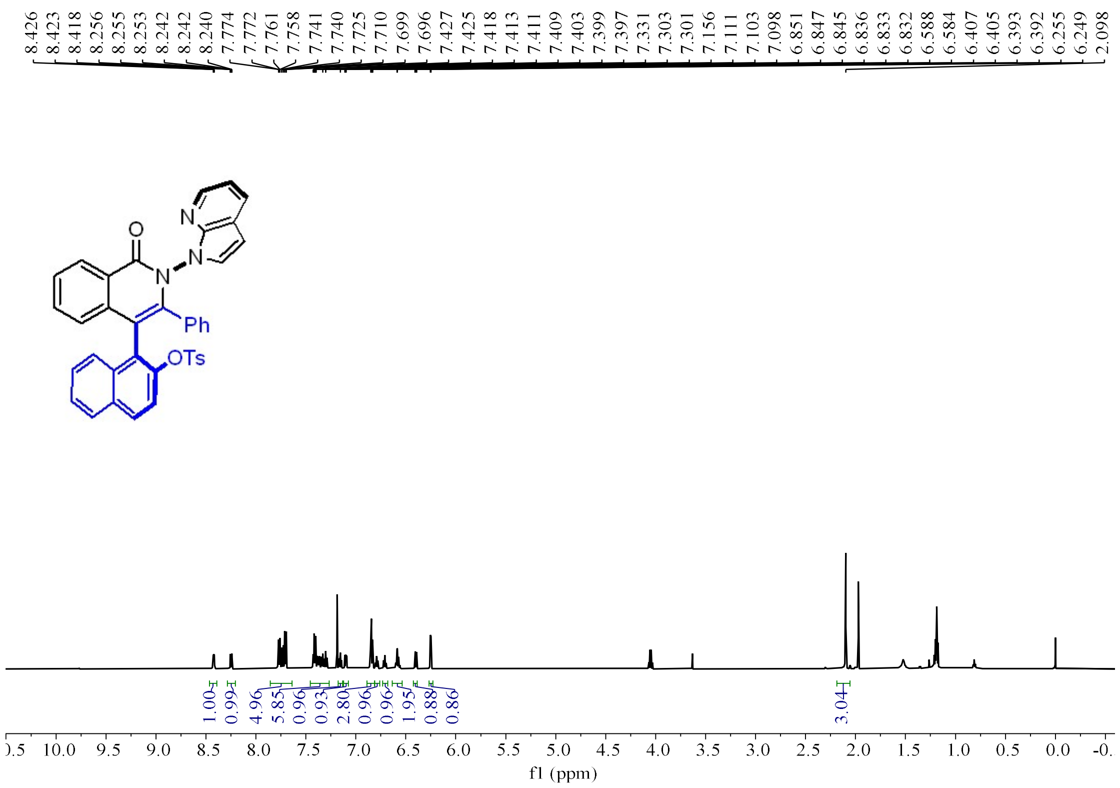


**Supplementary Figure 325. $^{1}$H NMR (600 MHz, $CDCl_3$) spectrum of 74.**

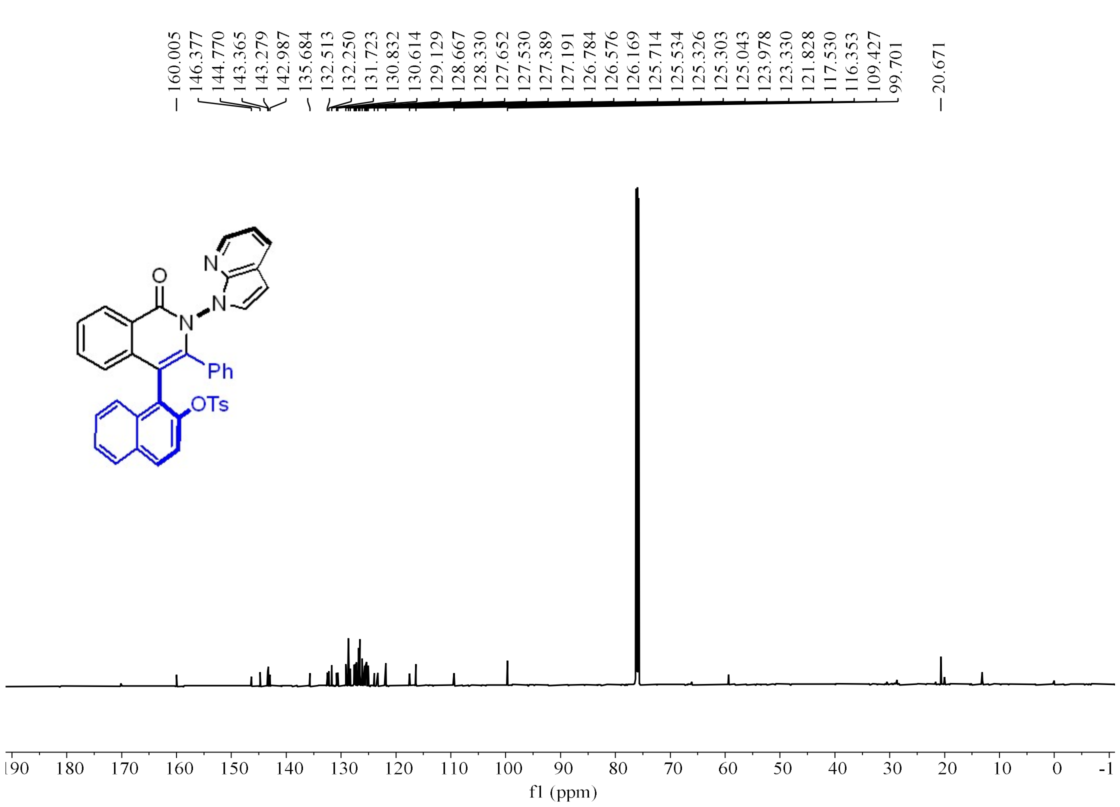


**Supplementary Figure 326. $^{13}$C NMR (150 MHz, $CDCl_3$) spectrum of 74.**

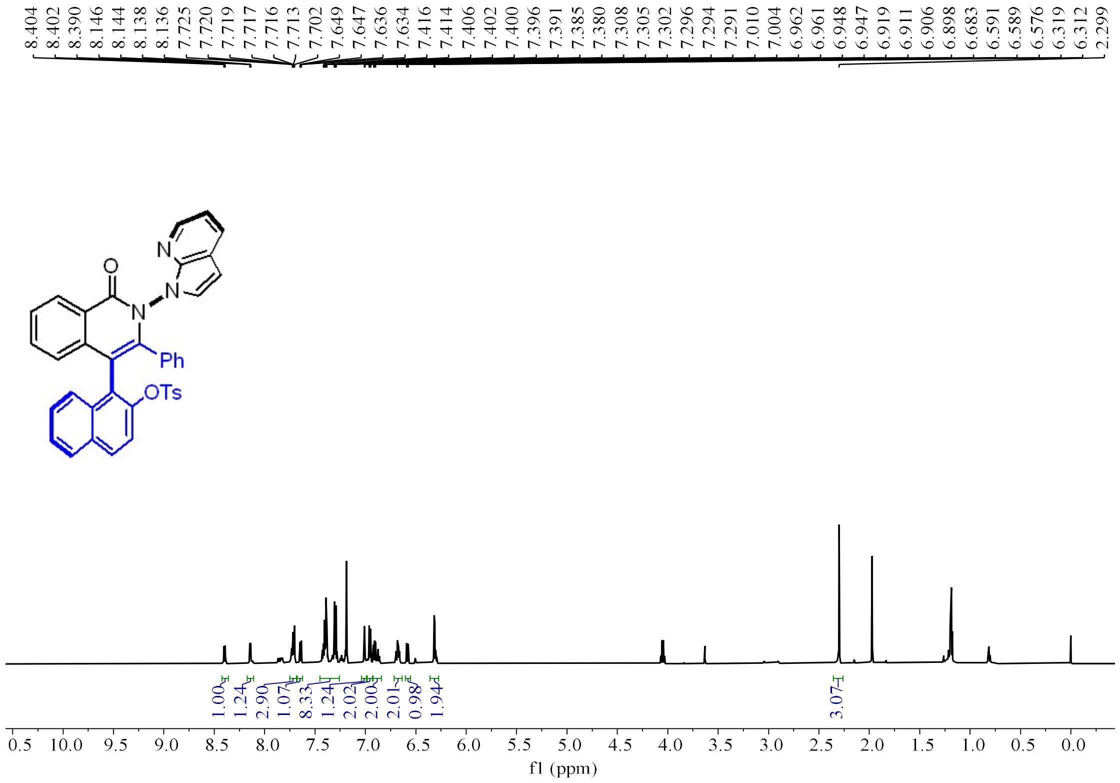


**Supplementary Figure 327. $^{1}$H NMR (600 MHz, CDCl$_3$) spectrum of dia-74.**

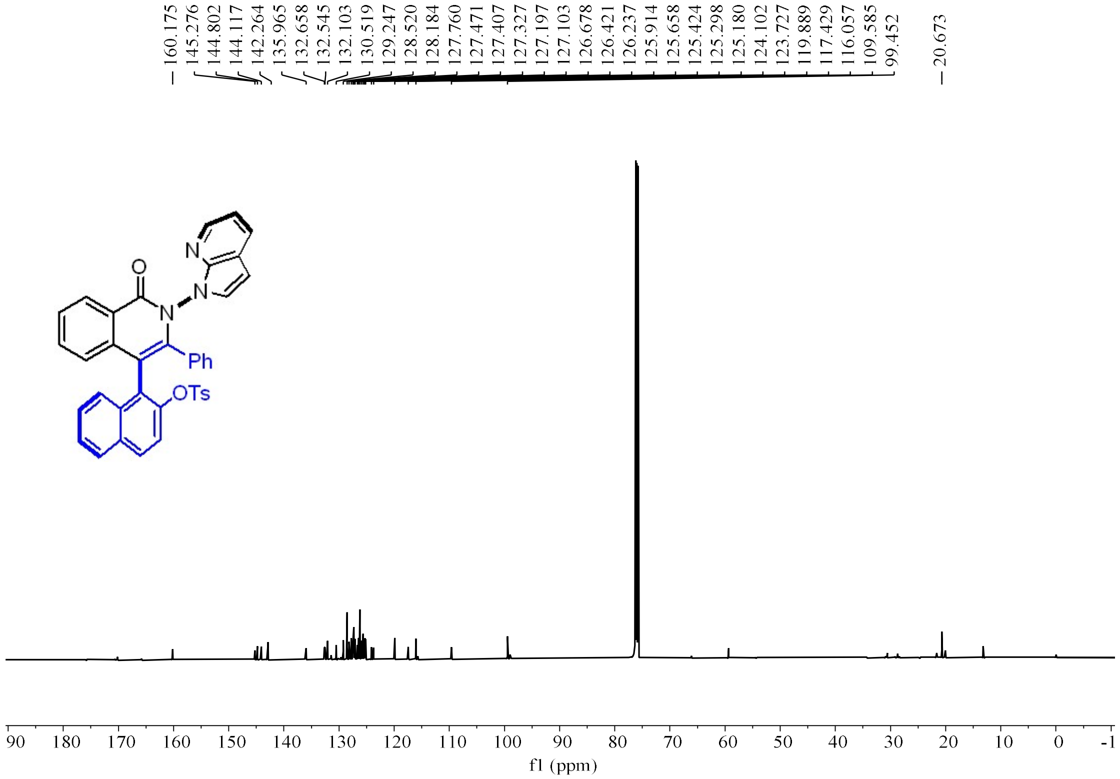


**Supplementary Figure 328. $^{13}$C NMR (150 MHz, CDCl$_3$) spectrum of dia-74.**

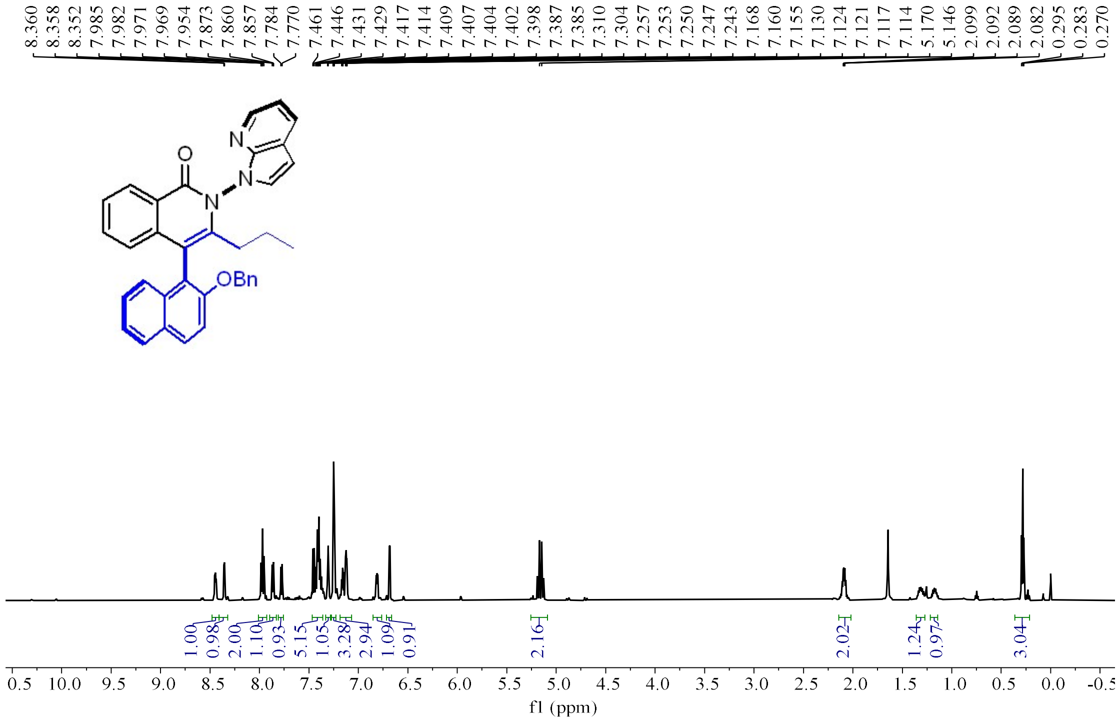


**Supplementary Figure 329. $^{1}$H NMR (600 MHz, $CDCl_3$) spectrum of dia-75.**

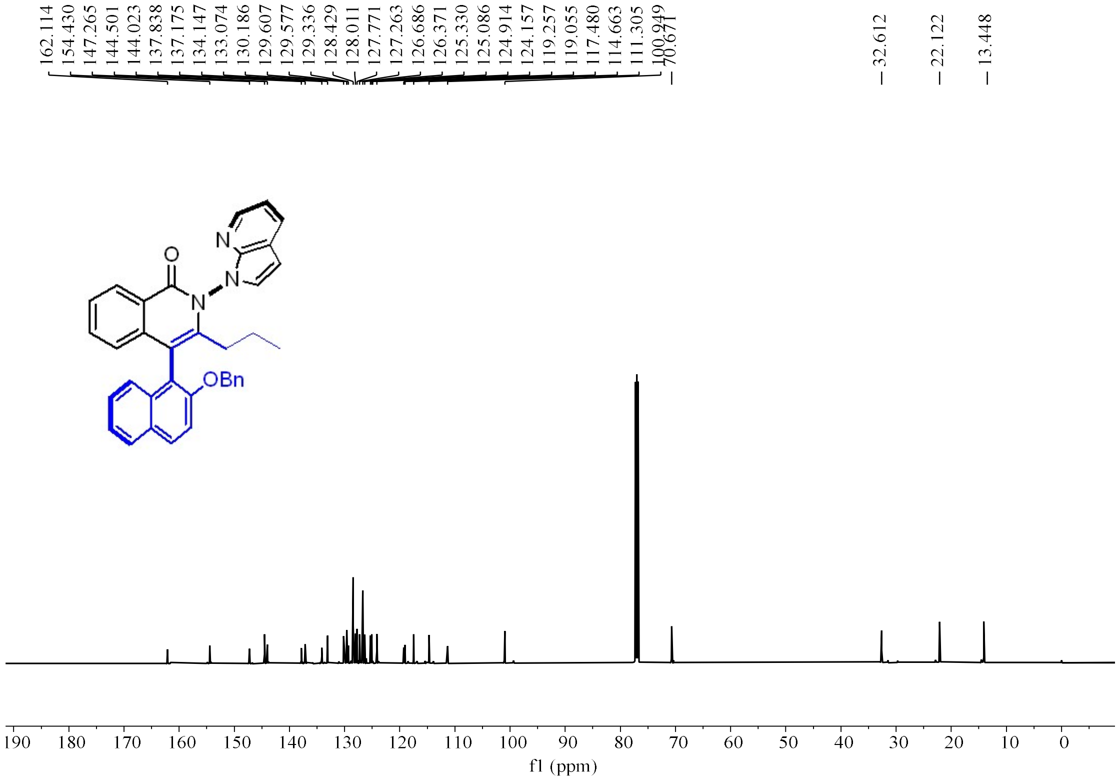


**Supplementary Figure 330. $^{13}$C NMR (150 MHz, $CDCl_3$) spectrum of dia-75.**

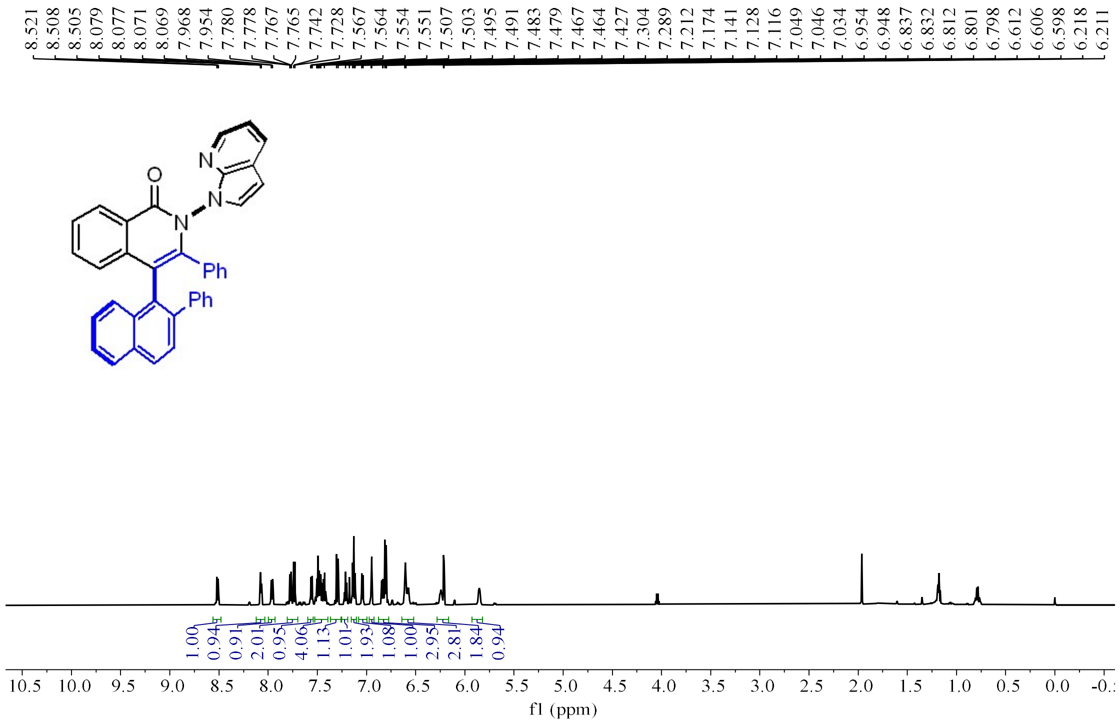

**Supplementary Figure 331.** $^{1}$**H NMR (600 MHz, CDCl$_3$) spectrum of dia-76.**

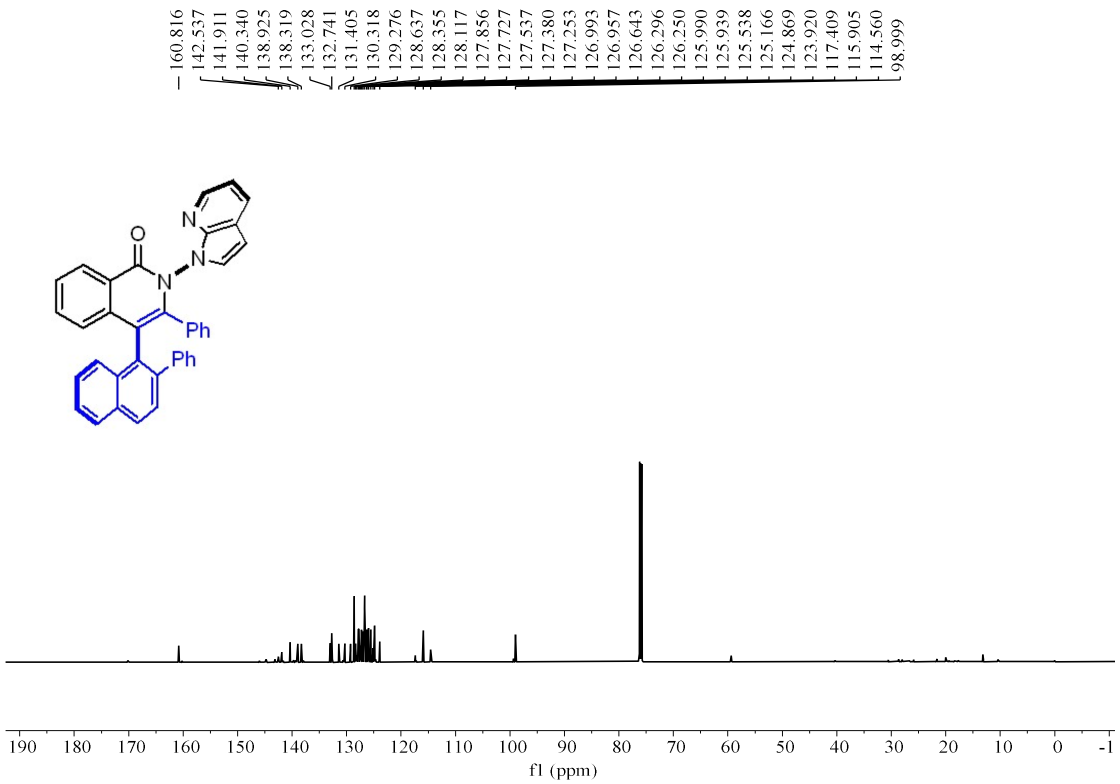

**Supplementary Figure 332.** $^{13}$**C NMR (150 MHz, CDCl$_3$) spectrum of dia-76.**

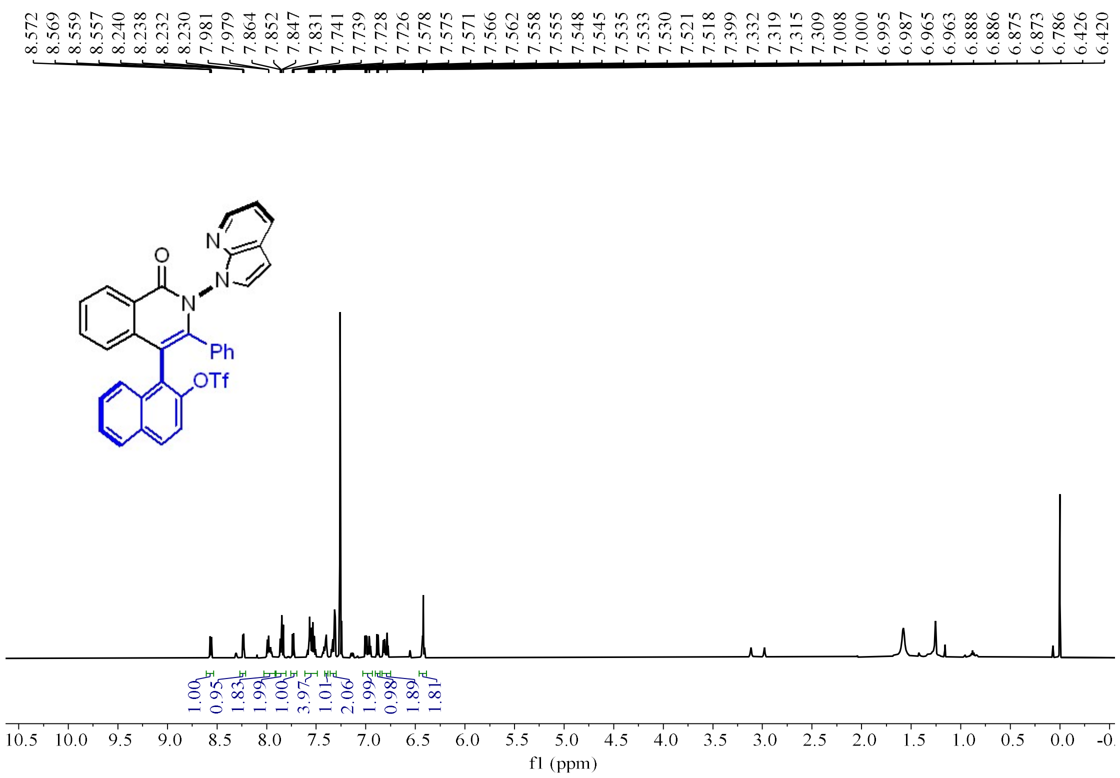


**Supplementary Figure 333. $^{1}$H NMR (600 MHz, $CDCl_3$) spectrum of dia-77.**

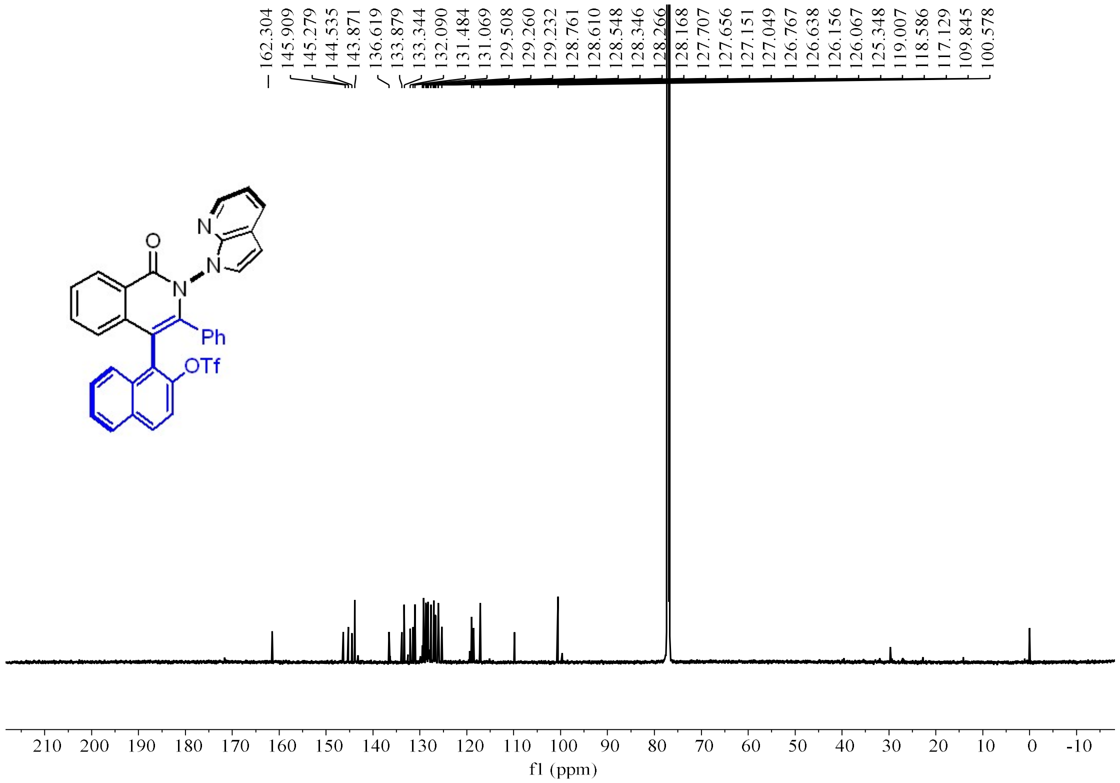


**Supplementary Figure 334. $^{1}$H NMR (600 MHz, $CDCl_3$) spectrum of dia-77.**

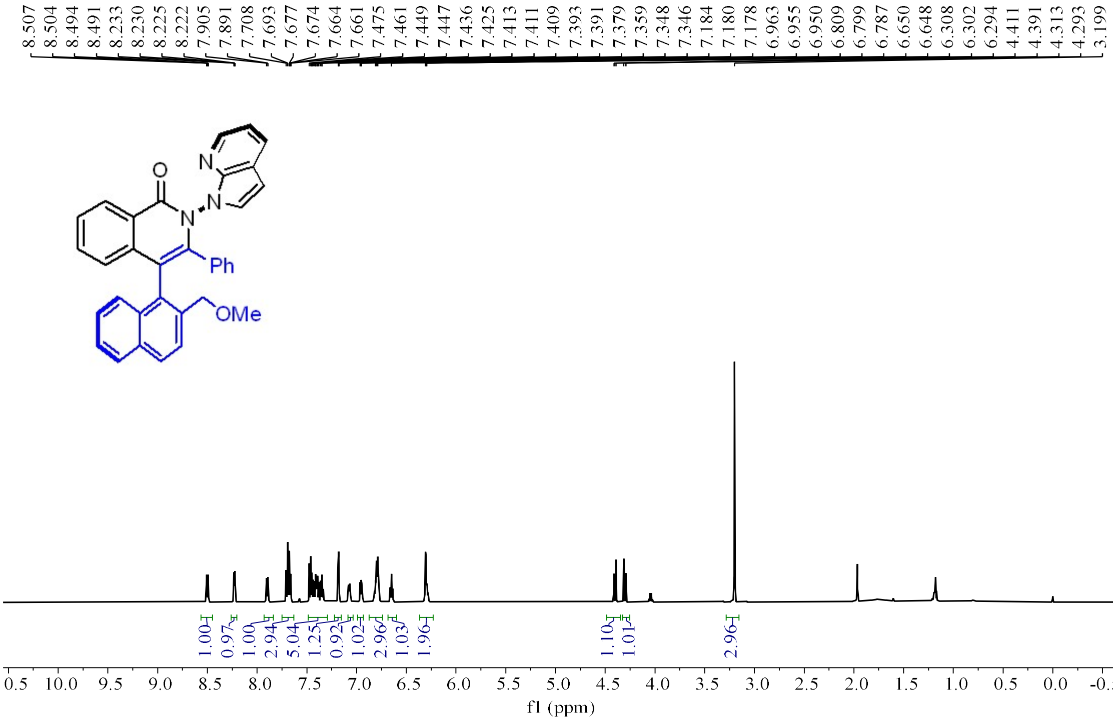


**Supplementary Figure 335.** $^{1}$**H NMR (600 MHz, CDCl$_3$) spectrum of dia-78.**

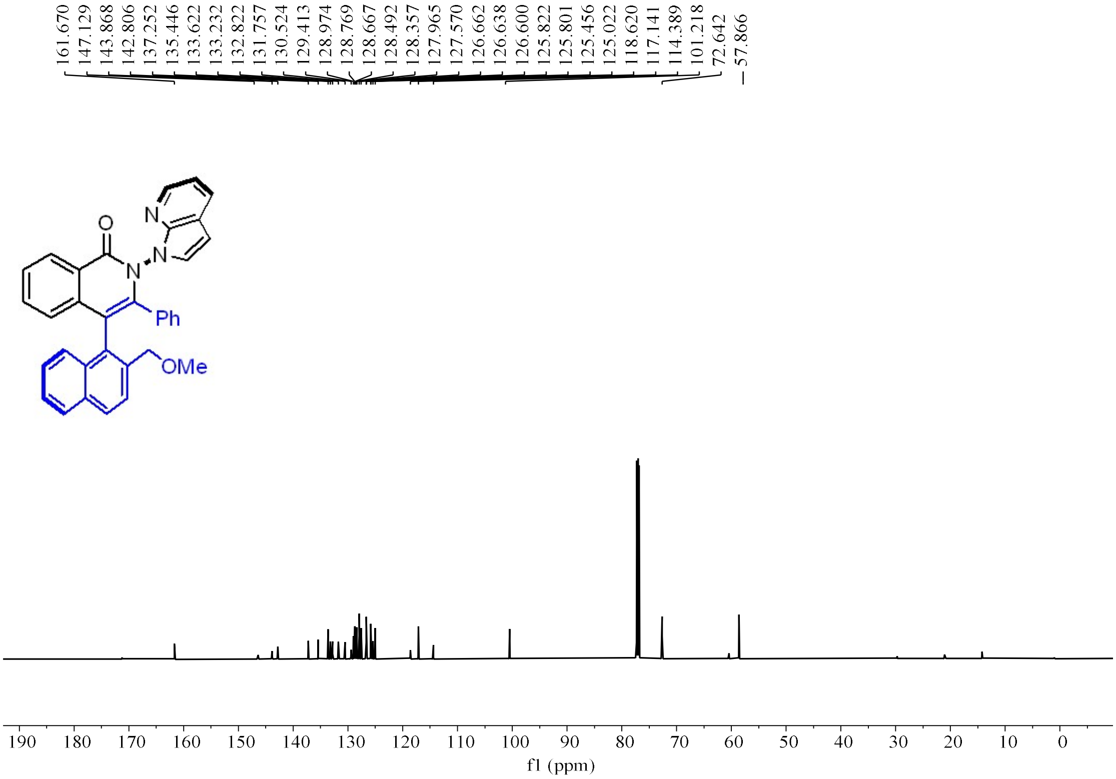


**Supplementary Figure 336.** $^{13}$**C NMR (150 MHz, CDCl$_3$) spectrum of dia-78.**

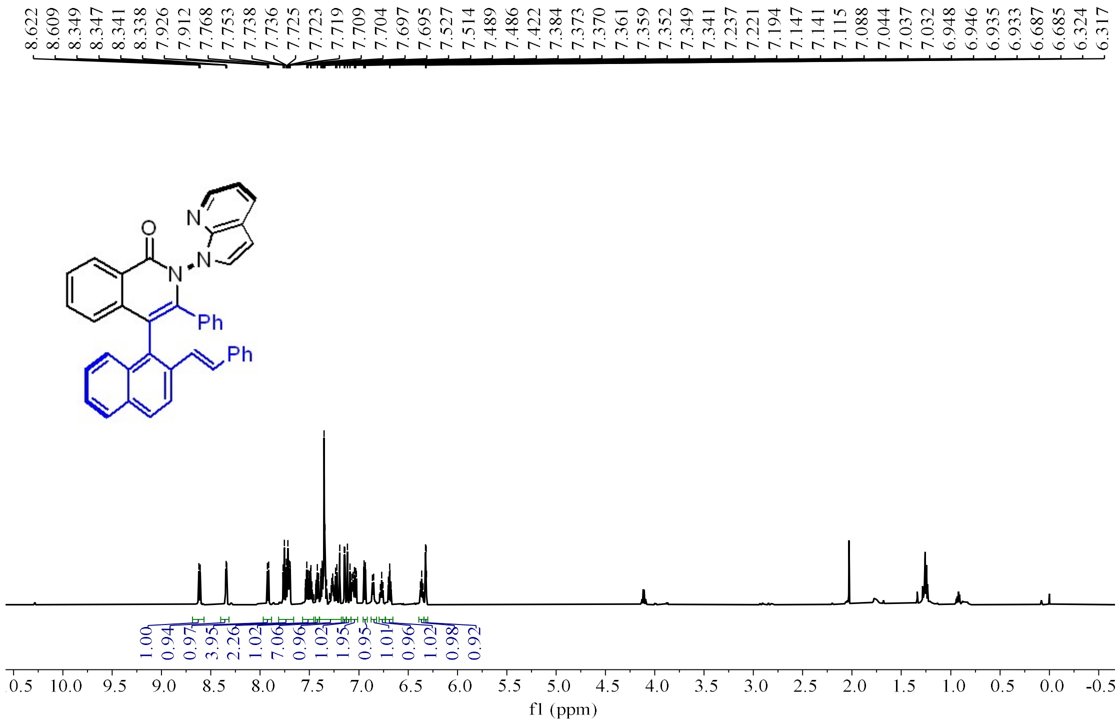

Supplementary Figure 337. $^{1}$H NMR (600 MHz, CDCl$_3$) spectrum of dia-79.

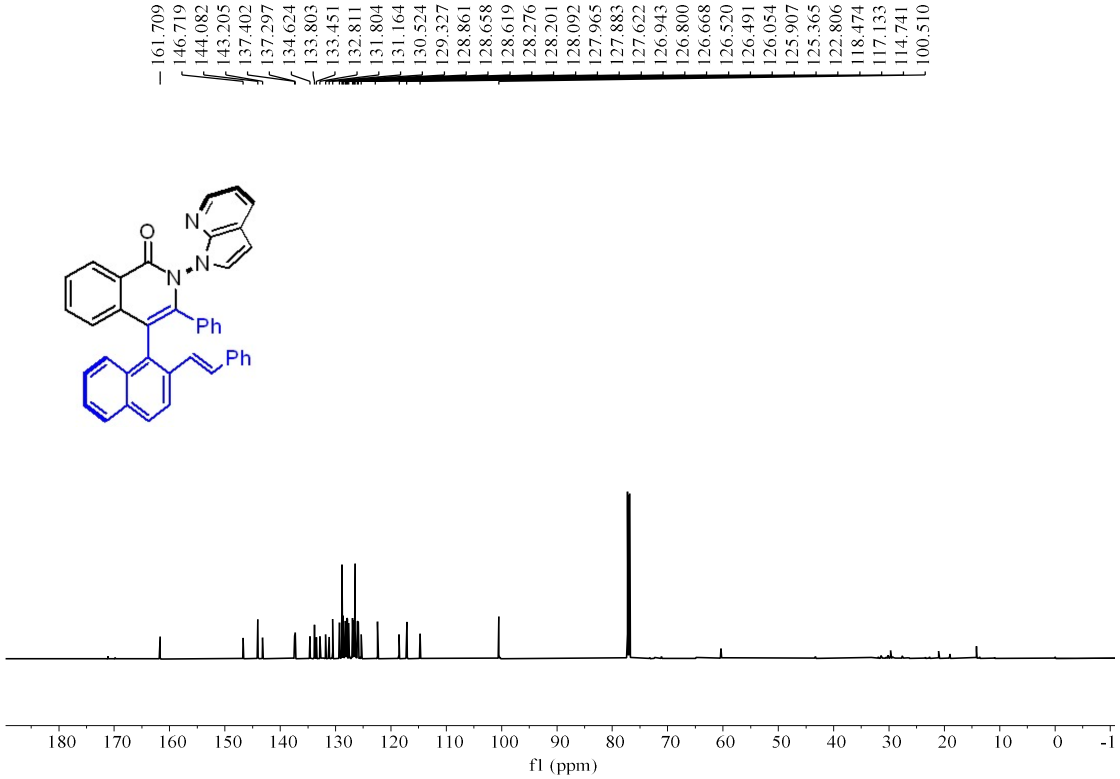

Supplementary Figure 338. $^{13}$C NMR (150 MHz, CDCl$_3$) spectrum of dia-79.

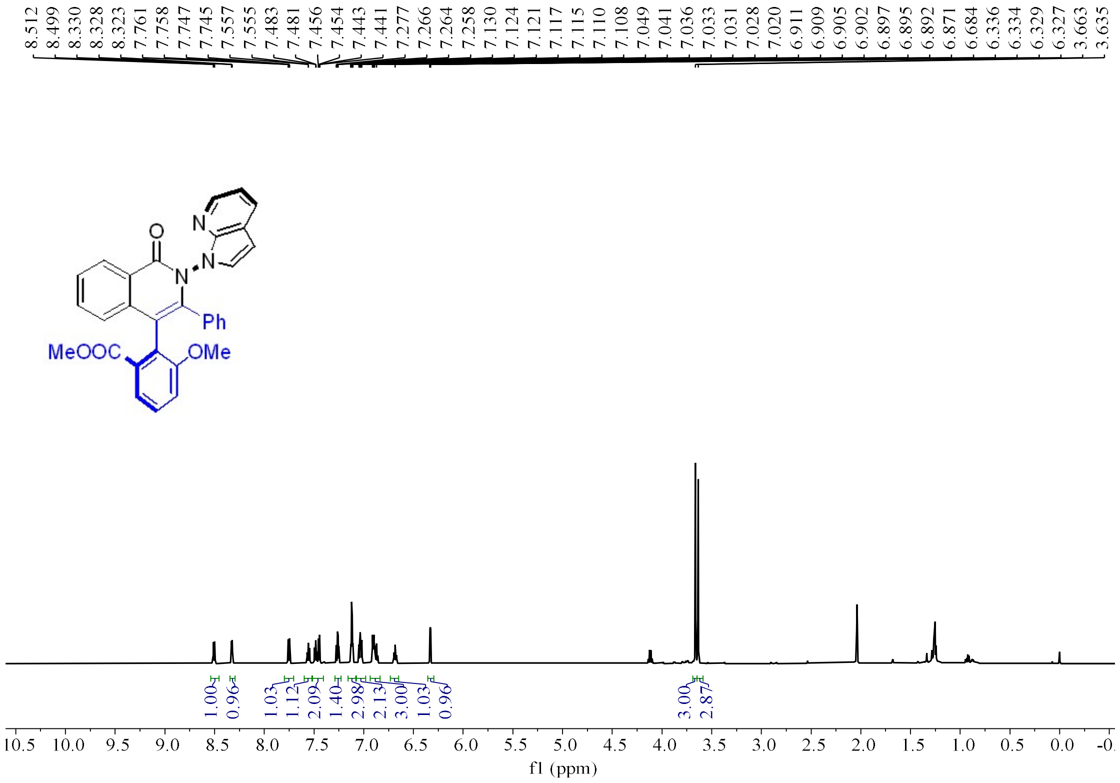


**Supplementary Figure 339.** $^{1}$**H NMR (600 MHz, CDCl**$_3$**) spectrum of dia-80.**

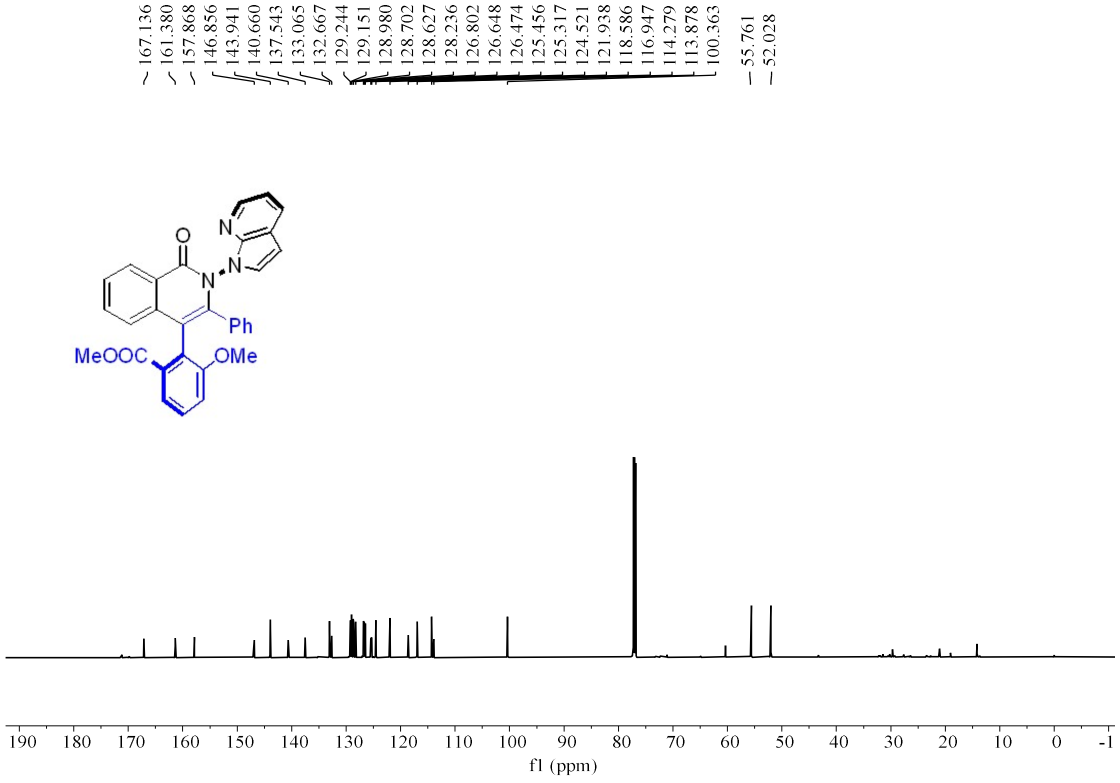


**Supplementary Figure 340.** $^{13}$**C NMR (150 MHz, CDCl**$_3$**) spectrum of dia-80.**

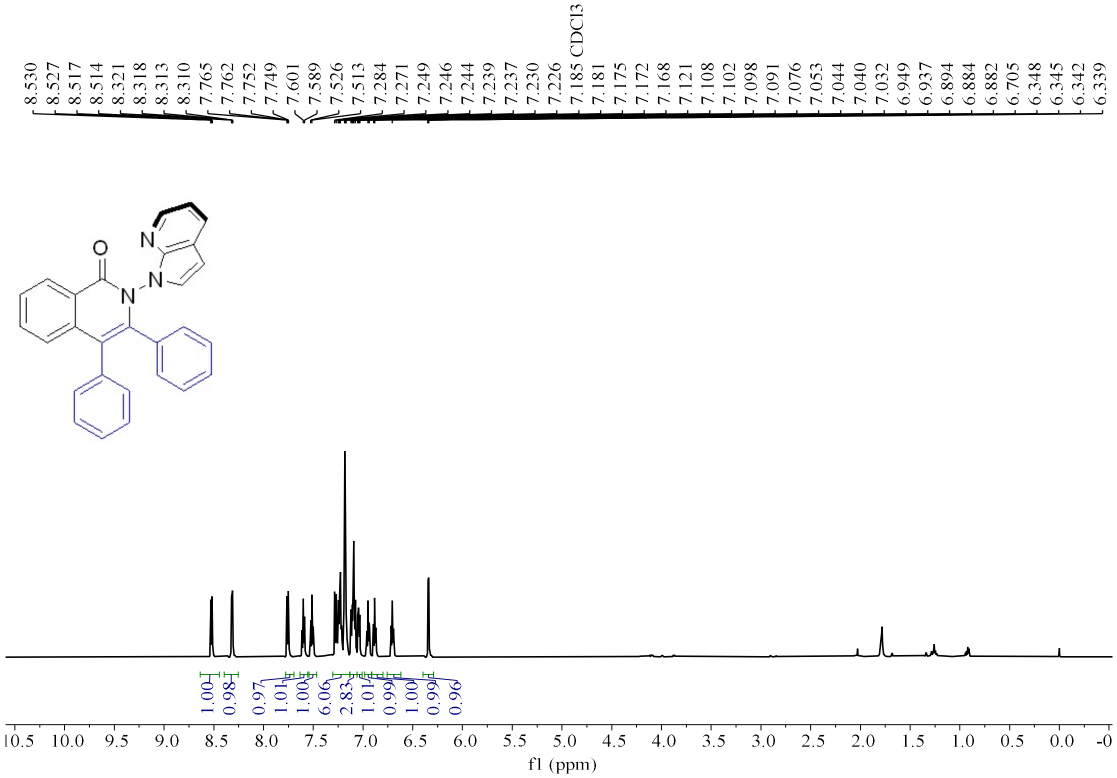


**Supplementary Figure 341. $^{1}$H NMR (600 MHz, CDCl$_3$) spectrum of dia-81.**

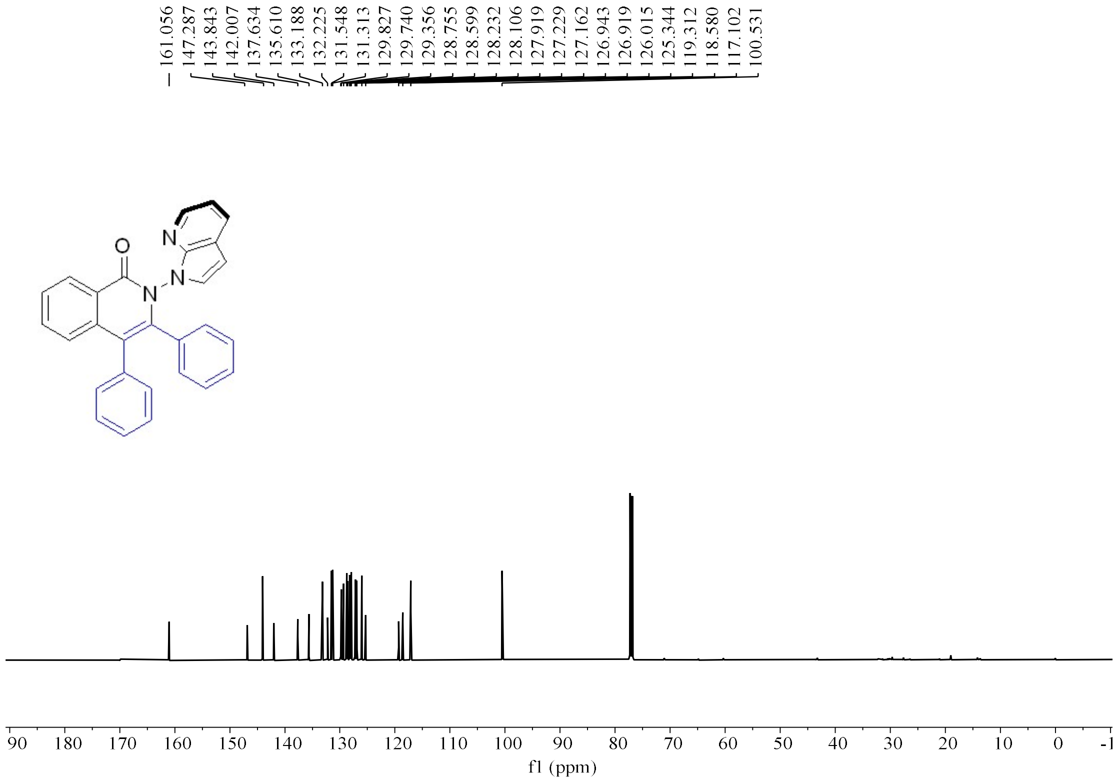


**Supplementary Figure 342. $^{13}$C NMR (150 MHz, CDCl$_3$) spectrum of dia-81.**

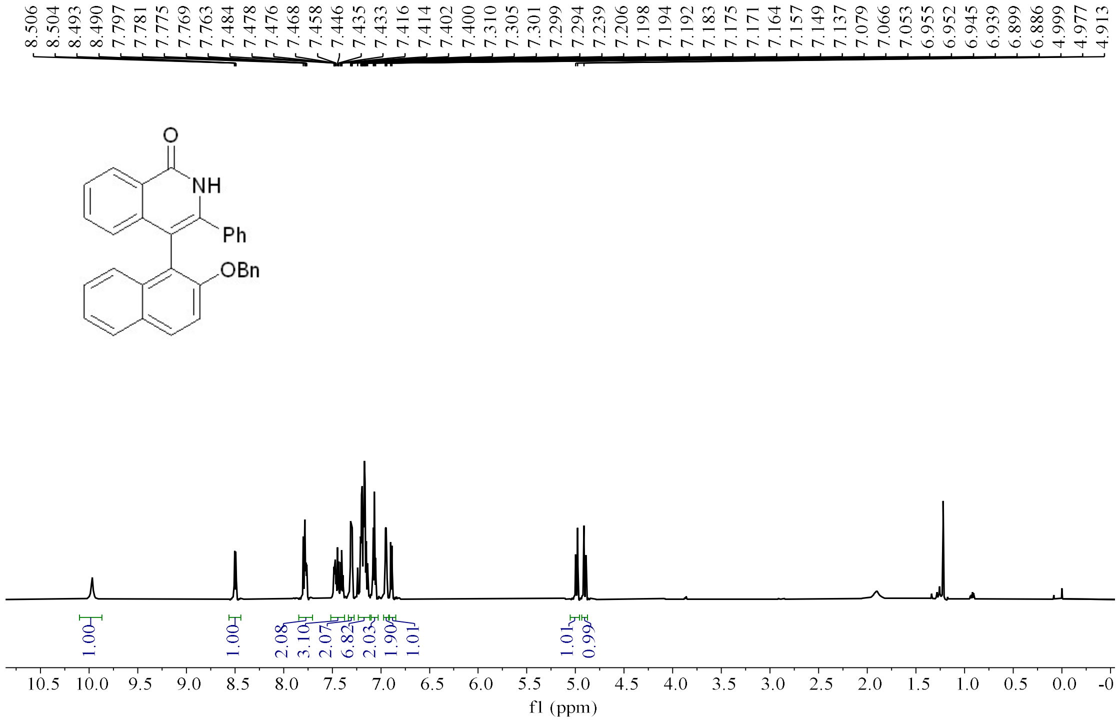


**Supplementary Figure 343.** $^{1}$**H NMR (600 MHz, CDCl$_3$) spectrum of 82.**

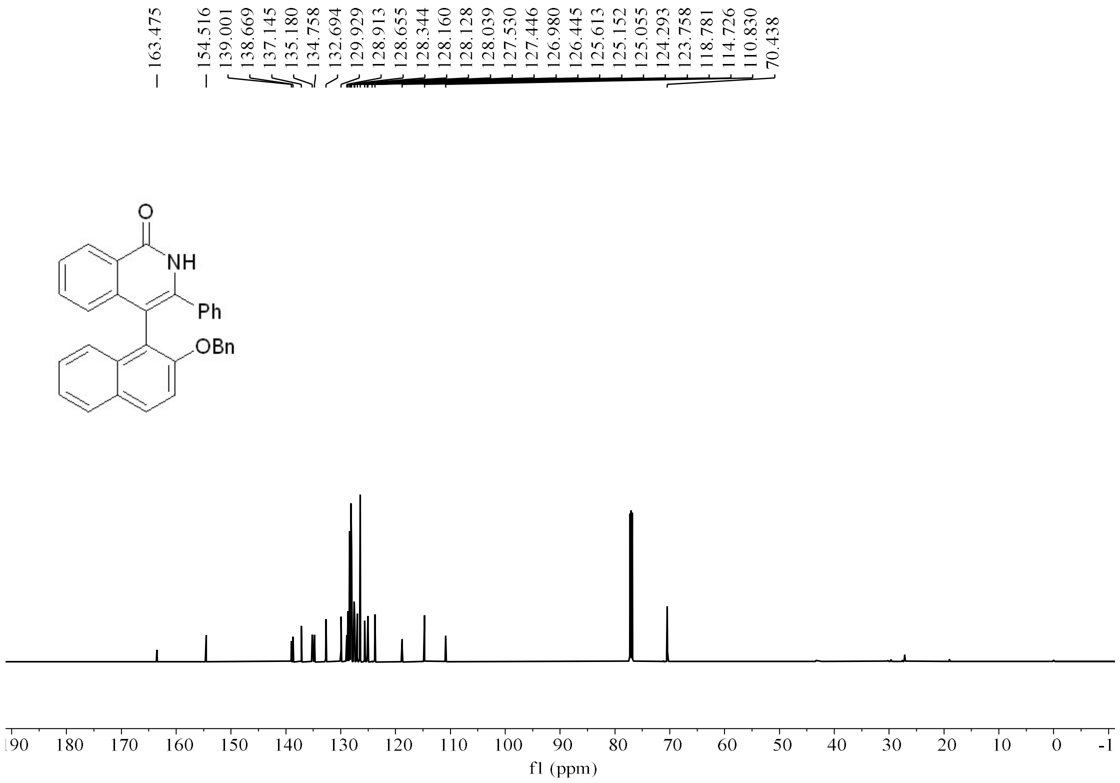


**Supplementary Figure 344.** $^{13}$**C NMR (150 MHz, CDCl$_3$) spectrum of 82.**

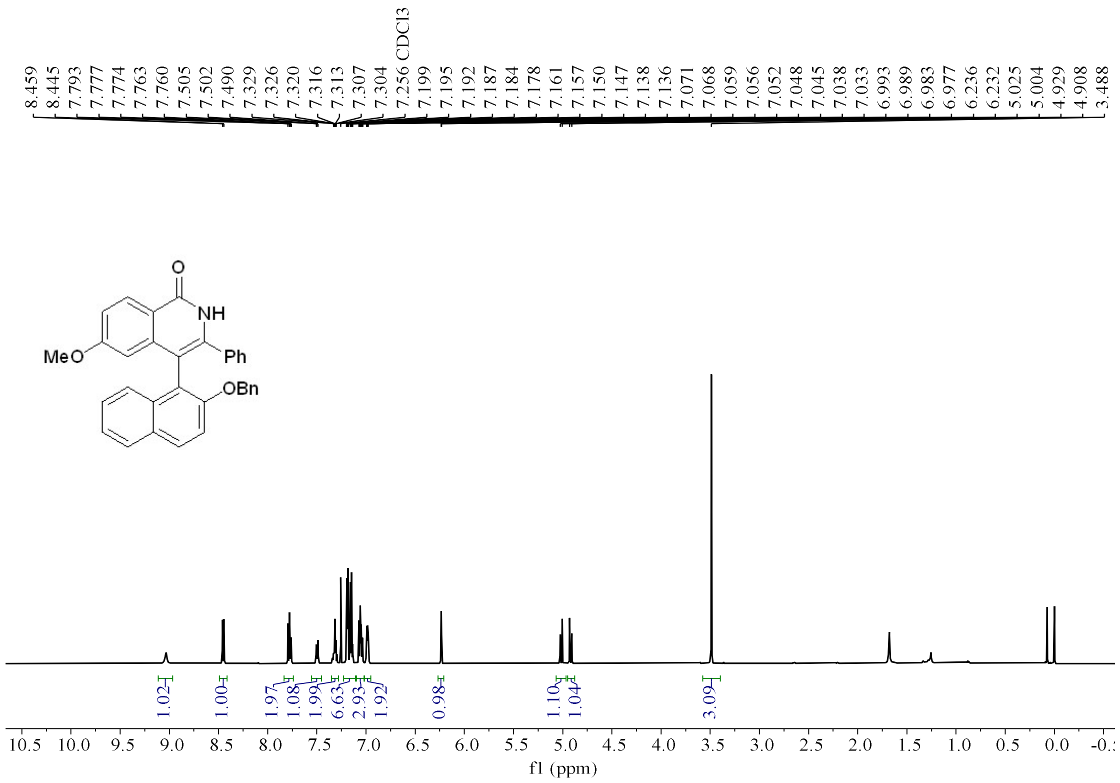

**Supplementary Figure 345. $^{1}$H NMR (600 MHz, CDCl$_3$) spectrum of 83.**

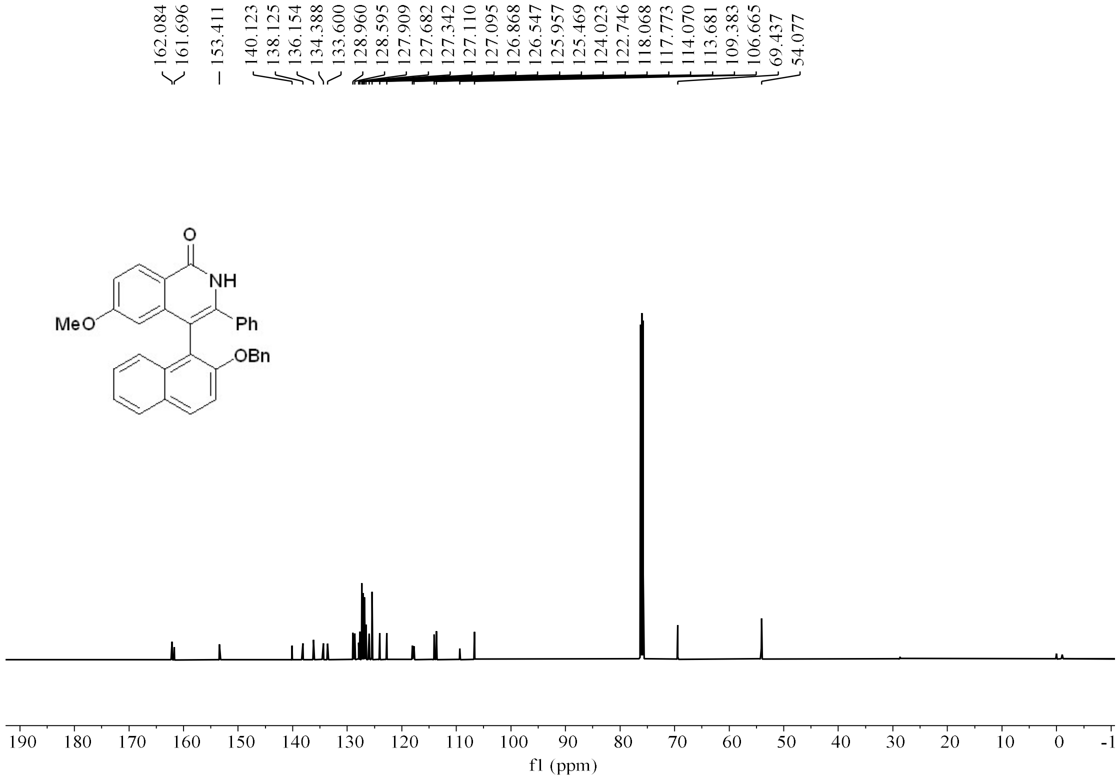

**Supplementary Figure 346. $^{13}$C NMR (150 MHz, CDCl$_3$) spectrum of 83.**

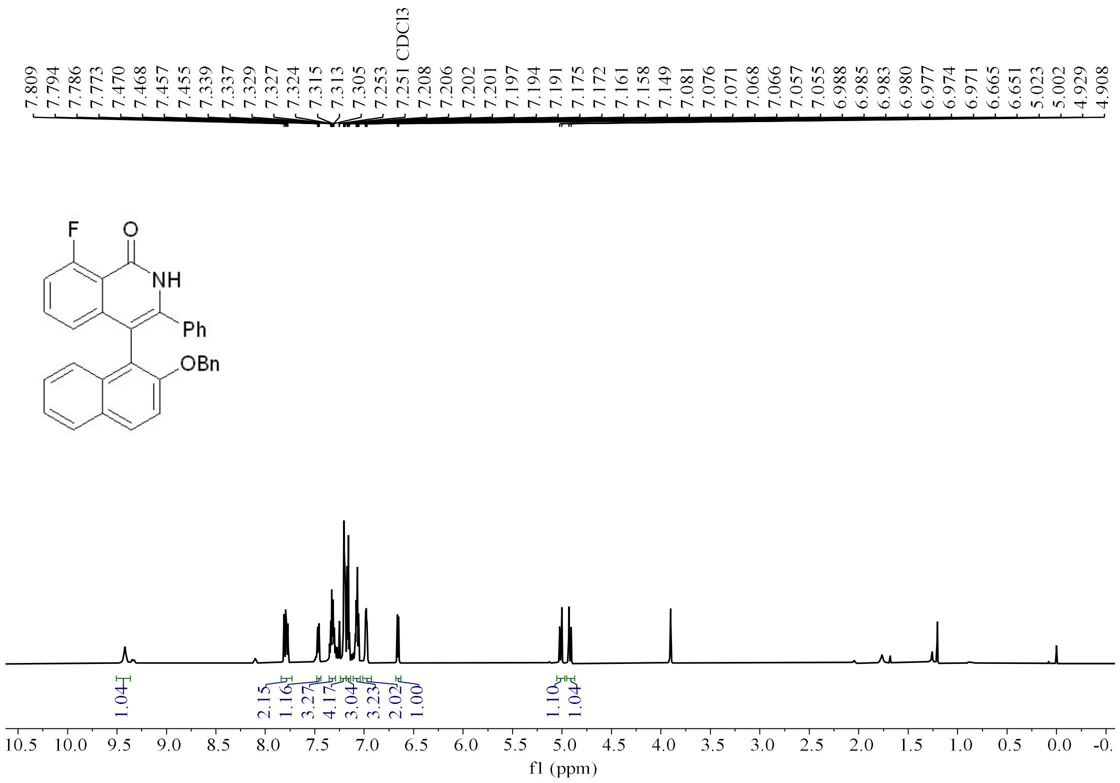


**Supplementary Figure 347. $^{1}$H NMR (600 MHz, CDCl$_3$) spectrum of 84.**

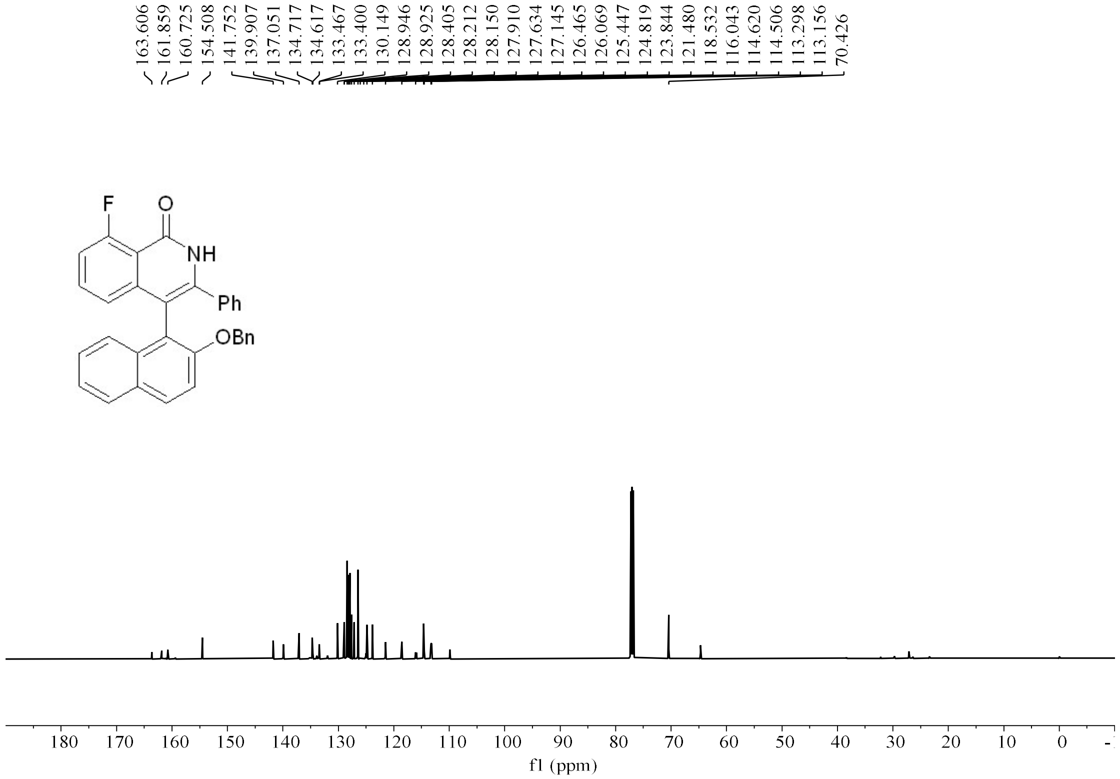


**Supplementary Figure 348. $^{13}$C NMR (150 MHz, CDCl$_3$) spectrum of 84.**

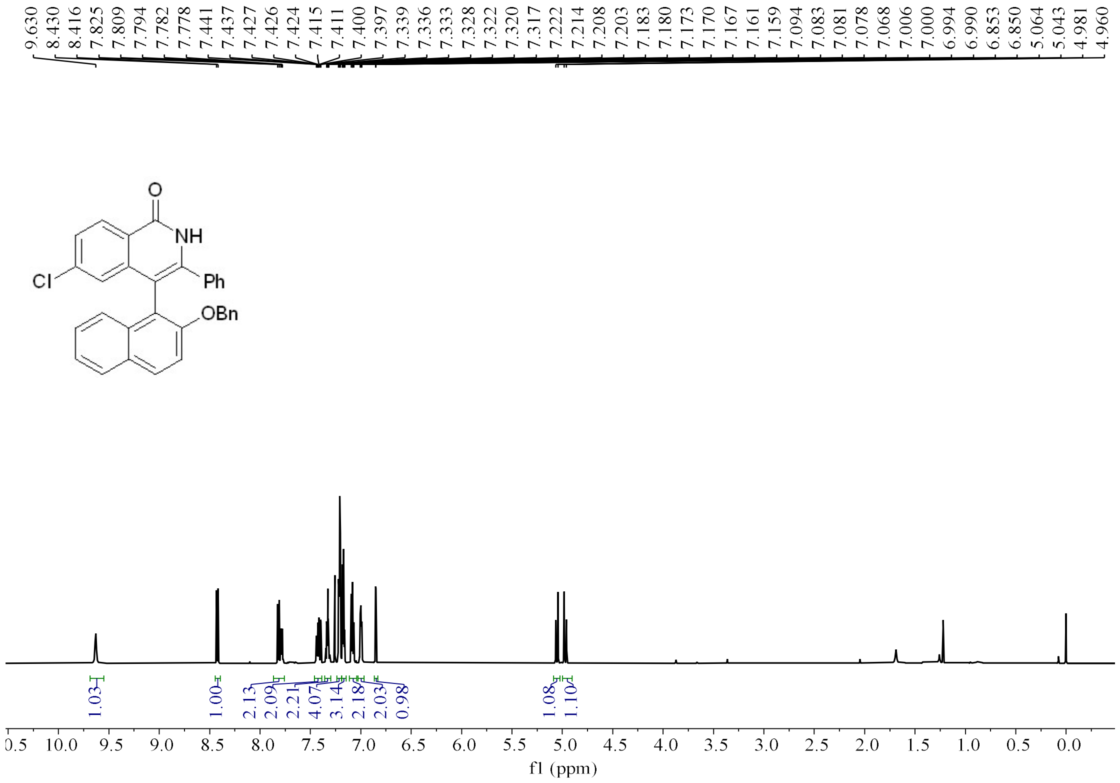


**Supplementary Figure 349.** $^{1}$**H NMR (600 MHz, CDCl**$_3$**) spectrum of 85.**

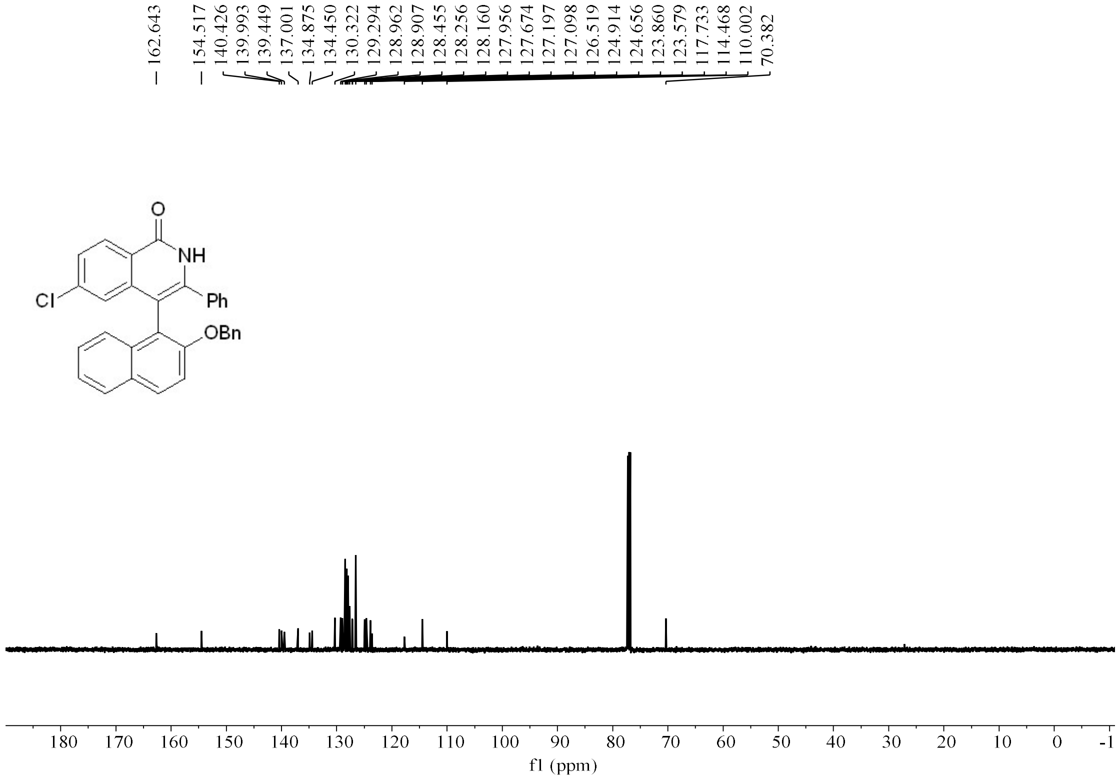


**Supplementary Figure 350.** $^{13}$**C NMR (150 MHz, CDCl**$_3$**) spectrum of 85.**

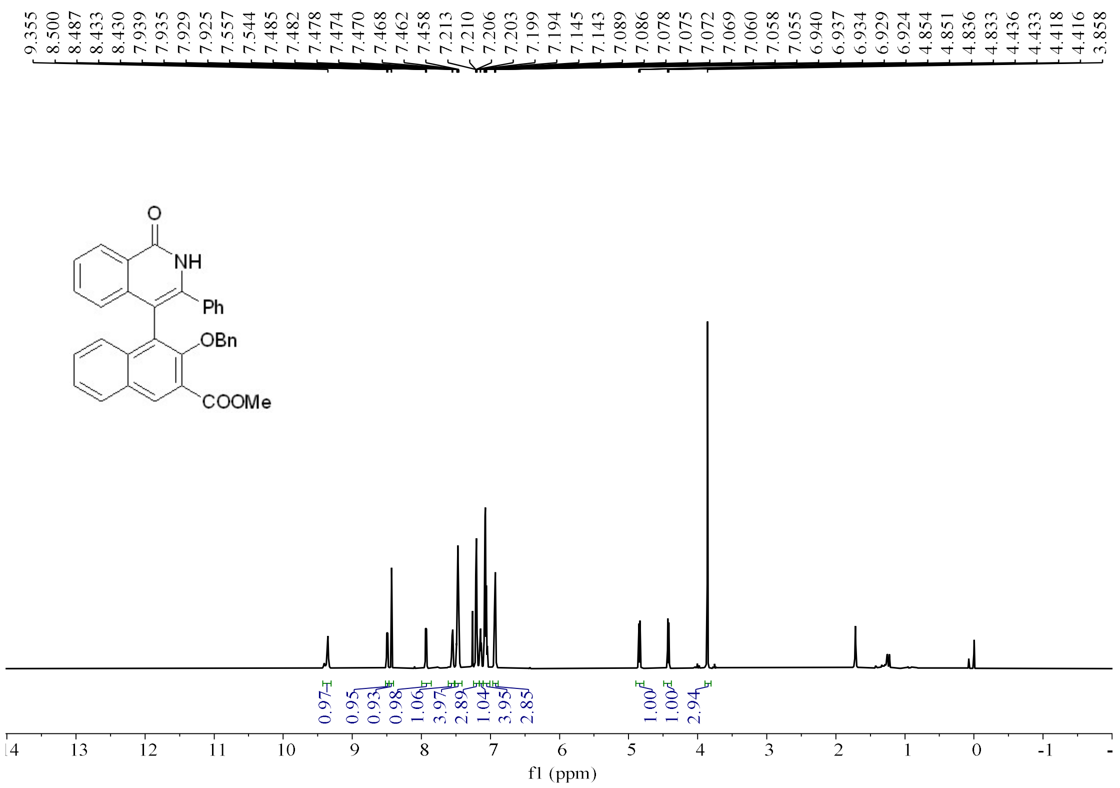


**Supplementary Figure 351.** $^{1}H$ **NMR (600 MHz,** $CDCl_3$**) spectrum of 86.**

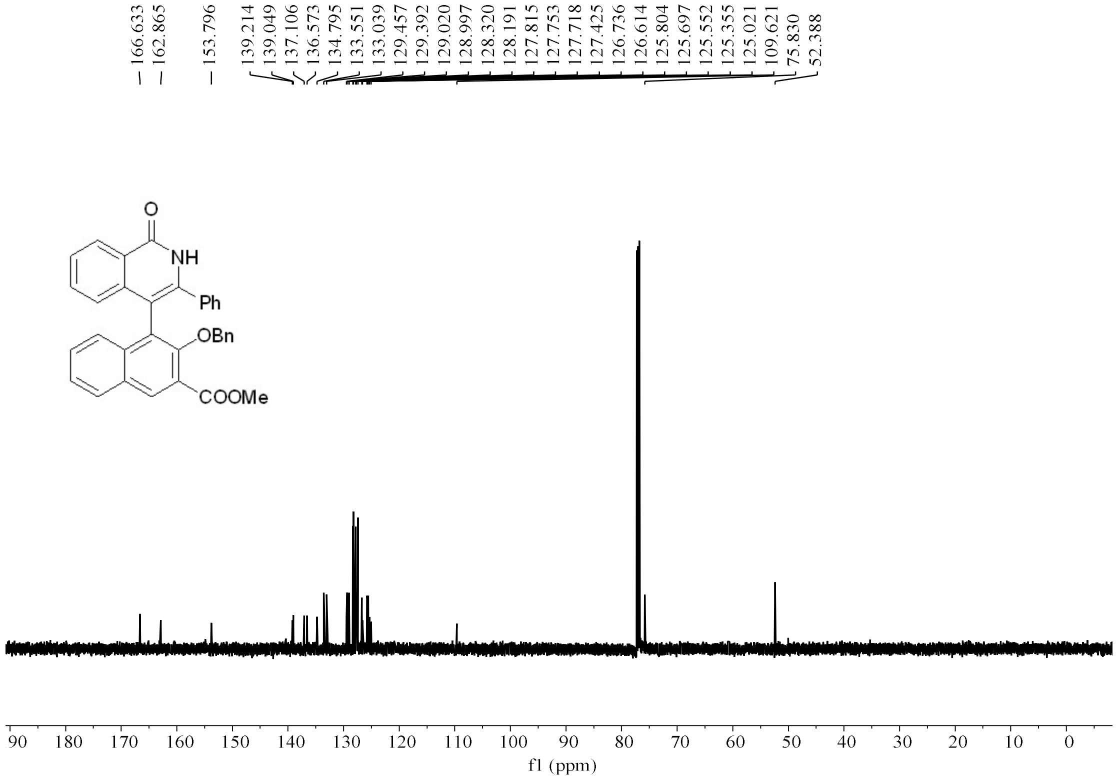


**Supplementary Figure 352.** $^{13}C$ **NMR (150 MHz,** $CDCl_3$**) spectrum of 86.**

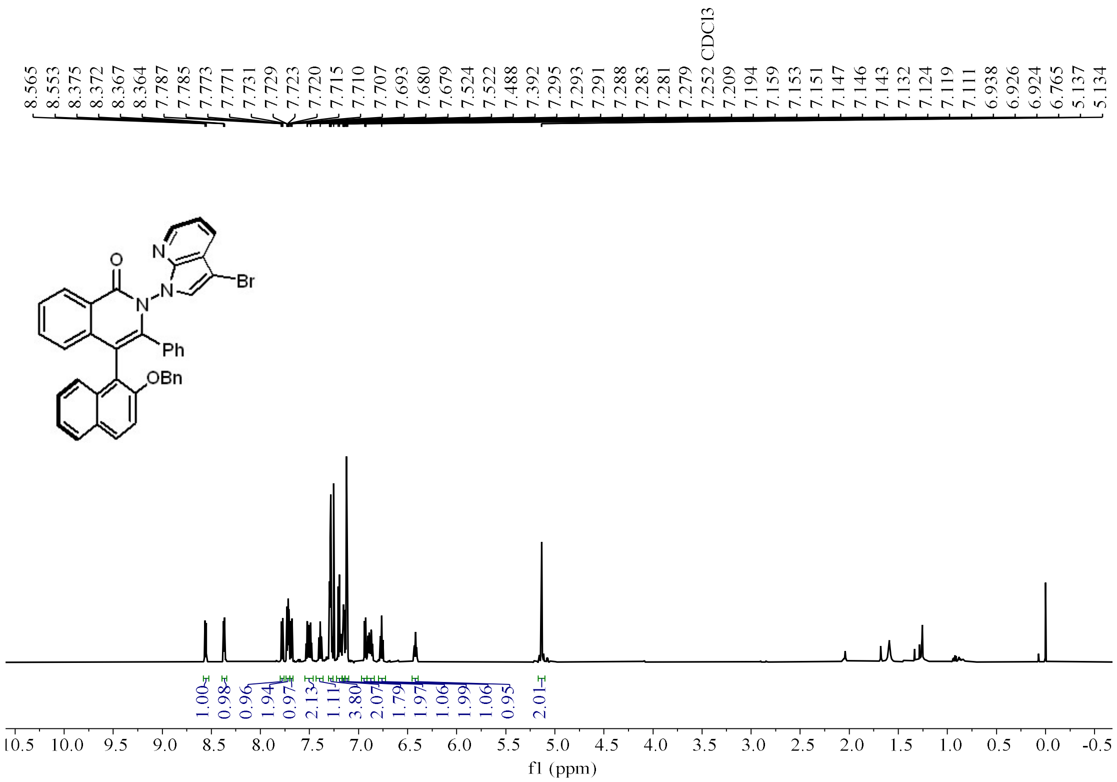


**Supplementary Figure 353. $^{1}$H NMR (600 MHz, $CDCl_3$) spectrum of 87.**

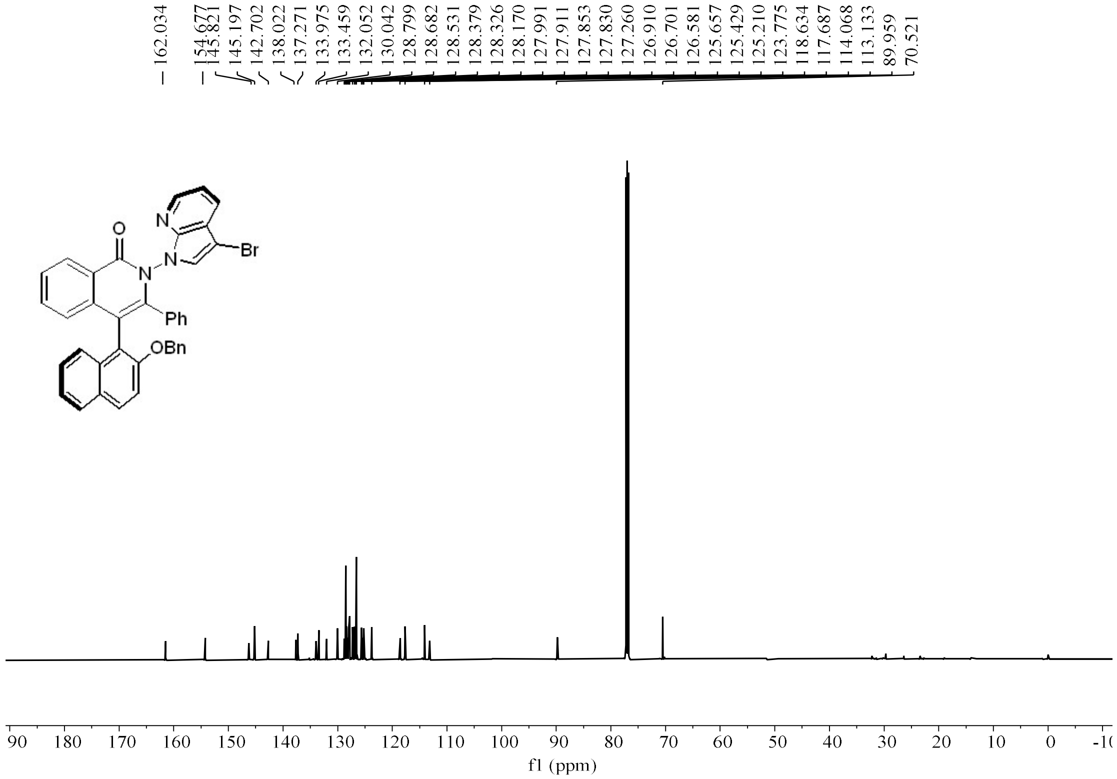


**Supplementary Figure 354. $^{13}$C NMR (150 MHz, $CDCl_3$) spectrum of 87.**

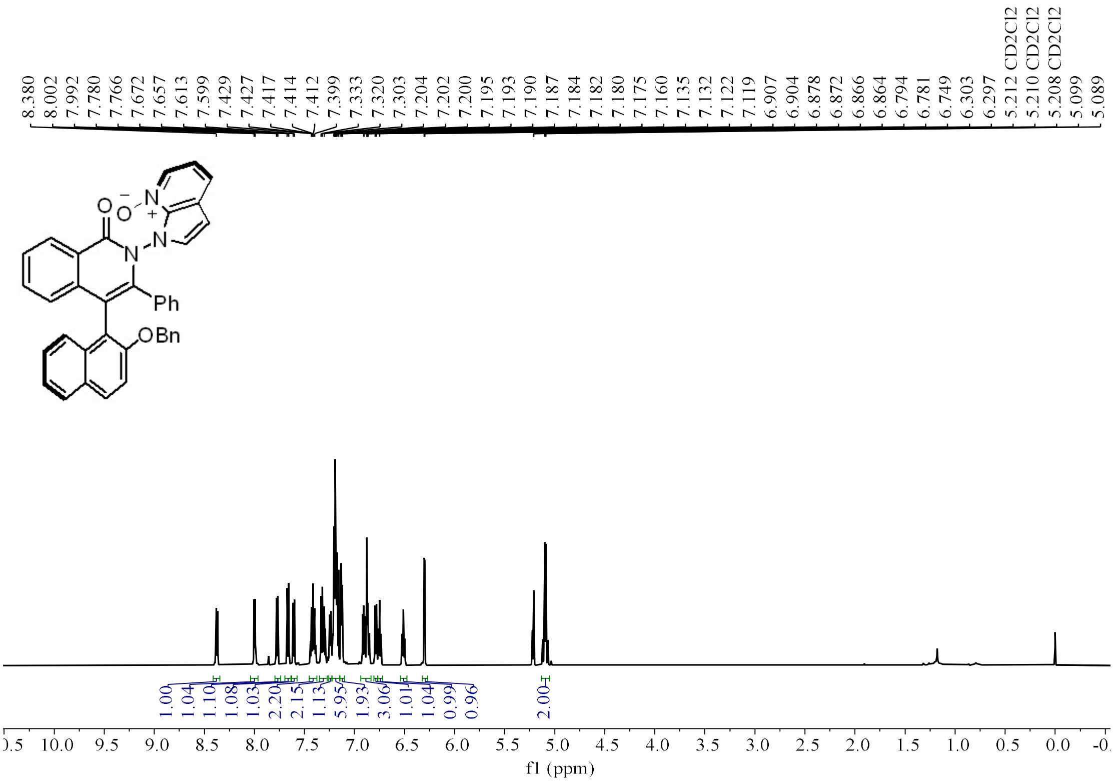


**Supplementary Figure 355. $^{1}$H NMR (600 MHz, $CDCl_3$) spectrum of 88.**

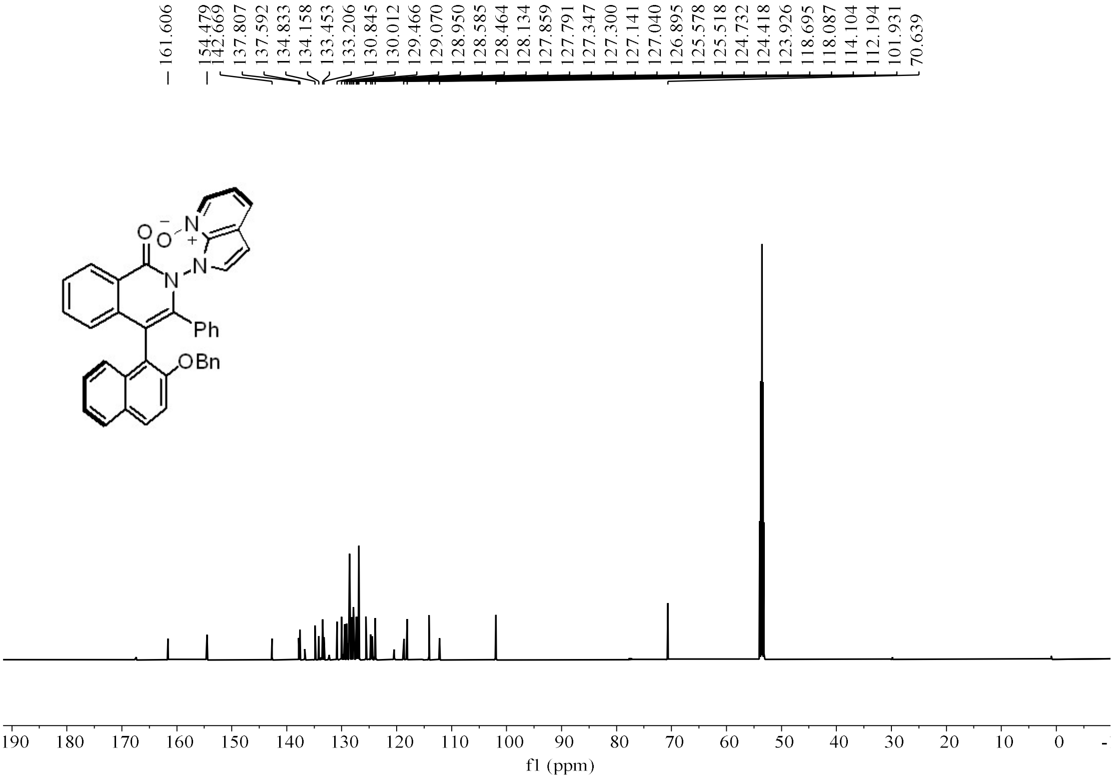


**Supplementary Figure 356. $^{13}$C NMR (150 MHz, $CDCl_3$) spectrum of 88.**

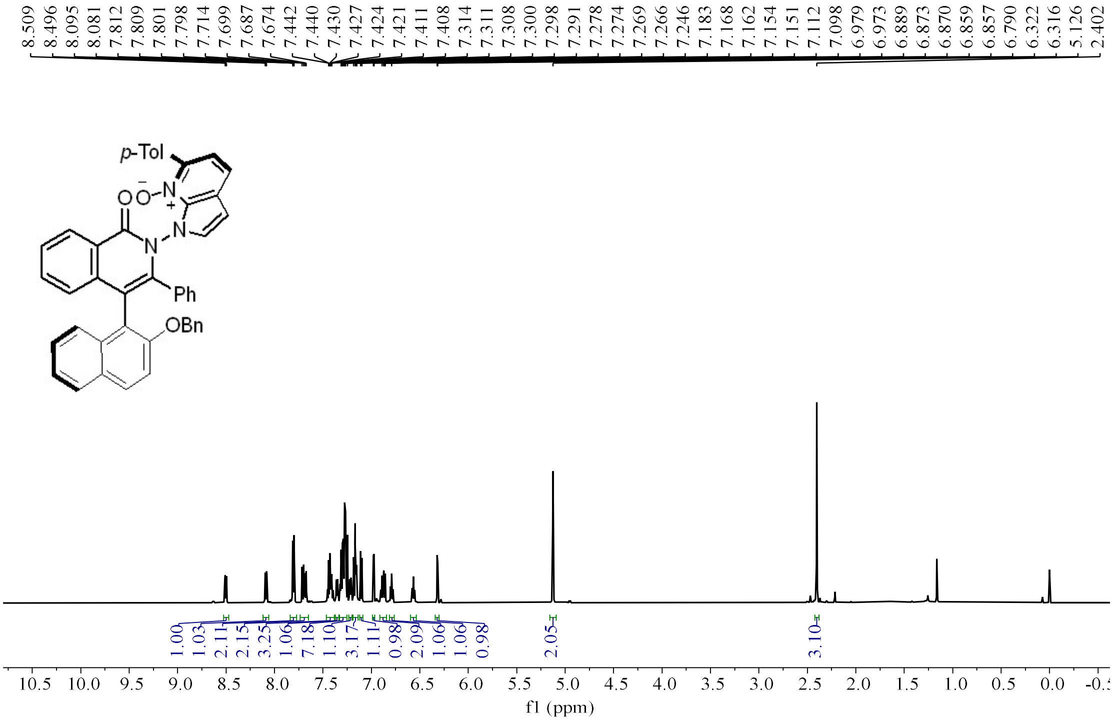


**Supplementary Figure 357.** **$^{1}$H NMR (600 MHz, $CDCl_3$) spectrum of 89.**

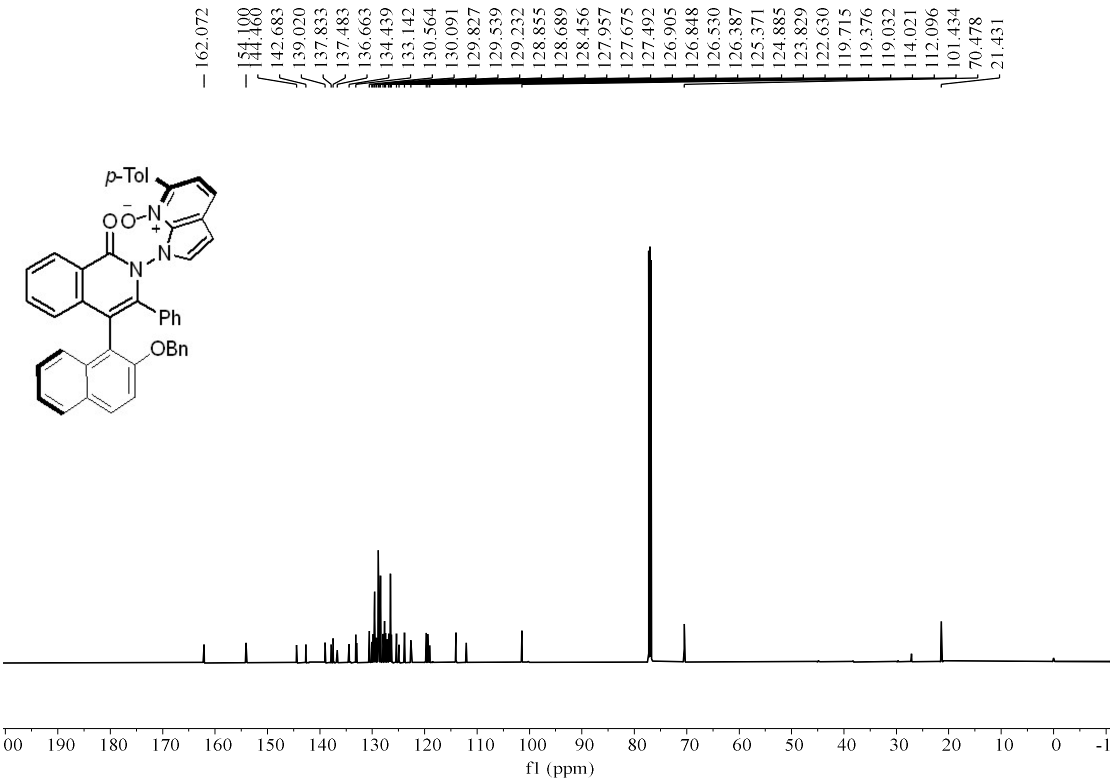


**Supplementary Figure 358.** **$^{13}$C NMR (150 MHz, $CDCl_3$) spectrum of 89.**

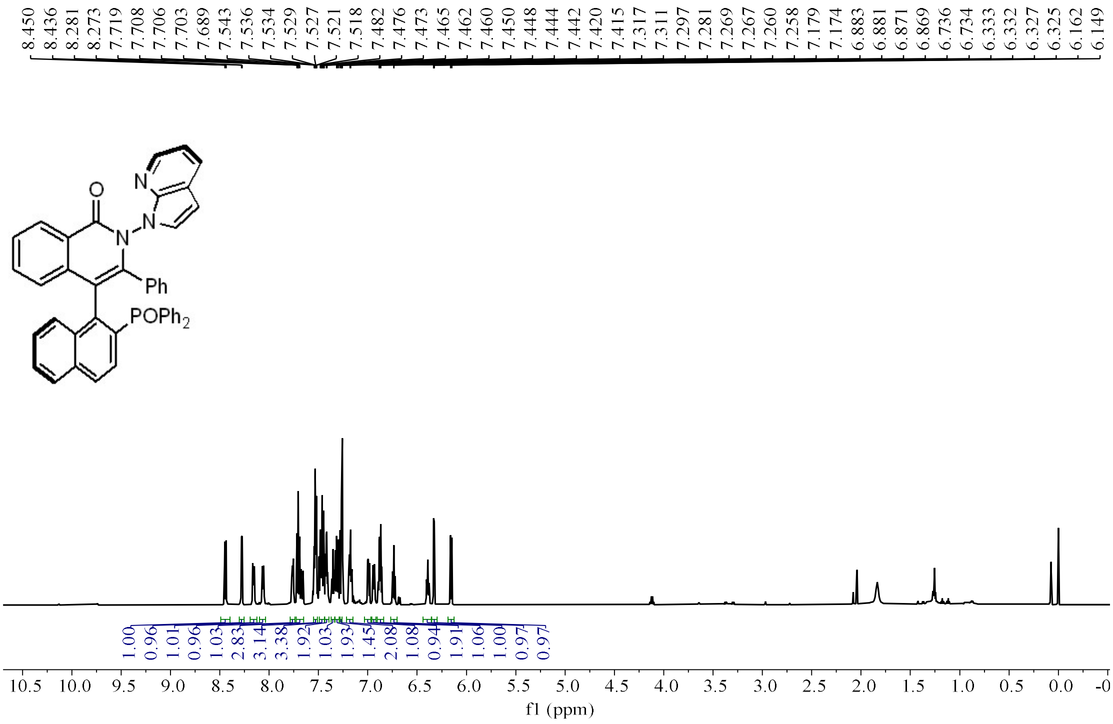


**Supplementary Figure 359. $^{1}$H NMR (600 MHz, $CDCl_3$) spectrum of 90.**

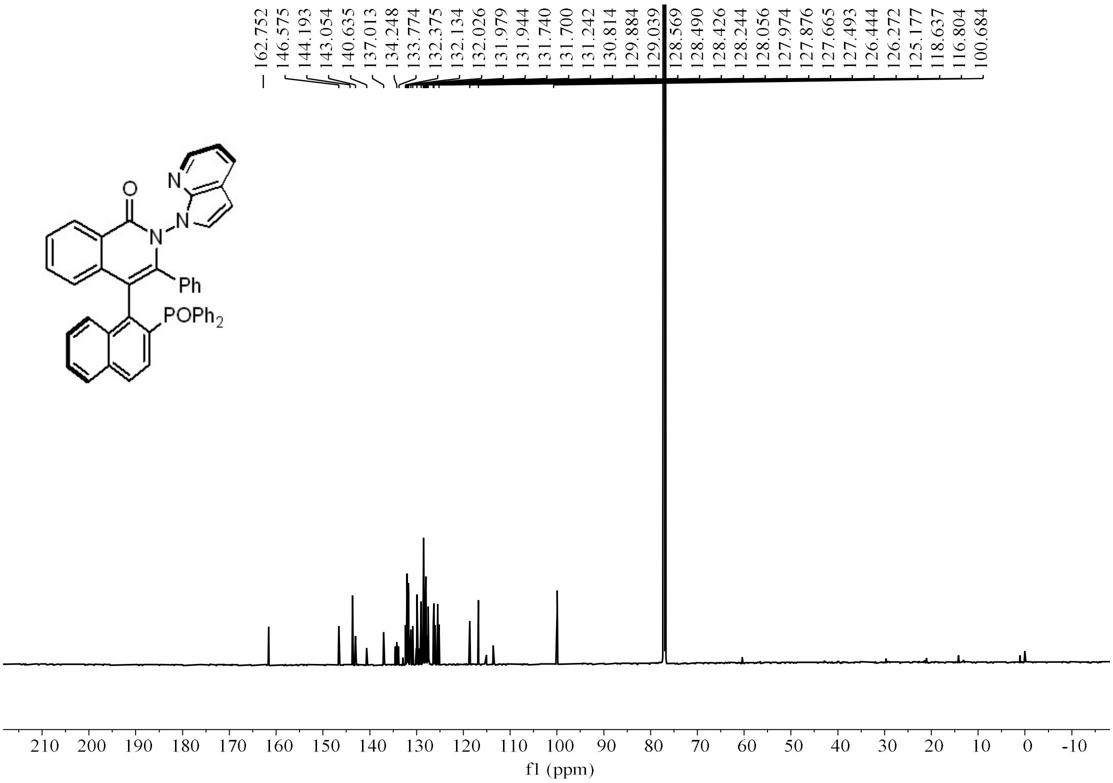


**Supplementary Figure 360. $^{13}$C NMR (150 MHz, $CDCl_3$) spectrum of 90.**

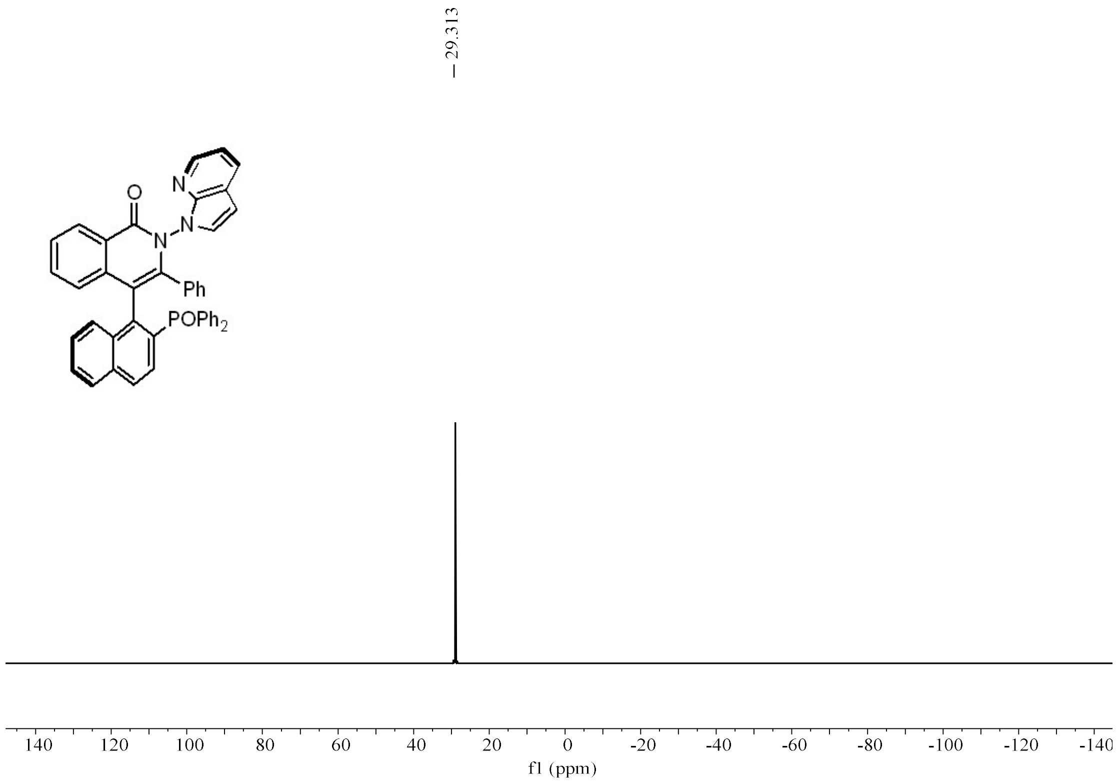


**Supplementary Figure 361. $^{31}$P NMR (243 MHz, $CDCl_3$) spectrum of 90.**

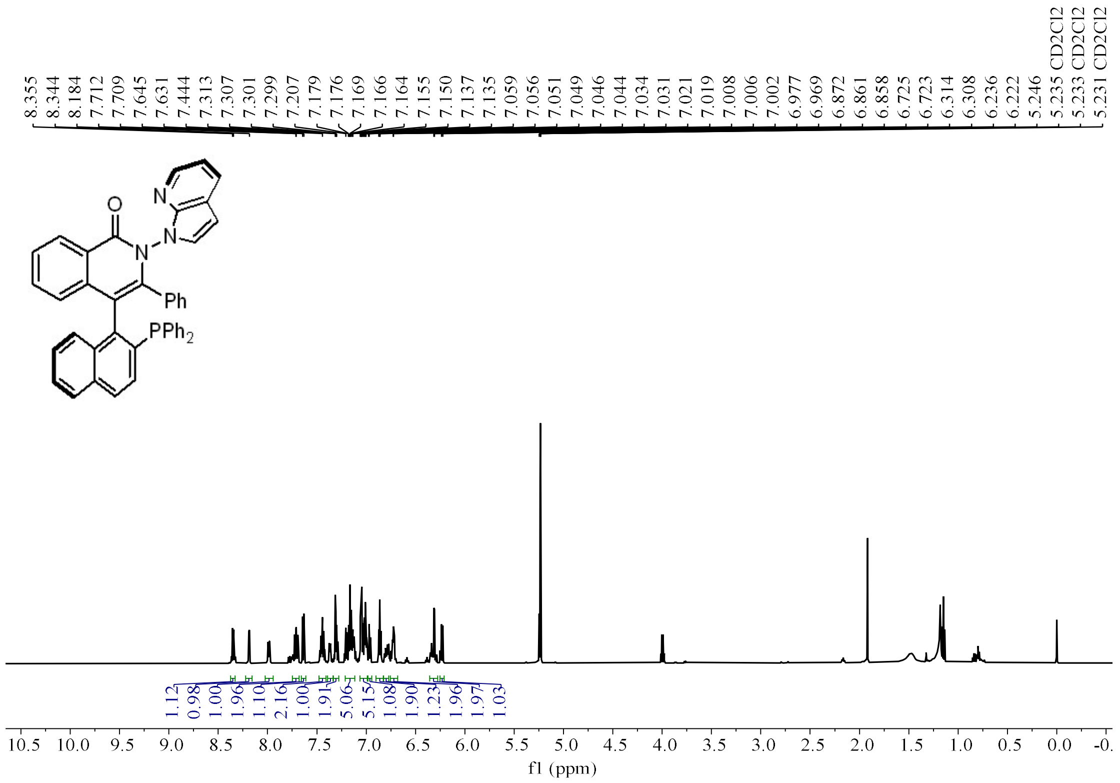


**Supplementary Figure 362. $^{1}$H NMR (600 MHz, $CDCl_3$) spectrum of 91.**

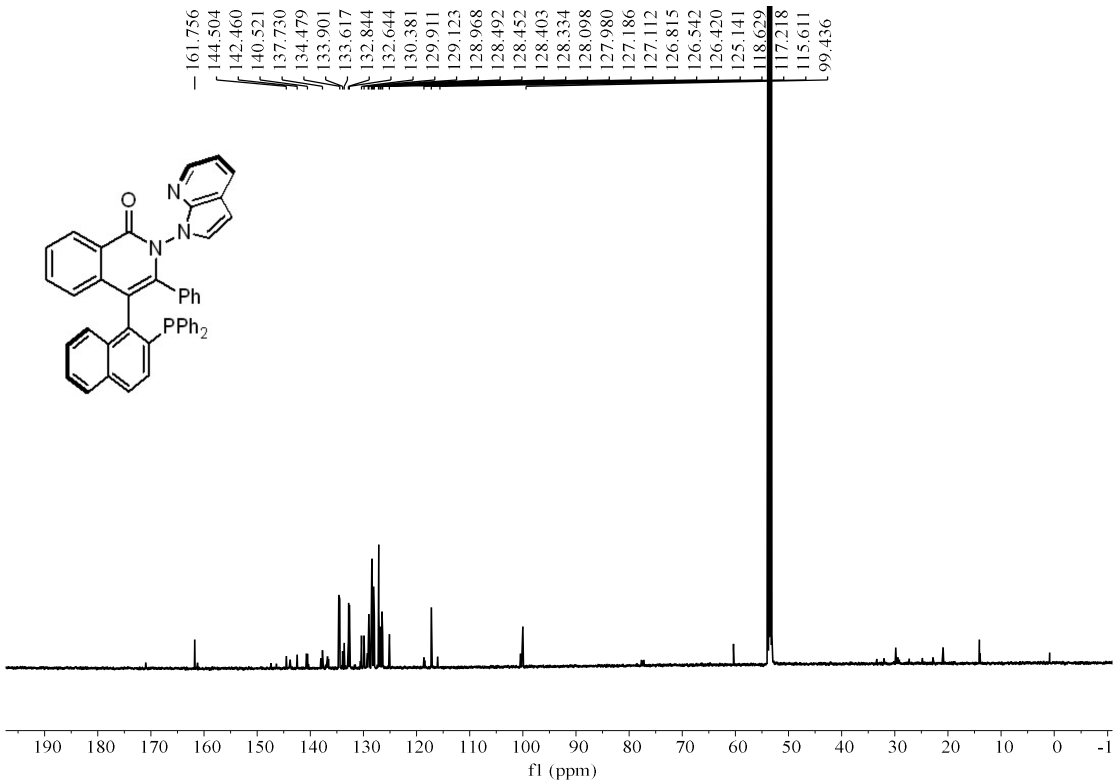


**Supplementary Figure 363. $^{13}$C NMR (150 MHz, $CDCl_3$) spectrum of 91.**

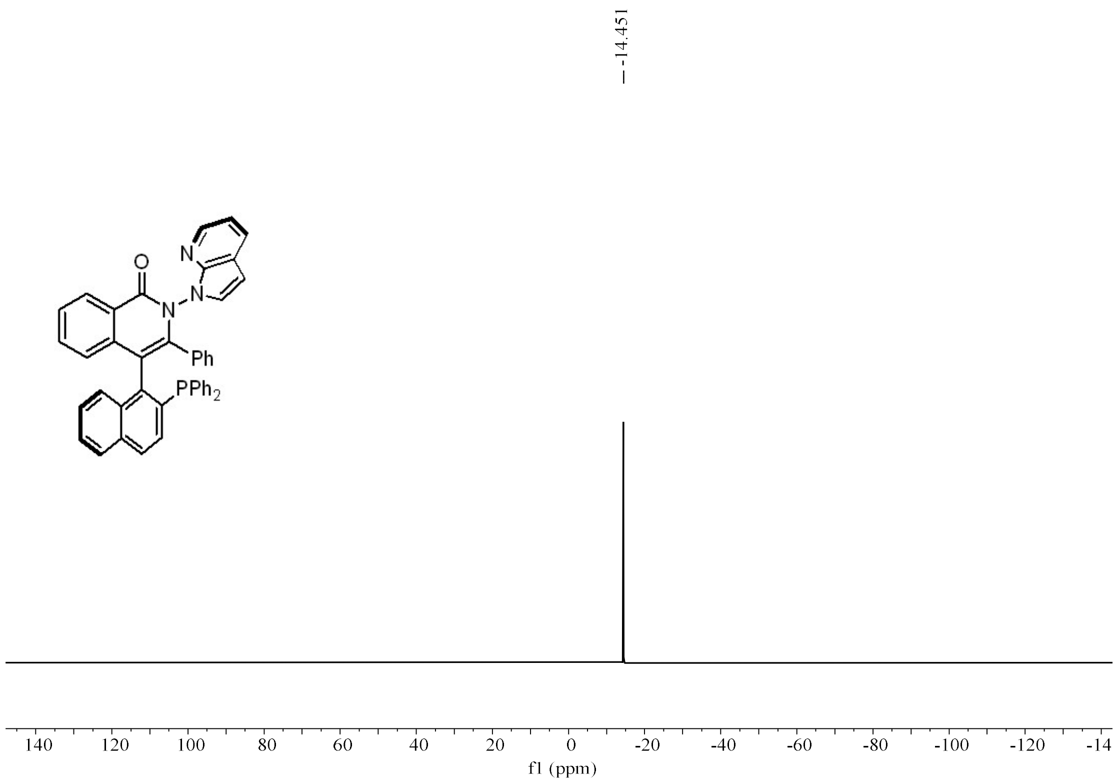


**Supplementary Figure 364. $^{31}$P NMR (243 MHz, CDCl3) spectrum of 91.**

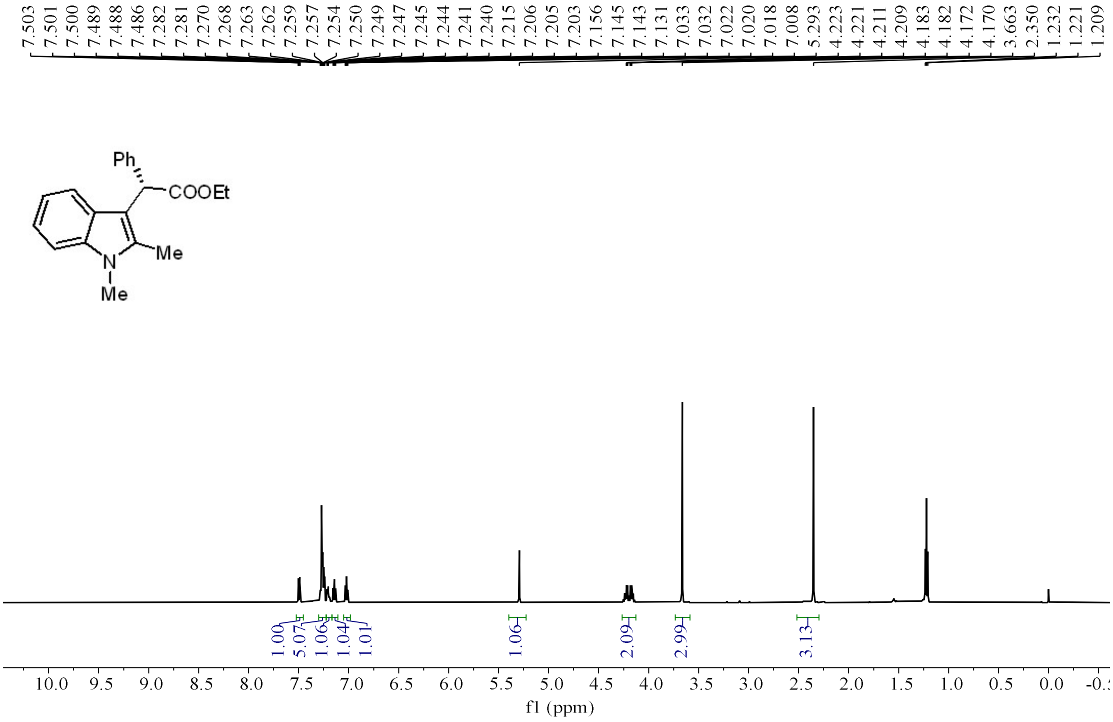


**Supplementary Figure 365. $^{1}$H NMR (600 MHz, $CDCl_3$) spectrum of 92.**

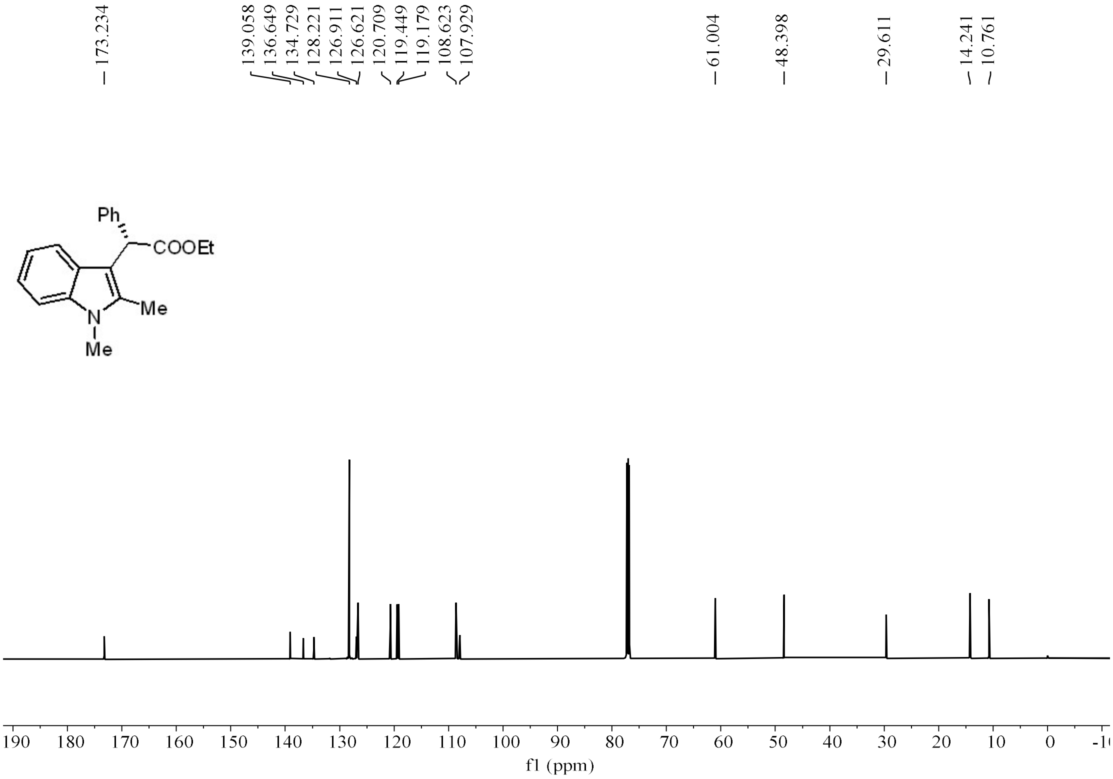


**Supplementary Figure 366. $^{13}$C NMR (150 MHz, $CDCl_3$) spectrum of 92.**

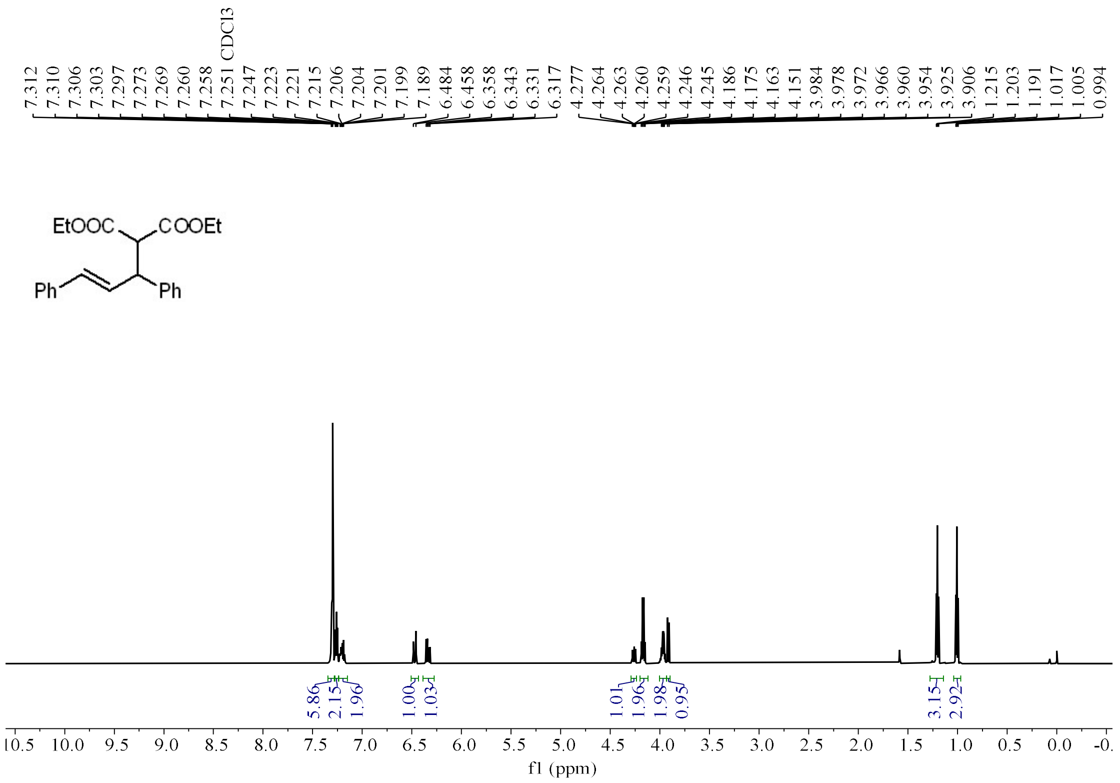

**Supplementary Figure 367. $^{1}$H NMR (600 MHz, $CDCl_3$) spectrum of 93.**

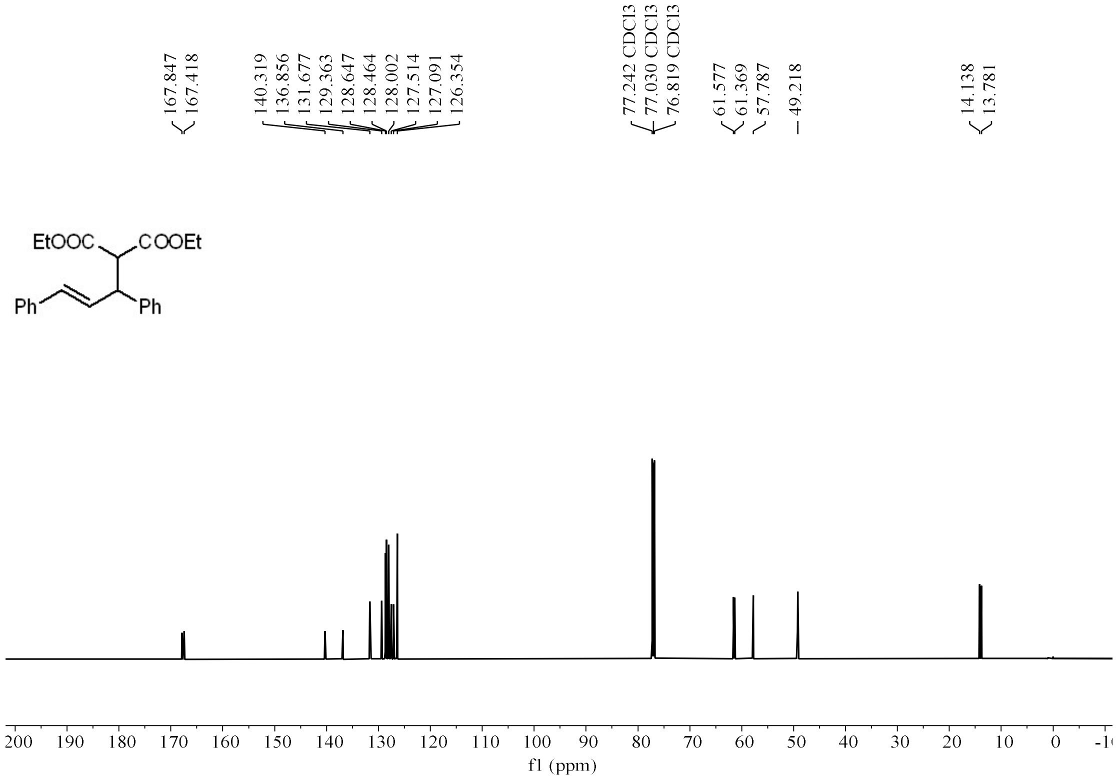

**Supplementary Figure 368. $^{13}$C NMR (150 MHz, $CDCl_3$) spectrum of 93.**

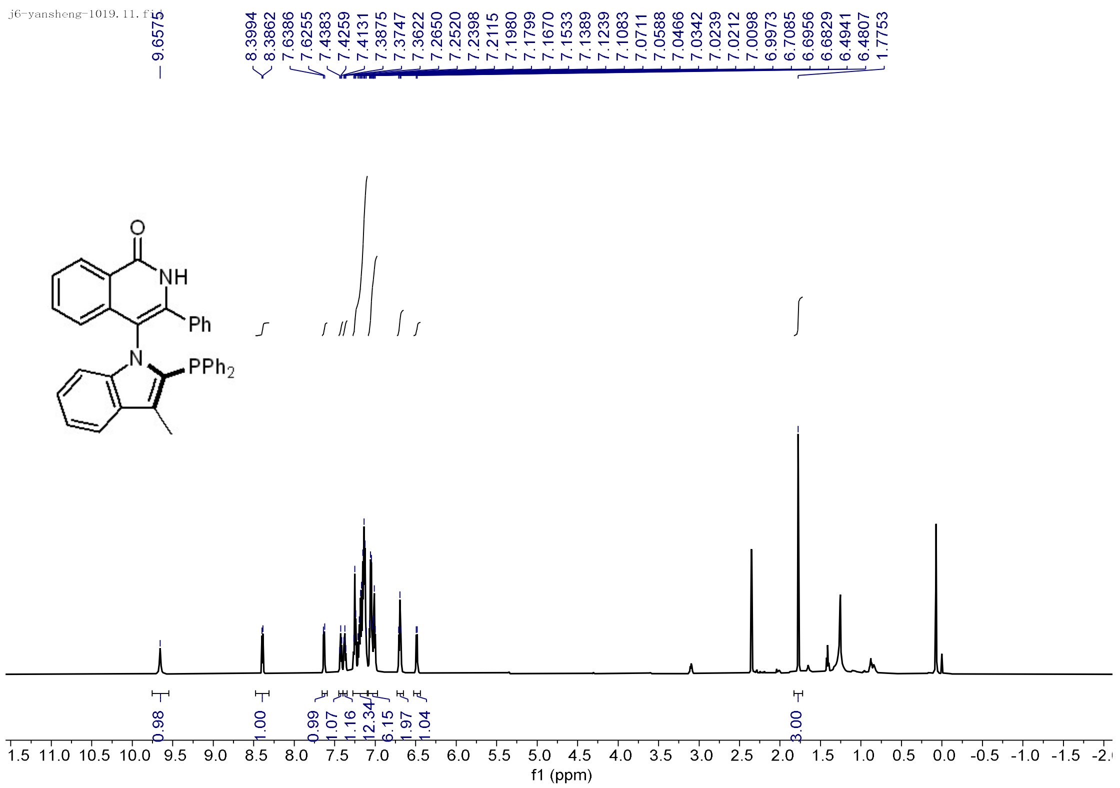


**Supplementary Figure 369. $^{1}$H NMR (600 MHz, $CDCl_3$) spectrum of 94.**

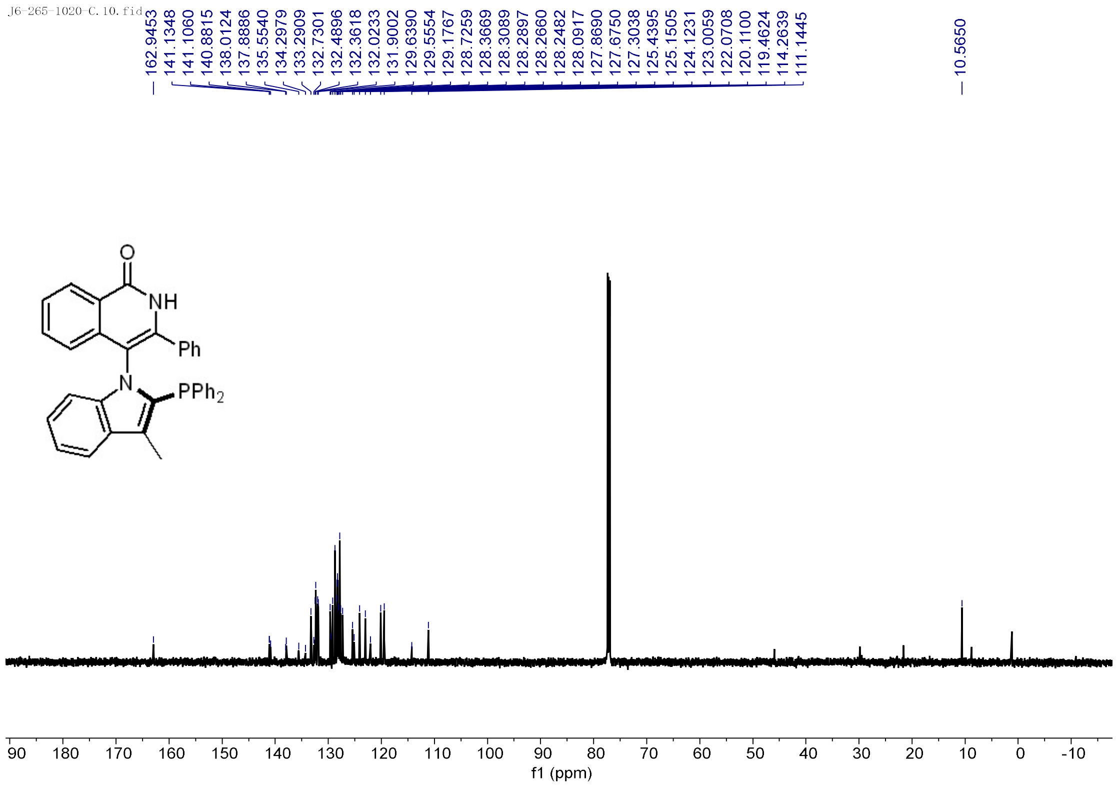


**Supplementary Figure 370. $^{13}$C NMR (150 MHz, $CDCl_3$) spectrum of 94.**

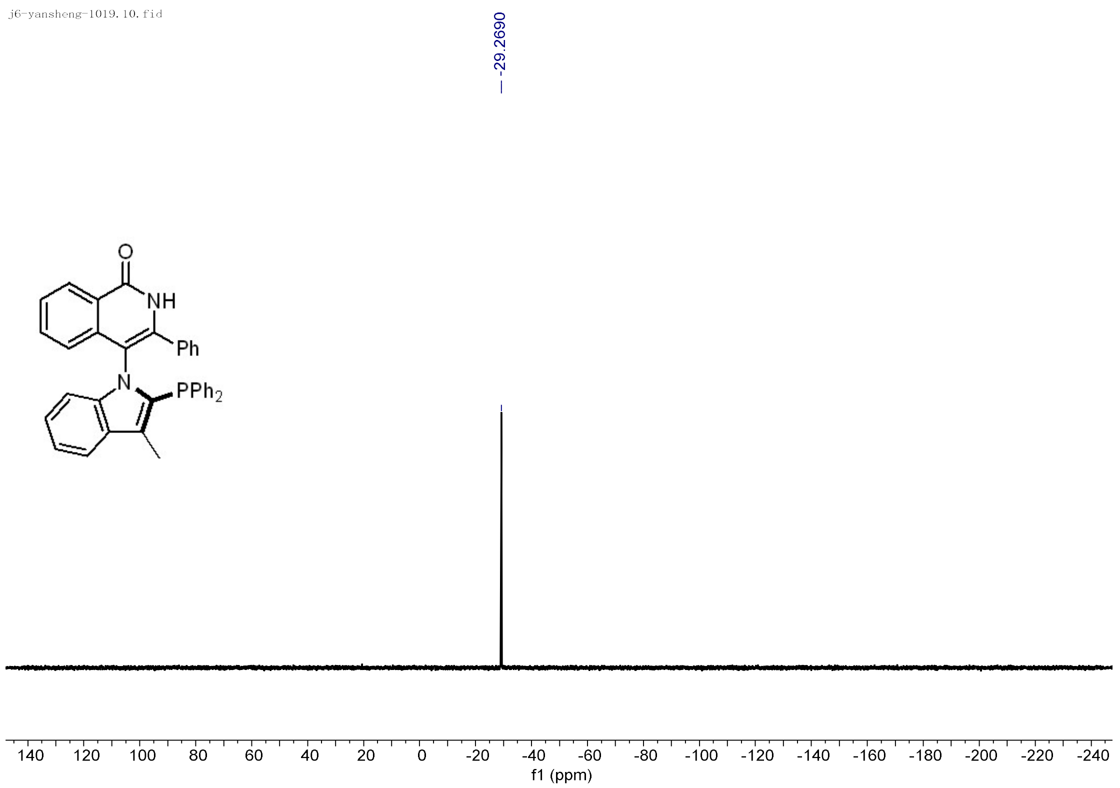


**Supplementary Figure 371. $^{31}$P NMR (243 MHz, $CDCl_3$) spectrum of 94.**

## Supplementary References


(1) (a) Ye, B.; Cramer, N. *J. Am. Chem. Soc.* **2013,** *135*, 636. (b) Ye, B.; Cramer, N. Cramer, *Angew. Chem., Int. Ed.* **2014,** *53*, 7896.

(2) Wang, F.; Jing, J.; Zhao, Y.; Zhu, X.; Zhang, X.; Zhao, L.; Hu, P.; Deng, W.-Q.; Li, X. Angew.Chem. Int. Ed. **2021**, *60*, 16628.

(3) Wang, F.; Qi, Z.; Zhao, Y.; Zhai, S.; Zheng, G.; Mi, R.; Huang, Z.; Zhu, X.; He, X.; Li, X. *Angew. Chem. Int. Ed.* **2020**, *59*, 13288.

(4) Campeau, L.-C.; Rousseaux, S.; Fagnou, K. J. *Am. Chem. Soc*. **2005**, *127*, 18020.

(5) Si, X.-J.; Yang, D.; Sun, M.-C.; Wei, D.; Song, M.-P.; Niu, J. *Nat. Synth*. **2022**, *1,* 709.

(6) (a) Palvoelgyi, A. M.; Schnuerch, M.; Bica-Schroeder, K. *Tedrehedron* **2020**, *76*, 131246. (b) Li, Z.; Chen, Y.; Wang, C.; Xu, G.; Shao, Y.; Zhang, X.; Tang, S.; Sun, J. *Angew. Chem. Int. Ed.* **2021**, *60*, 25714.

(7) El Baba, S.; Sartor, K.; Poulin, J.-C.; Kagan, H. B. *Bull. Soc. Chim. Fr.* **1994**, 525-533

(8) (a) A.D. Becke, J. Chem. Phys. 1993, 98, 5648; (b) P. J. Stephens, F. J. Devlin, C. F. Chabalowski, M. J. Frisch, J. Phys. Chem. 1994, 98, 11623; (c) S. Grimme, J. Antony, S. Ehrlich, H. Krieg, J. Chem. Phys. 2010, 132, 154104; (d) S. Grimme, S. Ehrlich, L. Goerigk, J. Comput. Chem. 2011, 32, 1456.

(9) F. Weigend, R. Ahlrichs, Phys. Chem. Chem. Phys. 2005, 7, 3297-3305.

(10) Gaussian 09, Revision E.01, M. J. Frisch, G. W. Trucks, H. B. Schlegel, G. E. Scuseria, M. A. Robb, J. R. Cheeseman, G. Scalmani, V. Barone, B. Mennucci, G. A. Petersson, H. Nakatsuji,

M. Caricato, X. Li, H. P. Hratchian, A. F. Izmaylov, J. Bloino, G. Zheng, J. L. Sonnenberg, M. Hada, M. Ehara, K. Toyota, R. Fukuda, J. Hasegawa, M. Ishida, T. Nakajima, Y. Honda, O. Kitao, H. Nakai, T. Vreven, J. A. Montgomery, Jr., J. E. Peralta, F. Ogliaro, M. Bearpark, J. J. Heyd, E. Brothers, K. N. Kudin, V. N. Staroverov, R. Kobayashi, J. Normand, K. Raghavachari, A. Rendell, J. C. Burant, S. S. Iyengar, J. Tomasi, M. Cossi, N. Rega, J. M. Millam, M. Klene, J. E. Knox, J. B. Cross, V. Bakken, C. Adamo, J. Jaramillo, R. Gomperts, R. E. Stratmann, O. Y azyev, A. J. Austin, R. Cammi, C. Pomelli, J. W. Ochterski, R. L. Martin, K. Morokuma, V. G. Zakrzewski, G. A. V oth, P. Salvador, J. J. Dannenberg, S. Dapprich, A. D. Daniels, Ö. Farkas, J. B. Foresman, J. V. Ortiz, J. Cioslowski, D. J. Fox, Gaussian, Inc., Wallingford CT, 2013.
(11)A. V. Marenich, C. J. Cramer, D. G. Truhlar, J. Phys. Chem. B. 2009, 113, 6378.
(12) (a) K. Fukui, J. Phys. Chem. 1970, 74, 4161; (b) K. Fukui, Acc. Chem. Res. 1981, 14, 363.
(13) (a) L. Goerigk, S. Grimme, J. Chem. Theory Comput. 2011, 7, 291; (b) E. Caldeweyher, C. Bannwarth, S. Grimme, J. Chem. Phys. 2017, 147, 034112.
(14) F. Weigend, R. Ahlrichs, Phys. Chem. Chem. Phys. 2005, 7, 3297-3305.
(15) (a) F. Neese, WIREs Comput. Mol. Sci. 2012, 2, 73-78; (b) F. Neese, F. Wennmohs, U. Becker, C. Riplinger, J. Chem. Phys. 2020, 152, 224108; (c) F. Neese, WIREs Comput. Mol. Sci, 2017, 8, e1327;(d) F. Neese, WIREs Comput. Mol. Sci. 2022,12, e1606.
(16) F. Weigend, Phys. Chem. Chem. Phys. 2006, 8, 1057.
(17) A. Hellweg, C. Hattig, S. Hofener, W. Klopper, Theor. Chem. Acc. 2007, 117, 587.
