# Supplementary material for: Rhodium-catalyzed enantioselective and diastereodivergent access to diaxially chiral heterocycles
Source: Nat Commun. 2023 Aug 3;14:4661. doi: 10.1038/s41467-023-39968-3 (PMC10400608; doi:10.1038/s41467-023-39968-3)
Supplement: Supplementary file 3 — Description of Additional Supplementary Files [file 41467_2023_39968_MOESM3_ESM.docx]

Files Names: Supplementary Data 1

Description: Date of Cartesian coordinates 23
