# Supplementary material for: Rhodium-catalyzed enantioselective and diastereodivergent access to diaxially chiral heterocycles
Source: Nat Commun. 2023 Aug 3;14:4661. doi: 10.1038/s41467-023-39968-3 (PMC10400608; doi:10.1038/s41467-023-39968-3)
Supplement: Supplementary file 4 — Supplementary Data 1 [file 41467_2023_39968_MOESM4_ESM.pdf]

Cartesian coordinates

23

C	-4.55326800	-0.58821100	1.21840300
C	-3.17699000	-0.61429800	1.29436100
C	-2.43102300	0.59215100	1.38086400
C	-3.13682400	1.84038400	1.38824100
C	-4.55719800	1.83091900	1.30789800
C	-5.25346900	0.64503600	1.22487900
H	-5.11164200	-1.52517700	1.15243400
H	-2.63511100	-1.56212300	1.28948900
C	-1.00211900	0.59385200	1.46078100
C	-2.39613800	3.04736500	1.47539000
H	-5.08764800	2.78682100	1.31369300
H	-6.34418100	0.65031500	1.16402300
C	-1.01747800	3.04381800	1.55321900
C	-0.31277400	1.81724800	1.54617900
H	-2.93621500	3.99745400	1.48057500
H	-0.47466800	3.98684500	1.61906500
C	-0.21328300	-0.58588900	1.45832300
C	0.63080100	-1.46599500	1.46907600
C	1.74611000	-2.35143700	1.49464200
C	3.04162600	-1.79439900	1.60198200
C	1.60873100	-3.75197000	1.41639600
C	4.16103600	-2.62198400	1.63001200
H	3.14794700	-0.71023200	1.66207300
C	2.73712400	-4.57096100	1.44491400
H	0.61058200	-4.18619700	1.33314200
C	4.01491900	-4.01191000	1.55164600
H	5.15486800	-2.17552500	1.71356500
H	2.61924200	-5.65562700	1.38354800
H	4.89520700	-4.65885400	1.57365200
O	1.03127000	1.71907600	1.61663200
C	1.86980200	2.85973400	1.72094800
H	1.73293400	3.51916900	0.84526200
H	1.62013500	3.43652400	2.62965700
C	3.27743100	2.32907500	1.78512500
C	3.81911800	1.91779900	3.01009800
C	4.00833400	2.11421900	0.60984900
C	5.07268000	1.30485300	3.06121600
H	3.24491400	2.06504200	3.92848700
C	5.26285600	1.50189000	0.65692800
H	3.58329700	2.41560300	-0.35108600
C	5.79628700	1.09495800	1.88329200
H	5.48369700	0.98424100	4.02124600

H	5.82296200	1.33586500	-0.26618600
H	6.77667800	0.61395200	1.92125800

(S_{NN})-**TS1**

C	-0.19650500	-4.27113900	-1.09883300
C	-0.39372900	-5.16452400	0.02511100
C	0.05599100	-4.49530500	1.16735300
C	0.50780400	-3.16103200	0.77170000
C	0.46819900	-3.09507700	-0.67371400
C	1.16924700	-2.15871600	1.67363200
C	1.24155800	-2.20137300	-1.61883400
H	-0.45413400	-4.49817200	-2.13319500
H	-0.00312200	-4.84508100	2.19658200
O	0.02910700	-0.20432300	-3.04482100
O	2.78326900	-3.95244400	2.94616100
C	1.48331500	-0.74806400	-1.27057500
C	0.82416500	0.26296900	-2.04519500
C	1.00158300	1.60242000	-1.77100300
C	1.85333200	2.01274300	-0.71194100
C	2.02771700	3.38538200	-0.38659800
C	2.86342400	3.76871000	0.64093900
C	3.56266800	2.79231000	1.39001900
C	3.41594500	1.45267600	1.09473700
C	2.56300800	1.02462500	0.04090200
C	2.38217700	-0.36155500	-0.28692900
C	-0.69209900	0.71191800	-3.83889600
C	2.65149500	-2.19829800	1.38857900
C	3.21340300	-1.38190100	0.42357100
C	4.60639200	-1.50186700	0.08954100
C	5.22311800	-0.70503300	-0.91309000
C	6.56416100	-0.84477700	-1.20707900
C	7.35170100	-1.79168900	-0.51031600
C	6.78038600	-2.58671500	0.46113700
C	5.40110500	-2.46991800	0.78392200
C	4.79881000	-3.30125800	1.76818100
C	3.45457300	-3.18098600	2.05118500
C	3.48599000	-4.95401100	3.64656400
H	0.49035700	2.36908100	-2.35168900
H	1.48235300	4.13487500	-0.96592600
H	2.98504200	4.82753000	0.88153100
H	4.21867300	3.10164200	2.20679700
H	3.95347600	0.70351500	1.67686400
H	-1.39835700	1.30278500	-3.23084100
H	-1.25820600	0.11584400	-4.56577600

H	-0.01651100	1.39917000	-4.37748300
H	4.61914800	0.02557600	-1.45154200
H	7.02023700	-0.22245100	-1.98031900
H	8.41349800	-1.89428500	-0.74674800
H	7.38331400	-3.32436300	0.99712800
H	5.42191800	-4.03707000	2.27536100
H	2.75764000	-5.44844400	4.30234500
H	4.29578400	-4.52576500	4.26311000
H	3.91968800	-5.70073200	2.95814400
H	0.96313100	-2.41510400	2.71931600
H	0.76850500	-1.15810900	1.47420700
H	2.22890800	-2.68774600	-1.71605500
H	0.76615400	-2.25665100	-2.60481600
H	-0.88675500	-6.13241700	-0.01039000
N	-2.99515600	-3.45640500	1.70877500
Rh	-1.56160000	-3.24165300	0.36435300
C	-3.77276600	-2.36852600	2.10868900
C	-3.40412600	-1.17775900	1.29450300
C	-2.37278800	-1.36392800	0.35329300
C	-4.05202300	0.04645400	1.45430400
C	-2.02259300	-0.27801700	-0.45179500
C	-3.67234100	1.13004600	0.65520400
H	-4.84302900	0.13107900	2.20364800
C	-2.66287200	0.96106000	-0.29665600
H	-1.26030200	-0.39568400	-1.21341800
H	-4.16260600	2.09954300	0.77211100
H	-2.36062400	1.80475400	-0.92401600
O	-4.60625500	-2.40497100	2.99467100
N	-3.13844600	-4.61858800	2.41924900
C	-2.98807000	-4.77804500	3.78762900
C	-3.37253000	-5.85391100	1.84419400
C	-3.12494500	-6.10553900	4.12059900
C	-3.36974200	-6.82060200	2.89850000
H	-3.07624600	-6.51862400	5.12554400
C	-3.58546500	-8.15368400	2.52273500
C	-3.77351300	-8.42506000	1.16717600
C	-3.73797100	-7.37488200	0.22766500
H	-3.60465400	-8.95492100	3.26592600
H	-3.94604100	-9.44636900	0.82169200
H	-3.88116200	-7.59142100	-0.83658200
H	-2.82590700	-3.90543800	4.41371300
N	-3.53286900	-6.09549600	0.54942000

(S_{NN})-**IM1**

C	0.23151400	-4.32279500	-1.53264500
C	-0.37781500	-5.02903000	-0.47444100
C	0.00020600	-4.38001000	0.77038000
C	0.79250200	-3.26403700	0.48765200
C	0.89247600	-3.16449000	-0.97170000
C	1.36327100	-2.28902800	1.47856100
C	1.73950000	-2.19944900	-1.75628400
H	0.17459300	-4.55318600	-2.59415000
H	-0.34100900	-4.66573900	1.76266200
O	0.03943600	-0.49103500	-3.04830400
O	3.15837600	-3.94993300	2.70039600
C	1.62530700	-0.75194200	-1.33087200
C	0.71859600	0.10247000	-2.04100000
C	0.59317100	1.43432300	-1.69847000
C	1.32309100	1.96896600	-0.60340500
C	1.15088800	3.31580500	-0.18300700
C	1.82636000	3.80746800	0.91481100
C	2.71045500	2.97291900	1.63974600
C	2.90444800	1.66361400	1.24976900
C	2.22233800	1.12793600	0.12500100
C	2.37664900	-0.23727400	-0.28656400
C	-0.86657900	0.27617400	-3.81560300
C	2.84835500	-2.10823900	1.27794600
C	3.33736100	-1.12662800	0.43382100
C	4.74923200	-0.98360100	0.21821000
C	5.29274100	-0.00206800	-0.65421700
C	6.65610500	0.10890400	-0.83651800
C	7.53925600	-0.76255500	-0.15563400
C	7.04168900	-1.73240300	0.68957300
C	5.64252700	-1.87341100	0.89762900
C	5.11701000	-2.88228300	1.75134900
C	3.75371700	-3.00532700	1.92758900
C	3.96672500	-4.87429300	3.39106100
H	-0.08864900	2.09068700	-2.23733000
H	0.45990000	3.95455900	-0.73912300
H	1.67695600	4.84316700	1.22932700
H	3.23775700	3.36684200	2.51152900
H	3.58048500	1.01867400	1.81221400
H	-1.63785800	0.73967900	-3.18124700
H	-1.35384600	-0.42170200	-4.50642200
H	-0.33718700	1.06236400	-4.38279000
H	4.61356400	0.66981800	-1.17991800
H	7.05604800	0.87116000	-1.50902000
H	8.61760500	-0.66742600	-0.30414600

H	7.72045600	-2.41150800	1.21222900
H	5.81557300	-3.55385800	2.24945100
H	3.28654300	-5.53255600	3.94710900
H	4.64411700	-4.36967800	4.10269900
H	4.57239400	-5.48313200	2.69638700
H	1.15191900	-2.64916300	2.49131700
H	0.87045100	-1.31680200	1.36105500
H	2.78649500	-2.52758500	-1.64627100
H	1.47143300	-2.28976200	-2.81561100
H	-0.99169200	-5.92349800	-0.56271500
N	-2.01908800	-1.28138600	-0.00612400
Rh	-1.19243800	-2.98180400	-0.61068100
C	-2.88592400	-0.60168300	-0.85851500
C	-3.12577000	-1.41059200	-2.08922400
C	-2.37104600	-2.59065100	-2.21724200
C	-4.02483700	-0.99604100	-3.07099400
C	-2.53043700	-3.35033900	-3.38172600
C	-4.17789400	-1.76761600	-4.22745500
H	-4.58421200	-0.06971300	-2.91745100
C	-3.42859300	-2.93918800	-4.37909400
H	-1.96629900	-4.27494800	-3.52665000
H	-4.87766800	-1.45899400	-5.00776900
H	-3.54519800	-3.54468200	-5.28245900
O	-3.34619000	0.50795300	-0.64907400
N	-1.63550400	-0.63465100	1.14185800
C	-1.13468200	0.65573000	1.24591000
C	-1.56007900	-1.24750000	2.38048300
C	-0.73529200	0.89901600	2.53801900
C	-0.98921600	-0.29847600	3.28841700
H	-0.30052200	1.82817900	2.89786100
C	-0.78871400	-0.73766900	4.60332900
C	-1.15231400	-2.04875500	4.91567400
C	-1.70133300	-2.88149200	3.92098700
H	-0.35534200	-0.07762200	5.35896400
H	-1.01482700	-2.44008300	5.92572200
H	-1.98760100	-3.90978400	4.16843300
H	-1.12672400	1.29910300	0.37544300
N	-1.90364600	-2.49786400	2.65769500

(*S*$_{NN}$)-**IM1'**

C	-0.06157200	-4.78482700	1.68279900
C	-0.14376600	-4.82738200	3.07470600
C	0.52880700	-3.64561800	3.59530700
C	1.07968600	-2.91126500	2.52431500

C	0.61817300	-3.54672700	1.30190900
C	2.01267300	-1.73683400	2.63216800
C	0.94573300	-3.17696800	-0.12097100
H	-0.51157400	-5.47601500	0.97378700
H	0.64767400	-3.39614400	4.64877800
O	-1.17792500	-1.94027700	-1.31157800
O	4.21267400	-3.03217900	3.65857800
C	0.97202200	-1.69009300	-0.40553600
C	-0.18238100	-1.08546800	-1.00540000
C	-0.22398400	0.27345700	-1.24177300
C	0.86627200	1.10219500	-0.87435800
C	0.81871800	2.51324600	-1.04245500
C	1.88213800	3.30869500	-0.67087500
C	3.04717200	2.72673100	-0.11468200
C	3.12369500	1.36000800	0.06046300
C	2.04295300	0.51393900	-0.30933800
C	2.07303900	-0.90489200	-0.10509600
C	-2.26629000	-1.50573400	-2.10040700
C	3.25516700	-1.95687700	1.80272900
C	3.28725300	-1.54732600	0.48158700
C	4.45210400	-1.77100000	-0.32407500
C	4.52265800	-1.37382800	-1.68677200
C	5.66166900	-1.59944500	-2.43223700
C	6.78123300	-2.23773600	-1.84670900
C	6.73909900	-2.64368900	-0.52908200
C	5.58048900	-2.42704100	0.26567000
C	5.51678100	-2.85700900	1.62024000
C	4.37724300	-2.63825800	2.36806300
C	5.26616300	-3.71261600	4.30106500
H	-1.10923200	0.73517200	-1.67546300
H	-0.08523100	2.95797700	-1.46614200
H	1.82731200	4.39213000	-0.80278400
H	3.88538400	3.36286800	0.17894300
H	4.01930900	0.91285300	0.49433300
H	-2.83805900	-0.70942600	-1.60119900
H	-2.91627400	-2.37948300	-2.22083200
H	-1.91663800	-1.14548300	-3.08445200
H	3.65952900	-0.88107200	-2.13596700
H	5.70089700	-1.28500700	-3.47761900
H	7.68001100	-2.41120600	-2.44345400
H	7.60040500	-3.14198600	-0.07636600
H	6.38279200	-3.36593900	2.04214200
H	4.92263000	-3.93473700	5.31988300
H	6.17846800	-3.09233900	4.35454900

H	5.51109000	-4.65931300	3.78752700
H	2.27566200	-1.59162400	3.68719500
H	1.51895600	-0.82906600	2.27358000
H	1.93099400	-3.61680600	-0.35264500
H	0.19616000	-3.65679100	-0.76217400
H	-0.65765000	-5.58050300	3.66958700
N	-2.94656000	-3.07724900	1.50521200
Rh	-1.13917000	-2.93178700	2.29574500
C	-3.52671100	-1.95030300	0.91422300
C	-2.74630600	-0.73488800	1.28064200
C	-1.56872000	-0.94930300	2.02162300
C	-3.17358600	0.54272700	0.92103500
C	-0.85082200	0.17553400	2.44140100
C	-2.41882900	1.65311000	1.31213800
H	-4.09576800	0.64783200	0.34350000
C	-1.26817200	1.46552400	2.08222100
H	0.04224800	0.07325100	3.05921800
H	-2.73131300	2.66095700	1.02875100
H	-0.68081500	2.32962600	2.40341600
O	-4.52365700	-1.97618600	0.21869900
N	-3.52981700	-4.28216300	1.18300500
C	-4.53710000	-4.87891600	1.92028700
C	-3.25188900	-5.06882000	0.08269200
C	-4.92233800	-6.05808600	1.32722700
C	-4.11679500	-6.20821200	0.14800100
H	-5.69555700	-6.73306900	1.68674100
C	-3.98545400	-7.15243800	-0.87982000
C	-3.03156100	-6.91104100	-1.86859800
C	-2.23278400	-5.75227000	-1.80518300
H	-4.60999400	-8.04910000	-0.90651100
H	-2.89194300	-7.61338600	-2.69282700
H	-1.47772800	-5.56713700	-2.57726700
H	-4.89631700	-4.38289100	2.81875800
N	-2.32744000	-4.83377000	-0.84064600

(S_{CC},S_{NN})-**TS2**

C	0.08987100	2.03083500	-1.74040300
C	-0.75078900	1.17626200	-2.50090800
C	-0.20377200	-0.13652400	-2.42661100
C	1.04105300	-0.07504000	-1.69916400
C	1.22817800	1.25974600	-1.25731700
C	1.94812900	-1.25158400	-1.46862300
C	2.39946600	1.87416600	-0.52506400
H	-0.04091700	3.09170900	-1.55336900

H	-0.61362300	-1.02907200	-2.88951000
O	2.01883000	2.65834300	2.08043300
O	2.83672100	-1.66366700	-4.03608900
C	2.94615600	1.05888300	0.62952400
C	2.64496100	1.47260500	1.97299100
C	3.02584100	0.69783800	3.05121000
C	3.74098500	-0.51087000	2.85424000
C	4.08064200	-1.36360200	3.93974500
C	4.77250500	-2.53886100	3.73103900
C	5.16511800	-2.91107600	2.42281300
C	4.85530100	-2.09970300	1.34962100
C	4.13352600	-0.88998300	1.53141300
C	3.74654200	-0.05501800	0.43086300
C	1.72348400	3.19268000	3.35728500
C	3.36466200	-0.93457000	-1.86591900
C	4.23031500	-0.37381700	-0.94716400
C	5.57983600	-0.05285500	-1.31824600
C	6.50328500	0.52919000	-0.40855200
C	7.79597200	0.81793100	-0.79680800
C	8.22151300	0.54133900	-2.11771900
C	7.34444600	-0.01260200	-3.02718100
C	6.00673900	-0.32097500	-2.65891600
C	5.08764400	-0.87341200	-3.59440400
C	3.79401700	-1.16058400	-3.21053400
C	3.15590700	-1.88573400	-5.38800600
H	2.75845300	0.99062300	4.06534800
H	3.77587000	-1.07195500	4.94831900
H	5.02081100	-3.18498500	4.57683200
H	5.71365300	-3.84241600	2.26358300
H	5.15396400	-2.38763500	0.34035500
H	1.01696900	2.54861000	3.90288400
H	1.25400100	4.16679800	3.17146500
H	2.64650200	3.33670700	3.94751200
H	6.17447900	0.74373100	0.60883000
H	8.49326600	1.26260500	-0.08307000
H	9.24681900	0.77173100	-2.41750700
H	7.66830400	-0.22123500	-4.05044000
H	5.43191900	-1.05280400	-4.61263000
H	2.24267200	-2.26100700	-5.86796500
H	3.95905800	-2.63591100	-5.50158700
H	3.46997800	-0.95349200	-5.89060300
H	1.57190700	-2.10500300	-2.04456500
H	1.93319200	-1.53256600	-0.40982600
H	3.19976000	2.04282200	-1.26597700

H	2.07173000	2.84967500	-0.14864000
H	-1.67892800	1.45114400	-2.99589900
N	-0.71105700	1.39197000	1.53192100
C	-0.44620400	0.59117400	2.60471200
C	-0.12224400	-0.79466100	2.16870000
C	-0.59083400	-1.21574600	0.91000100
C	0.62125900	-1.65792900	2.97700100
C	-0.27704800	-2.51249700	0.46356000
C	0.94950400	-2.93189300	2.51680300
H	0.95349000	-1.29714100	3.95068600
C	0.50085700	-3.35392100	1.25609700
H	-0.65261400	-2.85473700	-0.50177400
H	1.56486300	-3.59254400	3.13050800
H	0.75830800	-4.35194900	0.89239600
O	-0.50631900	0.91723600	3.79301900
N	-1.35557400	2.57550400	1.82391300
C	-2.49717900	2.66406700	2.60950700
C	-1.06538600	3.83096500	1.32295000
C	-2.95157800	3.95780200	2.64886100
C	-2.06132600	4.73074800	1.83395500
H	-3.82376200	4.31223300	3.19258800
C	-1.96228500	6.07317400	1.44850500
C	-0.91650300	6.43454700	0.59872000
C	-0.01225800	5.45195000	0.15399600
H	-2.68384200	6.81395500	1.80279900
H	-0.79255300	7.46827300	0.26969600
H	0.80465000	5.72952800	-0.52195800
H	-2.88313100	1.77253700	3.09113500
N	-0.07679700	4.16399800	0.50040400
Rh	-0.72781900	0.50594000	-0.36700100
C	-2.46917600	-0.79756500	0.47695900
C	-2.69869900	0.41667300	0.08192300
C	-3.78898100	1.35809700	-0.04755400
C	-3.59340100	2.61957700	-0.63726300
C	-5.05832300	1.04288700	0.48254800
C	-4.63568600	3.54305800	-0.69646400
H	-2.60299800	2.87890800	-1.00969100
C	-6.09768200	1.96722400	0.41678900
H	-5.21069600	0.07157900	0.95755700
C	-5.89018400	3.22115400	-0.17124600
H	-4.46072600	4.52713800	-1.13711600
H	-7.07513400	1.71434700	0.83496700
H	-6.70423700	3.94899300	-0.20967700
C	-3.69765900	-2.11409000	4.38324300

C	-3.29397700	-1.69128600	3.13509200
C	-3.58067900	-2.46640600	1.97700200
C	-4.30183300	-3.69590400	2.13518500
C	-4.71134200	-4.09772100	3.43674300
C	-4.41571700	-3.32633800	4.53942300
H	-3.45352700	-1.50892400	5.25924500
H	-2.73537800	-0.76310600	3.03056700
C	-3.17700700	-2.05627100	0.66789900
C	-4.58189500	-4.47577300	0.98412000
H	-5.26461200	-5.03430900	3.54535400
H	-4.73076300	-3.64641100	5.53524500
C	-4.17176000	-4.08085200	-0.27204800
C	-3.45866600	-2.86642700	-0.43311200
H	-5.13166100	-5.41271900	1.10386600
H	-4.39456500	-4.70812300	-1.13445600
O	-2.97680900	-2.43698700	-1.62761500
C	-3.31139400	-3.11977500	-2.82758100
H	-2.92872200	-4.15509600	-2.80505500
H	-4.41058000	-3.16461600	-2.93447700
C	-2.69195300	-2.35397500	-3.96485400
C	-1.61570800	-2.88134400	-4.68697500
C	-3.15149400	-1.06310100	-4.26629200
C	-1.00699500	-2.13409200	-5.70034900
H	-1.24362300	-3.88105200	-4.44798700
C	-2.54326600	-0.31541800	-5.27431300
H	-3.97822500	-0.63926300	-3.69091700
C	-1.46720500	-0.84887300	-5.99263200
H	-0.16870800	-2.55710300	-6.25844400
H	-2.90520200	0.69045400	-5.49999500
H	-0.98819400	-0.26007300	-6.77820300

(S_{CC},S_{NN})-**IM3**

C	0.18980000	1.55276400	-1.91354700
C	-0.59891600	0.59060500	-2.58366100
C	-0.00834200	-0.69806400	-2.36019600
C	1.17544500	-0.52105000	-1.58360400
C	1.27977600	0.87666300	-1.25012200
C	2.15756200	-1.60978200	-1.25247300
C	2.39292900	1.61077100	-0.53792200
H	0.02160600	2.62281900	-1.85219300
H	-0.37812800	-1.64585400	-2.74111700
O	1.91311800	2.60484500	1.98117100
O	3.17214600	-2.13511000	-3.75830400
C	2.94082100	0.92606300	0.69530000

C	2.58203100	1.43506500	1.98939800
C	2.94736700	0.75970500	3.13700700
C	3.70493100	-0.43678600	3.05872300
C	4.02457900	-1.19649300	4.21648000
C	4.77196800	-2.35223000	4.12384000
C	5.24193100	-2.79810800	2.86513600
C	4.95022600	-2.08006800	1.72286400
C	4.17179600	-0.89370500	1.78495800
C	3.79632700	-0.16087200	0.61144100
C	1.65592600	3.28090800	3.19786300
C	3.56237600	-1.22047600	-1.62953700
C	4.35445100	-0.54642200	-0.72060100
C	5.69533000	-0.16550700	-1.06406700
C	6.54284900	0.53636000	-0.16481000
C	7.82898700	0.88292800	-0.52625900
C	8.32421800	0.54545300	-1.80821900
C	7.52200600	-0.12704300	-2.70667300
C	6.19250500	-0.49672500	-2.36580700
C	5.34908600	-1.17148300	-3.29204400
C	4.06018400	-1.51328500	-2.93702100
C	3.57014400	-2.45385800	-5.07040000
H	2.62866600	1.11756600	4.11476600
H	3.65887700	-0.84892400	5.18613400
H	5.00303100	-2.92751500	5.02390400
H	5.83383500	-3.71401800	2.79869600
H	5.30530500	-2.42599300	0.75059700
H	0.96399000	2.70549700	3.83029400
H	1.18131200	4.23027800	2.91794000
H	2.59721600	3.48504200	3.73953700
H	6.16076000	0.79687700	0.82279400
H	8.46752300	1.42007800	0.17879600
H	9.34400300	0.82244900	-2.08663800
H	7.89951900	-0.38400400	-3.70008100
H	5.74551200	-1.39899500	-4.28124700
H	2.70830600	-2.93272100	-5.55378900
H	4.42430300	-3.15447800	-5.07915600
H	3.84904300	-1.55029000	-5.64156500
H	1.85940900	-2.52211400	-1.78162300
H	2.13195200	-1.82339100	-0.17873600
H	3.20567500	1.75733400	-1.26986100
H	2.00002500	2.59515700	-0.25920000
H	-1.51153600	0.77297300	-3.14642300
N	-0.78355000	1.21266600	1.38120600
C	-0.44841400	0.60567000	2.56576300

C	-0.01997700	-0.79420800	2.33312200
C	-0.16164900	-1.29233200	1.03658900
C	0.40562100	-1.61589600	3.38224000
C	0.07526200	-2.64995300	0.80014100
C	0.68470400	-2.95767500	3.13511900
H	0.49620500	-1.17882400	4.37862800
C	0.50560800	-3.47420000	1.84520300
H	-0.07745000	-3.07729000	-0.19178200
H	1.03016400	-3.60670500	3.94254300
H	0.69948900	-4.53217900	1.64922000
O	-0.55020000	1.11584800	3.68110500
N	-1.45434300	2.40619800	1.50495800
C	-2.62203900	2.56083400	2.23832300
C	-1.12153300	3.62575500	0.94298700
C	-3.06029100	3.85918600	2.17522600
C	-2.12523400	4.56720600	1.35111900
H	-3.94821200	4.25983700	2.65785100
C	-2.00096800	5.87643300	0.87154300
C	-0.92476800	6.16608300	0.03228700
C	-0.01552500	5.14671900	-0.30804200
H	-2.72918100	6.64530600	1.14240400
H	-0.78040100	7.17149800	-0.36835800
H	0.82565900	5.36719100	-0.97513100
H	-3.02904500	1.70859500	2.77259100
N	-0.10423600	3.88884100	0.13098400
Rh	-0.67067300	0.10516500	-0.37371100
C	-2.60335100	-0.92111700	-0.15589200
C	-2.79604900	0.30324500	-0.37491300
C	-3.66487500	1.43629400	-0.56372500
C	-3.23714000	2.62124000	-1.18161500
C	-5.00479600	1.33204200	-0.13624100
C	-4.12554200	3.67811400	-1.37127300
H	-2.20126100	2.71135500	-1.49735800
C	-5.89140800	2.38673500	-0.33768400
H	-5.33492700	0.41511400	0.35626300
C	-5.45426000	3.56540000	-0.95480500
H	-3.77023900	4.60063600	-1.83500500
H	-6.92762600	2.29509300	-0.00339500
H	-6.14792600	4.39660100	-1.10118600
C	-3.53161200	-2.03183100	3.86858700
C	-3.24445900	-1.66658700	2.57052500
C	-3.23061500	-2.63125600	1.53052000
C	-3.52109100	-3.99610200	1.84908400
C	-3.82814500	-4.33842600	3.19394400

C	-3.83115700	-3.37910600	4.18449300
H	-3.51028500	-1.27770600	4.65830300
H	-2.99953300	-0.63398400	2.32765300
C	-2.90278100	-2.28176500	0.17619100
C	-3.47106700	-4.97157600	0.81537500
H	-4.05059900	-5.38200300	3.43118200
H	-4.05663900	-3.65698300	5.21664500
C	-3.12534200	-4.62473000	-0.47149600
C	-2.82535700	-3.27809900	-0.79242600
H	-3.69061600	-6.01321800	1.06264300
H	-3.05205100	-5.37830300	-1.25818500
O	-2.38313300	-2.95509900	-2.04336900
C	-3.30434300	-3.03662100	-3.14330900
H	-2.87651700	-3.72196900	-3.89217700
H	-4.25731400	-3.46117100	-2.79006300
C	-3.51502700	-1.66926800	-3.74060200
C	-2.79698000	-1.26739300	-4.87393500
C	-4.38506500	-0.75545300	-3.12853800
C	-2.94422200	0.02409200	-5.38863400
H	-2.11159500	-1.97098500	-5.35441200
C	-4.53317100	0.53553000	-3.63594100
H	-4.94051300	-1.05280500	-2.23625500
C	-3.81099300	0.92779400	-4.76791900
H	-2.37710500	0.32636700	-6.27220300
H	-5.20357200	1.23983800	-3.14058600
H	-3.92398500	1.94005300	-5.16293500

(S_{CC},S_{NN})-**IM4**

C	0.36859400	1.57641900	-1.78008300
C	-0.38034200	0.56839900	-2.48011400
C	0.29816100	-0.65452200	-2.29157100
C	1.53033200	-0.39001000	-1.56470800
C	1.59288500	0.98993600	-1.26592400
C	2.54051300	-1.44804100	-1.22597400
C	2.67278400	1.77348000	-0.55464000
H	0.12341700	2.63069000	-1.69348700
H	-0.01113400	-1.62784600	-2.66610800
O	2.02138600	2.58212000	2.00132400
O	3.49549200	-2.02176500	-3.73325300
C	3.26560900	1.09675400	0.66697900
C	2.85290200	1.52522000	1.97583400
C	3.32480900	0.88799500	3.10856500
C	4.22754300	-0.20268700	3.00373500
C	4.67432700	-0.91610900	4.14967800

C	5.53457800	-1.98779200	4.02761900
C	5.99080100	-2.39287400	2.75020500
C	5.57848900	-1.71582800	1.61975900
C	4.68922000	-0.61149900	1.71254400
C	4.20694100	0.08597600	0.55506300
C	1.50651200	3.03766600	3.23670700
C	3.92509400	-1.02546800	-1.64822500
C	4.72419400	-0.28825700	-0.79521000
C	6.02978400	0.14422800	-1.20579300
C	6.87812700	0.91365900	-0.36453900
C	8.12945100	1.31031800	-0.79075700
C	8.58723300	0.95640400	-2.08217500
C	7.78273900	0.21783700	-2.92506800
C	6.48830200	-0.20393800	-2.51680600
C	5.64089800	-0.94767600	-3.38446800
C	4.38456700	-1.33625000	-2.96676800
C	3.85387500	-2.35823200	-5.05296200
H	2.99521800	1.19734000	4.09927400
H	4.31574400	-0.60332600	5.13373400
H	5.86437300	-2.52818000	4.91827800
H	6.67005700	-3.24372800	2.66003200
H	5.92711400	-2.03020800	0.63502500
H	0.89909300	2.26101000	3.72929600
H	0.86784200	3.89703200	3.00079500
H	2.31835800	3.36167000	3.91183600
H	6.52522400	1.18694100	0.63057400
H	8.76916800	1.89987700	-0.13008000
H	9.57966700	1.27309100	-2.41208800
H	8.13114200	-0.05181700	-3.92566100
H	6.00696700	-1.18893700	-4.38201400
H	2.99619800	-2.88925000	-5.48646600
H	4.73876600	-3.01898100	-5.07955400
H	4.06610300	-1.45882800	-5.65808500
H	2.25652900	-2.38130600	-1.72509100
H	2.53351300	-1.63746400	-0.14573000
H	3.48011100	1.98033600	-1.27728200
H	2.23292100	2.73108300	-0.25192800
H	-1.32081200	0.70407800	-3.00735800
N	-0.58745200	1.14968500	1.47398400
C	-0.48436300	0.21631000	2.44204600
C	-0.07685800	-1.09904800	1.80081300
C	-0.97052000	-1.82004100	0.94045400
C	1.14498000	-1.69092300	2.18863300
C	-0.55306300	-3.09703800	0.47183500

C	1.51907500	-2.93498600	1.71359800
H	1.78673500	-1.14552800	2.87758000
C	0.66242500	-3.64065500	0.84290600
H	-1.23719400	-3.64962500	-0.17521500
H	2.47516800	-3.36602200	2.01893500
H	0.95514100	-4.62552000	0.47102300
O	-0.69529300	0.32467300	3.64819800
N	-1.33585700	2.27597800	1.72167600
C	-2.51676300	2.29844500	2.45629700
C	-1.16675800	3.50538800	1.11234800
C	-3.10256500	3.53624600	2.36751900
C	-2.27017500	4.33025700	1.51251600
H	-4.02497400	3.84014300	2.85633600
C	-2.30471800	5.63300600	1.00122300
C	-1.28041500	6.02408000	0.13838400
C	-0.26108200	5.11082900	-0.19042600
H	-3.11399500	6.31891800	1.26432600
H	-1.26091900	7.02845500	-0.28941000
H	0.53958900	5.41214200	-0.87544500
H	-2.81921900	1.41336100	3.00478600
N	-0.19391100	3.86308700	0.28131900
Rh	-0.34923400	0.15069500	-0.32164100
C	-2.35837300	-1.33301000	0.61886700
C	-2.33706000	-0.13878500	-0.01175300
C	-3.42403200	0.77474500	-0.36653800
C	-3.14961600	1.98835000	-1.02554500
C	-4.76842200	0.52010200	-0.01024000
C	-4.16105300	2.89511500	-1.33917700
H	-2.11974200	2.23291900	-1.26891900
C	-5.78168800	1.41959300	-0.33402300
H	-5.01735800	-0.38617600	0.53803200
C	-5.48714600	2.61200700	-1.00638700
H	-3.90509000	3.83415800	-1.83538100
H	-6.81194400	1.19352200	-0.04721800
H	-6.28324600	3.31919600	-1.25116400
C	-3.73222300	-2.50602000	4.57992800
C	-3.29526600	-2.12735300	3.32819800
C	-3.91983800	-2.63742100	2.15341100
C	-5.02957000	-3.53675100	2.30547500
C	-5.45111600	-3.90698700	3.61241800
C	-4.81597100	-3.40737500	4.72833800
H	-3.23476900	-2.10183700	5.46460600
H	-2.46498000	-1.42695600	3.24644500
C	-3.50139900	-2.25263000	0.84167500

C	-5.68498300	-4.01993900	1.14292200
H	-6.29462200	-4.59515400	3.71427800
H	-5.14821300	-3.69885400	5.72751500
C	-5.26964200	-3.64885600	-0.11788200
C	-4.16845400	-2.76925200	-0.26897400
H	-6.53116900	-4.70193200	1.25955500
H	-5.77425400	-4.04387700	-1.00061900
O	-3.69459000	-2.40670100	-1.49227700
C	-4.59515300	-2.24583200	-2.58333200
H	-4.81129800	-3.22628300	-3.04643700
H	-5.54505000	-1.81711800	-2.22411200
C	-3.94472100	-1.32403800	-3.58106100
C	-2.94458300	-1.80496000	-4.43864900
C	-4.28041400	0.03442800	-3.62072500
C	-2.29843400	-0.94553200	-5.32892800
H	-2.67096900	-2.86302500	-4.40374300
C	-3.63982500	0.89600500	-4.51513300
H	-5.03622300	0.42627500	-2.93765700
C	-2.64927800	0.40840800	-5.37156800
H	-1.52002800	-1.33048500	-5.99201100
H	-3.90963400	1.95415600	-4.52941400
H	-2.14522200	1.08269700	-6.06808200

(R_{CC},S_{NN})-**TS2**

C	0.89376700	-0.87729700	-2.89208100
C	0.51683100	-2.21300900	-2.59113600
C	1.21156400	-2.59395800	-1.40563300
C	2.10096800	-1.51892000	-1.02722200
C	1.90465100	-0.44824800	-1.93475800
C	3.04441800	-1.56476800	0.14353600
C	2.63237700	0.87376500	-2.03391400
H	0.53706600	-0.25875100	-3.70903900
H	1.15760100	-3.55910900	-0.90512500
O	1.24527300	3.16187300	-1.40515200
O	4.84856700	-3.43434600	-0.75579400
C	2.85983800	1.59674800	-0.72185600
C	2.04799500	2.74124100	-0.41056600
C	2.14418300	3.35989800	0.82083100
C	3.05866400	2.88741700	1.79601800
C	3.12279900	3.45522100	3.09789400
C	4.01138600	2.97709200	4.03876900
C	4.88640300	1.91205200	3.71532800
C	4.85330800	1.34665500	2.45637600
C	3.94049200	1.80863500	1.47099000

C	3.84110100	1.20324300	0.17467500
C	0.44134100	4.31388700	-1.22971400
C	4.44174600	-1.17367000	-0.25884500
C	4.82470400	0.15315300	-0.22890400
C	6.14925700	0.54012100	-0.62634300
C	6.58397600	1.89312000	-0.61995800
C	7.86579300	2.22871500	-1.00629300
C	8.77118300	1.22350300	-1.42133900
C	8.37551400	-0.09780100	-1.44750200
C	7.06219800	-0.47584700	-1.05723000
C	6.63677000	-1.83291200	-1.10062600
C	5.35380200	-2.17214300	-0.72341300
C	5.66979000	-4.48232100	-1.21427700
H	1.49491000	4.19702100	1.07144100
H	2.44537400	4.27767800	3.34255600
H	4.04336500	3.42171900	5.03666200
H	5.58817800	1.53793300	4.46443600
H	5.52401300	0.52273600	2.20721000
H	1.06358200	5.19835700	-1.00270300
H	-0.30419900	4.15694600	-0.43531100
H	-0.07330000	4.46787400	-2.18646600
H	5.88672400	2.66868500	-0.30136200
H	8.18308500	3.27395700	-0.99298000
H	9.78464700	1.49697200	-1.72507800
H	9.07023300	-0.87660800	-1.77337600
H	7.34478700	-2.58563600	-1.44608500
H	6.56403600	-4.60825000	-0.57797300
H	5.99809600	-4.31528100	-2.25568700
H	5.06609600	-5.39842500	-1.17028900
H	3.03945300	-2.57760200	0.56258500
H	2.69560500	-0.88220600	0.92698900
H	3.60497600	0.67719300	-2.51667000
H	2.04548700	1.51771600	-2.69852300
H	-0.21248900	-2.81449800	-3.13055700
N	-0.85236600	1.05590700	-0.65069100
Rh	-0.10326100	-0.87179000	-0.91905100
C	-0.79221500	1.58987000	0.60107700
C	-0.13763100	0.65054000	1.55382300
C	-0.12257800	-0.71688500	1.21860200
C	0.43644100	1.09414500	2.74622100
C	0.48212700	-1.63424600	2.09541900
C	1.05931800	0.18203100	3.59907400
H	0.40511500	2.15977100	2.97365400
C	1.08092600	-1.18166500	3.27057700

H	0.47140500	-2.70091000	1.86485200
H	1.53995500	0.53333400	4.51446800
H	1.56570000	-1.89689900	3.93965100
O	-1.25310500	2.68111600	0.95399000
N	-1.75812000	1.64555400	-1.50463100
C	-3.08402700	1.88656600	-1.17249300
C	-1.56510700	1.97753800	-2.83236600
C	-3.76172800	2.39339600	-2.25216600
C	-2.82176400	2.46085500	-3.33275500
H	-4.81096300	2.67767400	-2.26976500
C	-2.86049300	2.85319100	-4.67618300
C	-1.68899600	2.73919300	-5.42535700
C	-0.52341900	2.23336200	-4.81990900
H	-3.78192200	3.23466700	-5.12382100
H	-1.66508800	3.03253200	-6.47682600
H	0.39590000	2.13256700	-5.40802000
C	-1.86808700	-1.46570500	0.56256100
C	-2.06284800	-1.33775900	-0.70951300
C	-3.17951100	-1.38833500	-1.63673800
C	-3.01113700	-1.06100100	-2.99216000
C	-4.47325200	-1.69548100	-1.16501900
C	-4.10762700	-1.02185500	-3.85304500
H	-2.01661400	-0.79979300	-3.35252400
C	-5.56495400	-1.66109700	-2.02935900
H	-4.60961100	-1.95538700	-0.11344800
C	-5.38661400	-1.31921000	-3.37582100
H	-3.96237000	-0.74000400	-4.89824500
H	-6.56261600	-1.89882400	-1.65165700
H	-6.24596700	-1.28245500	-4.04963600
C	-1.97701800	-5.58755000	1.89071700
C	-1.93212300	-4.27919900	1.45643500
C	-2.54702400	-3.24204800	2.21020400
C	-3.21933700	-3.58337900	3.42913800
C	-3.24862800	-4.94191300	3.84618300
C	-2.63970800	-5.92597900	3.09705800
H	-1.49998600	-6.37266700	1.29899400
H	-1.42620700	-4.01833100	0.52512900
C	-2.51027000	-1.87568700	1.79514200
C	-3.84487800	-2.54993800	4.17638200
H	-3.76565700	-5.19277800	4.77612600
H	-2.66773700	-6.96655400	3.42801200
C	-3.81127600	-1.23565000	3.76406900
C	-3.12483900	-0.88794500	2.57299300
H	-4.37233600	-2.81317800	5.09665300

H	-4.32164400	-0.46890000	4.34507000
O	-3.01587700	0.37218600	2.10761100
C	-3.60843000	1.47309800	2.77636100
H	-3.37633300	1.44702400	3.85476100
H	-3.08427900	2.34217600	2.34733700
C	-5.09933700	1.55506800	2.53859700
C	-5.91439800	2.28057400	3.41733600
C	-5.68184600	0.94348000	1.41977100
C	-7.28518500	2.40259800	3.17795600
H	-5.47227800	2.75609900	4.29782500
C	-7.05300100	1.06199900	1.18200900
H	-5.06136700	0.36668300	0.73308900
C	-7.85931400	1.79284900	2.05841700
H	-7.90866000	2.97203200	3.87163800
H	-7.48941600	0.57926600	0.30406600
H	-8.93204700	1.88521200	1.87282000
H	-3.42266500	1.67408000	-0.16645000
N	-0.44967700	1.85044700	-3.54264000

(R_{CC},S_{NN})-**IM3**

C	0.76549700	-0.99064900	-2.79679300
C	0.30151200	-2.27562200	-2.43209500
C	0.94014300	-2.64760500	-1.20157500
C	1.82905800	-1.59670500	-0.82678900
C	1.69072000	-0.53418900	-1.79052400
C	2.78857000	-1.65360500	0.32908600
C	2.48567400	0.74538300	-1.92565300
H	0.47266200	-0.39605800	-3.65644100
H	0.83852200	-3.59429800	-0.67593900
O	1.22975600	3.13300300	-1.40295900
O	4.51459000	-3.60937400	-0.54961700
C	2.73690800	1.49988600	-0.63837500
C	1.98379100	2.69474700	-0.37680700
C	2.08095300	3.33470900	0.84276900
C	2.94235200	2.83450700	1.85219600
C	2.99906800	3.42231400	3.14486700
C	3.84924700	2.92914800	4.11278100
C	4.69030500	1.82674500	3.82780000
C	4.66068900	1.23698300	2.58001600
C	3.78581400	1.71352200	1.56763300
C	3.68325600	1.08268700	0.28430700
C	0.55792700	4.37513800	-1.31148000
C	4.19568600	-1.32622800	-0.09612700
C	4.62825800	-0.01428600	-0.08795100

C	5.96722700	0.31383800	-0.48885900
C	6.45361200	1.64916300	-0.50396800
C	7.74824500	1.92823400	-0.89200500
C	8.61505600	0.88175000	-1.28737300
C	8.16904900	-0.42362100	-1.29246600
C	6.84114200	-0.74411100	-0.89981200
C	6.36417100	-2.08442500	-0.92100300
C	5.06844900	-2.36741300	-0.54045500
C	5.29743500	-4.69839900	-0.97906300
H	1.47021800	4.20999700	1.05800900
H	2.34765600	4.27329800	3.36014800
H	3.87577200	3.38959000	5.10362000
H	5.36155700	1.44126900	4.59880700
H	5.30349500	0.38269000	2.36077800
H	1.27239900	5.19442700	-1.11294400
H	-0.22118500	4.34782900	-0.53528300
H	0.08440100	4.53065400	-2.28936700
H	5.78581900	2.45590600	-0.20017400
H	8.10600100	2.96039800	-0.89501400
H	9.63909500	1.11086400	-1.59232500
H	8.83406300	-1.23405100	-1.60271900
H	7.04336700	-2.86985800	-1.25097400
H	6.18384700	-4.84225700	-0.33570700
H	5.63559400	-4.56916000	-2.02268200
H	4.65932600	-5.58966900	-0.91571600
H	2.74710000	-2.65641800	0.76993000
H	2.48354600	-0.93912400	1.10098700
H	3.45044900	0.47795200	-2.38969400
H	1.93811100	1.39101200	-2.62186300
H	-0.42779300	-2.87443800	-2.97494700
N	-1.03786400	1.11397300	-0.77581000
Rh	-0.33985400	-0.82910200	-0.79990200
C	-0.99785300	1.81152300	0.40087800
C	-0.34915000	1.01187500	1.46629300
C	-0.07300400	-0.32266800	1.16477600
C	-0.13652000	1.52404800	2.75088300
C	0.36115900	-1.17363700	2.18681100
C	0.34528900	0.68626000	3.75468800
H	-0.36238300	2.57673300	2.93363400
C	0.57763500	-0.66629900	3.47206400
H	0.52398700	-2.23495800	1.99684200
H	0.53232400	1.07719800	4.75709100
H	0.92819100	-1.33692000	4.26089000
O	-1.49605900	2.92447100	0.58654300

N	-1.88274600	1.61845100	-1.73064400
C	-3.23795400	1.84039700	-1.52770900
C	-1.56144400	1.97449700	-3.02766200
C	-3.80972100	2.35447400	-2.66365600
C	-2.76635800	2.45056100	-3.64368900
H	-4.85703300	2.61704300	-2.79030400
C	-2.67870700	2.84576900	-4.98361800
C	-1.44030100	2.73649700	-5.61743900
C	-0.33560600	2.23216300	-4.90534800
H	-3.55576000	3.21907800	-5.51853100
H	-1.31729900	3.03068900	-6.66173800
H	0.63485300	2.13277200	-5.40459900
C	-2.24157000	-1.63155800	0.06637800
C	-2.39230300	-1.35664800	-1.15092000
C	-3.24560000	-1.22821100	-2.30927100
C	-2.76253400	-0.91023200	-3.58759800
C	-4.63457900	-1.40121700	-2.12788300
C	-3.64357200	-0.75500100	-4.65755100
H	-1.69741900	-0.74952100	-3.72956500
C	-5.50860900	-1.25186200	-3.20034600
H	-5.01249200	-1.64155400	-1.13206700
C	-5.01658400	-0.92372300	-4.46963300
H	-3.25140700	-0.48028500	-5.63893800
H	-6.58234100	-1.38361100	-3.04621700
H	-5.70538700	-0.79491700	-5.30779900
C	-1.19293300	-5.43313300	1.77277000
C	-1.57961900	-4.23867300	1.20094100
C	-2.15299100	-3.20649200	1.99119000
C	-2.33315000	-3.43253300	3.39264300
C	-1.93092800	-4.67668100	3.94989000
C	-1.36696400	-5.65707400	3.16142700
H	-0.75327100	-6.21671200	1.15068100
H	-1.45410500	-4.06644300	0.13114600
C	-2.51989400	-1.94247000	1.43260900
C	-2.90061100	-2.39782300	4.18150700
H	-2.07397100	-4.84049500	5.02119700
H	-1.05748100	-6.60762900	3.60163800
C	-3.26509400	-1.18846500	3.63227700
C	-3.06068400	-0.94343300	2.25134600
H	-3.04996200	-2.57177200	5.24994200
H	-3.70967200	-0.41975500	4.26165800
O	-3.33590100	0.23886700	1.65699500
C	-3.67443000	1.37617100	2.44292500
H	-3.00749900	1.44509500	3.31742700

H	-3.42908200	2.23562300	1.80008500
C	-5.12789100	1.39021800	2.85225500
C	-5.53089500	2.09508400	3.99411200
C	-6.09603700	0.73409900	2.08044800
C	-6.87914100	2.15135900	4.35516600
H	-4.78112900	2.60386600	4.60699800
C	-7.44343000	0.78424400	2.44435600
H	-5.78319700	0.17744300	1.19483300
C	-7.83987200	1.49441200	3.58107000
H	-7.17987400	2.70470200	5.24820300
H	-8.18871400	0.26562400	1.83620400
H	-8.89410200	1.53292400	3.86542300
H	-3.67220600	1.58728900	-0.56644700
N	-0.38277800	1.84763900	-3.62685300

(R_{CC},S_{NN})-**IM4**

C	0.89259200	-0.79811000	-2.59916100
C	0.56345700	-2.14693600	-2.22707500
C	1.37123600	-2.47876500	-1.11398600
C	2.26913000	-1.36502500	-0.85519100
C	1.99850300	-0.33387000	-1.78458900
C	3.30071500	-1.36115500	0.23546400
C	2.67481300	1.00725300	-1.96368800
H	0.44447300	-0.21377300	-3.39722000
H	1.38036000	-3.42036300	-0.56891800
O	1.21953700	3.20157200	-1.13451900
O	5.00372600	-3.27239100	-0.74373900
C	3.01665500	1.74923300	-0.68514800
C	2.19883000	2.85996700	-0.27812600
C	2.45345200	3.52795900	0.90552400
C	3.52365900	3.12876100	1.74789000
C	3.76041200	3.75407900	3.00278100
C	4.79229700	3.33911300	3.81906000
C	5.63863300	2.27921000	3.41338300
C	5.43607600	1.65619500	2.19803400
C	4.37837500	2.05703600	1.33785500
C	4.11418500	1.40375300	0.08798700
C	0.33566100	4.25695500	-0.80530300
C	4.65492000	-0.98868500	-0.31360300
C	5.04245200	0.33549600	-0.38861900
C	6.31617000	0.69435400	-0.94515300
C	6.74809700	2.04381800	-1.05361900
C	7.98193900	2.35302300	-1.58909200
C	8.83922900	1.32322200	-2.04428300

C	8.44333100	0.00429600	-1.96220800
C	7.17806300	-0.34676500	-1.41796900
C	6.74929400	-1.70151900	-1.34818000
C	5.51211300	-2.01418100	-0.82349800
C	5.76917200	-4.34755900	-1.23618500
H	1.82000500	4.35438500	1.22417600
H	3.10159600	4.56884800	3.31396100
H	4.95738500	3.82741300	4.78259500
H	6.45324100	1.95411700	4.06468300
H	6.08697900	0.83734400	1.88809900
H	0.88383300	5.20582600	-0.66778200
H	-0.24662700	4.01886400	0.09926700
H	-0.34792200	4.35521500	-1.65712100
H	6.08764500	2.83805500	-0.70399400
H	8.29843300	3.39594000	-1.66293200
H	9.81537600	1.57598000	-2.46533500
H	9.10015700	-0.79328700	-2.31948600
H	7.41619500	-2.47500000	-1.72775300
H	6.72494500	-4.45053600	-0.69204700
H	5.98578100	-4.23185800	-2.31302600
H	5.17039200	-5.25535000	-1.08536200
H	3.33531600	-2.35389300	0.69816000
H	3.01365400	-0.64404600	1.01471800
H	3.60083400	0.84808300	-2.54181100
H	1.99995800	1.62859100	-2.56412000
H	-0.20225400	-2.76961500	-2.68536500
N	-0.87250400	1.06453100	-0.38128200
Rh	0.04666200	-0.77370200	-0.60920300
C	-0.83349900	1.39744300	0.92539900
C	-0.07429800	0.31932100	1.67753300
C	-0.52099500	-1.04202800	1.74430300
C	1.06684600	0.71238800	2.40771300
C	0.24469500	-1.95065800	2.52718100
C	1.79363900	-0.20071800	3.15201800
H	1.36378500	1.75843300	2.37138500
C	1.37837700	-1.54610500	3.20888600
H	-0.10540900	-2.98192700	2.60484100
H	2.68338300	0.12877500	3.69348000
H	1.94381300	-2.26797600	3.80300500
O	-1.28509100	2.39998800	1.47596400
N	-1.78279000	1.69728700	-1.19208000
C	-3.09495900	1.96127800	-0.82646500
C	-1.61144500	2.04286700	-2.52008300
C	-3.78288100	2.50350000	-1.88223600

C	-2.86614000	2.56619900	-2.98286500
H	-4.82533400	2.81172800	-1.86713100
C	-2.92391400	2.98077800	-4.31917900
C	-1.77392000	2.84473600	-5.09747300
C	-0.61013800	2.29481100	-4.52864800
H	-3.84436300	3.39378300	-4.73982400
H	-1.76658500	3.15251700	-6.14498800
H	0.29089600	2.17389000	-5.14060100
C	-1.82711100	-1.51153600	1.14947800
C	-1.84154800	-1.41358300	-0.19488700
C	-2.94693900	-1.55717800	-1.14910700
C	-2.80360500	-1.06546300	-2.46188500
C	-4.20416200	-2.09216600	-0.78695100
C	-3.86864100	-1.06517500	-3.36108700
H	-1.84824300	-0.64078700	-2.76210500
C	-5.26604600	-2.10220000	-1.69070400
H	-4.35368500	-2.49718400	0.21101900
C	-5.10932900	-1.58098100	-2.97939600
H	-3.72772000	-0.64556200	-4.35983200
H	-6.22749200	-2.52020700	-1.38144800
H	-5.94767500	-1.58057500	-3.68023800
C	-2.80807900	-5.70638500	1.82130900
C	-2.49926200	-4.38107000	1.60042100
C	-3.17029500	-3.34497200	2.31146100
C	-4.18502400	-3.71314100	3.25819100
C	-4.47633300	-5.08995000	3.46221600
C	-3.80433200	-6.06894900	2.76268300
H	-2.28324400	-6.48638400	1.26411300
H	-1.74094400	-4.10207900	0.86669100
C	-2.87981000	-1.96467900	2.09629900
C	-4.87522300	-2.68561800	3.95345900
H	-5.24968800	-5.35828600	4.18697300
H	-4.03833000	-7.12315400	2.92778000
C	-4.58734000	-1.35519500	3.74035800
C	-3.57564100	-0.99486300	2.81433200
H	-5.65678100	-2.96407100	4.66483800
H	-5.14644600	-0.58469500	4.26999900
O	-3.21882300	0.28584300	2.54854600
C	-3.99631300	1.38022400	2.98922700
H	-4.11767800	1.36446500	4.08754500
H	-3.37206900	2.25204300	2.73744900
C	-5.34394700	1.47055800	2.30278600
C	-6.32709000	2.33026800	2.81203100
C	-5.61948400	0.74195600	1.13810500

C	-7.55546100	2.46991300	2.16298000
H	-6.12776500	2.89978900	3.72473700
C	-6.84835200	0.87954000	0.48833100
H	-4.87399500	0.06249800	0.72717700
C	-7.81998400	1.74490000	0.99629600
H	-8.31102000	3.14600200	2.57086200
H	-7.03602000	0.30328200	-0.42085800
H	-8.78171100	1.85316400	0.48904100
H	-3.41582900	1.74451400	0.18297100
N	-0.51769700	1.89141900	-3.25869300

(S_{NN})-**IM2**

C	0.66671300	-4.27873800	-1.35569500
C	0.37886600	-5.13228100	-0.26904500
C	0.59398300	-4.37902500	0.95651700
C	0.94277700	-3.06615200	0.62379900
C	0.93398500	-2.95670100	-0.84053900
C	1.25855100	-1.94806900	1.57494900
C	1.47444100	-1.83112900	-1.68453900
H	0.63232800	-4.53783200	-2.41175900
H	0.45414400	-4.75604800	1.96782200
O	-0.53233600	-0.44935000	-2.93705000
O	3.36039200	-3.15219800	2.82311800
C	1.12564900	-0.41994800	-1.26721700
C	0.08711600	0.27046900	-1.97639600
C	-0.21206200	1.58769800	-1.68521600
C	0.46580100	2.26764700	-0.63818900
C	0.12307400	3.59802700	-0.27288100
C	0.75444100	4.22873400	0.77875300
C	1.75926500	3.55409000	1.51298000
C	2.11665500	2.26404900	1.17790000
C	1.48694300	1.58786900	0.09841600
C	1.82201100	0.24058200	-0.26661100
C	-1.56266300	0.15726100	-3.69429500
C	2.67150500	-1.46988900	1.33571800
C	2.94340500	-0.45105000	0.43894000
C	4.29957600	-0.06573700	0.16260000
C	4.62845800	0.95014300	-0.77578400
C	5.94241800	1.29523400	-1.01796400
C	6.99041900	0.63685300	-0.33176000
C	6.70425500	-0.36046900	0.57684800
C	5.36143700	-0.74200700	0.84509200
C	5.05722800	-1.78533600	1.76223300
C	3.74681900	-2.15088700	1.99045500

C	4.34712900	-3.88487000	3.51282900
H	-0.99416800	2.11730000	-2.22676400
H	-0.66121800	4.11039600	-0.83573200
H	0.47641200	5.24991300	1.05016600
H	2.25100800	4.05650600	2.34904600
H	2.88685100	1.74499000	1.74939100
H	-2.38605600	0.49306800	-3.04390600
H	-1.93893600	-0.61484100	-4.37448500
H	-1.17501300	1.01341900	-4.27397600
H	3.82338200	1.45899500	-1.30653600
H	6.17539300	2.07905800	-1.74219000
H	8.02810300	0.91778200	-0.52708800
H	7.51102300	-0.87716700	1.10326900
H	5.88229400	-2.29040100	2.26336600
H	3.81807900	-4.63560500	4.11417800
H	4.94018300	-3.23748900	4.18285700
H	5.03339400	-4.39741300	2.81564100
H	1.13939700	-2.30606200	2.60398400
H	0.56283900	-1.11600100	1.42095600
H	2.57306500	-1.93404100	-1.68230000
H	1.13057600	-1.99098100	-2.71328000
H	0.08568100	-6.17657300	-0.32942500
N	-2.09543000	-1.72900300	0.04787800
Rh	-1.07980900	-3.47613300	-0.35350400
C	-3.03579500	-1.30051800	-0.83294700
C	-3.12405600	-2.21747000	-1.99122900
C	-2.21798800	-3.29178600	-2.01884100
C	-4.06537900	-2.02293500	-3.00502600
C	-2.23951000	-4.14786200	-3.12421000
C	-4.08708700	-2.89176000	-4.09666500
H	-4.76342400	-1.18663700	-2.92134100
C	-3.16543200	-3.94347100	-4.15631500
H	-1.56426100	-5.00345300	-3.18433900
H	-4.81757700	-2.75512200	-4.89735000
H	-3.17472400	-4.62551000	-5.01122100
O	-3.71018100	-0.26472000	-0.70070600
N	-1.92070100	-0.99075200	1.19425200
C	-1.54165800	0.34320600	1.27693300
C	-2.08664300	-1.48246400	2.46608900
C	-1.44757300	0.72060600	2.59422500
C	-1.79066200	-0.42799500	3.38506800
H	-1.16881300	1.70933500	2.94895500
C	-1.92158800	-0.72972100	4.74518000
C	-2.32603100	-2.01879600	5.09863200

C	-2.59638500	-2.97193200	4.10662700
H	-1.72456300	0.02481400	5.51024300
H	-2.45323100	-2.29490400	6.14634300
H	-2.93029800	-3.97618900	4.37114000
H	-1.38916900	0.90649000	0.36524300
N	-2.47531900	-2.71604100	2.79635700
C	-5.47557100	-1.20372900	1.65844800
H	-4.38571600	-1.33258300	1.61042700
C	-5.75673600	-0.59697200	3.03795100
C	-6.06511800	-2.60809400	1.47402900
O	-5.95555700	-0.35713500	0.67852900
H	-5.20502100	-0.18575600	0.06079800
F	-5.77847100	-3.05715900	0.24474700
F	-7.38870600	-2.64316800	1.63273500
F	-5.52951800	-3.49821500	2.34925200
F	-5.07150100	0.54706000	3.16767600
F	-7.05124900	-0.33071600	3.22464800
F	-5.36018900	-1.41841300	4.03136100
C	-3.47697700	-5.51167400	0.94763300
H	-4.44747400	-5.36307200	1.45087800
C	-3.80754300	-6.12437700	-0.42157600
C	-2.68241300	-6.47358600	1.85051800
O	-2.82948300	-4.29700300	0.78650200
H	-2.77858400	-3.72168300	1.65984200
F	-2.32050800	-5.82717300	2.97531000
F	-1.55535300	-6.92855400	1.28262000
F	-3.41855200	-7.52889800	2.20636600
F	-4.54642100	-5.28546900	-1.13930000
F	-2.70347100	-6.42589300	-1.12378400
F	-4.50200400	-7.26144900	-0.25660300

(S_{CC},S_{NN})-**TS3**

C	0.14533400	1.00303200	-2.47120300
C	-0.31242700	-0.30427500	-2.84584900
C	0.45440200	-1.23406200	-2.11342500
C	1.44133900	-0.52076700	-1.32444400
C	1.27035800	0.87481500	-1.59266900
C	2.58832100	-1.15521200	-0.58055600
C	2.16426700	2.03219700	-1.23275900
H	-0.24321700	1.95051500	-2.83664200
H	0.31784200	-2.31227700	-2.07109200
O	1.26881700	3.58557300	0.85277100
O	3.65888100	-2.57382200	-2.63836200
C	2.86079000	2.01129600	0.10832100

C	2.38248800	2.86948100	1.15234400
C	3.03769500	2.94347700	2.36296600
C	4.15588000	2.11233800	2.62665500
C	4.79197400	2.10676900	3.89778000
C	5.84850600	1.25971000	4.15778800
C	6.31936800	0.38014100	3.15284800
C	5.72805200	0.37021200	1.90605400
C	4.63656900	1.23068800	1.60772300
C	3.98913700	1.23513100	0.32694800
C	0.75083800	4.47530200	1.81795500
C	3.89462700	-0.73188200	-1.20477900
C	4.55329800	0.41086500	-0.78673000
C	5.77008300	0.82670200	-1.42885200
C	6.46825600	2.00624400	-1.05312000
C	7.63720800	2.37376500	-1.68828200
C	8.16126900	1.57642600	-2.73308400
C	7.50075800	0.43177500	-3.12836800
C	6.29267400	0.02843500	-2.49732400
C	5.59250800	-1.13763100	-2.91465200
C	4.41464000	-1.49980800	-2.29494700
C	4.08907100	-3.40812300	-3.68905800
H	2.67559900	3.58940000	3.16065500
H	4.41598800	2.77881600	4.67331200
H	6.32277400	1.26203500	5.14220300
H	7.15253500	-0.29303700	3.36690300
H	6.08970800	-0.30906200	1.13322500
H	1.46631900	5.28754600	2.03787900
H	0.49819000	3.95080600	2.75274000
H	-0.16841900	4.89749800	1.39490200
H	6.06710600	2.62173800	-0.24742400
H	8.16044000	3.28297900	-1.38346900
H	9.08891500	1.87191700	-3.22913400
H	7.89802700	-0.18313600	-3.94020300
H	6.00399400	-1.72275100	-3.73659600
H	5.07064600	-3.86412000	-3.46918500
H	4.15906800	-2.85623400	-4.64311700
H	3.33700500	-4.20177100	-3.78634300
H	2.47916900	-2.24128200	-0.62091600
H	2.58961600	-0.85983200	0.47250000
H	2.93684400	2.07671300	-2.02026000
H	1.57105000	2.94904300	-1.30901400
H	-1.15774200	-0.53123600	-3.49174000
N	-1.45328900	-1.51296400	0.52491900
Rh	-0.63791500	0.03830100	-0.63782500

C	-2.80157400	-1.56578800	0.67193400
C	-3.47905000	-0.60638500	-0.22956400
C	-2.72878500	0.47159500	-0.74425800
C	-4.80484000	-0.81442500	-0.62129100
C	-3.32092000	1.28700700	-1.72852900
C	-5.38314800	0.01422700	-1.58109900
H	-5.34500800	-1.65506300	-0.18289900
C	-4.62753600	1.05263000	-2.14656700
H	-2.76388200	2.13155500	-2.12873100
H	-6.41436100	-0.15096900	-1.90038900
H	-5.07001300	1.69770700	-2.91001700
O	-3.41771400	-2.31450900	1.44475000
N	-0.72290200	-2.28855300	1.39548600
C	-0.74577900	-2.19118900	2.78181300
C	0.19197500	-3.26111800	1.03308200
C	0.15745600	-3.06663900	3.32867200
C	0.78372600	-3.75763000	2.23685100
H	0.34920700	-3.20198500	4.39012500
C	1.75921600	-4.75185400	2.09167700
C	2.06205200	-5.18749500	0.80146400
C	1.37962000	-4.64172900	-0.29951200
H	2.25831000	-5.18275300	2.96310200
H	2.80769800	-5.96725600	0.63637900
H	1.59409500	-5.00194300	-1.31044200
C	-1.81770900	1.53832600	0.54328100
C	-0.81472300	0.98796600	1.16199500
C	-0.10576400	1.00611700	2.41489800
C	-0.63387600	1.70999900	3.52109800
C	1.08504600	0.28173000	2.58587700
C	0.03289400	1.69663900	4.74480500
H	-1.57196400	2.25600900	3.41168900
C	1.74736900	0.26825000	3.80968800
H	1.47259700	-0.28504000	1.74509200
C	1.22700900	0.97875100	4.89341400
H	-0.38608900	2.24350500	5.59313900
H	2.67426800	-0.29744200	3.91549600
H	1.74571200	0.96940100	5.85479300
C	-5.16769200	1.87442700	3.16879700
C	-4.10250100	1.76140300	2.30100100
C	-3.67461400	2.87520800	1.52373300
C	-4.37993500	4.11603300	1.66156700
C	-5.47055500	4.19903800	2.57053300
C	-5.85864600	3.10373600	3.31059100
H	-5.48491700	1.00390100	3.74738700

H	-3.58543400	0.80837600	2.19740100
C	-2.56328100	2.79481700	0.62472500
C	-3.96929400	5.22314100	0.87667400
H	-5.99801200	5.15150800	2.66842400
H	-6.70001400	3.17710600	4.00334000
C	-2.91821900	5.12527100	-0.00900300
C	-2.20808100	3.90396500	-0.14125600
H	-4.51107800	6.16747900	0.97043900
H	-2.64984300	5.98326800	-0.62229500
O	-1.17841400	3.74259500	-1.01917900
C	-0.58223500	4.87511500	-1.62844500
H	-0.39394400	5.66425300	-0.88131700
H	0.40258900	4.51841100	-1.96694300
C	-1.36007700	5.42876900	-2.80165800
C	-1.36579300	6.80634600	-3.05516700
C	-2.05271300	4.57625400	-3.67157400
C	-2.04800200	7.32462900	-4.15904000
H	-0.83497500	7.48246100	-2.37845100
C	-2.74128000	5.09216800	-4.77104700
H	-2.05819100	3.50183100	-3.48213300
C	-2.74007400	6.46803500	-5.01888200
H	-2.04536500	8.40159700	-4.34291900
H	-3.28099100	4.41603200	-5.43826800
H	-3.27994000	6.87155100	-5.87855900
H	-1.41959100	-1.48611600	3.25699800
N	0.45047600	-3.68564900	-0.19811000
C	-2.58538900	-5.13540700	0.04513800
H	-1.84563900	-4.36491100	-0.23530600
C	-1.80020300	-6.44959900	0.10464500
C	-3.63489100	-5.11525700	-1.07625400
O	-3.17220800	-4.93918500	1.28004000
H	-3.25429400	-3.96761900	1.44477200
F	-4.58324700	-6.04278800	-0.92264900
F	-3.07636600	-5.29124800	-2.28758500
F	-4.24198300	-3.91242500	-1.09153900
F	-2.58900500	-7.50952100	0.31831400
F	-1.12377800	-6.67073600	-1.03873500
F	-0.90461100	-6.39980900	1.09851900

(S_{CC},S_{NN})-**IM5**

C	-0.15754800	1.13509100	-2.02859400
C	-0.67467200	-0.16508000	-2.30572400
C	0.15238000	-1.10464900	-1.63422400
C	1.23845700	-0.40284200	-1.00004800

C	1.05413100	0.98644400	-1.26429200
C	2.44400400	-1.06232000	-0.38216200
C	2.00469800	2.13268000	-1.03902700
H	-0.54115900	2.08327600	-2.39246500
H	0.01616800	-2.18147200	-1.59482000
O	1.38138400	3.76612900	1.06592100
O	3.25631100	-2.49617700	-2.54130300
C	2.84194000	2.12725400	0.21857500
C	2.48721400	3.01409800	1.28752200
C	3.24135800	3.07379800	2.44024800
C	4.35327600	2.21067000	2.61478800
C	5.09456900	2.19129300	3.82739700
C	6.14705600	1.31729500	4.00082300
C	6.50813000	0.42309800	2.96383100
C	5.81226800	0.42467400	1.77204800
C	4.72108300	1.31166200	1.56379900
C	3.96352700	1.32158800	0.34509600
C	0.88284300	4.58758900	2.09713100
C	3.68410600	-0.66245100	-1.14242500
C	4.40052300	0.47219100	-0.80594800
C	5.54693900	0.86630500	-1.57763800
C	6.30252800	2.03388900	-1.28398900
C	7.39974200	2.38133300	-2.04592000
C	7.79025400	1.57589500	-3.14167100
C	7.07064900	0.44260400	-3.45880600
C	5.93409000	0.05936300	-2.69603700
C	5.17208600	-1.09410100	-3.03162300
C	4.06535900	-1.43836100	-2.28352800
C	3.53164700	-3.32004700	-3.65117500
H	2.96847200	3.73989100	3.25734100
H	4.80319300	2.87454600	4.62905600
H	6.70296200	1.30965100	4.94153800
H	7.33964300	-0.27015300	3.10931900
H	6.08941100	-0.26542400	0.97412600
H	1.58604900	5.40426900	2.33988500
H	0.67684200	4.00516500	3.00985600
H	-0.05830500	5.01218700	1.72606500
H	6.00532500	2.65817800	-0.44062200
H	7.96911900	3.28140000	-1.80278700
H	8.66141700	1.85600300	-3.73881000
H	7.36461700	-0.17864700	-4.30888100
H	5.47860500	-1.68444100	-3.89466500
H	4.51849400	-3.80746900	-3.55964200
H	3.50290900	-2.74941900	-4.59620600

H	2.74851700	-4.08885600	-3.67040100
H	2.30604600	-2.14589600	-0.42066400
H	2.56585700	-0.78611800	0.66941200
H	2.68443800	2.12855300	-1.90928800
H	1.42573300	3.06008900	-1.08675000
H	-1.56718200	-0.40658500	-2.87623900
N	-1.41543400	-1.32845000	1.18040500
Rh	-0.85110500	0.20288000	-0.11454200
C	-2.68411100	-1.81349300	1.07759800
C	-3.52099800	-0.93637300	0.22985200
C	-2.87078900	0.11328500	-0.42634500
C	-4.89600600	-1.14516900	0.07670700
C	-3.60898100	0.95428400	-1.26118700
C	-5.63313500	-0.28964200	-0.73866400
H	-5.36582000	-1.97727800	0.60433500
C	-4.98539000	0.75463400	-1.41038100
H	-3.12655500	1.77833200	-1.78802100
H	-6.70867100	-0.43525600	-0.85974200
H	-5.55784800	1.42488600	-2.05710700
O	-3.07952100	-2.85716800	1.61593800
N	-0.55788100	-2.07899000	1.94692100
C	-0.44007400	-1.98398500	3.32564800
C	0.21678600	-3.13751300	1.50932200
C	0.44508000	-2.92366300	3.79160800
C	0.89337000	-3.67264300	2.65065000
H	0.74167700	-3.06129400	4.82841100
C	1.75773400	-4.75016100	2.41626500
C	1.87089700	-5.22699300	1.10902200
C	1.11823900	-4.63567300	0.07896700
H	2.31572800	-5.21375300	3.23374300
H	2.52320500	-6.07108900	0.87784700
H	1.17057300	-5.02847100	-0.94043100
C	-1.49652300	1.97122100	1.06612600
C	-0.61786100	1.33382300	1.70029300
C	0.15932800	1.14614000	2.89915100
C	-0.31726700	1.69713700	4.10811000
C	1.37135900	0.44444300	2.90682400
C	0.42024200	1.55386200	5.28248800
H	-1.27028100	2.23029600	4.11152700
C	2.10710800	0.30340400	4.08028900
H	1.73015900	0.01412300	1.97885400
C	1.63638000	0.85863100	5.27144900
H	0.04273000	1.98246700	6.21408100
H	3.05428900	-0.23774300	4.06014800

H	2.21266300	0.74814100	6.19288900
C	-5.35573900	1.56008000	2.86606900
C	-4.10313000	1.69427500	2.30578300
C	-3.81299300	2.77446800	1.43094800
C	-4.84580700	3.72306200	1.14284300
C	-6.12366700	3.56122600	1.74425600
C	-6.37712600	2.50099100	2.58716900
H	-5.56500600	0.71233000	3.52229300
H	-3.32646500	0.95772700	2.50912600
C	-2.52927700	2.92545600	0.80782100
C	-4.56690800	4.78628400	0.24453400
H	-6.90543400	4.29058300	1.51693400
H	-7.36501900	2.37938600	3.03701700
C	-3.33440900	4.91702500	-0.35844800
C	-2.30902000	3.97892800	-0.08170400
H	-5.35767700	5.50557200	0.01745800
H	-3.16017700	5.72368800	-1.06981000
O	-1.08827700	4.01825600	-0.67399500
C	-0.62487500	5.19170400	-1.31069300
H	-0.97970300	6.08699900	-0.76891700
H	0.47019700	5.16492200	-1.19712900
C	-0.98973600	5.27906200	-2.77713500
C	-0.44139200	6.30946800	-3.55471700
C	-1.85326800	4.35886100	-3.38210200
C	-0.75166000	6.41797700	-4.91051200
H	0.23948100	7.03153800	-3.09405800
C	-2.16286000	4.46515500	-4.74177300
H	-2.29111400	3.55720200	-2.78781700
C	-1.61439300	5.49359400	-5.50956000
H	-0.31575100	7.22490500	-5.50433900
H	-2.83780300	3.73810800	-5.19938800
H	-1.85579500	5.57620300	-6.57168000
H	-1.00562900	-1.22340900	3.85471900
N	0.29521600	-3.59807800	0.26720500
C	-2.59832300	-4.57265300	-0.97785600
H	-1.78249500	-4.11910100	-0.39032400
C	-2.30040100	-6.07685000	-1.01608500
C	-2.51438700	-3.91431200	-2.35882500
O	-3.85470600	-4.36589000	-0.43061300
H	-3.72498700	-3.89203000	0.42622900
F	-3.35551900	-4.44626900	-3.25007600
F	-1.26806400	-4.00045200	-2.87330300
F	-2.80198200	-2.60368500	-2.25890600
F	-3.21827000	-6.76479200	-1.70368200

F	-1.09895100	-6.33659000	-1.57434800
F	-2.27390500	-6.55726000	0.23292800

(S_{CC},S_{NN})-**IM6**

C	0.37721000	1.06043700	-2.53853800
C	-0.03099100	-0.27516700	-2.94947600
C	0.67347100	-1.19697600	-2.17695400
C	1.51979800	-0.45982600	-1.24772500
C	1.39988700	0.95003700	-1.56215700
C	2.64328700	-1.10132600	-0.47905400
C	2.27631700	2.11620600	-1.16307300
H	0.00167300	1.99054600	-2.95846200
H	0.55810300	-2.27753000	-2.15299600
O	1.81228800	3.64757800	1.09469000
O	3.41475700	-2.79605700	-2.48485000
C	3.18087700	2.02504800	0.05043700
C	2.91797900	2.87054300	1.17899000
C	3.75406000	2.86413200	2.27772700
C	4.86990800	1.99413800	2.33736500
C	5.70688500	1.93370500	3.48573500
C	6.77643100	1.06551800	3.53662400
C	7.05597900	0.21701400	2.43794800
C	6.26292500	0.25534800	1.30988700
C	5.15361500	1.14196200	1.22458900
C	4.29960900	1.20343800	0.07277000
C	1.40835900	4.36247200	2.24924700
C	3.88927200	-0.82965000	-1.29035400
C	4.62742500	0.32264200	-1.08791100
C	5.72536100	0.64955000	-1.95142900
C	6.49356300	1.83436000	-1.79118500
C	7.54236200	2.12011200	-2.64130300
C	7.87065900	1.22968500	-3.69139800
C	7.14049600	0.07372200	-3.87503700
C	6.05205500	-0.24741100	-3.01905400
C	5.28411000	-1.43051900	-3.20426700
C	4.22118700	-1.70951800	-2.36952100
C	3.63864800	-3.70830600	-3.53566600
H	3.54979000	3.49812000	3.13803400
H	5.48157300	2.58734800	4.33234200
H	7.40920900	1.02729600	4.42664100
H	7.90147200	-0.47293300	2.48616100
H	6.48054400	-0.40430700	0.46968200
H	2.11361800	5.18173200	2.47566800
H	1.33205300	3.69325800	3.11887400

H	0.41424300	4.75961000	2.02701500
H	6.24142400	2.51884900	-0.98014500
H	8.12264000	3.03536000	-2.50393600
H	8.70431800	1.46142000	-4.35865300
H	7.38923100	-0.61421700	-4.68740700
H	5.55050100	-2.09812500	-4.02300400
H	4.63584900	-4.17681500	-3.46115900
H	3.54780900	-3.21911600	-4.52154600
H	2.86819100	-4.48496100	-3.44545200
H	2.45177300	-2.17227900	-0.38849300
H	2.74890100	-0.68329000	0.52642400
H	2.92329100	2.29488500	-2.04056400
H	1.63420900	2.99843000	-1.05588100
H	-0.81831700	-0.49747000	-3.66805400
N	-1.49078200	-1.30356900	0.39762200
Rh	-0.55437200	0.10413600	-0.78849200
C	-2.80637500	-1.16960700	0.20115000
C	-3.02441900	-0.20145100	-0.94885800
C	-2.73382700	1.19813800	-0.84638500
C	-3.61012700	-0.71158900	-2.13070100
C	-3.03981000	2.02075300	-1.96219700
C	-3.88309200	0.12180200	-3.20258200
H	-3.85512300	-1.77286300	-2.17105400
C	-3.59268800	1.49719100	-3.11669800
H	-2.84574900	3.08848700	-1.88264100
H	-4.33770300	-0.28738000	-4.10784700
H	-3.81542800	2.16055500	-3.95508900
O	-3.74087500	-1.71752200	0.79711800
N	-1.02016000	-1.98709200	1.48538200
C	-1.27506700	-1.68762800	2.81963100
C	-0.02370800	-2.94089400	1.41920400
C	-0.46422600	-2.44620100	3.62529900
C	0.35316300	-3.25383500	2.76126700
H	-0.45790000	-2.43212700	4.71219300
C	1.34562400	-4.22936600	2.91517800
C	1.87285600	-4.81108900	1.76105900
C	1.39814200	-4.42093400	0.49590900
H	1.68938400	-4.53468400	3.90649200
H	2.64368900	-5.58104700	1.82814300
H	1.80567100	-4.88404500	-0.40827300
C	-2.22279300	1.81879200	0.42678000
C	-1.03825800	1.29132400	0.81016900
C	-0.35790500	1.37085500	2.10190100
C	-0.97827000	1.93747100	3.23941000

C	0.89048500	0.75353100	2.28214900
C	-0.36338900	1.88535200	4.48827300
H	-1.95967200	2.40005000	3.14406900
C	1.51166000	0.70623000	3.52852000
H	1.36292700	0.28982200	1.42210000
C	0.88648900	1.27101800	4.64162400
H	-0.86806000	2.32102500	5.35443700
H	2.48403500	0.21982600	3.62783200
H	1.36191100	1.22660000	5.62438900
C	-6.17687400	1.28142200	2.12948600
C	-4.90023900	1.44971800	1.63431900
C	-4.32416200	2.74566400	1.51986700
C	-5.09729200	3.87094400	1.96180600
C	-6.40985600	3.66367500	2.46587000
C	-6.94512900	2.39582400	2.54445000
H	-6.59081500	0.27323500	2.20328100
H	-4.33394400	0.56951500	1.33953100
C	-3.00285700	2.94950800	0.99559700
C	-4.51867300	5.16620000	1.89812000
H	-6.98630900	4.53225400	2.79538300
H	-7.95505500	2.24713700	2.93364000
C	-3.24278100	5.34872100	1.41831100
C	-2.48393300	4.24162600	0.95578000
H	-5.09893800	6.02294200	2.25002600
H	-2.80754300	6.34842000	1.39918200
O	-1.19909800	4.40166000	0.51544800
C	-0.85359200	5.50367500	-0.32513400
H	-1.21548400	6.45457800	0.09803200
H	0.24417100	5.52101700	-0.31014200
C	-1.35608000	5.31937500	-1.73421800
C	-2.66132900	5.68887600	-2.09114500
C	-0.53833900	4.71049200	-2.69621900
C	-3.14221000	5.44074800	-3.37963100
H	-3.31083200	6.16251900	-1.35208600
C	-1.01362800	4.46549600	-3.98588500
H	0.48034100	4.42393700	-2.42465600
C	-2.32025800	4.82656100	-4.32878000
H	-4.16310600	5.72719900	-3.64235200
H	-0.36411200	3.99302200	-4.72678700
H	-2.69515600	4.63463800	-5.33690200
H	-2.02400500	-0.94223100	3.06739700
N	0.45606300	-3.48983600	0.30990500
C	-2.72666400	-4.79236800	0.26668900
H	-1.91700400	-4.08787600	0.00664500

C	-2.02046200	-6.05675100	0.76834700
C	-3.49553600	-5.02729000	-1.04325600
O	-3.56702800	-4.34853300	1.26932400
H	-3.66268300	-3.36857300	1.19643900
F	-4.40526200	-6.00024000	-0.95739500
F	-2.66716800	-5.32249500	-2.06025700
F	-4.14880500	-3.89530900	-1.38107300
F	-2.87856200	-7.03696100	1.07064700
F	-1.16394000	-6.53327300	-0.15478000
F	-1.31438100	-5.77909800	1.87054400

(R_{CC},S_{NN})-**TS3**

C	0.34688300	2.94099500	2.11617200
C	-0.46858200	3.60689500	1.13242200
C	0.12735100	3.38901900	-0.14224100
C	1.23467200	2.50911800	0.02334500
C	1.38954000	2.26119700	1.45747100
C	2.18541900	2.09095500	-1.06682300
C	2.53044100	1.56832700	2.15453800
H	0.16164200	2.91427800	3.18854800
H	-0.22932000	3.78183400	-1.08980800
O	1.73833200	-0.87468000	3.12027900
O	3.16225300	4.53558400	-1.76640200
C	3.01678500	0.28937000	1.51547500
C	2.56072000	-0.96126000	2.04411900
C	2.96426000	-2.15276600	1.47872100
C	3.81685000	-2.15971700	0.34453200
C	4.13675700	-3.37232200	-0.32555800
C	4.93811700	-3.36321000	-1.44678200
C	5.45763900	-2.14327900	-1.94656900
C	5.15363600	-0.94991400	-1.32451700
C	4.32073700	-0.92251800	-0.17221100
C	3.91058600	0.30159700	0.45339800
C	1.43709300	-2.06026500	3.83723200
C	3.61046100	2.46948500	-0.74205800
C	4.43814100	1.61276000	-0.03603400
C	5.78285900	2.00219800	0.28464900
C	6.66311500	1.16347600	1.02101300
C	7.95067400	1.56605700	1.31129200
C	8.41588200	2.83129400	0.88064800
C	7.58207300	3.67274000	0.17404700
C	6.24967700	3.28849800	-0.13849800
C	5.37316100	4.15893000	-0.84381500
C	4.08185200	3.76449600	-1.12563500

C	3.51523500	5.84327800	-2.14935400
H	2.59170600	-3.10433100	1.84640500
H	3.69940100	-4.30146900	0.04098900
H	5.16338700	-4.30014400	-1.96170600
H	6.09207900	-2.14739900	-2.83618400
H	5.53941300	-0.00930700	-1.72058300
H	0.85500500	-2.76336300	3.22516800
H	0.83653800	-1.74858800	4.70094200
H	2.36110700	-2.55275700	4.18628900
H	6.30632200	0.18797000	1.35242400
H	8.61390000	0.90620800	1.87514900
H	9.43748000	3.14205100	1.11261100
H	7.93575200	4.65369300	-0.15441700
H	5.74578300	5.13846600	-1.14217200
H	4.34280300	5.84600000	-2.88107300
H	3.81045900	6.45600100	-1.27901500
H	2.62321000	6.28495500	-2.61299400
H	1.87581500	2.57831200	-1.99886800
H	2.12696900	1.01067600	-1.22425700
H	3.36555600	2.28720300	2.19250000
H	2.22572300	1.36251800	3.18733700
H	-1.35706300	4.20393900	1.32007100
N	-0.39651500	-0.62754400	0.52619300
C	-0.93343900	-1.39992100	1.49928400
C	-1.68967900	-0.59745100	2.48571400
C	-2.05453900	0.71662500	2.12141800
C	-2.02518600	-1.11014500	3.74252500
C	-2.68851400	1.52991900	3.08079100
C	-2.66866700	-0.30005400	4.67594300
H	-1.75676900	-2.14426000	3.96599000
C	-2.97953500	1.02892300	4.34655200
H	-2.98240200	2.54649200	2.81457900
H	-2.92720900	-0.69422600	5.66124300
H	-3.47266400	1.67149500	5.08034000
O	-0.84042700	-2.63910800	1.58528800
N	-0.12284800	-1.29033000	-0.64817400
C	1.08325000	-1.30135800	-1.32230000
C	-1.06909100	-1.95786200	-1.41044400
C	0.94468800	-1.95263000	-2.52453900
C	-0.42195700	-2.38488900	-2.60939300
H	1.74384100	-2.13380700	-3.23868400
C	-1.21858100	-3.07806800	-3.52983400
C	-2.55628200	-3.29527000	-3.20071000
C	-3.06542900	-2.82440800	-1.97502800

H	-0.80401800	-3.44312700	-4.47261300
H	-3.21686900	-3.83468800	-3.88241700
H	-4.11640300	-2.99272900	-1.71499400
H	1.95933400	-0.88002600	-0.85138300
N	-2.33588600	-2.15917500	-1.07759900
Rh	-0.63980300	1.45856400	0.69313100
C	-2.88596100	1.00946300	0.38393400
C	-2.08649800	1.02748200	-0.64023500
C	-2.13171600	0.91379000	-2.07789500
C	-3.33620300	0.54146400	-2.71193900
C	-0.99005600	1.13525900	-2.86553500
C	-3.39366400	0.42233700	-4.09732600
H	-4.21589400	0.33391100	-2.10092900
C	-1.05039900	1.00847600	-4.25225300
H	-0.05157300	1.38293200	-2.37614400
C	-2.25181300	0.65795500	-4.87398600
H	-4.32989900	0.12824400	-4.57743800
H	-0.14941900	1.16869200	-4.84883100
H	-2.29736100	0.55066800	-5.96024900
C	-5.69869000	-2.08050100	1.38805100
C	-4.79452700	-1.09818100	1.04002700
C	-5.19311900	0.26681800	1.02464500
C	-6.55131600	0.60134200	1.33566000
C	-7.45309700	-0.43801800	1.69364300
C	-7.03470200	-1.75123500	1.72793000
H	-5.37897100	-3.12522400	1.39457300
H	-3.78195600	-1.36524400	0.73228200
C	-4.28187700	1.31120100	0.66881800
C	-6.95523100	1.96187500	1.26386800
H	-8.48658900	-0.17660200	1.93585400
H	-7.73509700	-2.54278100	2.00475800
C	-6.07305300	2.95546100	0.89743000
C	-4.72494800	2.63049000	0.59942500
H	-7.98985300	2.21702200	1.50717200
H	-6.40024300	3.99554500	0.85867300
O	-3.79881900	3.58011000	0.29728300
C	-4.17317200	4.70681000	-0.49697800
H	-5.03203100	4.43660400	-1.13304700
H	-4.47678600	5.54496100	0.15420400
C	-2.99276900	5.09376600	-1.34990600
C	-2.67016500	4.32794900	-2.47995000
C	-2.17571100	6.17766000	-1.00578900
C	-1.55464300	4.64606200	-3.25577700
H	-3.28561900	3.46686800	-2.74779800

C	-1.05768500	6.49974600	-1.78108400
H	-2.41518500	6.77408400	-0.12115100
C	-0.74713200	5.73468500	-2.90839500
H	-1.31344600	4.03252200	-4.12547200
H	-0.42668300	7.34642900	-1.50096500
H	0.12727000	5.98149700	-3.51532200
C	0.38258800	-5.34858600	0.44002100
H	-0.67385800	-5.38179300	0.76440900
C	0.44704400	-6.08613900	-0.90380600
C	1.18554600	-6.05583600	1.54532000
O	0.85004000	-4.06780000	0.25522500
H	0.21754500	-3.43669500	0.70866400
F	0.66387600	-7.24643500	1.86986300
F	1.18309600	-5.29786900	2.65862300
F	2.46952200	-6.25015500	1.20124700
F	0.13663200	-7.38906600	-0.77323200
F	-0.43139800	-5.54528000	-1.75733600
F	1.66177600	-6.00936700	-1.45831500

(R_{CC},S_{NN})-**IM5**

C	0.12676300	3.00967600	1.90415000
C	-0.60611600	3.66894400	0.88144200
C	0.04762700	3.36988000	-0.36312900
C	1.15164500	2.52108300	-0.11620100
C	1.18925100	2.25689700	1.31053400
C	2.17379900	2.09356200	-1.13431400
C	2.27363900	1.57543400	2.10708200
H	-0.09097400	3.03094100	2.96923400
H	-0.26055100	3.73809800	-1.33837200
O	1.52216400	-0.88700900	3.07171000
O	3.17433300	4.54683400	-1.77114300
C	2.83416900	0.30343800	1.51489700
C	2.38142100	-0.95681900	2.02582200
C	2.82682700	-2.14120000	1.47538600
C	3.74049400	-2.13192600	0.38981900
C	4.12154300	-3.33686500	-0.26216300
C	5.00200500	-3.31236100	-1.32239700
C	5.54067500	-2.08370400	-1.77875800
C	5.17610600	-0.89730400	-1.17640100
C	4.26224400	-0.88617800	-0.08686300
C	3.79556400	0.32970600	0.51412500
C	1.22886600	-2.07393100	3.79119600
C	3.56553900	2.49097900	-0.70487400
C	4.34392100	1.64700600	0.06732600

C	5.65371500	2.05329200	0.49372700
C	6.48274900	1.22666300	1.29983200
C	7.73711000	1.64715700	1.69238600
C	8.21829300	2.91827800	1.29828600
C	7.43327300	3.74755500	0.52454300
C	6.13599500	3.34493500	0.10588500
C	5.30827500	4.20240800	-0.67096500
C	4.04918000	3.79080100	-1.05511100
C	3.54336500	5.85534000	-2.13785300
H	2.45263000	-3.09907900	1.82577500
H	3.67377800	-4.27391200	0.07100100
H	5.27897700	-4.24402900	-1.82149200
H	6.23905400	-2.07583800	-2.61904600
H	5.57783200	0.05000600	-1.53925700
H	0.68592600	-2.79699100	3.16648500
H	0.58999200	-1.77034200	4.62942200
H	2.15526000	-2.53615400	4.17356500
H	6.11371600	0.24638000	1.60288800
H	8.36151800	0.99702600	2.30935400
H	9.21331500	3.24326500	1.61182800
H	7.79924800	4.73291700	0.22402900
H	5.69133900	5.18611500	-0.94124600
H	4.42945200	5.86022800	-2.79736100
H	3.75760400	6.48006700	-1.25252400
H	2.68884800	6.28130000	-2.68026400
H	1.93318400	2.57257300	-2.09021600
H	2.14001900	1.01077100	-1.28530900
H	3.09086300	2.30696900	2.22385800
H	1.87861400	1.36254200	3.10735800
H	-1.49104500	4.28563000	1.00499500
N	-0.55378400	-0.57952900	0.37671000
C	-0.97009100	-1.39755000	1.37615800
C	-1.62536400	-0.64246200	2.46079900
C	-1.72070000	0.74223200	2.29192400
C	-2.20823400	-1.28085300	3.56128600
C	-2.40545700	1.49759800	3.24789900
C	-2.88885500	-0.52472000	4.51316900
H	-2.13633000	-2.36823100	3.63080100
C	-2.98339400	0.86393700	4.35342400
H	-2.52658900	2.57562000	3.12731100
H	-3.35992600	-1.01098600	5.37027900
H	-3.52583800	1.46161600	5.09075300
O	-0.84602100	-2.63511600	1.38715800
N	-0.22822100	-1.21126300	-0.79800200

C	1.03119900	-1.27300700	-1.37093100
C	-1.12065300	-1.89615900	-1.60776700
C	0.97493200	-1.96038000	-2.55835200
C	-0.38870500	-2.37276200	-2.73755800
H	1.82440700	-2.17985300	-3.20001500
C	-1.11998900	-3.08781600	-3.69411400
C	-2.48087100	-3.28369700	-3.46162700
C	-3.07589200	-2.76911300	-2.29455400
H	-0.63768100	-3.48811800	-4.58910000
H	-3.09241200	-3.84354500	-4.17188300
H	-4.14452000	-2.92369700	-2.10898500
H	1.87505000	-0.85445200	-0.84193200
N	-2.41063600	-2.07605600	-1.36803100
Rh	-0.83272500	1.47302000	0.60294800
C	-2.96106700	1.49289000	-0.08700300
C	-2.19715400	1.24211700	-1.05035200
C	-1.95124600	0.94796100	-2.43925300
C	-3.03854400	0.53176300	-3.23473500
C	-0.68637100	1.07496900	-3.02937200
C	-2.85648700	0.26731000	-4.58880500
H	-4.01694400	0.40178700	-2.76987800
C	-0.50691900	0.80237000	-4.38421600
H	0.15801600	1.36420000	-2.41449000
C	-1.58969700	0.40255300	-5.17004000
H	-3.70345900	-0.06441500	-5.19346700
H	0.48978700	0.88780000	-4.82214200
H	-1.44752200	0.18036800	-6.23004100
C	-5.21837600	-1.85770900	1.33575100
C	-4.50256900	-0.78641900	0.84205100
C	-4.87406000	0.53992900	1.18168600
C	-6.00528000	0.75172900	2.03447300
C	-6.72113300	-0.37558700	2.52123800
C	-6.33417500	-1.65527100	2.18305900
H	-4.90606900	-2.87102400	1.07274700
H	-3.64901300	-0.97098300	0.18665200
C	-4.13533200	1.67937400	0.70891600
C	-6.37289400	2.08104000	2.37987700
H	-7.58191700	-0.20831600	3.17388700
H	-6.88782800	-2.51477400	2.56861200
C	-5.65651700	3.16125900	1.91377700
C	-4.52912400	2.96184500	1.08266000
H	-7.23483300	2.23598900	3.03382300
H	-5.92638400	4.18252800	2.18963200
O	-3.77686200	4.03479500	0.68918500

C	-4.28981500	4.86903800	-0.36604400
H	-5.20561400	4.41486400	-0.77448300
H	-4.55067000	5.85107300	0.06266300
C	-3.24774700	5.02070600	-1.44348600
C	-3.17531700	4.09458300	-2.49259700
C	-2.29507200	6.04650500	-1.37740000
C	-2.16884700	4.18325500	-3.45574800
H	-3.90663400	3.28629200	-2.55088700
C	-1.28739800	6.14300700	-2.34065000
H	-2.34122000	6.77340400	-0.56185500
C	-1.22194000	5.20967400	-3.38031500
H	-2.11849600	3.44233000	-4.25553100
H	-0.54962100	6.94654000	-2.27808200
H	-0.43160600	5.27990700	-4.13148800
C	0.23513900	-5.34316300	0.38912800
H	-0.74136700	-5.23900000	0.89525800
C	-0.02794700	-6.13233400	-0.89973900
C	1.14699500	-6.08653400	1.37622300
O	0.79514700	-4.12635900	0.06293200
H	0.21386800	-3.42588300	0.47213700
F	0.61165400	-7.24143200	1.79465900
F	1.34901700	-5.31940500	2.46648200
F	2.35288400	-6.35830200	0.85344600
F	-0.52137800	-7.35773000	-0.64416800
F	-0.93111000	-5.48091500	-1.64453700
F	1.08012900	-6.28251600	-1.63299100

(R_{CC},S_{NN})-**IM6**

C	0.66172000	2.79331200	2.40988300
C	-0.22345500	3.54429600	1.53666700
C	0.24696600	3.37922000	0.21566200
C	1.35552500	2.45821400	0.24579300
C	1.65364400	2.16156200	1.64613900
C	2.21207200	2.09211500	-0.93922600
C	2.82552800	1.39836600	2.20891200
H	0.54616200	2.69655100	3.48905600
H	-0.17698800	3.82445800	-0.68034600
O	2.02769100	-1.09638700	3.07275600
O	3.16924300	4.57011500	-1.57683200
C	3.22076200	0.14687600	1.45793500
C	2.77106500	-1.12570900	1.93601600
C	3.10302000	-2.28688500	1.26887300
C	3.87810800	-2.23961000	0.08231800
C	4.12889500	-3.41564500	-0.67634900

C	4.84873600	-3.35332100	-1.85018100
C	5.34900000	-2.11245600	-2.31767200
C	5.10948100	-0.95290300	-1.60943300
C	4.36464500	-0.98155300	-0.39833900
C	4.02728800	0.20730600	0.32920500
C	1.70784700	-2.33149300	3.69169300
C	3.66328500	2.44236600	-0.70658900
C	4.53474000	1.53792000	-0.12331600
C	5.90658700	1.89264400	0.10796600
C	6.83086900	1.00207400	0.71894100
C	8.14335000	1.37398500	0.92655300
C	8.59042300	2.65872200	0.53580300
C	7.71477900	3.54947600	-0.04913100
C	6.35622300	3.19716000	-0.27504100
C	5.43832000	4.11534200	-0.85527900
C	4.12221300	3.75215300	-1.05425700
C	3.52678500	5.88365000	-1.93778200
H	2.71642600	-3.24955900	1.58898700
H	3.71011300	-4.36018100	-0.32481800
H	5.02555800	-4.26217700	-2.43025000
H	5.91781900	-2.07346900	-3.24969600
H	5.48054400	0.00416700	-1.97912000
H	1.01126900	-2.91968000	3.08027100
H	1.23688600	-2.08538800	4.65225900
H	2.61670300	-2.92717300	3.88135200
H	6.48780300	0.01203400	1.02047900
H	8.84033100	0.67519500	1.39463200
H	9.63172400	2.94500500	0.70231700
H	8.05498400	4.54505100	-0.34614700
H	5.80070700	5.10639600	-1.12626200
H	4.30107800	5.89369000	-2.72519600
H	3.89614300	6.45711500	-1.06921100
H	2.61735000	6.36252200	-2.32271700
H	1.84037200	2.63124900	-1.81783600
H	2.12858900	1.02041300	-1.14591600
H	3.68792000	2.08474700	2.22921600
H	2.59156200	1.13611100	3.24786800
H	-1.07419300	4.14738400	1.84494500
N	-0.38415200	-0.61756300	0.65447500
C	-1.07305100	-1.16680700	1.66132000
C	-1.58990200	-0.05988200	2.55710500
C	-2.52683500	0.92909200	2.09487800
C	-1.22524700	-0.08455200	3.92464100
C	-3.02466200	1.86137300	3.04873400

C	-1.72223200	0.84609800	4.81713200
H	-0.55229200	-0.87247900	4.25833900
C	-2.63002900	1.82940000	4.37216700
H	-3.74779100	2.60158800	2.70154400
H	-1.42600800	0.80944500	5.86795000
H	-3.03451700	2.55785500	5.07910600
O	-1.27820400	-2.36371000	1.91365500
N	-0.19733300	-1.31954000	-0.50670100
C	0.98567400	-1.36437900	-1.22065800
C	-1.20256800	-1.90512900	-1.26294700
C	0.78119300	-1.98112500	-2.43063700
C	-0.61000700	-2.33510500	-2.48859400
H	1.54842000	-2.17576500	-3.17561600
C	-1.46870800	-2.95521100	-3.40556100
C	-2.80942900	-3.09870400	-3.04865500
C	-3.26047800	-2.63099500	-1.79878500
H	-1.09996600	-3.31632200	-4.36880200
H	-3.51806800	-3.57654900	-3.72816500
H	-4.31239900	-2.74121200	-1.51344700
H	1.88730200	-0.96604500	-0.77866700
N	-2.46921300	-2.03647400	-0.90484400
Rh	-0.46226800	1.42810700	0.97547600
C	-3.06715200	0.98436600	0.69167000
C	-2.08689700	1.16315500	-0.21707400
C	-2.12403200	1.12243300	-1.67648000
C	-3.29493700	0.77325000	-2.38709200
C	-0.95623500	1.34912100	-2.42482400
C	-3.29332600	0.69863800	-3.77748800
H	-4.20439600	0.53353300	-1.84130700
C	-0.94963500	1.26527200	-3.81593800
H	-0.03596500	1.56830200	-1.89638600
C	-2.12293200	0.95064700	-4.50390300
H	-4.21140300	0.41977200	-4.30034800
H	-0.01797400	1.43316500	-4.36152700
H	-2.12419100	0.87872700	-5.59405000
C	-5.73568200	-2.38026900	1.38036400
C	-4.88338400	-1.32120800	1.15011100
C	-5.39503700	-0.05112600	0.75884700
C	-6.81262900	0.09320000	0.58071500
C	-7.66146600	-1.01984100	0.83329300
C	-7.13756600	-2.23077700	1.23030300
H	-5.32066400	-3.34783400	1.67213000
H	-3.80770300	-1.46962700	1.22030600
C	-4.53602200	1.06195100	0.49832500

C	-7.32472800	1.34121500	0.13898300
H	-8.73967400	-0.89695800	0.70001200
H	-7.79839400	-3.08007300	1.41976700
C	-6.48814000	2.40700300	-0.11274100
C	-5.08977500	2.26942100	0.07527600
H	-8.40365100	1.45005000	0.00155900
H	-6.89968200	3.36404600	-0.43639100
O	-4.23335300	3.31322300	-0.11680400
C	-4.47120300	4.22090100	-1.18930900
H	-4.90174100	3.67836400	-2.04657200
H	-5.19060600	4.99947800	-0.87551000
C	-3.15578900	4.84632900	-1.57274800
C	-2.43590900	4.36728400	-2.67376900
C	-2.60900500	5.87882800	-0.79638500
C	-1.19562600	4.91996600	-3.00380700
H	-2.84026500	3.54610700	-3.26800300
C	-1.36895400	6.43165200	-1.12168300
H	-3.16313700	6.25067200	0.06973900
C	-0.66209300	5.95408500	-2.23079100
H	-0.64275400	4.52888100	-3.86037000
H	-0.95197500	7.23582800	-0.51084900
H	0.30821700	6.38448200	-2.48775900
C	0.11025500	-5.27526900	0.82372100
H	-0.92194300	-5.46799400	1.16887700
C	0.19638900	-5.84029800	-0.60278300
C	1.03277800	-6.00217400	1.81608300
O	0.44185100	-3.93935300	0.79275800
H	-0.29569500	-3.37156700	1.16457200
F	0.75435100	-7.30742800	1.91794100
F	0.88710300	-5.46096600	3.03843000
F	2.32889200	-5.88204700	1.47979200
F	-0.02661000	-7.16583300	-0.62976300
F	-0.73238900	-5.25833300	-1.36982600
F	1.39382700	-5.61575600	-1.15768100
